# Author Correction: In vivo CRISPR screens reveal Serpinb9 and Adam2 as regulators of immune therapy response in lung cancer

**DOI:** 10.1038/s41467-023-41255-0

**Published:** 2023-09-05

**Authors:** Dzana Dervovic, Ahmad A. Malik, Edward L. Y. Chen, Masahiro Narimatsu, Nina Adler, Somaieh Afiuni-Zadeh, Dagmar Krenbek, Sebastien Martinez, Ricky Tsai, Jonathan Boucher, Jacob M. Berman, Katie Teng, Arshad Ayyaz, YiQing Lü, Geraldine Mbamalu, Sampath K. Loganathan, Jongbok Lee, Li Zhang, Cynthia Guidos, Jeffrey Wrana, Arschang Valipour, Philippe P. Roux, Jüri Reimand, Hartland W. Jackson, Daniel Schramek

**Affiliations:** 1 Centre for Molecular and Systems Biology, Lunenfeld-Tanenbaum Research Institute, Mount Sinai Hospital, Toronto, ON Canada; 2 Department of Molecular Genetics, University of Toronto, Toronto, ON Canada; 3 Computational Biology Program, Ontario Institute for Cancer Research, Toronto, ON Canada; 4 Department of Pathology and Bacteriology, Klinik Floridsdorf, Vienna, Austria; 5 Institute for Research in Immunology and Cancer (IRIC), Université de Montréal, Montreal, QC Canada; 6 Department of Biological Sciences, University of Calgary, Calgary, AB Canada; 7 Department of Otolaryngology, Head and Neck Surgery, McGill University, Montreal, QC Canada; 8 Toronto General Hospital Research Institute, University Health Network, Toronto, ON Canada; 9 Departments of Laboratory Medicine and Pathobiology, Immunology, University of Toronto, Toronto, ON Canada; 10 SickKids Research Institute, University Health Network, Toronto, ON Canada; 11 Karl-Landsteiner-Institute for Lung Research and Pulmonary Oncology, Klinik Floridsdorf, Vienna, Austria; 12 Department of Pathology and Cell Biology, Faculty of Medicine, Université de Montréal, Montreal, QC Canada

**Keywords:** Non-small-cell lung cancer, Immunotherapy, Tumour immunology, Oncogenes

Correction to: *Nature Communications* 10.1038/s41467-023-38841-7, published online 31 May 2023

The original version of this Article contained an inaccuracy in the Methods section “CyTOF analysis”.

The original sentence “Cells were then washed twice by centrifuging through PBS (800 × *g*, 5′) prior to re-suspending in PBS with 4-element EQ normalization beads (Standard BioTools: 201078)” should have read “Cells were then washed twice by centrifuging through PBS at 800 × *g*, 5’ prior to re-suspending in Maxpar Cell Acquisition Solution (Standard BioTools: 201241) with 4-element EQ normalization beads (Standard BioTools: 201078)”.

This has now been corrected in the PDF and HTML versions of the Article.

