# Supplementary material for: In vivo CRISPR screens reveal Serpinb9 and Adam2 as regulators of immune therapy response in lung cancer
Source: Nat Commun. 2023 May 31;14:3150. doi: 10.1038/s41467-023-38841-7 (PMC10232477; doi:10.1038/s41467-023-38841-7)
Supplement: Supplementary file 1 — Supplementary Information [file 41467_2023_38841_MOESM1_ESM.pdf]

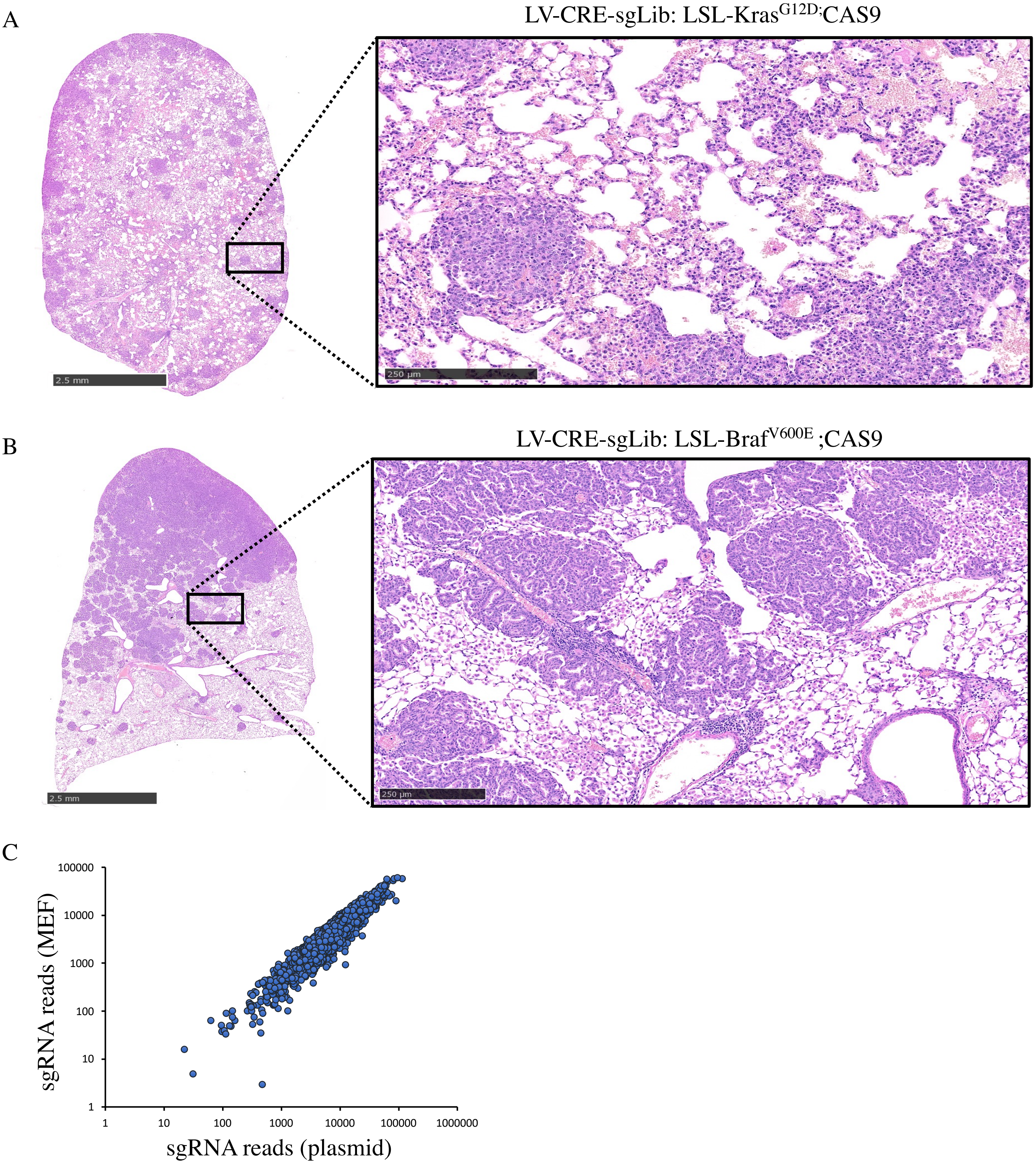


**Supplementary Figure 1: Histological characterization of *Kras*$^{G12D}$;CAS9 and Braf $^{V600E}$;Cas9 lung tumors and sgRNA distribution in CRISPR library**

**A** and **B,** Representative H&E staining of lungs showing hundreds of independent tumors induced by LV-sgNTC-Cre intranasally instilled into the lungs of *Kras*$^{G12D}$;Cas9 (A) and Braf$^{V12E}$;Cas9 (B) mice. Number of mice used >20. The scale bars represents 2.5 mm for the lung lobe and 250 $\mu$m for magnified area in the quadrant respectively. **C,** Graph showing sgRNA representation in plasmid pool (x-axis) and MEFs transduced with the pooled lentiviral library targeting 573 genes (2273 sgRNA, y-axis) confirmed by NGS sequencing.

**Supplementary Figure 1**

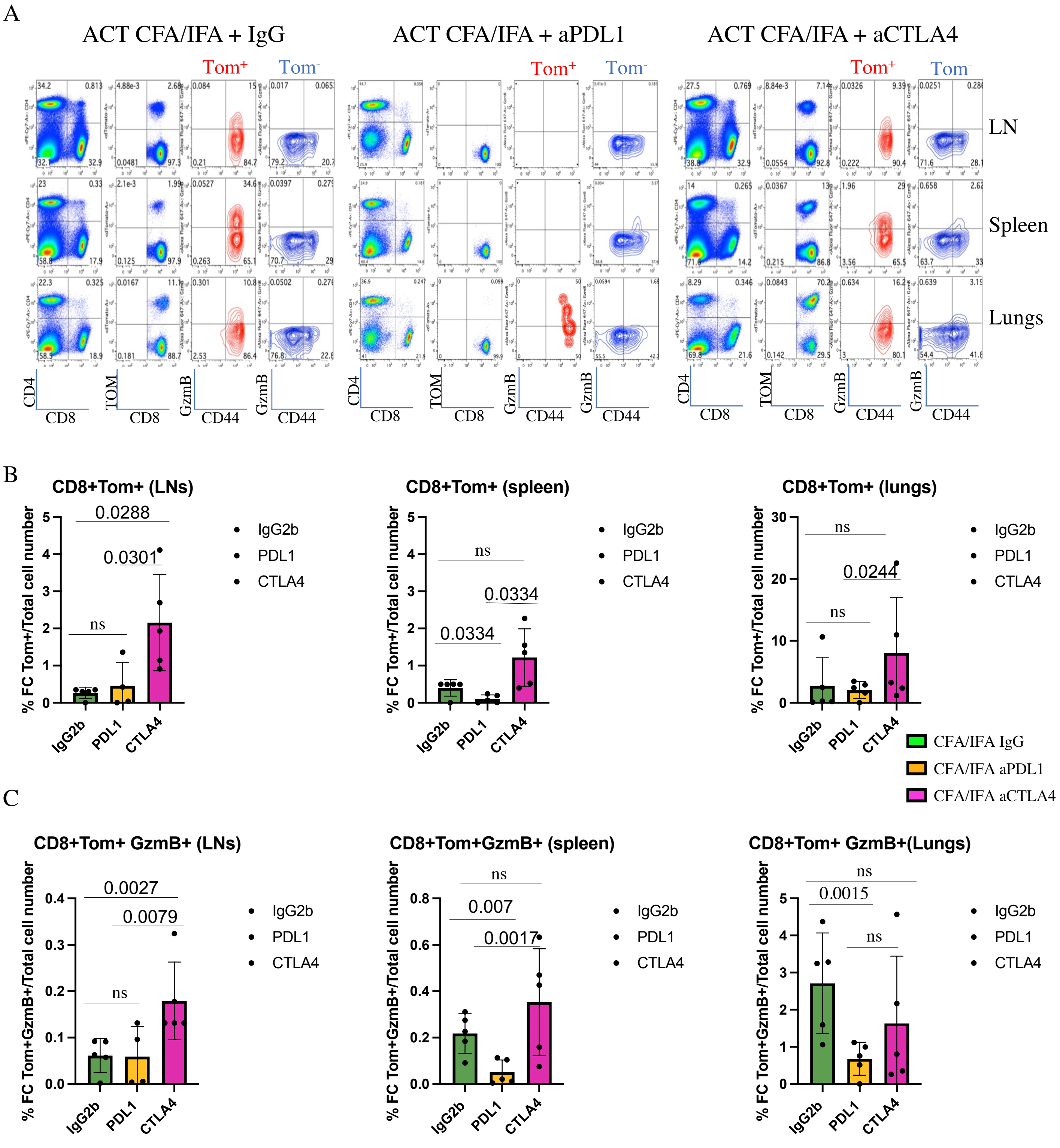


**Supplementary Figure 2: Effect of PD-L1 and CTLA4 blocking antibodies on efficacy of ACT**

**A,** Flow cytometry analysis of CD44 and GzmB expression in endogenous CD8$^+$ Tom$^-$ and exogenous CD8$^+$ Tom$^+$ OT-I T cells T cells isolated from LNs, spleen and lung of Kras$^{G12D}$ lung tumor-bearing mice subjected to ACT of CD8$^+$OT-I cells with IgG2b (*n*=3), anti-PDL1(*n*=4) or anti-CTL4 (*n*=4) treatment examined over two independent experiments. Cells were pre-gated on FSC/SSC, FSCA/FSCH, SSCW/SSSCH, and FSC/DAPI$^-$. **B** and **C,** Fold change of total OT-I (B) and OT-I GzmB$^+$ (**C**) cell numbers gated on CD8$^+$ T cells from mice subjected to different treatments in (A). Data are mean ± s.e.m. Two-sided Student's t-test at indicated time-points ns (not significant).

**Supplementary Figure 2**

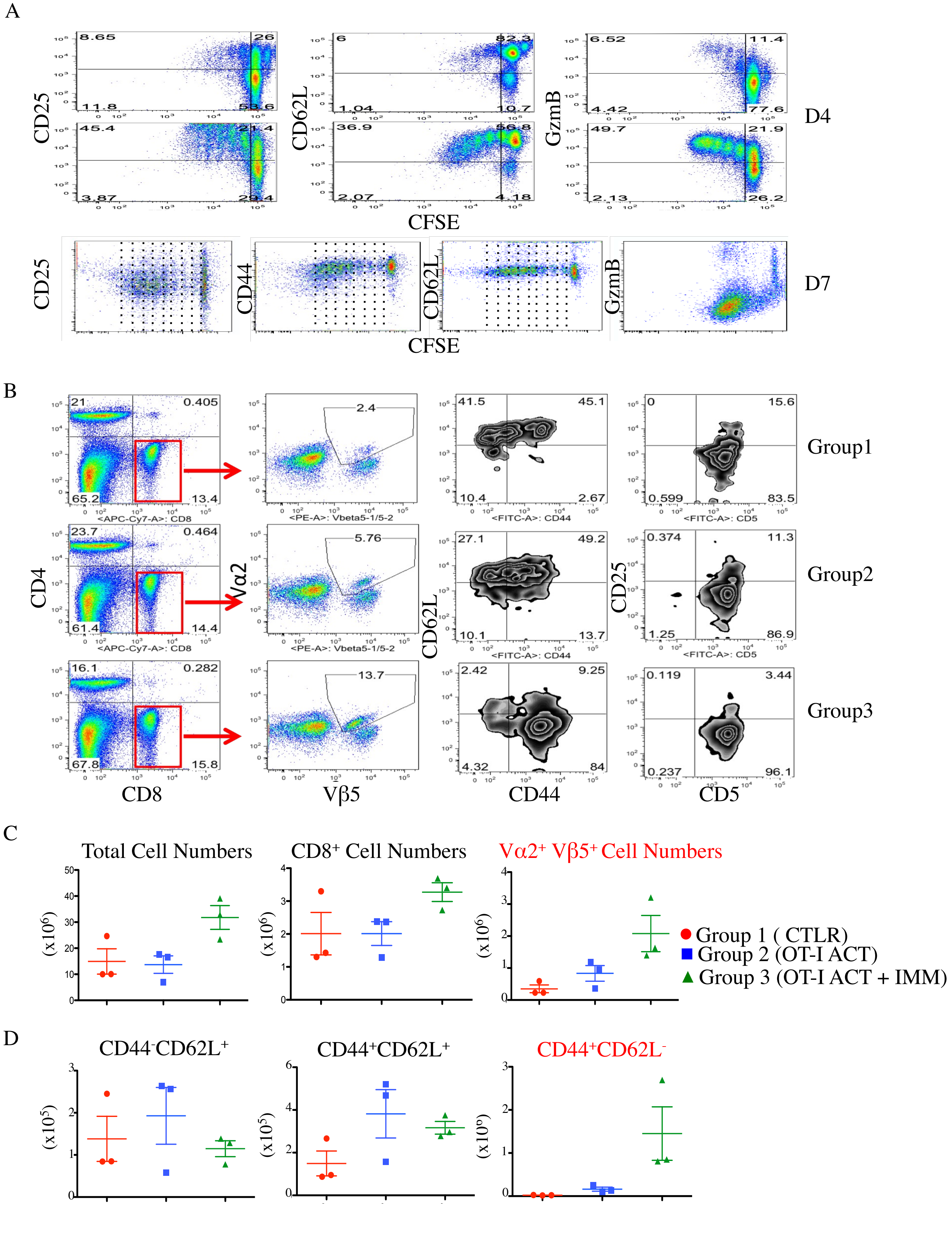


**Supplementary Figure 3**

**Supplementary Figure 3: *In vitro* and *in vivo* activation profile of OT-I CD8 T cells**

**A,** Flow cytometry analysis of column purified, CFSE labeled splenic OT-I CD8 T cells unstimulated or stimulated with anti-CD3/CD28 cells for 4 or 7 days, examined over two independent experiments. Expression profile of CD25, CD62L selectin, CD44 and GzmB is depicted. Cells were pre-gated on FSC/SSC, FSCA/FSCH, SSCW/SSSCH, and FSC/DAPI$^{-}$. CFSE dilution was used to measure T cell proliferation. **B,** Flow cytometry analysis of CD4 and CD8 expression on total T cells isolated from the spleen of C57BL/6 untreated mice (group 1, *n*=3) and after ACT of OT-I T cells in C57BL/6 mice without treatment (group 2, *n*=3) or immunized with SIINFEKL emulsified in CFA on day 1 and IFA on day 7 after ACT (group 3, *n*=3). Expression of OT-I TCR-specific chain Vα2/Vβ5, CD44, CD62L, CD5 and CD25 on gated CD8$^{+}$ T cells is shown. **C,** Total cell numbers, CD8 T cell numbers, numbers of TCR-specific Vα2/Vβ5$^{+}$ CD8 T cells from group 1, 2 and 3 described in (B) were quantified by flow cytometry**.** Data were analysed by two-sided student's t-test and present mean **±** s.e.m. for each group examined. **D,** Total cell numbers of naïve (CD44$^{-}$CD62L$^{+}$), central memory (CD44$^{+}$CD62L$^{+}$), and activated (CD44$^{+}$CD62L$^{-}$) cells gated on CD8$^{+}$ T cells from group 1, 2 and 3 described in (B) quantified by flow cytometry are shown. Results are representative of three independent experiments. Data were analysed by two-sided student's t-test and present mean **±** s.e.m. for each group examined.

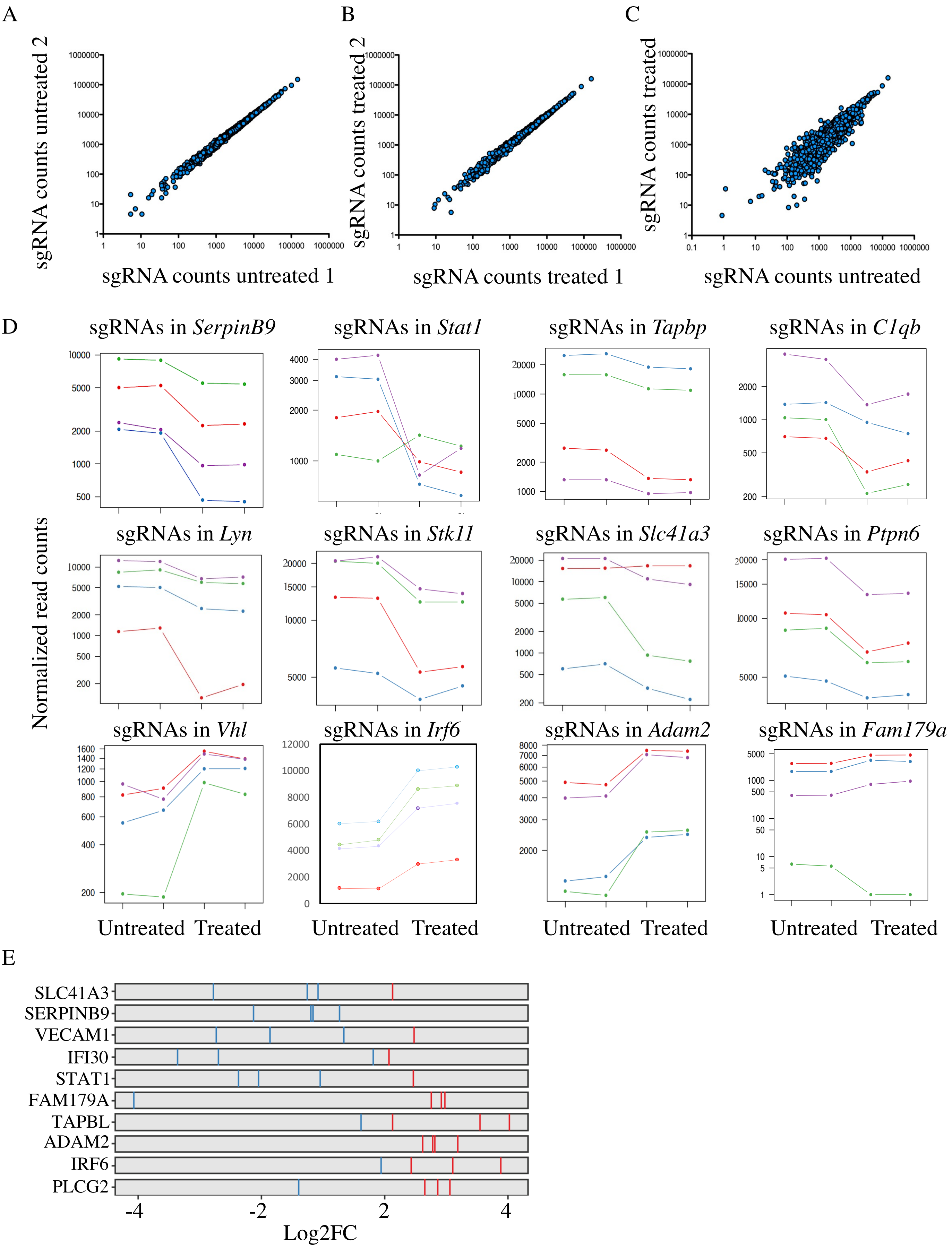


**Supplementary Figure 4**

**Supplementary Figure 4. CRISPR screen hits identified after treatment with OT-I cells in *Kras*$^{G12D}$;CAS9 mice**

**A-C** Graph showing sgRNA representation from two groups of 5 untreated lungs (A), two groups of 5 treated lungs with ACT of OT-I cells (B) and sgRNA representation between untreated versus treated lungs with ACT of OT-I cells (C) isolated from *Kras*$^{G12D}$;CAS9 mice transduced with sgRNA library at P2. Experimental data are representative of two independent experiments. **D,** MAGeCK analysis graphs showing RRA scores for top depleted/enriched hits obtained from lungs of untreated and treated *Kras*$^{G12D}$;CAS9 mice with ACT of OT-I cells transduced with sgRNA library (targeting 573 genes=2273 sgRNA) at P2. A comprehensive CRIPSR screen analysis was obtained by use of MAGeCK (Model-based Analysis of Genome-wide CRISPR-Cas9 Knockout) algorithm. Each color represents different sgRNA. The most efficient sgRNAs based on MAGeck RRA score, which ranks sgRNA efficiency based on their p-values calculated from the negative binomial model and uses a modified algorithm named alpha-RRA to identify positively or negatively selected genes, were chosen for follow up. The RRA scores are similar to log fold change in differential expression allowing a direct comparison across multiple conditions (efficiency of sgRNAs in untreated vs treated lungs were compared). **E,** MAGeCK Flute analysis showing top five depleted and top five enriched hits obtained from lungs of untreated and treated *Kras*$^{G12D}$;CAS9 mice with ACT of OT-I cells, transduced with sgRNA library (targeting 573 genes=2273 sgRNA) at P2.

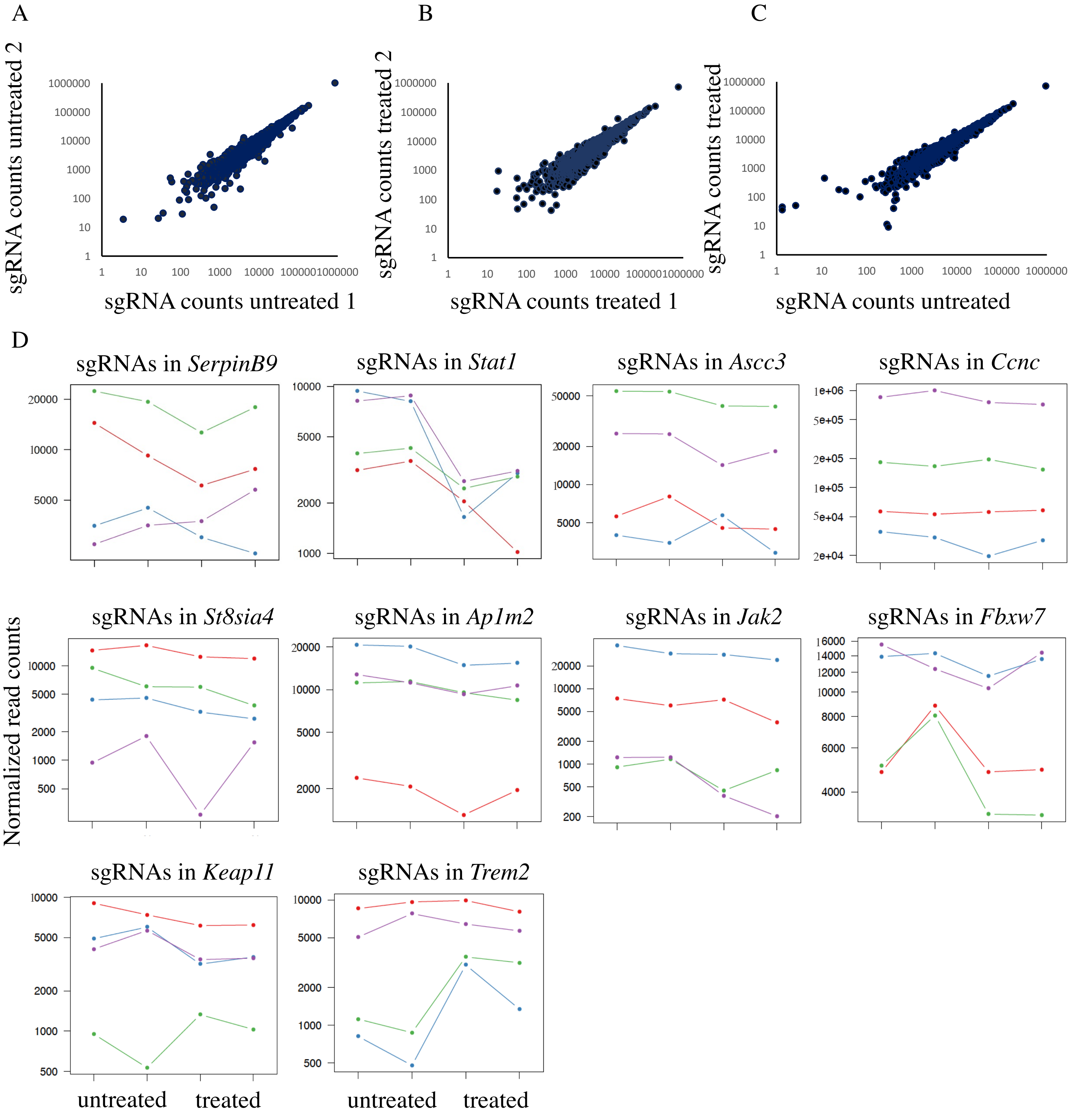


**Supplementary Figure 5: CRISPR screen hits identified after treatment with OT-I cells in *Braf*$^{V600E}$;CAS9 mice**

**A-C** Graph showing sgRNA representation from two groups of 5 untreated lungs (A), two groups of 5 treated lungs with ACT of OT-I cells (B), and sgRNA representation between untreated versus treated lungs with ACT of OT-I cells (C) isolated from lungs of *Braf*$^{V600E}$;CAS9 mice transduced with sgRNA library at P2. Experimental data are representative of two independent experiments. **D,** MAGeCK analysis graphs showing RRA scores for top depleted/enriched hits obtained from lungs of untreated and treated *Braf*$^{V600E}$;CAS9 mice with ACT of OT-I cells, transduced with sgRNA library at P2.

**Supplementary Figure 5**

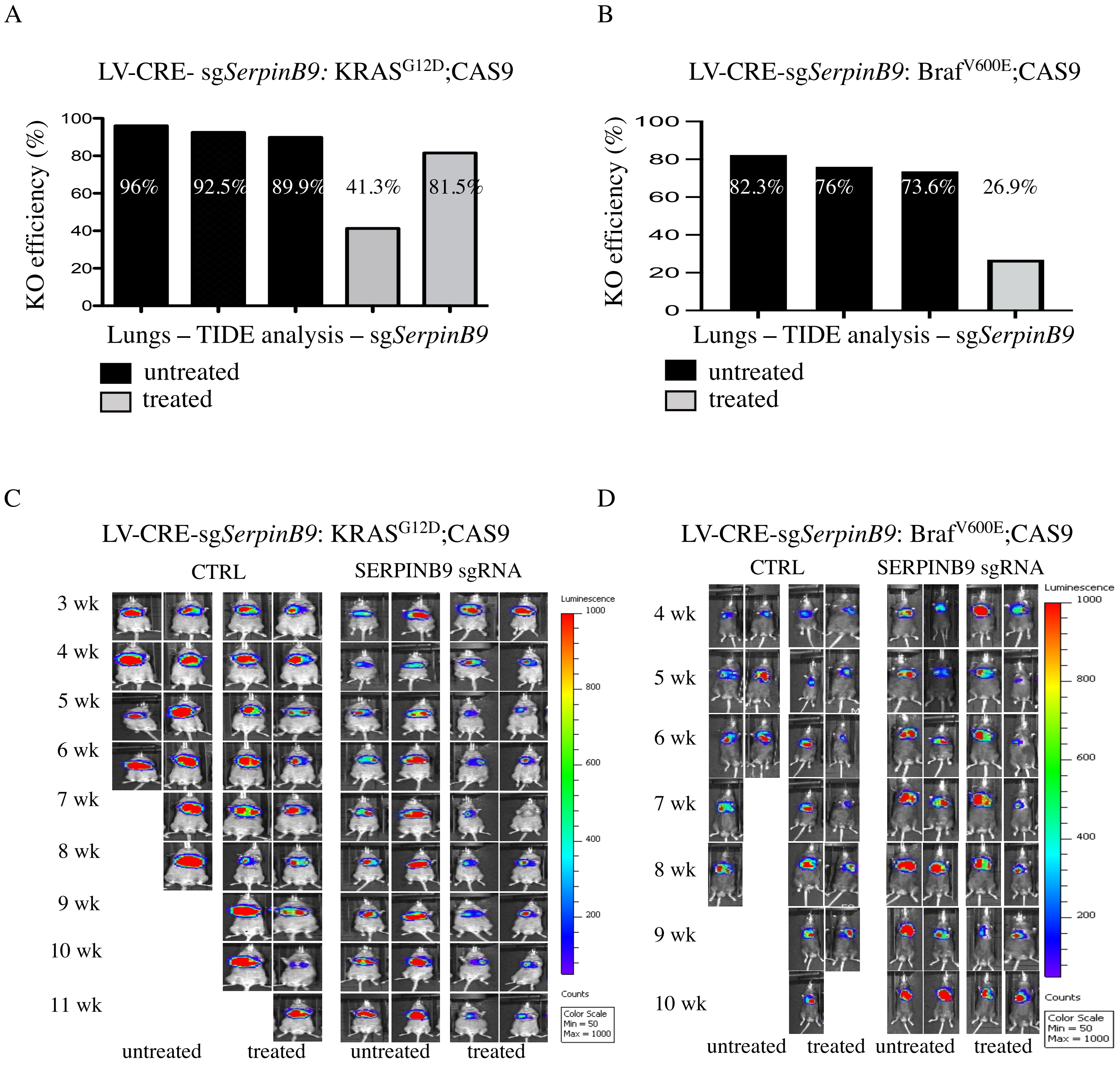


**Supplementary Figure 6: SERPINB9 restrains tumor growth in mouse models of lung cancer**

**A** and **B,** Gene editing efficiency of *Serpinb9* was determined using Sanger sequencing of PCR-amplified sgRNA target sites followed by Tracking of Indels by Decomposition (TIDE) algorithm from bulk or GFP sorted tumor cells isolated from five untreated or treated biologically independent lungs of *Kras*$^{G12D}$;CAS9 mice (A) or from four untreated or treated lungs of *Braf*$^{V600E}$;CAS9 mice (B) transduced with LV-sg*SerpinB9*-Cre at P2. **C** and **D,** Representative images of untreated or ACT-treated *Kras*$^{G12D}$;CAS9 mice (C) and *Braf*$^{V600E}$;CAS9 mice (D) inhaled at P2 with sgNTC or sg*Serpinb9* measured by BLI once a week. The pseudocolor images (rainbow color scale) were adjusted to the same threshold (1000 counts) and the signals are expressed in BLI counts.

**Supplementary Figure 6**

A

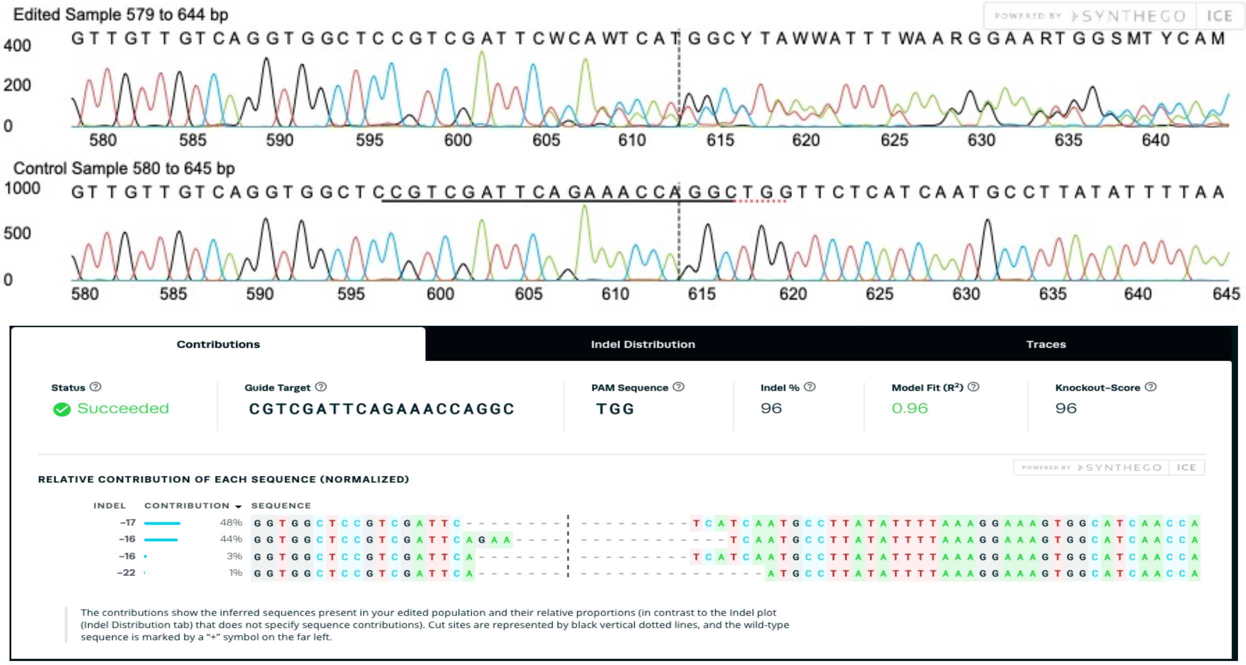


B

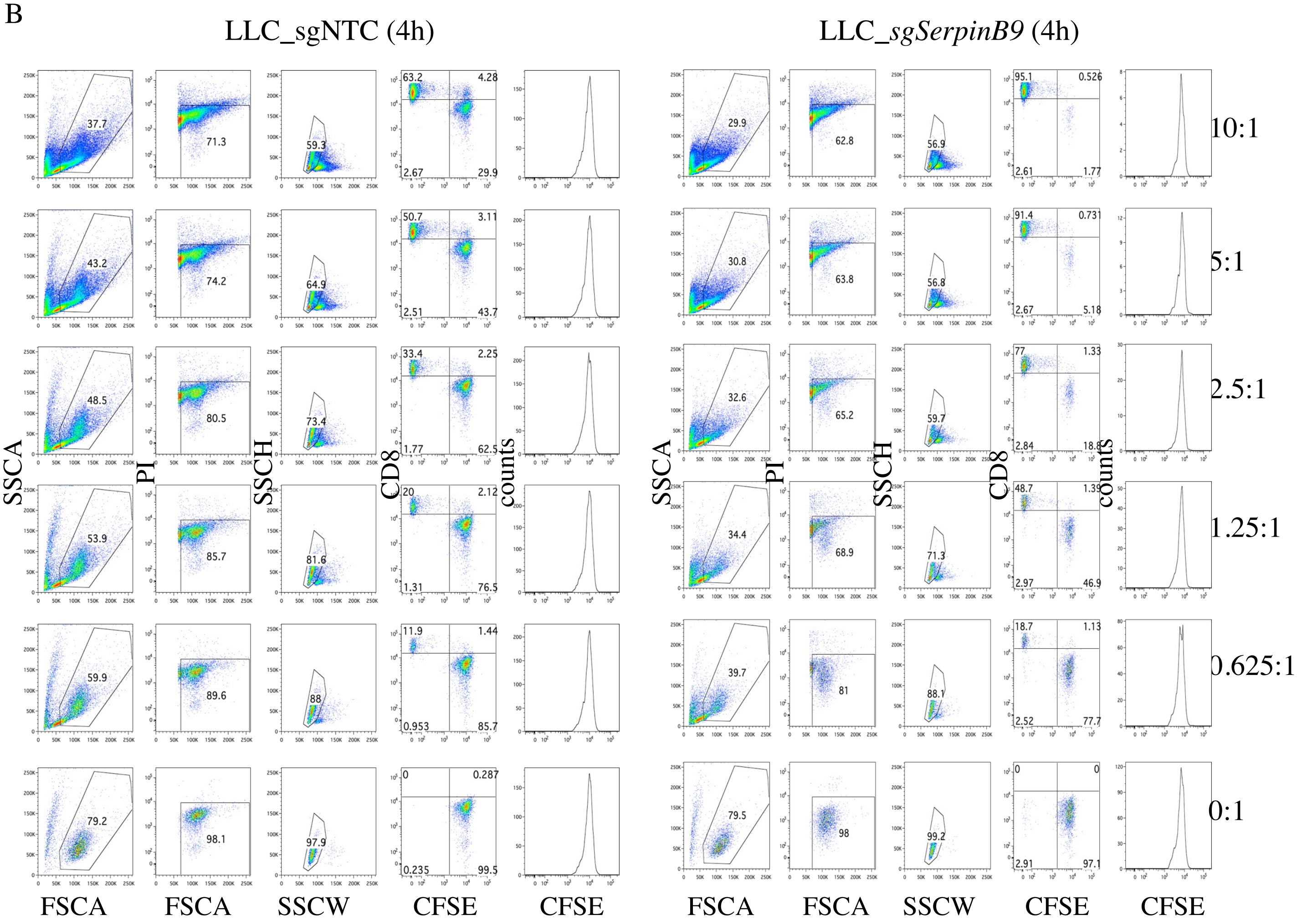


**Supplementary Figure 7**

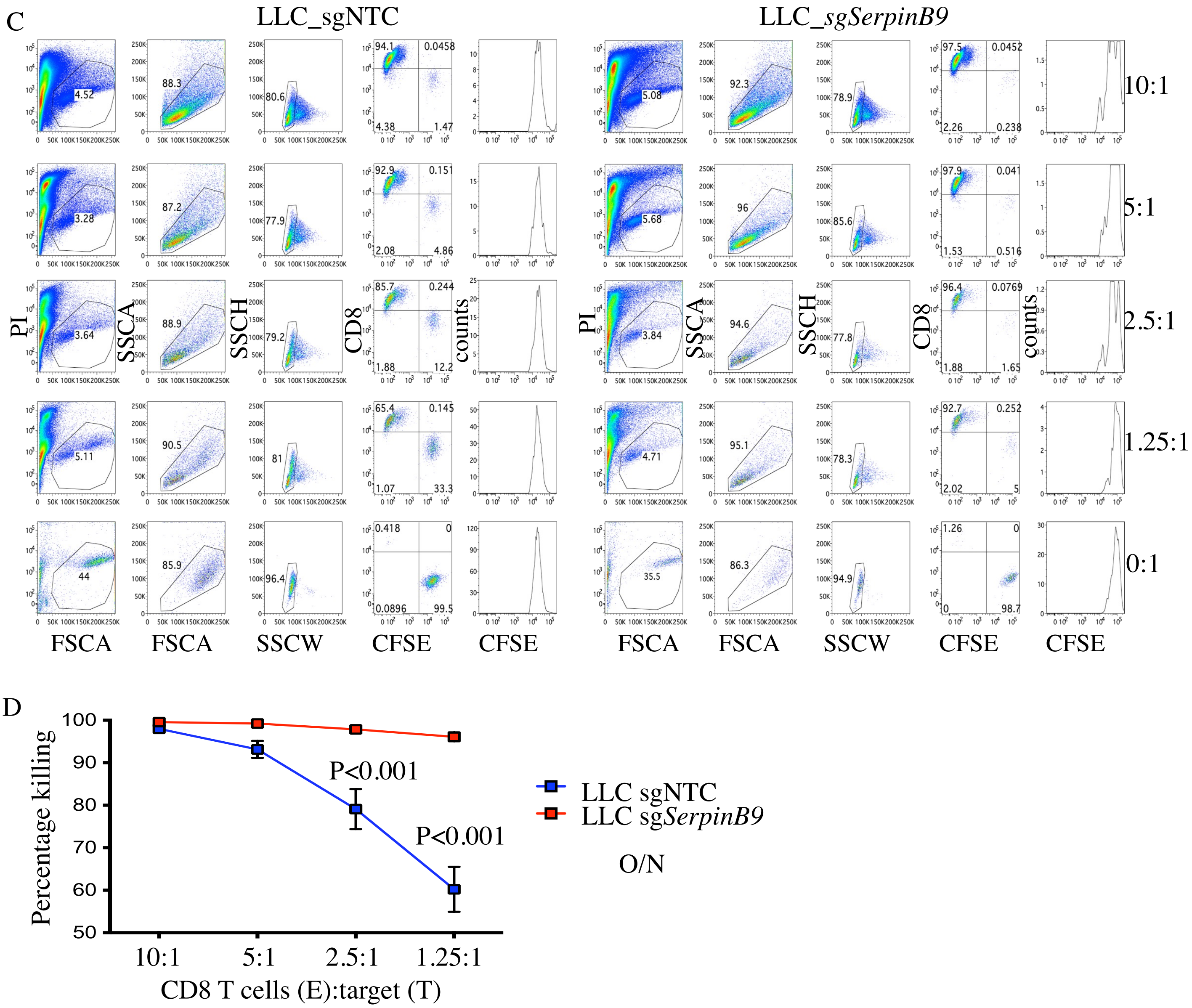


**Supplementary Figure 7: Genetic depletion of *Serpinb9* enhances T cell-mediated killing *in vitro***

**A,** Gene editing efficiency of *Serpinb9* in clonal murine Lewis Lung Carcinoma (LLC) cells was determined using Sanger sequencing of PCR-amplified sgRNA target sites followed by Inference of CRISPR Edits (ICE) analysis. **B,** Representative flow cytometry-based killing assay (4h) of CFSE labeled LLC cells transduced with sgNTC or sg*Serpinb9* (target) by *in vitro* activated OT-I effector cells (E) at different ratios. **C,** Representative flow cytometry-based killing assay (overnight) of CFSE labeled LLC cells transduced with sgNTC or sg*Serpinb9* (target) by *in vitro* activated OT-I effector cells (E) at different ratios. **D,** sgNTC control and sg*Serpinb9* LLC cells were labeled with CFSE and used as targets (T) and cocultured with activated OT-I effector cells (E) at different E:T ratio overnight. Graphs show percentage of T cell mediated killing at different E:T transduced with sgNTC (blue) or sg*Serpinb9* (red). Flow cytometry analysis was used to calculate the ratio of $PI^-$ $CFSE^+$ depleted cells cultured with effector cells and divided with $PI^-$ $CFSE^+$ depleted cells without effector cells at different time points. Numbers represent fold change of the mean values obtained from triplicate cocultures. Two independent experiments were performed for each time point. Significance in percent changes in the tumor volume between different groups were calculated by two-tailed unpaired t test with Welch's correction. Data presents mean ± s.e.m. for each group examined.

**Supplementary Figure 7 continued**

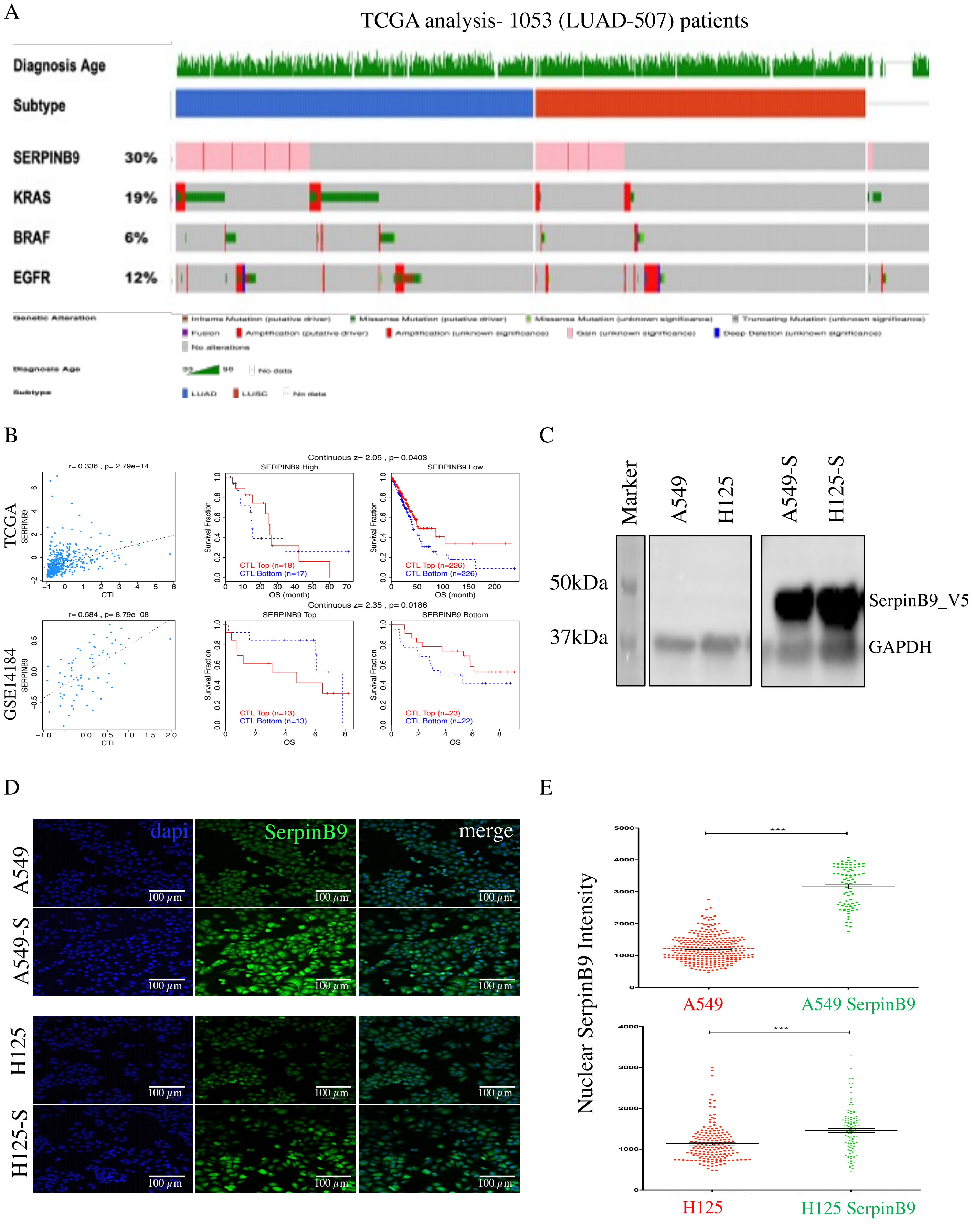


**Supplementary Figure 8**

F

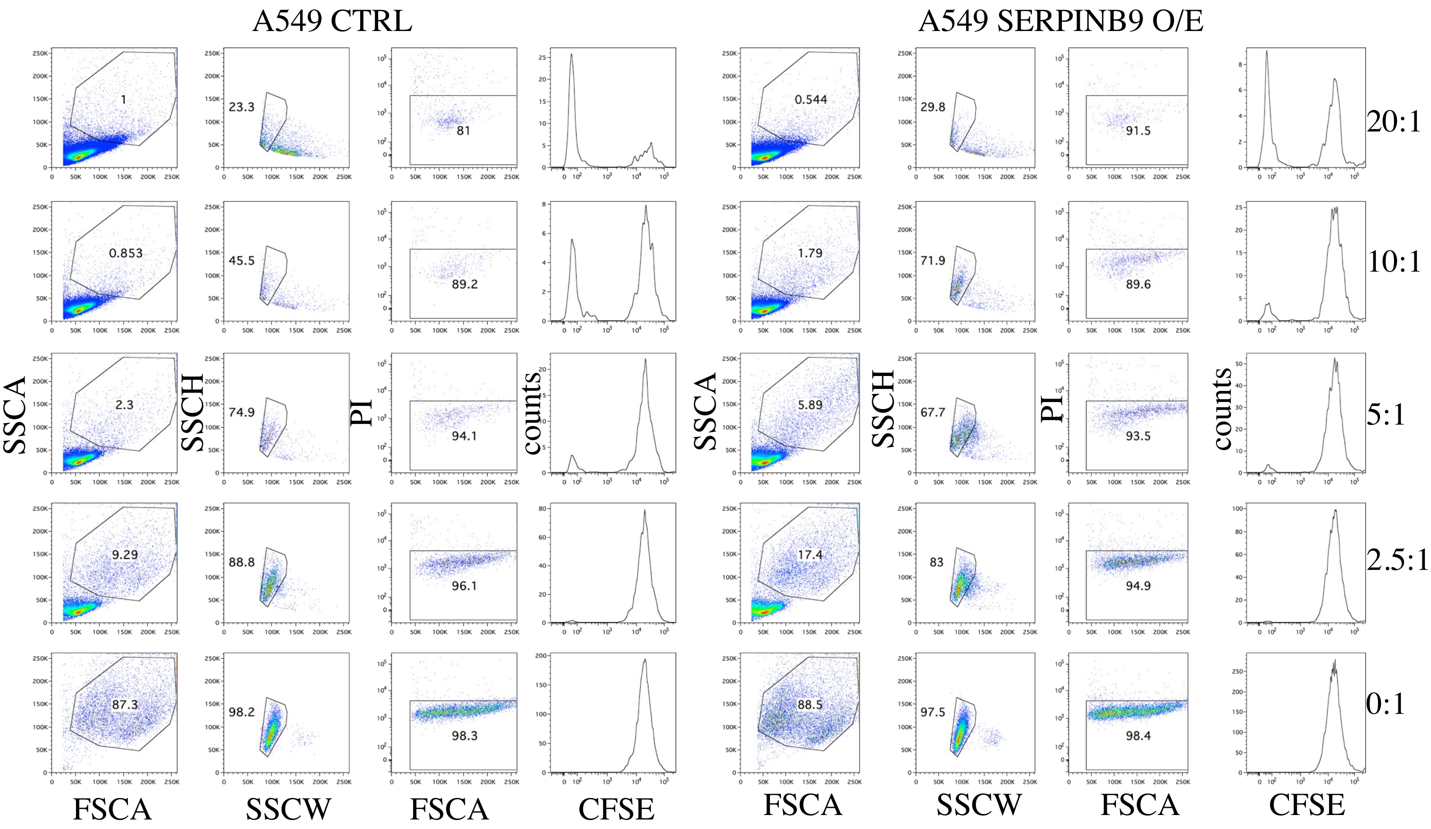


G

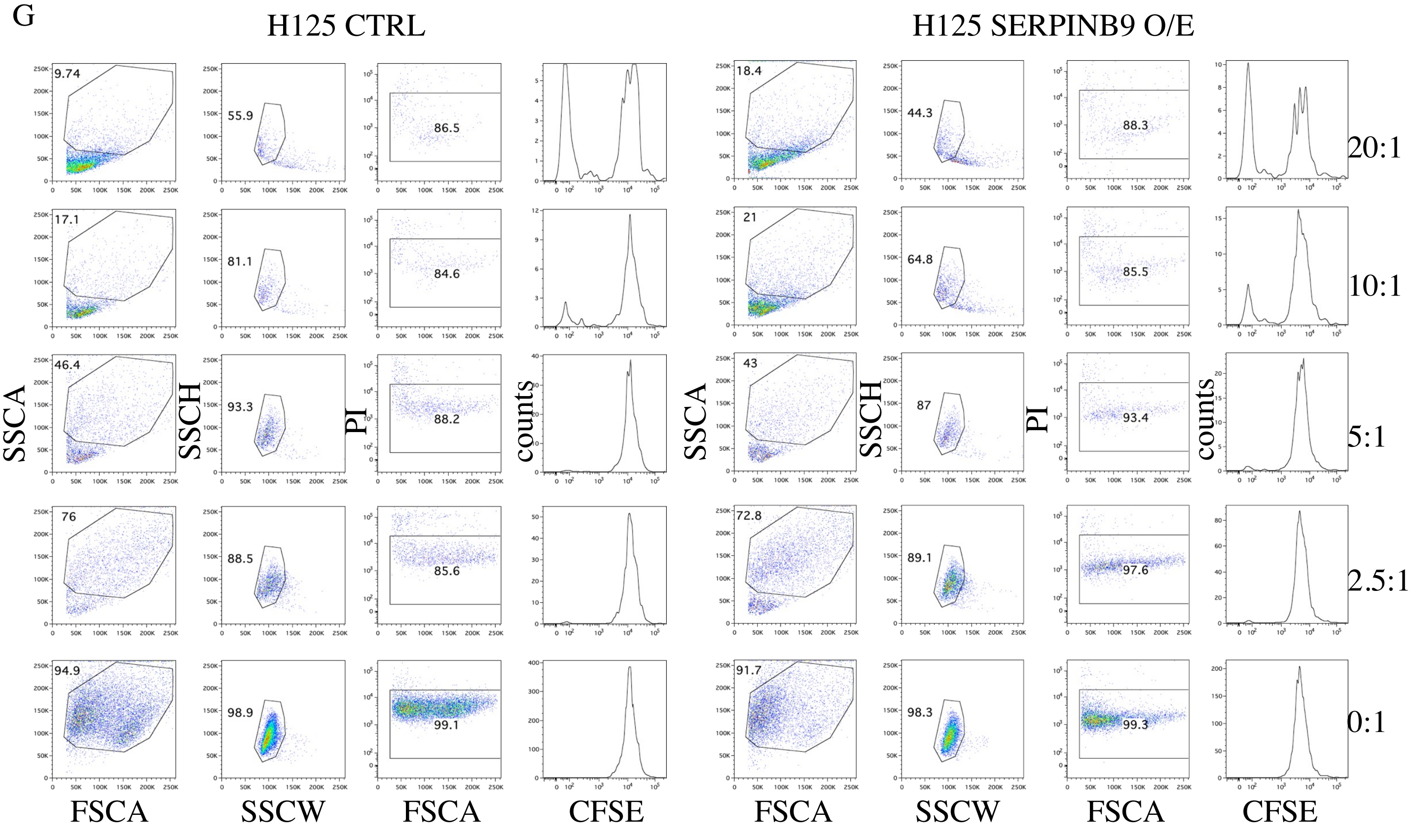


**Supplementary Figure 8 continued**

**Supplementary Figure 8: Expression, CNA and SNV of *SERPINB9* in human LUAD**

**A,** TCGA analysis depicted as a cBioPortal OncoPrint reveals that 38% of LUAD (*n*=191/507) and 27% of LUSC (*n*=127/499) exhibit gain or amplification of S*ERPINB*9, together accounting for 30% of these two lung cancer subtypes. **B,** Tumor Immune Disfunction and Exclusion (TIDE) analysis from TCGA (*n*=487) and GSE14184 (*n*=71) LUAD cohorts illustrates that low expression of SERPINB9 significantly correlates with higher level of CTLs in tumors and overall survival. **C,** Western blot analysis blotted for V5-tag showing overexpression of SERPINB9 (43kDa) and GAPDH (37kDa) in A549 and H125 human cancer cell lines. Data are representative of at least three independent experiments. **D,** Representative images of SERPINB9 staining in human lung cancer cell lines A549 and H125 without or with overexpression of SERPINB9. **E,** Quantification of SERPINB9 staining in A549 and H125 cancer cell lines without or with overexpression of SERPINB9 (n=4 biological samples from 3 independent experiments, for each cell line). Data were analysed by two-sided student's t-test and present mean ± s.e.m.(*** represents p<0.0001) F. Representative flow cytometry-based killing assay (overnight) of CFSE labeled A549 cells transduced with CTRL or SERPINB9 (target) by *in vitro* activated DNT (γδ) effector cells (E) at different ratios. G. Representative flow cytometry-based killing assay (overnight) of CFSE labeled H125 cells transduced with CTRL or SERPINB9 (target) by *in vitro* activated DNT (γδ) effector cells (E) at different ratios. Data presents mean ± s.e.m. of three replicates examined over two independent experiments by two-sided student's t-test.

A

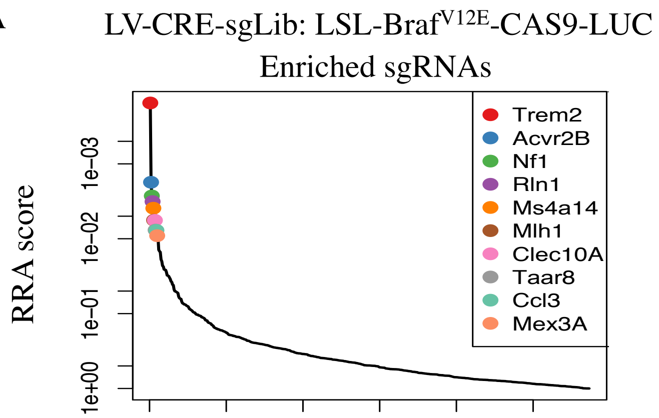


B

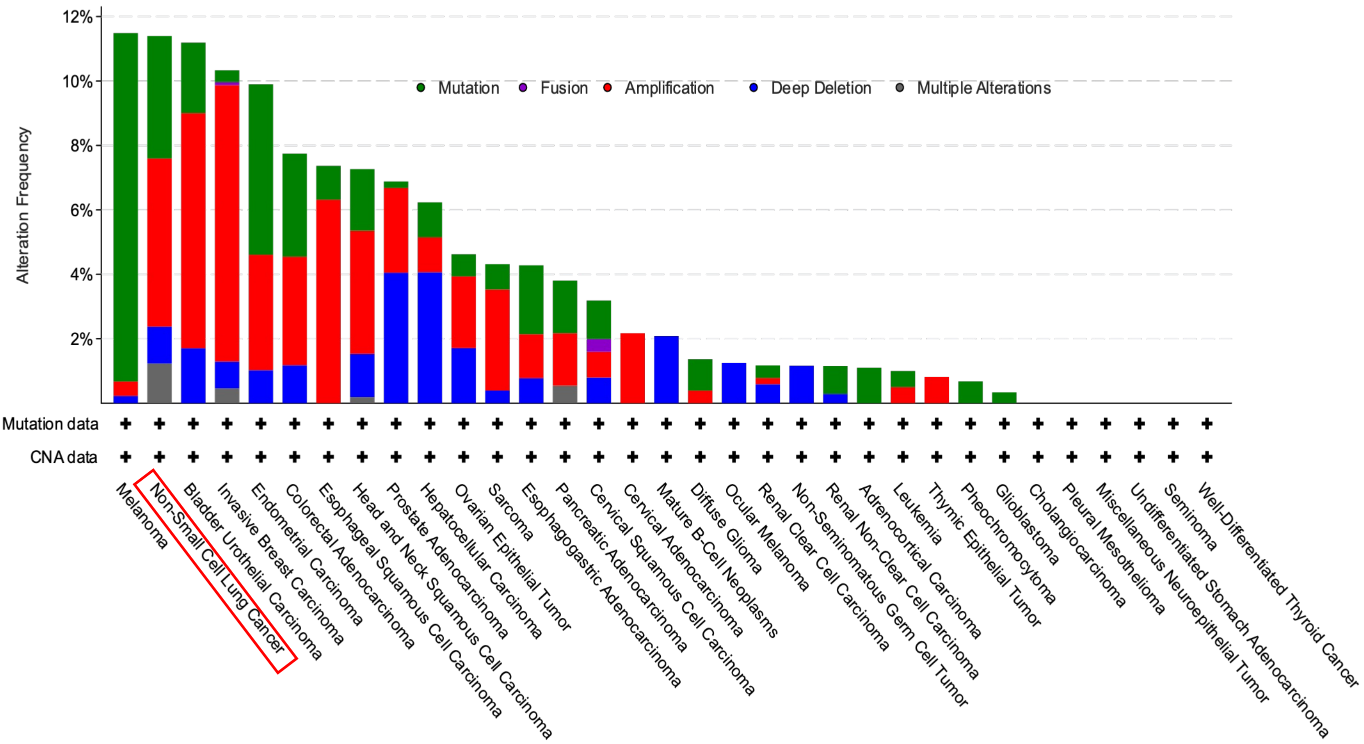


**Supplementary Figure 9: Pan-cancer expression of ADAM2**

**A,** Top 10 genes whose sgRNAs are enriched in lungs of ACT-treated *Braf* $^{V600E}$;Cas9 mice. (untreated, *n*=10; treated, *n*=10; RRA, Robust Rank Aggregation, which identifies statistically relevant genes). Data are representative of two independent experiments **B,** Bar grah shows alteration (mutation, fusion, amplification, deep deletion, and multiple alterations) frequencies of *ADAM2* across 33 tumor types obtained from TCGA dataset. *ADAM2* is amplified in ~5% of NSCLC cases analyzed.

**Supplementary Figure 9**

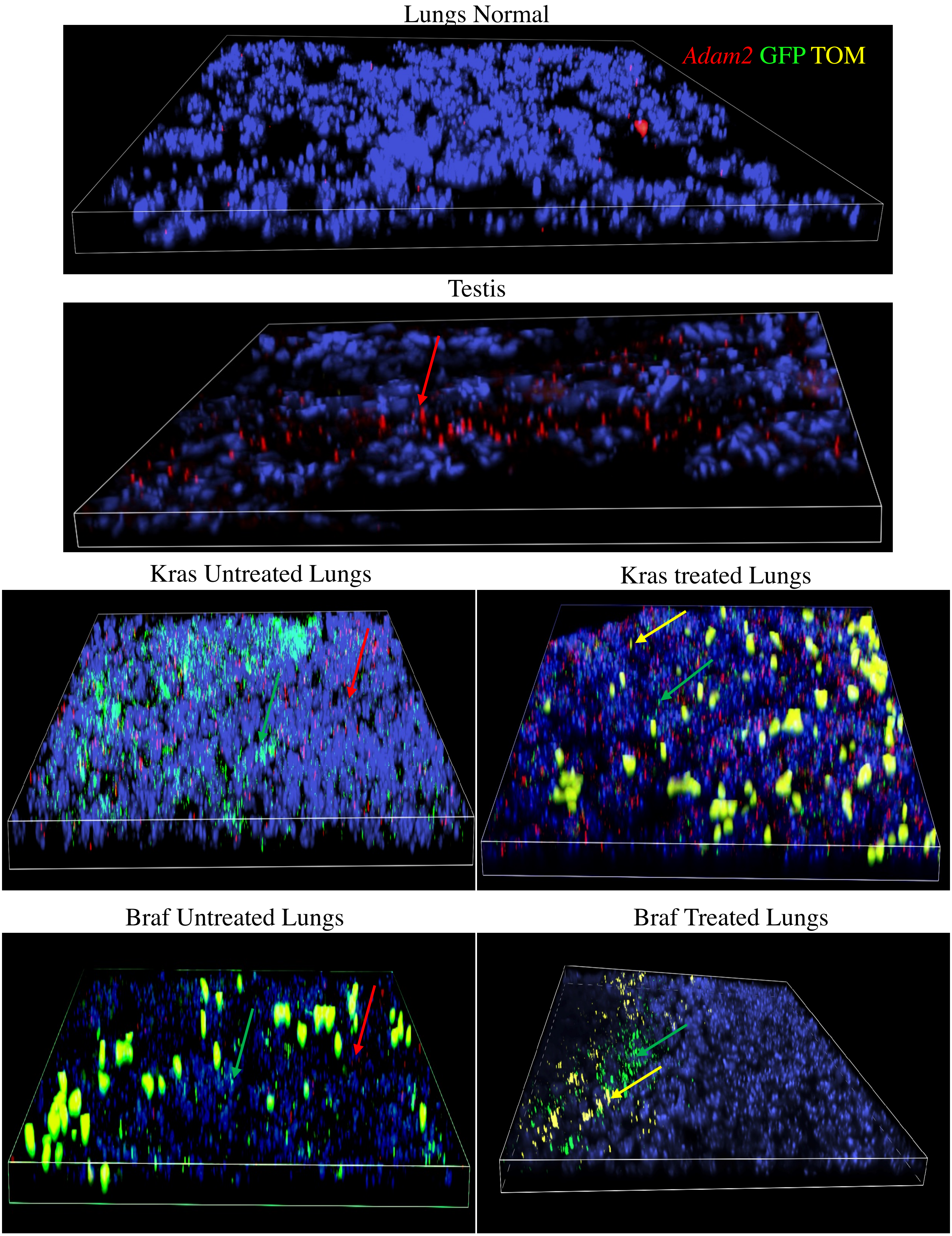


**Supplementary Figure 10**

**Supplementary Figure 10: KRas$^{G12D}$ induces expression of ADAM2 in lung tumors**

Representative 3D images from normal lungs (negative control), testis (positive control), and untreated or treated with ACT of OT-I cells *Kras*$^{G12D}$- and *Braf*$^{V600E}$-driven lung tumors probed for *Adam2* (red), GFP (green), and TOM (OT-I cells, yellow) using RNAscope. (n=5 normal lungs; n=3 testis; n=6 *Kras*$^{G12D}$Cas9; n=3 *Kras*$^{G12D}$Cas9+OT-I; n=4 *Braf*$^{V600E}$Cas9; n=3 *Braf*$^{V600E}$Cas9+OT-I cells; biologically independent samples). Images were captured with Nikon eclipse Ti2 inverted fluorescent microscope.

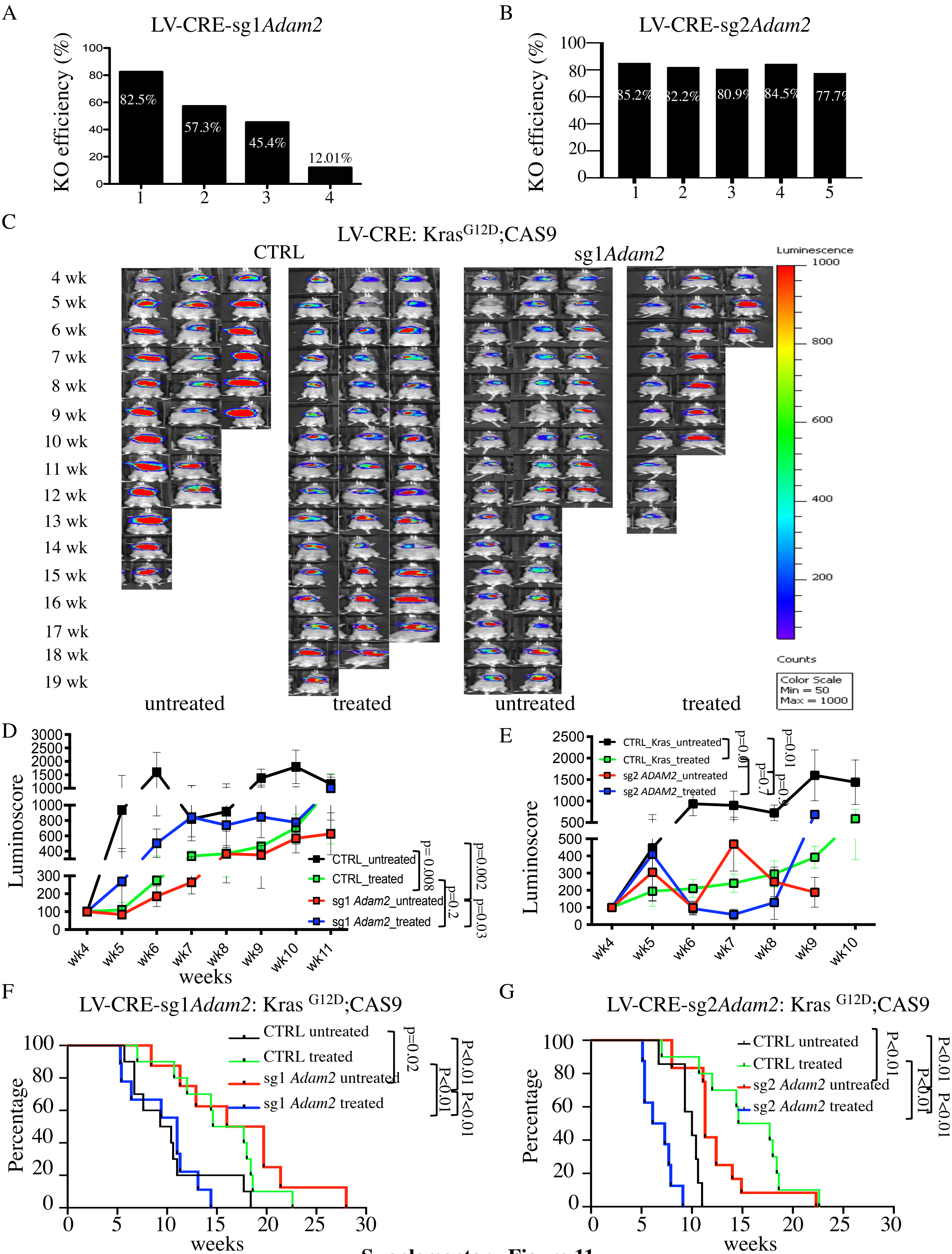


**Supplementary Figure 11**

**Supplementary Figure 11: Loss of ADAM2 inhibits T cell-mediated immune responses in $KRas^{G12D}$-driven lung tumors**

**A** and **B,** Gene editing efficiency of *Adam2* was determined using Sanger sequencing data of PCR-amplified sgRNA target sites followed by Tracking of Indels by Decomposition (TIDE) algorithm on bulk or GFP sorted tumor cells isolated from four and five different lungs of *Kras*$^{G12D}$;CAS9 mice transduced with sg1*Adam2* (A) or sg2*Adam2* (B), respectively. **C,** Representative images of untreated or ACT-treated *Kras*$^{G12D}$;CAS9 mice inhaled at P2 with sgNTC (untreated, n=10; treated, *n*=10) or sg*Adam2* untreated, *n*=20; treated, n=18; sg1 or sg2) measured by BLI. The pseudocolor images (rainbow color scale) were adjusted to the same threshold (1000 counts) and the signals are expressed in BLI counts. **(D** and **E)** Graphs show luminoscore in radiance (total flux p/s) and expressed as fold induction, from untreated (n=20) or ACT-treated *Kras*$^{G12D}$ (n=18);CAS9 mice transduced with sg1*Adam2* (D) or sg2*Adam2* (E). The p values for tumor growth were determined by an unpaired two-sided t-test with Welch's correction. Data are mean **±** s.e.m. **F,** Tumor free survival of *Kras*$^{G12D}$;CAS9 mice transduced with sgNTC (CTRL untreated n=10 and treated *n*=10) vs. sg1*Adam2* (untreated *n*=8 and treated n=10). **G,** Tumor free survival of *Kras*$^{G12D}$;CAS9 mice transduced with sgNTC (CTRL untreated n=7 and treated *n*=10) vs. sg2*Adam2* (untreated *n*=12 and treated n=8). Comparison of survival curves was performed by Log-rank (Mantel-Cox test).

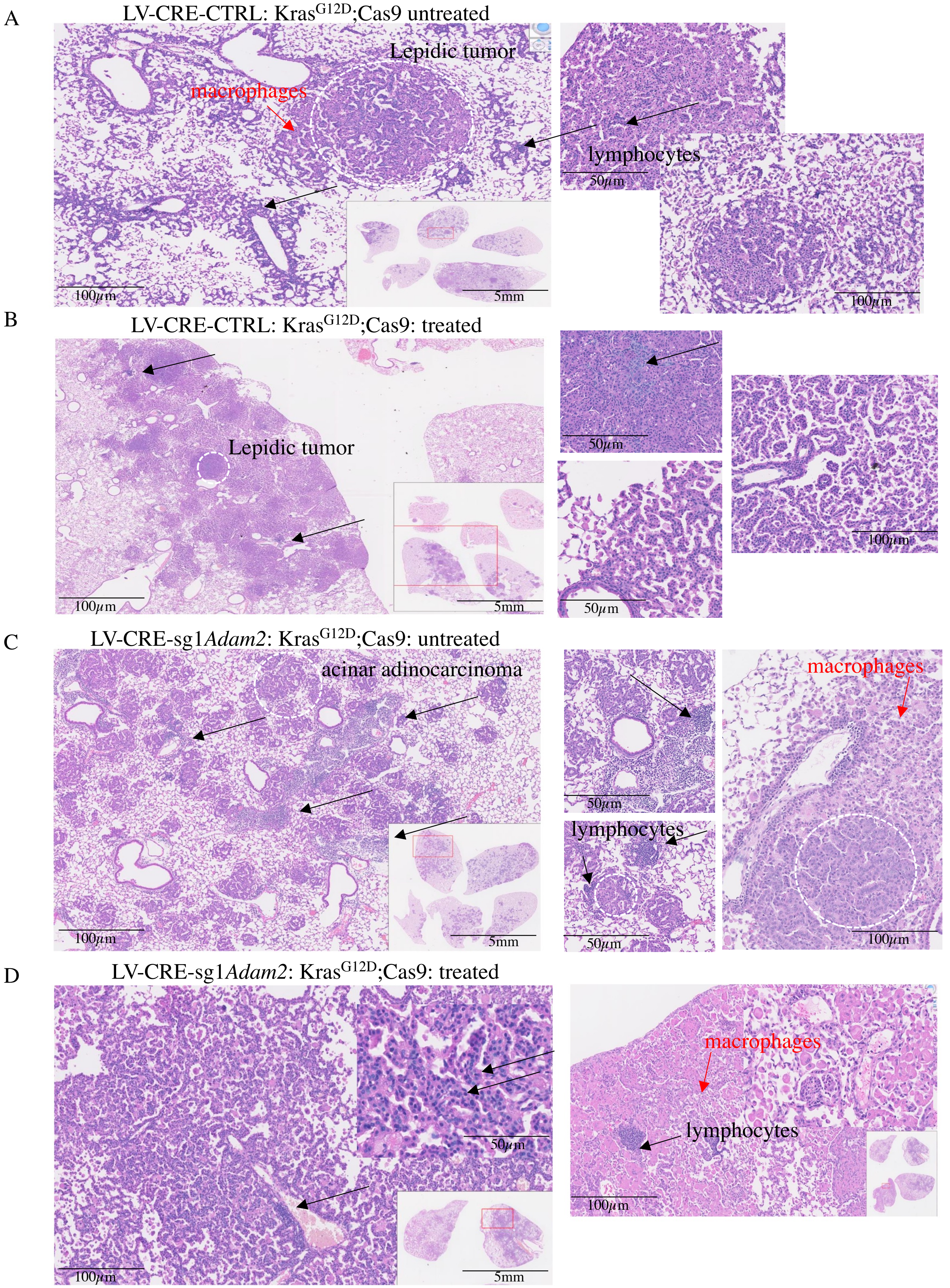


**Supplementary Figure 12**

**Supplementary Figure 12: ADAM2 controls tumor progression and immune cell infiltrates**

**A-D,** Representative H&E staining of lung sections from untreated or ACT-treated $Kras^{G12D}$;CAS9 lungs transduced with sgNTC (A and B); untreated n=4, treated n=4, biologically independent samples or sg*Adam2* (C and D) untreated n=3 and treated n=4, biologically independent samples. **A,** Image shows a multifocal to confluent lepidic, solid and micropapillary structures, fewer lymphatic infiltrates, some subpleural almost changes, intraalveolar macrophages/single and multinucleated cells. **B,** Large confluent tumor mass with some dense nodules. In the periphery lepidic, centrally micropapillary, nodules solid, little lymphoplasmacytic infiltration near artery and bronchus. **C,** Tumor with a pattern akin to acinar adenocarcinoma (nodules with holes), denser lymphoplasmacytic infiltrates at tumor's borders and around vessels and bronchiole. **D,** Adenocarcinoma with mostly lepidic pattern, with macrophages within alveolar spaces. Mostly normal alveoli with immune large infiltrates of single and multinucleated cells. Black arrows point to lymphocytes and red arrows point to macrophages.

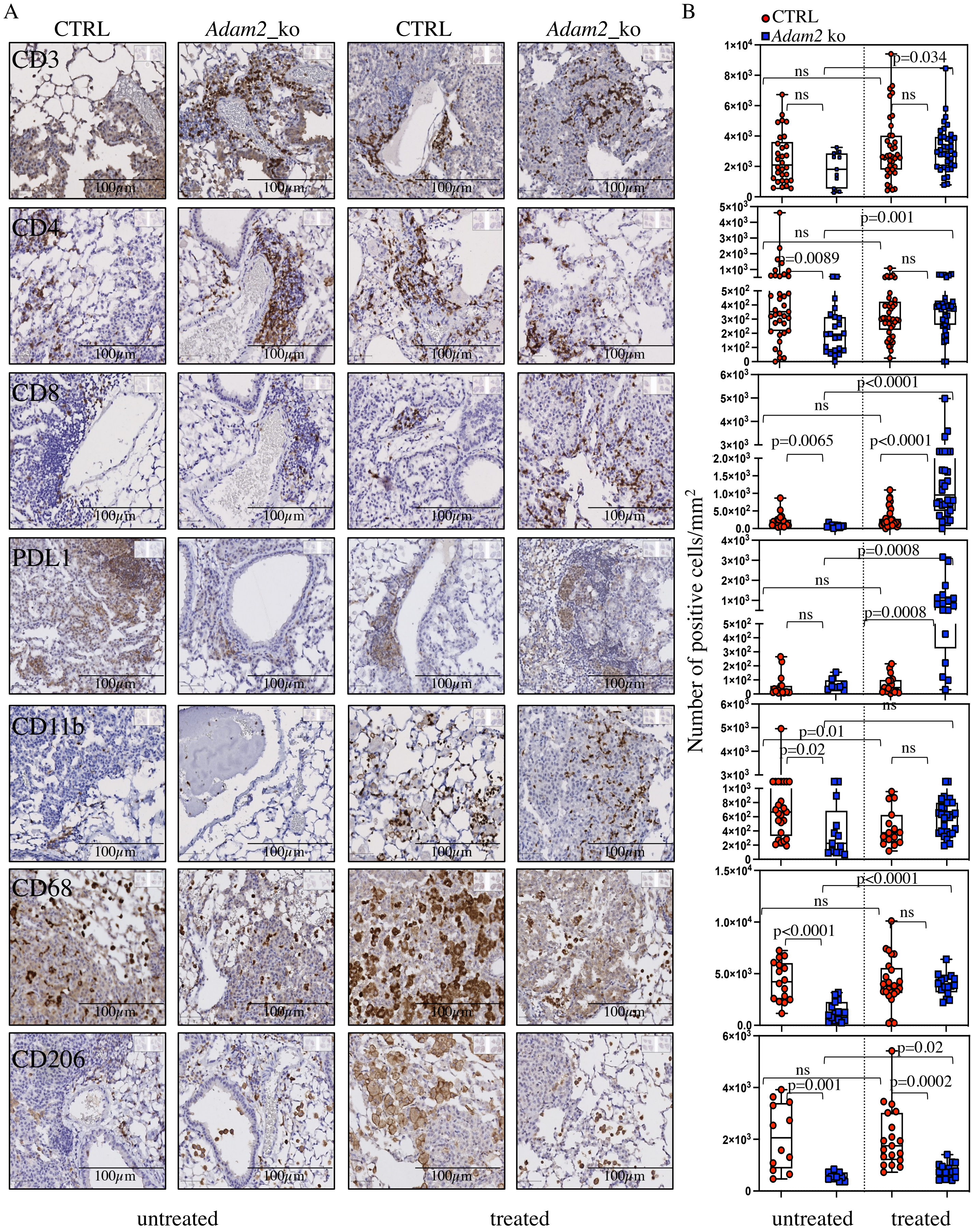


**Supplementary Figure 13**

**Supplementary Figure 13: *Adam2*-deficient ACT-treated lungs exhibit increased CD8 T cell infiltrates and PD-L1 expression**

**A,** Representative IHC images (20x) showing CD3, CD4, CD8, PDL1, CD11b, CD68 and CD206 staining of untreated or treated $Kras^{G12D}$;CAS9 lungs transduced with sgNTC and sgAdam2. The scale bars represent 100$\mu$m. **B,** Quantification of immune markers described in (A) using QuPath software. Significant increase in $CD8^{+}$ and $PDL1^{+}$ cells as well as decreased numbers of $CD68^{+}$ $CD206^{+}$ immunosuppressive M2 macrophages were observed in *Adam2* knock-out lungs compared to CTRL lungs. Data analysis was performed on biologically independent untreated or treated $Kras^{G12D}$;CAS9 lungs transduced with sgNTC and sgAdam2. Every dot represents and independent ROI. Quantification data are shown as mean ±SEM analyzed by two-sided unpaired t-test with Welch's correction. Box and whiskers plots illustrate the median, first and third quartiles, maximum and minimum of relative abundance for markers tested between two groups analyzed by two-sided student's t-test.

A

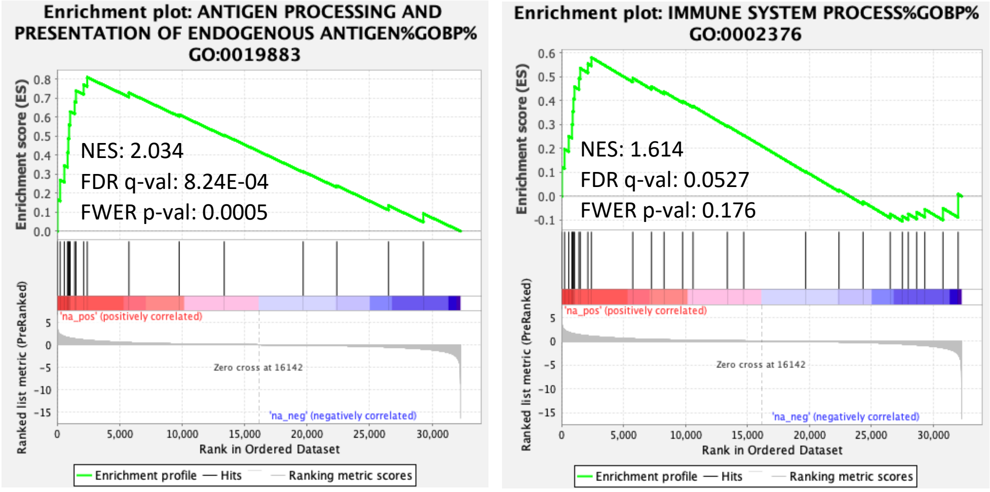


B

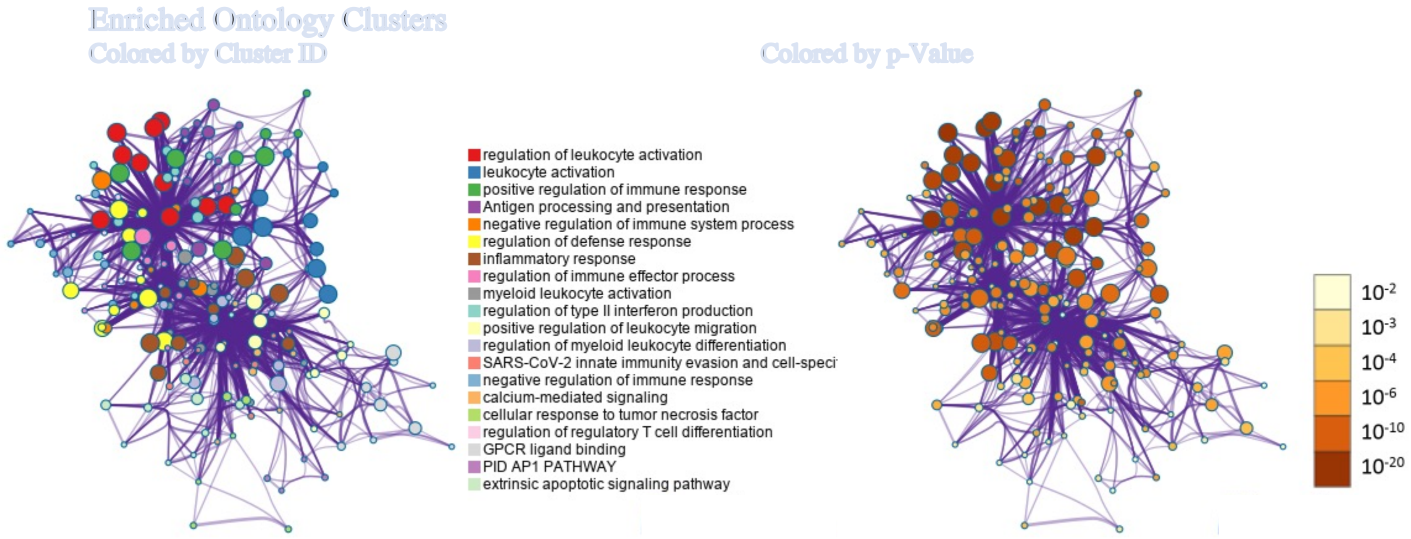


C

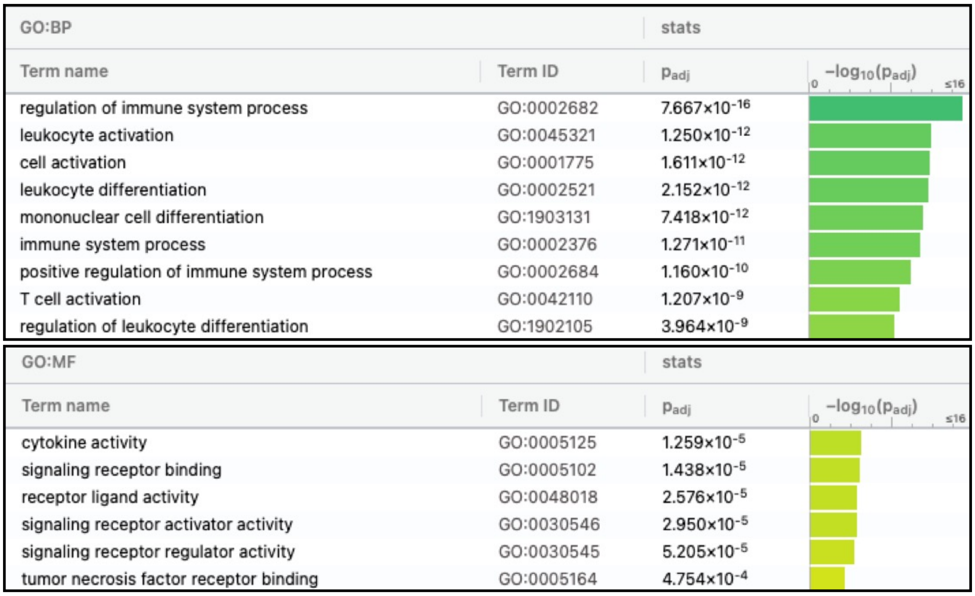

| GO:BP Term name | Term ID | $p_{adj}$ |
|---|---|---|
| regulation of immune system process | GO:0002682 | $7.667\times10^{-16}$ |
| leukocyte activation | GO:0045321 | $1.250\times10^{-12}$ |
| cell activation | GO:0001775 | $1.611\times10^{-12}$ |
| leukocyte differentiation | GO:0002521 | $2.152\times10^{-12}$ |
| mononuclear cell differentiation | GO:1903131 | $7.418\times10^{-12}$ |
| immune system process | GO:0002376 | $1.271\times10^{-11}$ |
| positive regulation of immune system process | GO:0002684 | $1.160\times10^{-10}$ |
| T cell activation | GO:0042110 | $1.207\times10^{-9}$ |
| regulation of leukocyte differentiation | GO:1902105 | $3.964\times10^{-9}$ |

| GO:MF Term name | Term ID | $p_{adj}$ |
|---|---|---|
| cytokine activity | GO:0005125 | $1.259\times10^{-5}$ |
| signaling receptor binding | GO:0005102 | $1.438\times10^{-5}$ |
| receptor ligand activity | GO:0048018 | $2.576\times10^{-5}$ |
| signaling receptor activator activity | GO:0030546 | $2.950\times10^{-5}$ |
| signaling receptor regulator activity | GO:0030545 | $5.205\times10^{-5}$ |
| tumor necrosis factor receptor binding | GO:0005164 | $4.754\times10^{-4}$ |

**Supplementary Figure 14**

**Supplementary Figure 14: GSEA, MetaScape and gProfiler analysis of *Adam2* knock-out and control lung tumors.**

**A,** Gene set enrichment analysis reveals upregulation of ANTIGEN PROCESSING AND PRESENTATION OF ENDOGENOUS ANTIGEN and IMMUNE SYSTEM PROCESS in ACT-treated *Adam2* knock-out tumors compared to control tumors isolated 6 weeks after tumor induction. **B**, MetaScape analysis showing enriched ontology clusters colored by cluster ID and by p-value in ACT-treated *Adam2* knock-out tumors compared to control tumors isolated 6 weeks after tumor induction from n=4 independent biological samples from both groups (Zhou *et al*. Nature Communications, 2019, 10[1]:1523)[1]. **C**, gProfiler analysis showing the top upregulated GO terms for biological processes (BP) and molecular function (MF) in ACT-treated *Adam2* knock-out tumors compared to control tumors isolated 6 weeks after tumor induction. The analysis was performed on sets of differentially expressed genes (DEGs) with Log2FC>2 using Benjamini-Hochberg FDR (FDR<0.05) between Adam2 ko compared to CTRL tumors isolated from C57BL/6 mice.

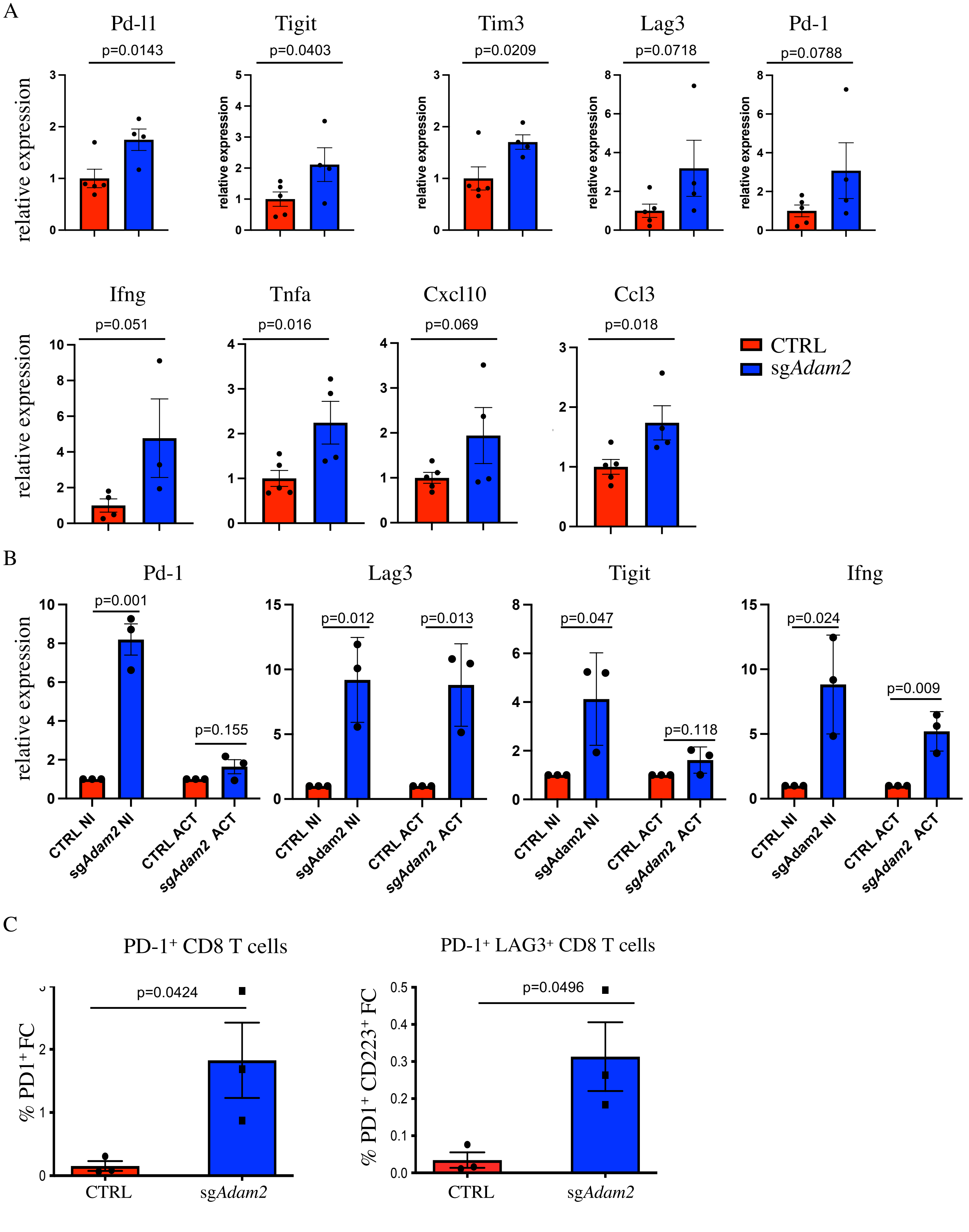


**Supplementary Figure 15**

**Supplementary Figure 15: *Adam2*-deficient lung tumors and infiltrating CD8 T cells exhibit increased expression of several cytokines and immune checkpoint receptors**

**A,** Quantitative RT-PCR analysis shows increased expression of indicated cytokines and immune checkpoint receptors in ACT-treated *Adam2* knock-out $Kras^{G12D}$;CAS9 lung tumors compared to control tumors (n=3 CTRL; n=6 sg1 or sg2 *Adam2* biologically independent samples). Changes in gene expression relative to control were normalized to *Ppib* housekeeping gene. Data are expressed using the ddCt analysis. Data presents mean ± s.e.m. comparison of experimental groups with control groups examined by student t-test. **B**, Quantitative RT-PCR analysis shows increased expression of indicated immune checkpoint receptors and *Ifng* in CD8 T-cells isolated from untreated and ACT-treated control and *Adam2* knock-out $Kras^{G12D}$;CAS9 lung tumors (untreated; n=3 CTRL; n=6 sg1 or sg2 *Adam2*; treated: n=5 CTRL and n=4 sg1 or sg2*Adam2* biologically independent samples). Changes in gene expression relative to control were normalized to *Ppib* housekeeping gene. Data are expressed using the ddCt analysis. Data presents mean ± s.e.m. comparison of experimental groups with control groups examined by student t-test. **C**, FACS analysis pre-gated on FSC/SSC, SSCW/SSCH, FSCA/$DAPI^{-}$ shows expression of PD-1 and LAG3 in CD8 T-cells isolated from untreated and ACT-treated control and Adam2 knock-out $Kras^{G12D}$;CAS9 lung tumors (n=3 biologically independent samples for each group examined over at least three independent experiments). Data presents mean ± s.e.m. analyzed by two-sided student's t-test.

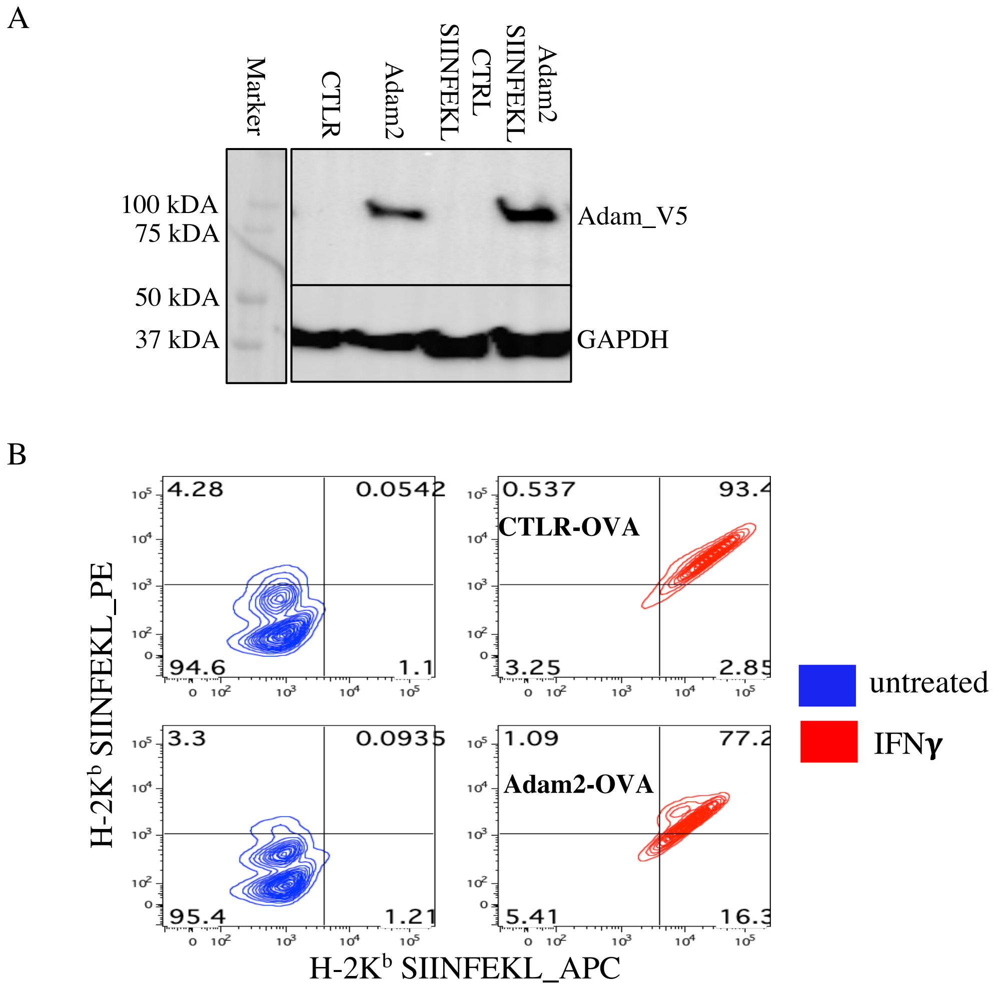


**Supplementary Figure 16: Ectopic expression of Adam2 in LLC cells**

**A,** Western blot analysis showing expression of Adam2-V5 in LLC cell line transduced with vector-only control (CTRL) lentivirus or Adam2 ORF overexpressing lentivirus, blotted for V5 tag and GAPDH. Data are representative of at least three independent experiments. **B,** Flow cytometry analysis of $H2K^b$ or $H2K^b$SIINFEKL in LLC cell lines without or with overexpression of ADAM2, pre-gated on FSC/SSC, SSCW/SSCH, FSCA/$DAPI^-$. Data are representative of at least three independent experiments.

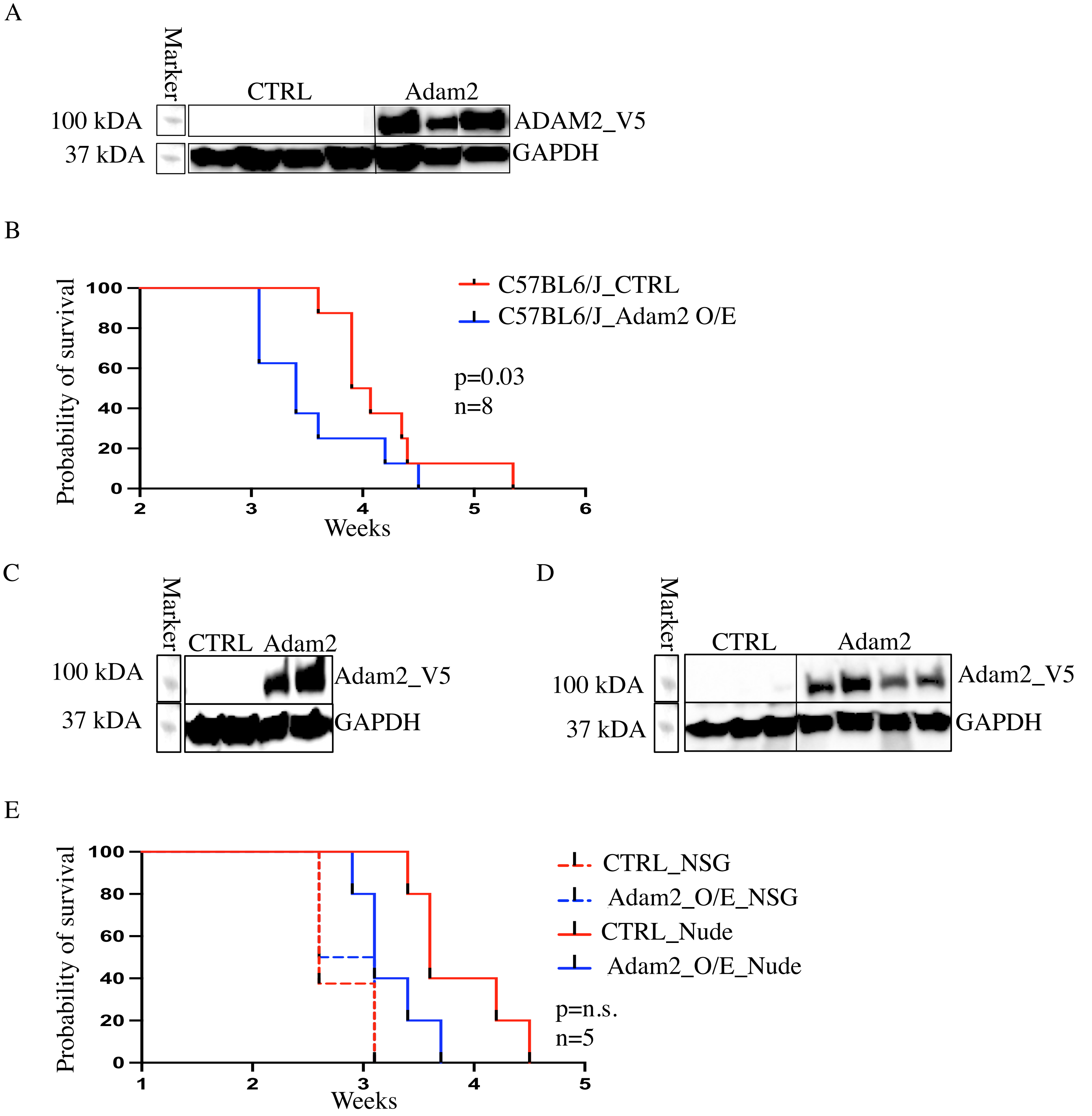


**Supplementary Figure 17: Adam2 promotes tumorigenesis in the absence of Ag-specific immunity**

**A,** Western blot analysis showing expression of Adam2-V5 blotted for V5 tag and GAPDH in tumors isolated from s.c. transplanted CTRL (n=4) or Adam2 O/E (n=3) cells into C57BL/6 mice. **B,** Kaplan-Meier survival analysis of the C57BL/6 mice bearing CTRL or Adam2 O/E tumors (n=8, biologically independent samples). Comparison of survival curves was performed by Log-rank (Mantel-Cox test). **C** and **D,** Western blot analysis showing expression of Adam2-V5 blotted for V5 tag and GAPDH in tumors isolated from NSG mice (D) and Nude mice (D). Data are representative of biologically independent n=2 CTRL and n=2 Adam2 O/E for NSG; n=3 CTRL and n=4 Adam2 O/E for Nude mice. **E,** Kaplan-Meier survival analysis of NSG and Nude mice bearing CTRL or Adam2 O/E tumors (n=5 for each group). Comparison of survival curves was performed by Log-rank (Mantel-Cox test).

**Supplementary Figure 17**

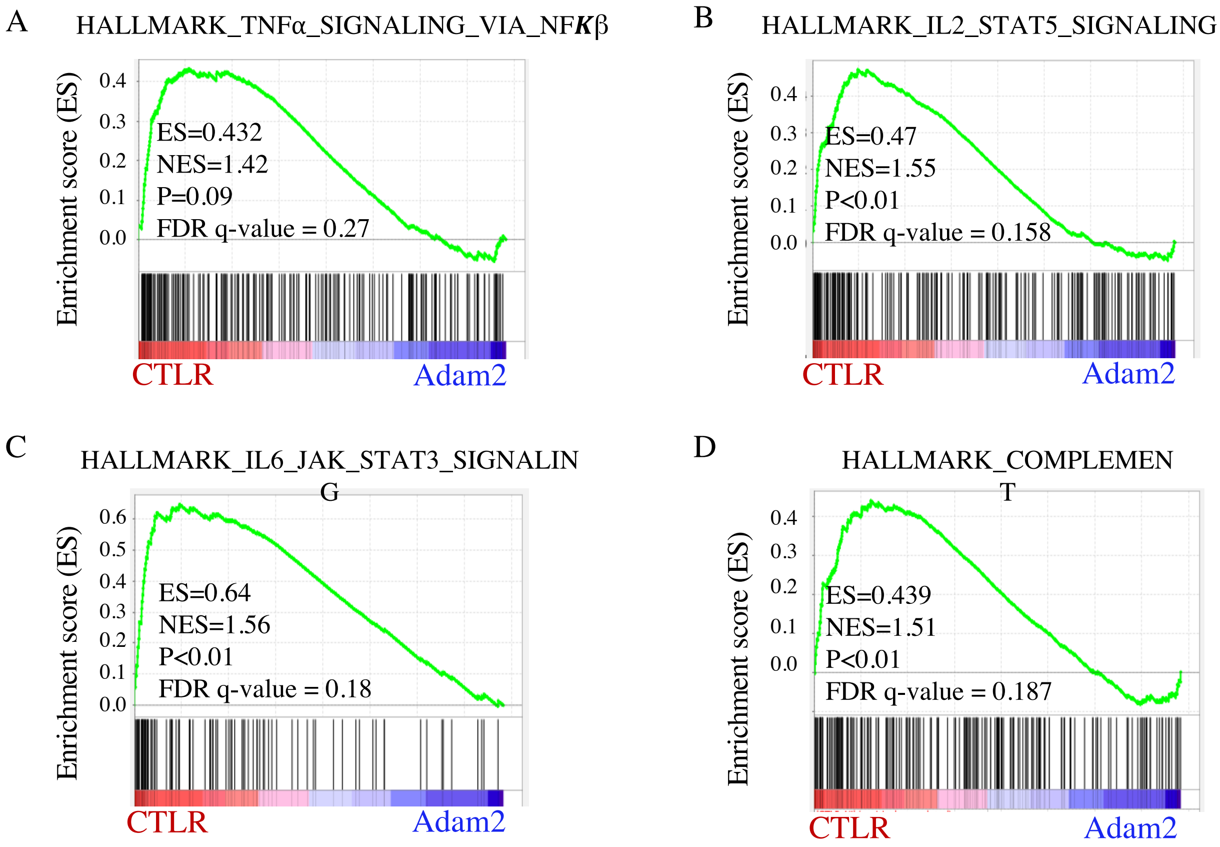


**Supplementary Figure 18: Adam2 downregulates multiple pathways associated with immune function**

**A-D**, Gene set enrichment analysis reveals downregulation of TNFa signaling via NFkb (A), IL2_STAT5_signaling (B), IL6_JAK_STAT3 signaling (C), and complement pathways (D) in tumors overexpressing Adam2 in comparison to CTRL tumors isolated from C57BL/6 mice on day 9 after tumor onset. RNAseq was performed on 3 biologically independent samples from each group.

**Supplementary Figure 18**

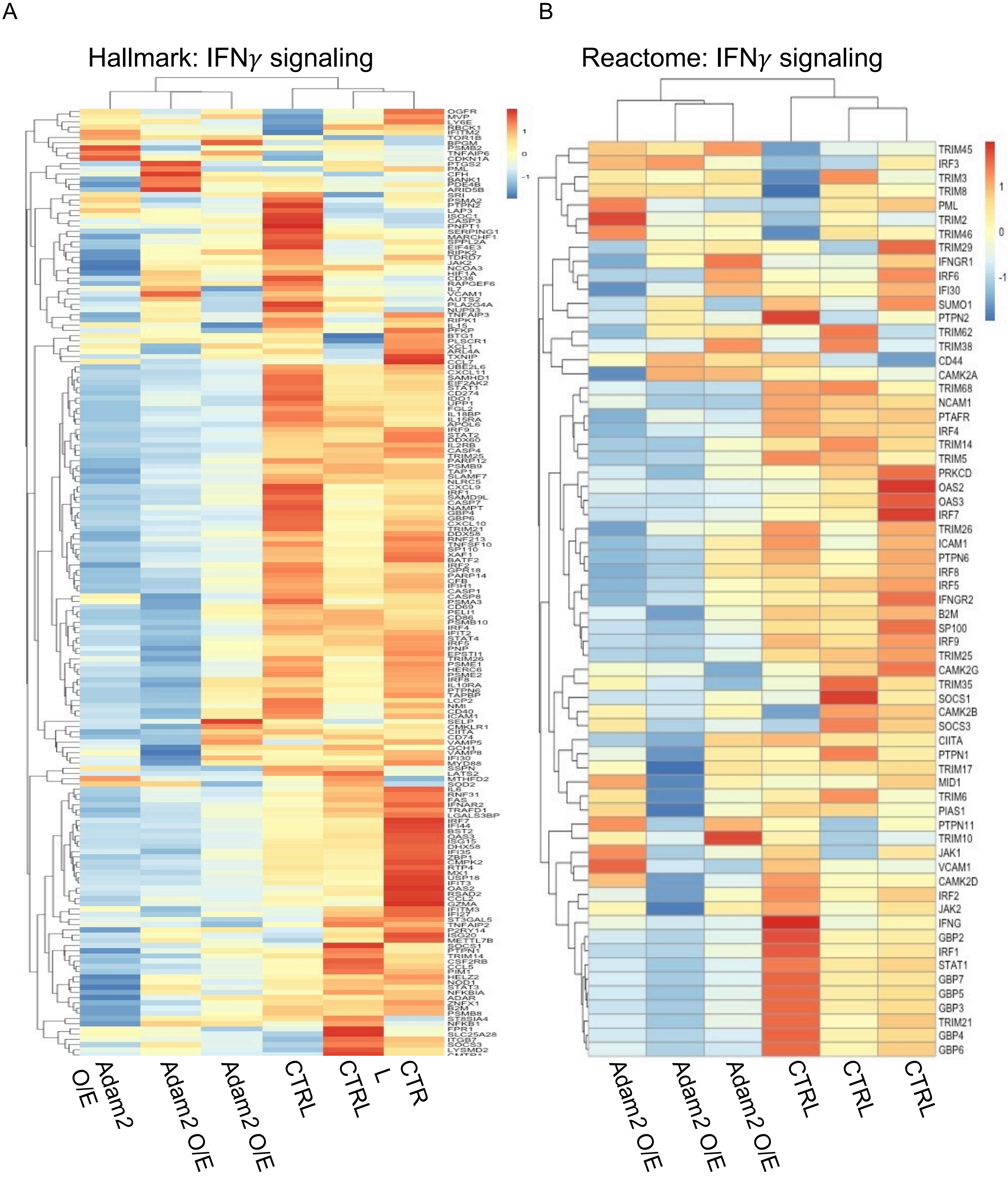


**Supplementary Figure 19: GSEA of Adam2 O/E tumors**

**A** and **B,** Heatmap of GSEA Hallmark (A) and Reactome (B) IFNγ signaling genes in ADAM2 O/E tumors versus CTRL tumors isolated from C57BL/6 mice on day 9 after tumor onset. RNAseq was performed on 3 biologically independent samples from each group.

**Supplementary Figure 19**

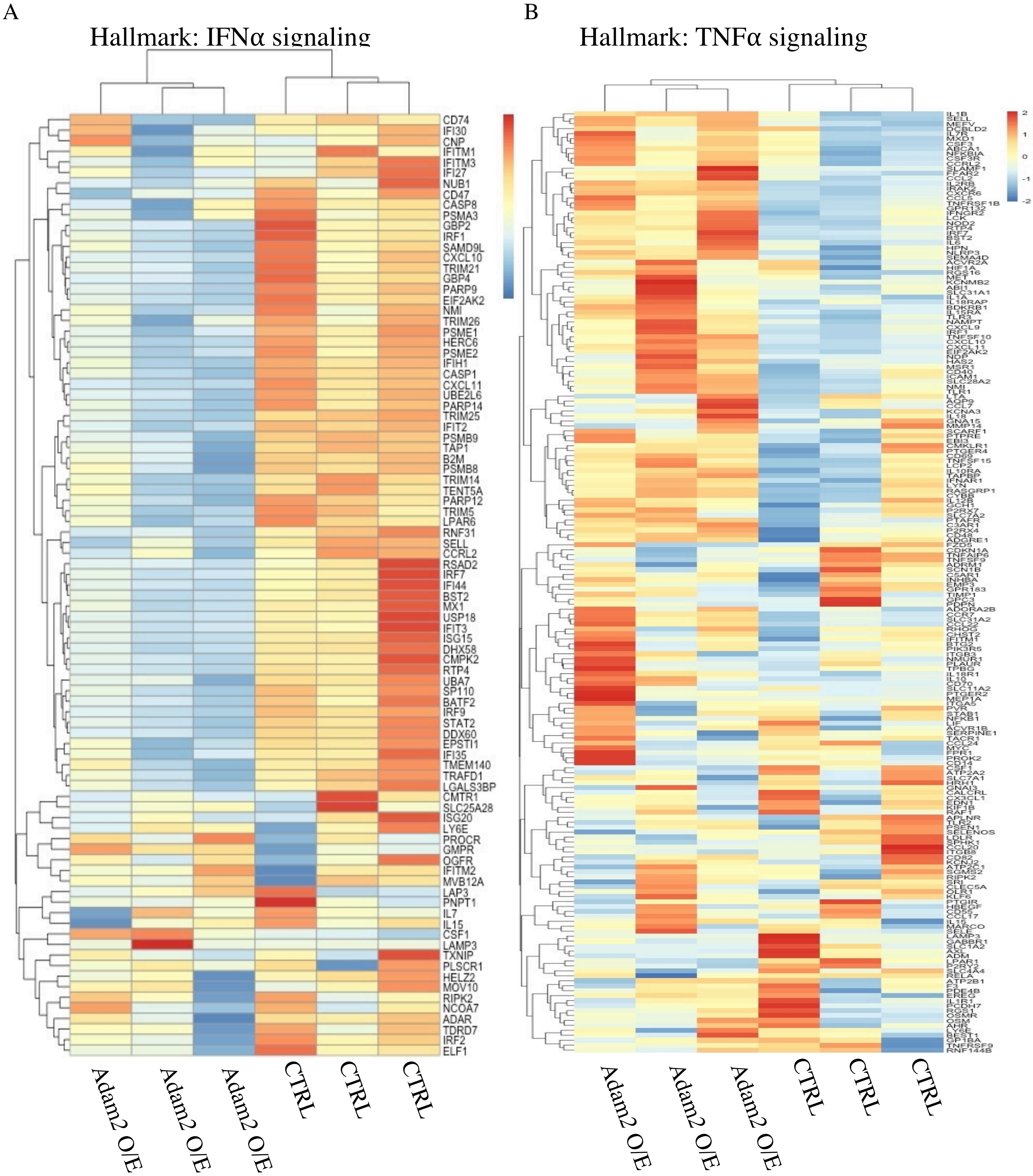


**Supplementary Figure 20: GSEA of Adam2 O/E tumors**

**A** and **B,** Heatmap of GSEA Hallmark IFNa signaling (A) and TNFa signaling (B) genes in Adam2 O/E tumors versus CTRL tumors isolated from C57BL/6 mice on day 9 after tumor onset. RNAseq was performed on 3 biologically independent samples from each group.

**Supplementary Figure 20**

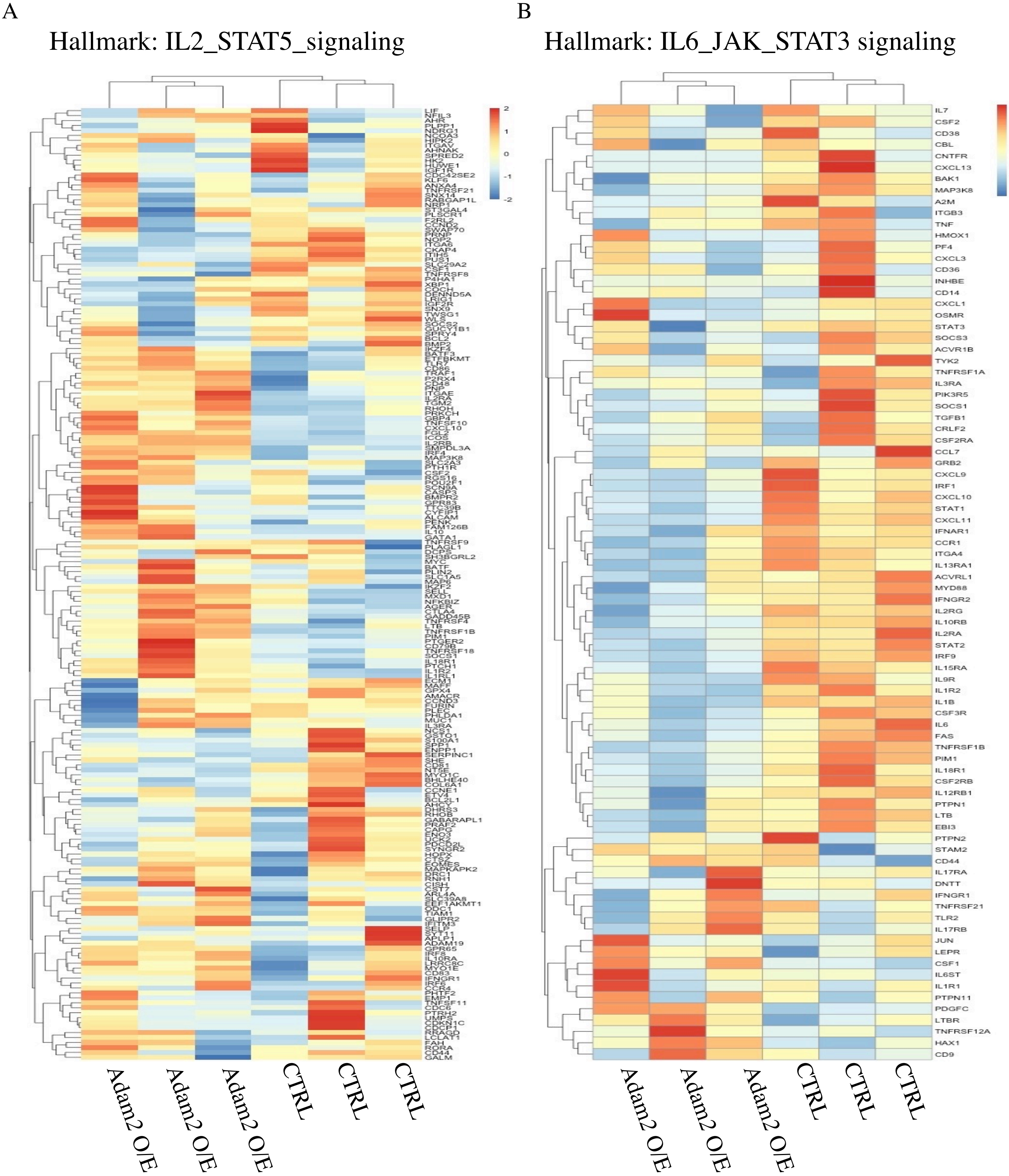


**Supplementary Figure 21: GSEA of Adam2 O/E tumors**

**A** and **B,** Heatmap of GSEA Hallmark IL2_STAT5 signaling (a) and Hallmark of IL6_JAK_STAT3 signaling (b) in Adam2 O/E tumors versus CTRL tumors isolated from C57BL/6 mice on day 9 after tumor onset. RNAseq was performed on 3 biologically independent samples from each group.

**Supplementary Figure 21**

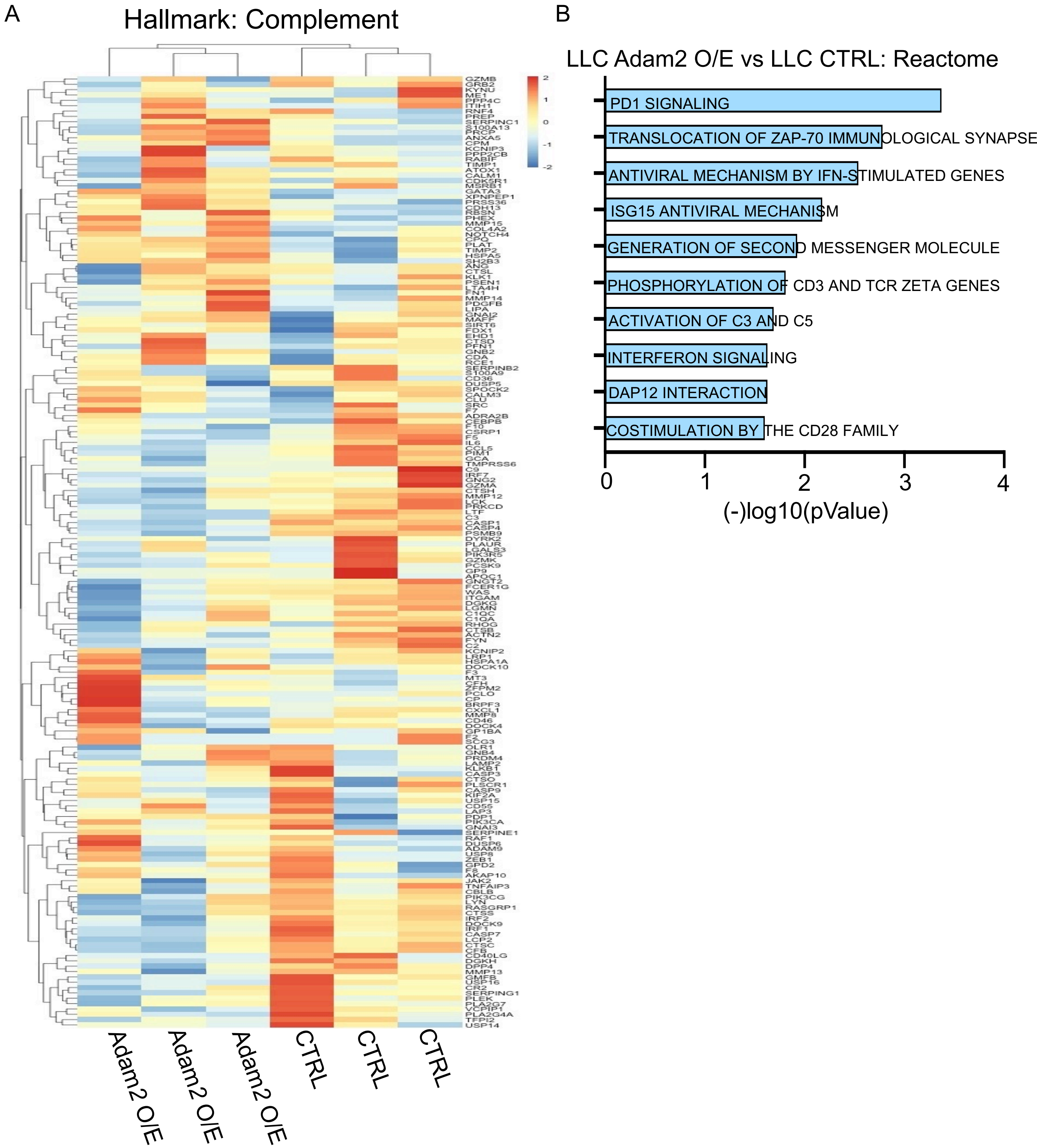


**Supplementary Figure 22: GSEA of Adam2 O/E tumors**

**A,** Heatmap of GSEA Hallmark Complement genes in Adam2 O/E tumors versus CTRL tumors. **B,** Bar graph showing Reactome GSEA terms of biological processes downregulated in Adam2 O/E compared to CTRL tumors isolated from C57BL/6 mice on day 9 after tumor onset. Bar graph showing Gene Ontology of the DEGs (FC>2, $p<0.05$) downregulated between Adam2 O/E versus CTRL tumors assigned to Biological Process. RNAseq was performed on 3 biologically independent samples from each group.

**Supplementary Figure 22**

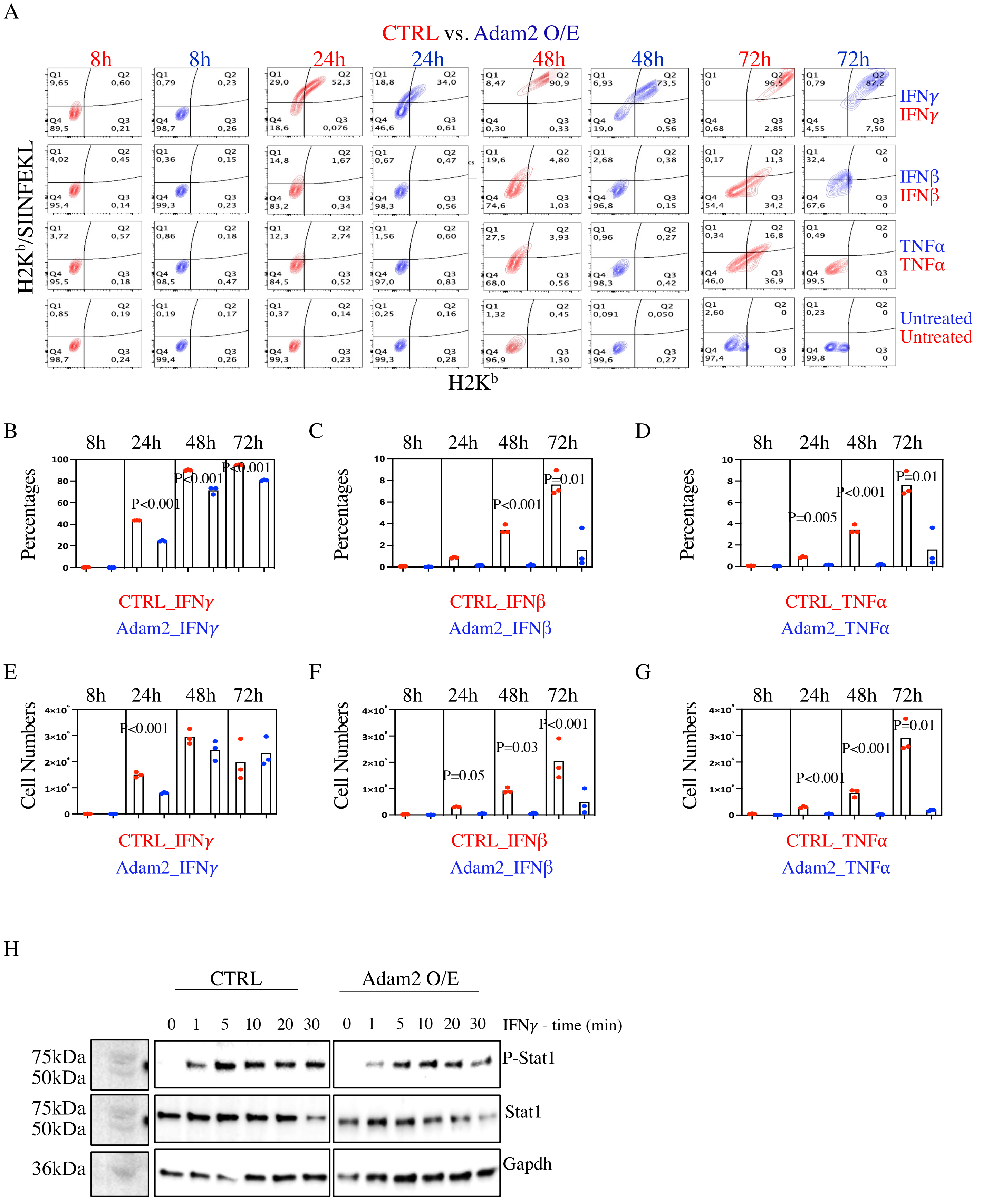


**Supplementary Figure 23**

**Supplementary Figure 23: Adam2 regulates IFN induced expression of MHC-class I and activation of Stat1.** **A,** Flow cytometry analysis of cell surface expression for H2K$^b$/ H2K$^b$SIINFEKL on CTRL versus ADAM2 O/E cells untreated or treated with IFNg, IFNb or TNFa at 8h, 24h, 48h and 72h, pre-gated on FSC/SSC, FSCA/FSCH, SSCW/SSCH, FSCA/DAPI$^-$ (n=3 biologically independent samples over at least 3 independent experiments for each condition). **B-D,** The percentage of H2K$^b$/ H2K$^b$SIINFEKL cell surface expression in CTRL versus Adam2 O/E LLC cells treated with IFNg (B), IFNb (C), or TNFa (D) (n=3 biologically independent samples over at least 3 independent experiments for each condition). Data presents mean ± s.e.m. analyzed by two-sided student's t-test. **E-G**, Total cell numbers of CTRL versus Adam2 O/E LLC cells expressing H2K$^b$/ H2K$^b$SIINFEKL treated with IFNg (E), IFNb (F), or TNFa (G) (n=3 biologically independent samples over at least 3 independent experiments for each condition). Data presents mean ± s.e.m. analyzed by two-sided student's t-test. **H,** Western blot analysis for Stat1 and P-Stat1 on CTRL and ADAM2 O/E LLC cells treated with 100ng/ml IFNg at indicated time points (n=3 independent experiments).

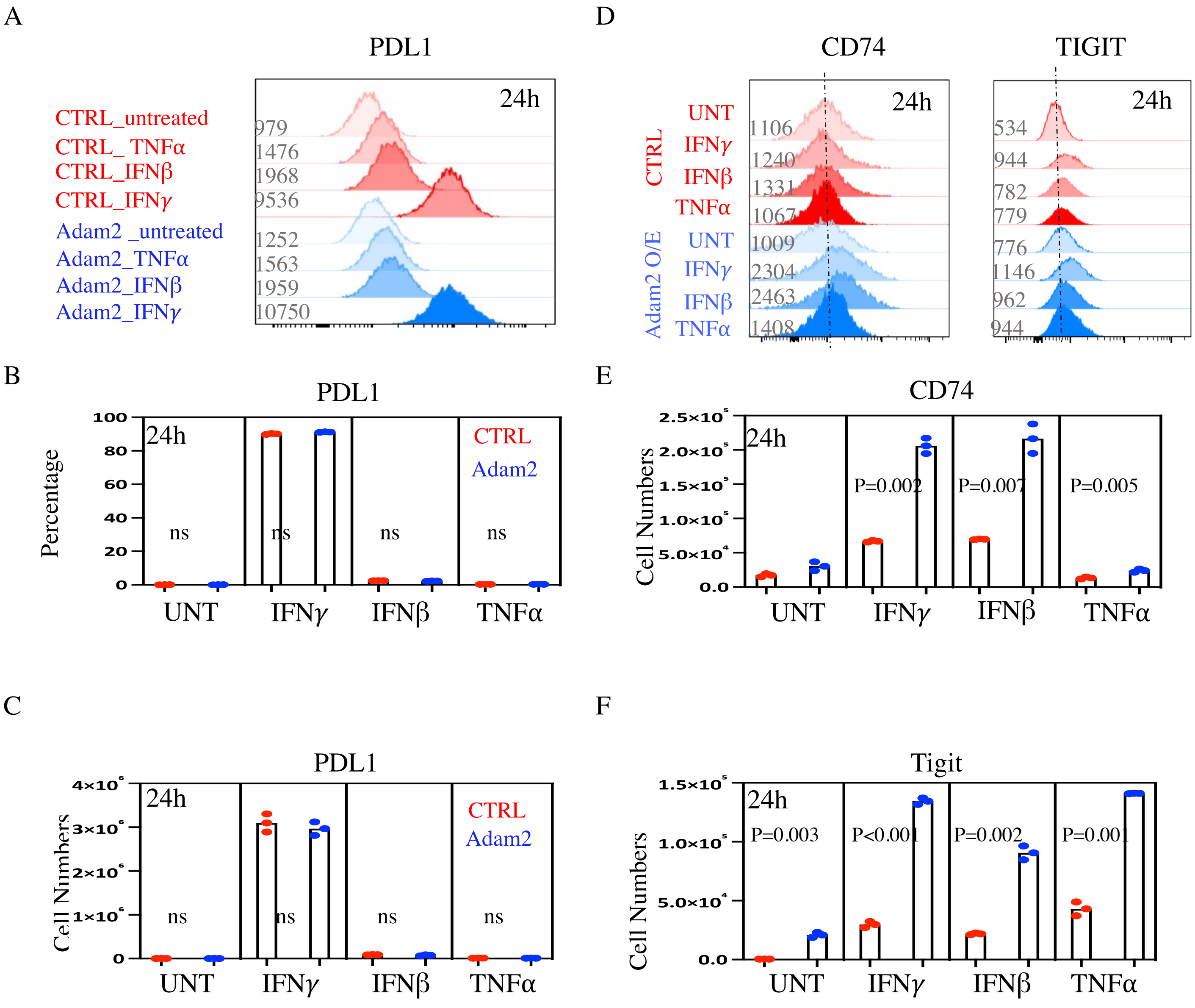


**Supplementary Figure 24: Adam2 regulates expression of IFN-regulated genes**

**A,** Flow cytometry analysis for cell surface expression of PDL1 on CTRL versus ADAM2 O/E cells untreated or treated with IFNg, IFNb or TNFa for 24h. FACS anlaysis was performed on pre-gated FSC/SSC, FSCA/FSCH, SSCW/SSCH, FSCA/DAPI- cells. **B,** Bar graph showing the percentage of PDL1 expressing CTRL and ADAM2 O/E cells from (A) (n=3 biologically independent samples over at least 3 independent experiments for each condition). Data presents mean ± s.e.m. analyzed by two-sided student's t-test. **C,** Bar graph showing the total cell numbers of PDL1 expressing CTRL and ADAM2 O/E cells from (A) (n=3 biologically independent samples over at least 3 independent experiments for each condition). Data presents mean ± s.e.m. analyzed by two-sided student's t-test. **D,** Flow cytometry analysis for cell surface expression of CD74 and Tigit on CTRL and ADAM2 O/E cells untreated or treated with IFNg, IFNb or TNFa for 24h. (n=3 biologically independent samples over at least 3 independent experiments for each condition). Data presents mean ± s.e.m. analyzed by two-sided student's t-test. **E** and **F**, Bar graph showing the total number of CTRL and ADAM2 O/E LLC cells expressing CD74 (E) and Tigit (F) untreated or treated with IFNg, IFNb or TNFa for 24h (n=3 biologically independent samples over at least 3 independent experiments for each condition). Data presents mean ± s.e.m. analyzed by two-sided student's t-test.

**Supplementary Figure 24**

A

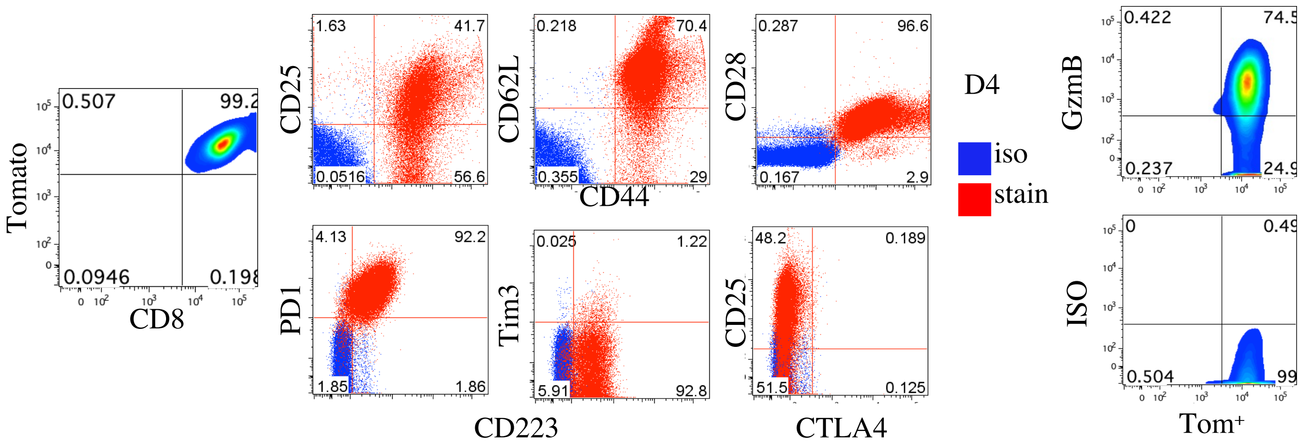


B

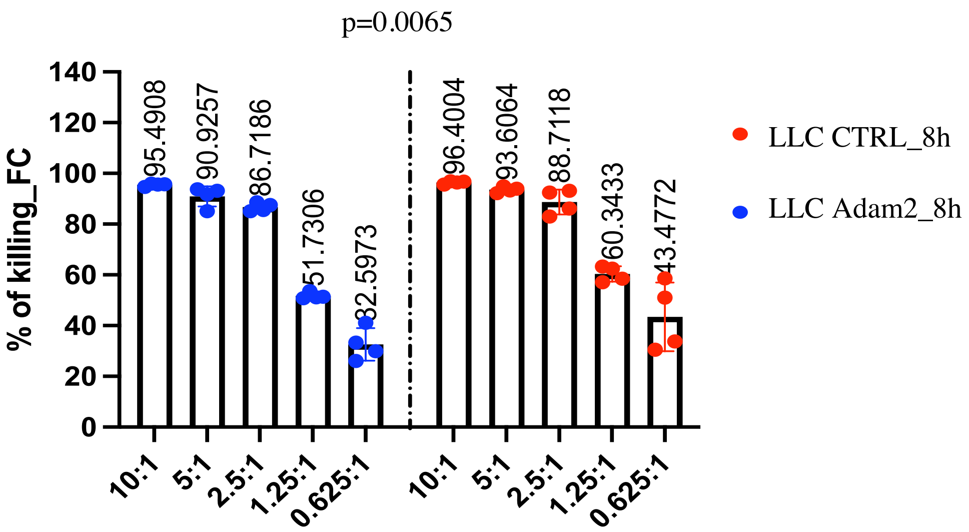


**Supplementary Figure 25: Overexpression of Adam2 in the LLC cells augments Ag-specific T-cell mediated killing**

**a,** Flow cytometry analysis of SIINFEKL-activated and expended OT-I splenocytes after 4 days showing cell expression of CD8 and Tomato, activation markers CD25 (IL2Rα), CD28, CD44, CD62L and exhaustion markers PD1, CD223 (Lag3), Tim3, CTLA4 and cytotoxic marker GzmB versus isotype control. Isotype control is shown in blue and stains in red (n=3 independent experiments for each condition). FACS anlysis was performed on pre-gated FSC/SSC, FSCA/FSCH, SSCW/SSCH, FSCA/DAPI$^{-}$ cells. **B,** The effect of Adam2 overexpression on OT-I cell-mediated killing was measured by flow cytometry. CTRL or Adam2 O/E cells labeled with CFSE were used as targets (T) and cocultured with OT-I cells (E) at different E:T ratio for 8h. The difference between overexpression and control conditions were measured by two-sided student's t-test (n=3 biologically independent samples over at least 3 independent experiments for each condition). Data presents mean **±** s.e.m. analyzed by two-sided student's t-test.

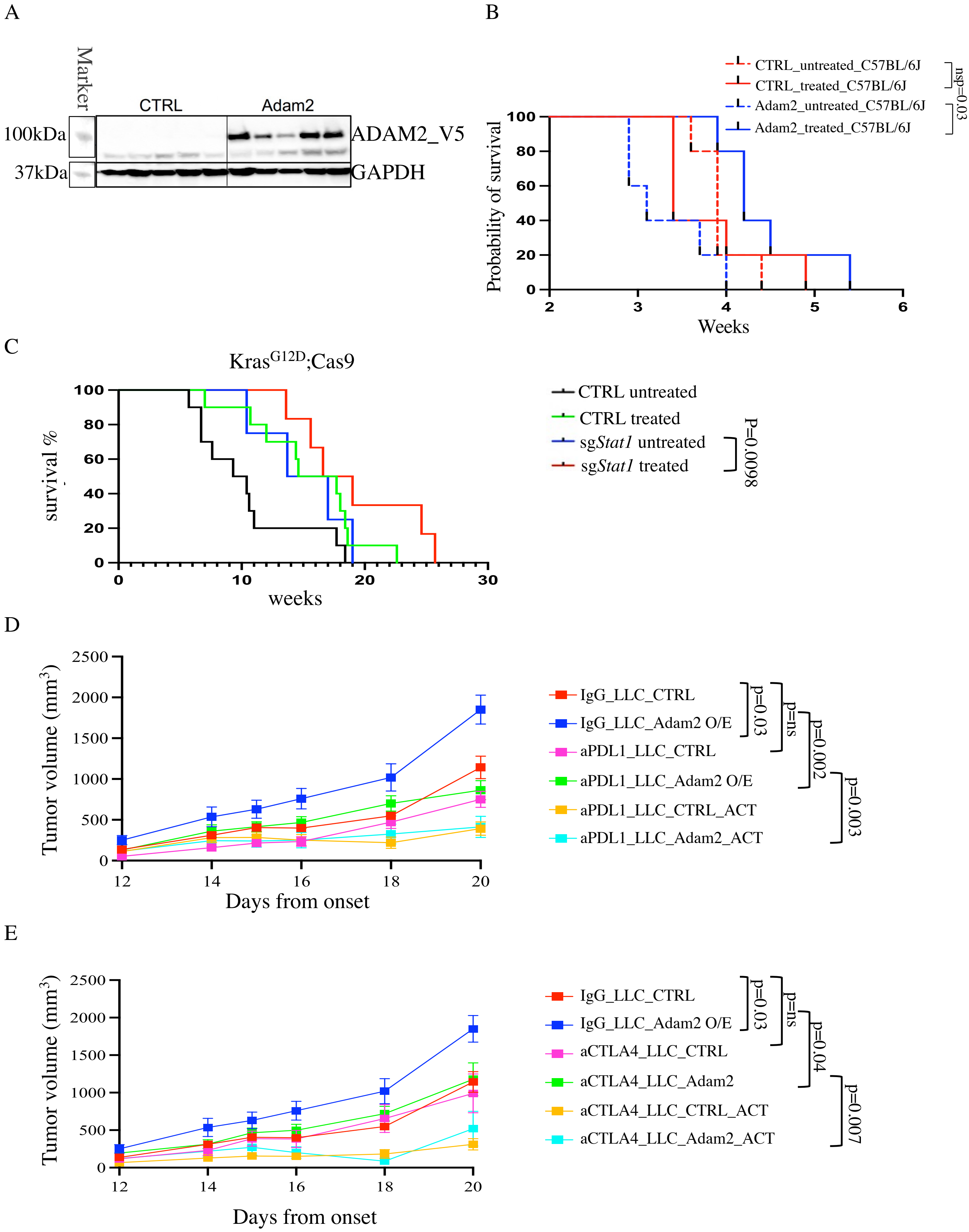


**Supplementary Figure 26**

**Supplementary Figure 26: Adam2 is reducing the inflammatory tumor milieu**

**A,** Western blot analysis showing expression of Adam2-V5 blotted for V5 tag and GAPDH in tumors isolated from s.c. transplanted CTRL (n=5) or Adam2 O/E (n=5) cells into untreated and ACT-treated C57BL/6 mice. **B,** Tumor free survival of C57BL/6 mice bearing CTRL (n=5) or Adam2 O/E tumors (n=5), untreated or treated with ACT of OT-I cells. Comparison of survival curves was performed by Log-rank (Mantel-Cox test). **C,** Tumor free survival of *Kras*$^{G12D}$;CAS9 mice transduced with sgNTC (untreated n=10; treated *n*=10) vs. sg*Stat1* (untreated *n*=4; treated n=6). Comparison of survival curves was performed by Log-rank (Mantel-Cox test). **D,** Tumor volume of s.c. transplanted CTRL or Adam2 O/E cells (0.1x10$^{6}$) in C57BL/6 mice, untreated or treated with OT-I cells in absence (IgG) or presence of PDL1 blocking antibody. Treatment was started once the tumor reached 100 mm$^{3}$. (n=5 for each group) **E,** Tumor volume of s.c. transplanted CTRL or Adam2 O/E cells (0.1x10$^{6}$) in C57BL/6 mice, untreated or treated with OT-I cells in absence (IgG) or presence of CTLA4 blocking antibody. Treatment was started once the tumor reached 100 mm$^{3}$. (n=5 for each group). **D-E**, The Wilcoxon matched-pairs signed-rank test was used to test for significant differences between untreated and treated groups. Data presents mean ± s.e.m. analyzed by two-sided student's t-test.

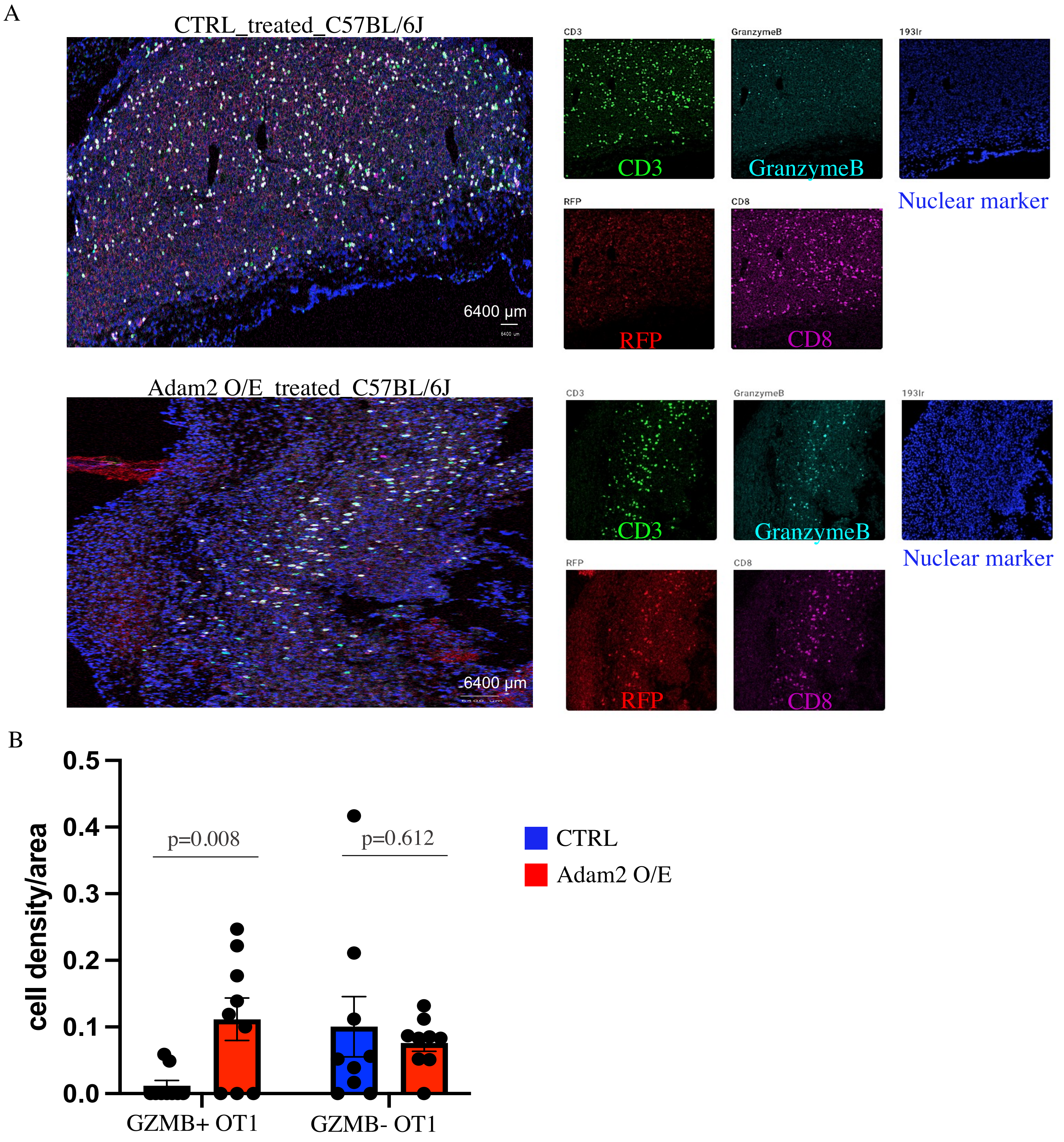


**Supplementary Figure 27: Adam2 O/E promotes LLC-specific T cell mediated cytotoxicity *in vivo***

**A,** Representative images of CTRL or Adam2 O/E LLC lung tumors isolated at end points. Increased infiltration of CD3$^+$CD8$^+$TOM$^+$GzmB$^+$ Ova-specific OTI cells was detected by IMC in Adam2 O/E tumors compared to CTRL LLC tumors. The scale bars represent 6400$\mu$m. **B,** Elevated numbers of GzmB$^+$ OT1 cell density/area were detected in Adam2 O/E compared to CTRL LLC tumors (n=4 biologically independent samples per each condition). Data presents mean ± s.e.m. analyzed by two-sided student's t-test.

**Supplementary Figure 27**

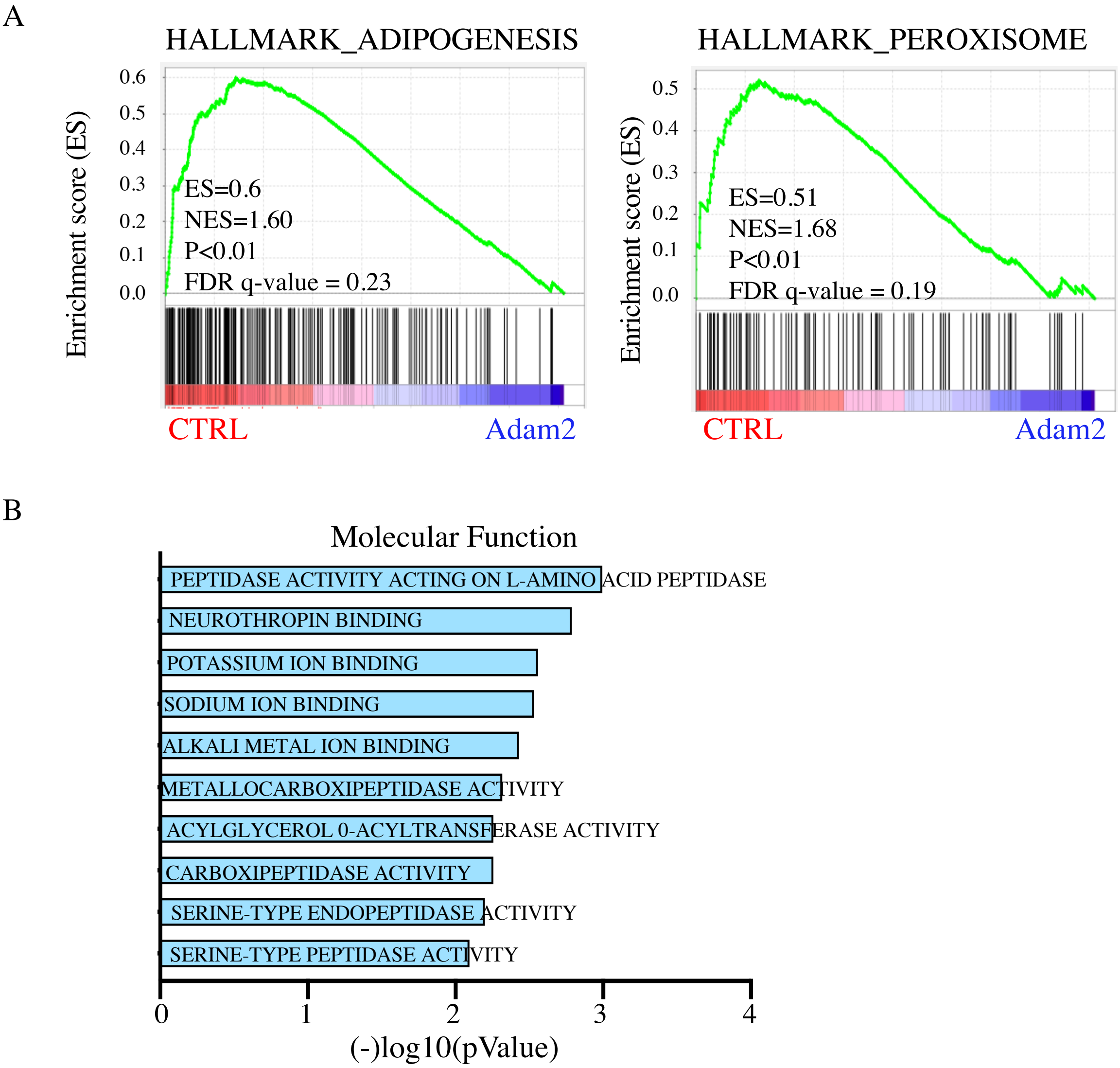


**Supplementary Figure 28: Regulatory network induced by Adam2 O/E after Ag-specific T cell transfer**
**A,** Gene set enrichment analysis (GSEA) revealed significantly downregulated genes ($p<0.01$, $FDR<0.25$) involved in adipogenesis and peroxisome function in Adam2 O/E tumors treated with ACT of OT-I cells. **B,** GSEA revealed downregulation of multiple molecular functions (eg. peptidase activity, neurothropin binding etc.) in Adam2 O/E LLC tumors compared to CTRL tumors, after treatment with ACT of OT-I cells, isolated from C57BL/6 mice. RNA seq data was performed on 3 independent biological experiments from each group. Bar graph show Gene Ontology of the DEGs ($FC>2$, $p<0.05$) downregulated in Adam2 O/E compared to CTRL lung tumors, after treatment with ACT of OT-I cells, assigned to molecular function.

**Supplementary Figure 28**

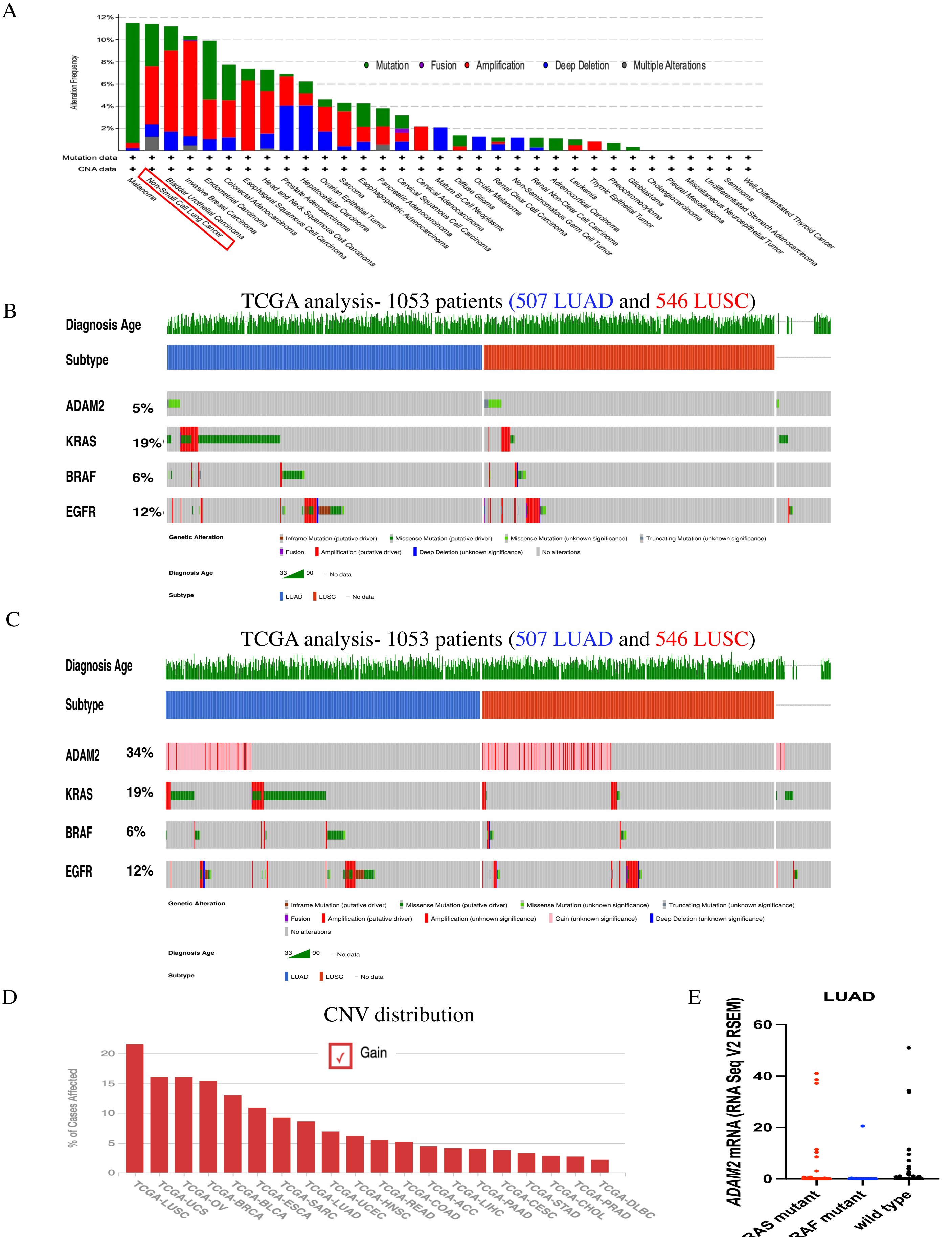


**Supplementary Figure 29**

**Supplementary Figure 29: Expression of ADAM2 in human LUAD**

**A,** TCAG analysis showing *ADAM2* alterations across different cancer types. **B** and **C,** TCAG analysis depicted as a cBioPortal OncoPrint reveals that 4% of LUAD (*n*=20/507) and 6% of LUSC (*n*=29/469) have *ADAM2* missense mutations, together account for 5% of these two lung cancer subtypes (B) and that 28% of LUAD (*n*=141/507) and 45% of LUSC (*n*=210/469) exhibit *ADAM2* gains, together account for 34% of these two lung cancer subtypes (C). **D,** CNV (copy number variant) distribution of *ADAM2* across TCGA cancer types. **E,** mRNA (RNA seq by expectation – maximization) of *ADAM2* and mutational status of *KRAS2* and *BRAF* in TCGA LUAD patients. Number of patients analyzed: 74 = *KRAS* mutation, 19 = *BRAF* mutation and 139 = WT for *BRAF* and *KRAS*.

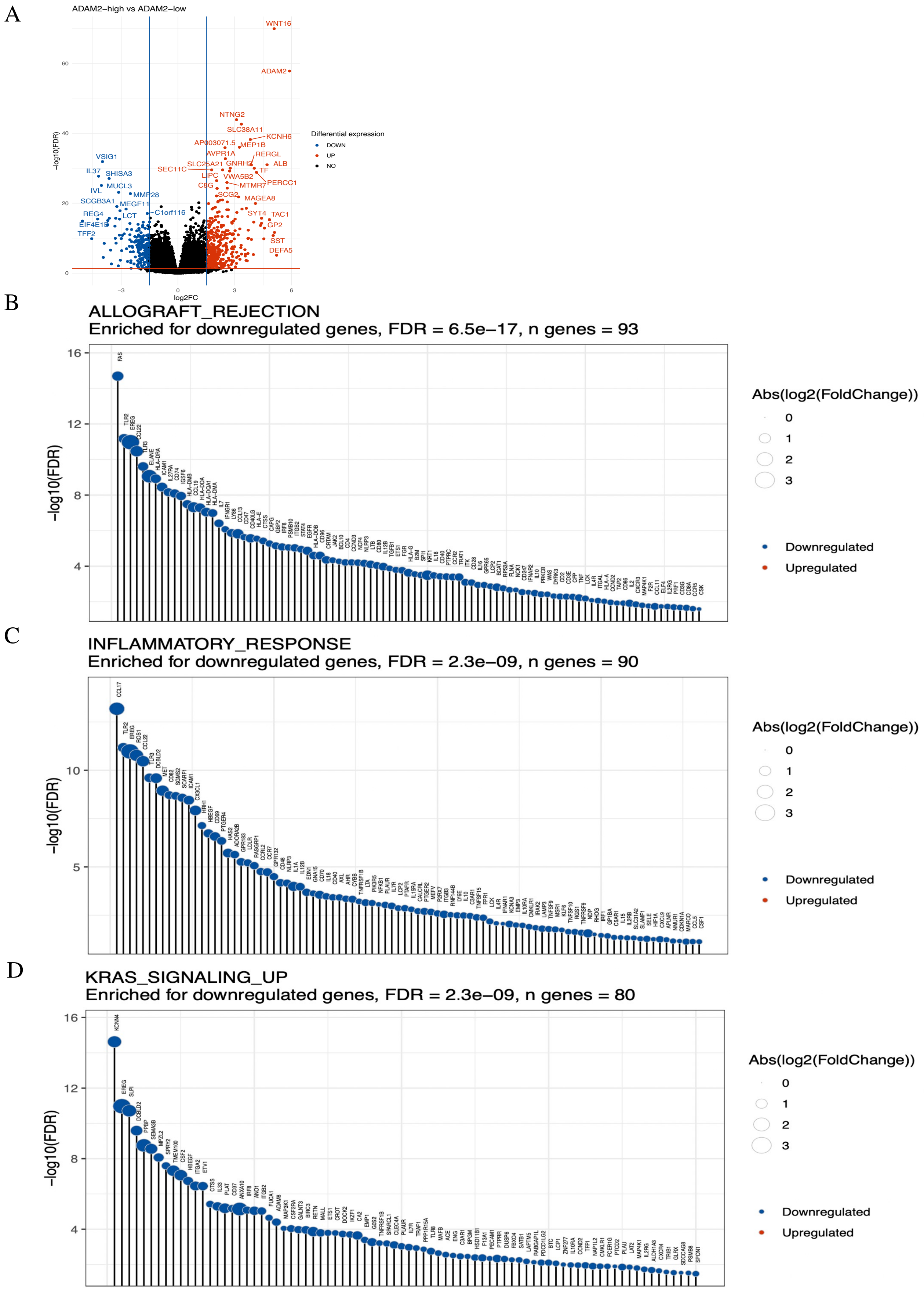


**Supplementary Figure 30**

**Supplementary Figure 30: GSEA of ADAM2-high versus ADAM2-low LUAD tumors**

**A,** Volcano plot showing differentially expressed genes between ADAM2 low=439 and ADAM2 high=71 TCGA LUAD tumors. Downregulated genes with FDR<0.05 and log2FC < 1.5 are highlighted in blue and upregulated genes with FDR<0.05 and log2FC > 1.5 are highlighted in red. A gene significance value cutoff of 0.05 and gene sets of 50 to 1000 genes were used as the parameters for ActivePathways. Significantly enriched pathways were highlighted (FDR < 0.05). **B-D,** GSEA for downregulated genes in the ADAM2-high group using the ActivePathways R package involved in Allograft rejection (B), Inflammatory response (C) and Kras signaling up (D) pathways. Plots show the genes in each pathway that contributed to its enrichment. Height represents significance, node size represents absolute value of the log2FC, and colour represents direction (blue=down, red=up). Enrichment analysis was performed separately for up and downregulated genes. Total number of patients analyzed=510.

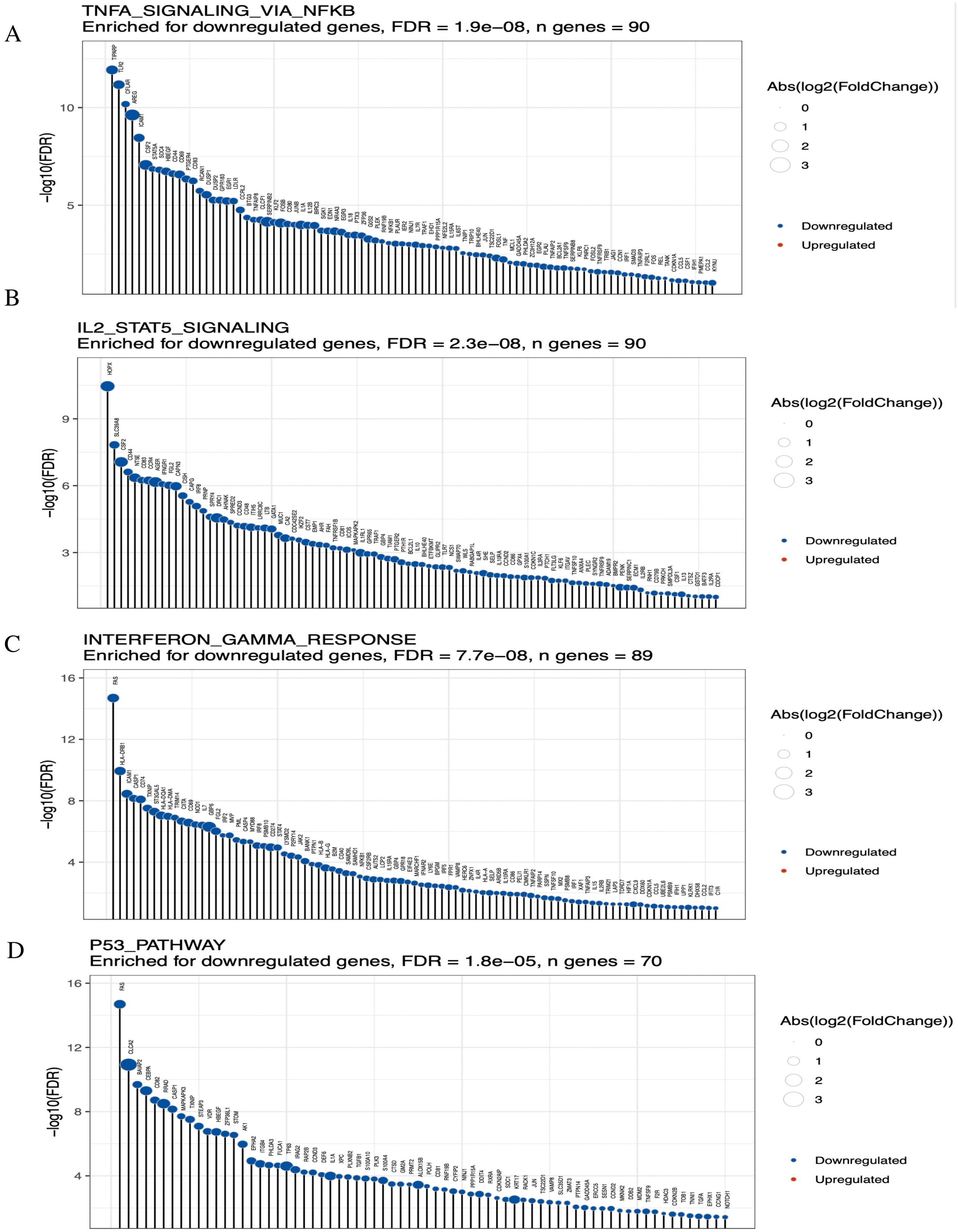


**Supplementary Figure 31: GSEA of ADAM2-high versus ADAM2-low LUAD tumors**

**A-D,** GSEA for downregulated genes in the ADAM2-high group using the ActivePathways R package involved in TNFA signaling via KFKB (A), IL2 STAT5 signaling (B), interferon gamma response (C) and p53 pathway (D). Total number of patients analyzed=510.

**Supplementary Figure 31**

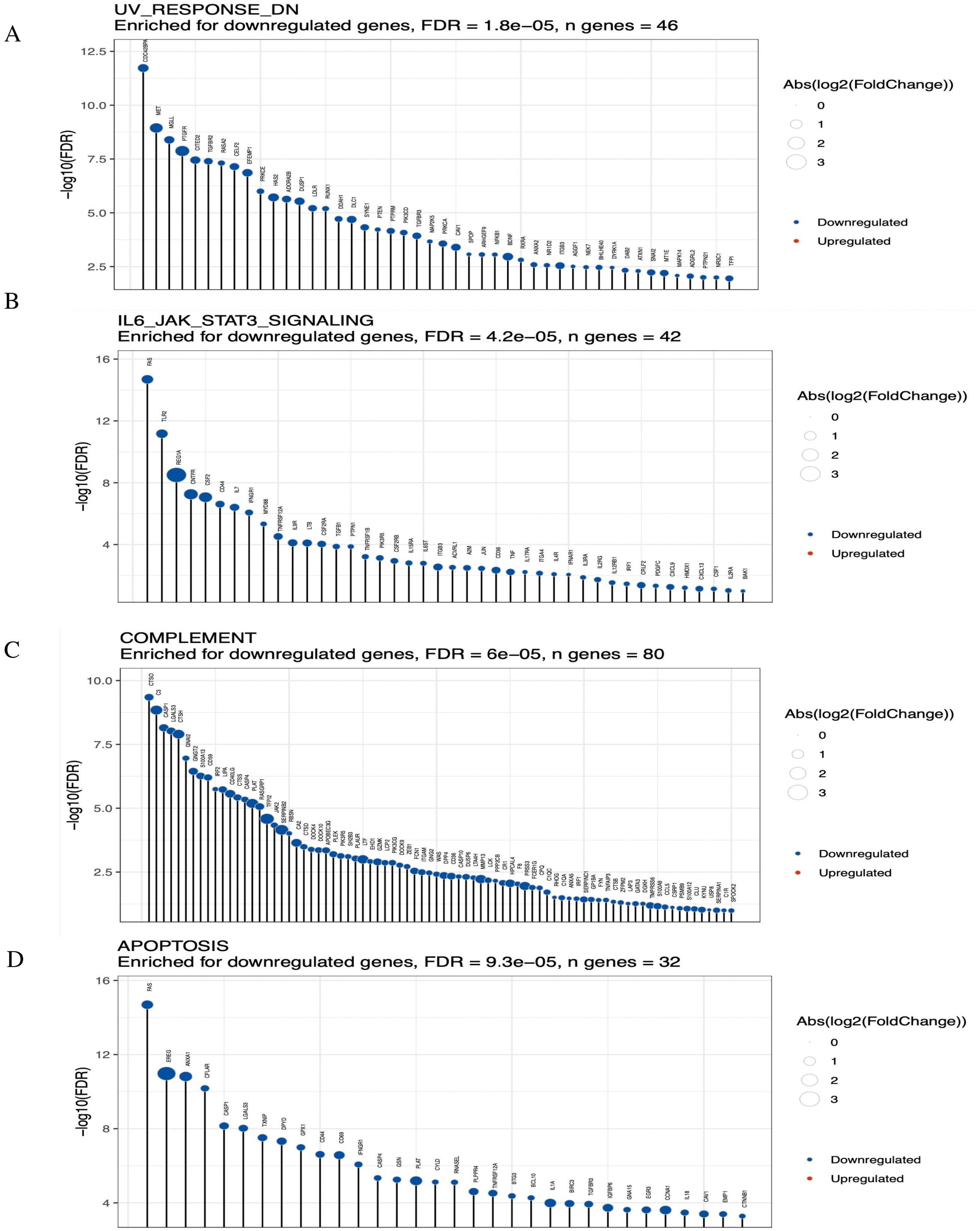


**Supplementary Figure 32: GSEA of ADAM2-high versus ADAM2-low LUAD tumors**

**A-D,** GSEA for downregulated genes in the ADAM2-high group using the ActivePathways R package involved in UV response (A), IL6-JAK_STAT3 signaling (B), complement (C) and apoptosis (D). Total number of patients analyzed=510.

**Supplementary Figure 32**

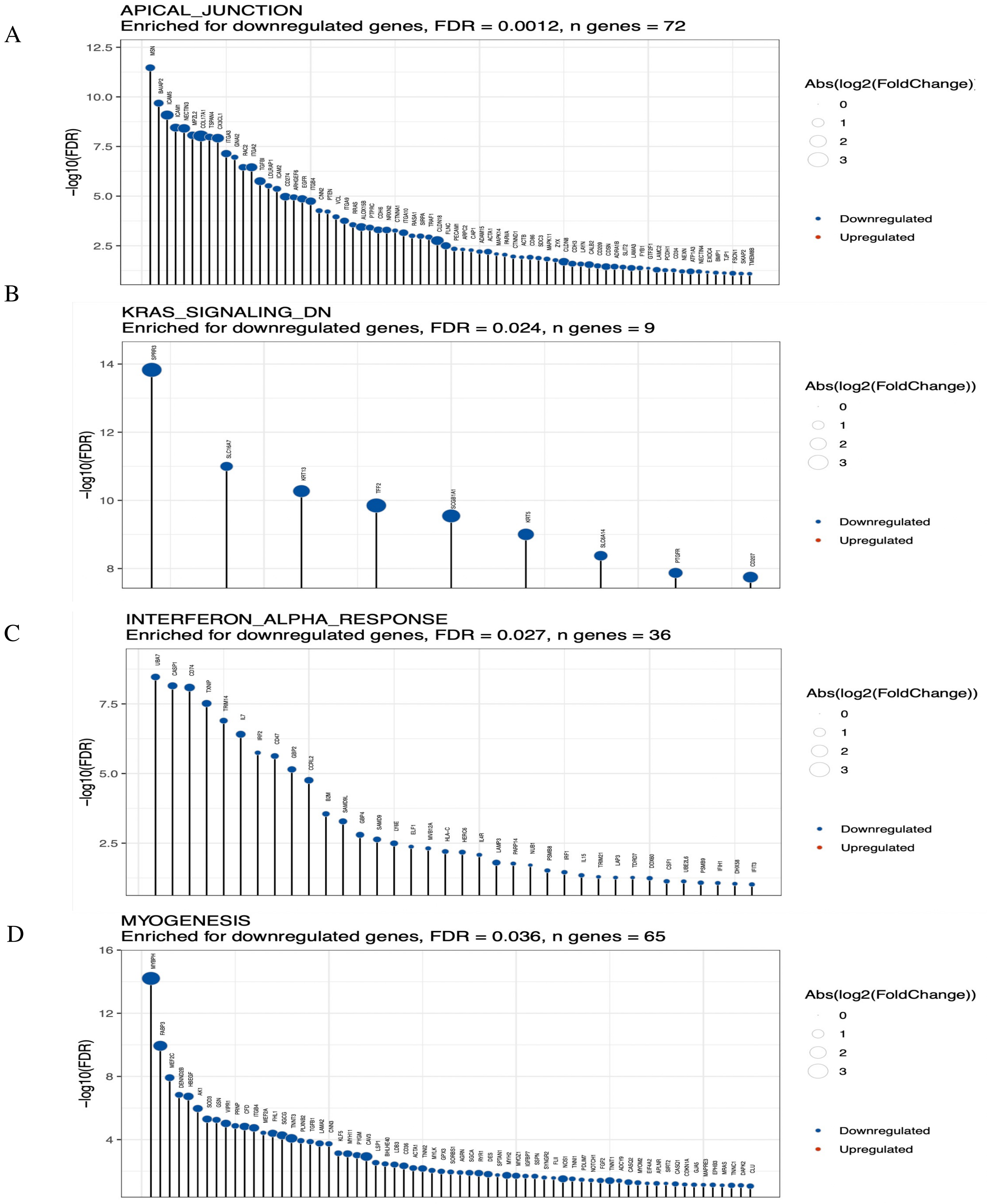


**Supplementary Figure 33: GSEA of ADAM2-high versus ADAM2-low LUAD tumors**
**A-D,** GSEA for downregulated genes in the ADAM2-high group using the ActivePathways R package involved in apical junction (A), Kras signaling (B), Interferon alpha response (C) and myogenesis (D). Total number of patients analyzed=510.

**Supplementary Figure 33**

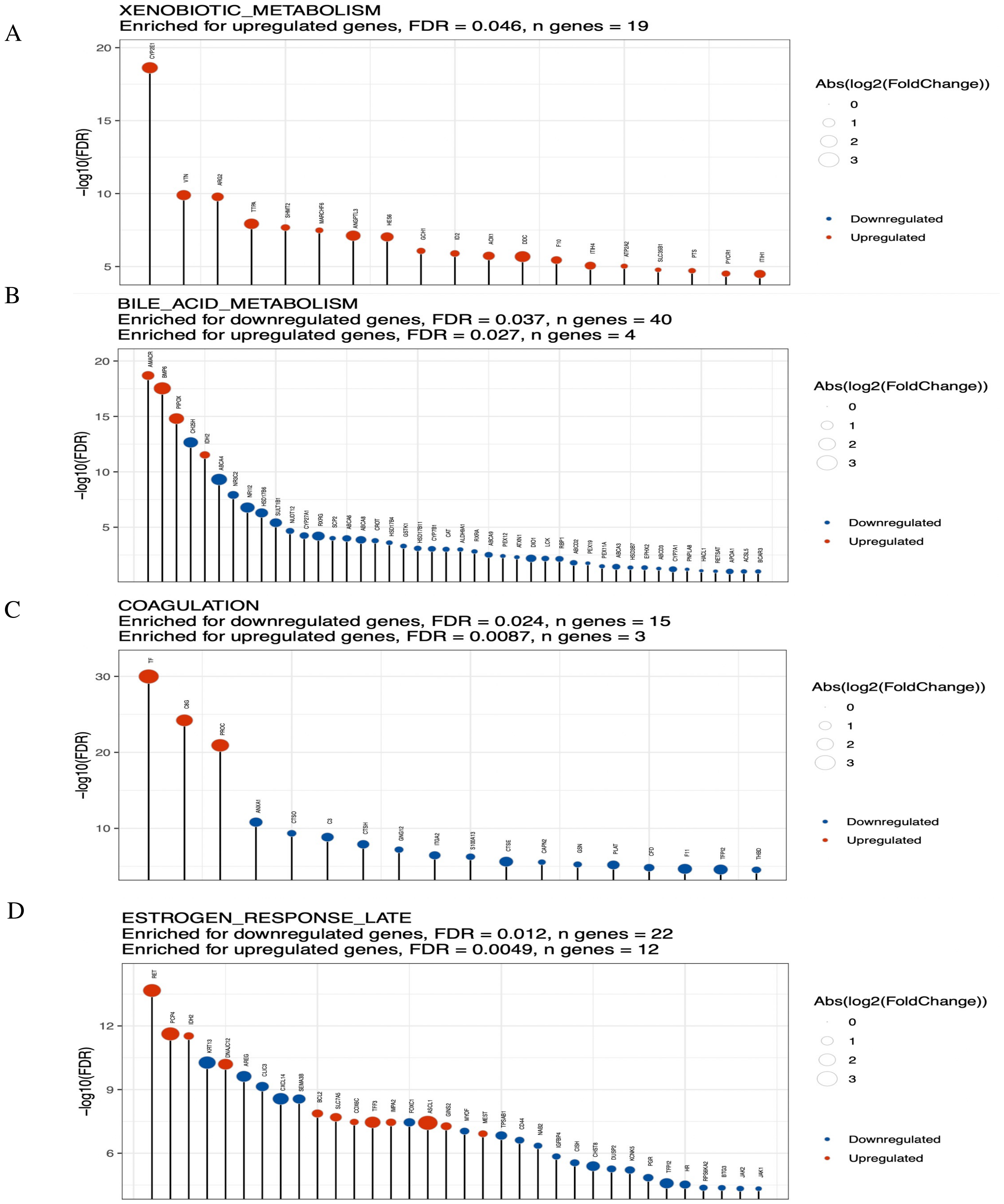


**Supplementary Figure 34: GSEA of ADAM2-high versus ADAM2-low LUAD tumors**

**A-D,** GSEA for downregulated/upregulated genes in the ADAM2-high group using the ActivePathways R package involved in Xenobiotic metabolism (A), Bile acid metabolism (B), Coagulation (C) and Estrogen response late (D). Total number of patients analyzed=510.

**Supplementary Figure 34**

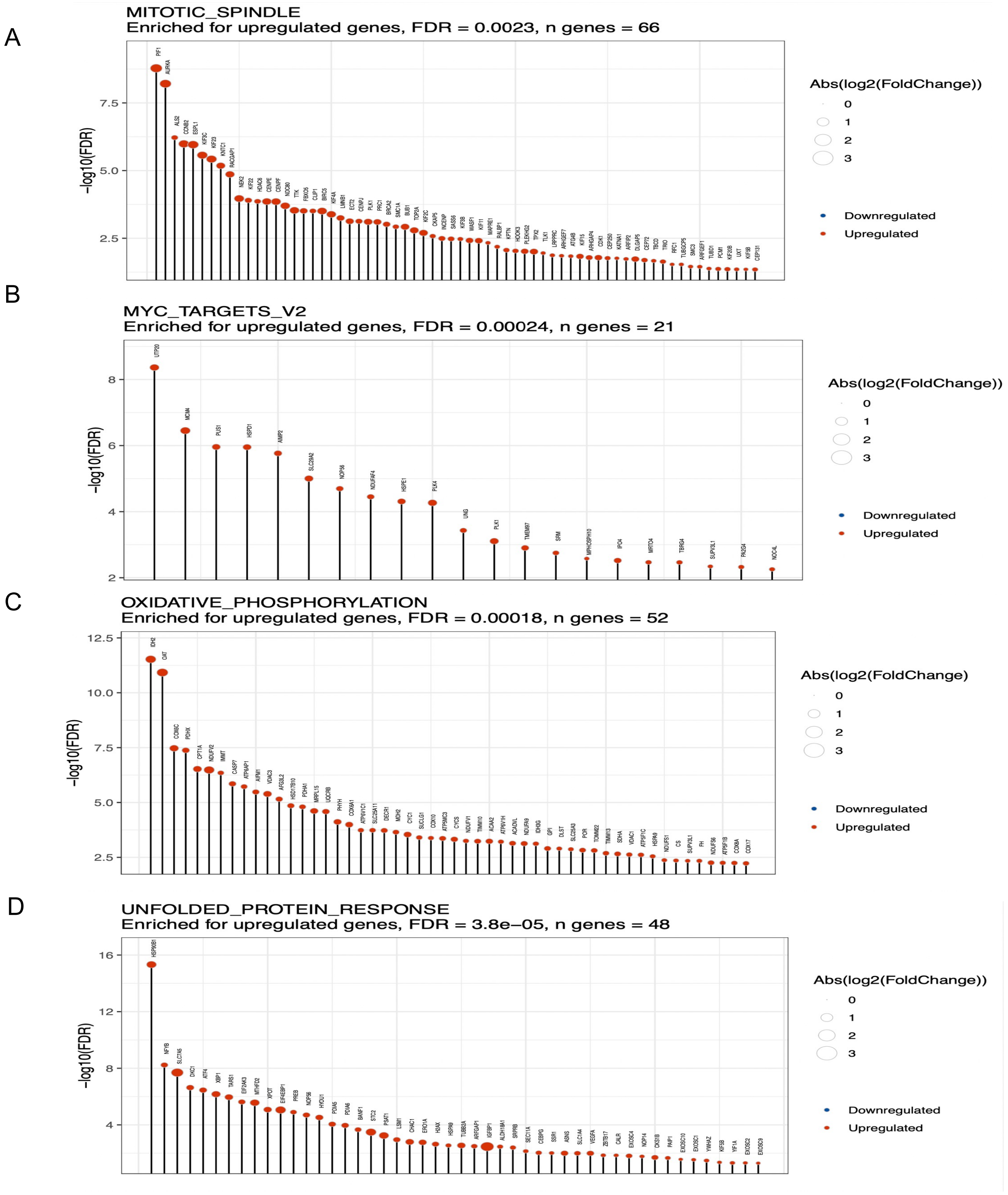


**Supplementary Figure 35: GSEA of ADAM2-high versus ADAM2-low LUAD tumors**
**A-D,** GSEA for upregulated genes in the ADAM2-high group using the ActivePathways R package involved in Mitotic spindle (A), Myc target (B), Oxidative phosphorylation (C) and Unfolded protein response (D). Total number of patients analyzed=510.

**Supplementary Figure 35**

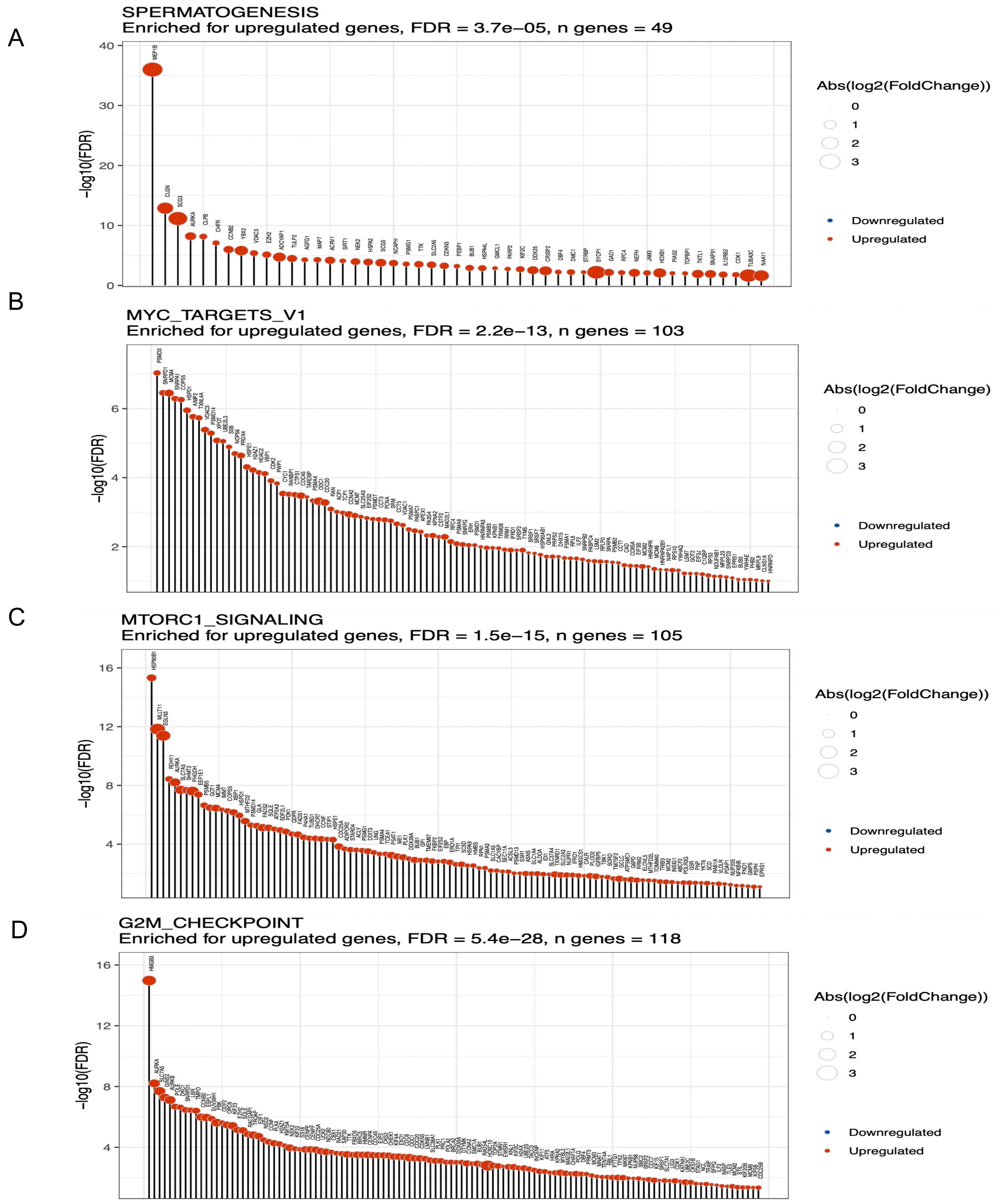


**Supplementary Figure 36: GSEA of ADAM2-high versus ADAM2-low LUAD tumors**

**A-D,** GSEA for upregulated genes in the ADAM2-high group using the ActivePathways R package involved in Spermatogenesis (A), Myc target V1 (B), MTORC1 signaling (C) and G2M checkpoint (D). Total number of patients analyzed=510.

**Supplementary Figure 36**

A

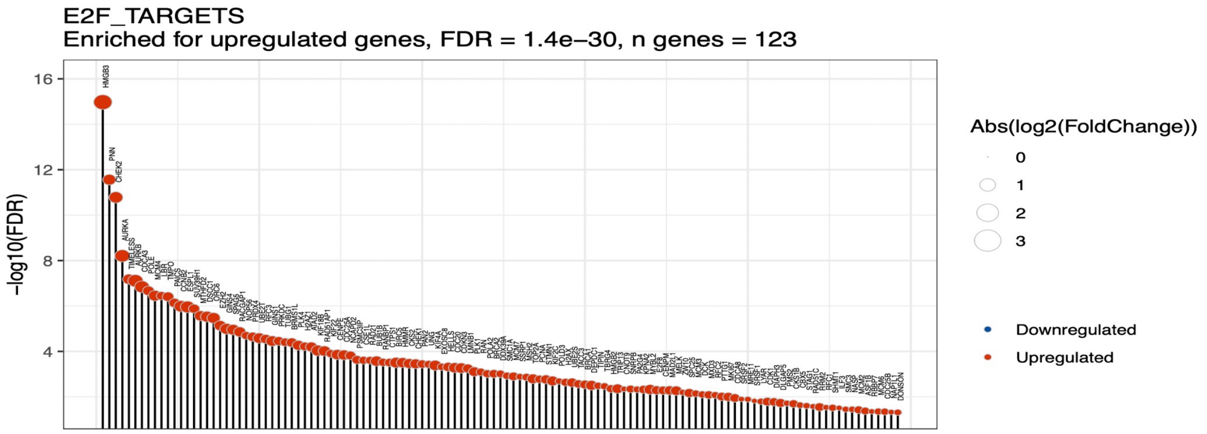


**Supplementary Figure 37: GSEA of ADAM2-high versus ADAM2-low LUAD tumors**

**A,** GSEA for upregulated genes in the ADAM2-high group using the ActivePathways R package involved in E2F targets. Total number of patients analyzed=510.

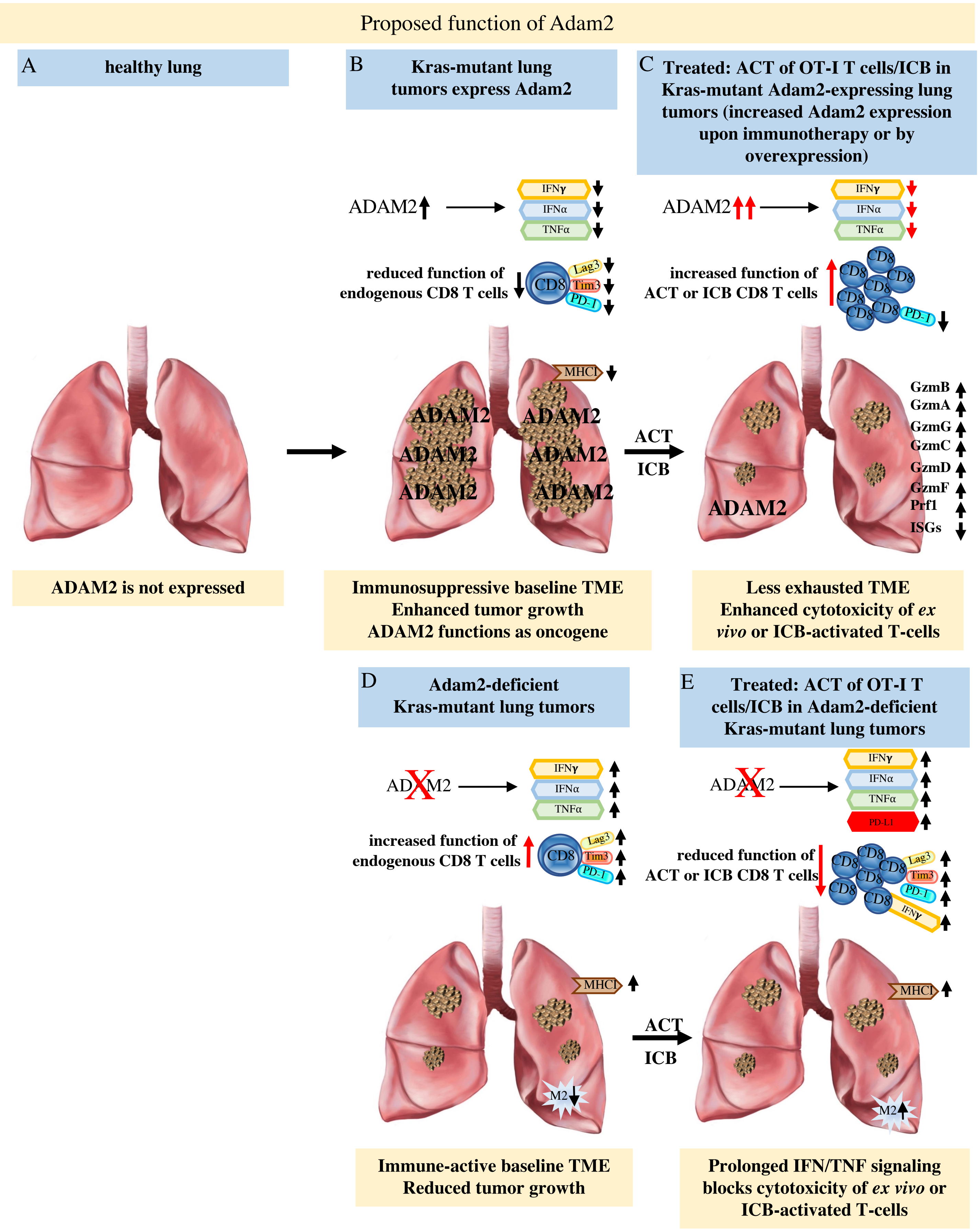


**Supplementary Figure 38**

**Supplementary Figure 38: Proposed functions of ADAM2**

A, Adam2 is absent in normal lung.

B, Adam2 is aberrantly expressed in KRas-mutant lung tumors and functions as an oncogene by reducing IFN/TNF-signaling, reducing MHC-I presentation. As such, Adam2 functions as an immune suppressant in this setting. Forced expression of ADAM2 in LLC cells corroborated these findings and also triggered a less exhausted TME marked by reduced IFN/TNF-signaling, reduced MHC-presentation and rapid outgrowth of tumors (in an immune-system dependent manner).

C, Treated KRas-mutant lung tumors exhibit further elevated Adam2 expression levels and a good response to adoptive transfer of OT1 T-cells and immune checkpoint blockade (ICB). Forced expression of Adam2 in LLC cells treated with ACT or ICB showed that Adam2 overexpression indeed sensitizes TME to cancer immunotherapy marked by significantly elevated expression levels of granzymes and perforin and reduced expression of PD-1 on infiltrating Ag-specific cytotoxic T-cells.

D, Genetically ablating Adam2 in KRas-mutant lung tumors leads to increased IFN/TNF signaling and MHC presentation, increased CD8 cells and decreased protumoral M2 macrophages and overall better immune control and less tumor burden.

E, Loss of Adam2 blocks ACT and ICB: The prolonged IFN/TNF-signaling observed in KRas-mutant Adam2 KO lung tumors promotes T-cell exhaustion and blocks cytotoxicity of treated lung tumors by *ex vivo*- or ICB-activated T-cells marked by increased expression of PD-1, PD-L1, TIGIT and LAG3 as well as IFN and TNFs.

Lung picture was generated by courtesy of Ella Fific.

Reference:
1. Zhou et al., Metascape provides a biologist-oriented resource for the analysis of systems-level datasets, Nature Communications, **10**, Article number: 1523 (2019)
