# Supplementary material for: In vivo CRISPR screens reveal Serpinb9 and Adam2 as regulators of immune therapy response in lung cancer
Source: Nat Commun. 2023 May 31;14:3150. doi: 10.1038/s41467-023-38841-7 (PMC10232477; doi:10.1038/s41467-023-38841-7)
Supplement: Supplementary file 3 — Description of Additional Supplementary Files [file 41467_2023_38841_MOESM3_ESM.pdf]

Description of Additional Supplementary Files

Supplementary Data 1. List of multi-guide sgRNA libraries used in this manuscript related to Main Figure 1 F and G; 3 A and B; Supplementary Information 1C; 4 A-C; 5 A-D; 9 A

Sheet 1 lists all the genes, sgRNAs as well as oligo sequences used to clone the immune gene library.
Sheet 2 lists all the genes, sgRNAs as well as oligo sequences used to clone the non-targeting control library.
Sheet 2 lists the primers used to clone the immune gene as well as the non-targeting control library.

Supplementary Data 2. Summary of NGS counts and library coverage related to Supplementary Information 1C

List of all the genes, guides and counts for all sgRNAs targeting immune genes in plasmid vs. transduced mouse embryonic fibroblasts (MEFs).

Supplementary Data 3. Summary of NGS counts and coverage in Kras^{G12D} and Braf^{V600E} lungs transduced with Immune library related to Main Figure 1 F and G; 3 A and B; Supplementary Information 1C; 4 A-C; 5 A-D; 9 A

Sheet 1 lists Kras^{G12D} Lung gene summary
Sheet 2 lists Kras^{G12D} Lung sgRNA counts normalized
Sheet 3 lists Braf^{V600E} Lung gene summary
Sheet 4 lists Kras^{V600E} Lung sgRNA counts normalized

Supplementary Data 4. List of DESeq2 LFC from RNA sequencing data _GSEA_MetaScape_gProfiler in Adam2 KO compared to CTRL tumors related to Main Figure 3 G-H; Supplementary Information 4 A-C

Sheet 1 DESeq2LFC from Adam2 ko vs. control lung tumors
Sheet 2 GSEA report for positively regulated genes in Adam2 ko lungs
Sheet 3 GSEA report for negatively regulated genes in Adam2 ko lungs
Sheet 4 Metascape Annotation
Sheet 5 Metascape Enrichment of biological processes enriched in Adam2 ko lungs
Sheet 5 gProfiler homo sapiens of pathways enriched in Adam2 ko lungs

Supplementary Data 5. ADAM2 mRNA expression in Pan Cancer, LUAD and LUSC from TCGA datasets related to Supplementary Information 9 B; 29 A-D

Sheet 1 lists the expression level, copy number alterations and mutations of *ADAM2* in 510 lung adenocarcinoma (LUAD) TCGA tumors.
Sheet 2 lists the expression level, copy number alterations and mutations of *ADAM2* in 498 lung squamous cell carcinoma (LUSC) TCGA tumors.
Sheet 3 lists the expression level, copy number alterations and mutations of *ADAM2* in 8424 TCGA tumors of the indicate cancer type (pan cancer analysis).

Supplementary Data 6. ADAM2 mRNA expression in Kras and Braf LUAD analysed from TCGA database related to Main Figure 6A; Supplementary Figure 29E

Supplementary Data 7. Differential expression and pathway enrichment analysis of TCGA LUAD samples with ADAM2 expression related to Main Figure 6D; Supplementary Information 30-37

Sheet 1 Differential expression analysis of TCGA LUAD samples with ADAM2 expression

Sheet 2 Pathway enrichment analysis of TCGA LUAD samples with ADAM2 expression

Supplementary Data 8. List of animal model, antibodies, cell lines and primers used in the study

Sheet 1 List of mouse models used in the study

Sheet 2 List of flow cytometry antibodies used in the study

Sheet 3 List of IHC antibodies used in the study

Sheet 4 List of IMC antibodies used in the study

Sheet 4 List of WB antibodies used in the study

Sheet 5 List of cytokines and proteome profiler mouse cytokine array kit used in the study

Sheet 6 List of RNA specific probes used for RNA scope analysis

Sheet 6 Cell lines used in the study

Sheet 7 QRT primers used in the study

Sheet 8 List of antibodies used in CyTOF analysis
