# Supplementary material for: In vivo CRISPR screens reveal Serpinb9 and Adam2 as regulators of immune therapy response in lung cancer
Source: Nat Commun. 2023 May 31;14:3150. doi: 10.1038/s41467-023-38841-7 (PMC10232477; doi:10.1038/s41467-023-38841-7)
Supplement: Supplementary file 12 — Reporting Summary [file 41467_2023_38841_MOESM12_ESM.pdf]

nature portfolio

Corresponding author(s): Daniel Schramek

Last updated by author(s): May 8, 2023

Reporting Summary

Nature Portfolio wishes to improve the reproducibility of the work that we publish. This form provides structure for consistency and transparency in reporting. For further information on Nature Portfolio policies, see our Editorial Policies and the Editorial Policy Checklist.

Statistics

For all statistical analyses, confirm that the following items are present in the figure legend, table legend, main text, or Methods section.

n/a	Confirmed	
☐	☒	The exact sample size (*n*) for each experimental group/condition, given as a discrete number and unit of measurement
☐	☒	A statement on whether measurements were taken from distinct samples or whether the same sample was measured repeatedly
☐	☒	The statistical test(s) used AND whether they are one- or two-sided<br>*Only common tests should be described solely by name; describe more complex techniques in the Methods section.*
☒	☐	A description of all covariates tested
☐	☒	A description of any assumptions or corrections, such as tests of normality and adjustment for multiple comparisons
☐	☒	A full description of the statistical parameters including central tendency (e.g. means) or other basic estimates (e.g. regression coefficient) AND variation (e.g. standard deviation) or associated estimates of uncertainty (e.g. confidence intervals)
☐	☒	For null hypothesis testing, the test statistic (e.g. *F*, *t*, *r*) with confidence intervals, effect sizes, degrees of freedom and *P* value noted<br>*Give P values as exact values whenever suitable.*
☒	☐	For Bayesian analysis, information on the choice of priors and Markov chain Monte Carlo settings
☒	☐	For hierarchical and complex designs, identification of the appropriate level for tests and full reporting of outcomes
☐	☒	Estimates of effect sizes (e.g. Cohen's *d*, Pearson's *r*), indicating how they were calculated

Our web collection on statistics for biologists contains articles on many of the points above.

Software and code

Policy information about availability of computer code

Data collection	Illumina HiSeq2000, Nikon Elements (NIS ElementsAR ver. 4.6.0), CFX384 (Biorad), Backman Coulter MoFlo Astrios EQ, Cell Sorter, BD Fortessa X-20, Hyperion Imaging System (Fluidigm); Helios instrument,
Data analysis	ZEISS ZEN microscopy software; NDP.view 2.9.25; Fiji 2.1.0; QuPath 0.3.2; Hisat2/bowtie2 version 2.1.0 and counts were obtained using featureCounts (Subread package version 1.6.3); Python 3.8; Model-based Analysis of Genome-wide CRISPR-Cas9 Knockout (MAGeCK) algorithm (https://sourceforge.net/p/mageck/wiki/Home/); MAGeCKFlute (https://bioconductor.org/packages/release/bioc/html/MAGeCKFlute.html); Atom 1.58.0x64; GSEA 4.2.0; TIDE.nki.nl (https://tide.nki.nl); ICE synthego (https://ice.synthego.com/#/); FlowJo version 9.9.6 and 10.8.1; Primer3Plus (https://www.primer3plus.com), GraphPad Prism 9.1.0; Unnormalized gene expression counts (STAR); TCGAbiolinks R (v. 2.18.0); DESeq2 R package (v. 1.30.1); ActivePathways R package (v. 1.1.1); MSigDB database; ImcPQ pipline JacksonGroupLTRI; PhenoGraph (v.2.0); Helios software (v.6.7.1014); CytoBank; R-4.0, R studio software program (v.3.6.2); Microsoft Excell (v.16.5); Snapgene (v.6.0.5); Metascape A Gene Annotation & Analysis Resource (https://metascape.org/gp/index.html#/main/step1)

For manuscripts utilizing custom algorithms or software that are central to the research but not yet described in published literature, software must be made available to editors and reviewers. We strongly encourage code deposition in a community repository (e.g. GitHub). See the Nature Portfolio guidelines for submitting code & software for further information.

Data

Policy information about availability of data

All manuscripts must include a data availability statement. This statement should provide the following information, where applicable:

- Accession codes, unique identifiers, or web links for publicly available datasets
- A description of any restrictions on data availability
- For clinical datasets or third party data, please ensure that the statement adheres to our policy

All RNAseq data are available in at NCBI Gene Expression Ominbus GEO accession GSE200628. RNA sequencing data were aligned to the GRCm38 - mm10 Mouse Genome Reference Consortium. Custom designed Immune gene targeting library was selected from Rooney et al, 2015 study. Screening data are presented in Supplementary Data Table 1,2,3. DESeq2 GSEA MetaScape gProfiler data are presented in Supplementary Data Table 4. The TCGA Pan Cancer, LUAD, LUSC for ADAM2 mRNA expression are presented in Supplementary Data Table 5,6. The pathway enrichment analysis of TCGA LUAD samples with ADAM2 expression representing the 50 cancer hallmark pathways from the MSigDB database are presented in Supplementary Data Table 7.

Human research participants

Policy information about studies involving human research participants and Sex and Gender in Research.

Reporting on sex and gender	Sex or gender of healthy donors is not publicly available.
Population characteristics	Age or gender of healthy donors is not publicaly available.
Recruitment	Healthy donors - volunteers.
Ethics oversight	Blood samples from volunteers were collected with informed consent using a protocol (#05-0221-T) approved by University Health Network (UHN) Research Ethics Board and kid gift provided by Dr. Li Zhang.

Note that full information on the approval of the study protocol must also be provided in the manuscript.

Field-specific reporting

Please select the one below that is the best fit for your research. If you are not sure, read the appropriate sections before making your selection.

☒ Life sciences ☐ Behavioural & social sciences ☐ Ecological, evolutionary & environmental sciences

For a reference copy of the document with all sections, see nature.com/documents/nr-reporting-summary-flat.pdf

Life sciences study design

All studies must disclose on these points even when the disclosure is negative.

Sample size	No statistical methods were used to pre-determine sample sizes. Sample size for in vivo screening studies were empirically determined using barcode library recovery studies such that adequate coverage (>200 fold) of screening library could be achieved. Sample sizes for other in vivo experiments such as target validation, with or without use of the checkpoint inhibitors, was determined based on prior knowledge of typical biological variability with the tumor models and immunotherapy treatments.
Data exclusions	No data were excluded from the study.
Replication	Results presented in the manuscript were replicated at least twice in independent experiments or in two complementary tumor models. Results between replicates were reproducible.
Randomization	Allocation of experimental animals to the treatment groups were randomized to treatment condition(s) by cage following tumor onset. Covariant are not relevant to this study as isogenic mice with clean genetic background were used. In vitro experiments were performed using identical cellular preparations and were randomly assigned to the different experimental conditions.
Blinding	All tumor measurements conducted in this study were blinded to group allocation. All bioinformatics studies were automated and can be considered as blinded. The researchers conducting TCGA analysis of human LUAD samples and QRT analysis were blinded to group allocation. For experiments other than mentioned here, investigators were not blinded to treatment groups or genotypes, as knowledge of this information was essential to conduct the studies. Experiments performed with expended human double negative T cells were blinded.

Reporting for specific materials, systems and methods

We require information from authors about some types of materials, experimental systems and methods used in many studies. Here, indicate whether each material, system or method listed is relevant to your study. If you are not sure if a list item applies to your research, read the appropriate section before selecting a response.

Materials & experimental systems

n/a	Involved in the study
	x Antibodies
	x Eukaryotic cell lines
x	Palaeontology and archaeology
	x Animals and other organisms
x	Clinical data
x	Dual use research of concern

Methods

n/a	Involved in the study
x	ChIP-seq
	x Flow cytometry
x	MRI-based neuroimaging

Antibodies

Antibodies used	Antibodies used in this study are listed in Supplementary Table 8.
Validation	Flow cytometry:<br>CD3 Purified antimouse; Induction of a cellular active state from a normal resting state, C57BLK6 splenocytes; Manufacturer<br>CD3 FITC antimouse; C57BL/6 mouse splenocytes; Manufacturer<br>CD3 PerCP/Cyanine5.5 anti-mouse; C57BL/6 mouse splenocytes ; Manufacturer<br>CD3 UltraLEAF Purified antihuman; Human PBMCs; Manufacturer<br>CD4 PECY7 antimouse; C57BL/6 mouse splenocytes; Manufacturer<br>CD8a APCCy7 Rat antiMouse; C57BL/6 mouse splenocytes were stained with CD3 FITC and CD8; Manufacturer<br>CD8a BV711 Rat antiMouse; C57BL/6 mouse splenocytes were stained with CD3 PE and CD8a; Manufacturer<br>CD8a APCCy7 antimouse; C57BL/6 mouse splenocytes were stained with CD3 PE and CD8a; Manufacturer<br>CD11b FITC antimousehuman; C57BL/6 mouse bone marrow cells were stained with CD11b; Manufacturer<br>CD11c APC antimouse; C57BL/6 mouse splenocytes; Manufacturer<br>CD11c FITC antimouse; 57BL/6 mouse splenocytes stained with APC anti-mouse I-A/I-E (clone M5/114.15.2) and FITC N418; Manufacturer<br>CD25 APC antimouse; Con A-stimulated (3 days)splenocytes stained with PC61 APC; Manufacturer<br>CD39 APC antimouse; C57BL/6 Mouse splenocytes were stained with CD4 FITC, CD25 PE, and CD39 (clone Duha59) APC; Manufacturer<br>CD44 BV421 antimouse; C57BL/6 mouse splenocytes were stained with CD44 (clone IM7) Brilliant Violet 421; Manufacturer<br>CD44 PE Rat antiMouse; C57BL/6 mouse splenocytes; Manufacturer<br>CD45RB PerCPCy55antimouse; C57BL/6 mouse splenocytes; Manufacturer<br>CD45.2 BV 711 antimouse; C57BL/6 mouse splenocytes; Manufacturer<br>CD45 FITC antimouse; C57BL/6 mouse splenocytes; Manufacturer<br>CD74 BV605 Rat antiMouse; CD74 Mouse (Tested in Development); Manufacturer<br>TIGIT APC antimouse; TIGIT Vstm3 Antibody ConA-stimulated (3 days) C57BL/6 mouse splenocytes; Manufacturer<br>CD326 EpCAM PE antimouse; TE-71 (mouse thymic epithelial stromal cell line) cells were stained with CD326 (clone G8.8) PE; Manufacturer<br>CD326 EpCAM PerCP/Cyanine5.5 anti-mouse; Mouse thymic epithelial stromal cell line (TE-71) stained with G8.8 PerCP/Cyanine5.5; Manufacturer<br>CD366 TIM3 PE Mouse antiMouse; BALB/c anti-mouse CD3e activated splenocytes for 4 days; Manufacturer<br>CD366 Tim3 APC antimouse; BALB/c anti-mouse CD3e activated splenocytes for 4 days; Manufacturer<br>CD366 Tim3 APC antimouse; Mouse Tim-3 transfected cells were stained with anti-mouse CD366 (Tim-3, clone RMT3-23) APC; Manufacturer<br>CD45R PerCPCy55antimouse; C57BL/6 mouse splenocytes; Manufacturer<br>CD45.2 PerCPCy55antimouse; C57BL/6 mouse splenocytes; Manufacturer<br>CD45.2 FITC antimouse; C57BL/6 mouse splenocytes; Manufacturer<br>CD223 LAG3 Brilliant Violet 421 antimouse; ConA-stimulated (3 days) C57BL/6 mouse splenocytes; Manufacturer<br>CD279 PD1 Alexa Fluor 647 antimouse; Con A and IL-2 stimulated C57BL/6 splenocytes (three days) were stained with CD3 FITC and CD279 (clone 29F.1A12) Alexa Fluor® 647; Manufacturer<br>CD274 B7H1, PDL1 APC antimouse; C57BL/6 mouse splenocytes; Manufacturer<br>Granzyme B Recombinant Antibody PE antimouse/antihuman; Human peripheral blood mononuclear cells were stained with CD8 Pacific Blue™, fixed and permeabilized, and then stained with Granzyme B PE; Manufacturer<br>Granzyme B Recombinant Antibody APC antmouse/antihuman Human; peripheral blood lymphocytes were surface stained with CD8 PE, then intracellularly stained with Granzyme B; Manufacturer<br>H2Kb FITC antimouse; C57BL/6 mouse splenocytes; Manufacturer<br>H2Kb bound to SIINFEKL PE antimouse; C57BL/6 mouse splenocytes were pulsed with or without SIINFEKL for 2 hours and then stained with 25-D1.16 PE; Manufacturer<br>H2Kb Antibody APC antimouse; C57BL/6 mouse splenocytes; Manufacturer<br>CD16/32 Antibody Purified antimouse; C57BL/6 mouse splenocytes; Manufacturer<br>TCR V51, 52 Antibody PE antimouse; C57BL/6 splenocytes stained with CD3e (145-2C11) APC and MR9-4 PE; Manufacturer<br>TCR V alpha 2 Monoclonal Antibody FITC antimouse; MOLT16 cells or negative control HPB-ALL cells FITC antimouse<br>CD8a LY 2 MicroBeads; Mouse splenocytes purity of pre and post CD8 beads treament was tested by flow cytometry; Manufacturer

DAPI   Manufacturer
ZOMBI Zombie UV™ Fixable Viability Kit; Manufacturer
InVivoMAb antimouse PD1 CD279 Zelenay, S., et al. (2015), Twyman-Saint Victor, C., et al. (2015); Manufacturer
InVivoMAb rat IgG2a isotype control, anti-trinitrophenol Zelenay, S., et al. (2015), Twyman-Saint Victor, C., et al. (2015); Manufacturer
InVivoMAb antimouse CTLA4 Gao, J., et al. (2016), Stephan, S. B., et al. (2015), Ozdemir, B. C., et al. (2014); Manufacturer
InVivoPlus mouse IgG2b isotype control Gao, J., et al. (2016), Stephan, S. B., et al. (2015), Ozdemir, B. C., et al. (2014); Manufacturer

Cytof: Each Ab lot was used at saturating concentrations that were empirically pre-determined by titrating 3-fold serial dilutions on cell suspensions from mouse tissues known to contain a mixture of cells positive and negative for each marker. The dilution showing the highest stain index ((Median metal intensity of Positives-MMI of negatives)/2xSD of negatives). Spleen or thymus were used to validate and titrate all Abs, with the exception of Epcam, gp38 and CD31 which were titrated on enzymatically digested mouse thymus or lung.

MICROSCOPY:
anti-CD3 Rabbit polyclonal to CD3; Immunohistochemical analysis of formalin-fixed, paraffin-embedded human tonsil tissue
anti-CD4 Rabbit monoclonal to CD4; Formaldehyde-fixed, non-permeabilized mouse colon tissue
mouse anti-CD8 Rabbit monoclonal to CD8; Immunohistochemical analysis of paraffin-embedded mouse spleen tissue mouse
anti-CD11b Rabbit monoclonal to CD11b; paraffin embedded Mouse lung tissue sections by Immunohistochemistry
mouse anti-PDL1 Rabbit monoclonal to PDL1; Immunohistochemical analysis of paraffin-embedded Renca syngeneic tumor using PD-L1
mouse anti-CD68 Rabbit polyclonal to CD68; Paraffin-embedded mouse spleen tissue stained for CD68
mouseMMR/CD206 polyclonal gait IgG; MMR/CD206 was detected in perfusion fixed frozen sections of mouse testis
SerpinB9 Polyclonal Antibody; Jiang L. et al, 2000
V5 Tag antibody Monoclonal; validated on 70% confluent V5-H3-6XHis transfected HEK-293 cells
WESTERN:
V5 Tag antibody Monoclonal; V5 untrasfected or transiently transfected HEK 293Tcells
Direct-BlotTM HRP anti-GAPDH Monoclonal; Whole cell lysates (15 µg protein) from HeLa and NIH3T3 cells

Eukaryotic cell lines

Policy information about cell lines and Sex and Gender in Research

Cell line source(s)

A549 (Male 58Y) and H125 (Male 61Y) cell lines were purchased from ATCC. LLC1 (LLC) cancer cell line was received as a gift from Dr. Hansen He (The Princess Margaret Cancer Centre). LLC Lewis lung carcinoma is a cell line established from the lung of a C57BL mouse bearing a tumor resulting from an implantation of primary Lewis lung carcinoma. The HEK 293T cells, used for LV production, were purchased from ATCC.

Authentication

The cell lines were obtained from ATCC collection and identification as well as authentication was performed by company.

Mycoplasma contamination

All cell lines were tested for mycoplasma and confirmed to be negative.

Commonly misidentified lines
(See ICLAC register)

No commonly misidentified cell lines were used in this study.

Animals and other research organisms

Policy information about studies involving animals; ARRIVE guidelines recommended for reporting animal research, and Sex and Gender in Research

Laboratory animals

The animals used in this study, LSL-KrasG12D (008179)103, LSL-BrafV600E (017837)104, R26-LSL-CAS9-GFP (026175), FVB.129S6(B6) Gt(ROSA)26Sortm1(Luc)Kael/J (005125), C57BL/6-Tg(TcraTcrb)1100Mjb/J (003831, also known as OT-I), Gt(ROSA)26Sortm4(ACTB-tdTomato,-EGFP)Luo/J (007576, also known as mT/mG), Gt(ROSA)26Sortm1(CAG-Brainbow2.1)Cle/J (013731, also known as R26R-Confetti), NOD.Cg-Prkdcscid IL2rgtm1Wjl/Sz/J (005557, also known as NSG), NU/J (002019, known as Nude) and C57BL/6J (000664) were purchased from the Jackson Laboratory. CRISPR screen in the LSL-KrasG12D-CAS9-LUC and LSL-BrafV600E-CAS9-LUC was

	performed in a F1 FVBNxC57BL/6J background. Genotyping was performed by PCR using genomic DNA prepared from mouse ear punches. All details of the used animal models are listed in Table S8<br>The age of animals used include: P2 neonates for intranasal instillation; 3-4 weeks for adoptive T cell transfer (ACT); 8-12 weeks for tumor transplant and/or ACT. For all experimental groups, mice were age matched.
Wild animals	There was no wild animals used in this study.
Reporting on sex	Equal numbers of male and female animals were used throughout the study without any bias.
Field-collected samples	Samples were not collected from the field
Ethics oversight	Animal husbandry, ethical handling of mice and all animal work were carried out according to guidelines approved by Canadian Council of Animal Care and under protocols approved by the Centre for Phenogenomics Animal Care Committee (18-0272H).

Note that full information on the approval of the study protocol must also be provided in the manuscript.

Flow Cytometry

Plots

Confirm that:

☐ The axis labels state the marker and fluorochrome used (e.g. CD4-FITC).

☒ The axis scales are clearly visible. Include numbers along axes only for bottom left plot of group (a 'group' is an analysis of identical markers).

☐ All plots are contour plots with outliers or pseudocolor plots.

☒ A numerical value for number of cells or percentage (with statistics) is provided.

Methodology

Sample preparation	TISSUE PREPARATION:Single cell suspensions from spleen, LNs, lung or implanted tumors were washed with FACS buffer (DPBS + 1%BSA+ 1mM EDTA + 0.1% sodium azide), incubated with FC block (CD16/32) for 30' at 4°C, stained with appropriate antibodies and washed twice in FACS buffer. Dead cells were excluded from all data by forward and side scatter and 4',6-diamidino-2-phenylindole, dihydrochloride (DAPI, Molecular Probes - 5mg/ml, used 1/50,000) or fixable viability dye eFluorTM 450 (1/1000, Invitrogen). Cells were stained by standard staining techniques and analyzed on Fortessa flow cytometer (BD Biosciences). For intracellular staining, the cells were permeabilized using eBioscienceTM Foxp3/Transcription factor Staining Buffer Set (00-5523-00,Thermo Fisher Scientific) according to manufacturer's recommendation. Data Files were analyzed using Flow-Jo (Tree Star).<br><br>TUMOR PREPARATION: Tumor tissues (fresh or frozen) were minced into small pieces using surgical blade and scalpel (08-957-5D, Fisher Scientific) and processed using the tumor dissociation kit (130-096-730, Miltenyi Biotec) as recommended by supplier. Single-cell suspensions from lung or implanted tumors were obtained using kit and the gentleMACS Octo Dissociator. Dissociated cells were passaged through 70µm cell strainer (BD), collected in a 50ml falcon tube, and resuspended in staining buffer (1%BSA in PBS).
Instrument	BD LSRFortessa Cell Analyzer, Hellios Mass Cytometry (CyTOF), Beckman Coulter MoFlo Astrios EQ Cell sorter
Software	The FACS data was analyzed using FlowJo software.
Cell population abundance	Cell abundance ranged from 10% and above. After cell sorting the purity check was performed.
Gating strategy	Dead cells were excluded from all data analysis by following gating strategy: viable dye (DAPI, PI, or fixable viability dye eFluorTM 450-Zombi dye); FSCA/SSCA; FSCA/FSCH; SSCW/SSCH, followed by cell-specific antibodies.

☒ Tick this box to confirm that a figure exemplifying the gating strategy is provided in the Supplementary Information.
