# Supplementary material for: In vivo CRISPR screens reveal Serpinb9 and Adam2 as regulators of immune therapy response in lung cancer
Source: Nat Commun. 2023 May 31;14:3150. doi: 10.1038/s41467-023-38841-7 (PMC10232477; doi:10.1038/s41467-023-38841-7)
Supplement: Supplementary file 13 — Source Data [file 41467_2023_38841_MOESM13_ESM.zip › Supplementary Figure 30-37.zip!/Differential expression and pathway enrichment analysis of TCGA LUAD samples with ADAM2 expressiont/Volcano_plot.pdf]

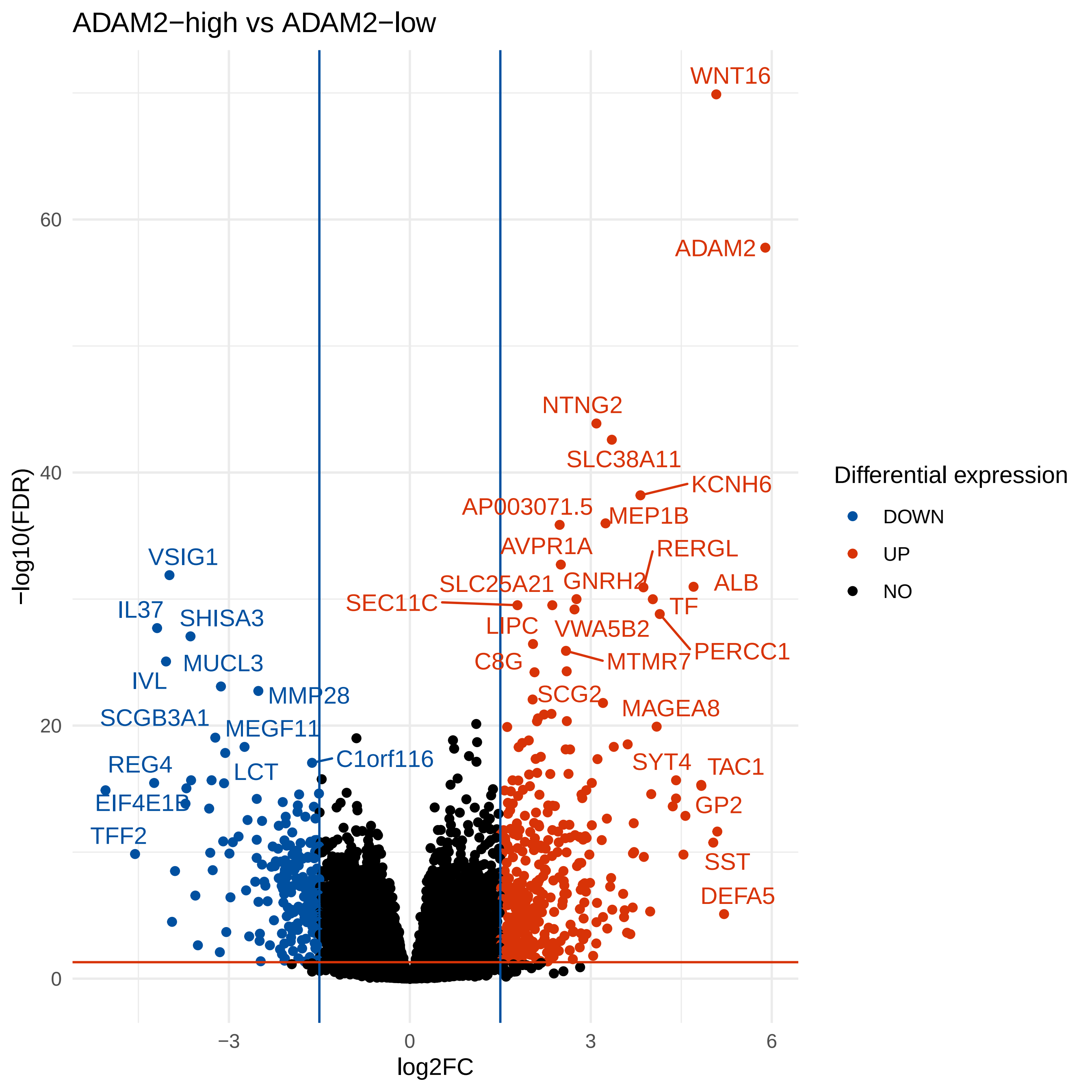
ADAM2−high vs ADAM2−low
−log10(FDR)
log2FC
60
40
20
0
−3
0
3
6
WNT16
ADAM2
NTNG2
SLC38A11
KCNH6
AP003071.5
MEP1B
AVPR1A
RERGL
VSIG1
SLC25A21
GNRH2
ALB
SEC11C
TF
IL37
SHISA3
LIPC
VWA5B2
PERCC1
MUCL3
C8G
MTMR7
IVL
MMP28
SCG2
MAGEA8
SCGB3A1
MEGF11
C1orf116
SYT4
TAC1
REG4
LCT
EIF4E1B
GP2
TFF2
SST
DEFA5
Differential expression
DOWN
UP
NO
