# Supplementary material for: In vivo CRISPR screens reveal Serpinb9 and Adam2 as regulators of immune therapy response in lung cancer
Source: Nat Commun. 2023 May 31;14:3150. doi: 10.1038/s41467-023-38841-7 (PMC10232477; doi:10.1038/s41467-023-38841-7)

Pathways enriched for differentially expressed genes in ADAM2−high samples vs ADAM2−low samples

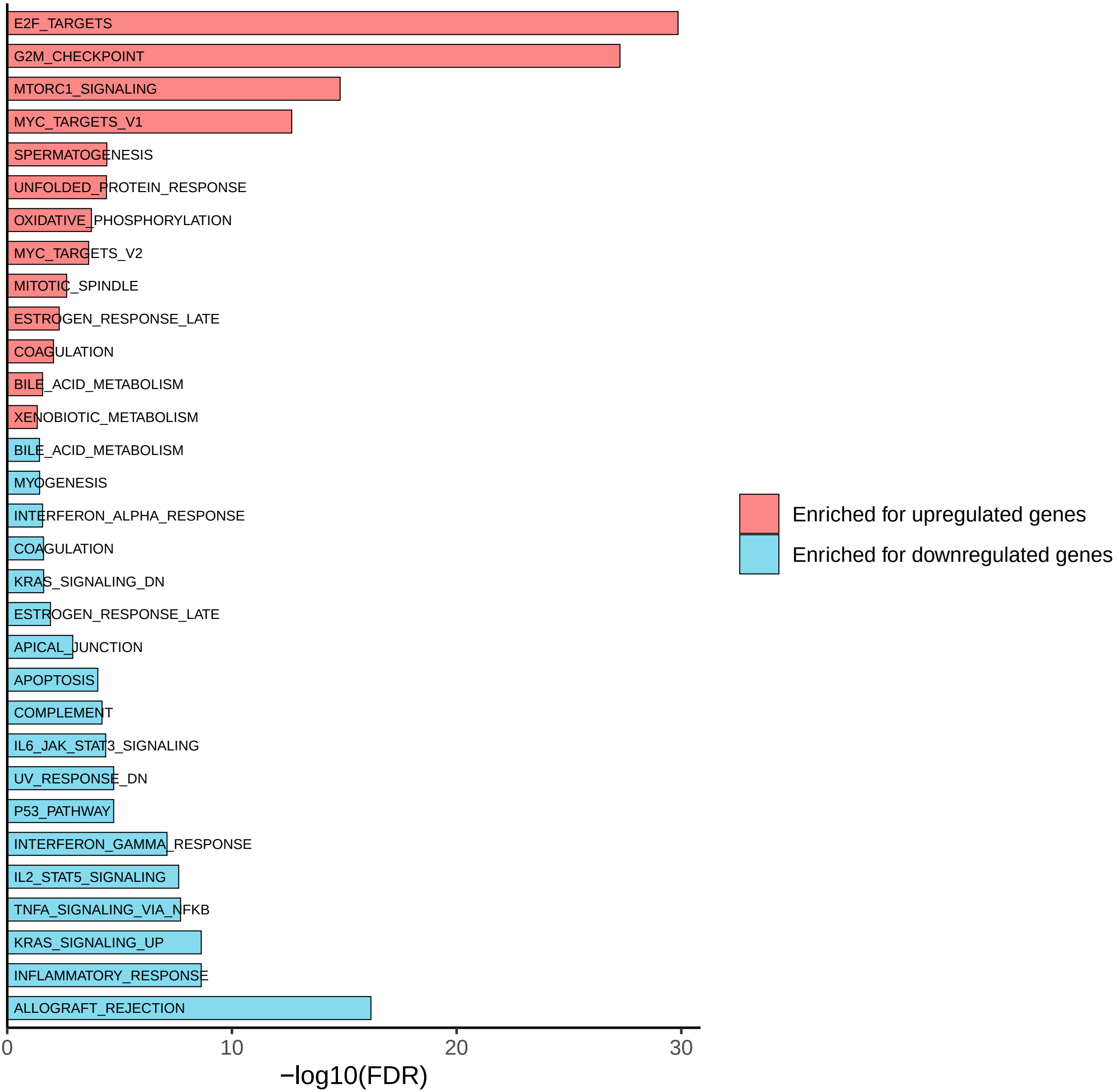

Supplement: Supplementary file 13 — Source Data [file 41467_2023_38841_MOESM13_ESM.zip › Supplementary Figure 30-37.zip!/Differential expression and pathway enrichment analysis of TCGA LUAD samples with ADAM2 expressiont/Pathway_signifiance_barplot.pdf]
