# Supplementary material for: In vivo CRISPR screens reveal Serpinb9 and Adam2 as regulators of immune therapy response in lung cancer
Source: Nat Commun. 2023 May 31;14:3150. doi: 10.1038/s41467-023-38841-7 (PMC10232477; doi:10.1038/s41467-023-38841-7)
Supplement: Supplementary file 13 — Source Data [file 41467_2023_38841_MOESM13_ESM.zip › Supplementary Figure 30-37.zip!/Differential expression and pathway enrichment analysis of TCGA LUAD samples with ADAM2 expressiont/ADAM2_methods_figure_descriptions.docx]

Methods:

**Differential expression and pathway enrichment analysis of TCGA LUAD samples with ADAM2 expression**

To enable the analysis, LUAD tumor samples from 510 LUAD patients were first split into two groups based on their expression of the *ADAM2* gene using consensus normalised RNA-seq data of the TCGA PanCanAtlas project (unc.edu_PANCAN_IlluminaHiSeq_RNASeqV2.geneExp_whitelisted.tsv). LUAD samples were assigned to the ADAM2-high group (FPKM-UQ >= 1) or the ADAM2-low group (FPKM-UQ<1)~~.~~ Unnormalized gene expression counts (STAR) for the TCGA LUAD cohort were then downloaded using the TCGAbiolinks R package^1^ (v. 2.18.0). Differential gene expression analysis was performed on the unnormalized STAR counts by comparing the two sample groups using the DESeq2 R package^2^ (v. 1.30.1). The analysis focused on protein coding genes and filtered non-coding RNA genes. Two separate pathway enrichment analyses were conducted for genes that were upregulated and downregulated in the ADAM2-high group, respectively, using the ActivePathways R package^3,4^(v. 1.1.1). Gene sets representing the 50 cancer hallmark pathways from the MSigDB database^5^ were used in the enrichment analysis. A gene significance value cutoff of 0.05 and gene sets of 50 to 1000 genes were used as the parameters for ActivePathways. Significantly enriched pathways were highlighted (FDR < 0.05). Genes in each significant pathway were visualised with respect to log2-transformed fold-change (FC) and significance (FDR) in ADAM2-high vs ADAM2-low LUAD samples.

Volcano plot:

Volcano plot showing differentially expressed genes between ADAM2-low and ADAM2-high tumours in the TCGA LUAD cohort. Genes with FDR<0.05 and log2FC < 1.5 are highlighted in blue and genes with FDR<0.05 and log2FC > 1.5 are highlighted in red.

Pathway results:

Bar plots showing the pathways that were significantly enriched for upregulated and downregulated genes in the TCGA LUAD cohort (FDR < 0.05). Pathways enriched for upregulated genes are shown in red and pathways enriched for downregulated genes are shown in blue. -log10 transformed FDR-adjusted p-values are shown on the x-axis.

Pathway contributing gene plots:

For each pathway, all the genes that contributed to its enrichment are displayed. The FDR-adjusted p-value (-log10) for its differential expression is shown on the y-axis and the log2 transformed absolute value of its fold change is represented by the size of the circle. The absolute value of the log2 fold change was capped at 3 for visualization.

1	Colaprico, A. *et al.* TCGAbiolinks: an R/Bioconductor package for integrative analysis of TCGA data. *Nucleic Acids Res* **44**, e71, doi:10.1093/nar/gkv1507 (2016).

2	Love, M. I., Huber, W. & Anders, S. Moderated estimation of fold change and dispersion for RNA-seq data with DESeq2. *Genome Biol* **15**, 550, doi:10.1186/s13059-014-0550-8 (2014).

3	Paczkowska, M. *et al.* Integrative pathway enrichment analysis of multivariate omics data. *Nat Commun* **11**, 735, doi:10.1038/s41467-019-13983-9 (2020).

4	Paczkowska, M. *et al.* Author Correction: Integrative pathway enrichment analysis of multivariate omics data. *Nat Commun* **13**, 7570, doi:10.1038/s41467-022-32342-9 (2022).

5	Liberzon, A. *et al.* The Molecular Signatures Database (MSigDB) hallmark gene set collection. *Cell Syst* **1**, 417-425, doi:10.1016/j.cels.2015.12.004 (2015).
