# Supplementary material for: In vivo CRISPR screens reveal Serpinb9 and Adam2 as regulators of immune therapy response in lung cancer
Source: Nat Commun. 2023 May 31;14:3150. doi: 10.1038/s41467-023-38841-7 (PMC10232477; doi:10.1038/s41467-023-38841-7)
Supplement: Supplementary file 13 — Source Data [file 41467_2023_38841_MOESM13_ESM.zip › Supplementary Figure 30-37.zip!/Differential expression and pathway enrichment analysis of TCGA LUAD samples with ADAM2 expressiont/Pathway_DE_genes_significance_log2FC.pdf]

E2F_TARGETS
Enriched for upregulated genes, FDR = 1.4e−30, n genes = 123

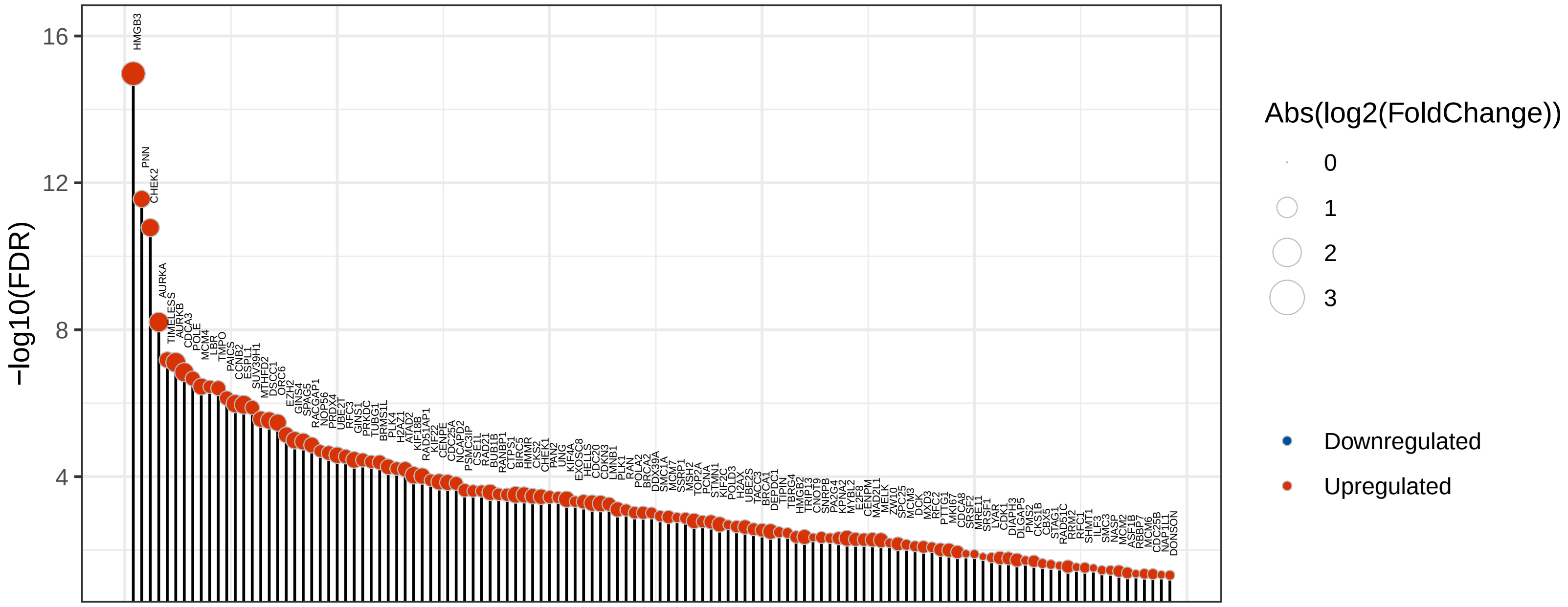

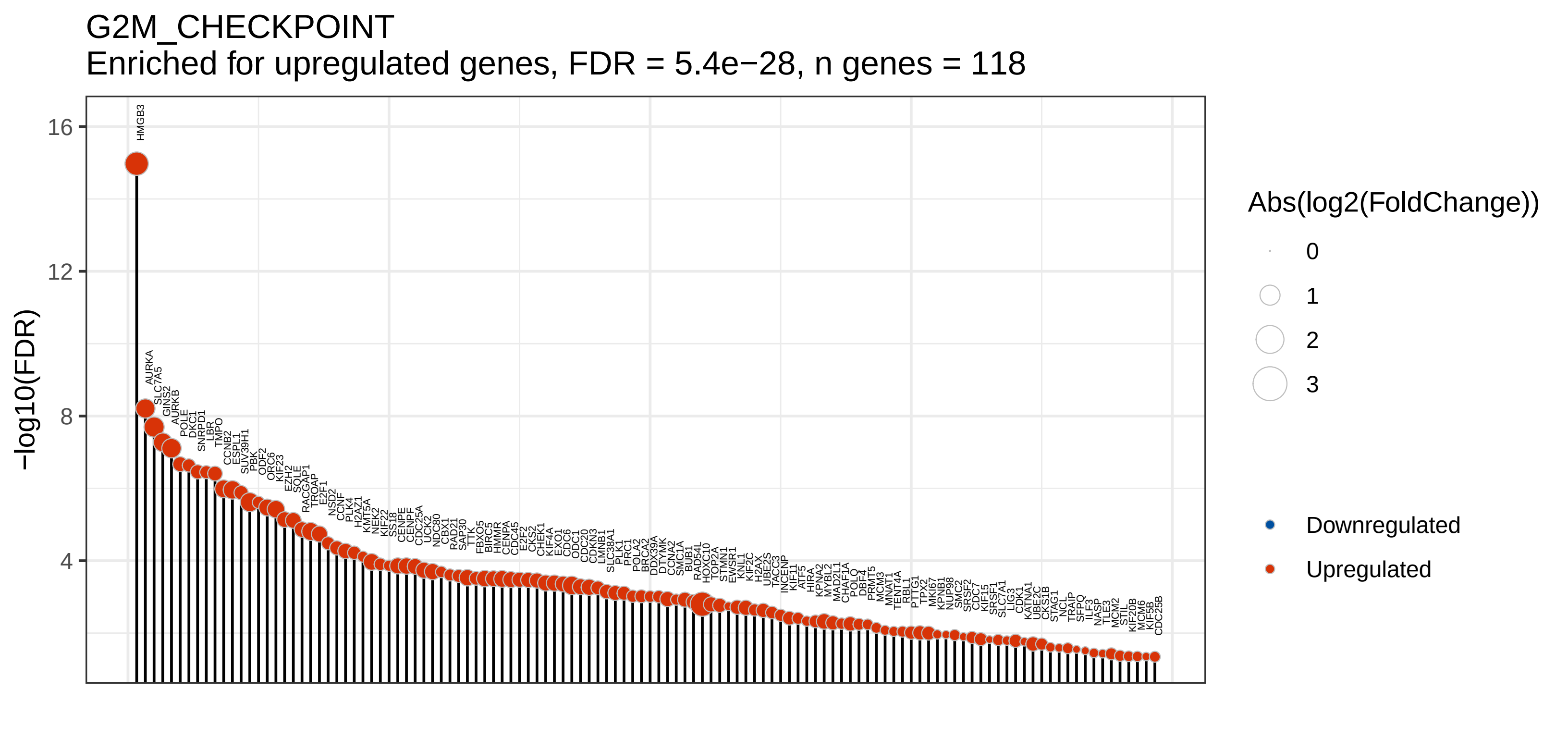
G2M_CHECKPOINT
Enriched for upregulated genes, FDR = 5.4e−28, n genes = 118
-log10(FDR)
16
12
8
4
HMGB3
AURKA
SLC7A5
GINS2
AURKB
POLE
DKC1
SNRPD1
LBR
TMPO
CCNB2
ESPL1
SUV39H1
PBK
ODF2
ORC6
KIF23
EZH2
SQLE
RACGAP1
TROAP
E2F1
NSD2
CCNF
PLK4
H2AZ1
KMT5A
NEK2
KIF22
SS18
CENPE
CENPF
CDC25A
UCK2
NDC80
CBX1
RAD21
SAP30
TTK
FBXO5
BIRC5
HMMR
CENPA
CDC45
E2F2
CKS2
CHEK1
KIF4A
EXO1
CDC6
ODC1
CDC20
CDKN3
LMNB1
SLC38A1
PLK1
PRC1
POLA2
BRCA2
DDX39A
DTYMK
CCNA2
SMC1A
BUB1
RAD54L
HOXC10
TOP2A
STMN1
EWSR1
KNL1
KIF2C
H2AX
UBE2S
TACC3
INCENP
KIF11
ATF5
HIRA
KPNA2
MYBL2
MAD2L1
CHAF1A
POLQ
DBF4
PRMT5
MCM3
MNAT1
TENT4A
RBL1
PTTG1
TPX2
MKI67
KPNB1
NUP98
SMC2
SRSF2
CDC7
KIF15
SRSF1
SLC7A1
LIG3
CDK1
KATNA1
UBE2C
CKS1B
STAG1
NCL
TRAIP
SFPQ
ILF3
NASP
TLE3
MCM2
STIL
KIF20B
MCM6
KIF5B
CDC25B
Abs(log2(FoldChange))
0
1
2
3
Downregulated
Upregulated

MTORC1_SIGNALING
Enriched for upregulated genes, FDR = 1.5e−15, n genes = 105

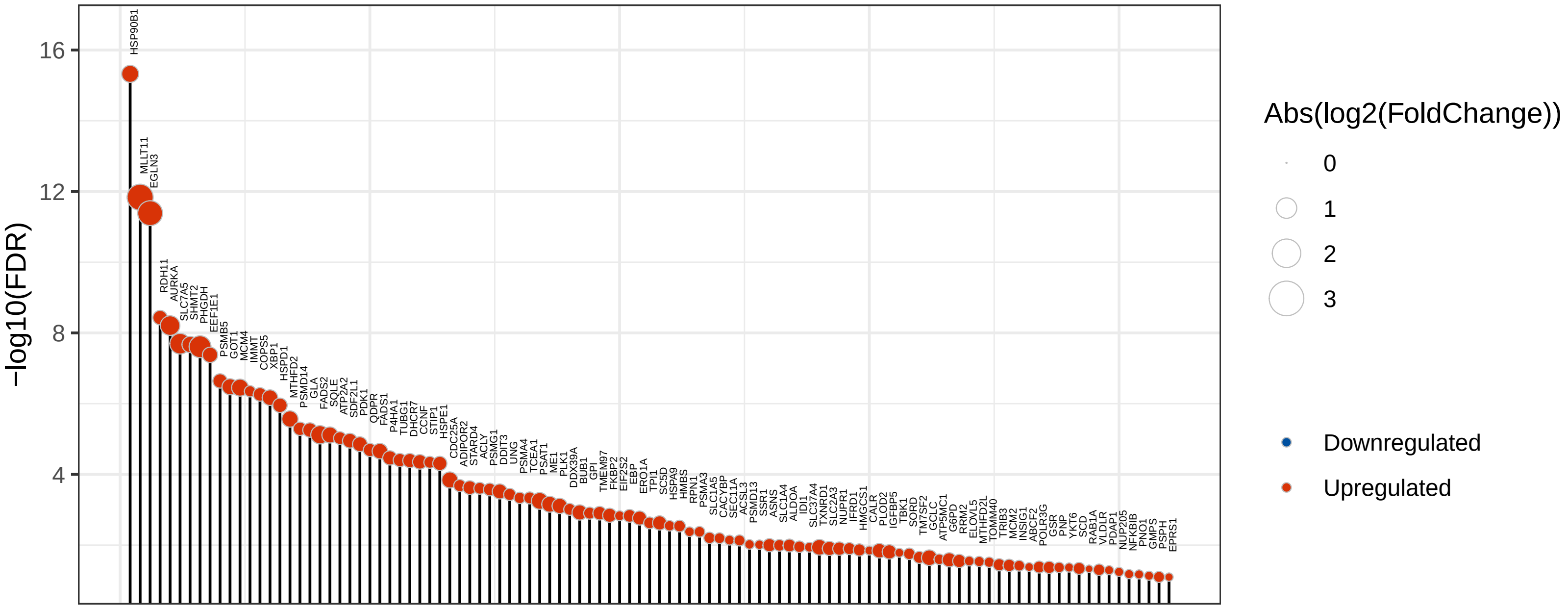

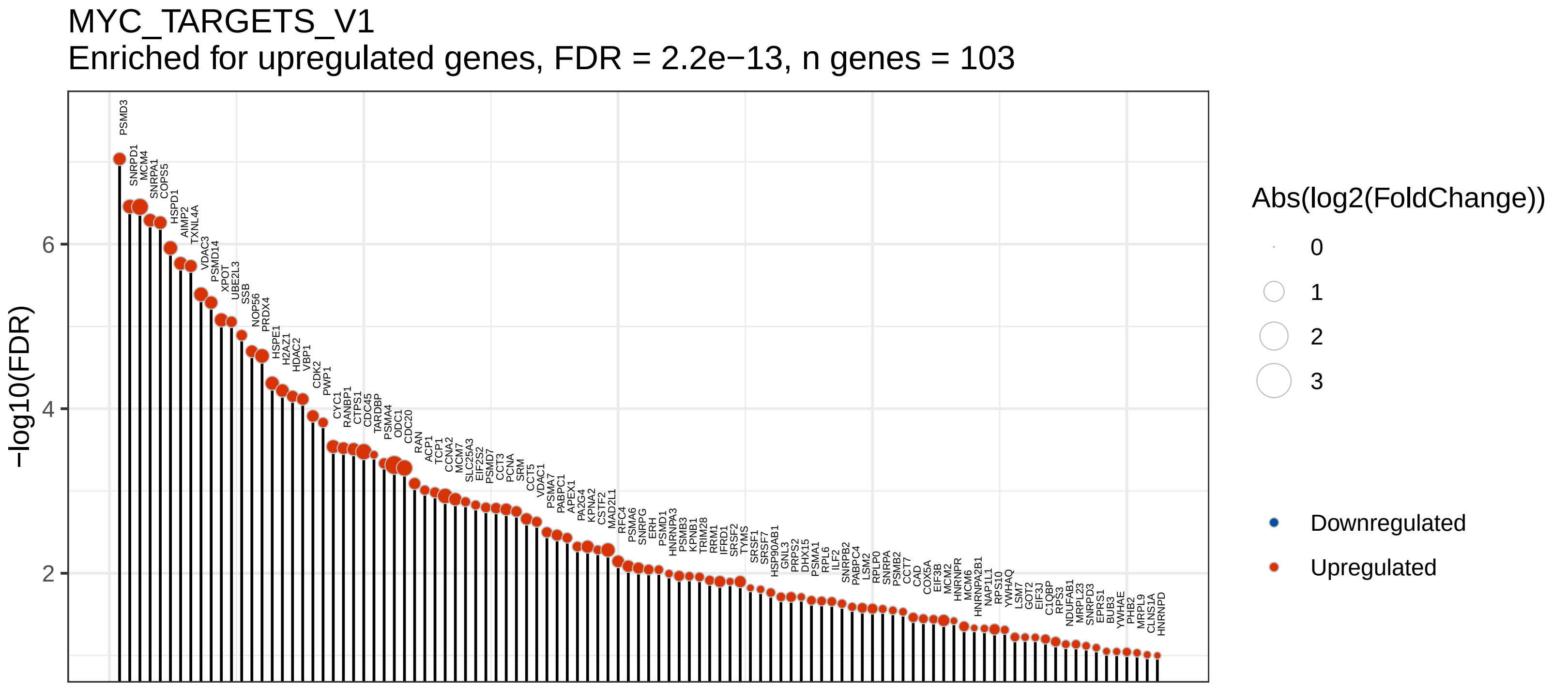
MYC_TARGETS_V1
Enriched for upregulated genes, FDR = 2.2e−13, n genes = 103
−log10(FDR)
2
4
6
PSMD3
SNRPD1
MCM4
SNRPA1
COPS5
HSPD1
AIMP2
TXNL4A
VDAC3
PSMD14
XPOT
UBE2L3
SSB
NOP56
PRDX4
HSPE1
H2AZ1
HDAC2
VBP1
CDK2
PWP1
CYC1
RANBP1
CTPS1
CDC45
TARDBP
PSMA4
ODC1
CDC20
RAN
ACP1
TCP1
CCNA2
MCM7
SLC25A3
EIF2S2
PSMD7
CCT3
PCNA
SRM
CCT5
VDAC1
PSMA7
PABPC1
APEX1
PA2G4
KPNA2
CSTF2
MAD2L1
RFC4
PSMA6
SNRPG
ERH
PSMD1
HNRNPA3
PSMB3
KPNB1
TRIM28
RRM1
IFRD1
SRSF2
TYMS
SRSF1
SRSF7
HSP90AB1
GNL3
PRPS2
DHX15
PSMA1
RPL6
ILF2
SNRPB2
PABPC4
LSM2
RPLP0
SNRPA
PSMB2
CCT7
CAD
COX5A
EIF3B
MCM2
HNRNPR
MCM6
HNRNPA2B1
NAP1L1
RPS10
YWHAQ
LSM7
GOT2
EIF3J
C1QBP
RPS3
NDUFAB1
MRPL23
SNRPD3
EPRS1
BUB3
YWHAE
PHB2
MRPL9
CLNS1A
HNRNPD
Abs(log2(FoldChange))
0
1
2
3
Downregulated
Upregulated

SPERMATOGENESIS
Enriched for upregulated genes, FDR = 3.7e−05, n genes = 49

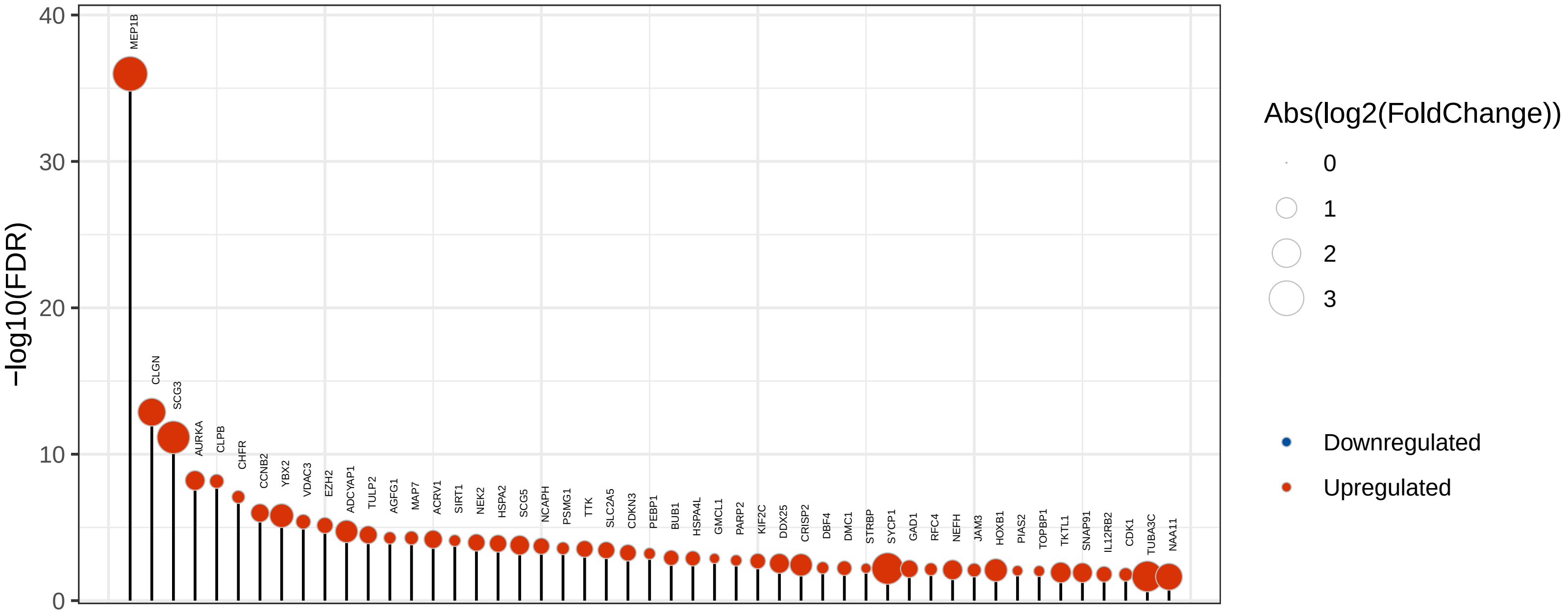

UNFOLDED_PROTEIN_RESPONSE
Enriched for upregulated genes, FDR = 3.8e−05, n genes = 48

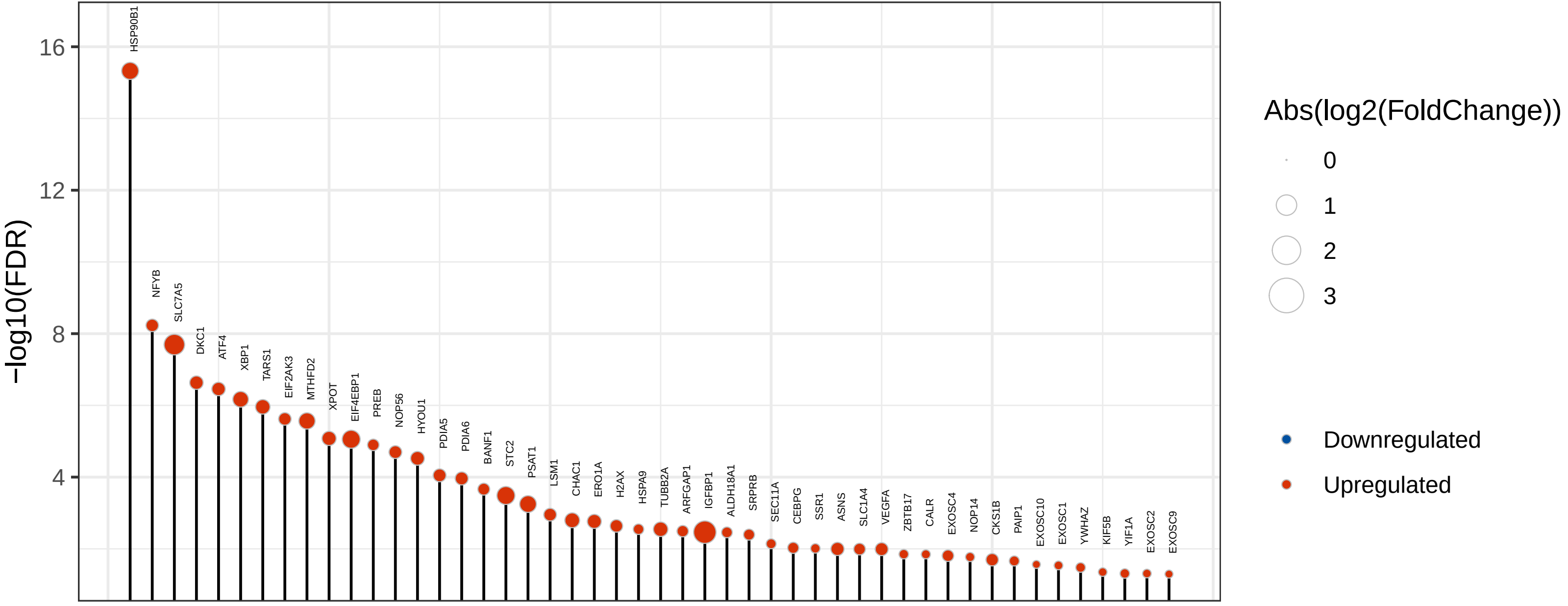

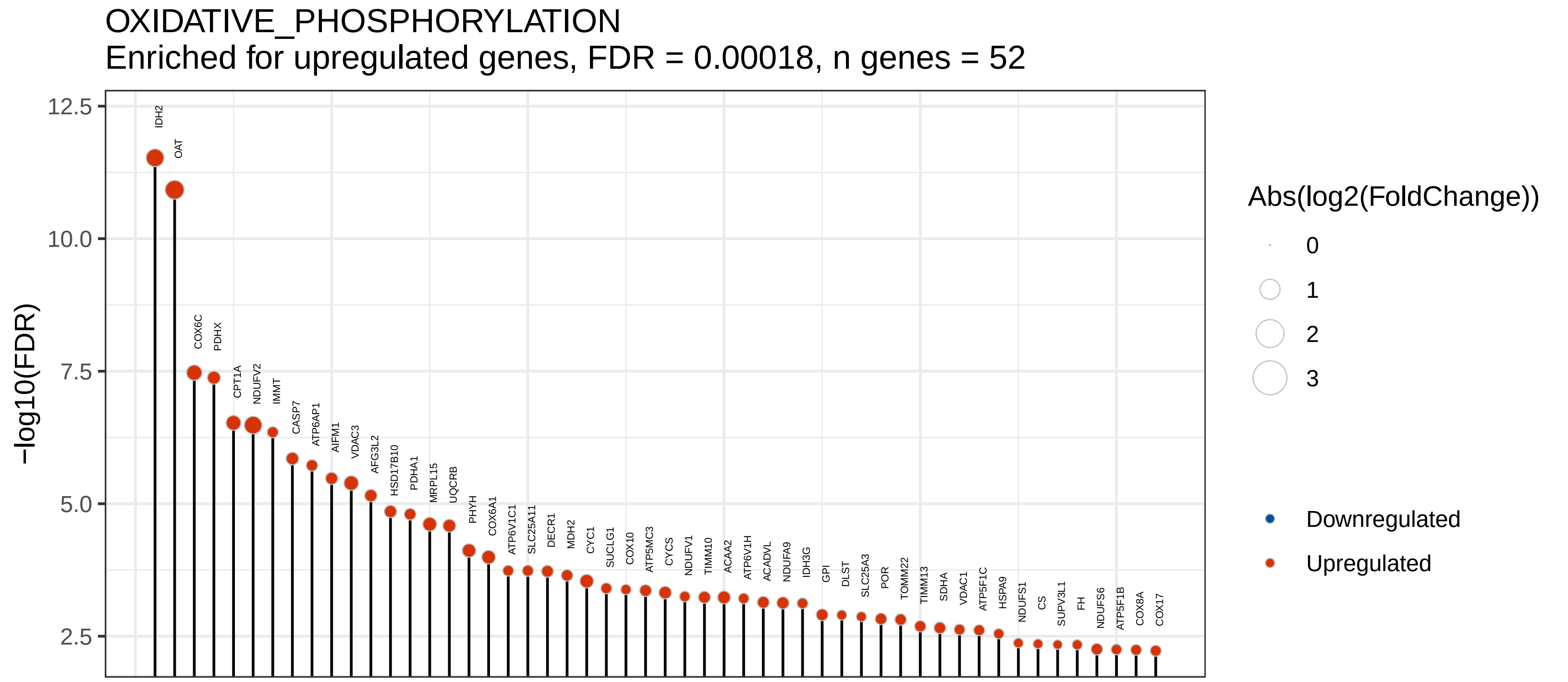
OXIDATIVE_PHOSPHORYLATION
Enriched for upregulated genes, FDR = 0.00018, n genes = 52
-log10(FDR)
12.5
10.0
7.5
5.0
2.5
IDH2
OAT
COX6C
PDHX
CPT1A
NDUFV2
IMMT
CASP7
ATP6AP1
AIFM1
VDAC3
AFG3L2
HSD17B10
PDHA1
MRPL15
UQCRB
PHYH
COX6A1
ATP6V1C1
SLC25A11
DECR1
MDH2
CYC1
SUCLG1
COX10
ATP5MC3
CYCS
NDUFV1
TIMM10
ACAA2
ATP6V1H
ACADVL
NDUFA9
IDH3G
GPI
DLST
SLC25A3
POR
TOMM22
TIMM13
SDHA
VDAC1
ATP5F1C
HSPA9
NDUFS1
CS
SUPV3L1
FH
NDUFS6
ATP5F1B
COX8A
COX17
Abs(log2(FoldChange))
0
1
2
3
Downregulated
Upregulated

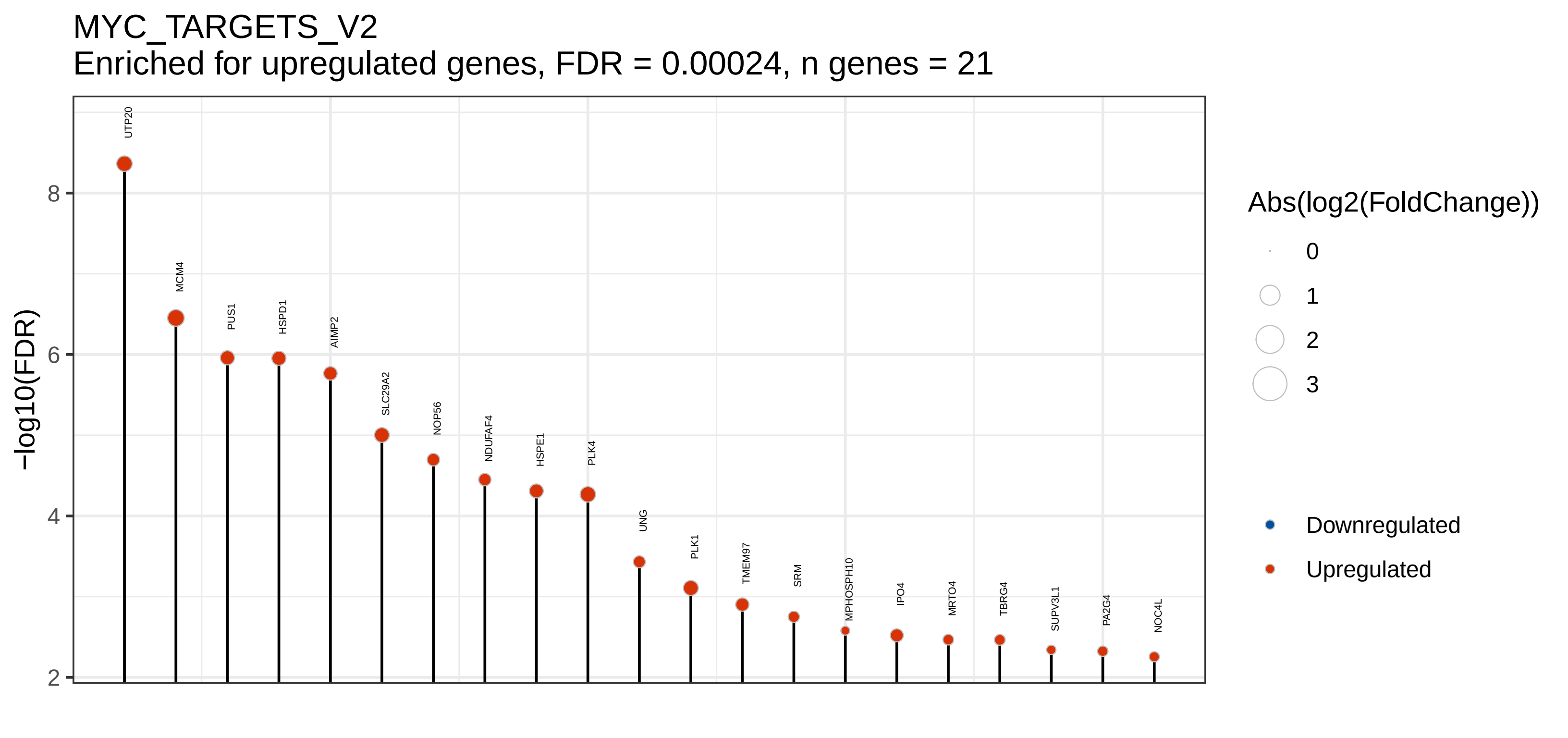
MYC_TARGETS_V2
Enriched for upregulated genes, FDR = 0.00024, n genes = 21
-log10(FDR)
8
6
4
2
UTP20
MCM4
PUS1
HSPD1
AIMP2
SLC29A2
NOP56
NDUFAF4
HSPE1
PLK4
UNG
PLK1
TMEM97
SRM
MPHOSPH10
IPO4
MRTO4
TBRG4
SUPV3L1
PA2G4
NOC4L
Abs(log2(FoldChange))
0
1
2
3
Downregulated
Upregulated

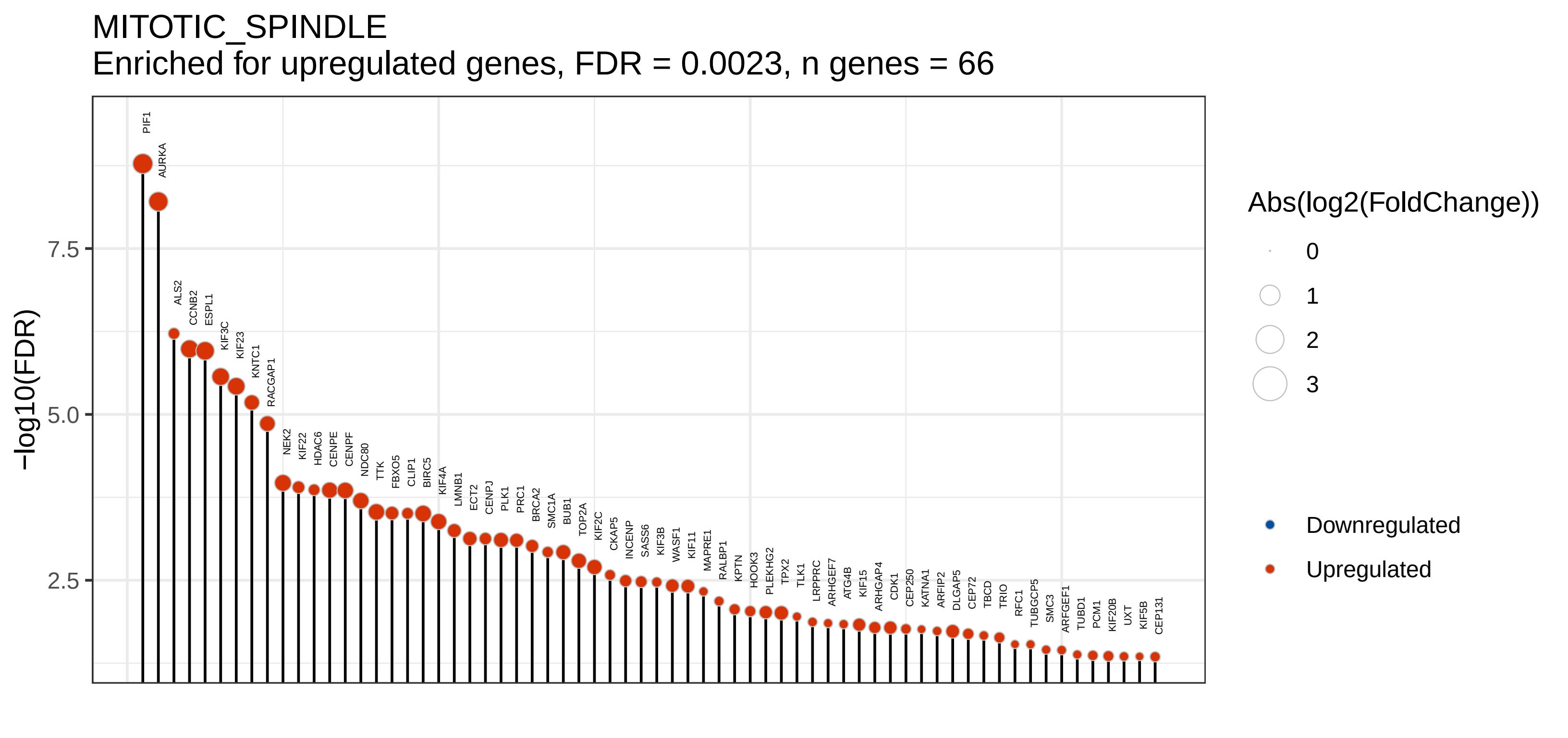
MITOTIC_SPINDLE
Enriched for upregulated genes, FDR = 0.0023, n genes = 66
-log10(FDR)
7.5
5.0
2.5
PIF1
AURKA
ALS2
CCNB2
ESPL1
KIF3C
KIF23
KNTC1
RACGAP1
NEK2
KIF22
HDAC6
CENPE
CENPF
NDC80
TTK
FBXO5
CLIP1
BIRC5
KIF4A
LMNB1
ECT2
CENPJ
PLK1
PRC1
BRCA2
SMC1A
BUB1
TOP2A
KIF2C
CKAP5
INCENP
SASS6
KIF3B
WASF1
KIF11
MAPRE1
RALBP1
KPTN
HOOK3
PLEKHG2
TPX2
TLK1
LRPPRC
ARHGEF7
ATG4B
KIF15
ARHGAP4
CDK1
CEP250
KATNA1
ARFIP2
DLGAP5
CEP72
TBCD
TRIO
RFC1
TUBGCP5
SMC3
ARFGEF1
TUBD1
PCM1
KIF20B
UXT
KIF5B
CEP131
Abs(log2(FoldChange))
0
1
2
3
Downregulated
Upregulated

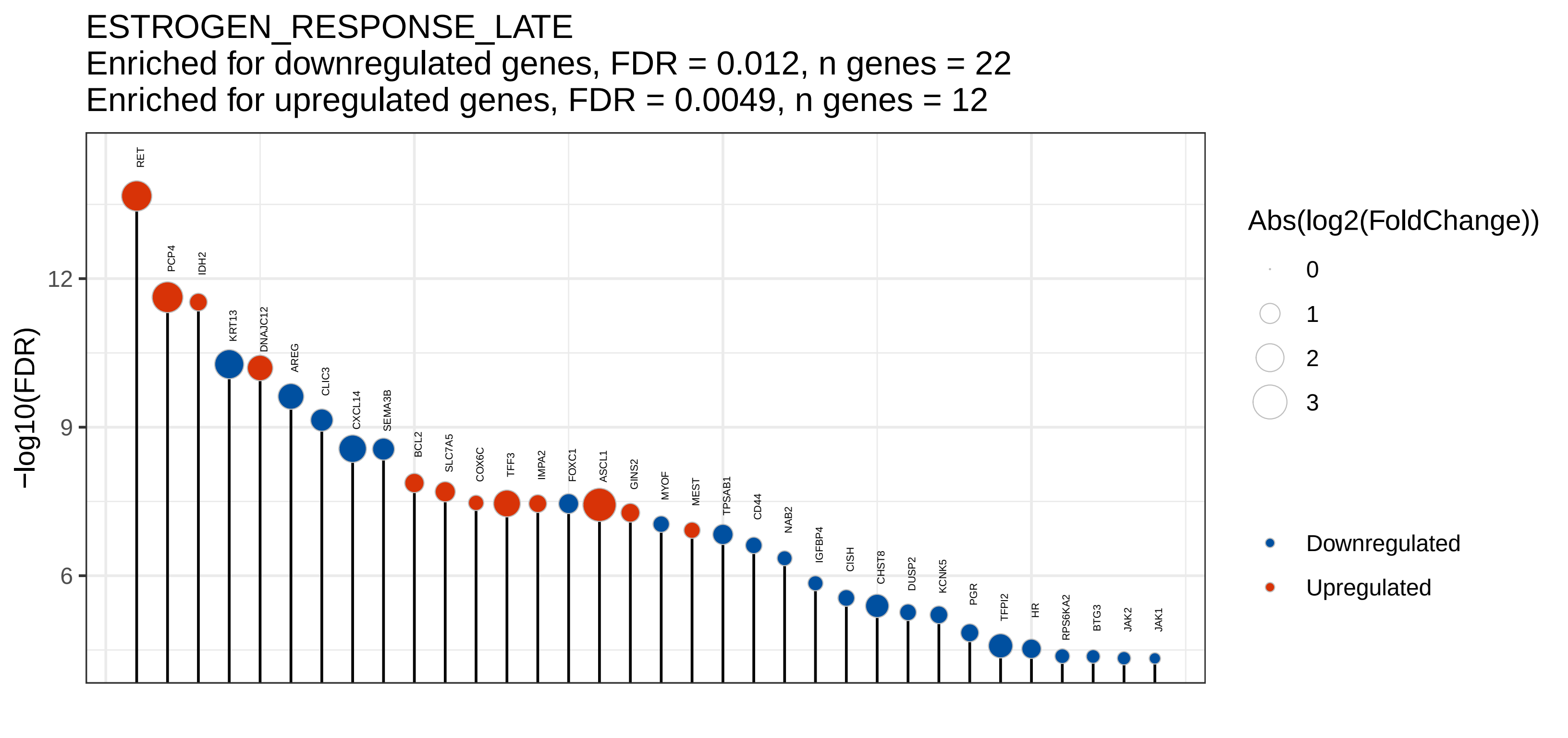

ESTROGEN_RESPONSE_LATE
Enriched for downregulated genes, FDR = 0.012, n genes = 22
Enriched for upregulated genes, FDR = 0.0049, n genes = 12
−log10(FDR)
12
9
6
RET
PCP4
IDH2
KRT13
DNAJC12
AREG
CLIC3
CXCL14
SEMA3B
BCL2
SLC7A5
COX6C
TFF3
IMPA2
FOXC1
ASCL1
GINS2
MYOF
MEST
TPSAB1
CD44
NAB2
IGFBP4
CISH
CHST8
DUSP2
KCNK5
PGR
TFPI2
HR
RPS6KA2
BTG3
JAK2
JAK1
Abs(log2(FoldChange))
0
1
2
3
Downregulated
Upregulated

COAGULATION
Enriched for downregulated genes, FDR = 0.024, n genes = 15
Enriched for upregulated genes, FDR = 0.0087, n genes = 3

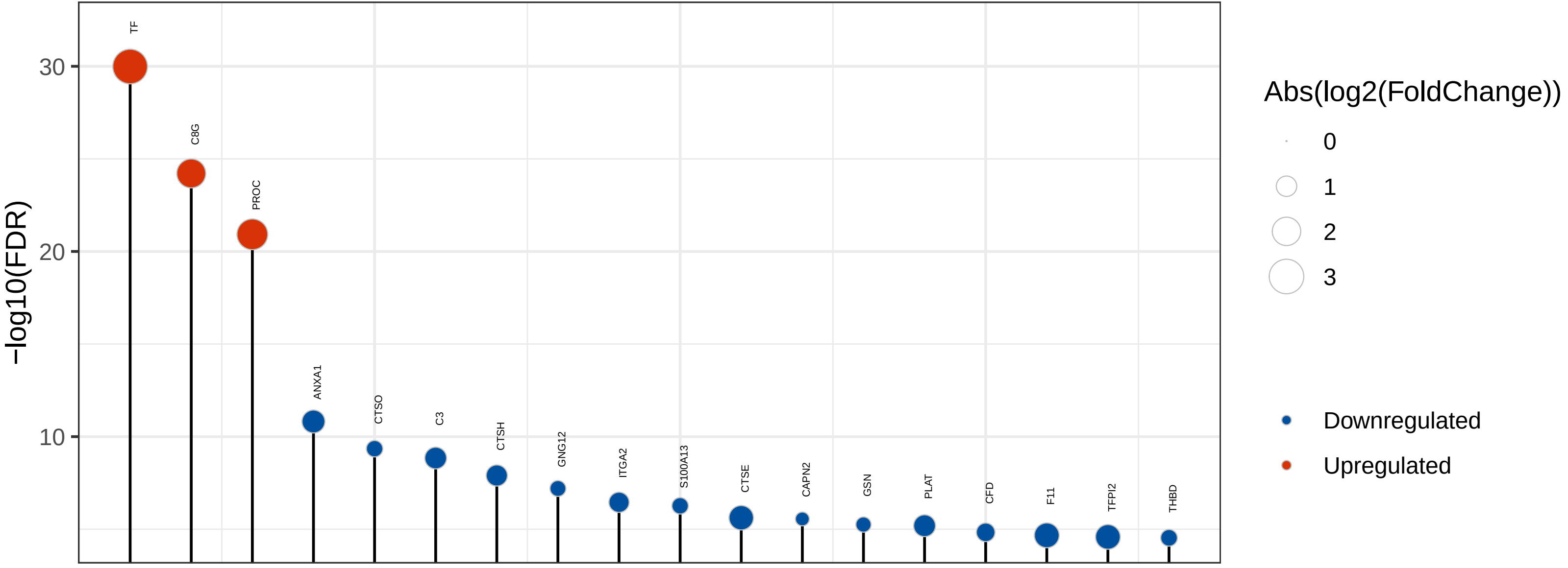

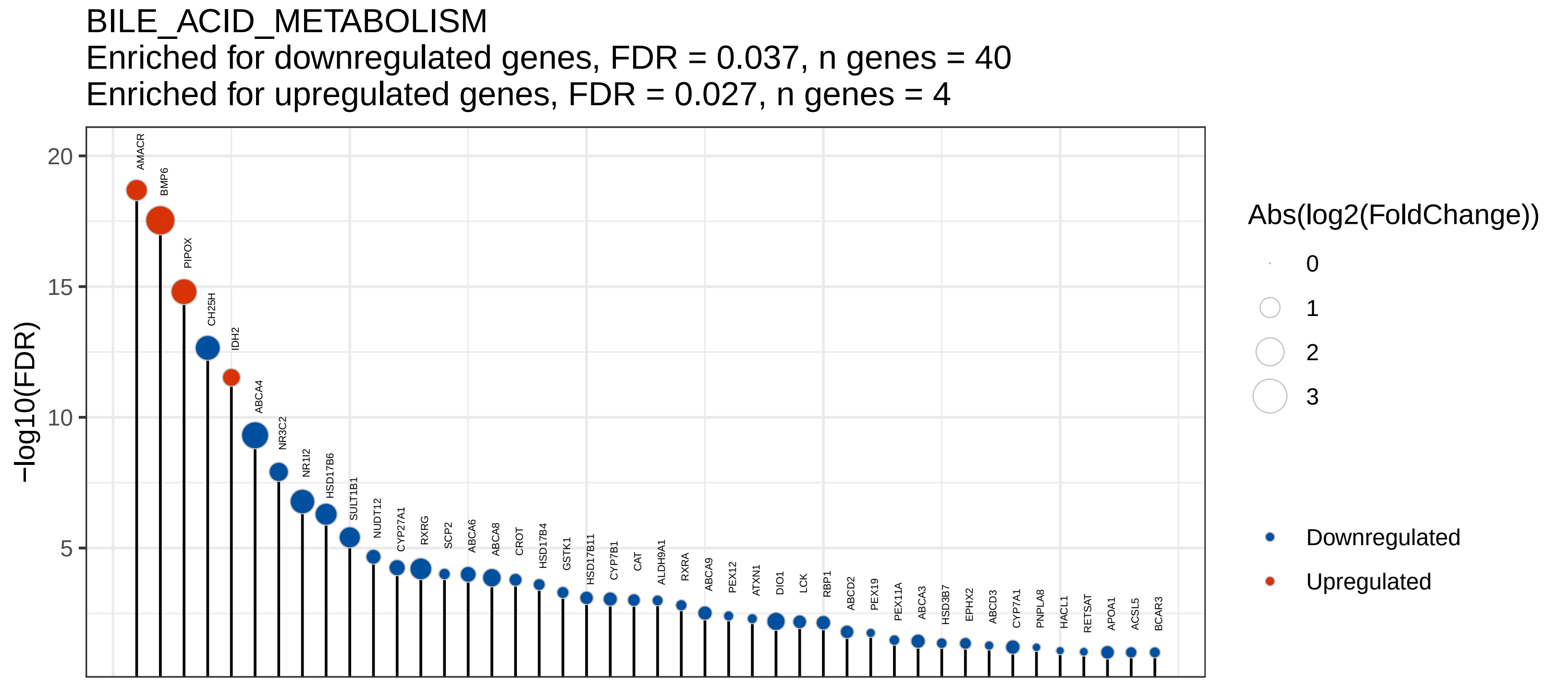
BILE_ACID_METABOLISM
Enriched for downregulated genes, FDR = 0.037, n genes = 40
Enriched for upregulated genes, FDR = 0.027, n genes = 4
-log10(FDR)
20
15
10
5
AMACR
BMP6
PIPOX
CH25H
IDH2
ABCA4
NR3C2
NR1I2
HSD17B6
SULT1B1
NUDT12
CYP27A1
RXRG
SCP2
ABCA6
ABCA8
CROT
HSD17B4
GSTK1
HSD17B11
CYP7B1
CAT
ALDH9A1
RXRA
ABCA9
PEX12
ATXN1
DIO1
LCK
RBP1
ABCD2
PEX19
PEX11A
ABCA3
HSD3B7
EPHX2
ABCD3
CYP7A1
PNPLA8
HACL1
RETSAT
APOA1
ACSL5
BCAR3
Abs(log2(FoldChange))
0
1
2
3
Downregulated
Upregulated

XENOBIOTIC_METABOLISM
Enriched for upregulated genes, FDR = 0.046, n genes = 19

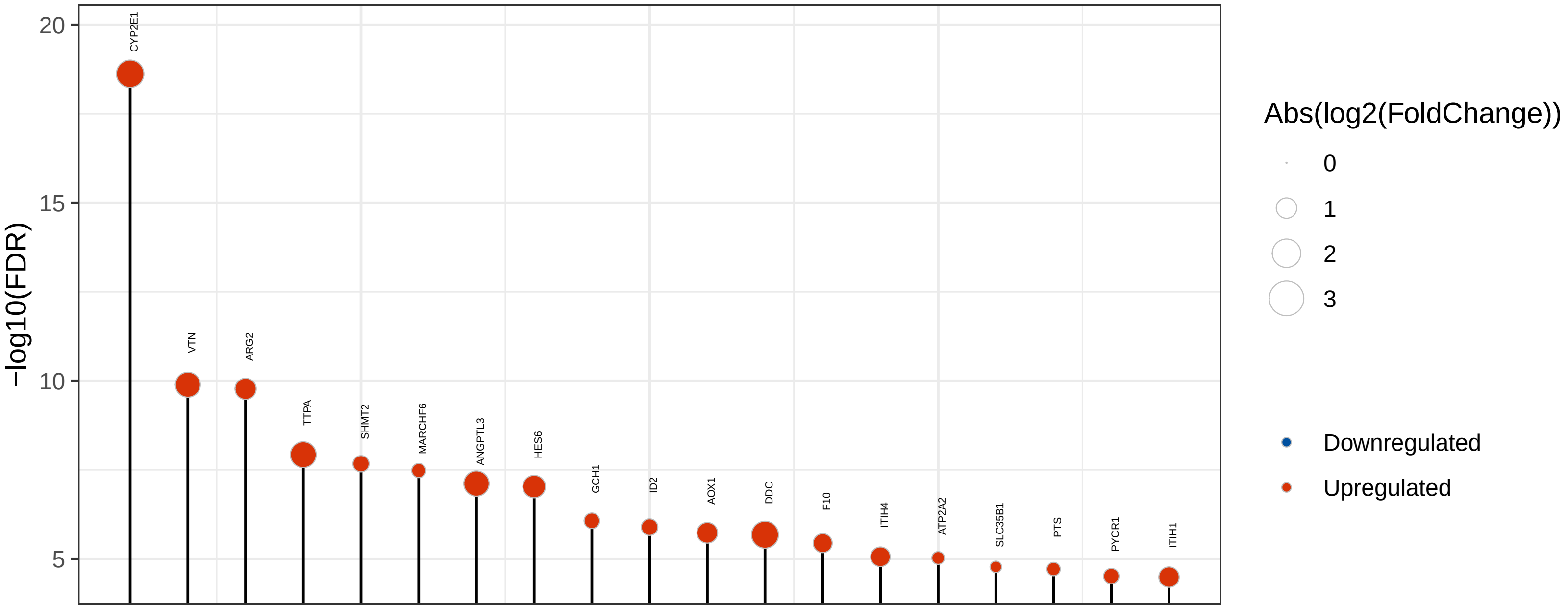

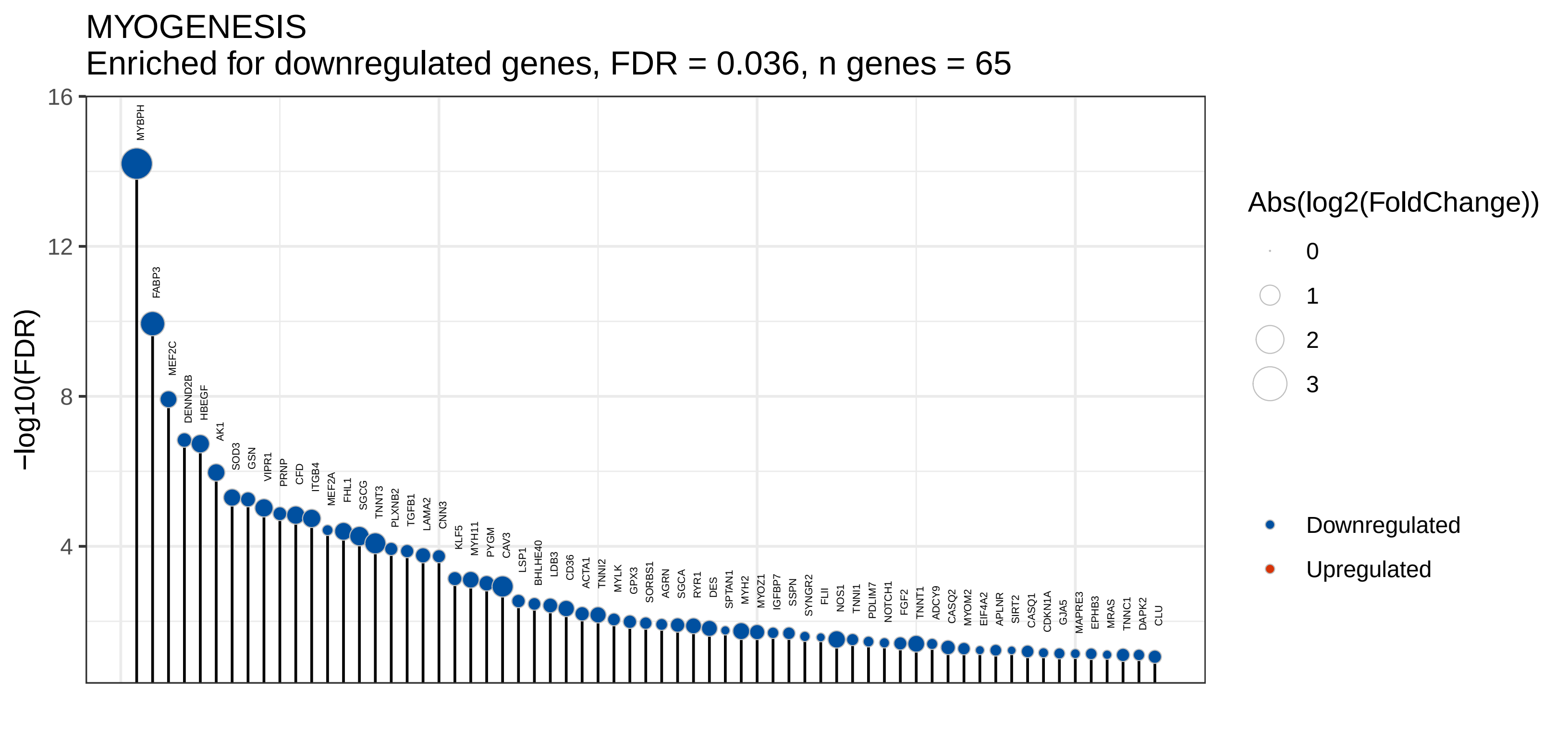

MYOGENESIS
Enriched for downregulated genes, FDR = 0.036, n genes = 65
-log10(FDR)
16
12
8
4
MYBPH
FABP3
MEF2C
DENND2B
HBEGF
AK1
SOD3
GSN
VIPR1
PRNP
CFD
ITGB4
MEF2A
FHL1
SGCG
TNNT3
PLXNB2
TGFB1
LAMA2
CNN3
KLF5
MYH11
PYGM
CAV3
LSP1
BHLHE40
LDB3
CD36
ACTA1
TNNI2
MYLK
GPX3
SORBS1
AGRN
SGCA
RYR1
DES
SPTAN1
MYH2
MYOZ1
IGFBP7
SSPN
SYNGR2
FLII
NOS1
TNNI1
PDLIM7
NOTCH1
FGF2
TNNT1
ADCY9
CASQ2
MYOM2
EIF4A2
APLNR
SIRT2
CASQ1
CDKN1A
GJA5
MAPRE3
EPHB3
MRAS
TNNC1
DAPK2
CLU
Abs(log2(FoldChange))
0
1
2
3
Downregulated
Upregulated

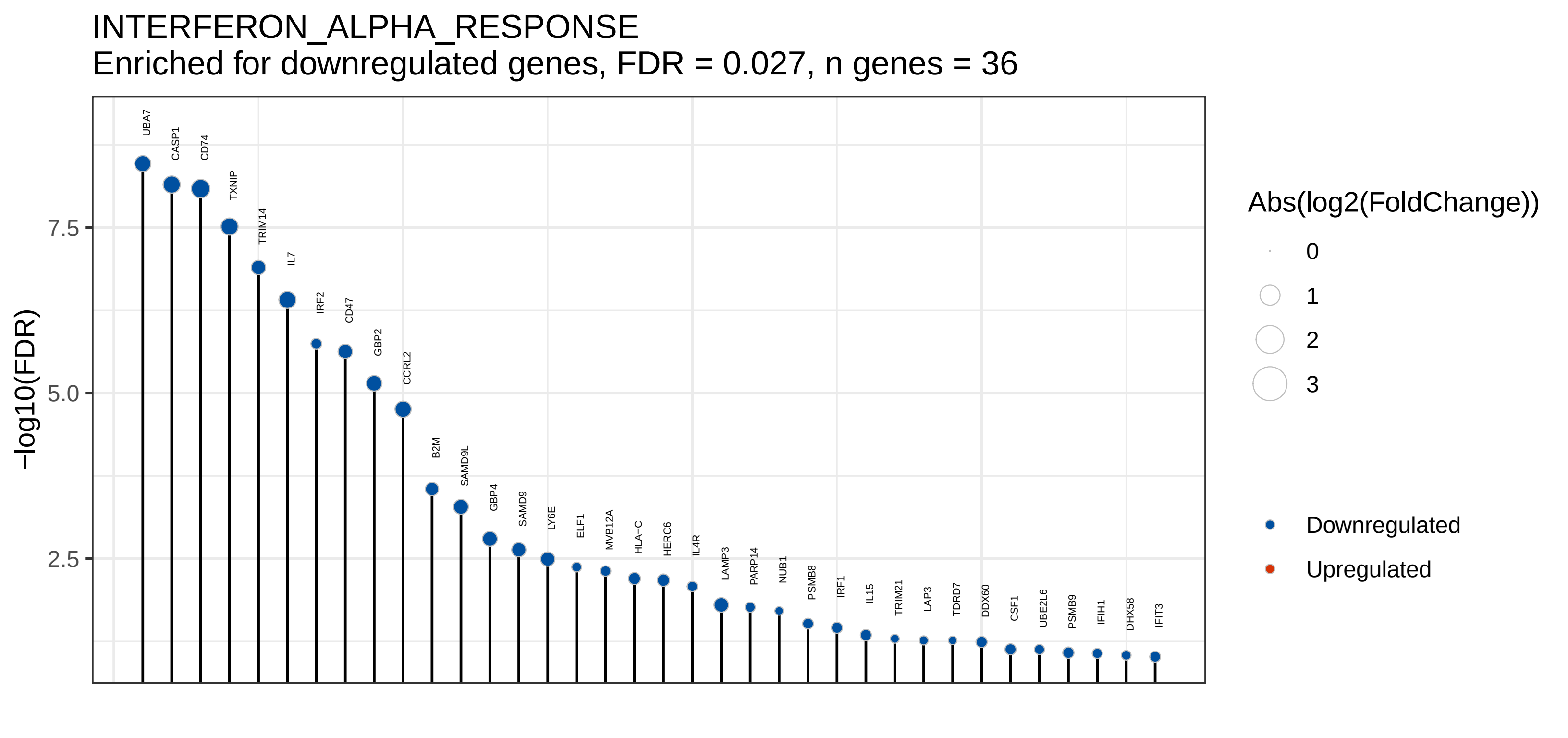

INTERFERON_ALPHA_RESPONSE
Enriched for downregulated genes, FDR = 0.027, n genes = 36
−log10(FDR)
7.5
5.0
2.5
UBA7
CASP1
CD74
TXNIP
TRIM14
IL7
IRF2
CD47
GBP2
CCRL2
B2M
SAMD9L
GBP4
SAMD9
LY6E
ELF1
MVB12A
HLA−C
HERC6
IL4R
LAMP3
PARP14
NUB1
PSMB8
IRF1
IL15
TRIM21
LAP3
TDRD7
DDX60
CSF1
UBE2L6
PSMB9
IFIH1
DHX58
IFIT3
Abs(log2(FoldChange))
0
1
2
3
Downregulated
Upregulated

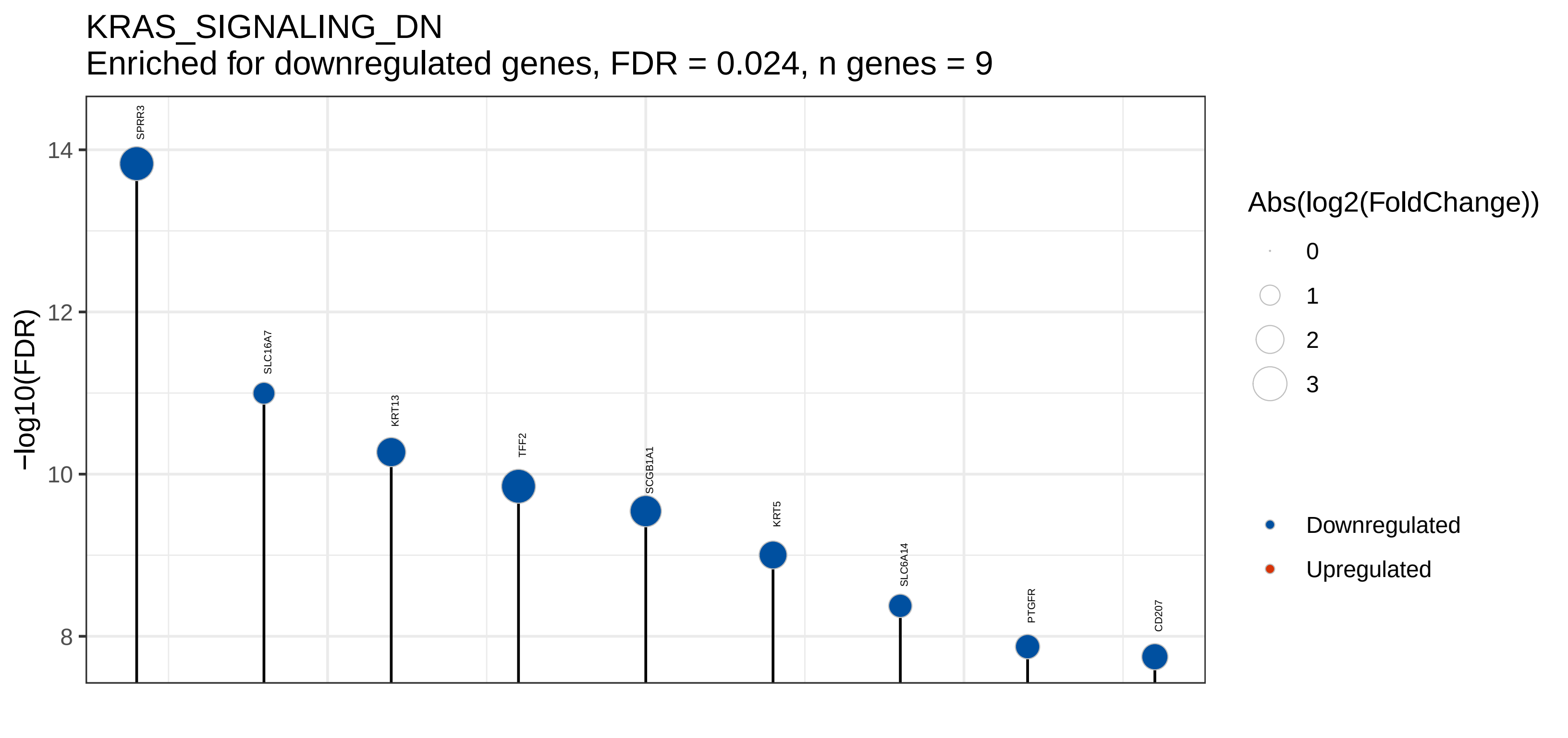

KRAS_SIGNALING_DN
Enriched for downregulated genes, FDR = 0.024, n genes = 9
-log10(FDR)
14
12
10
8
SPRR3
SLC16A7
KRT13
TFF2
SCGB1A1
KRT5
SLC6A14
PTGFR
CD207
Abs(log2(FoldChange))
0
1
2
3
Downregulated
Upregulated

APICAL_JUNCTION
Enriched for downregulated genes, FDR = 0.0012, n genes = 72

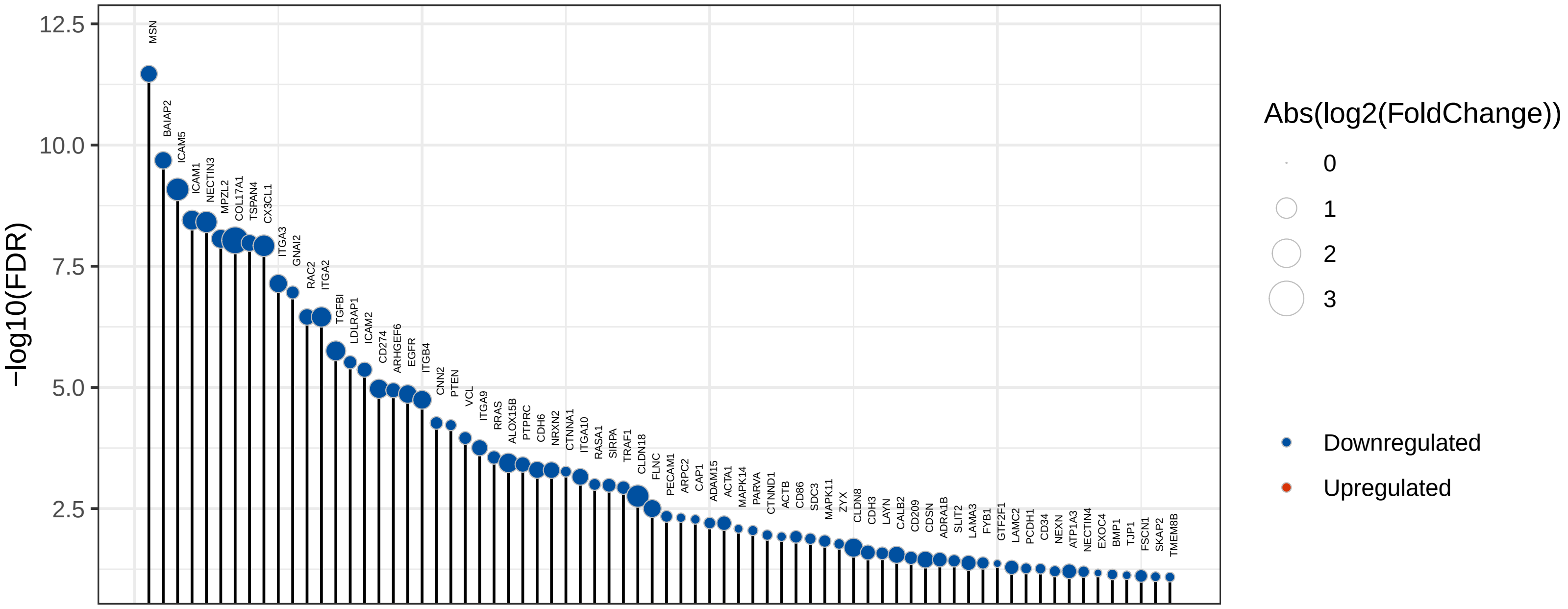

APOPTOSIS
Enriched for downregulated genes, FDR = 9.3e−05, n genes = 32

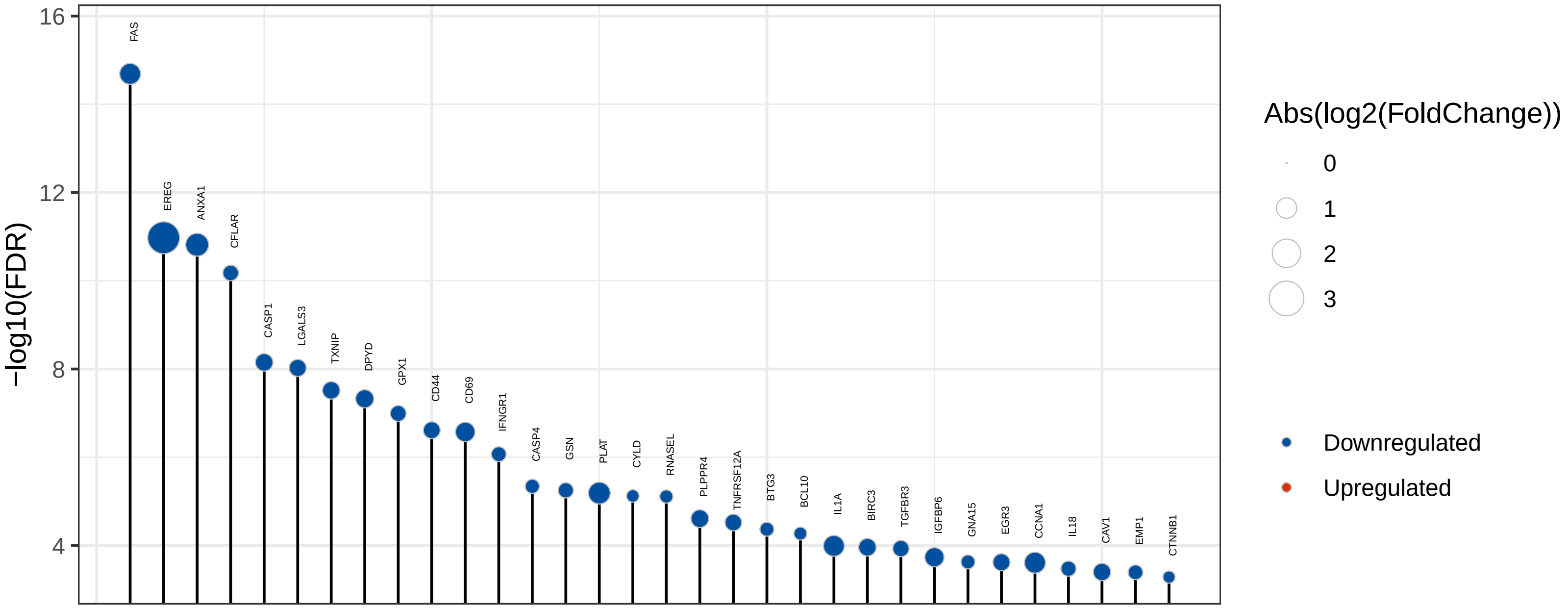

COMPLEMENT
Enriched for downregulated genes, FDR = 6e−05, n genes = 80

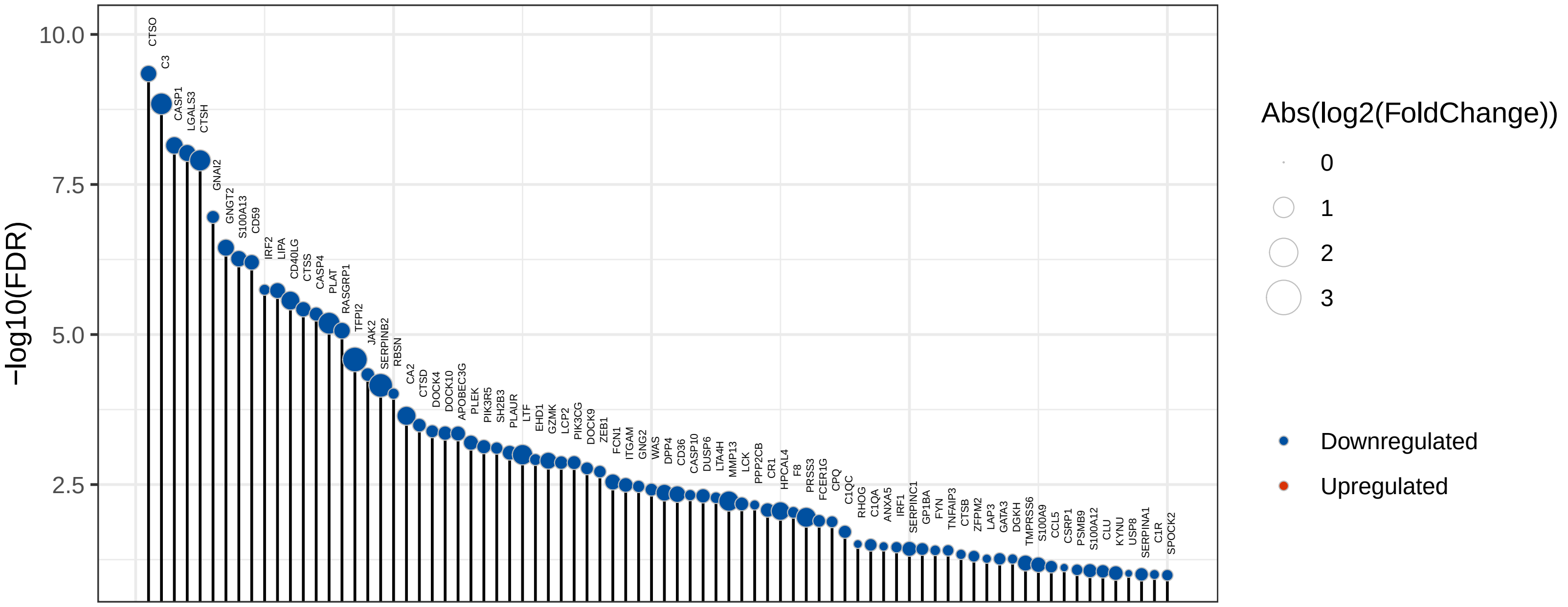

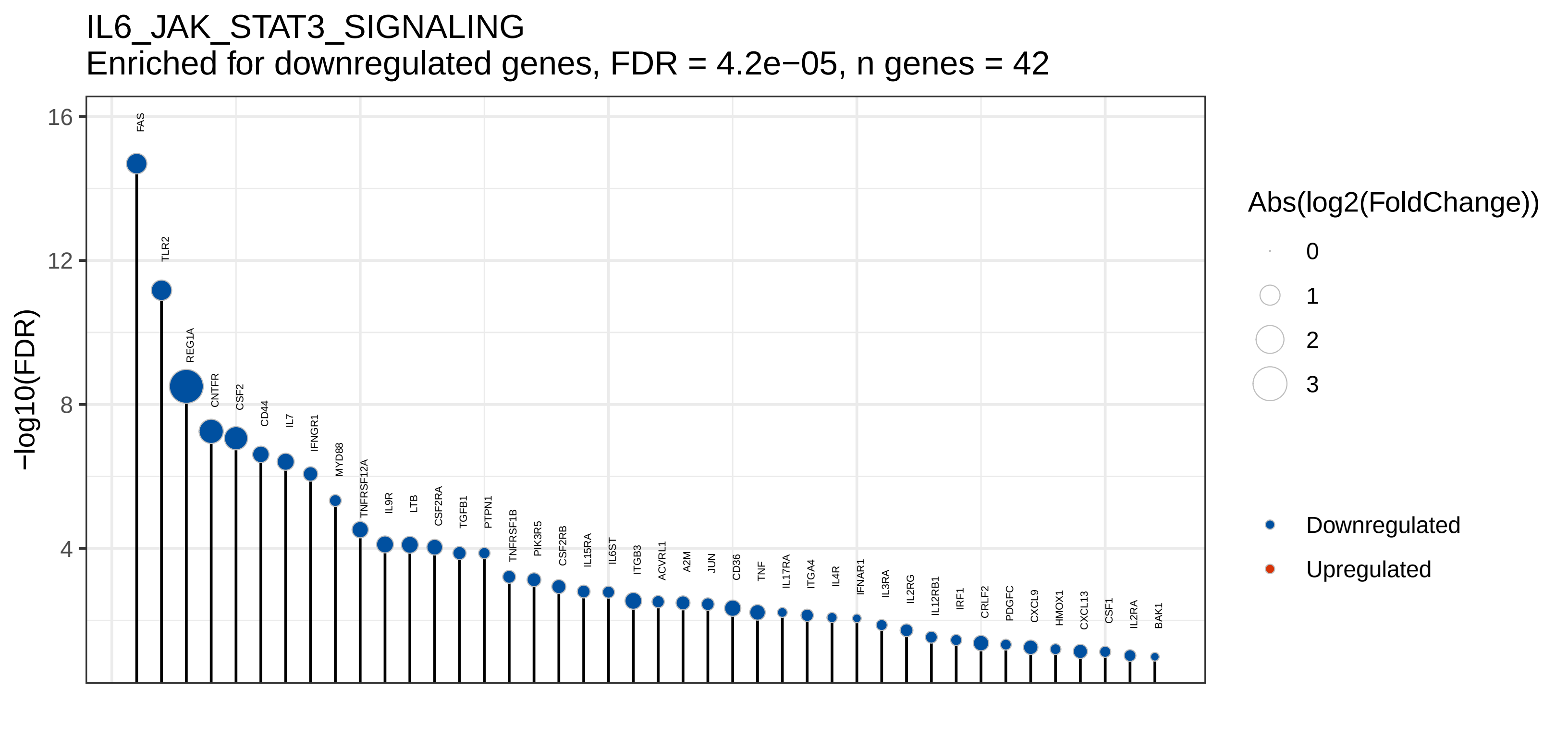

IL6_JAK_STAT3_SIGNALING
Enriched for downregulated genes, FDR = 4.2e−05, n genes = 42
−log10(FDR)
16
12
8
4
FAS
TLR2
REG1A
CNTFR
CSF2
CD44
IL7
IFNGR1
MYD88
TNFRSF12A
IL9R
LTB
CSF2RA
TGFB1
PTPN1
TNFRSF1B
PIK3R5
CSF2RB
IL15RA
IL6ST
ITGB3
ACVRL1
A2M
JUN
CD36
TNF
IL17RA
ITGA4
IL4R
IFNAR1
IL3RA
IL2RG
IL12RB1
IRF1
CRLF2
PDGFC
CXCL9
HMOX1
CXCL13
CSF1
IL2RA
BAK1
Abs(log2(FoldChange))
0
1
2
3
Downregulated
Upregulated

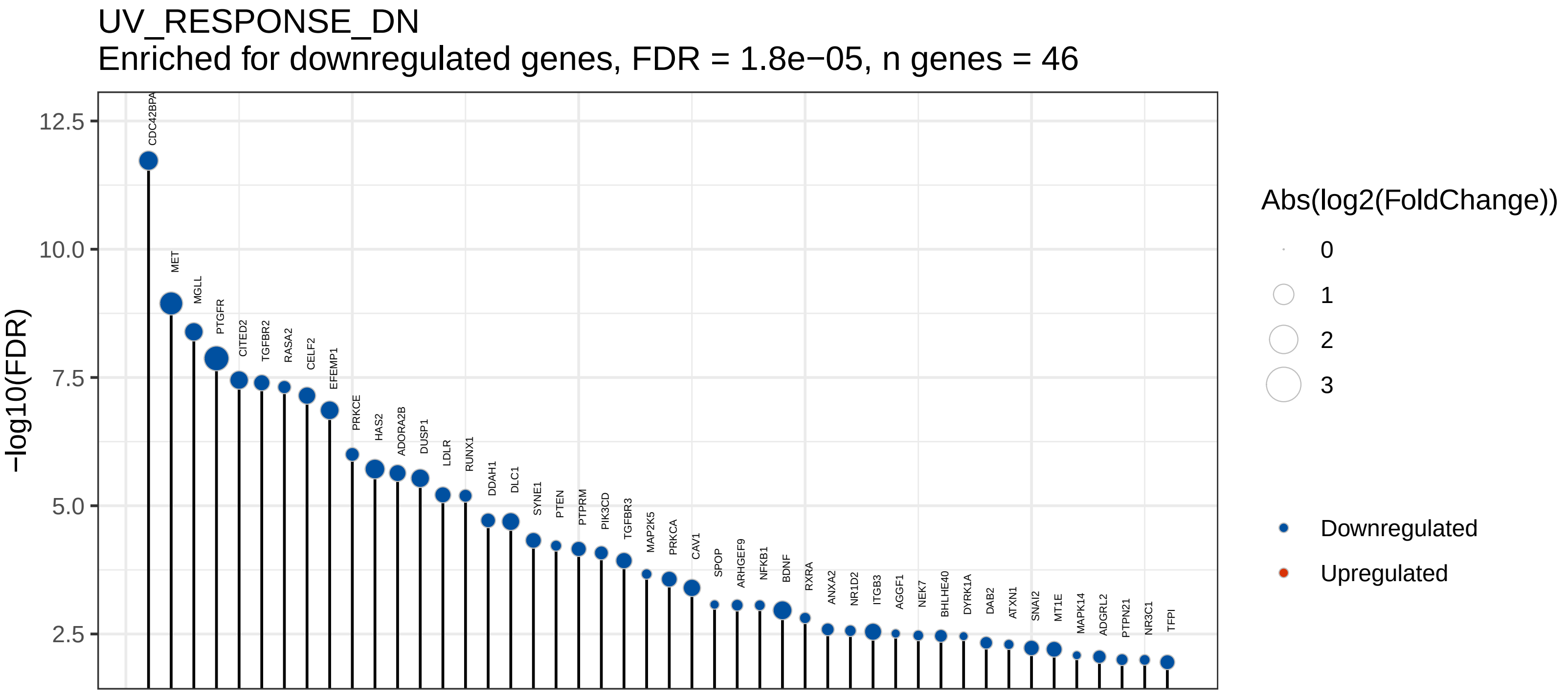
UV_RESPONSE_DN
Enriched for downregulated genes, FDR = 1.8e−05, n genes = 46
−log10(FDR)
12.5
10.0
7.5
5.0
2.5
CDC42BPA
MET
MGLL
PTGFR
CITED2
TGFBR2
RASA2
CELF2
EFEMP1
PRKCE
HAS2
ADORA2B
DUSP1
LDLR
RUNX1
DDAH1
DLC1
SYNE1
PTEN
PTPRM
PIK3CD
TGFBR3
MAP2K5
PRKCA
CAV1
SPOP
ARHGEF9
NFKB1
BDNF
RXRA
ANXA2
NR1D2
ITGB3
AGGF1
NEK7
BHLHE40
DYRK1A
DAB2
ATXN1
SNAI2
MT1E
MAPK14
ADGRL2
PTPN21
NR3C1
TFPI
Abs(log2(FoldChange))
0
1
2
3
Downregulated
Upregulated

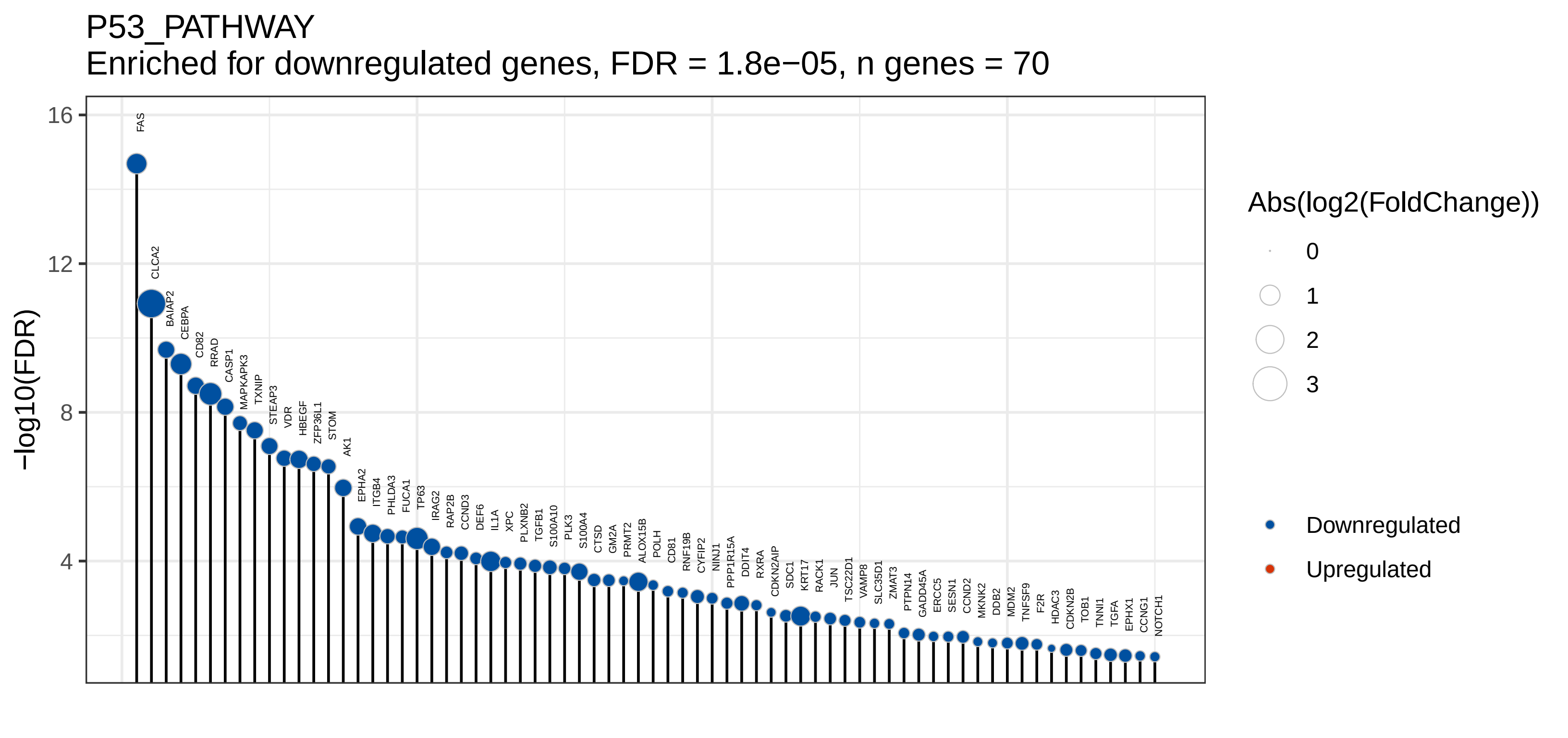
P53_PATHWAY
Enriched for downregulated genes, FDR = 1.8e−05, n genes = 70
−log10(FDR)
16
12
8
4
FAS
CLCA2
BAIAP2
CEBPA
CD82
RRAD
CASP1
MAPKAPK3
TXNIP
STEAP3
VDR
HBEGF
ZFP36L1
STOM
AK1
EPHA2
ITGB4
PHLDA3
FUCA1
TP63
IRAG2
RAP2B
CCND3
DEF6
IL1A
XPC
PLXNB2
TGFB1
S100A10
PLK3
S100A4
CTSD
GM2A
PRMT2
ALOX15B
POLH
CD81
RNF19B
CYFIP2
NINJ1
PPP1R15A
DDIT4
RXRA
CDKN2AIP
SDC1
KRT17
RACK1
JUN
TSC22D1
VAMP8
SLC35D1
ZMAT3
PTPN14
GADD45A
ERCC5
SESN1
CCND2
MKNK2
DDB2
MDM2
TNFSF9
F2R
HDAC3
CDKN2B
TOB1
TNNI1
TGFA
EPHX1
CCNG1
NOTCH1
Abs(log2(FoldChange))
0
1
2
3
Downregulated
Upregulated

INTERFERON_GAMMA_RESPONSE
Enriched for downregulated genes, FDR = 7.7e−08, n genes = 89

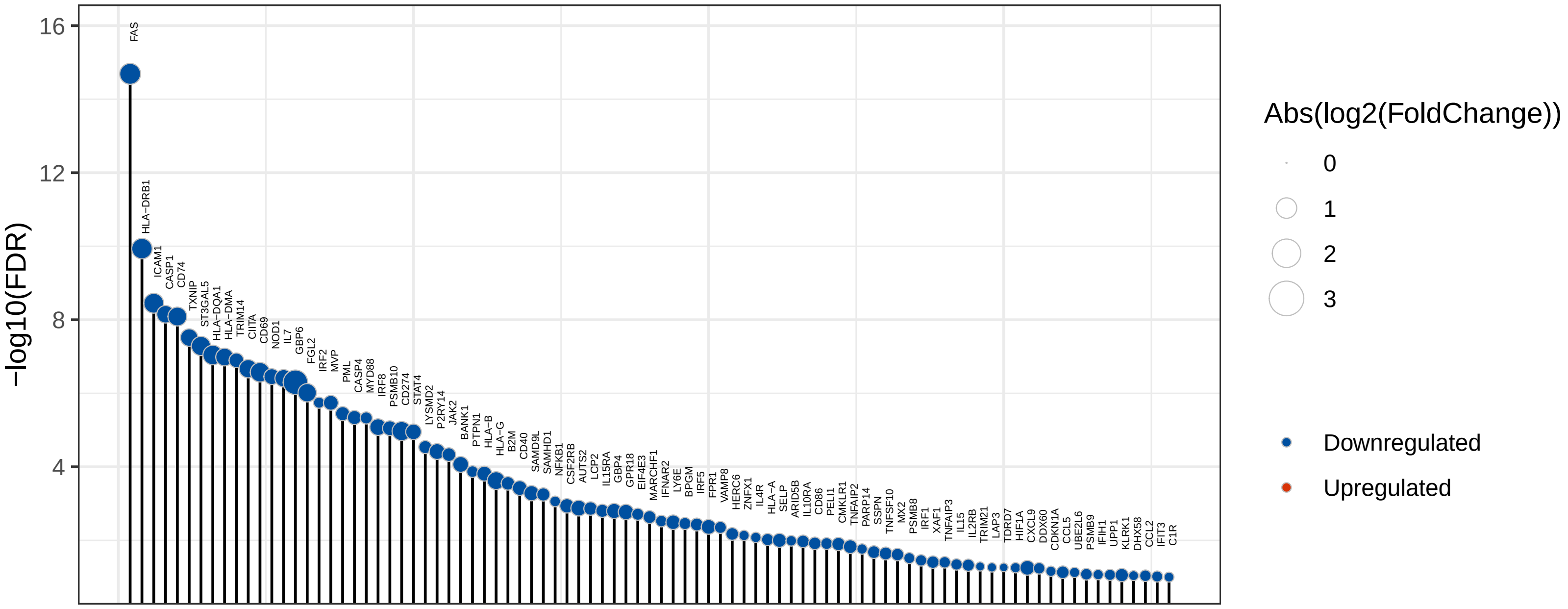

IL2_STAT5_SIGNALING
Enriched for downregulated genes, FDR = 2.3e−08, n genes = 90

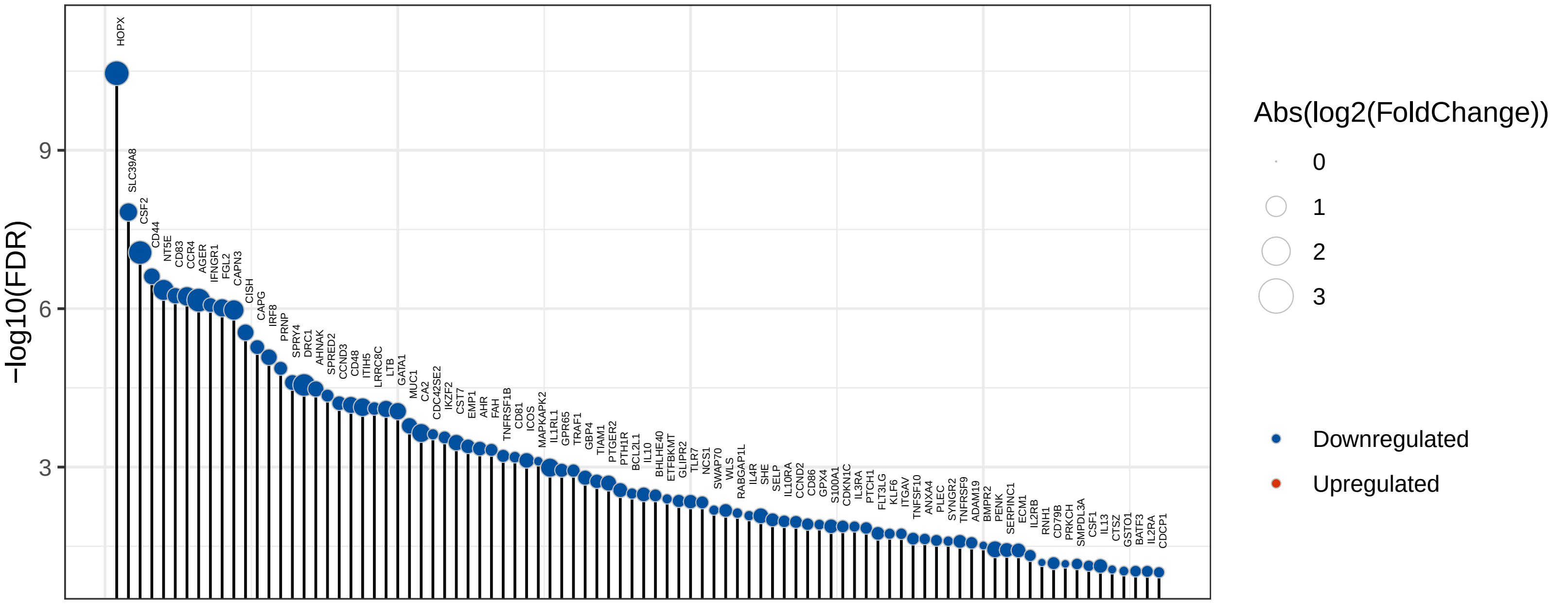

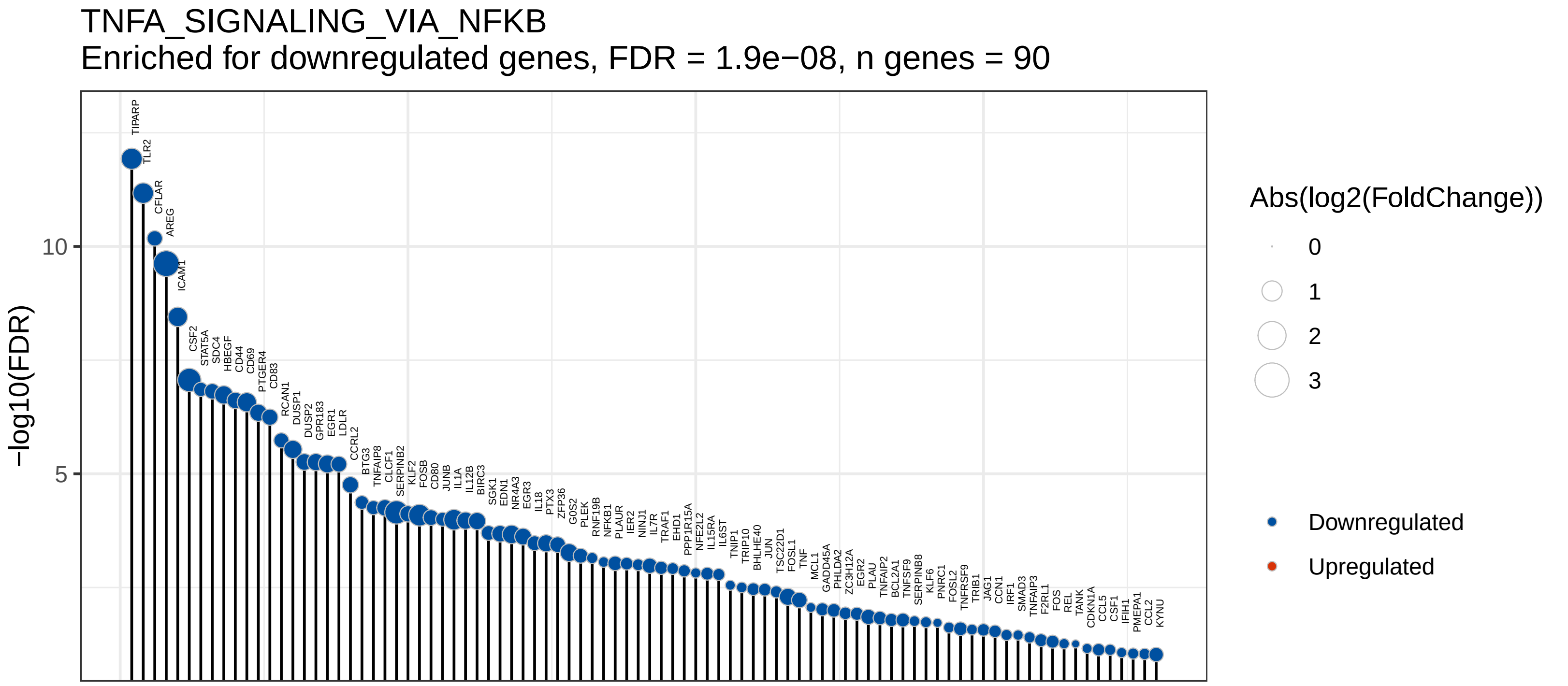
TNFA_SIGNALING_VIA_NFKB
Enriched for downregulated genes, FDR = 1.9e−08, n genes = 90
−log10(FDR)
10
5
TIPARP
TLR2
CFLAR
AREG
ICAM1
CSF2
STAT5A
SDC4
HBEGF
CD44
CD69
PTGER4
CD83
RCAN1
DUSP1
DUSP2
GPR183
EGR1
LDLR
CCRL2
BTG3
TNFAIP8
CLCF1
SERPINB2
KLF2
FOSB
CD80
JUNB
IL1A
IL12B
BIRC3
SGK1
EDN1
NR4A3
EGR3
IL18
PTX3
ZFP36
G0S2
PLEK
RNF19B
NFKB1
PLAUR
IER2
NINJ1
IL7R
TRAF1
EHD1
PPP1R15A
NFE2L2
IL15RA
IL6ST
TNIP1
TRIP10
BHLHE40
JUN
TSC22D1
FOSL1
TNF
MCL1
GADD45A
PHLDA2
ZC3H12A
EGR2
PLAU
TNFAIP2
BCL2A1
TNFSF9
SERPINB8
KLF6
PNRC1
FOSL2
TNFRSF9
TRIB1
JAG1
CCN1
IRF1
SMAD3
TNFAIP3
F2RL1
FOS
REL
TANK
CDKN1A
CCL5
CSF1
IFIH1
PMEPA1
CCL2
KYNU
Abs(log2(FoldChange))
0
1
2
3
Downregulated
Upregulated

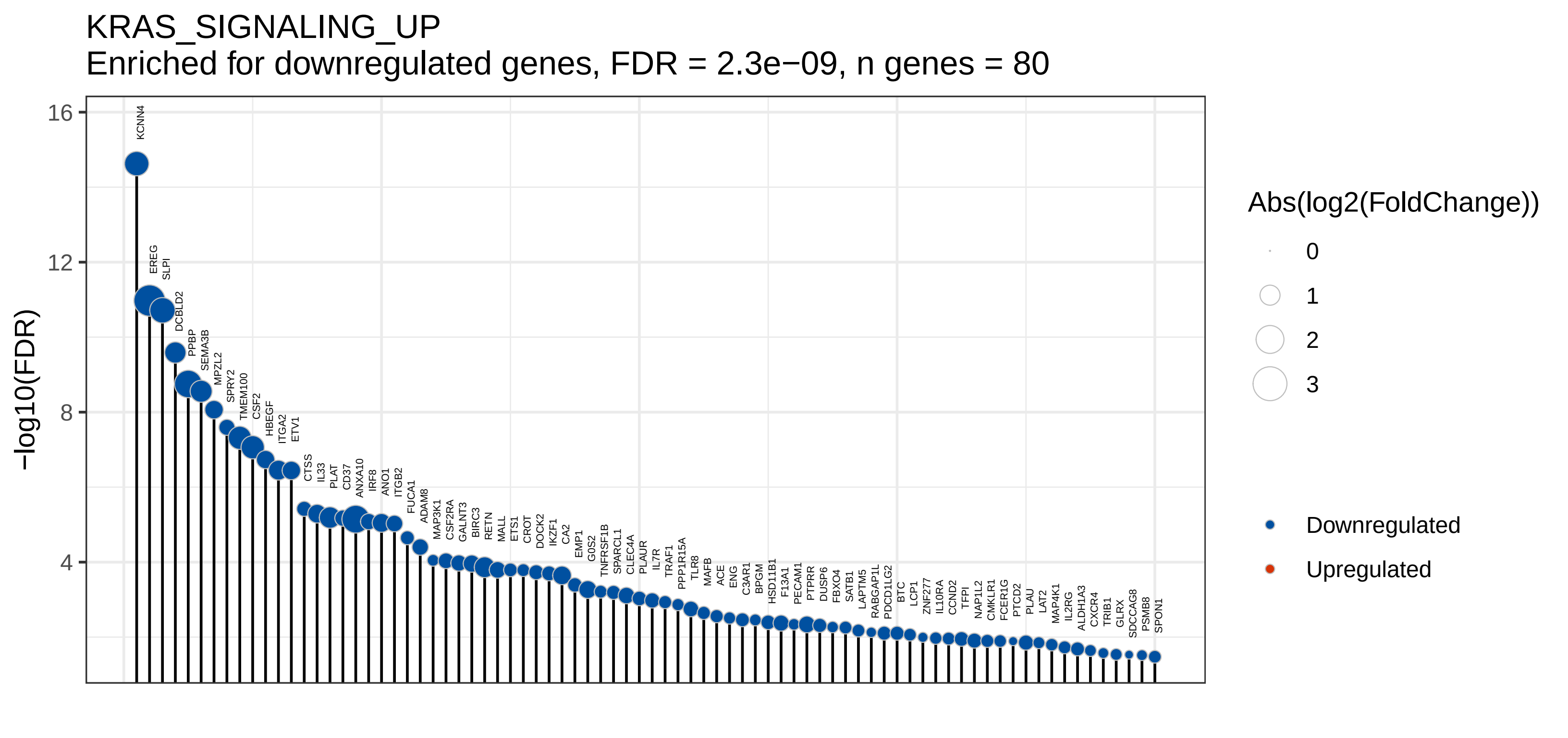
KRAS_SIGNALING_UP
Enriched for downregulated genes, FDR = 2.3e−09, n genes = 80
−log10(FDR)
16
12
8
4
Abs(log2(FoldChange))
0
1
2
3
Downregulated
Upregulated
KCNN4
EREG
SLPI
DCBLD2
PPBP
SEMA3B
MPZL2
SPRY2
TMEM100
CSF2
HBEGF
ITGA2
ETV1
CTSS
IL33
PLAT
CD37
ANXA10
IRF8
ANO1
ITGB2
FUCA1
ADAM8
MAP3K1
CSF2RA
GALNT3
BIRC3
RETN
MALL
ETS1
CROT
DOCK2
IKZF1
CA2
EMP1
G0S2
TNFRSF1B
SPARCL1
CLEC4A
PLAUR
IL7R
TRAF1
PPP1R15A
TLR8
MAFB
ACE
ENG
C3AR1
BPGM
HSD11B1
F13A1
PECAM1
PTPRR
DUSP6
FBXO4
SATB1
LAPTM5
RABGAP1L
PDCD1LG2
BTC
LCP1
ZNF277
IL10RA
CCND2
TFPI
NAP1L2
CMKLR1
FCER1G
PTCD2
PLAU
LAT2
MAP4K1
IL2RG
ALDH1A3
CXCR4
TRIB1
GLRX
SDCCAG8
PSMB8
SPON1

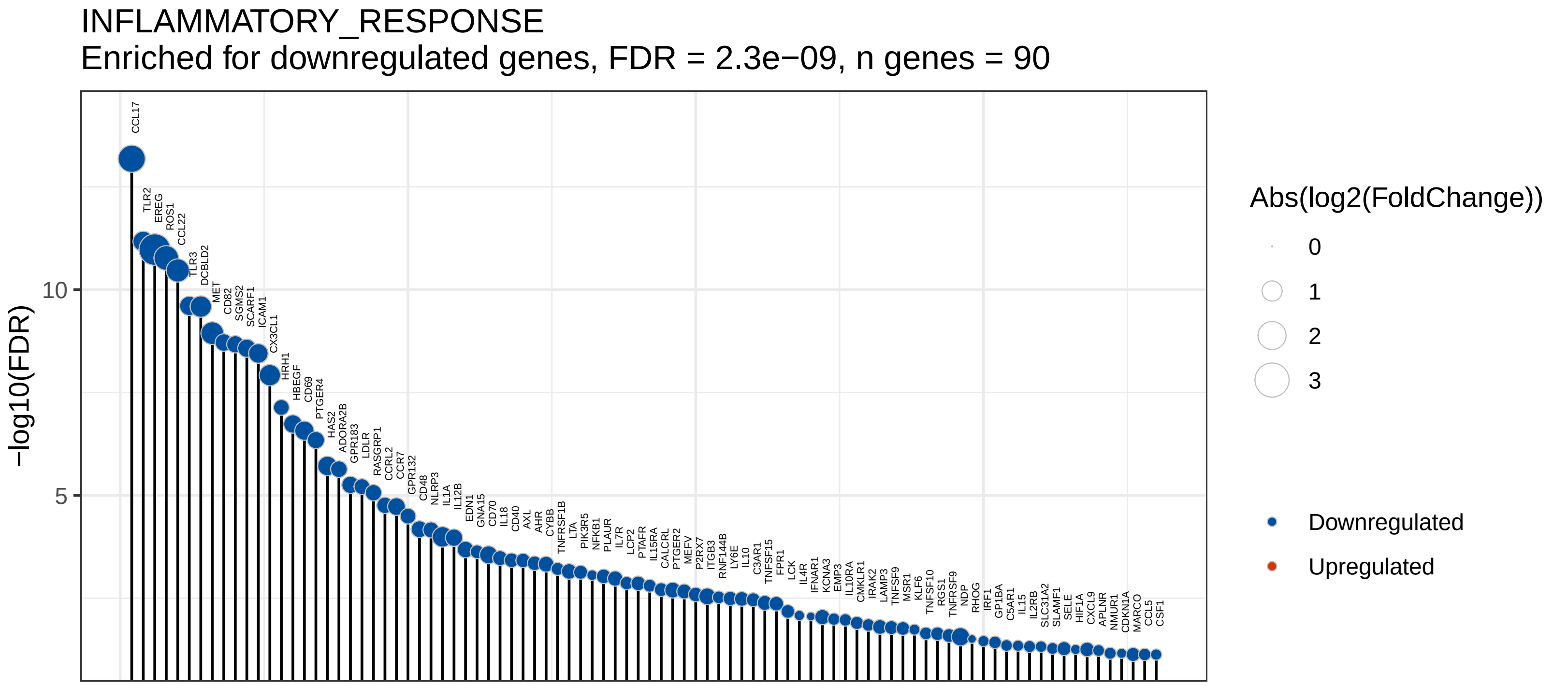
INFLAMMATORY_RESPONSE
Enriched for downregulated genes, FDR = 2.3e−09, n genes = 90
−log10(FDR)
10
5
Abs(log2(FoldChange))
0
1
2
3
Downregulated
Upregulated
CCL17
TLR2
EREG
ROS1
CCL22
TLR3
DCBLD2
MET
CD82
SGMS2
SCARF1
ICAM1
CX3CL1
HRH1
HBEGF
CD69
PTGER4
HAS2
ADORA2B
GPR183
LDLR
RASGRP1
CCRL2
CCR7
GPR132
CD48
NLRP3
IL1A
IL12B
EDN1
GNA15
CD70
IL18
CD40
AXL
AHR
CYBB
TNFRSF1B
LTA
PIK3R5
NFKB1
PLAUR
IL7R
LCP2
PTAFR
IL15RA
CALCRL
PTGER2
MEFV
P2RX7
ITGB3
RNF144B
LY6E
IL10
C3AR1
TNFSF15
FPR1
LCK
IL4R
IFNAR1
KCNA3
EMP3
IL10RA
CMKLR1
IRAK2
LAMP3
TNFSF9
MSR1
KLF6
TNFSF10
RGS1
TNFRSF9
NDP
RHOG
IRF1
GP1BA
C5AR1
IL15
IL2RB
SLC31A2
SLAMF1
SELE
HIF1A
CXCL9
APLNR
NMUR1
CDKN1A
MARCO
CCL5
CSF1

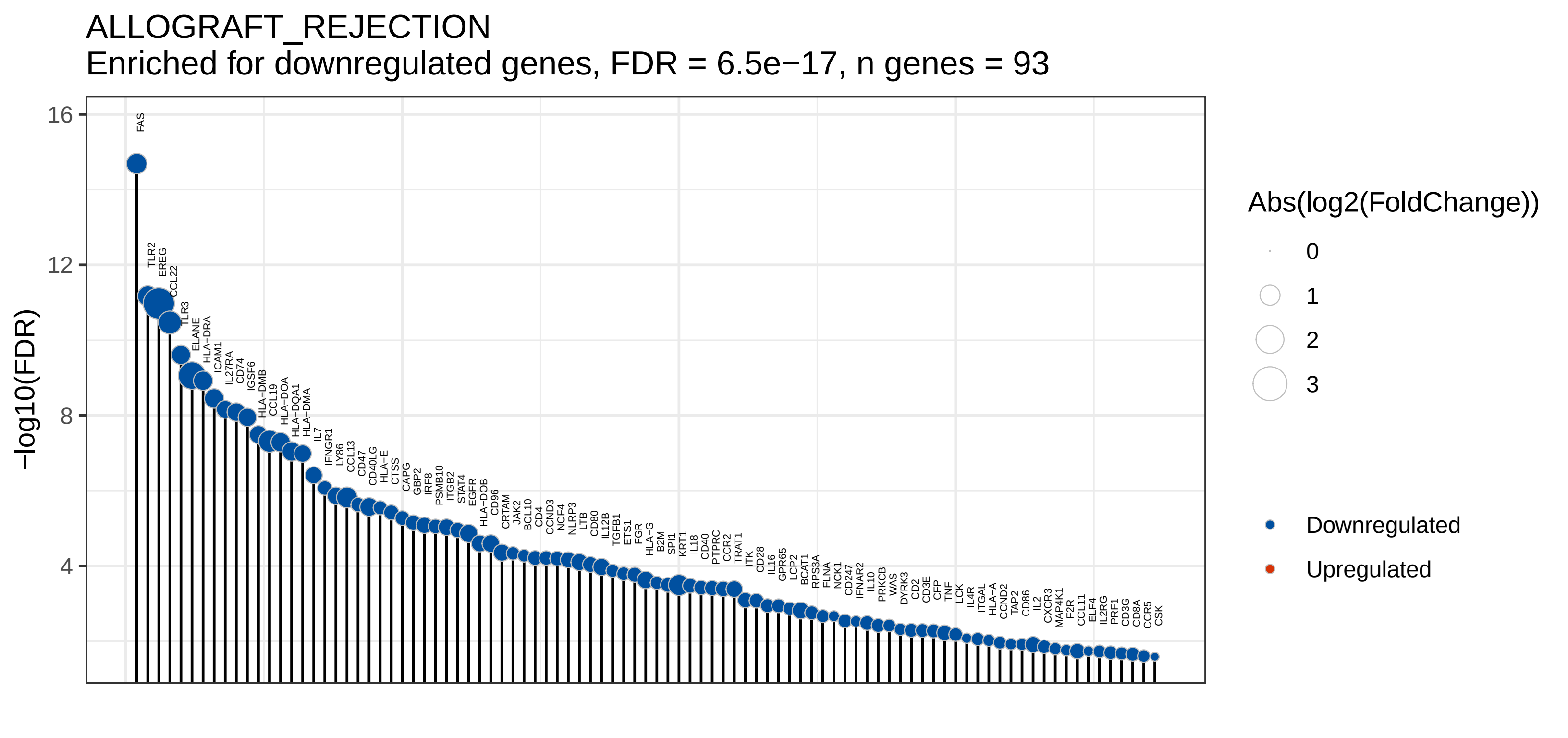
ALLOGRAFT_REJECTION
Enriched for downregulated genes, FDR = 6.5e−17, n genes = 93
−log10(FDR)
16
12
8
4
FAS
TLR2
EREG
CCL22
TLR3
ELANE
HLA−DRA
ICAM1
IL27RA
CD74
IGSF6
HLA−DMB
CCL19
HLA−DOA
HLA−DQA1
HLA−DMA
IL7
IFNGR1
LY86
CCL13
CD47
CD40LG
HLA−E
CTSS
CAPG
GBP2
IRF8
PSMB10
ITGB2
STAT4
EGFR
HLA−DOB
CD96
CRTAM
JAK2
BCL10
CD4
CCND3
NCF4
NLRP3
LTB
CD80
IL12B
TGFB1
ETS1
FGR
HLA−G
B2M
SPI1
KRT1
IL18
CD40
PTPRC
CCR2
TRAT1
ITK
CD28
IL16
GPR65
LCP2
BCAT1
RPS3A
FLNA
NCK1
CD247
IFNAR2
IL10
PRKCB
WAS
DYRK3
CD2
CD3E
CFP
TNF
LCK
IL4R
ITGAL
HLA−A
CCND2
TAP2
CD86
IL2
CXCR3
MAP4K1
F2R
CCL11
ELF4
IL2RG
PRF1
CD3G
CD8A
CCR5
CSK
Abs(log2(FoldChange))
0
1
2
3
Downregulated
Upregulated
