# Supplementary material for: In vivo CRISPR screens reveal Serpinb9 and Adam2 as regulators of immune therapy response in lung cancer
Source: Nat Commun. 2023 May 31;14:3150. doi: 10.1038/s41467-023-38841-7 (PMC10232477; doi:10.1038/s41467-023-38841-7)
Supplement: Supplementary file 13 — Source Data [file 41467_2023_38841_MOESM13_ESM.zip › Supplementary Figure 12.zip!/13 mice slides with lung Adenocarcinoma.pdf]

Histologic evaluation of 13 slides of mice lung with carcinomas:

The tumors in the 13 slides resemble human **invasive nonmucinous adenocarcinoma of the lung**, with a mixture of lowgrade (lepidic) and high grade (solid, micropapillary) patterns.
Acinar pattern is rare. Typical papillary pattern with fibrovascular cores is not seen.

The grow mostly multifocal or confluent, like in metastatic disease, growth patterns as this are also seen in (multifocal) invasive mucinous adenocarcinoma of the lung in human.

The tumorcells show little to some atypia with intracellular vacuoles but no real extracellular mucin production, so they are non mucinous carcinomas by definition. Most of the nodules show invasive growth with destruction of lung architecture.

Patterndescription (and grading) is difficult as there is almost no stroma within the tumors. (Patterns in human lung adenocarcinoma are somewhat defined by relation of tumor growth to stroma)

Therefore it is difficult to distinguish lepidic (preinvasive along pre-existing lung structures, G1 patten) from micropapillary (small tufts of tumorcells, small papillary structures without fibrovascular core) and sometimes even solid pattern (sheets of tumorcells without acinar or papillary structures), both are G3 patterns (by currend definition (WHO 2021: G3 tumor is defined by having at least 20% of high grade pattern)

Papillary pattern is not seen (no stroma no fibrovascular cores), acinar patten is sometimes visible but not as common and easily seen as in human lung cancer samples.

These patterns seem typically for tumors grown in mouse models described as “bronchioalveolar”. This term was actually discontinued in human tumors with the WHO Classification of 2015 as it included both low grade (lepidic) and high grade (micropapillary) and seems therefore actually quite fitting for the tumors in mice.

A further problem with grading would be definition of tumorislands as multiple tumors as then every tumorcell nests would have to be graded. But also if the tumors were defined as one multifocal process, grading is in my opinion not to recommend, as high and low grade patterns are not easily distinguishable and therefore pattern % as needed for grading cannot be evaluated properly. This are problem arises also in human lung carcinomas that typicaly show that kind of growth pattern, namely invasive mucinous adenocarcinoma, and they are still not recommended to be graded

Immune cell infiltrates are seen at the borders of the tumors and around vascular and bronchiolar structures. Intraalveolar macrophages commonly seen, sometimes multinucleated cells and sometimes intraalveolar single cells with somewhat atypical nuclei and broad eosinophilic cytoplasm without immunohistochemistry difficult to say if all of them are just macrophages or some tumorcells as well.

1 HE.9758-3ovab6 (Lung Adeno Carcinoma) multifocal multiple smaller nodules

Lepidic pattern with immune cell infiltrate around a vein. Note the borders of the island with possibility to follow the alveolar structure. Some small acinar structures at the border of the immune cell infiltrate.

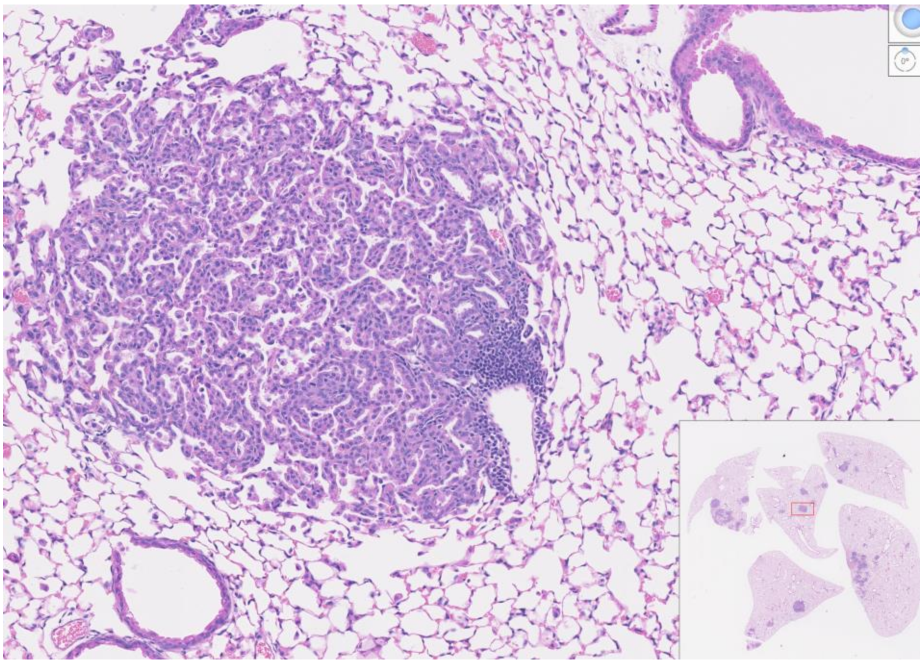

Same sample other site:

More nodular tumorformations, alveoli seem to be pushed away by sheets of tumorcells and small tumortufts (micropapillary structures without fibrovascular cores). Some acinar structures at the border of the immune cell infiltrate. The Pattern is for best described as micropapillary.

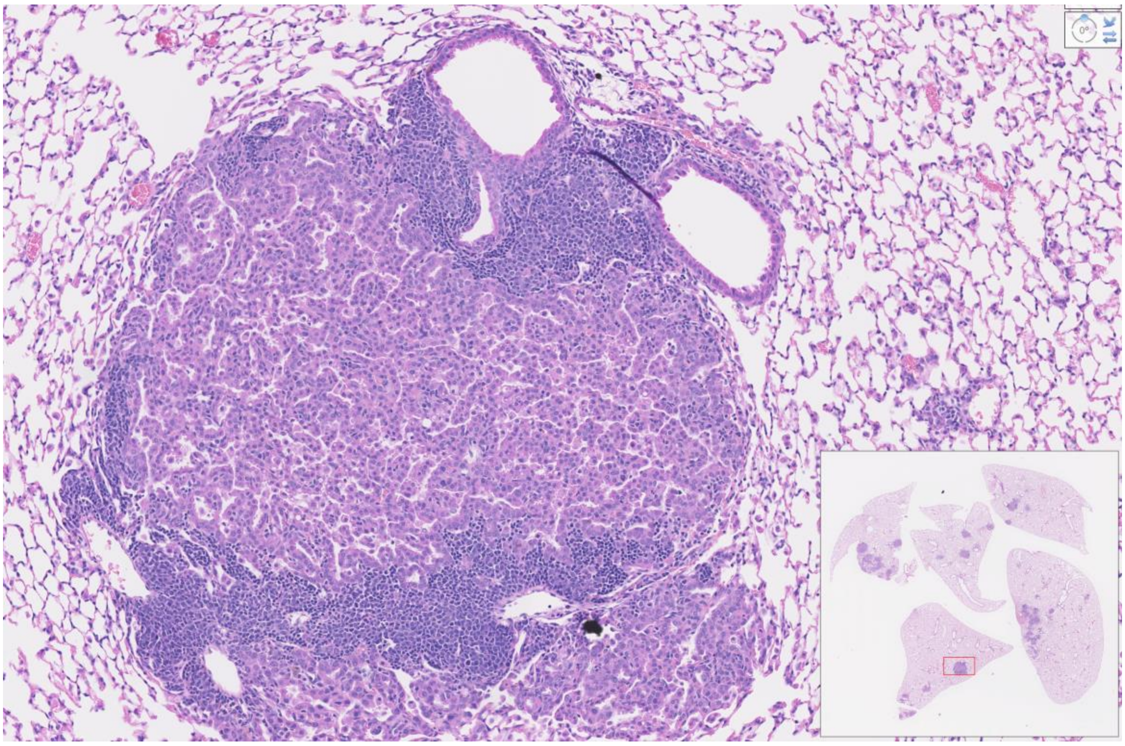

2 HE.4308-3ovab6 (Lung Adeno Carcinoma)

Large confluent tumor mass with some dense nodules. In the periphery lepidic, centrally micropapillary, nodules solid,

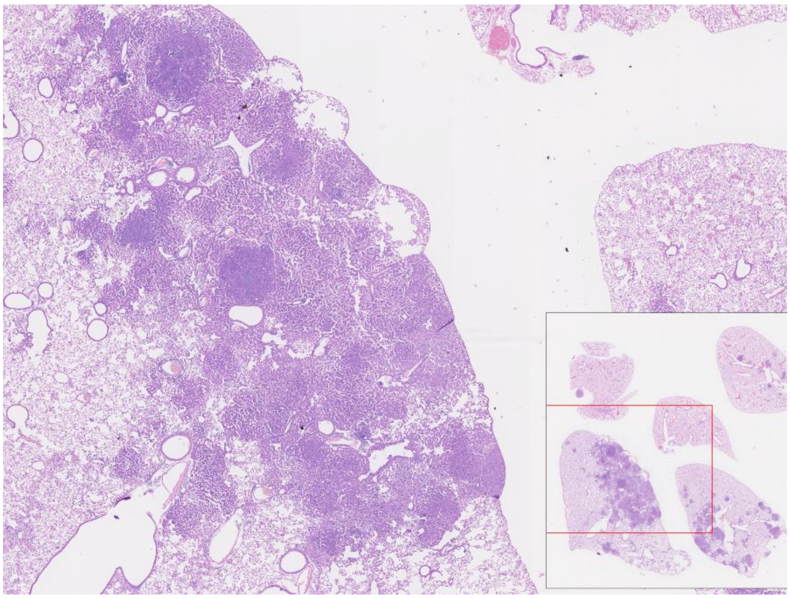

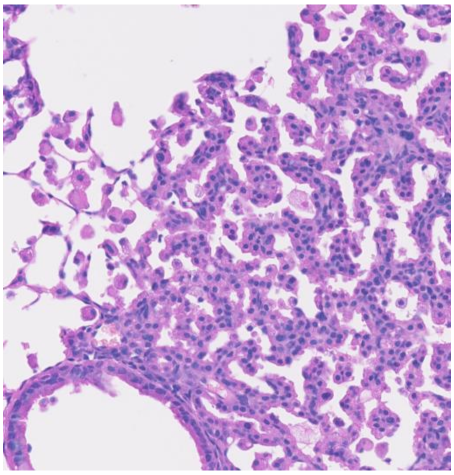

Vacuols but no extracellular mucin production

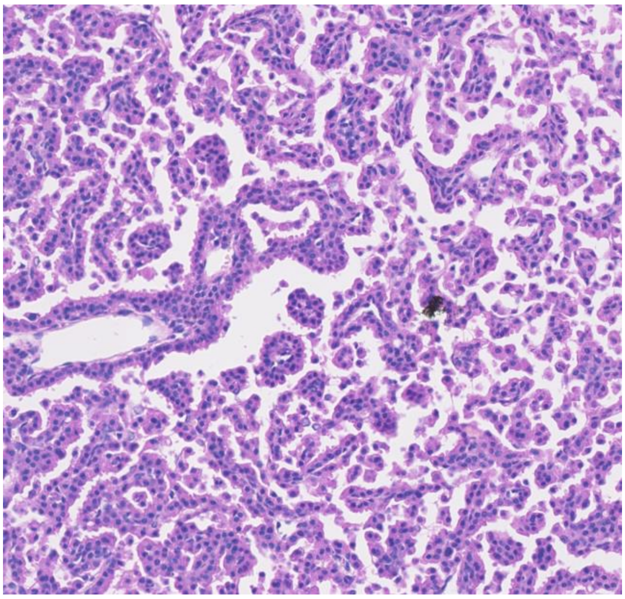

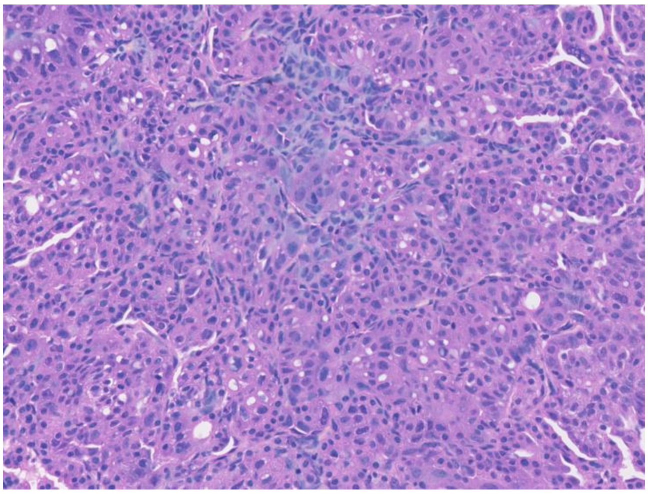

little lymphoplasmacytic infiltration near artery and bronchus

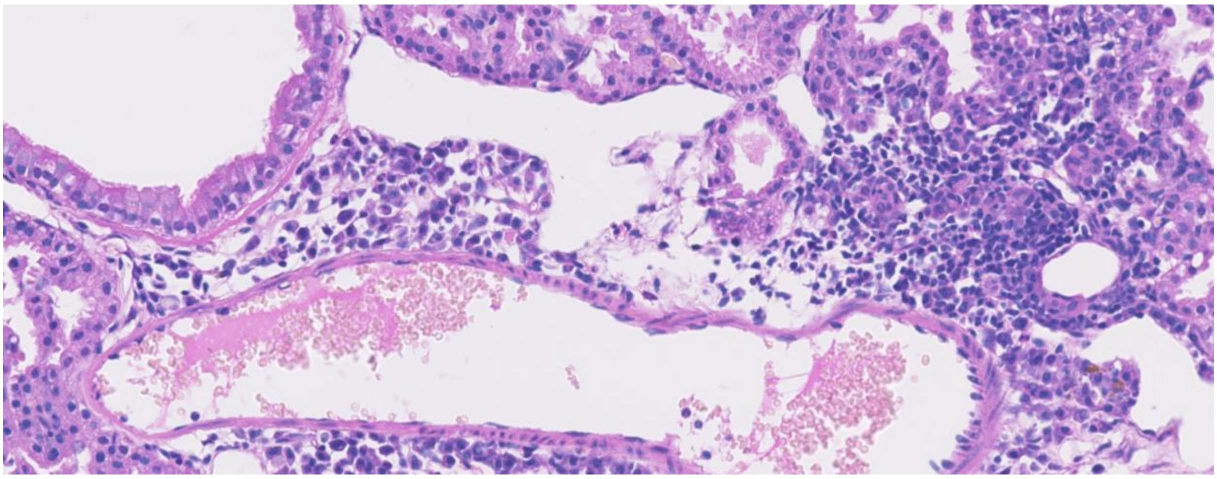

3 HE.9007.ADAM2 multifocal lung Adenocarcinoma

Tumor with a pattern closest to acinar adenocarcinoma (nodules with holes), denser lymphoplasmacytic infiltrates at tumorest borders and around vessels and bronchioli

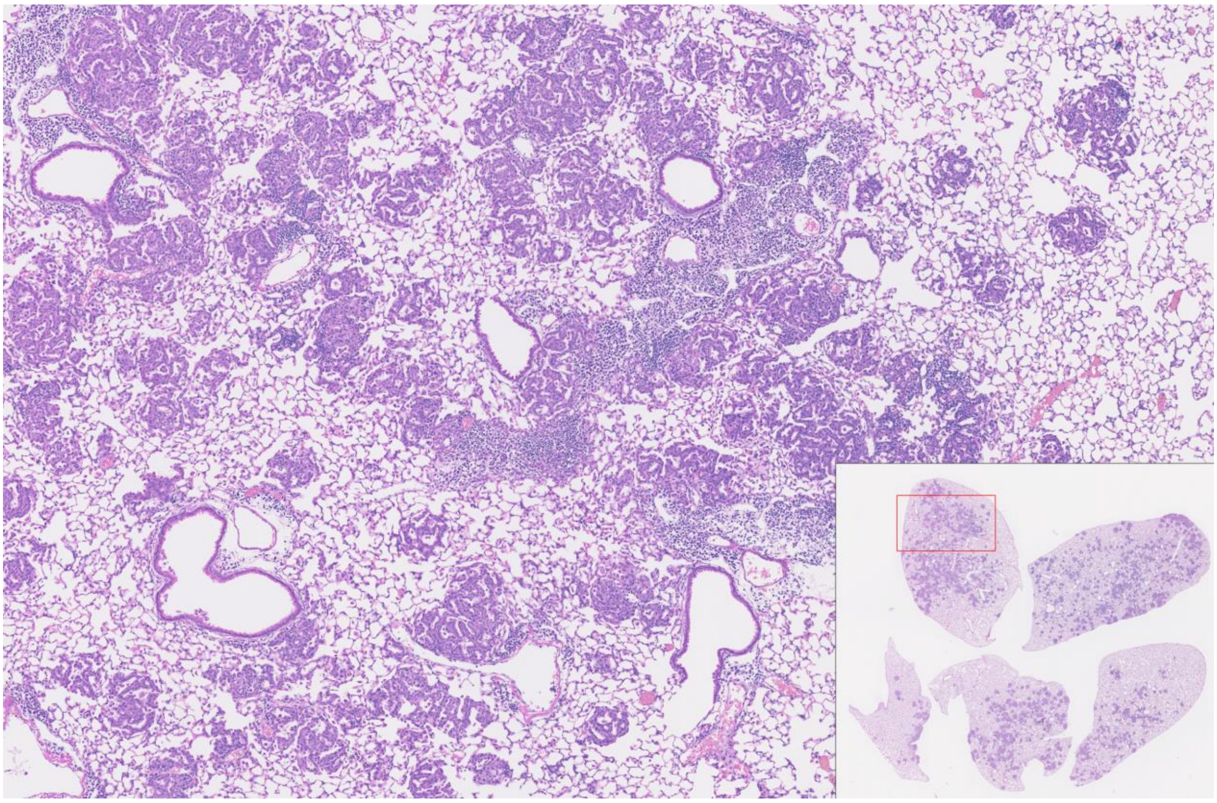

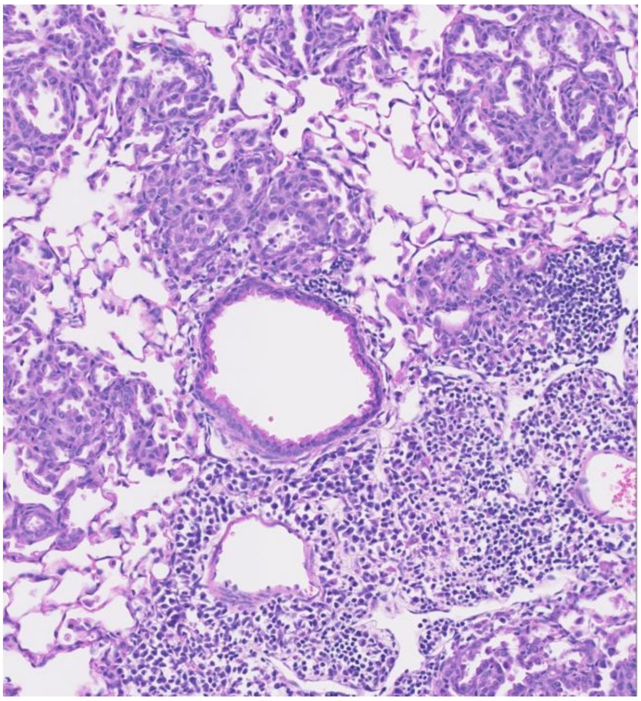

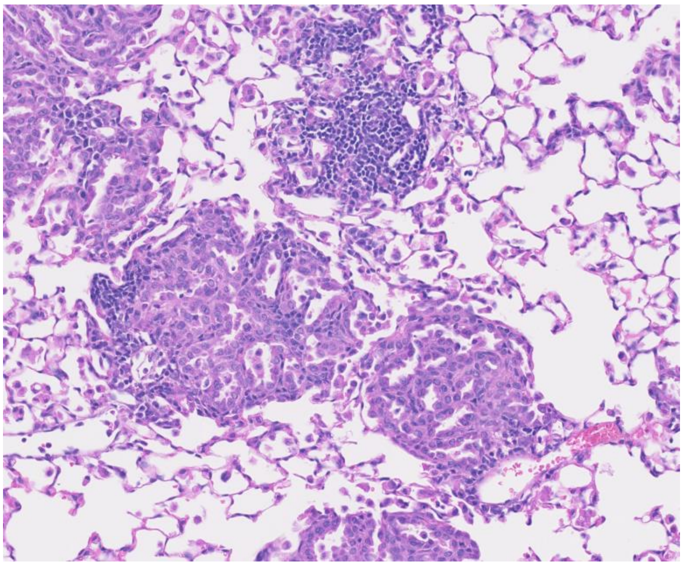

4 HE.7561.Lib3.B6NI

Multifocal to confluent lepidic, solid and micropapillary structures, fewer lymphatic infiltrates , some subpleural almost emysematic changes, intraalveolar macrophages/single and multicucleated cells

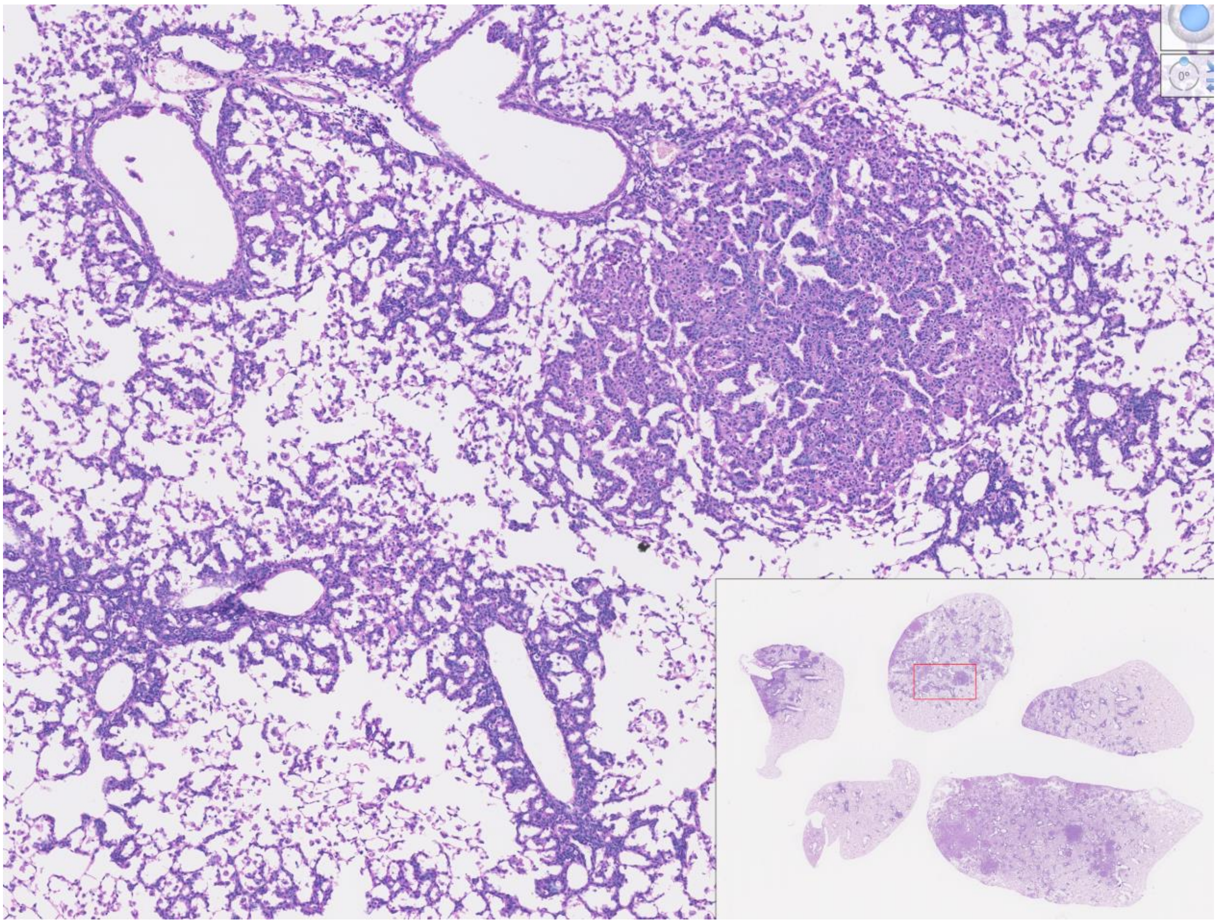

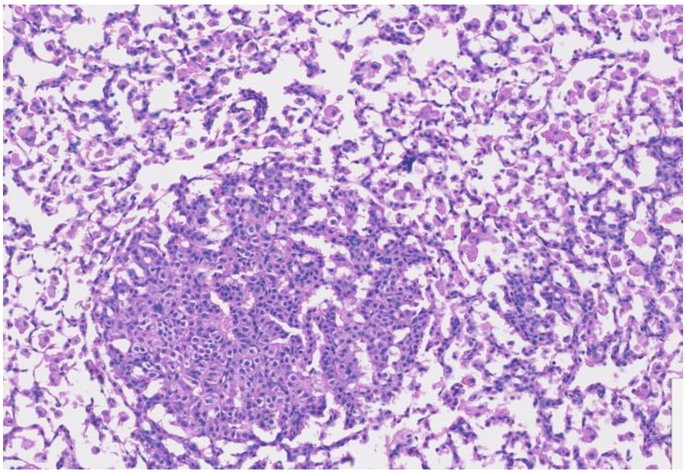

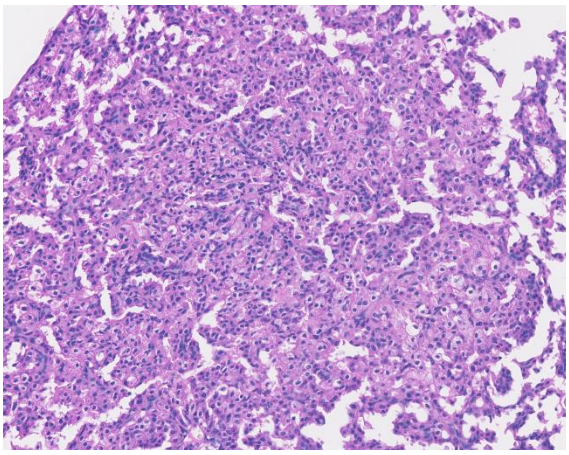

5 H.4304.3ovab6

Confluent nodules (solid, micropapillary, some lepidic), few immune cells

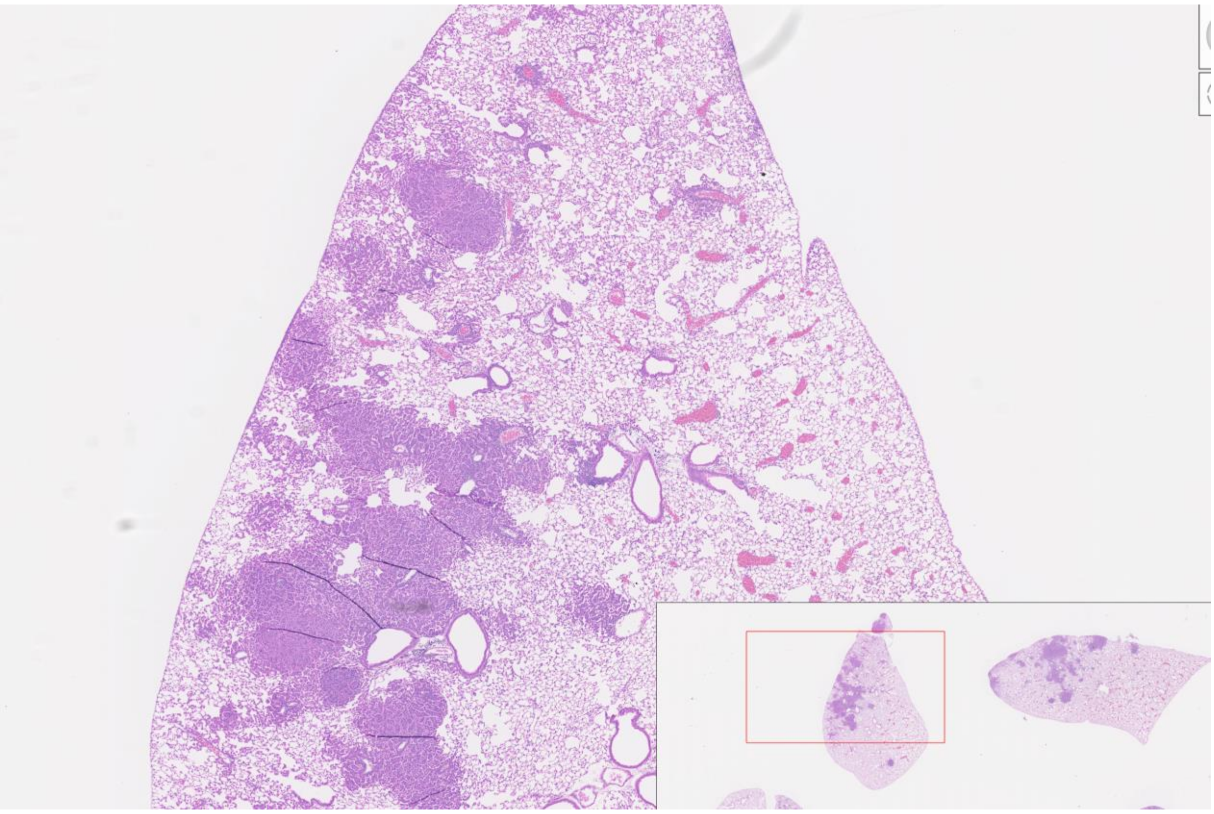

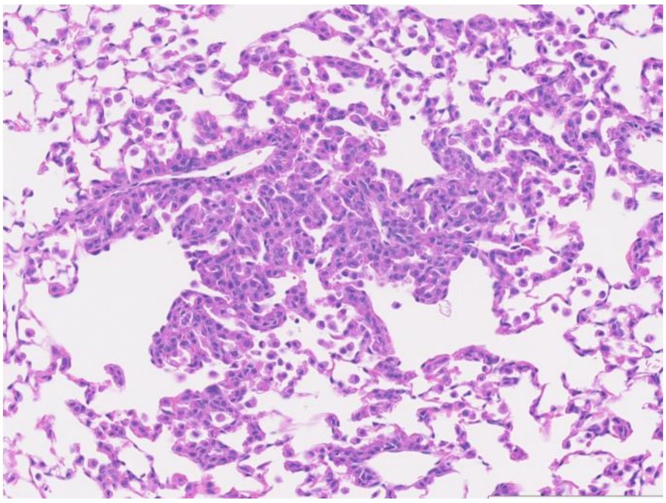

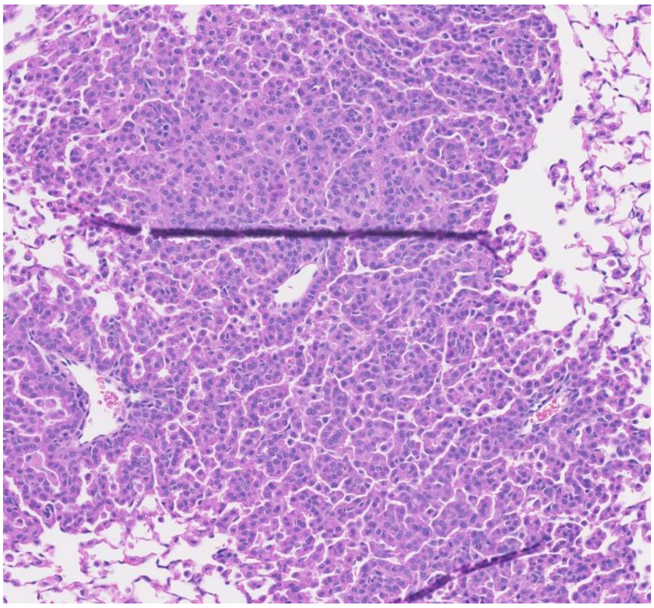

Lepidic pattern solid and micropapillary →

6 HE.2773-3ovabl

Multifocal, partly Confluent nodules with solid lepidic and micropapillary structures

intraalveolar atypical round sometimes multinucleated, vacuolated (foamy) cells probably macrophages or tumor cells, some lymphos and plasma cells.

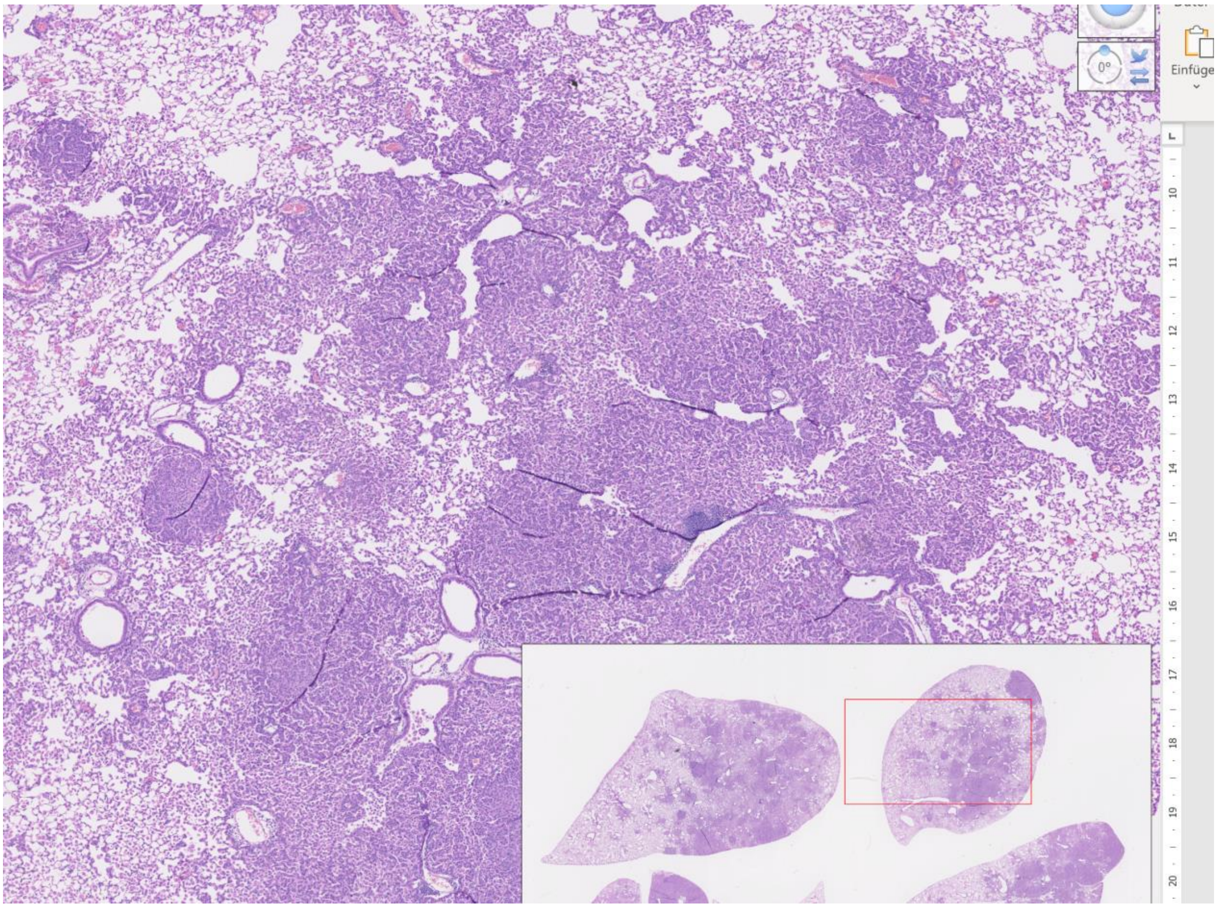

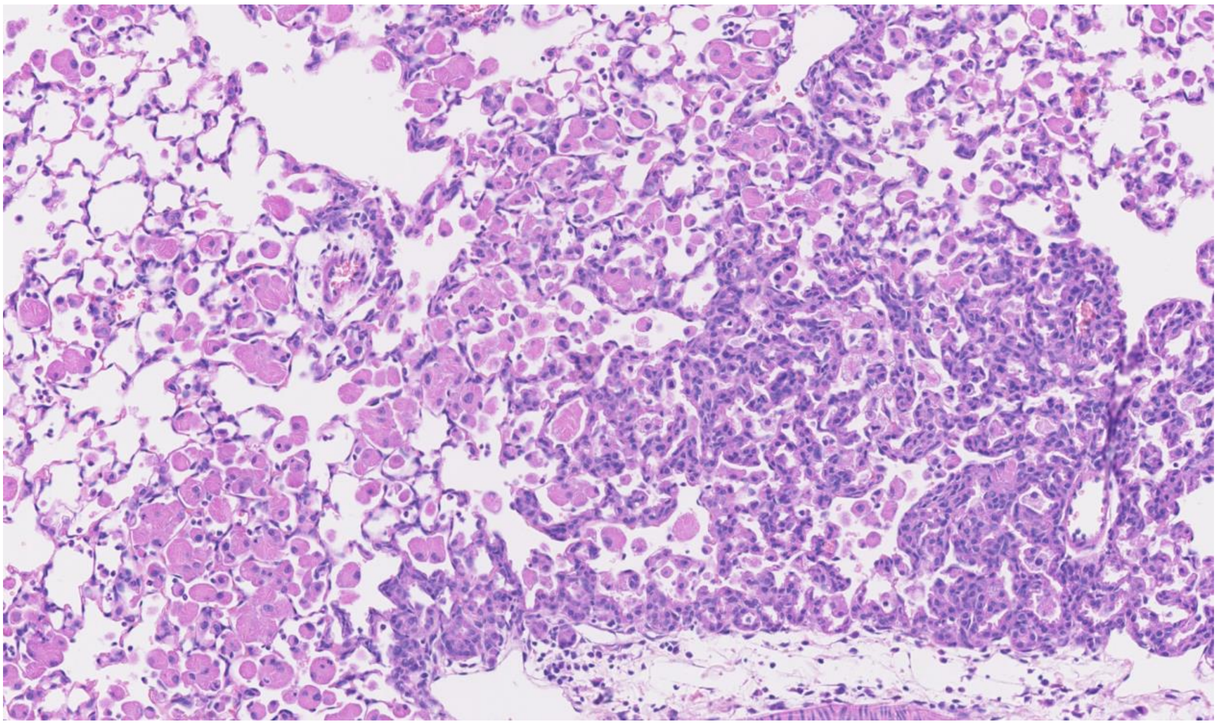

7 H.2209.3ovab6

Multifocal, centrally confluent, mostly lepidic and micropapilarry, some stromaformation with acinar structures and some lymphoplasmacytic infiltrates along vascular and bronchiolar structures.

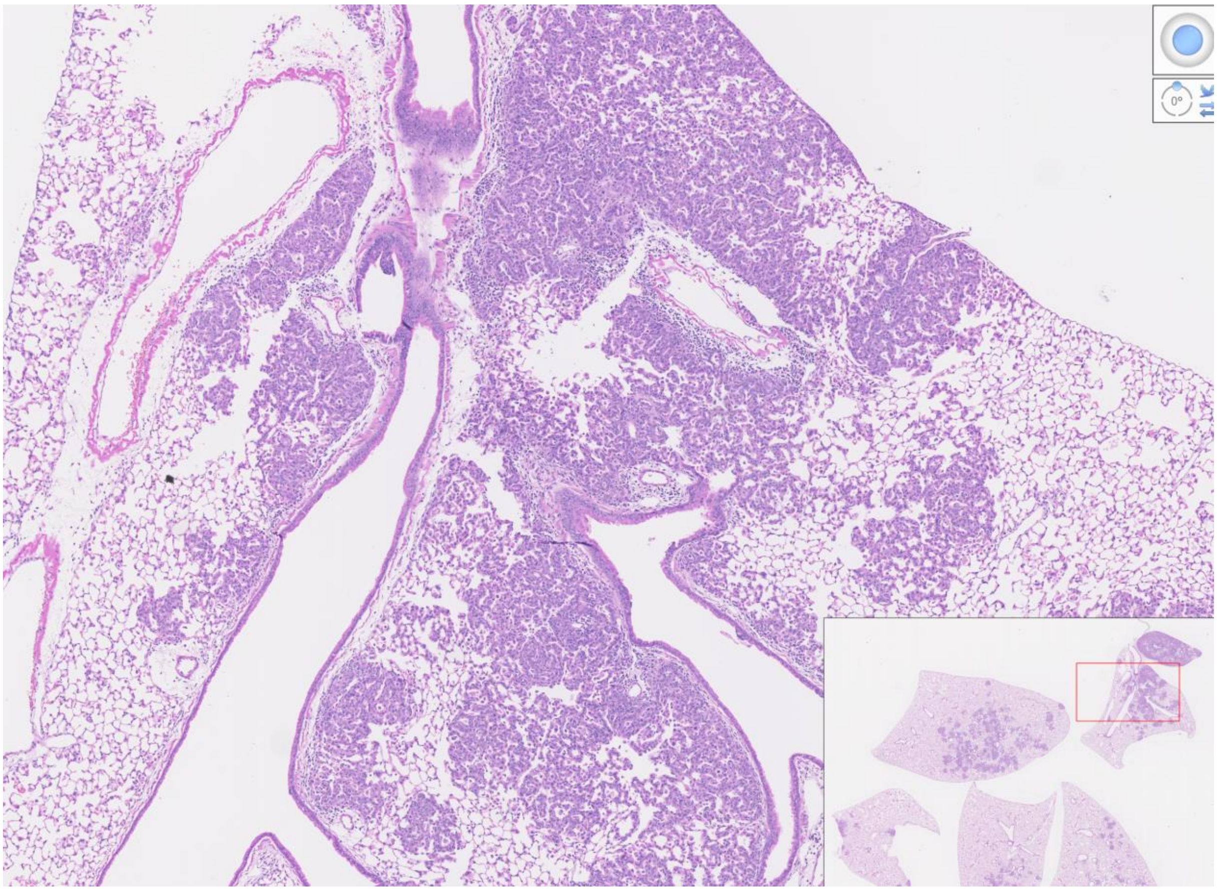

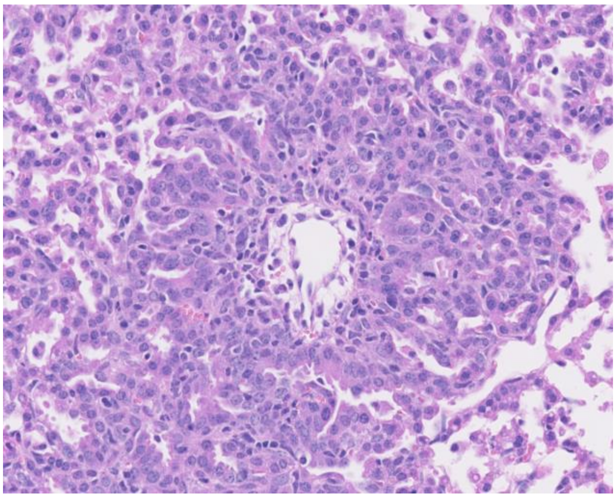

Rosette like growth around vein

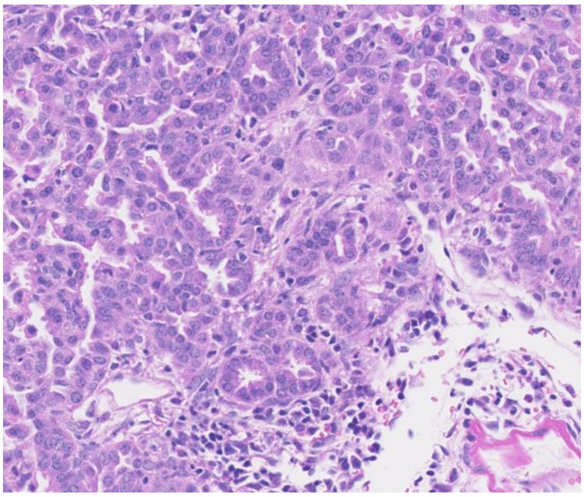

small acinar and little stroma near bronchus

8 HE.2191.ADAM2

Most unusual tumor, Focal nests with mostly lepidic pattern, large infiltrates of single and multinucleated cells with large cytoplasma with cleft like inclusions, mostly aspects of macrophages, to exclude be tumor cells, immunohistochemistry would be helpful (in case of tumorcells it would be giant cell carcinoma.),

Adenocarcinoma with mostly lepidic pattern , with macrophages within alveolar spaces

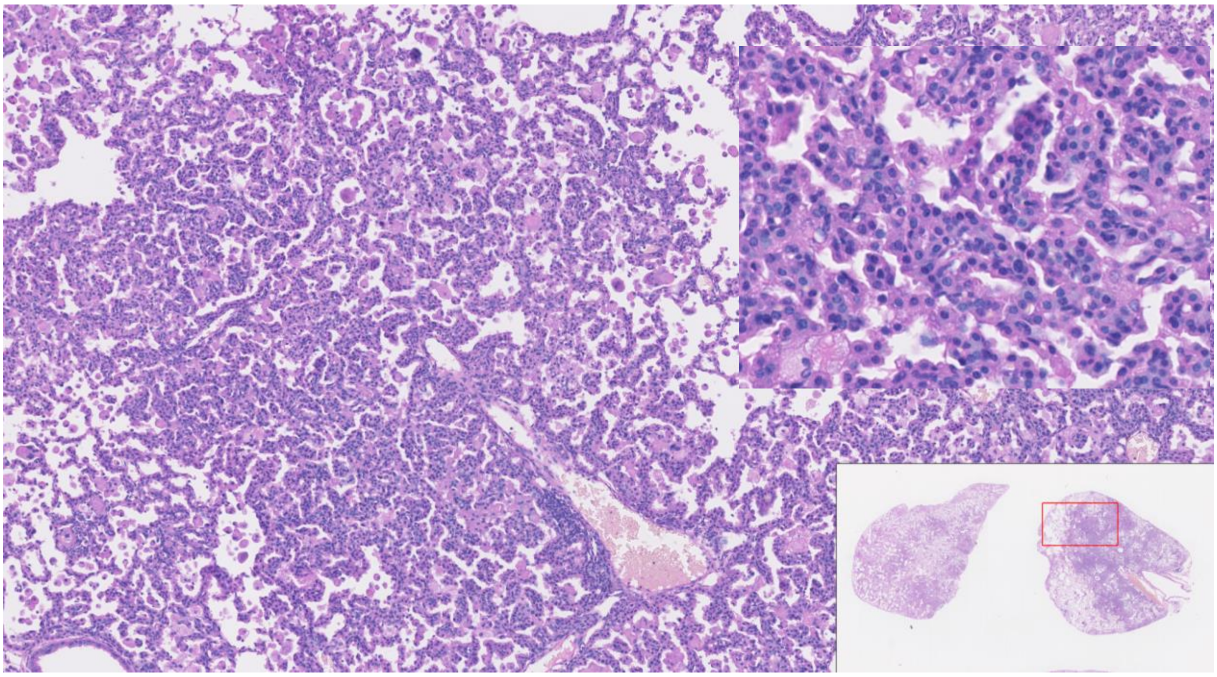

Mostly normal alveoli, immune cell infiltrates. Single cells with lots of cytoplasm sometimes multinucleated, probably not tumorcells (more likely macrophages)

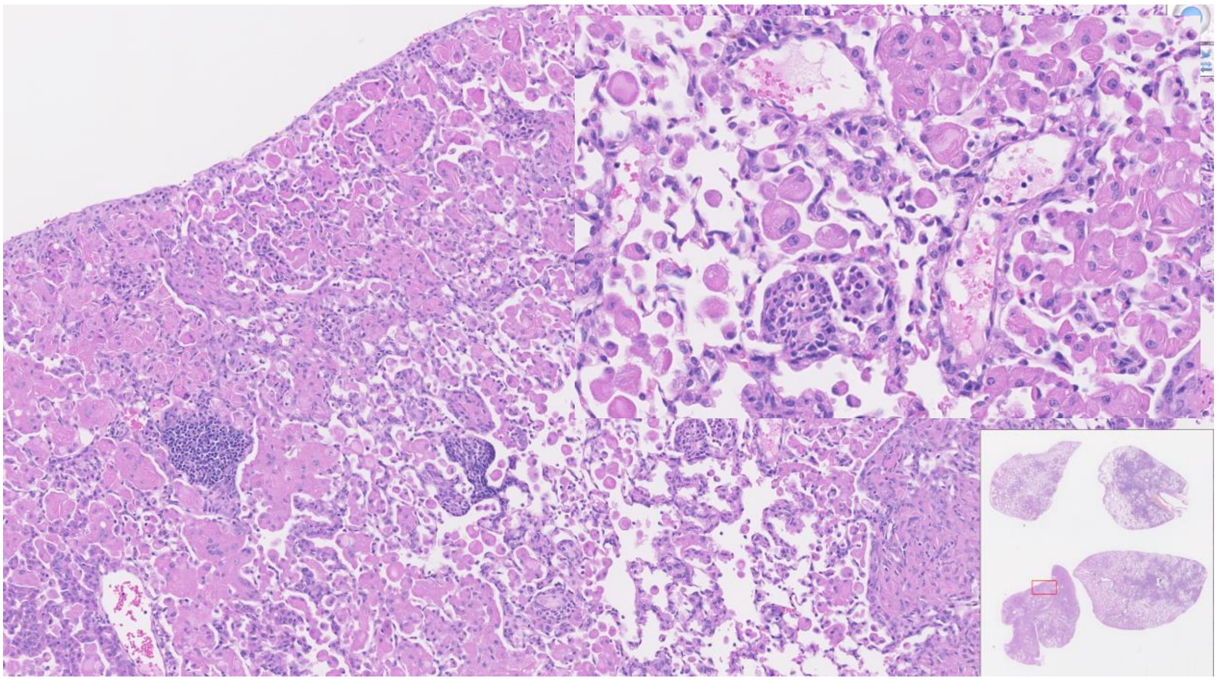

9 HE.1896.ADAM2

Confluent tumor sheets, solid discohesive tumorformations, dense rosette like growth around vessels, immune cell infiltrates along vessels and bronchiole.

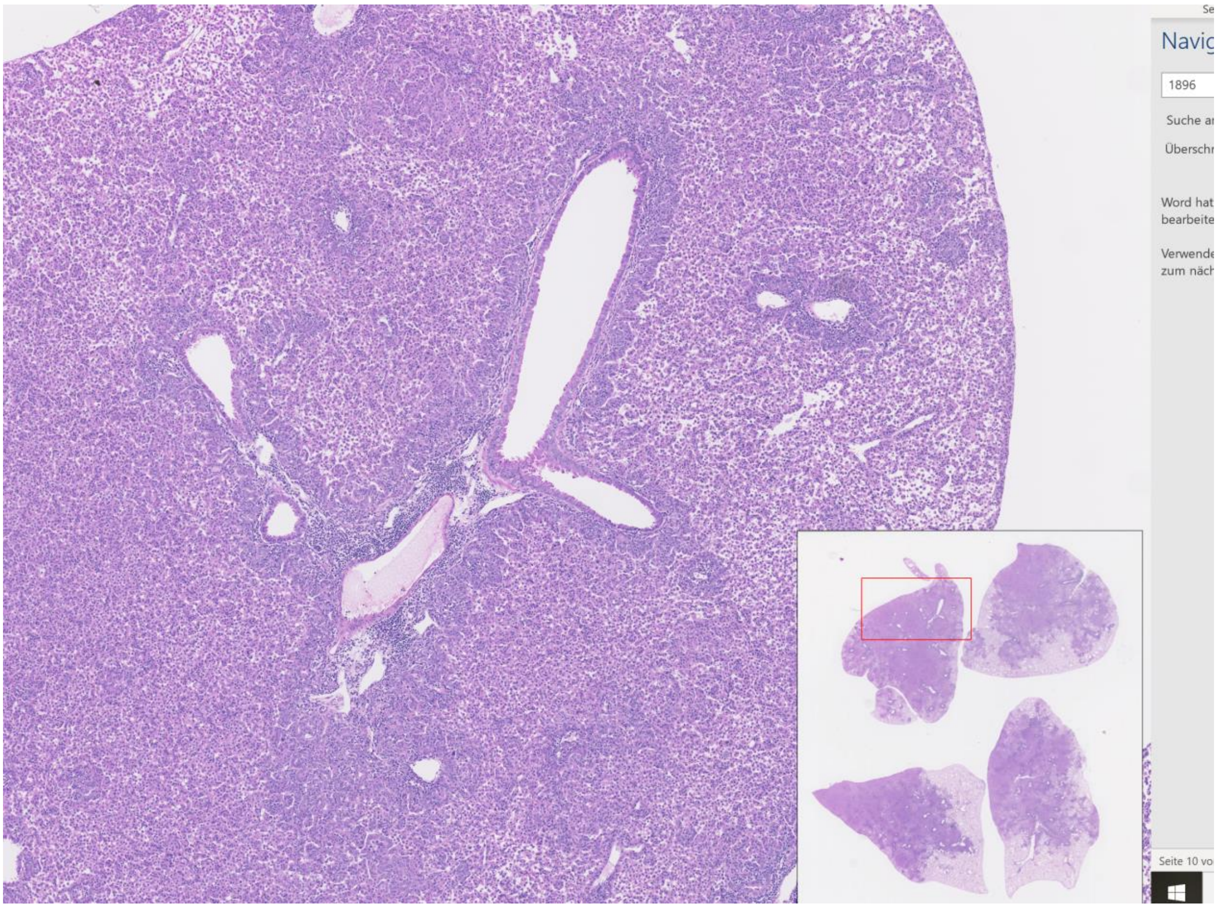

tumorcell nests with peribronchial immunecells, immunecells between tumorcells

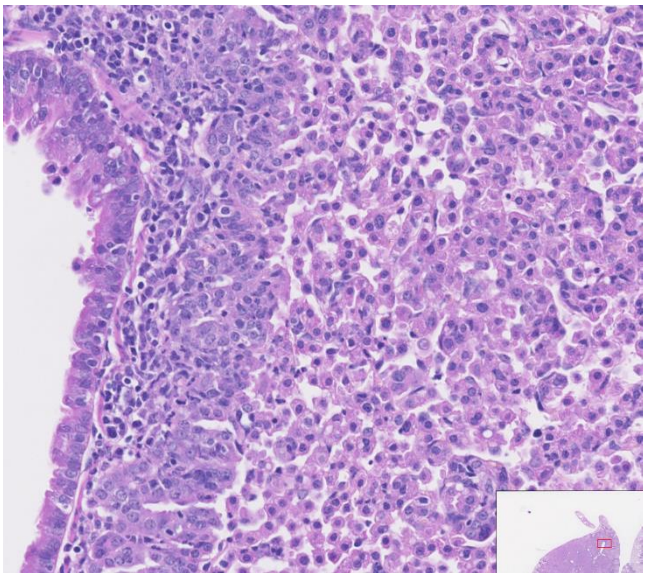

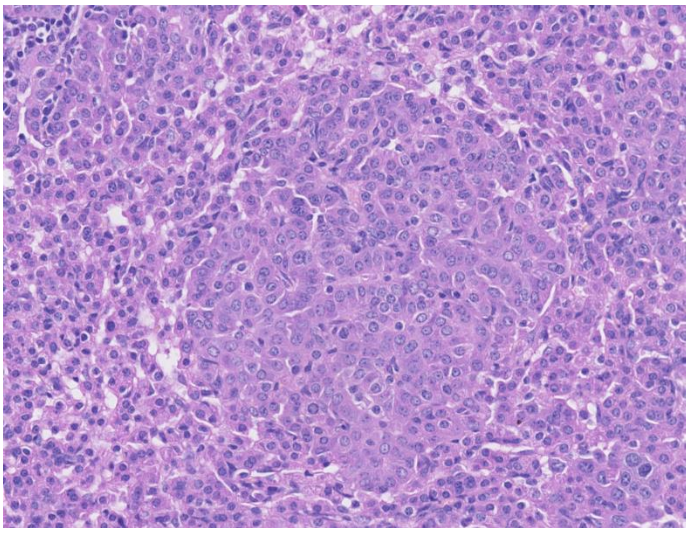

10 HE.1895.ADAM2

Confluent tumor mass with solid nodules unformed solid like tumor sheets, lepidic patterns at the borders, immune cell infiltrates perivascular, peribronchiolar, and some immune cells between tumorcells, some areas with stroma formation and acinar tumorcell clusters.

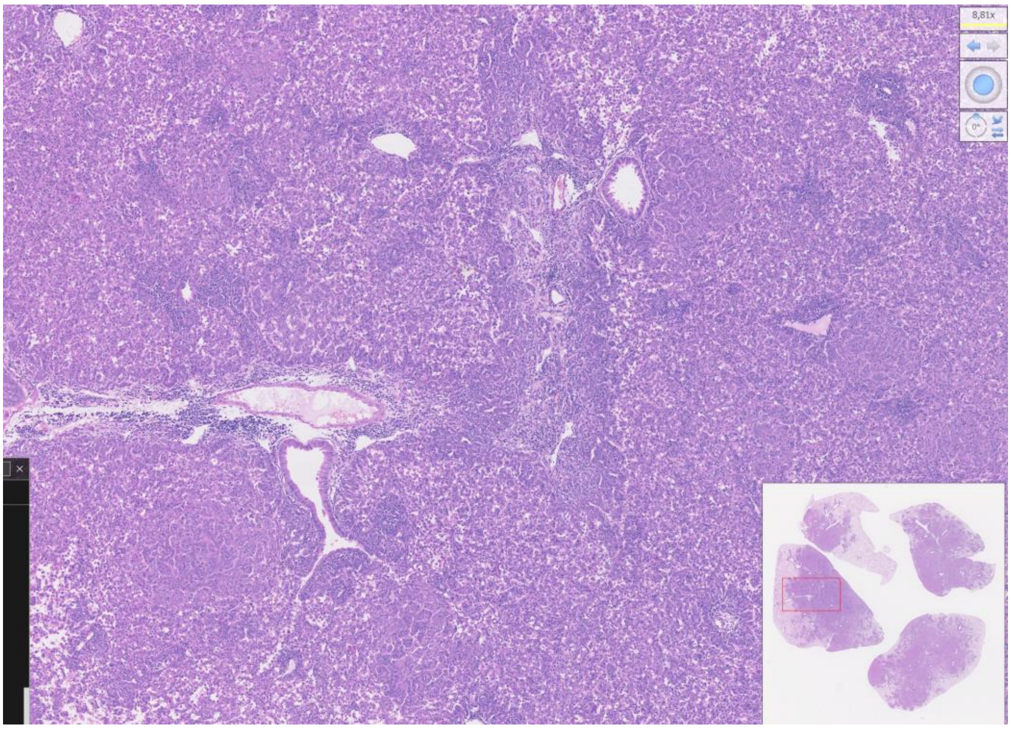

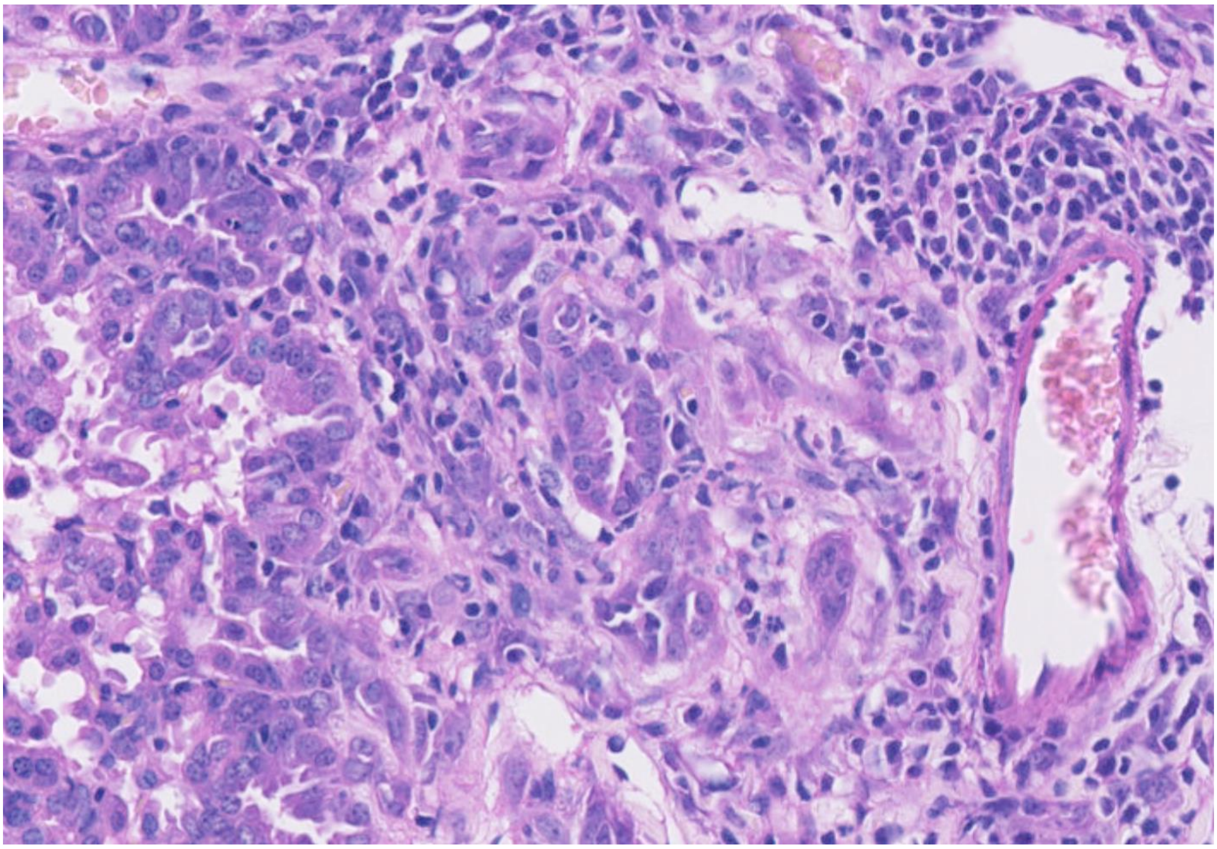

11 HE.1212.ADAM2

Multifocal, partly confluent nodules, solid and micropapillary tumorgrowth patter, peripheral lepicic pattern, tense lymphoplasmacytic infiltrates perivascular, peribronchiolar.

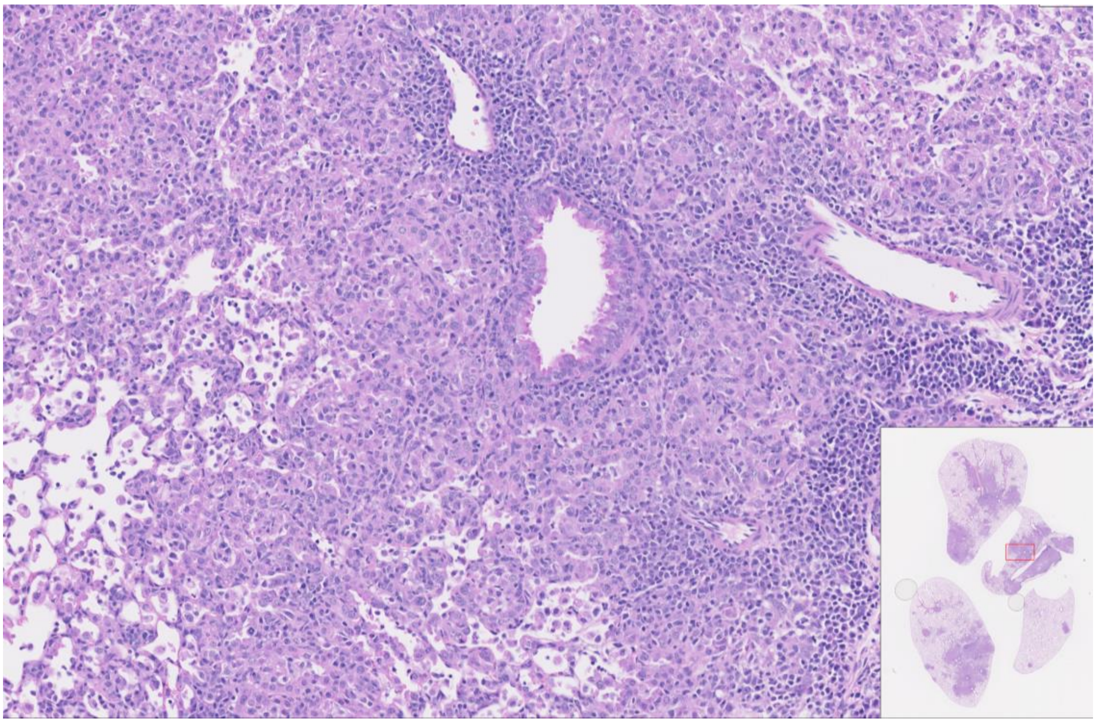

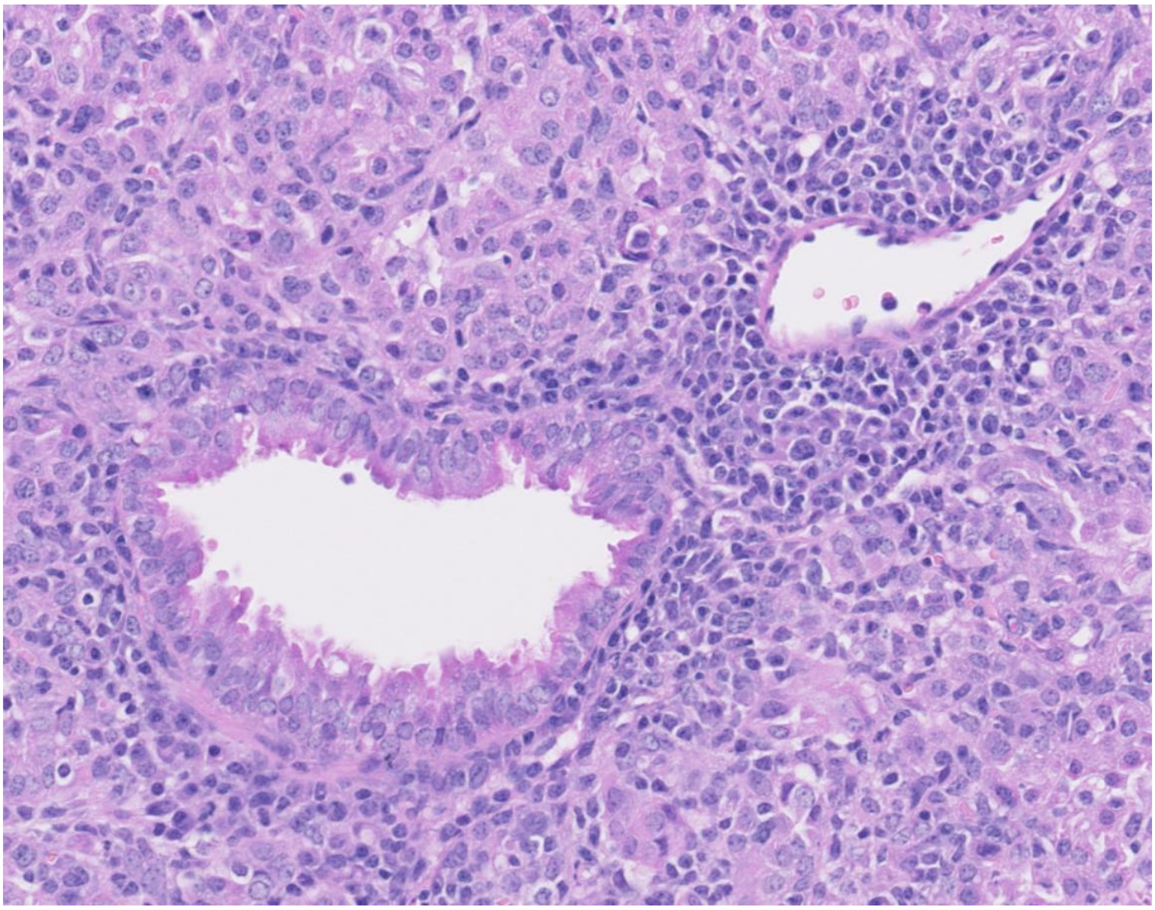

12 HE.1211.ADAM2

Multifocal tumorcellnests, solidlike and micropapillary, bridges of lepidic carcinoma between tumorislands, lymphocyte-nodules at the borders and sometimes within tumorcellclusters., some immunoinfiltrate peribronchial and perivascular.

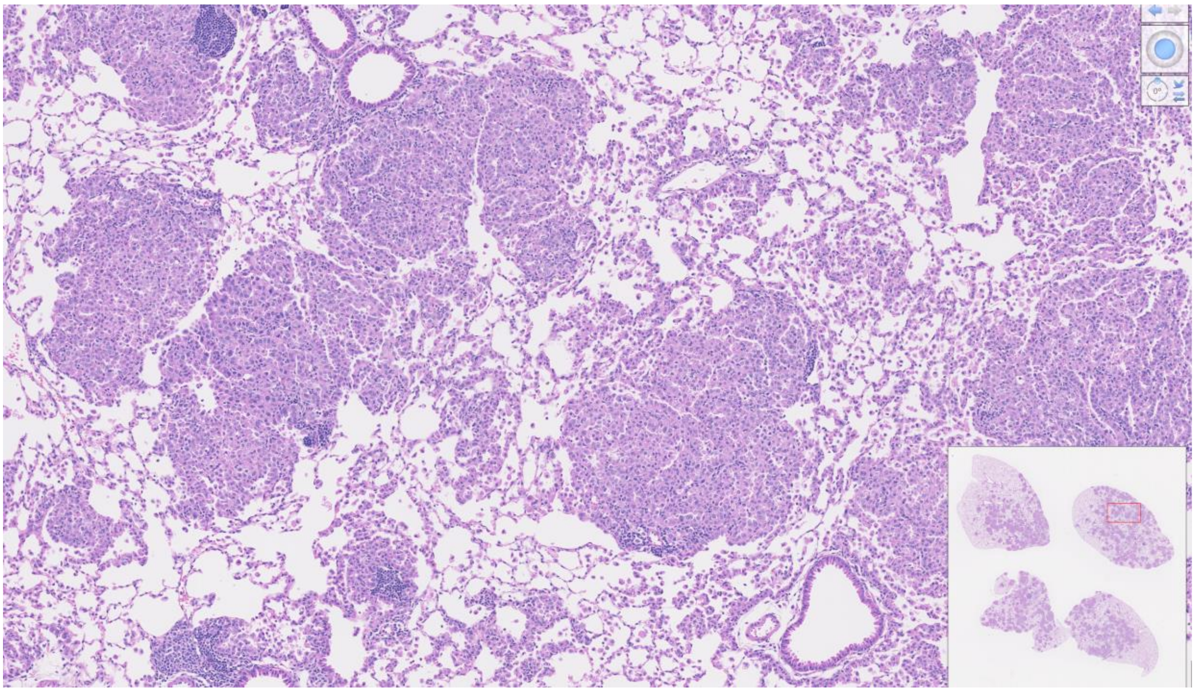

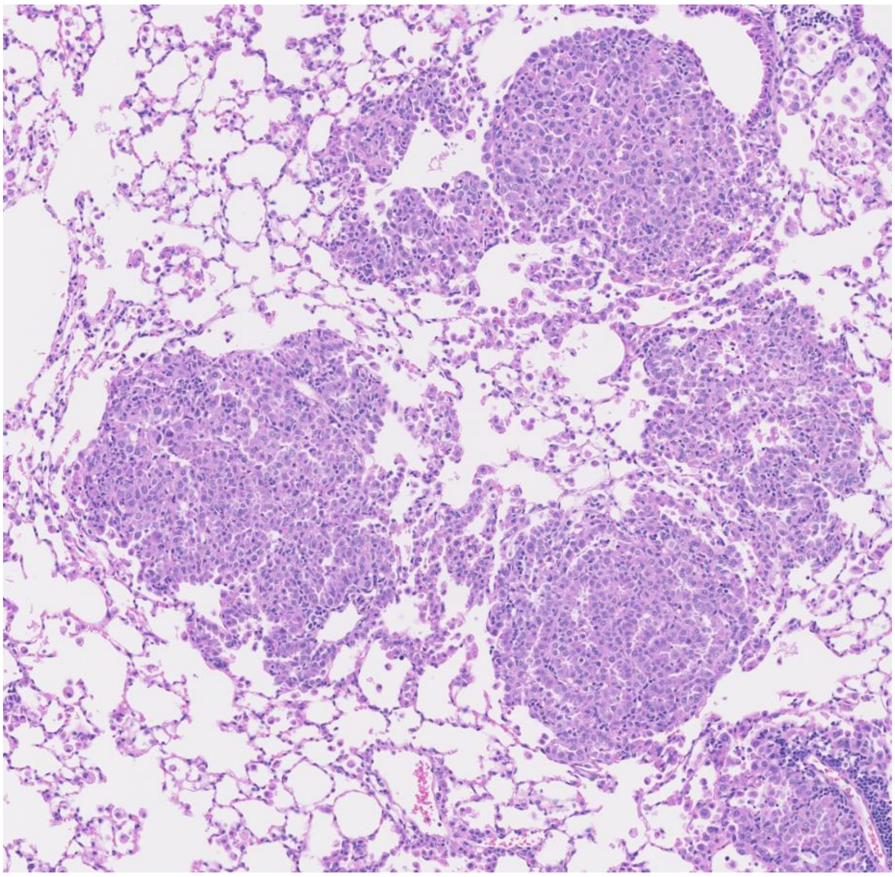

12 HE.1204.ADAM2

Confluent nodules, solid, semisolid and micropapillary structures, some of the single (giant cells, probably macrophages) again.

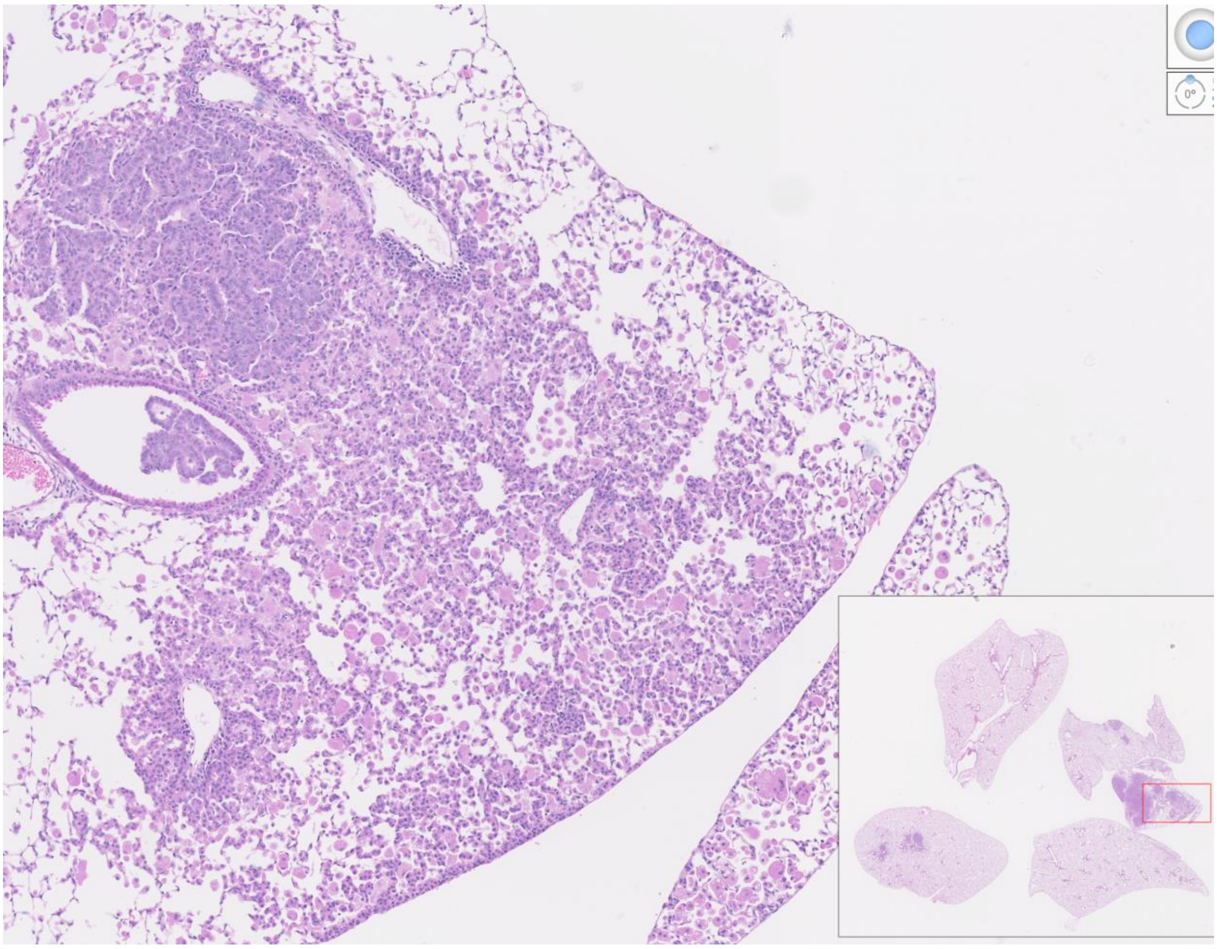

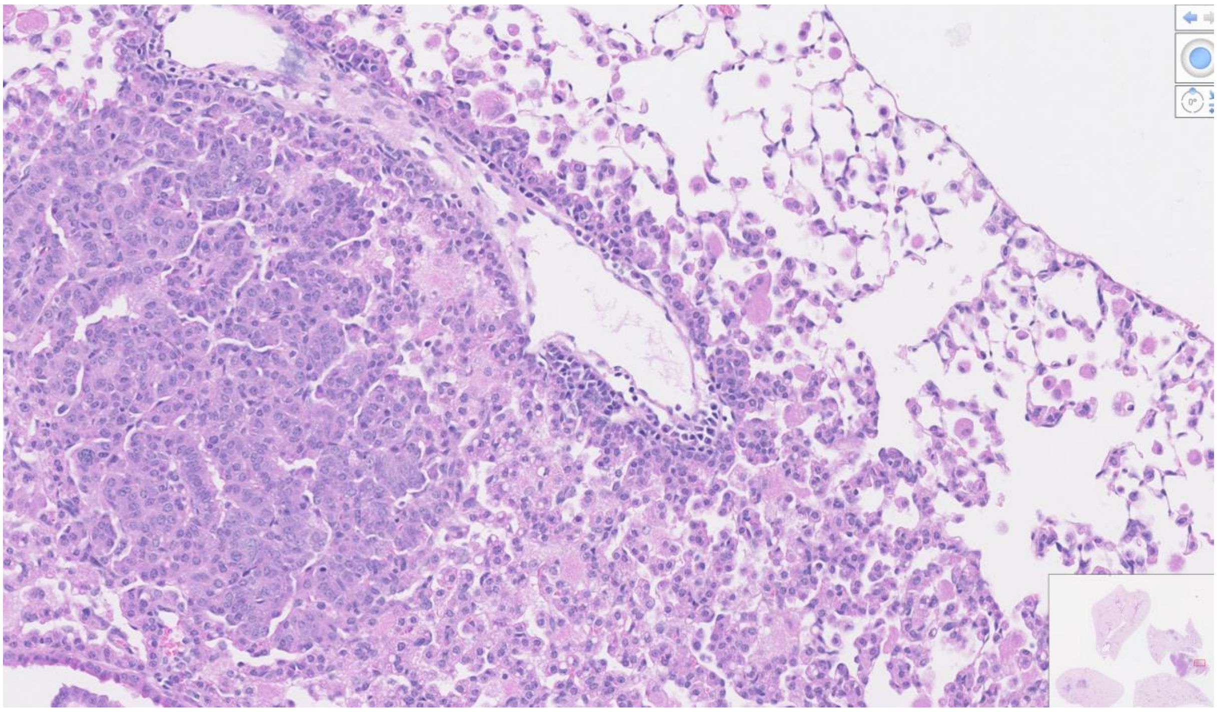

The END
