# Supplementary material for: In vivo CRISPR screens reveal Serpinb9 and Adam2 as regulators of immune therapy response in lung cancer
Source: Nat Commun. 2023 May 31;14:3150. doi: 10.1038/s41467-023-38841-7 (PMC10232477; doi:10.1038/s41467-023-38841-7)
Supplement: Supplementary file 13 — Source Data [file 41467_2023_38841_MOESM13_ESM.zip › Figure 4E.zip!/my_analysis.Gsea.1640146340255_Hallmark/HALLMARK_PI3K_AKT_MTOR_SIGNALING.html]

Details for gene set HALLMARK\_PI3K\_AKT\_MTOR\_SIGNALING[GSEA]

|  || Dataset | December 21, 2021\_for GSEA run\_collapsed\_to\_symbols.GSEA\_B6\_NI.cls #CTLR\_NI\_versus\_ADAM2\_NI |
| Phenotype | GSEA\_B6\_NI.cls#CTLR\_NI\_versus\_ADAM2\_NI |
| Upregulated in class | CTLR\_NI |
| GeneSet | HALLMARK\_PI3K\_AKT\_MTOR\_SIGNALING |
| Enrichment Score (ES) | 0.27831528 |
| Normalized Enrichment Score (NES) | 1.2286693 |
| Nominal p-value | 0.122 |
| FDR q-value | 0.4856212 |
| FWER p-Value | 1.0 |
Table: GSEA Results Summary

  

Fig 1: Enrichment plot: HALLMARK\_PI3K\_AKT\_MTOR\_SIGNALING      
 Profile of the Running ES Score & Positions of GeneSet Members on the Rank Ordered List

  

| SYMBOL | TITLE | RANK IN GENE LIST | RANK METRIC SCORE | RUNNING ES | CORE ENRICHMENT || 1 | FASLG | Fas ligand [Source:HGNC Symbol;Acc:HGNC:11936] | 68 | 1.912 | 0.0515 | Yes |
| 2 | STAT2 | signal transducer and activator of transcription 2 [Source:HGNC Symbol;Acc:HGNC:11363] | 86 | 1.830 | 0.1038 | Yes |
| 3 | LCK | "LCK proto-oncogene, Src family tyrosine kinase [Source:HGNC Symbol;Acc:HGNC:6524]" | 286 | 1.331 | 0.1299 | Yes |
| 4 | IL2RG | interleukin 2 receptor subunit gamma [Source:HGNC Symbol;Acc:HGNC:6010] | 432 | 1.141 | 0.1539 | Yes |
| 5 | CAMK4 | calcium/calmodulin dependent protein kinase IV [Source:HGNC Symbol;Acc:HGNC:1464] | 520 | 1.048 | 0.1790 | Yes |
| 6 | NOD1 | nucleotide binding oligomerization domain containing 1 [Source:HGNC Symbol;Acc:HGNC:16390] | 536 | 1.034 | 0.2082 | Yes |
| 7 | TRIB3 | tribbles pseudokinase 3 [Source:HGNC Symbol;Acc:HGNC:16228] | 901 | 0.841 | 0.2094 | Yes |
| 8 | GNGT1 | G protein subunit gamma transducin 1 [Source:HGNC Symbol;Acc:HGNC:4411] | 1028 | 0.801 | 0.2247 | Yes |
| 9 | GNA14 | G protein subunit alpha 14 [Source:HGNC Symbol;Acc:HGNC:4382] | 1041 | 0.794 | 0.2471 | Yes |
| 10 | SFN | stratifin [Source:HGNC Symbol;Acc:HGNC:10773] | 1200 | 0.732 | 0.2583 | Yes |
| 11 | TIAM1 | TIAM Rac1 associated GEF 1 [Source:HGNC Symbol;Acc:HGNC:11805] | 1317 | 0.689 | 0.2710 | Yes |
| 12 | VAV3 | vav guanine nucleotide exchange factor 3 [Source:HGNC Symbol;Acc:HGNC:12659] | 1490 | 0.630 | 0.2783 | Yes |
| 13 | SLA | Src like adaptor [Source:HGNC Symbol;Acc:HGNC:10902] | 1841 | 0.535 | 0.2715 | No |
| 14 | PIKFYVE | "phosphoinositide kinase, FYVE-type zinc finger containing [Source:HGNC Symbol;Acc:HGNC:23785]" | 2270 | 0.473 | 0.2578 | No |
| 15 | ACTR3 | actin related protein 3 [Source:HGNC Symbol;Acc:HGNC:170] | 2582 | 0.436 | 0.2505 | No |
| 16 | PRKAA2 | protein kinase AMP-activated catalytic subunit alpha 2 [Source:HGNC Symbol;Acc:HGNC:9377] | 2700 | 0.426 | 0.2555 | No |
| 17 | FGF22 | fibroblast growth factor 22 [Source:HGNC Symbol;Acc:HGNC:3679] | 3083 | 0.390 | 0.2423 | No |
| 18 | MAPK10 | mitogen-activated protein kinase 10 [Source:HGNC Symbol;Acc:HGNC:6872] | 3088 | 0.390 | 0.2534 | No |
| 19 | MYD88 | MYD88 innate immune signal transduction adaptor [Source:HGNC Symbol;Acc:HGNC:7562] | 3202 | 0.373 | 0.2571 | No |
| 20 | ACTR2 | actin related protein 2 [Source:HGNC Symbol;Acc:HGNC:169] | 3441 | 0.336 | 0.2516 | No |
| 21 | RPS6KA1 | ribosomal protein S6 kinase A1 [Source:HGNC Symbol;Acc:HGNC:10430] | 3617 | 0.313 | 0.2495 | No |
| 22 | CAB39 | calcium binding protein 39 [Source:HGNC Symbol;Acc:HGNC:20292] | 3763 | 0.294 | 0.2488 | No |
| 23 | GSK3B | glycogen synthase kinase 3 beta [Source:HGNC Symbol;Acc:HGNC:4617] | 3768 | 0.294 | 0.2571 | No |
| 24 | CAB39L | calcium binding protein 39 like [Source:HGNC Symbol;Acc:HGNC:20290] | 3784 | 0.292 | 0.2647 | No |
| 25 | IRAK4 | interleukin 1 receptor associated kinase 4 [Source:HGNC Symbol;Acc:HGNC:17967] | 4046 | 0.260 | 0.2555 | No |
| 26 | TBK1 | TANK binding kinase 1 [Source:HGNC Symbol;Acc:HGNC:11584] | 4121 | 0.252 | 0.2581 | No |
| 27 | CDK2 | cyclin dependent kinase 2 [Source:HGNC Symbol;Acc:HGNC:1771] | 4345 | 0.228 | 0.2504 | No |
| 28 | MAPK8 | mitogen-activated protein kinase 8 [Source:HGNC Symbol;Acc:HGNC:6881] | 4460 | 0.217 | 0.2494 | No |
| 29 | DDIT3 | DNA damage inducible transcript 3 [Source:HGNC Symbol;Acc:HGNC:2726] | 4509 | 0.212 | 0.2525 | No |
| 30 | PRKCB | protein kinase C beta [Source:HGNC Symbol;Acc:HGNC:9395] | 4572 | 0.207 | 0.2546 | No |
| 31 | DAPP1 | dual adaptor of phosphotyrosine and 3-phosphoinositides 1 [Source:HGNC Symbol;Acc:HGNC:16500] | 4588 | 0.206 | 0.2597 | No |
| 32 | PLCB1 | phospholipase C beta 1 [Source:HGNC Symbol;Acc:HGNC:15917] | 4612 | 0.204 | 0.2641 | No |
| 33 | RIPK1 | receptor interacting serine/threonine kinase 1 [Source:HGNC Symbol;Acc:HGNC:10019] | 4756 | 0.192 | 0.2606 | No |
| 34 | MKNK1 | MAPK interacting serine/threonine kinase 1 [Source:HGNC Symbol;Acc:HGNC:7110] | 4823 | 0.186 | 0.2617 | No |
| 35 | SMAD2 | SMAD family member 2 [Source:HGNC Symbol;Acc:HGNC:6768] | 4875 | 0.181 | 0.2637 | No |
| 36 | CDKN1B | cyclin dependent kinase inhibitor 1B [Source:HGNC Symbol;Acc:HGNC:1785] | 4931 | 0.175 | 0.2653 | No |
| 37 | ATF1 | activating transcription factor 1 [Source:HGNC Symbol;Acc:HGNC:783] | 4935 | 0.175 | 0.2702 | No |
| 38 | CLTC | clathrin heavy chain [Source:HGNC Symbol;Acc:HGNC:2092] | 5312 | 0.141 | 0.2502 | No |
| 39 | GRB2 | growth factor receptor bound protein 2 [Source:HGNC Symbol;Acc:HGNC:4566] | 5321 | 0.141 | 0.2538 | No |
| 40 | UBE2D3 | ubiquitin conjugating enzyme E2 D3 [Source:HGNC Symbol;Acc:HGNC:12476] | 5615 | 0.118 | 0.2384 | No |
| 41 | MAPK1 | mitogen-activated protein kinase 1 [Source:HGNC Symbol;Acc:HGNC:6871] | 5638 | 0.116 | 0.2404 | No |
| 42 | NFKBIB | NFKB inhibitor beta [Source:HGNC Symbol;Acc:HGNC:7798] | 6288 | 0.071 | 0.2007 | No |
| 43 | CDK1 | cyclin dependent kinase 1 [Source:HGNC Symbol;Acc:HGNC:1722] | 6401 | 0.062 | 0.1954 | No |
| 44 | MKNK2 | MAPK interacting serine/threonine kinase 2 [Source:HGNC Symbol;Acc:HGNC:7111] | 6739 | 0.041 | 0.1749 | No |
| 45 | ACACA | acetyl-CoA carboxylase alpha [Source:HGNC Symbol;Acc:HGNC:84] | 6836 | 0.034 | 0.1697 | No |
| 46 | RALB | RAS like proto-oncogene B [Source:HGNC Symbol;Acc:HGNC:9840] | 6926 | 0.027 | 0.1648 | No |
| 47 | MAPK9 | mitogen-activated protein kinase 9 [Source:HGNC Symbol;Acc:HGNC:6886] | 7127 | 0.017 | 0.1525 | No |
| 48 | TNFRSF1A | TNF receptor superfamily member 1A [Source:HGNC Symbol;Acc:HGNC:11916] | 7339 | 0.004 | 0.1390 | No |
| 49 | NCK1 | NCK adaptor protein 1 [Source:HGNC Symbol;Acc:HGNC:7664] | 7446 | -0.001 | 0.1322 | No |
| 50 | ARPC3 | actin related protein 2/3 complex subunit 3 [Source:HGNC Symbol;Acc:HGNC:706] | 7577 | -0.008 | 0.1241 | No |
| 51 | PFN1 | profilin 1 [Source:HGNC Symbol;Acc:HGNC:8881] | 7810 | -0.019 | 0.1098 | No |
| 52 | GRK2 | G protein-coupled receptor kinase 2 [Source:HGNC Symbol;Acc:HGNC:289] | 7984 | -0.028 | 0.0995 | No |
| 53 | RPS6KA3 | ribosomal protein S6 kinase A3 [Source:HGNC Symbol;Acc:HGNC:10432] | 8182 | -0.039 | 0.0880 | No |
| 54 | MAP2K3 | mitogen-activated protein kinase kinase 3 [Source:HGNC Symbol;Acc:HGNC:6843] | 8186 | -0.039 | 0.0889 | No |
| 55 | PTPN11 | protein tyrosine phosphatase non-receptor type 11 [Source:HGNC Symbol;Acc:HGNC:9644] | 8451 | -0.053 | 0.0735 | No |
| 56 | EIF4E | eukaryotic translation initiation factor 4E [Source:HGNC Symbol;Acc:HGNC:3287] | 8798 | -0.073 | 0.0534 | No |
| 57 | RAC1 | Rac family small GTPase 1 [Source:HGNC Symbol;Acc:HGNC:9801] | 9083 | -0.087 | 0.0377 | No |
| 58 | YWHAB | tyrosine 3-monooxygenase/tryptophan 5-monooxygenase activation protein beta [Source:HGNC Symbol;Acc:HGNC:12849] | 9098 | -0.088 | 0.0393 | No |
| 59 | UBE2N | ubiquitin conjugating enzyme E2 N [Source:HGNC Symbol;Acc:HGNC:12492] | 9173 | -0.092 | 0.0373 | No |
| 60 | AP2M1 | adaptor related protein complex 2 subunit mu 1 [Source:HGNC Symbol;Acc:HGNC:564] | 9180 | -0.092 | 0.0396 | No |
| 61 | TRAF2 | TNF receptor associated factor 2 [Source:HGNC Symbol;Acc:HGNC:12032] | 9218 | -0.094 | 0.0399 | No |
| 62 | HSP90B1 | heat shock protein 90 beta family member 1 [Source:HGNC Symbol;Acc:HGNC:12028] | 9406 | -0.105 | 0.0310 | No |
| 63 | PPP2R1B | protein phosphatase 2 scaffold subunit Abeta [Source:HGNC Symbol;Acc:HGNC:9303] | 9437 | -0.106 | 0.0322 | No |
| 64 | EGFR | epidermal growth factor receptor [Source:HGNC Symbol;Acc:HGNC:3236] | 9513 | -0.111 | 0.0306 | No |
| 65 | RAF1 | "Raf-1 proto-oncogene, serine/threonine kinase [Source:HGNC Symbol;Acc:HGNC:9829]" | 9515 | -0.112 | 0.0338 | No |
| 66 | ADCY2 | adenylate cyclase 2 [Source:HGNC Symbol;Acc:HGNC:233] | 9591 | -0.117 | 0.0324 | No |
| 67 | CXCR4 | C-X-C motif chemokine receptor 4 [Source:HGNC Symbol;Acc:HGNC:2561] | 9672 | -0.123 | 0.0308 | No |
| 68 | CFL1 | cofilin 1 [Source:HGNC Symbol;Acc:HGNC:1874] | 9807 | -0.131 | 0.0261 | No |
| 69 | ITPR2 | "inositol 1,4,5-trisphosphate receptor type 2 [Source:HGNC Symbol;Acc:HGNC:6181]" | 10181 | -0.153 | 0.0065 | No |
| 70 | PRKAG1 | protein kinase AMP-activated non-catalytic subunit gamma 1 [Source:HGNC Symbol;Acc:HGNC:9385] | 10201 | -0.154 | 0.0098 | No |
| 71 | HRAS | "HRas proto-oncogene, GTPase [Source:HGNC Symbol;Acc:HGNC:5173]" | 10331 | -0.161 | 0.0062 | No |
| 72 | PLA2G12A | phospholipase A2 group XIIA [Source:HGNC Symbol;Acc:HGNC:18554] | 10336 | -0.161 | 0.0107 | No |
| 73 | PIK3R3 | phosphoinositide-3-kinase regulatory subunit 3 [Source:HGNC Symbol;Acc:HGNC:8981] | 10512 | -0.172 | 0.0045 | No |
| 74 | RPTOR | regulatory associated protein of MTOR complex 1 [Source:HGNC Symbol;Acc:HGNC:30287] | 10761 | -0.186 | -0.0060 | No |
| 75 | PITX2 | paired like homeodomain 2 [Source:HGNC Symbol;Acc:HGNC:9005] | 10960 | -0.198 | -0.0130 | No |
| 76 | PTEN | phosphatase and tensin homolog [Source:HGNC Symbol;Acc:HGNC:9588] | 11136 | -0.208 | -0.0181 | No |
| 77 | CSNK2B | casein kinase 2 beta [Source:HGNC Symbol;Acc:HGNC:2460] | 11149 | -0.209 | -0.0128 | No |
| 78 | PAK4 | p21 (RAC1) activated kinase 4 [Source:HGNC Symbol;Acc:HGNC:16059] | 11300 | -0.217 | -0.0161 | No |
| 79 | MAP3K7 | mitogen-activated protein kinase kinase kinase 7 [Source:HGNC Symbol;Acc:HGNC:6859] | 11555 | -0.232 | -0.0256 | No |
| 80 | AKT1 | AKT serine/threonine kinase 1 [Source:HGNC Symbol;Acc:HGNC:391] | 11570 | -0.233 | -0.0197 | No |
| 81 | E2F1 | E2F transcription factor 1 [Source:HGNC Symbol;Acc:HGNC:3113] | 11698 | -0.241 | -0.0208 | No |
| 82 | MAPKAP1 | MAPK associated protein 1 [Source:HGNC Symbol;Acc:HGNC:18752] | 11841 | -0.250 | -0.0226 | No |
| 83 | DUSP3 | dual specificity phosphatase 3 [Source:HGNC Symbol;Acc:HGNC:3069] | 11852 | -0.251 | -0.0160 | No |
| 84 | PRKAR2A | protein kinase cAMP-dependent type II regulatory subunit alpha [Source:HGNC Symbol;Acc:HGNC:9391] | 11992 | -0.260 | -0.0173 | No |
| 85 | ARF1 | ADP ribosylation factor 1 [Source:HGNC Symbol;Acc:HGNC:652] | 12056 | -0.264 | -0.0136 | No |
| 86 | ECSIT | ECSIT signaling integrator [Source:HGNC Symbol;Acc:HGNC:29548] | 12145 | -0.270 | -0.0114 | No |
| 87 | PIN1 | "peptidylprolyl cis/trans isomerase, NIMA-interacting 1 [Source:HGNC Symbol;Acc:HGNC:8988]" | 12154 | -0.270 | -0.0040 | No |
| 88 | PPP1CA | protein phosphatase 1 catalytic subunit alpha [Source:HGNC Symbol;Acc:HGNC:9281] | 12176 | -0.272 | 0.0026 | No |
| 89 | FGF17 | fibroblast growth factor 17 [Source:HGNC Symbol;Acc:HGNC:3673] | 12301 | -0.281 | 0.0028 | No |
| 90 | ARHGDIA | Rho GDP dissociation inhibitor alpha [Source:HGNC Symbol;Acc:HGNC:678] | 12426 | -0.291 | 0.0033 | No |
| 91 | CALR | calreticulin [Source:HGNC Symbol;Acc:HGNC:1455] | 12856 | -0.324 | -0.0147 | No |
| 92 | PLCG1 | phospholipase C gamma 1 [Source:HGNC Symbol;Acc:HGNC:9065] | 13040 | -0.342 | -0.0165 | No |
| 93 | THEM4 | thioesterase superfamily member 4 [Source:HGNC Symbol;Acc:HGNC:17947] | 13108 | -0.348 | -0.0107 | No |
| 94 | AKT1S1 | AKT1 substrate 1 [Source:HGNC Symbol;Acc:HGNC:28426] | 13462 | -0.387 | -0.0220 | No |
| 95 | CDK4 | cyclin dependent kinase 4 [Source:HGNC Symbol;Acc:HGNC:1773] | 13588 | -0.403 | -0.0183 | No |
| 96 | SQSTM1 | sequestosome 1 [Source:HGNC Symbol;Acc:HGNC:11280] | 13604 | -0.406 | -0.0074 | No |
| 97 | IL4 | interleukin 4 [Source:HGNC Symbol;Acc:HGNC:6014] | 13785 | -0.416 | -0.0068 | No |
| 98 | TSC2 | TSC complex subunit 2 [Source:HGNC Symbol;Acc:HGNC:12363] | 14018 | -0.435 | -0.0090 | No |
| 99 | RIT1 | Ras like without CAAX 1 [Source:HGNC Symbol;Acc:HGNC:10023] | 14133 | -0.454 | -0.0031 | No |
| 100 | PDK1 | pyruvate dehydrogenase kinase 1 [Source:HGNC Symbol;Acc:HGNC:8809] | 14661 | -0.544 | -0.0211 | No |
| 101 | SLC2A1 | solute carrier family 2 member 1 [Source:HGNC Symbol;Acc:HGNC:11005] | 14992 | -0.661 | -0.0229 | No |
| 102 | NGF | nerve growth factor [Source:HGNC Symbol;Acc:HGNC:7808] | 15003 | -0.664 | -0.0042 | No |
| 103 | MAP2K6 | mitogen-activated protein kinase kinase 6 [Source:HGNC Symbol;Acc:HGNC:6846] | 15022 | -0.673 | 0.0143 | No |
| 104 | CDKN1A | cyclin dependent kinase inhibitor 1A [Source:HGNC Symbol;Acc:HGNC:1784] | 15428 | -0.929 | 0.0154 | No |
Table: GSEA details [plain text format]

  

Fig 2: HALLMARK\_PI3K\_AKT\_MTOR\_SIGNALING      
 Blue-Pink O' Gram in the Space of the Analyzed GeneSet

  

Fig 3: HALLMARK\_PI3K\_AKT\_MTOR\_SIGNALING: Random ES distribution      
 Gene set null distribution of ES for **HALLMARK\_PI3K\_AKT\_MTOR\_SIGNALING**
