# Supplementary material for: In vivo CRISPR screens reveal Serpinb9 and Adam2 as regulators of immune therapy response in lung cancer
Source: Nat Commun. 2023 May 31;14:3150. doi: 10.1038/s41467-023-38841-7 (PMC10232477; doi:10.1038/s41467-023-38841-7)
Supplement: Supplementary file 13 — Source Data [file 41467_2023_38841_MOESM13_ESM.zip › Figure 4E.zip!/my_analysis.Gsea.1640146340255_Hallmark/HALLMARK_KRAS_SIGNALING_UP.html]

Details for gene set HALLMARK\_KRAS\_SIGNALING\_UP[GSEA]

|  || Dataset | December 21, 2021\_for GSEA run\_collapsed\_to\_symbols.GSEA\_B6\_NI.cls #CTLR\_NI\_versus\_ADAM2\_NI |
| Phenotype | GSEA\_B6\_NI.cls#CTLR\_NI\_versus\_ADAM2\_NI |
| Upregulated in class | CTLR\_NI |
| GeneSet | HALLMARK\_KRAS\_SIGNALING\_UP |
| Enrichment Score (ES) | 0.42641264 |
| Normalized Enrichment Score (NES) | 1.3781575 |
| Nominal p-value | 0.0 |
| FDR q-value | 0.2907768 |
| FWER p-Value | 0.802 |
Table: GSEA Results Summary

  

Fig 1: Enrichment plot: HALLMARK\_KRAS\_SIGNALING\_UP      
 Profile of the Running ES Score & Positions of GeneSet Members on the Rank Ordered List

  

| SYMBOL | TITLE | RANK IN GENE LIST | RANK METRIC SCORE | RUNNING ES | CORE ENRICHMENT || 1 | MMP10 | matrix metallopeptidase 10 [Source:HGNC Symbol;Acc:HGNC:7156] | 48 | 2.057 | 0.0188 | Yes |
| 2 | SERPINA3 | serpin family A member 3 [Source:HGNC Symbol;Acc:HGNC:16] | 50 | 2.046 | 0.0405 | Yes |
| 3 | CLEC4A | C-type lectin domain family 4 member A [Source:HGNC Symbol;Acc:HGNC:13257] | 53 | 2.001 | 0.0616 | Yes |
| 4 | PDCD1LG2 | programmed cell death 1 ligand 2 [Source:HGNC Symbol;Acc:HGNC:18731] | 72 | 1.900 | 0.0806 | Yes |
| 5 | CXCL10 | C-X-C motif chemokine ligand 10 [Source:HGNC Symbol;Acc:HGNC:10637] | 142 | 1.666 | 0.0939 | Yes |
| 6 | CFB | complement factor B [Source:HGNC Symbol;Acc:HGNC:1037] | 245 | 1.387 | 0.1021 | Yes |
| 7 | MAP7 | microtubule associated protein 7 [Source:HGNC Symbol;Acc:HGNC:6869] | 282 | 1.335 | 0.1139 | Yes |
| 8 | HSD11B1 | hydroxysteroid 11-beta dehydrogenase 1 [Source:HGNC Symbol;Acc:HGNC:5208] | 291 | 1.319 | 0.1274 | Yes |
| 9 | APOD | apolipoprotein D [Source:HGNC Symbol;Acc:HGNC:612] | 293 | 1.314 | 0.1413 | Yes |
| 10 | SATB1 | SATB homeobox 1 [Source:HGNC Symbol;Acc:HGNC:10541] | 300 | 1.300 | 0.1548 | Yes |
| 11 | IL1B | interleukin 1 beta [Source:HGNC Symbol;Acc:HGNC:5992] | 303 | 1.298 | 0.1684 | Yes |
| 12 | PRDM1 | PR/SET domain 1 [Source:HGNC Symbol;Acc:HGNC:9346] | 340 | 1.253 | 0.1794 | Yes |
| 13 | BIRC3 | baculoviral IAP repeat containing 3 [Source:HGNC Symbol;Acc:HGNC:591] | 369 | 1.208 | 0.1905 | Yes |
| 14 | TLR8 | toll like receptor 8 [Source:HGNC Symbol;Acc:HGNC:15632] | 386 | 1.192 | 0.2021 | Yes |
| 15 | C3AR1 | complement C3a receptor 1 [Source:HGNC Symbol;Acc:HGNC:1319] | 387 | 1.191 | 0.2148 | Yes |
| 16 | IL2RG | interleukin 2 receptor subunit gamma [Source:HGNC Symbol;Acc:HGNC:6010] | 432 | 1.141 | 0.2241 | Yes |
| 17 | PSMB8 | proteasome 20S subunit beta 8 [Source:HGNC Symbol;Acc:HGNC:9545] | 467 | 1.101 | 0.2336 | Yes |
| 18 | ACE | angiotensin I converting enzyme [Source:HGNC Symbol;Acc:HGNC:2707] | 499 | 1.074 | 0.2430 | Yes |
| 19 | TMEM100 | transmembrane protein 100 [Source:HGNC Symbol;Acc:HGNC:25607] | 502 | 1.069 | 0.2542 | Yes |
| 20 | G0S2 | G0/G1 switch 2 [Source:HGNC Symbol;Acc:HGNC:30229] | 516 | 1.054 | 0.2646 | Yes |
| 21 | PLAU | "plasminogen activator, urokinase [Source:HGNC Symbol;Acc:HGNC:9052]" | 537 | 1.033 | 0.2743 | Yes |
| 22 | ANGPTL4 | angiopoietin like 4 [Source:HGNC Symbol;Acc:HGNC:16039] | 565 | 1.018 | 0.2834 | Yes |
| 23 | SPON1 | spondin 1 [Source:HGNC Symbol;Acc:HGNC:11252] | 661 | 0.956 | 0.2874 | Yes |
| 24 | GLRX | glutaredoxin [Source:HGNC Symbol;Acc:HGNC:4330] | 694 | 0.937 | 0.2953 | Yes |
| 25 | H2BC3 | H2B clustered histone 3 [Source:HGNC Symbol;Acc:HGNC:4751] | 704 | 0.930 | 0.3046 | Yes |
| 26 | EPHB2 | EPH receptor B2 [Source:HGNC Symbol;Acc:HGNC:3393] | 729 | 0.915 | 0.3128 | Yes |
| 27 | IL7R | interleukin 7 receptor [Source:HGNC Symbol;Acc:HGNC:6024] | 816 | 0.869 | 0.3165 | Yes |
| 28 | TNFRSF1B | TNF receptor superfamily member 1B [Source:HGNC Symbol;Acc:HGNC:11917] | 845 | 0.864 | 0.3238 | Yes |
| 29 | RETN | resistin [Source:HGNC Symbol;Acc:HGNC:20389] | 993 | 0.822 | 0.3231 | Yes |
| 30 | DOCK2 | dedicator of cytokinesis 2 [Source:HGNC Symbol;Acc:HGNC:2988] | 1067 | 0.785 | 0.3267 | Yes |
| 31 | TRAF1 | TNF receptor associated factor 1 [Source:HGNC Symbol;Acc:HGNC:12031] | 1077 | 0.781 | 0.3344 | Yes |
| 32 | IL10RA | interleukin 10 receptor subunit alpha [Source:HGNC Symbol;Acc:HGNC:5964] | 1082 | 0.778 | 0.3424 | Yes |
| 33 | ITGA2 | integrin subunit alpha 2 [Source:HGNC Symbol;Acc:HGNC:6137] | 1109 | 0.769 | 0.3489 | Yes |
| 34 | RGS16 | regulator of G protein signaling 16 [Source:HGNC Symbol;Acc:HGNC:9997] | 1189 | 0.735 | 0.3517 | Yes |
| 35 | CTSS | cathepsin S [Source:HGNC Symbol;Acc:HGNC:2545] | 1201 | 0.731 | 0.3587 | Yes |
| 36 | MAFB | MAF bZIP transcription factor B [Source:HGNC Symbol;Acc:HGNC:6408] | 1239 | 0.719 | 0.3640 | Yes |
| 37 | ADAM8 | ADAM metallopeptidase domain 8 [Source:HGNC Symbol;Acc:HGNC:215] | 1301 | 0.695 | 0.3674 | Yes |
| 38 | MAP4K1 | mitogen-activated protein kinase kinase kinase kinase 1 [Source:HGNC Symbol;Acc:HGNC:6863] | 1305 | 0.695 | 0.3746 | Yes |
| 39 | IRF8 | interferon regulatory factor 8 [Source:HGNC Symbol;Acc:HGNC:5358] | 1378 | 0.667 | 0.3770 | Yes |
| 40 | ARG1 | arginase 1 [Source:HGNC Symbol;Acc:HGNC:663] | 1507 | 0.624 | 0.3754 | Yes |
| 41 | ITGB2 | integrin subunit beta 2 [Source:HGNC Symbol;Acc:HGNC:6155] | 1632 | 0.585 | 0.3736 | Yes |
| 42 | FCER1G | Fc fragment of IgE receptor Ig [Source:HGNC Symbol;Acc:HGNC:3611] | 1652 | 0.578 | 0.3785 | Yes |
| 43 | CIDEA | cell death inducing DFFA like effector a [Source:HGNC Symbol;Acc:HGNC:1976] | 1661 | 0.576 | 0.3841 | Yes |
| 44 | PTPRR | protein tyrosine phosphatase receptor type R [Source:HGNC Symbol;Acc:HGNC:9680] | 1690 | 0.571 | 0.3884 | Yes |
| 45 | TNFAIP3 | TNF alpha induced protein 3 [Source:HGNC Symbol;Acc:HGNC:11896] | 1713 | 0.564 | 0.3930 | Yes |
| 46 | IKZF1 | IKAROS family zinc finger 1 [Source:HGNC Symbol;Acc:HGNC:13176] | 1741 | 0.557 | 0.3972 | Yes |
| 47 | LCP1 | lymphocyte cytosolic protein 1 [Source:HGNC Symbol;Acc:HGNC:6528] | 1759 | 0.553 | 0.4019 | Yes |
| 48 | SNAP25 | synaptosome associated protein 25 [Source:HGNC Symbol;Acc:HGNC:11132] | 1825 | 0.538 | 0.4035 | Yes |
| 49 | MAP3K1 | mitogen-activated protein kinase kinase kinase 1 [Source:HGNC Symbol;Acc:HGNC:6848] | 1827 | 0.537 | 0.4091 | Yes |
| 50 | MPZL2 | myelin protein zero like 2 [Source:HGNC Symbol;Acc:HGNC:3496] | 1897 | 0.525 | 0.4102 | Yes |
| 51 | CSF2 | colony stimulating factor 2 [Source:HGNC Symbol;Acc:HGNC:2434] | 1913 | 0.521 | 0.4148 | Yes |
| 52 | ALDH1A3 | aldehyde dehydrogenase 1 family member A3 [Source:HGNC Symbol;Acc:HGNC:409] | 1932 | 0.516 | 0.4191 | Yes |
| 53 | SCG5 | secretogranin V [Source:HGNC Symbol;Acc:HGNC:10816] | 1980 | 0.507 | 0.4215 | Yes |
| 54 | LAT2 | linker for activation of T cells family member 2 [Source:HGNC Symbol;Acc:HGNC:12749] | 1988 | 0.506 | 0.4264 | Yes |
| 55 | NIN | ninein [Source:HGNC Symbol;Acc:HGNC:14906] | 2292 | 0.468 | 0.4118 | No |
| 56 | PPBP | pro-platelet basic protein [Source:HGNC Symbol;Acc:HGNC:9240] | 2311 | 0.466 | 0.4156 | No |
| 57 | TSPAN13 | tetraspanin 13 [Source:HGNC Symbol;Acc:HGNC:21643] | 2557 | 0.441 | 0.4045 | No |
| 58 | PLEK2 | pleckstrin 2 [Source:HGNC Symbol;Acc:HGNC:19238] | 2778 | 0.421 | 0.3947 | No |
| 59 | SOX9 | SRY-box transcription factor 9 [Source:HGNC Symbol;Acc:HGNC:11204] | 2780 | 0.419 | 0.3991 | No |
| 60 | CD37 | CD37 molecule [Source:HGNC Symbol;Acc:HGNC:1666] | 2793 | 0.417 | 0.4028 | No |
| 61 | USH1C | USH1 protein network component harmonin [Source:HGNC Symbol;Acc:HGNC:12597] | 2853 | 0.411 | 0.4033 | No |
| 62 | SNAP91 | synaptosome associated protein 91 [Source:HGNC Symbol;Acc:HGNC:14986] | 2880 | 0.411 | 0.4060 | No |
| 63 | ALDH1A2 | aldehyde dehydrogenase 1 family member A2 [Source:HGNC Symbol;Acc:HGNC:15472] | 2989 | 0.406 | 0.4034 | No |
| 64 | MMP9 | matrix metallopeptidase 9 [Source:HGNC Symbol;Acc:HGNC:7176] | 3018 | 0.401 | 0.4058 | No |
| 65 | SLPI | secretory leukocyte peptidase inhibitor [Source:HGNC Symbol;Acc:HGNC:11092] | 3174 | 0.376 | 0.3998 | No |
| 66 | LAPTM5 | lysosomal protein transmembrane 5 [Source:HGNC Symbol;Acc:HGNC:29612] | 3250 | 0.366 | 0.3989 | No |
| 67 | KIF5C | kinesin family member 5C [Source:HGNC Symbol;Acc:HGNC:6325] | 3308 | 0.356 | 0.3990 | No |
| 68 | INHBA | inhibin subunit beta A [Source:HGNC Symbol;Acc:HGNC:6066] | 3323 | 0.354 | 0.4018 | No |
| 69 | DCBLD2 | "discoidin, CUB and LCCL domain containing 2 [Source:HGNC Symbol;Acc:HGNC:24627]" | 3417 | 0.340 | 0.3994 | No |
| 70 | CSF2RA | colony stimulating factor 2 receptor subunit alpha [Source:HGNC Symbol;Acc:HGNC:2435] | 3459 | 0.333 | 0.4003 | No |
| 71 | TPH1 | tryptophan hydroxylase 1 [Source:HGNC Symbol;Acc:HGNC:12008] | 3489 | 0.329 | 0.4019 | No |
| 72 | HOXD11 | homeobox D11 [Source:HGNC Symbol;Acc:HGNC:5134] | 3541 | 0.323 | 0.4021 | No |
| 73 | HDAC9 | histone deacetylase 9 [Source:HGNC Symbol;Acc:HGNC:14065] | 3665 | 0.306 | 0.3974 | No |
| 74 | VWA5A | von Willebrand factor A domain containing 5A [Source:HGNC Symbol;Acc:HGNC:6658] | 3775 | 0.293 | 0.3935 | No |
| 75 | CAB39L | calcium binding protein 39 like [Source:HGNC Symbol;Acc:HGNC:20290] | 3784 | 0.292 | 0.3961 | No |
| 76 | LY96 | lymphocyte antigen 96 [Source:HGNC Symbol;Acc:HGNC:17156] | 3916 | 0.277 | 0.3905 | No |
| 77 | CMKLR1 | chemerin chemokine-like receptor 1 [Source:HGNC Symbol;Acc:HGNC:2121] | 3919 | 0.276 | 0.3933 | No |
| 78 | RBM4 | RNA binding motif protein 4 [Source:HGNC Symbol;Acc:HGNC:9901] | 3975 | 0.268 | 0.3926 | No |
| 79 | NAP1L2 | nucleosome assembly protein 1 like 2 [Source:HGNC Symbol;Acc:HGNC:7638] | 3987 | 0.267 | 0.3948 | No |
| 80 | PRRX1 | paired related homeobox 1 [Source:HGNC Symbol;Acc:HGNC:9142] | 4018 | 0.264 | 0.3956 | No |
| 81 | ZNF277 | zinc finger protein 277 [Source:HGNC Symbol;Acc:HGNC:13070] | 4186 | 0.245 | 0.3874 | No |
| 82 | TOR1AIP2 | torsin 1A interacting protein 2 [Source:HGNC Symbol;Acc:HGNC:24055] | 4190 | 0.245 | 0.3899 | No |
| 83 | EREG | epiregulin [Source:HGNC Symbol;Acc:HGNC:3443] | 4321 | 0.231 | 0.3839 | No |
| 84 | AMMECR1 | AMMECR nuclear protein 1 [Source:HGNC Symbol;Acc:HGNC:467] | 4370 | 0.226 | 0.3832 | No |
| 85 | BTC | betacellulin [Source:HGNC Symbol;Acc:HGNC:1121] | 4475 | 0.215 | 0.3788 | No |
| 86 | CCSER2 | coiled-coil serine rich protein 2 [Source:HGNC Symbol;Acc:HGNC:29197] | 4570 | 0.207 | 0.3749 | No |
| 87 | STRN | striatin [Source:HGNC Symbol;Acc:HGNC:11424] | 4787 | 0.189 | 0.3630 | No |
| 88 | GNG11 | G protein subunit gamma 11 [Source:HGNC Symbol;Acc:HGNC:4403] | 4922 | 0.175 | 0.3562 | No |
| 89 | FGF9 | fibroblast growth factor 9 [Source:HGNC Symbol;Acc:HGNC:3687] | 4979 | 0.170 | 0.3544 | No |
| 90 | PLAUR | "plasminogen activator, urokinase receptor [Source:HGNC Symbol;Acc:HGNC:9053]" | 5260 | 0.146 | 0.3378 | No |
| 91 | ADAM17 | ADAM metallopeptidase domain 17 [Source:HGNC Symbol;Acc:HGNC:195] | 5273 | 0.145 | 0.3386 | No |
| 92 | SPRY2 | sprouty RTK signaling antagonist 2 [Source:HGNC Symbol;Acc:HGNC:11270] | 5302 | 0.141 | 0.3383 | No |
| 93 | CROT | carnitine O-octanoyltransferase [Source:HGNC Symbol;Acc:HGNC:2366] | 5358 | 0.138 | 0.3362 | No |
| 94 | IL1RL2 | interleukin 1 receptor like 2 [Source:HGNC Symbol;Acc:HGNC:5999] | 5402 | 0.135 | 0.3348 | No |
| 95 | RBP4 | retinol binding protein 4 [Source:HGNC Symbol;Acc:HGNC:9922] | 5444 | 0.132 | 0.3336 | No |
| 96 | KCNN4 | potassium calcium-activated channel subfamily N member 4 [Source:HGNC Symbol;Acc:HGNC:6293] | 5520 | 0.126 | 0.3301 | No |
| 97 | WDR33 | WD repeat domain 33 [Source:HGNC Symbol;Acc:HGNC:25651] | 5672 | 0.114 | 0.3216 | No |
| 98 | PTBP2 | polypyrimidine tract binding protein 2 [Source:HGNC Symbol;Acc:HGNC:17662] | 5735 | 0.110 | 0.3187 | No |
| 99 | CDADC1 | cytidine and dCMP deaminase domain containing 1 [Source:HGNC Symbol;Acc:HGNC:20299] | 5828 | 0.104 | 0.3139 | No |
| 100 | CBL | Cbl proto-oncogene [Source:HGNC Symbol;Acc:HGNC:1541] | 5833 | 0.103 | 0.3147 | No |
| 101 | TRIB1 | tribbles pseudokinase 1 [Source:HGNC Symbol;Acc:HGNC:16891] | 6002 | 0.091 | 0.3048 | No |
| 102 | USP12 | ubiquitin specific peptidase 12 [Source:HGNC Symbol;Acc:HGNC:20485] | 6053 | 0.087 | 0.3025 | No |
| 103 | ATG10 | autophagy related 10 [Source:HGNC Symbol;Acc:HGNC:20315] | 6076 | 0.085 | 0.3020 | No |
| 104 | SDCCAG8 | SHH signaling and ciliogenesis regulator SDCCAG8 [Source:HGNC Symbol;Acc:HGNC:10671] | 6440 | 0.059 | 0.2792 | No |
| 105 | ANO1 | anoctamin 1 [Source:HGNC Symbol;Acc:HGNC:21625] | 6572 | 0.050 | 0.2712 | No |
| 106 | ABCB1 | ATP binding cassette subfamily B member 1 [Source:HGNC Symbol;Acc:HGNC:40] | 6707 | 0.042 | 0.2630 | No |
| 107 | PEG3 | paternally expressed 3 [Source:HGNC Symbol;Acc:HGNC:8826] | 6754 | 0.040 | 0.2605 | No |
| 108 | SCG3 | secretogranin III [Source:HGNC Symbol;Acc:HGNC:13707] | 6953 | 0.026 | 0.2480 | No |
| 109 | ST6GAL1 | "ST6 beta-galactoside alpha-2,6-sialyltransferase 1 [Source:HGNC Symbol;Acc:HGNC:10860]" | 6995 | 0.025 | 0.2456 | No |
| 110 | PTGS2 | prostaglandin-endoperoxide synthase 2 [Source:HGNC Symbol;Acc:HGNC:9605] | 7157 | 0.015 | 0.2353 | No |
| 111 | ERO1A | endoplasmic reticulum oxidoreductase 1 alpha [Source:HGNC Symbol;Acc:HGNC:13280] | 7181 | 0.013 | 0.2340 | No |
| 112 | ETV1 | ETS variant transcription factor 1 [Source:HGNC Symbol;Acc:HGNC:3490] | 7182 | 0.013 | 0.2341 | No |
| 113 | TSPAN7 | tetraspanin 7 [Source:HGNC Symbol;Acc:HGNC:11854] | 7293 | 0.007 | 0.2271 | No |
| 114 | AVL9 | AVL9 cell migration associated [Source:HGNC Symbol;Acc:HGNC:28994] | 7298 | 0.007 | 0.2269 | No |
| 115 | LIF | LIF interleukin 6 family cytokine [Source:HGNC Symbol;Acc:HGNC:6596] | 7310 | 0.006 | 0.2263 | No |
| 116 | GABRA3 | gamma-aminobutyric acid type A receptor subunit alpha3 [Source:HGNC Symbol;Acc:HGNC:4077] | 7367 | 0.002 | 0.2227 | No |
| 117 | ETV5 | ETS variant transcription factor 5 [Source:HGNC Symbol;Acc:HGNC:3494] | 7400 | 0.001 | 0.2206 | No |
| 118 | EMP1 | epithelial membrane protein 1 [Source:HGNC Symbol;Acc:HGNC:3333] | 7529 | -0.006 | 0.2124 | No |
| 119 | FBXO4 | F-box protein 4 [Source:HGNC Symbol;Acc:HGNC:13583] | 7633 | -0.010 | 0.2059 | No |
| 120 | PRELID3B | PRELI domain containing 3B [Source:HGNC Symbol;Acc:HGNC:15892] | 7661 | -0.011 | 0.2042 | No |
| 121 | RELN | reelin [Source:HGNC Symbol;Acc:HGNC:9957] | 7693 | -0.013 | 0.2024 | No |
| 122 | ETS1 | "ETS proto-oncogene 1, transcription factor [Source:HGNC Symbol;Acc:HGNC:3488]" | 7841 | -0.021 | 0.1931 | No |
| 123 | GFPT2 | glutamine-fructose-6-phosphate transaminase 2 [Source:HGNC Symbol;Acc:HGNC:4242] | 7969 | -0.027 | 0.1852 | No |
| 124 | PPP1R15A | protein phosphatase 1 regulatory subunit 15A [Source:HGNC Symbol;Acc:HGNC:14375] | 8019 | -0.030 | 0.1823 | No |
| 125 | CBR4 | carbonyl reductase 4 [Source:HGNC Symbol;Acc:HGNC:25891] | 8046 | -0.031 | 0.1810 | No |
| 126 | CCND2 | cyclin D2 [Source:HGNC Symbol;Acc:HGNC:1583] | 8119 | -0.036 | 0.1767 | No |
| 127 | PTCD2 | pentatricopeptide repeat domain 2 [Source:HGNC Symbol;Acc:HGNC:25734] | 8173 | -0.039 | 0.1737 | No |
| 128 | ID2 | inhibitor of DNA binding 2 [Source:HGNC Symbol;Acc:HGNC:5361] | 8387 | -0.048 | 0.1604 | No |
| 129 | GYPC | glycophorin C (Gerbich blood group) [Source:HGNC Symbol;Acc:HGNC:4704] | 8394 | -0.048 | 0.1606 | No |
| 130 | EVI5 | ecotropic viral integration site 5 [Source:HGNC Symbol;Acc:HGNC:3501] | 8558 | -0.058 | 0.1507 | No |
| 131 | GADD45G | growth arrest and DNA damage inducible gamma [Source:HGNC Symbol;Acc:HGNC:4097] | 8654 | -0.064 | 0.1452 | No |
| 132 | HBEGF | heparin binding EGF like growth factor [Source:HGNC Symbol;Acc:HGNC:3059] | 8784 | -0.071 | 0.1376 | No |
| 133 | NRP1 | neuropilin 1 [Source:HGNC Symbol;Acc:HGNC:8004] | 9151 | -0.091 | 0.1149 | No |
| 134 | ZNF639 | zinc finger protein 639 [Source:HGNC Symbol;Acc:HGNC:30950] | 9639 | -0.121 | 0.0848 | No |
| 135 | CXCR4 | C-X-C motif chemokine receptor 4 [Source:HGNC Symbol;Acc:HGNC:2561] | 9672 | -0.123 | 0.0840 | No |
| 136 | TSPAN1 | tetraspanin 1 [Source:HGNC Symbol;Acc:HGNC:20657] | 9854 | -0.134 | 0.0737 | No |
| 137 | IGF2 | insulin like growth factor 2 [Source:HGNC Symbol;Acc:HGNC:5466] | 9922 | -0.138 | 0.0709 | No |
| 138 | SEMA3B | semaphorin 3B [Source:HGNC Symbol;Acc:HGNC:10724] | 9978 | -0.141 | 0.0688 | No |
| 139 | GALNT3 | polypeptide N-acetylgalactosaminyltransferase 3 [Source:HGNC Symbol;Acc:HGNC:4125] | 10082 | -0.147 | 0.0637 | No |
| 140 | GUCY1A1 | guanylate cyclase 1 soluble subunit alpha 1 [Source:HGNC Symbol;Acc:HGNC:4685] | 10119 | -0.149 | 0.0630 | No |
| 141 | MMD | monocyte to macrophage differentiation associated [Source:HGNC Symbol;Acc:HGNC:7153] | 10243 | -0.156 | 0.0567 | No |
| 142 | TFPI | tissue factor pathway inhibitor [Source:HGNC Symbol;Acc:HGNC:11760] | 10316 | -0.160 | 0.0537 | No |
| 143 | GPNMB | glycoprotein nmb [Source:HGNC Symbol;Acc:HGNC:4462] | 10333 | -0.161 | 0.0544 | No |
| 144 | KLF4 | Kruppel like factor 4 [Source:HGNC Symbol;Acc:HGNC:6348] | 10415 | -0.167 | 0.0509 | No |
| 145 | MTMR10 | myotubularin related protein 10 [Source:HGNC Symbol;Acc:HGNC:25999] | 10581 | -0.176 | 0.0422 | No |
| 146 | RABGAP1L | RAB GTPase activating protein 1 like [Source:HGNC Symbol;Acc:HGNC:24663] | 10642 | -0.180 | 0.0402 | No |
| 147 | AKT2 | AKT serine/threonine kinase 2 [Source:HGNC Symbol;Acc:HGNC:392] | 11023 | -0.201 | 0.0178 | No |
| 148 | SCN1B | sodium voltage-gated channel beta subunit 1 [Source:HGNC Symbol;Acc:HGNC:10586] | 11044 | -0.202 | 0.0186 | No |
| 149 | FUCA1 | alpha-L-fucosidase 1 [Source:HGNC Symbol;Acc:HGNC:4006] | 11365 | -0.221 | 0.0003 | No |
| 150 | DUSP6 | dual specificity phosphatase 6 [Source:HGNC Symbol;Acc:HGNC:3072] | 11560 | -0.232 | -0.0097 | No |
| 151 | IL33 | interleukin 33 [Source:HGNC Symbol;Acc:HGNC:16028] | 11634 | -0.237 | -0.0119 | No |
| 152 | YRDC | yrdC N6-threonylcarbamoyltransferase domain containing [Source:HGNC Symbol;Acc:HGNC:28905] | 11663 | -0.238 | -0.0112 | No |
| 153 | ADGRL4 | adhesion G protein-coupled receptor L4 [Source:HGNC Symbol;Acc:HGNC:20822] | 11763 | -0.245 | -0.0150 | No |
| 154 | ITGBL1 | integrin subunit beta like 1 [Source:HGNC Symbol;Acc:HGNC:6164] | 11866 | -0.252 | -0.0189 | No |
| 155 | BTBD3 | BTB domain containing 3 [Source:HGNC Symbol;Acc:HGNC:15854] | 11895 | -0.253 | -0.0180 | No |
| 156 | MALL | "mal, T cell differentiation protein like [Source:HGNC Symbol;Acc:HGNC:6818]" | 12124 | -0.269 | -0.0299 | No |
| 157 | PRKG2 | protein kinase cGMP-dependent 2 [Source:HGNC Symbol;Acc:HGNC:9416] | 12298 | -0.280 | -0.0381 | No |
| 158 | ETV4 | ETS variant transcription factor 4 [Source:HGNC Symbol;Acc:HGNC:3493] | 12369 | -0.286 | -0.0396 | No |
| 159 | ADGRA2 | adhesion G protein-coupled receptor A2 [Source:HGNC Symbol;Acc:HGNC:17849] | 12421 | -0.290 | -0.0398 | No |
| 160 | PECAM1 | platelet and endothelial cell adhesion molecule 1 [Source:HGNC Symbol;Acc:HGNC:8823] | 12443 | -0.292 | -0.0381 | No |
| 161 | BMP2 | bone morphogenetic protein 2 [Source:HGNC Symbol;Acc:HGNC:1069] | 12618 | -0.305 | -0.0461 | No |
| 162 | CBX8 | chromobox 8 [Source:HGNC Symbol;Acc:HGNC:15962] | 12774 | -0.318 | -0.0527 | No |
| 163 | DNMBP | dynamin binding protein [Source:HGNC Symbol;Acc:HGNC:30373] | 13291 | -0.366 | -0.0821 | No |
| 164 | F13A1 | coagulation factor XIII A chain [Source:HGNC Symbol;Acc:HGNC:3531] | 13508 | -0.393 | -0.0919 | No |
| 165 | AKAP12 | A-kinase anchoring protein 12 [Source:HGNC Symbol;Acc:HGNC:370] | 13876 | -0.428 | -0.1111 | No |
| 166 | ANKH | ANKH inorganic pyrophosphate transport regulator [Source:HGNC Symbol;Acc:HGNC:15492] | 14048 | -0.439 | -0.1175 | No |
| 167 | BPGM | bisphosphoglycerate mutase [Source:HGNC Symbol;Acc:HGNC:1093] | 14058 | -0.441 | -0.1134 | No |
| 168 | TMEM158 | transmembrane protein 158 [Source:HGNC Symbol;Acc:HGNC:30293] | 14203 | -0.465 | -0.1177 | No |
| 169 | PLAT | "plasminogen activator, tissue type [Source:HGNC Symbol;Acc:HGNC:9051]" | 14379 | -0.490 | -0.1238 | No |
| 170 | SPP1 | secreted phosphoprotein 1 [Source:HGNC Symbol;Acc:HGNC:11255] | 14610 | -0.535 | -0.1330 | No |
| 171 | MYCN | "MYCN proto-oncogene, bHLH transcription factor [Source:HGNC Symbol;Acc:HGNC:7559]" | 14712 | -0.557 | -0.1336 | No |
| 172 | FLT4 | fms related receptor tyrosine kinase 4 [Source:HGNC Symbol;Acc:HGNC:3767] | 14722 | -0.559 | -0.1282 | No |
| 173 | MMP11 | matrix metallopeptidase 11 [Source:HGNC Symbol;Acc:HGNC:7157] | 14904 | -0.621 | -0.1333 | No |
| 174 | F2RL1 | F2R like trypsin receptor 1 [Source:HGNC Symbol;Acc:HGNC:3538] | 14941 | -0.640 | -0.1288 | No |
| 175 | TNNT2 | "troponin T2, cardiac type [Source:HGNC Symbol;Acc:HGNC:11949]" | 14979 | -0.655 | -0.1243 | No |
| 176 | PLVAP | plasmalemma vesicle associated protein [Source:HGNC Symbol;Acc:HGNC:13635] | 14985 | -0.657 | -0.1176 | No |
| 177 | NGF | nerve growth factor [Source:HGNC Symbol;Acc:HGNC:7808] | 15003 | -0.664 | -0.1116 | No |
| 178 | TRIB2 | tribbles pseudokinase 2 [Source:HGNC Symbol;Acc:HGNC:30809] | 15069 | -0.701 | -0.1084 | No |
| 179 | SPARCL1 | SPARC like 1 [Source:HGNC Symbol;Acc:HGNC:11220] | 15169 | -0.760 | -0.1067 | No |
| 180 | CFH | complement factor H [Source:HGNC Symbol;Acc:HGNC:4883] | 15171 | -0.760 | -0.0987 | No |
| 181 | GPRC5B | G protein-coupled receptor class C group 5 member B [Source:HGNC Symbol;Acc:HGNC:13308] | 15199 | -0.782 | -0.0921 | No |
| 182 | CPE | carboxypeptidase E [Source:HGNC Symbol;Acc:HGNC:2303] | 15202 | -0.784 | -0.0839 | No |
| 183 | EPB41L3 | erythrocyte membrane protein band 4.1 like 3 [Source:HGNC Symbol;Acc:HGNC:3380] | 15218 | -0.791 | -0.0765 | No |
| 184 | ENG | endoglin [Source:HGNC Symbol;Acc:HGNC:3349] | 15264 | -0.815 | -0.0707 | No |
| 185 | TMEM176A | transmembrane protein 176A [Source:HGNC Symbol;Acc:HGNC:24930] | 15349 | -0.861 | -0.0670 | No |
| 186 | ADAMDEC1 | ADAM like decysin 1 [Source:HGNC Symbol;Acc:HGNC:16299] | 15390 | -0.881 | -0.0602 | No |
| 187 | HKDC1 | hexokinase domain containing 1 [Source:HGNC Symbol;Acc:HGNC:23302] | 15461 | -0.971 | -0.0544 | No |
| 188 | JUP | junction plakoglobin [Source:HGNC Symbol;Acc:HGNC:6207] | 15477 | -1.001 | -0.0447 | No |
| 189 | TMEM176B | transmembrane protein 176B [Source:HGNC Symbol;Acc:HGNC:29596] | 15507 | -1.048 | -0.0355 | No |
| 190 | CA2 | carbonic anhydrase 2 [Source:HGNC Symbol;Acc:HGNC:1373] | 15522 | -1.087 | -0.0248 | No |
| 191 | IGFBP3 | insulin like growth factor binding protein 3 [Source:HGNC Symbol;Acc:HGNC:5472] | 15536 | -1.107 | -0.0139 | No |
| 192 | NR1H4 | nuclear receptor subfamily 1 group H member 4 [Source:HGNC Symbol;Acc:HGNC:7967] | 15660 | -2.101 | 0.0005 | No |
Table: GSEA details [plain text format]

  

Fig 2: HALLMARK\_KRAS\_SIGNALING\_UP      
 Blue-Pink O' Gram in the Space of the Analyzed GeneSet

  

Fig 3: HALLMARK\_KRAS\_SIGNALING\_UP: Random ES distribution      
 Gene set null distribution of ES for **HALLMARK\_KRAS\_SIGNALING\_UP**
