# Supplementary material for: In vivo CRISPR screens reveal Serpinb9 and Adam2 as regulators of immune therapy response in lung cancer
Source: Nat Commun. 2023 May 31;14:3150. doi: 10.1038/s41467-023-38841-7 (PMC10232477; doi:10.1038/s41467-023-38841-7)
Supplement: Supplementary file 13 — Source Data [file 41467_2023_38841_MOESM13_ESM.zip › Figure 4E.zip!/my_analysis.Gsea.1640146340255_Hallmark/HALLMARK_ANDROGEN_RESPONSE.html]

Details for gene set HALLMARK\_ANDROGEN\_RESPONSE[GSEA]

|  || Dataset | December 21, 2021\_for GSEA run\_collapsed\_to\_symbols.GSEA\_B6\_NI.cls #CTLR\_NI\_versus\_ADAM2\_NI |
| Phenotype | GSEA\_B6\_NI.cls#CTLR\_NI\_versus\_ADAM2\_NI |
| Upregulated in class | CTLR\_NI |
| GeneSet | HALLMARK\_ANDROGEN\_RESPONSE |
| Enrichment Score (ES) | 0.26140374 |
| Normalized Enrichment Score (NES) | 1.0675722 |
| Nominal p-value | 0.4369748 |
| FDR q-value | 0.72422844 |
| FWER p-Value | 1.0 |
Table: GSEA Results Summary

  

Fig 1: Enrichment plot: HALLMARK\_ANDROGEN\_RESPONSE      
 Profile of the Running ES Score & Positions of GeneSet Members on the Rank Ordered List

  

| SYMBOL | TITLE | RANK IN GENE LIST | RANK METRIC SCORE | RUNNING ES | CORE ENRICHMENT || 1 | HOMER2 | homer scaffold protein 2 [Source:HGNC Symbol;Acc:HGNC:17513] | 37 | 2.189 | 0.0791 | Yes |
| 2 | MAP7 | microtubule associated protein 7 [Source:HGNC Symbol;Acc:HGNC:6869] | 282 | 1.335 | 0.1132 | Yes |
| 3 | B2M | beta-2-microglobulin [Source:HGNC Symbol;Acc:HGNC:914] | 441 | 1.131 | 0.1452 | Yes |
| 4 | INPP4B | inositol polyphosphate-4-phosphatase type II B [Source:HGNC Symbol;Acc:HGNC:6075] | 1292 | 0.698 | 0.1166 | Yes |
| 5 | SLC26A2 | solute carrier family 26 member 2 [Source:HGNC Symbol;Acc:HGNC:10994] | 1515 | 0.621 | 0.1254 | Yes |
| 6 | TNFAIP8 | TNF alpha induced protein 8 [Source:HGNC Symbol;Acc:HGNC:17260] | 1675 | 0.574 | 0.1366 | Yes |
| 7 | MAF | MAF bZIP transcription factor [Source:HGNC Symbol;Acc:HGNC:6776] | 1702 | 0.567 | 0.1561 | Yes |
| 8 | BMPR1B | bone morphogenetic protein receptor type 1B [Source:HGNC Symbol;Acc:HGNC:1077] | 1705 | 0.566 | 0.1770 | Yes |
| 9 | IQGAP2 | IQ motif containing GTPase activating protein 2 [Source:HGNC Symbol;Acc:HGNC:6111] | 1845 | 0.534 | 0.1880 | Yes |
| 10 | PTK2B | protein tyrosine kinase 2 beta [Source:HGNC Symbol;Acc:HGNC:9612] | 1877 | 0.529 | 0.2057 | Yes |
| 11 | ALDH1A3 | aldehyde dehydrogenase 1 family member A3 [Source:HGNC Symbol;Acc:HGNC:409] | 1932 | 0.516 | 0.2214 | Yes |
| 12 | SELENOP | selenoprotein P [Source:HGNC Symbol;Acc:HGNC:10751] | 2188 | 0.481 | 0.2230 | Yes |
| 13 | HSD17B14 | hydroxysteroid 17-beta dehydrogenase 14 [Source:HGNC Symbol;Acc:HGNC:23238] | 2235 | 0.478 | 0.2379 | Yes |
| 14 | SPDEF | SAM pointed domain containing ETS transcription factor [Source:HGNC Symbol;Acc:HGNC:17257] | 2611 | 0.433 | 0.2299 | Yes |
| 15 | LIFR | LIF receptor subunit alpha [Source:HGNC Symbol;Acc:HGNC:6597] | 3029 | 0.399 | 0.2180 | Yes |
| 16 | ELL2 | elongation factor for RNA polymerase II 2 [Source:HGNC Symbol;Acc:HGNC:17064] | 3031 | 0.399 | 0.2327 | Yes |
| 17 | ABCC4 | ATP binding cassette subfamily C member 4 [Source:HGNC Symbol;Acc:HGNC:55] | 3304 | 0.356 | 0.2285 | Yes |
| 18 | NCOA4 | nuclear receptor coactivator 4 [Source:HGNC Symbol;Acc:HGNC:7671] | 3376 | 0.345 | 0.2368 | Yes |
| 19 | STEAP4 | STEAP4 metalloreductase [Source:HGNC Symbol;Acc:HGNC:21923] | 3483 | 0.330 | 0.2423 | Yes |
| 20 | CENPN | centromere protein N [Source:HGNC Symbol;Acc:HGNC:30873] | 3513 | 0.326 | 0.2526 | Yes |
| 21 | ELK4 | ETS transcription factor ELK4 [Source:HGNC Symbol;Acc:HGNC:3326] | 3658 | 0.307 | 0.2548 | Yes |
| 22 | HERC3 | HECT and RLD domain containing E3 ubiquitin protein ligase 3 [Source:HGNC Symbol;Acc:HGNC:4876] | 3729 | 0.299 | 0.2614 | Yes |
| 23 | TPD52 | tumor protein D52 [Source:HGNC Symbol;Acc:HGNC:12005] | 4259 | 0.236 | 0.2362 | No |
| 24 | KRT8 | keratin 8 [Source:HGNC Symbol;Acc:HGNC:6446] | 4293 | 0.233 | 0.2428 | No |
| 25 | SGK1 | serum/glucocorticoid regulated kinase 1 [Source:HGNC Symbol;Acc:HGNC:10810] | 4462 | 0.217 | 0.2401 | No |
| 26 | GSR | glutathione-disulfide reductase [Source:HGNC Symbol;Acc:HGNC:4623] | 4657 | 0.200 | 0.2350 | No |
| 27 | SRP19 | signal recognition particle 19 [Source:HGNC Symbol;Acc:HGNC:11300] | 4920 | 0.175 | 0.2248 | No |
| 28 | PIAS1 | protein inhibitor of activated STAT 1 [Source:HGNC Symbol;Acc:HGNC:2752] | 5467 | 0.130 | 0.1945 | No |
| 29 | NGLY1 | N-glycanase 1 [Source:HGNC Symbol;Acc:HGNC:17646] | 5506 | 0.127 | 0.1968 | No |
| 30 | SAT1 | spermidine/spermine N1-acetyltransferase 1 [Source:HGNC Symbol;Acc:HGNC:10540] | 6202 | 0.076 | 0.1550 | No |
| 31 | APPBP2 | amyloid beta precursor protein binding protein 2 [Source:HGNC Symbol;Acc:HGNC:622] | 6598 | 0.048 | 0.1315 | No |
| 32 | MERTK | "MER proto-oncogene, tyrosine kinase [Source:HGNC Symbol;Acc:HGNC:7027]" | 6672 | 0.044 | 0.1284 | No |
| 33 | SRF | serum response factor [Source:HGNC Symbol;Acc:HGNC:11291] | 6679 | 0.044 | 0.1297 | No |
| 34 | PTPN21 | protein tyrosine phosphatase non-receptor type 21 [Source:HGNC Symbol;Acc:HGNC:9651] | 6717 | 0.041 | 0.1288 | No |
| 35 | B4GALT1 | "beta-1,4-galactosyltransferase 1 [Source:HGNC Symbol;Acc:HGNC:924]" | 6824 | 0.035 | 0.1233 | No |
| 36 | HMGCR | 3-hydroxy-3-methylglutaryl-CoA reductase [Source:HGNC Symbol;Acc:HGNC:5006] | 6855 | 0.033 | 0.1226 | No |
| 37 | HMGCS1 | 3-hydroxy-3-methylglutaryl-CoA synthase 1 [Source:HGNC Symbol;Acc:HGNC:5007] | 6875 | 0.031 | 0.1225 | No |
| 38 | ACSL3 | acyl-CoA synthetase long chain family member 3 [Source:HGNC Symbol;Acc:HGNC:3570] | 7111 | 0.018 | 0.1081 | No |
| 39 | PDLIM5 | PDZ and LIM domain 5 [Source:HGNC Symbol;Acc:HGNC:17468] | 7279 | 0.008 | 0.0977 | No |
| 40 | CAMKK2 | calcium/calmodulin dependent protein kinase kinase 2 [Source:HGNC Symbol;Acc:HGNC:1470] | 7294 | 0.007 | 0.0970 | No |
| 41 | CDK6 | cyclin dependent kinase 6 [Source:HGNC Symbol;Acc:HGNC:1777] | 7351 | 0.004 | 0.0936 | No |
| 42 | CDC14B | cell division cycle 14B [Source:HGNC Symbol;Acc:HGNC:1719] | 7457 | -0.002 | 0.0869 | No |
| 43 | DBI | "diazepam binding inhibitor, acyl-CoA binding protein [Source:HGNC Symbol;Acc:HGNC:2690]" | 7846 | -0.021 | 0.0628 | No |
| 44 | RPS6KA3 | ribosomal protein S6 kinase A3 [Source:HGNC Symbol;Acc:HGNC:10432] | 8182 | -0.039 | 0.0427 | No |
| 45 | GNAI3 | G protein subunit alpha i3 [Source:HGNC Symbol;Acc:HGNC:4387] | 8386 | -0.048 | 0.0315 | No |
| 46 | XRCC5 | X-ray repair cross complementing 5 [Source:HGNC Symbol;Acc:HGNC:12833] | 8473 | -0.054 | 0.0280 | No |
| 47 | ARID5B | AT-rich interaction domain 5B [Source:HGNC Symbol;Acc:HGNC:17362] | 8765 | -0.070 | 0.0119 | No |
| 48 | HPGD | 15-hydroxyprostaglandin dehydrogenase [Source:HGNC Symbol;Acc:HGNC:5154] | 8794 | -0.072 | 0.0128 | No |
| 49 | UAP1 | UDP-N-acetylglucosamine pyrophosphorylase 1 [Source:HGNC Symbol;Acc:HGNC:12457] | 8960 | -0.080 | 0.0052 | No |
| 50 | LMAN1 | "lectin, mannose binding 1 [Source:HGNC Symbol;Acc:HGNC:6631]" | 8976 | -0.081 | 0.0072 | No |
| 51 | ZBTB10 | zinc finger and BTB domain containing 10 [Source:HGNC Symbol;Acc:HGNC:30953] | 9002 | -0.083 | 0.0087 | No |
| 52 | STK39 | serine/threonine kinase 39 [Source:HGNC Symbol;Acc:HGNC:17717] | 9143 | -0.090 | 0.0031 | No |
| 53 | UBE2I | ubiquitin conjugating enzyme E2 I [Source:HGNC Symbol;Acc:HGNC:12485] | 9299 | -0.098 | -0.0032 | No |
| 54 | TSC22D1 | TSC22 domain family member 1 [Source:HGNC Symbol;Acc:HGNC:16826] | 9431 | -0.106 | -0.0077 | No |
| 55 | PGM3 | phosphoglucomutase 3 [Source:HGNC Symbol;Acc:HGNC:8907] | 9441 | -0.107 | -0.0043 | No |
| 56 | ZMIZ1 | zinc finger MIZ-type containing 1 [Source:HGNC Symbol;Acc:HGNC:16493] | 9517 | -0.112 | -0.0049 | No |
| 57 | IDI1 | isopentenyl-diphosphate delta isomerase 1 [Source:HGNC Symbol;Acc:HGNC:5387] | 9590 | -0.117 | -0.0052 | No |
| 58 | VAPA | VAMP associated protein A [Source:HGNC Symbol;Acc:HGNC:12648] | 9656 | -0.122 | -0.0048 | No |
| 59 | CCND1 | cyclin D1 [Source:HGNC Symbol;Acc:HGNC:1582] | 9851 | -0.134 | -0.0123 | No |
| 60 | ITGAV | integrin subunit alpha V [Source:HGNC Symbol;Acc:HGNC:6150] | 9955 | -0.139 | -0.0138 | No |
| 61 | ACTN1 | actinin alpha 1 [Source:HGNC Symbol;Acc:HGNC:163] | 9983 | -0.141 | -0.0102 | No |
| 62 | ADAMTS1 | ADAM metallopeptidase with thrombospondin type 1 motif 1 [Source:HGNC Symbol;Acc:HGNC:217] | 9998 | -0.142 | -0.0058 | No |
| 63 | SLC38A2 | solute carrier family 38 member 2 [Source:HGNC Symbol;Acc:HGNC:13448] | 10103 | -0.148 | -0.0070 | No |
| 64 | PA2G4 | proliferation-associated 2G4 [Source:HGNC Symbol;Acc:HGNC:8550] | 10108 | -0.148 | -0.0017 | No |
| 65 | GUCY1A1 | guanylate cyclase 1 soluble subunit alpha 1 [Source:HGNC Symbol;Acc:HGNC:4685] | 10119 | -0.149 | 0.0032 | No |
| 66 | MYL12A | myosin light chain 12A [Source:HGNC Symbol;Acc:HGNC:16701] | 10314 | -0.160 | -0.0033 | No |
| 67 | FKBP5 | FKBP prolyl isomerase 5 [Source:HGNC Symbol;Acc:HGNC:3721] | 10352 | -0.163 | 0.0004 | No |
| 68 | CCND3 | cyclin D3 [Source:HGNC Symbol;Acc:HGNC:1585] | 10446 | -0.168 | 0.0007 | No |
| 69 | GPD1L | glycerol-3-phosphate dehydrogenase 1 like [Source:HGNC Symbol;Acc:HGNC:28956] | 10502 | -0.172 | 0.0035 | No |
| 70 | UBE2J1 | ubiquitin conjugating enzyme E2 J1 [Source:HGNC Symbol;Acc:HGNC:17598] | 10703 | -0.183 | -0.0025 | No |
| 71 | SMS | spermine synthase [Source:HGNC Symbol;Acc:HGNC:11123] | 10835 | -0.190 | -0.0038 | No |
| 72 | ABHD2 | "abhydrolase domain containing 2, acylglycerol lipase [Source:HGNC Symbol;Acc:HGNC:18717]" | 11126 | -0.208 | -0.0147 | No |
| 73 | ADRM1 | adhesion regulating molecule 1 [Source:HGNC Symbol;Acc:HGNC:15759] | 11166 | -0.210 | -0.0094 | No |
| 74 | MAK | male germ cell associated kinase [Source:HGNC Symbol;Acc:HGNC:6816] | 11505 | -0.230 | -0.0226 | No |
| 75 | AKT1 | AKT serine/threonine kinase 1 [Source:HGNC Symbol;Acc:HGNC:391] | 11570 | -0.233 | -0.0180 | No |
| 76 | SORD | sorbitol dehydrogenase [Source:HGNC Symbol;Acc:HGNC:11184] | 11863 | -0.252 | -0.0274 | No |
| 77 | DHCR24 | 24-dehydrocholesterol reductase [Source:HGNC Symbol;Acc:HGNC:2859] | 12239 | -0.276 | -0.0411 | No |
| 78 | XRCC6 | X-ray repair cross complementing 6 [Source:HGNC Symbol;Acc:HGNC:4055] | 12394 | -0.288 | -0.0403 | No |
| 79 | NDRG1 | N-myc downstream regulated 1 [Source:HGNC Symbol;Acc:HGNC:7679] | 12779 | -0.318 | -0.0531 | No |
| 80 | ELOVL5 | ELOVL fatty acid elongase 5 [Source:HGNC Symbol;Acc:HGNC:21308] | 12900 | -0.328 | -0.0486 | No |
| 81 | SPCS3 | signal peptidase complex subunit 3 [Source:HGNC Symbol;Acc:HGNC:26212] | 12968 | -0.335 | -0.0404 | No |
| 82 | TMEM50A | transmembrane protein 50A [Source:HGNC Symbol;Acc:HGNC:30590] | 13008 | -0.338 | -0.0303 | No |
| 83 | SEC24D | "SEC24 homolog D, COPII coat complex component [Source:HGNC Symbol;Acc:HGNC:10706]" | 13032 | -0.341 | -0.0191 | No |
| 84 | SCD | stearoyl-CoA desaturase [Source:HGNC Symbol;Acc:HGNC:10571] | 13043 | -0.342 | -0.0070 | No |
| 85 | RAB4A | "RAB4A, member RAS oncogene family [Source:HGNC Symbol;Acc:HGNC:9781]" | 13051 | -0.343 | 0.0053 | No |
| 86 | PLPP1 | phospholipid phosphatase 1 [Source:HGNC Symbol;Acc:HGNC:9228] | 13203 | -0.356 | 0.0089 | No |
| 87 | FADS1 | fatty acid desaturase 1 [Source:HGNC Symbol;Acc:HGNC:3574] | 13531 | -0.396 | 0.0026 | No |
| 88 | RRP12 | ribosomal RNA processing 12 homolog [Source:HGNC Symbol;Acc:HGNC:29100] | 13536 | -0.397 | 0.0171 | No |
| 89 | AKAP12 | A-kinase anchoring protein 12 [Source:HGNC Symbol;Acc:HGNC:370] | 13876 | -0.428 | 0.0113 | No |
| 90 | INSIG1 | insulin induced gene 1 [Source:HGNC Symbol;Acc:HGNC:6083] | 13987 | -0.430 | 0.0202 | No |
| 91 | KRT19 | keratin 19 [Source:HGNC Symbol;Acc:HGNC:6436] | 13989 | -0.430 | 0.0361 | No |
| 92 | ANKH | ANKH inorganic pyrophosphate transport regulator [Source:HGNC Symbol;Acc:HGNC:15492] | 14048 | -0.439 | 0.0488 | No |
| 93 | DNAJB9 | DnaJ heat shock protein family (Hsp40) member B9 [Source:HGNC Symbol;Acc:HGNC:6968] | 14214 | -0.467 | 0.0555 | No |
| 94 | PMEPA1 | "prostate transmembrane protein, androgen induced 1 [Source:HGNC Symbol;Acc:HGNC:14107]" | 14329 | -0.484 | 0.0662 | No |
| 95 | H1-0 | H1.0 linker histone [Source:HGNC Symbol;Acc:HGNC:4714] | 14582 | -0.529 | 0.0697 | No |
Table: GSEA details [plain text format]

  

Fig 2: HALLMARK\_ANDROGEN\_RESPONSE      
 Blue-Pink O' Gram in the Space of the Analyzed GeneSet

  

Fig 3: HALLMARK\_ANDROGEN\_RESPONSE: Random ES distribution      
 Gene set null distribution of ES for **HALLMARK\_ANDROGEN\_RESPONSE**
