# Supplementary material for: In vivo CRISPR screens reveal Serpinb9 and Adam2 as regulators of immune therapy response in lung cancer
Source: Nat Commun. 2023 May 31;14:3150. doi: 10.1038/s41467-023-38841-7 (PMC10232477; doi:10.1038/s41467-023-38841-7)
Supplement: Supplementary file 13 — Source Data [file 41467_2023_38841_MOESM13_ESM.zip › Figure 4E.zip!/my_analysis.Gsea.1640146340255_Hallmark/HALLMARK_P53_PATHWAY.html]

Details for gene set HALLMARK\_P53\_PATHWAY[GSEA]

|  || Dataset | December 21, 2021\_for GSEA run\_collapsed\_to\_symbols.GSEA\_B6\_NI.cls #CTLR\_NI\_versus\_ADAM2\_NI |
| Phenotype | GSEA\_B6\_NI.cls#CTLR\_NI\_versus\_ADAM2\_NI |
| Upregulated in class | ADAM2\_NI |
| GeneSet | HALLMARK\_P53\_PATHWAY |
| Enrichment Score (ES) | -0.2547982 |
| Normalized Enrichment Score (NES) | -0.83046734 |
| Nominal p-value | 0.79831934 |
| FDR q-value | 0.870613 |
| FWER p-Value | 1.0 |
Table: GSEA Results Summary

  

Fig 1: Enrichment plot: HALLMARK\_P53\_PATHWAY      
 Profile of the Running ES Score & Positions of GeneSet Members on the Rank Ordered List

  

| SYMBOL | TITLE | RANK IN GENE LIST | RANK METRIC SCORE | RUNNING ES | CORE ENRICHMENT || 1 | SERPINB5 | serpin family B member 5 [Source:HGNC Symbol;Acc:HGNC:8949] | 71 | 1.904 | 0.0188 | No |
| 2 | ST14 | ST14 transmembrane serine protease matriptase [Source:HGNC Symbol;Acc:HGNC:11344] | 136 | 1.687 | 0.0354 | No |
| 3 | IRAG2 | "inositol 1,4,5-triphosphate receptor associated 2 [Source:HGNC Symbol;Acc:HGNC:6690]" | 168 | 1.592 | 0.0530 | No |
| 4 | UPP1 | uridine phosphorylase 1 [Source:HGNC Symbol;Acc:HGNC:12576] | 191 | 1.509 | 0.0701 | No |
| 5 | TAP1 | "transporter 1, ATP binding cassette subfamily B member [Source:HGNC Symbol;Acc:HGNC:43]" | 241 | 1.389 | 0.0840 | No |
| 6 | CASP1 | caspase 1 [Source:HGNC Symbol;Acc:HGNC:1499] | 248 | 1.383 | 0.1007 | No |
| 7 | TCHH | trichohyalin [Source:HGNC Symbol;Acc:HGNC:11791] | 255 | 1.375 | 0.1172 | No |
| 8 | CYFIP2 | cytoplasmic FMR1 interacting protein 2 [Source:HGNC Symbol;Acc:HGNC:13760] | 304 | 1.294 | 0.1300 | No |
| 9 | DRAM1 | DNA damage regulated autophagy modulator 1 [Source:HGNC Symbol;Acc:HGNC:25645] | 402 | 1.165 | 0.1381 | No |
| 10 | FAS | Fas cell surface death receptor [Source:HGNC Symbol;Acc:HGNC:11920] | 418 | 1.154 | 0.1513 | No |
| 11 | ATF3 | activating transcription factor 3 [Source:HGNC Symbol;Acc:HGNC:785] | 453 | 1.117 | 0.1628 | No |
| 12 | IL1A | interleukin 1 alpha [Source:HGNC Symbol;Acc:HGNC:5991] | 540 | 1.032 | 0.1700 | No |
| 13 | TRAFD1 | TRAF-type zinc finger domain containing 1 [Source:HGNC Symbol;Acc:HGNC:24808] | 598 | 0.998 | 0.1785 | No |
| 14 | PERP | p53 apoptosis effector related to PMP22 [Source:HGNC Symbol;Acc:HGNC:17637] | 657 | 0.957 | 0.1866 | No |
| 15 | LDHB | lactate dehydrogenase B [Source:HGNC Symbol;Acc:HGNC:6541] | 687 | 0.938 | 0.1962 | No |
| 16 | VDR | vitamin D receptor [Source:HGNC Symbol;Acc:HGNC:12679] | 740 | 0.906 | 0.2040 | No |
| 17 | MXD1 | MAX dimerization protein 1 [Source:HGNC Symbol;Acc:HGNC:6761] | 898 | 0.842 | 0.2042 | No |
| 18 | TRIB3 | tribbles pseudokinase 3 [Source:HGNC Symbol;Acc:HGNC:16228] | 901 | 0.841 | 0.2144 | No |
| 19 | RGS16 | regulator of G protein signaling 16 [Source:HGNC Symbol;Acc:HGNC:9997] | 1189 | 0.735 | 0.2049 | No |
| 20 | SFN | stratifin [Source:HGNC Symbol;Acc:HGNC:10773] | 1200 | 0.732 | 0.2133 | No |
| 21 | SOCS1 | suppressor of cytokine signaling 1 [Source:HGNC Symbol;Acc:HGNC:19383] | 1359 | 0.674 | 0.2114 | No |
| 22 | COQ8A | coenzyme Q8A [Source:HGNC Symbol;Acc:HGNC:16812] | 1776 | 0.550 | 0.1912 | No |
| 23 | CDKN2A | cyclin dependent kinase inhibitor 2A [Source:HGNC Symbol;Acc:HGNC:1787] | 1779 | 0.550 | 0.1979 | No |
| 24 | MAPKAPK3 | MAPK activated protein kinase 3 [Source:HGNC Symbol;Acc:HGNC:6888] | 1952 | 0.512 | 0.1931 | No |
| 25 | ZBTB16 | zinc finger and BTB domain containing 16 [Source:HGNC Symbol;Acc:HGNC:12930] | 1992 | 0.505 | 0.1967 | No |
| 26 | NUDT15 | nudix hydrolase 15 [Source:HGNC Symbol;Acc:HGNC:23063] | 2086 | 0.494 | 0.1968 | No |
| 27 | STEAP3 | STEAP3 metalloreductase [Source:HGNC Symbol;Acc:HGNC:24592] | 2286 | 0.469 | 0.1897 | No |
| 28 | NOTCH1 | notch receptor 1 [Source:HGNC Symbol;Acc:HGNC:7881] | 2327 | 0.465 | 0.1929 | No |
| 29 | RNF19B | ring finger protein 19B [Source:HGNC Symbol;Acc:HGNC:26886] | 2349 | 0.460 | 0.1971 | No |
| 30 | BAK1 | BCL2 antagonist/killer 1 [Source:HGNC Symbol;Acc:HGNC:949] | 2363 | 0.457 | 0.2019 | No |
| 31 | APP | amyloid beta precursor protein [Source:HGNC Symbol;Acc:HGNC:620] | 2788 | 0.418 | 0.1797 | No |
| 32 | PITPNC1 | phosphatidylinositol transfer protein cytoplasmic 1 [Source:HGNC Symbol;Acc:HGNC:21045] | 2983 | 0.407 | 0.1721 | No |
| 33 | CGRRF1 | cell growth regulator with ring finger domain 1 [Source:HGNC Symbol;Acc:HGNC:15528] | 3007 | 0.403 | 0.1756 | No |
| 34 | GM2A | GM2 ganglioside activator [Source:HGNC Symbol;Acc:HGNC:4367] | 3039 | 0.397 | 0.1785 | No |
| 35 | TNNI1 | "troponin I1, slow skeletal type [Source:HGNC Symbol;Acc:HGNC:11945]" | 3110 | 0.387 | 0.1787 | No |
| 36 | BTG2 | BTG anti-proliferation factor 2 [Source:HGNC Symbol;Acc:HGNC:1131] | 3319 | 0.354 | 0.1696 | No |
| 37 | CDKN2AIP | CDKN2A interacting protein [Source:HGNC Symbol;Acc:HGNC:24325] | 3341 | 0.351 | 0.1726 | No |
| 38 | VAMP8 | vesicle associated membrane protein 8 [Source:HGNC Symbol;Acc:HGNC:12647] | 3355 | 0.349 | 0.1760 | No |
| 39 | IFI30 | IFI30 lysosomal thiol reductase [Source:HGNC Symbol;Acc:HGNC:5398] | 3485 | 0.330 | 0.1717 | No |
| 40 | TP63 | tumor protein p63 [Source:HGNC Symbol;Acc:HGNC:15979] | 3718 | 0.300 | 0.1604 | No |
| 41 | CCP110 | centriolar coiled-coil protein 110 [Source:HGNC Symbol;Acc:HGNC:24342] | 3725 | 0.300 | 0.1637 | No |
| 42 | APAF1 | apoptotic peptidase activating factor 1 [Source:HGNC Symbol;Acc:HGNC:576] | 3739 | 0.298 | 0.1666 | No |
| 43 | VWA5A | von Willebrand factor A domain containing 5A [Source:HGNC Symbol;Acc:HGNC:6658] | 3775 | 0.293 | 0.1679 | No |
| 44 | RAD51C | RAD51 paralog C [Source:HGNC Symbol;Acc:HGNC:9820] | 3925 | 0.276 | 0.1617 | No |
| 45 | DDB2 | damage specific DNA binding protein 2 [Source:HGNC Symbol;Acc:HGNC:2718] | 4125 | 0.251 | 0.1519 | No |
| 46 | DDIT3 | DNA damage inducible transcript 3 [Source:HGNC Symbol;Acc:HGNC:2726] | 4509 | 0.212 | 0.1297 | No |
| 47 | ACVR1B | activin A receptor type 1B [Source:HGNC Symbol;Acc:HGNC:172] | 4621 | 0.203 | 0.1251 | No |
| 48 | PTPRE | protein tyrosine phosphatase receptor type E [Source:HGNC Symbol;Acc:HGNC:9669] | 4700 | 0.197 | 0.1224 | No |
| 49 | TCN2 | transcobalamin 2 [Source:HGNC Symbol;Acc:HGNC:11653] | 4713 | 0.195 | 0.1241 | No |
| 50 | EPS8L2 | EPS8 like 2 [Source:HGNC Symbol;Acc:HGNC:21296] | 4852 | 0.183 | 0.1174 | No |
| 51 | DGKA | diacylglycerol kinase alpha [Source:HGNC Symbol;Acc:HGNC:2849] | 4949 | 0.173 | 0.1133 | No |
| 52 | SDC1 | syndecan 1 [Source:HGNC Symbol;Acc:HGNC:10658] | 4954 | 0.172 | 0.1152 | No |
| 53 | IER5 | immediate early response 5 [Source:HGNC Symbol;Acc:HGNC:5393] | 5163 | 0.154 | 0.1036 | No |
| 54 | SLC3A2 | solute carrier family 3 member 2 [Source:HGNC Symbol;Acc:HGNC:11026] | 5375 | 0.137 | 0.0917 | No |
| 55 | SLC7A11 | solute carrier family 7 member 11 [Source:HGNC Symbol;Acc:HGNC:11059] | 5535 | 0.125 | 0.0829 | No |
| 56 | ABCC5 | ATP binding cassette subfamily C member 5 [Source:HGNC Symbol;Acc:HGNC:56] | 5602 | 0.119 | 0.0801 | No |
| 57 | ANKRA2 | ankyrin repeat family A member 2 [Source:HGNC Symbol;Acc:HGNC:13208] | 5718 | 0.111 | 0.0741 | No |
| 58 | DNTTIP2 | deoxynucleotidyltransferase terminal interacting protein 2 [Source:HGNC Symbol;Acc:HGNC:24013] | 5764 | 0.108 | 0.0725 | No |
| 59 | TXNIP | thioredoxin interacting protein [Source:HGNC Symbol;Acc:HGNC:16952] | 5781 | 0.106 | 0.0728 | No |
| 60 | KIF13B | kinesin family member 13B [Source:HGNC Symbol;Acc:HGNC:14405] | 5821 | 0.104 | 0.0715 | No |
| 61 | SERTAD3 | SERTA domain containing 3 [Source:HGNC Symbol;Acc:HGNC:17931] | 5913 | 0.098 | 0.0668 | No |
| 62 | HDAC3 | histone deacetylase 3 [Source:HGNC Symbol;Acc:HGNC:4854] | 5982 | 0.093 | 0.0636 | No |
| 63 | TGFB1 | transforming growth factor beta 1 [Source:HGNC Symbol;Acc:HGNC:11766] | 6038 | 0.088 | 0.0611 | No |
| 64 | ZFP36L1 | ZFP36 ring finger protein like 1 [Source:HGNC Symbol;Acc:HGNC:1107] | 6057 | 0.086 | 0.0610 | No |
| 65 | RB1 | RB transcriptional corepressor 1 [Source:HGNC Symbol;Acc:HGNC:9884] | 6141 | 0.080 | 0.0566 | No |
| 66 | SAT1 | spermidine/spermine N1-acetyltransferase 1 [Source:HGNC Symbol;Acc:HGNC:10540] | 6202 | 0.076 | 0.0537 | No |
| 67 | STOM | stomatin [Source:HGNC Symbol;Acc:HGNC:3383] | 6209 | 0.076 | 0.0542 | No |
| 68 | PCNA | proliferating cell nuclear antigen [Source:HGNC Symbol;Acc:HGNC:8729] | 6214 | 0.075 | 0.0549 | No |
| 69 | SP1 | Sp1 transcription factor [Source:HGNC Symbol;Acc:HGNC:11205] | 6235 | 0.074 | 0.0545 | No |
| 70 | RRAD | "RRAD, Ras related glycolysis inhibitor and calcium channel regulator [Source:HGNC Symbol;Acc:HGNC:10446]" | 6449 | 0.058 | 0.0415 | No |
| 71 | TPD52L1 | TPD52 like 1 [Source:HGNC Symbol;Acc:HGNC:12006] | 6452 | 0.058 | 0.0420 | No |
| 72 | RHBDF2 | rhomboid 5 homolog 2 [Source:HGNC Symbol;Acc:HGNC:20788] | 6617 | 0.048 | 0.0320 | No |
| 73 | MKNK2 | MAPK interacting serine/threonine kinase 2 [Source:HGNC Symbol;Acc:HGNC:7111] | 6739 | 0.041 | 0.0247 | No |
| 74 | NOL8 | nucleolar protein 8 [Source:HGNC Symbol;Acc:HGNC:23387] | 6744 | 0.040 | 0.0249 | No |
| 75 | FBXW7 | F-box and WD repeat domain containing 7 [Source:HGNC Symbol;Acc:HGNC:16712] | 6880 | 0.031 | 0.0166 | No |
| 76 | HMOX1 | heme oxygenase 1 [Source:HGNC Symbol;Acc:HGNC:5013] | 6937 | 0.027 | 0.0133 | No |
| 77 | TRIAP1 | TP53 regulated inhibitor of apoptosis 1 [Source:HGNC Symbol;Acc:HGNC:26937] | 6967 | 0.026 | 0.0118 | No |
| 78 | FOS | "Fos proto-oncogene, AP-1 transcription factor subunit [Source:HGNC Symbol;Acc:HGNC:3796]" | 7164 | 0.014 | -0.0007 | No |
| 79 | H2AW | H2A.W histone [Source:HGNC Symbol;Acc:HGNC:20507] | 7216 | 0.011 | -0.0039 | No |
| 80 | H1-2 | "H1.2 linker histone, cluster member [Source:HGNC Symbol;Acc:HGNC:4716]" | 7276 | 0.008 | -0.0076 | No |
| 81 | NUPR1 | "nuclear protein 1, transcriptional regulator [Source:HGNC Symbol;Acc:HGNC:29990]" | 7282 | 0.008 | -0.0078 | No |
| 82 | LIF | LIF interleukin 6 family cytokine [Source:HGNC Symbol;Acc:HGNC:6596] | 7310 | 0.006 | -0.0095 | No |
| 83 | CCNK | cyclin K [Source:HGNC Symbol;Acc:HGNC:1596] | 7397 | 0.001 | -0.0151 | No |
| 84 | POLH | DNA polymerase eta [Source:HGNC Symbol;Acc:HGNC:9181] | 7443 | -0.001 | -0.0180 | No |
| 85 | BLCAP | BLCAP apoptosis inducing factor [Source:HGNC Symbol;Acc:HGNC:1055] | 7769 | -0.017 | -0.0387 | No |
| 86 | CSRNP2 | cysteine and serine rich nuclear protein 2 [Source:HGNC Symbol;Acc:HGNC:16006] | 7962 | -0.027 | -0.0508 | No |
| 87 | PPP1R15A | protein phosphatase 1 regulatory subunit 15A [Source:HGNC Symbol;Acc:HGNC:14375] | 8019 | -0.030 | -0.0541 | No |
| 88 | PTPN14 | protein tyrosine phosphatase non-receptor type 14 [Source:HGNC Symbol;Acc:HGNC:9647] | 8097 | -0.035 | -0.0586 | No |
| 89 | CCND2 | cyclin D2 [Source:HGNC Symbol;Acc:HGNC:1583] | 8119 | -0.036 | -0.0595 | No |
| 90 | BTG1 | BTG anti-proliferation factor 1 [Source:HGNC Symbol;Acc:HGNC:1130] | 8137 | -0.037 | -0.0602 | No |
| 91 | PRKAB1 | protein kinase AMP-activated non-catalytic subunit beta 1 [Source:HGNC Symbol;Acc:HGNC:9378] | 8158 | -0.038 | -0.0610 | No |
| 92 | SLC35D1 | solute carrier family 35 member D1 [Source:HGNC Symbol;Acc:HGNC:20800] | 8512 | -0.055 | -0.0831 | No |
| 93 | CD82 | CD82 molecule [Source:HGNC Symbol;Acc:HGNC:6210] | 8645 | -0.063 | -0.0909 | No |
| 94 | HBEGF | heparin binding EGF like growth factor [Source:HGNC Symbol;Acc:HGNC:3059] | 8784 | -0.071 | -0.0989 | No |
| 95 | ELP1 | elongator complex protein 1 [Source:HGNC Symbol;Acc:HGNC:5959] | 8823 | -0.074 | -0.1005 | No |
| 96 | RAD9A | RAD9 checkpoint clamp component A [Source:HGNC Symbol;Acc:HGNC:9827] | 9066 | -0.086 | -0.1151 | No |
| 97 | ABHD4 | "abhydrolase domain containing 4, N-acyl phospholipase B [Source:HGNC Symbol;Acc:HGNC:20154]" | 9114 | -0.089 | -0.1170 | No |
| 98 | TPRKB | TP53RK binding protein [Source:HGNC Symbol;Acc:HGNC:24259] | 9181 | -0.092 | -0.1201 | No |
| 99 | RALGDS | ral guanine nucleotide dissociation stimulator [Source:HGNC Symbol;Acc:HGNC:9842] | 9281 | -0.097 | -0.1254 | No |
| 100 | TM7SF3 | transmembrane 7 superfamily member 3 [Source:HGNC Symbol;Acc:HGNC:23049] | 9366 | -0.102 | -0.1295 | No |
| 101 | ISCU | iron-sulfur cluster assembly enzyme [Source:HGNC Symbol;Acc:HGNC:29882] | 9379 | -0.103 | -0.1290 | No |
| 102 | TSC22D1 | TSC22 domain family member 1 [Source:HGNC Symbol;Acc:HGNC:16826] | 9431 | -0.106 | -0.1310 | No |
| 103 | RCHY1 | ring finger and CHY zinc finger domain containing 1 [Source:HGNC Symbol;Acc:HGNC:17479] | 9454 | -0.107 | -0.1311 | No |
| 104 | CEBPA | CCAAT enhancer binding protein alpha [Source:HGNC Symbol;Acc:HGNC:1833] | 9503 | -0.110 | -0.1329 | No |
| 105 | RAP2B | "RAP2B, member of RAS oncogene family [Source:HGNC Symbol;Acc:HGNC:9862]" | 9578 | -0.116 | -0.1362 | No |
| 106 | HEXIM1 | HEXIM P-TEFb complex subunit 1 [Source:HGNC Symbol;Acc:HGNC:24953] | 9704 | -0.125 | -0.1428 | No |
| 107 | TOB1 | "transducer of ERBB2, 1 [Source:HGNC Symbol;Acc:HGNC:11979]" | 9783 | -0.130 | -0.1462 | No |
| 108 | POM121 | POM121 transmembrane nucleoporin [Source:HGNC Symbol;Acc:HGNC:19702] | 9790 | -0.130 | -0.1450 | No |
| 109 | IP6K2 | inositol hexakisphosphate kinase 2 [Source:HGNC Symbol;Acc:HGNC:17313] | 9825 | -0.132 | -0.1456 | No |
| 110 | WWP1 | WW domain containing E3 ubiquitin protein ligase 1 [Source:HGNC Symbol;Acc:HGNC:17004] | 9848 | -0.133 | -0.1454 | No |
| 111 | NINJ1 | ninjurin 1 [Source:HGNC Symbol;Acc:HGNC:7824] | 9852 | -0.134 | -0.1439 | No |
| 112 | RAB40C | "RAB40C, member RAS oncogene family [Source:HGNC Symbol;Acc:HGNC:18285]" | 9882 | -0.135 | -0.1441 | No |
| 113 | JUN | "Jun proto-oncogene, AP-1 transcription factor subunit [Source:HGNC Symbol;Acc:HGNC:6204]" | 10257 | -0.157 | -0.1664 | No |
| 114 | HRAS | "HRas proto-oncogene, GTPase [Source:HGNC Symbol;Acc:HGNC:5173]" | 10331 | -0.161 | -0.1691 | No |
| 115 | KLF4 | Kruppel like factor 4 [Source:HGNC Symbol;Acc:HGNC:6348] | 10415 | -0.167 | -0.1724 | No |
| 116 | CCND3 | cyclin D3 [Source:HGNC Symbol;Acc:HGNC:1585] | 10446 | -0.168 | -0.1723 | No |
| 117 | ADA | adenosine deaminase [Source:HGNC Symbol;Acc:HGNC:186] | 10516 | -0.173 | -0.1746 | No |
| 118 | PPM1D | "protein phosphatase, Mg2+/Mn2+ dependent 1D [Source:HGNC Symbol;Acc:HGNC:9277]" | 10574 | -0.175 | -0.1762 | No |
| 119 | ERCC5 | "ERCC excision repair 5, endonuclease [Source:HGNC Symbol;Acc:HGNC:3437]" | 10670 | -0.181 | -0.1801 | No |
| 120 | MDM2 | MDM2 proto-oncogene [Source:HGNC Symbol;Acc:HGNC:6973] | 10902 | -0.194 | -0.1926 | No |
| 121 | CTSD | cathepsin D [Source:HGNC Symbol;Acc:HGNC:2529] | 11141 | -0.209 | -0.2054 | No |
| 122 | PRMT2 | protein arginine methyltransferase 2 [Source:HGNC Symbol;Acc:HGNC:5186] | 11227 | -0.213 | -0.2083 | No |
| 123 | PLK3 | polo like kinase 3 [Source:HGNC Symbol;Acc:HGNC:2154] | 11238 | -0.214 | -0.2063 | No |
| 124 | TP53 | tumor protein p53 [Source:HGNC Symbol;Acc:HGNC:11998] | 11287 | -0.216 | -0.2068 | No |
| 125 | FUCA1 | alpha-L-fucosidase 1 [Source:HGNC Symbol;Acc:HGNC:4006] | 11365 | -0.221 | -0.2090 | No |
| 126 | HSPA4L | heat shock protein family A (Hsp70) member 4 like [Source:HGNC Symbol;Acc:HGNC:17041] | 11561 | -0.233 | -0.2188 | No |
| 127 | DEF6 | DEF6 guanine nucleotide exchange factor [Source:HGNC Symbol;Acc:HGNC:2760] | 11657 | -0.238 | -0.2220 | No |
| 128 | RRP8 | ribosomal RNA processing 8 [Source:HGNC Symbol;Acc:HGNC:29030] | 11660 | -0.238 | -0.2192 | No |
| 129 | HINT1 | histidine triad nucleotide binding protein 1 [Source:HGNC Symbol;Acc:HGNC:4912] | 11842 | -0.250 | -0.2278 | No |
| 130 | WRAP73 | "WD repeat containing, antisense to TP73 [Source:HGNC Symbol;Acc:HGNC:12759]" | 11881 | -0.253 | -0.2271 | No |
| 131 | PLXNB2 | plexin B2 [Source:HGNC Symbol;Acc:HGNC:9104] | 11915 | -0.254 | -0.2262 | No |
| 132 | SLC19A2 | solute carrier family 19 member 2 [Source:HGNC Symbol;Acc:HGNC:10938] | 11969 | -0.258 | -0.2264 | No |
| 133 | GADD45A | growth arrest and DNA damage inducible alpha [Source:HGNC Symbol;Acc:HGNC:4095] | 12093 | -0.267 | -0.2311 | No |
| 134 | RPS27L | ribosomal protein S27 like [Source:HGNC Symbol;Acc:HGNC:18476] | 12108 | -0.268 | -0.2287 | No |
| 135 | FGF13 | fibroblast growth factor 13 [Source:HGNC Symbol;Acc:HGNC:3670] | 12167 | -0.271 | -0.2291 | No |
| 136 | FDXR | ferredoxin reductase [Source:HGNC Symbol;Acc:HGNC:3642] | 12240 | -0.277 | -0.2303 | No |
| 137 | FOXO3 | forkhead box O3 [Source:HGNC Symbol;Acc:HGNC:3821] | 12304 | -0.281 | -0.2310 | No |
| 138 | JAG2 | jagged canonical Notch ligand 2 [Source:HGNC Symbol;Acc:HGNC:6189] | 12440 | -0.291 | -0.2361 | No |
| 139 | PDGFA | platelet derived growth factor subunit A [Source:HGNC Symbol;Acc:HGNC:8799] | 12465 | -0.293 | -0.2340 | No |
| 140 | PROCR | protein C receptor [Source:HGNC Symbol;Acc:HGNC:9452] | 12467 | -0.293 | -0.2305 | No |
| 141 | H2AJ | H2A.J histone [Source:HGNC Symbol;Acc:HGNC:14456] | 12573 | -0.302 | -0.2336 | No |
| 142 | XPC | "XPC complex subunit, DNA damage recognition and repair factor [Source:HGNC Symbol;Acc:HGNC:12816]" | 12574 | -0.302 | -0.2299 | No |
| 143 | BMP2 | bone morphogenetic protein 2 [Source:HGNC Symbol;Acc:HGNC:1069] | 12618 | -0.305 | -0.2289 | No |
| 144 | BAIAP2 | BAR/IMD domain containing adaptor protein 2 [Source:HGNC Symbol;Acc:HGNC:947] | 12760 | -0.316 | -0.2341 | No |
| 145 | NDRG1 | N-myc downstream regulated 1 [Source:HGNC Symbol;Acc:HGNC:7679] | 12779 | -0.318 | -0.2314 | No |
| 146 | FAM162A | family with sequence similarity 162 member A [Source:HGNC Symbol;Acc:HGNC:17865] | 12830 | -0.321 | -0.2306 | No |
| 147 | IER3 | immediate early response 3 [Source:HGNC Symbol;Acc:HGNC:5392] | 12833 | -0.322 | -0.2268 | No |
| 148 | TSPYL2 | TSPY like 2 [Source:HGNC Symbol;Acc:HGNC:24358] | 12840 | -0.323 | -0.2232 | No |
| 149 | TM4SF1 | transmembrane 4 L six family member 1 [Source:HGNC Symbol;Acc:HGNC:11853] | 12973 | -0.335 | -0.2276 | No |
| 150 | PIDD1 | p53-induced death domain protein 1 [Source:HGNC Symbol;Acc:HGNC:16491] | 13068 | -0.344 | -0.2295 | No |
| 151 | OSGIN1 | oxidative stress induced growth inhibitor 1 [Source:HGNC Symbol;Acc:HGNC:30093] | 13132 | -0.350 | -0.2293 | No |
| 152 | SEC61A1 | SEC61 translocon subunit alpha 1 [Source:HGNC Symbol;Acc:HGNC:18276] | 13211 | -0.357 | -0.2299 | No |
| 153 | TAX1BP3 | Tax1 binding protein 3 [Source:HGNC Symbol;Acc:HGNC:30684] | 13562 | -0.400 | -0.2476 | No |
| 154 | PMM1 | phosphomannomutase 1 [Source:HGNC Symbol;Acc:HGNC:9114] | 13600 | -0.405 | -0.2450 | No |
| 155 | IRAK1 | interleukin 1 receptor associated kinase 1 [Source:HGNC Symbol;Acc:HGNC:6112] | 13614 | -0.407 | -0.2409 | No |
| 156 | PVT1 | Pvt1 oncogene [Source:HGNC Symbol;Acc:HGNC:9709] | 13677 | -0.414 | -0.2398 | No |
| 157 | S100A10 | S100 calcium binding protein A10 [Source:HGNC Symbol;Acc:HGNC:10487] | 13784 | -0.416 | -0.2415 | No |
| 158 | EI24 | EI24 autophagy associated transmembrane protein [Source:HGNC Symbol;Acc:HGNC:13276] | 13875 | -0.427 | -0.2421 | No |
| 159 | CDK5R1 | cyclin dependent kinase 5 regulatory subunit 1 [Source:HGNC Symbol;Acc:HGNC:1775] | 14006 | -0.433 | -0.2451 | No |
| 160 | PHLDA3 | pleckstrin homology like domain family A member 3 [Source:HGNC Symbol;Acc:HGNC:8934] | 14084 | -0.445 | -0.2447 | No |
| 161 | TRAF4 | TNF receptor associated factor 4 [Source:HGNC Symbol;Acc:HGNC:12034] | 14147 | -0.457 | -0.2430 | No |
| 162 | GLS2 | glutaminase 2 [Source:HGNC Symbol;Acc:HGNC:29570] | 14330 | -0.484 | -0.2489 | Yes |
| 163 | RACK1 | receptor for activated C kinase 1 [Source:HGNC Symbol;Acc:HGNC:4399] | 14370 | -0.489 | -0.2454 | Yes |
| 164 | BAX | "BCL2 associated X, apoptosis regulator [Source:HGNC Symbol;Acc:HGNC:959]" | 14408 | -0.493 | -0.2417 | Yes |
| 165 | KLK8 | kallikrein related peptidase 8 [Source:HGNC Symbol;Acc:HGNC:6369] | 14414 | -0.493 | -0.2359 | Yes |
| 166 | PLK2 | polo like kinase 2 [Source:HGNC Symbol;Acc:HGNC:19699] | 14434 | -0.499 | -0.2310 | Yes |
| 167 | RXRA | retinoid X receptor alpha [Source:HGNC Symbol;Acc:HGNC:10477] | 14541 | -0.522 | -0.2315 | Yes |
| 168 | ZNF365 | zinc finger protein 365 [Source:HGNC Symbol;Acc:HGNC:18194] | 14681 | -0.550 | -0.2337 | Yes |
| 169 | CLCA2 | chloride channel accessory 2 [Source:HGNC Symbol;Acc:HGNC:2016] | 14772 | -0.574 | -0.2324 | Yes |
| 170 | DCXR | dicarbonyl and L-xylulose reductase [Source:HGNC Symbol;Acc:HGNC:18985] | 14777 | -0.576 | -0.2256 | Yes |
| 171 | SESN1 | sestrin 1 [Source:HGNC Symbol;Acc:HGNC:21595] | 14857 | -0.602 | -0.2233 | Yes |
| 172 | RPS12 | ribosomal protein S12 [Source:HGNC Symbol;Acc:HGNC:10385] | 14871 | -0.606 | -0.2167 | Yes |
| 173 | RETSAT | retinol saturase [Source:HGNC Symbol;Acc:HGNC:25991] | 14885 | -0.613 | -0.2100 | Yes |
| 174 | RPL18 | ribosomal protein L18 [Source:HGNC Symbol;Acc:HGNC:10310] | 14911 | -0.624 | -0.2040 | Yes |
| 175 | ABAT | 4-aminobutyrate aminotransferase [Source:HGNC Symbol;Acc:HGNC:23] | 14915 | -0.625 | -0.1965 | Yes |
| 176 | AEN | apoptosis enhancing nuclease [Source:HGNC Symbol;Acc:HGNC:25722] | 14938 | -0.638 | -0.1901 | Yes |
| 177 | CCNG1 | cyclin G1 [Source:HGNC Symbol;Acc:HGNC:1592] | 14944 | -0.640 | -0.1825 | Yes |
| 178 | MXD4 | MAX dimerization protein 4 [Source:HGNC Symbol;Acc:HGNC:13906] | 15053 | -0.691 | -0.1810 | Yes |
| 179 | RPL36 | ribosomal protein L36 [Source:HGNC Symbol;Acc:HGNC:13631] | 15082 | -0.708 | -0.1741 | Yes |
| 180 | EPHA2 | EPH receptor A2 [Source:HGNC Symbol;Acc:HGNC:3386] | 15102 | -0.719 | -0.1665 | Yes |
| 181 | CTSF | cathepsin F [Source:HGNC Symbol;Acc:HGNC:2531] | 15187 | -0.771 | -0.1624 | Yes |
| 182 | TNFSF9 | TNF superfamily member 9 [Source:HGNC Symbol;Acc:HGNC:11939] | 15266 | -0.818 | -0.1574 | Yes |
| 183 | ALOX15B | arachidonate 15-lipoxygenase type B [Source:HGNC Symbol;Acc:HGNC:434] | 15300 | -0.841 | -0.1492 | Yes |
| 184 | SPHK1 | sphingosine kinase 1 [Source:HGNC Symbol;Acc:HGNC:11240] | 15316 | -0.845 | -0.1398 | Yes |
| 185 | S100A4 | S100 calcium binding protein A4 [Source:HGNC Symbol;Acc:HGNC:10494] | 15340 | -0.855 | -0.1308 | Yes |
| 186 | CD81 | CD81 molecule [Source:HGNC Symbol;Acc:HGNC:1701] | 15353 | -0.863 | -0.1209 | Yes |
| 187 | DDIT4 | DNA damage inducible transcript 4 [Source:HGNC Symbol;Acc:HGNC:24944] | 15359 | -0.865 | -0.1106 | Yes |
| 188 | F2R | coagulation factor II thrombin receptor [Source:HGNC Symbol;Acc:HGNC:3537] | 15375 | -0.870 | -0.1009 | Yes |
| 189 | AK1 | adenylate kinase 1 [Source:HGNC Symbol;Acc:HGNC:361] | 15402 | -0.892 | -0.0916 | Yes |
| 190 | ZMAT3 | zinc finger matrin-type 3 [Source:HGNC Symbol;Acc:HGNC:29983] | 15415 | -0.913 | -0.0811 | Yes |
| 191 | CDKN1A | cyclin dependent kinase inhibitor 1A [Source:HGNC Symbol;Acc:HGNC:1784] | 15428 | -0.929 | -0.0705 | Yes |
| 192 | ITGB4 | integrin subunit beta 4 [Source:HGNC Symbol;Acc:HGNC:6158] | 15452 | -0.960 | -0.0602 | Yes |
| 193 | CDH13 | cadherin 13 [Source:HGNC Symbol;Acc:HGNC:1753] | 15514 | -1.064 | -0.0510 | Yes |
| 194 | TGFA | transforming growth factor alpha [Source:HGNC Symbol;Acc:HGNC:11765] | 15521 | -1.084 | -0.0381 | Yes |
| 195 | CDKN2B | cyclin dependent kinase inhibitor 2B [Source:HGNC Symbol;Acc:HGNC:1788] | 15551 | -1.144 | -0.0259 | Yes |
| 196 | EPHX1 | epoxide hydrolase 1 [Source:HGNC Symbol;Acc:HGNC:3401] | 15605 | -1.355 | -0.0127 | Yes |
| 197 | INHBB | inhibin subunit beta B [Source:HGNC Symbol;Acc:HGNC:6067] | 15606 | -1.356 | 0.0040 | Yes |
Table: GSEA details [plain text format]

  

Fig 2: HALLMARK\_P53\_PATHWAY      
 Blue-Pink O' Gram in the Space of the Analyzed GeneSet

  

Fig 3: HALLMARK\_P53\_PATHWAY: Random ES distribution      
 Gene set null distribution of ES for **HALLMARK\_P53\_PATHWAY**
