# Supplementary material for: In vivo CRISPR screens reveal Serpinb9 and Adam2 as regulators of immune therapy response in lung cancer
Source: Nat Commun. 2023 May 31;14:3150. doi: 10.1038/s41467-023-38841-7 (PMC10232477; doi:10.1038/s41467-023-38841-7)
Supplement: Supplementary file 13 — Source Data [file 41467_2023_38841_MOESM13_ESM.zip › Figure 4E.zip!/my_analysis.Gsea.1640146340255_Hallmark/HALLMARK_SPERMATOGENESIS.html]

Details for gene set HALLMARK\_SPERMATOGENESIS[GSEA]

|  || Dataset | December 21, 2021\_for GSEA run\_collapsed\_to\_symbols.GSEA\_B6\_NI.cls #CTLR\_NI\_versus\_ADAM2\_NI |
| Phenotype | GSEA\_B6\_NI.cls#CTLR\_NI\_versus\_ADAM2\_NI |
| Upregulated in class | CTLR\_NI |
| GeneSet | HALLMARK\_SPERMATOGENESIS |
| Enrichment Score (ES) | 0.31909359 |
| Normalized Enrichment Score (NES) | 1.1674615 |
| Nominal p-value | 0.10788382 |
| FDR q-value | 0.6231723 |
| FWER p-Value | 1.0 |
Table: GSEA Results Summary

  

Fig 1: Enrichment plot: HALLMARK\_SPERMATOGENESIS      
 Profile of the Running ES Score & Positions of GeneSet Members on the Rank Ordered List

  

| SYMBOL | TITLE | RANK IN GENE LIST | RANK METRIC SCORE | RUNNING ES | CORE ENRICHMENT || 1 | CLGN | calmegin [Source:HGNC Symbol;Acc:HGNC:2060] | 73 | 1.898 | 0.0335 | Yes |
| 2 | IL12RB2 | interleukin 12 receptor subunit beta 2 [Source:HGNC Symbol;Acc:HGNC:5972] | 109 | 1.769 | 0.0669 | Yes |
| 3 | MAP7 | microtubule associated protein 7 [Source:HGNC Symbol;Acc:HGNC:6869] | 282 | 1.335 | 0.0827 | Yes |
| 4 | IL13RA2 | interleukin 13 receptor subunit alpha 2 [Source:HGNC Symbol;Acc:HGNC:5975] | 311 | 1.279 | 0.1066 | Yes |
| 5 | DDX25 | DEAD-box helicase 25 [Source:HGNC Symbol;Acc:HGNC:18698] | 357 | 1.220 | 0.1283 | Yes |
| 6 | ACE | angiotensin I converting enzyme [Source:HGNC Symbol;Acc:HGNC:2707] | 499 | 1.074 | 0.1409 | Yes |
| 7 | CAMK4 | calcium/calmodulin dependent protein kinase IV [Source:HGNC Symbol;Acc:HGNC:1464] | 520 | 1.048 | 0.1607 | Yes |
| 8 | H1-6 | "H1.6 linker histone, cluster member [Source:HGNC Symbol;Acc:HGNC:4720]" | 613 | 0.987 | 0.1746 | Yes |
| 9 | SLC2A5 | solute carrier family 2 member 5 [Source:HGNC Symbol;Acc:HGNC:11010] | 656 | 0.958 | 0.1912 | Yes |
| 10 | ALOX15 | arachidonate 15-lipoxygenase [Source:HGNC Symbol;Acc:HGNC:433] | 690 | 0.938 | 0.2080 | Yes |
| 11 | DPEP3 | dipeptidase 3 [Source:HGNC Symbol;Acc:HGNC:23029] | 699 | 0.934 | 0.2262 | Yes |
| 12 | CCT6B | chaperonin containing TCP1 subunit 6B [Source:HGNC Symbol;Acc:HGNC:1621] | 709 | 0.927 | 0.2443 | Yes |
| 13 | TSSK2 | testis specific serine kinase 2 [Source:HGNC Symbol;Acc:HGNC:11401] | 765 | 0.892 | 0.2587 | Yes |
| 14 | GAPDHS | "glyceraldehyde-3-phosphate dehydrogenase, spermatogenic [Source:HGNC Symbol;Acc:HGNC:24864]" | 1244 | 0.717 | 0.2424 | Yes |
| 15 | CHRM4 | cholinergic receptor muscarinic 4 [Source:HGNC Symbol;Acc:HGNC:1953] | 1368 | 0.670 | 0.2480 | Yes |
| 16 | IFT88 | intraflagellar transport 88 [Source:HGNC Symbol;Acc:HGNC:20606] | 1476 | 0.635 | 0.2539 | Yes |
| 17 | PHF7 | PHD finger protein 7 [Source:HGNC Symbol;Acc:HGNC:18458] | 1556 | 0.608 | 0.2611 | Yes |
| 18 | PARP2 | poly(ADP-ribose) polymerase 2 [Source:HGNC Symbol;Acc:HGNC:272] | 1912 | 0.521 | 0.2488 | Yes |
| 19 | SCG5 | secretogranin V [Source:HGNC Symbol;Acc:HGNC:10816] | 1980 | 0.507 | 0.2547 | Yes |
| 20 | NEFH | neurofilament heavy [Source:HGNC Symbol;Acc:HGNC:7737] | 1993 | 0.505 | 0.2640 | Yes |
| 21 | SPATA6 | spermatogenesis associated 6 [Source:HGNC Symbol;Acc:HGNC:18309] | 2082 | 0.494 | 0.2683 | Yes |
| 22 | SLC12A2 | solute carrier family 12 member 2 [Source:HGNC Symbol;Acc:HGNC:10911] | 2317 | 0.466 | 0.2627 | Yes |
| 23 | DMRT1 | doublesex and mab-3 related transcription factor 1 [Source:HGNC Symbol;Acc:HGNC:2934] | 2532 | 0.445 | 0.2579 | Yes |
| 24 | TTK | TTK protein kinase [Source:HGNC Symbol;Acc:HGNC:12401] | 2550 | 0.443 | 0.2657 | Yes |
| 25 | ART3 | ADP-ribosyltransferase 3 (inactive) [Source:HGNC Symbol;Acc:HGNC:725] | 2617 | 0.431 | 0.2701 | Yes |
| 26 | ACRV1 | acrosomal vesicle protein 1 [Source:HGNC Symbol;Acc:HGNC:127] | 2678 | 0.426 | 0.2748 | Yes |
| 27 | LDHC | lactate dehydrogenase C [Source:HGNC Symbol;Acc:HGNC:6544] | 2845 | 0.411 | 0.2724 | Yes |
| 28 | SNAP91 | synaptosome associated protein 91 [Source:HGNC Symbol;Acc:HGNC:14986] | 2880 | 0.411 | 0.2785 | Yes |
| 29 | ADCYAP1 | adenylate cyclase activating polypeptide 1 [Source:HGNC Symbol;Acc:HGNC:241] | 2898 | 0.411 | 0.2857 | Yes |
| 30 | CRISP2 | cysteine rich secretory protein 2 [Source:HGNC Symbol;Acc:HGNC:12024] | 2920 | 0.411 | 0.2927 | Yes |
| 31 | BUB1 | BUB1 mitotic checkpoint serine/threonine kinase [Source:HGNC Symbol;Acc:HGNC:1148] | 2974 | 0.409 | 0.2975 | Yes |
| 32 | CNIH2 | cornichon family AMPA receptor auxiliary protein 2 [Source:HGNC Symbol;Acc:HGNC:28744] | 2994 | 0.405 | 0.3044 | Yes |
| 33 | RFC4 | replication factor C subunit 4 [Source:HGNC Symbol;Acc:HGNC:9972] | 3006 | 0.403 | 0.3118 | Yes |
| 34 | PSMG1 | proteasome assembly chaperone 1 [Source:HGNC Symbol;Acc:HGNC:3043] | 3053 | 0.394 | 0.3168 | Yes |
| 35 | PHKG2 | phosphorylase kinase catalytic subunit gamma 2 [Source:HGNC Symbol;Acc:HGNC:8931] | 3147 | 0.380 | 0.3185 | Yes |
| 36 | DDX4 | DEAD-box helicase 4 [Source:HGNC Symbol;Acc:HGNC:18700] | 3346 | 0.350 | 0.3128 | Yes |
| 37 | AGFG1 | ArfGAP with FG repeats 1 [Source:HGNC Symbol;Acc:HGNC:5175] | 3358 | 0.348 | 0.3191 | Yes |
| 38 | SIRT1 | sirtuin 1 [Source:HGNC Symbol;Acc:HGNC:14929] | 3694 | 0.302 | 0.3036 | No |
| 39 | ARL4A | ADP ribosylation factor like GTPase 4A [Source:HGNC Symbol;Acc:HGNC:695] | 3817 | 0.289 | 0.3016 | No |
| 40 | HSPA2 | heat shock protein family A (Hsp70) member 2 [Source:HGNC Symbol;Acc:HGNC:5235] | 4234 | 0.239 | 0.2797 | No |
| 41 | DBF4 | DBF4 zinc finger [Source:HGNC Symbol;Acc:HGNC:17364] | 4451 | 0.218 | 0.2702 | No |
| 42 | PIAS2 | protein inhibitor of activated STAT 2 [Source:HGNC Symbol;Acc:HGNC:17311] | 4502 | 0.213 | 0.2712 | No |
| 43 | YBX2 | Y-box binding protein 2 [Source:HGNC Symbol;Acc:HGNC:17948] | 4648 | 0.201 | 0.2659 | No |
| 44 | ZPBP | zona pellucida binding protein [Source:HGNC Symbol;Acc:HGNC:15662] | 4741 | 0.193 | 0.2639 | No |
| 45 | TULP2 | TUB like protein 2 [Source:HGNC Symbol;Acc:HGNC:12424] | 4796 | 0.188 | 0.2642 | No |
| 46 | TCP11 | t-complex 11 [Source:HGNC Symbol;Acc:HGNC:11658] | 4813 | 0.187 | 0.2670 | No |
| 47 | DMC1 | DNA meiotic recombinase 1 [Source:HGNC Symbol;Acc:HGNC:2927] | 4833 | 0.185 | 0.2695 | No |
| 48 | NCAPH | non-SMC condensin I complex subunit H [Source:HGNC Symbol;Acc:HGNC:1112] | 4969 | 0.171 | 0.2642 | No |
| 49 | NAA11 | "N-alpha-acetyltransferase 11, NatA catalytic subunit [Source:HGNC Symbol;Acc:HGNC:28125]" | 5150 | 0.154 | 0.2557 | No |
| 50 | CDKN3 | cyclin dependent kinase inhibitor 3 [Source:HGNC Symbol;Acc:HGNC:1791] | 5225 | 0.148 | 0.2540 | No |
| 51 | MLF1 | myeloid leukemia factor 1 [Source:HGNC Symbol;Acc:HGNC:7125] | 5259 | 0.146 | 0.2548 | No |
| 52 | CHFR | checkpoint with forkhead and ring finger domains [Source:HGNC Symbol;Acc:HGNC:20455] | 5287 | 0.143 | 0.2559 | No |
| 53 | NEK2 | NIMA related kinase 2 [Source:HGNC Symbol;Acc:HGNC:7745] | 5335 | 0.140 | 0.2557 | No |
| 54 | TOPBP1 | DNA topoisomerase II binding protein 1 [Source:HGNC Symbol;Acc:HGNC:17008] | 5347 | 0.139 | 0.2578 | No |
| 55 | MLLT10 | MLLT10 histone lysine methyltransferase DOT1L cofactor [Source:HGNC Symbol;Acc:HGNC:16063] | 5800 | 0.105 | 0.2309 | No |
| 56 | PGS1 | phosphatidylglycerophosphate synthase 1 [Source:HGNC Symbol;Acc:HGNC:30029] | 5832 | 0.103 | 0.2310 | No |
| 57 | GFI1 | growth factor independent 1 transcriptional repressor [Source:HGNC Symbol;Acc:HGNC:4237] | 5880 | 0.100 | 0.2299 | No |
| 58 | AURKA | aurora kinase A [Source:HGNC Symbol;Acc:HGNC:11393] | 5931 | 0.097 | 0.2287 | No |
| 59 | ACRBP | acrosin binding protein [Source:HGNC Symbol;Acc:HGNC:17195] | 6224 | 0.075 | 0.2114 | No |
| 60 | TNNI3 | "troponin I3, cardiac type [Source:HGNC Symbol;Acc:HGNC:11947]" | 6308 | 0.069 | 0.2075 | No |
| 61 | CDK1 | cyclin dependent kinase 1 [Source:HGNC Symbol;Acc:HGNC:1722] | 6401 | 0.062 | 0.2028 | No |
| 62 | RAD17 | RAD17 checkpoint clamp loader component [Source:HGNC Symbol;Acc:HGNC:9807] | 6708 | 0.042 | 0.1840 | No |
| 63 | TSN | translin [Source:HGNC Symbol;Acc:HGNC:12379] | 6852 | 0.033 | 0.1755 | No |
| 64 | SCG3 | secretogranin III [Source:HGNC Symbol;Acc:HGNC:13707] | 6953 | 0.026 | 0.1696 | No |
| 65 | EZH2 | enhancer of zeste 2 polycomb repressive complex 2 subunit [Source:HGNC Symbol;Acc:HGNC:3527] | 7580 | -0.008 | 0.1295 | No |
| 66 | GPR182 | G protein-coupled receptor 182 [Source:HGNC Symbol;Acc:HGNC:13708] | 7876 | -0.022 | 0.1110 | No |
| 67 | RPL39L | ribosomal protein L39 like [Source:HGNC Symbol;Acc:HGNC:17094] | 8011 | -0.029 | 0.1029 | No |
| 68 | NPY5R | neuropeptide Y receptor Y5 [Source:HGNC Symbol;Acc:HGNC:7958] | 8263 | -0.042 | 0.0876 | No |
| 69 | PCSK4 | proprotein convertase subtilisin/kexin type 4 [Source:HGNC Symbol;Acc:HGNC:8746] | 8291 | -0.043 | 0.0868 | No |
| 70 | BRAF | "B-Raf proto-oncogene, serine/threonine kinase [Source:HGNC Symbol;Acc:HGNC:1097]" | 8347 | -0.046 | 0.0842 | No |
| 71 | VDAC3 | voltage dependent anion channel 3 [Source:HGNC Symbol;Acc:HGNC:12674] | 8705 | -0.067 | 0.0626 | No |
| 72 | GMCL1 | "germ cell-less 1, spermatogenesis associated [Source:HGNC Symbol;Acc:HGNC:23843]" | 8845 | -0.075 | 0.0551 | No |
| 73 | CCNB2 | cyclin B2 [Source:HGNC Symbol;Acc:HGNC:1580] | 8869 | -0.076 | 0.0552 | No |
| 74 | PDHA2 | pyruvate dehydrogenase E1 subunit alpha 2 [Source:HGNC Symbol;Acc:HGNC:8807] | 8915 | -0.078 | 0.0539 | No |
| 75 | KIF2C | kinesin family member 2C [Source:HGNC Symbol;Acc:HGNC:6393] | 9097 | -0.088 | 0.0440 | No |
| 76 | PAPOLB | poly(A) polymerase beta [Source:HGNC Symbol;Acc:HGNC:15970] | 9374 | -0.103 | 0.0283 | No |
| 77 | ZC3H14 | zinc finger CCCH-type containing 14 [Source:HGNC Symbol;Acc:HGNC:20509] | 9932 | -0.138 | -0.0047 | No |
| 78 | MTOR | mechanistic target of rapamycin kinase [Source:HGNC Symbol;Acc:HGNC:3942] | 10094 | -0.147 | -0.0121 | No |
| 79 | STAM2 | signal transducing adaptor molecule 2 [Source:HGNC Symbol;Acc:HGNC:11358] | 10125 | -0.149 | -0.0110 | No |
| 80 | CSNK2A2 | casein kinase 2 alpha 2 [Source:HGNC Symbol;Acc:HGNC:2459] | 10140 | -0.150 | -0.0089 | No |
| 81 | TEKT2 | tektin 2 [Source:HGNC Symbol;Acc:HGNC:11725] | 10207 | -0.155 | -0.0100 | No |
| 82 | STRBP | spermatid perinuclear RNA binding protein [Source:HGNC Symbol;Acc:HGNC:16462] | 10249 | -0.156 | -0.0095 | No |
| 83 | GSG1 | germ cell associated 1 [Source:HGNC Symbol;Acc:HGNC:19716] | 10311 | -0.160 | -0.0102 | No |
| 84 | COIL | coilin [Source:HGNC Symbol;Acc:HGNC:2184] | 10726 | -0.184 | -0.0331 | No |
| 85 | OAZ3 | ornithine decarboxylase antizyme 3 [Source:HGNC Symbol;Acc:HGNC:8097] | 10752 | -0.186 | -0.0310 | No |
| 86 | LPIN1 | lipin 1 [Source:HGNC Symbol;Acc:HGNC:13345] | 10845 | -0.191 | -0.0331 | No |
| 87 | TKTL1 | transketolase like 1 [Source:HGNC Symbol;Acc:HGNC:11835] | 11399 | -0.224 | -0.0641 | No |
| 88 | HSPA4L | heat shock protein family A (Hsp70) member 4 like [Source:HGNC Symbol;Acc:HGNC:17041] | 11561 | -0.233 | -0.0698 | No |
| 89 | TALDO1 | transaldolase 1 [Source:HGNC Symbol;Acc:HGNC:11559] | 11575 | -0.234 | -0.0659 | No |
| 90 | PEBP1 | phosphatidylethanolamine binding protein 1 [Source:HGNC Symbol;Acc:HGNC:8630] | 11650 | -0.238 | -0.0659 | No |
| 91 | PRKAR2A | protein kinase cAMP-dependent type II regulatory subunit alpha [Source:HGNC Symbol;Acc:HGNC:9391] | 11992 | -0.260 | -0.0826 | No |
| 92 | ODF1 | outer dense fiber of sperm tails 1 [Source:HGNC Symbol;Acc:HGNC:8113] | 12114 | -0.268 | -0.0850 | No |
| 93 | TLE4 | "TLE family member 4, transcriptional corepressor [Source:HGNC Symbol;Acc:HGNC:11840]" | 12141 | -0.270 | -0.0812 | No |
| 94 | NPHP1 | nephrocystin 1 [Source:HGNC Symbol;Acc:HGNC:7905] | 12179 | -0.272 | -0.0781 | No |
| 95 | NOS1 | nitric oxide synthase 1 [Source:HGNC Symbol;Acc:HGNC:7872] | 12230 | -0.275 | -0.0758 | No |
| 96 | NF2 | neurofibromin 2 [Source:HGNC Symbol;Acc:HGNC:7773] | 12234 | -0.276 | -0.0704 | No |
| 97 | TNP2 | transition protein 2 [Source:HGNC Symbol;Acc:HGNC:11952] | 12306 | -0.281 | -0.0693 | No |
| 98 | ELOVL3 | ELOVL fatty acid elongase 3 [Source:HGNC Symbol;Acc:HGNC:18047] | 12423 | -0.290 | -0.0709 | No |
| 99 | JAM3 | junctional adhesion molecule 3 [Source:HGNC Symbol;Acc:HGNC:15532] | 12587 | -0.303 | -0.0753 | No |
| 100 | IDE | insulin degrading enzyme [Source:HGNC Symbol;Acc:HGNC:5381] | 12846 | -0.323 | -0.0854 | No |
| 101 | SHE | Src homology 2 domain containing E [Source:HGNC Symbol;Acc:HGNC:27004] | 12935 | -0.332 | -0.0844 | No |
| 102 | SEPTIN4 | septin 4 [Source:HGNC Symbol;Acc:HGNC:9165] | 13060 | -0.344 | -0.0854 | No |
| 103 | MAST2 | microtubule associated serine/threonine kinase 2 [Source:HGNC Symbol;Acc:HGNC:19035] | 13114 | -0.348 | -0.0818 | No |
| 104 | IP6K1 | inositol hexakisphosphate kinase 1 [Source:HGNC Symbol;Acc:HGNC:18360] | 13224 | -0.358 | -0.0816 | No |
| 105 | GRM8 | glutamate metabotropic receptor 8 [Source:HGNC Symbol;Acc:HGNC:4600] | 14309 | -0.483 | -0.1416 | No |
| 106 | HSPA1L | heat shock protein family A (Hsp70) member 1 like [Source:HGNC Symbol;Acc:HGNC:5234] | 14359 | -0.488 | -0.1349 | No |
| 107 | CLPB | caseinolytic mitochondrial matrix peptidase chaperone subunit B [Source:HGNC Symbol;Acc:HGNC:30664] | 14565 | -0.526 | -0.1375 | No |
| 108 | POMC | proopiomelanocortin [Source:HGNC Symbol;Acc:HGNC:9201] | 14577 | -0.528 | -0.1276 | No |
| 109 | ZC2HC1C | zinc finger C2HC-type containing 1C [Source:HGNC Symbol;Acc:HGNC:20354] | 14755 | -0.568 | -0.1275 | No |
| 110 | PACRG | parkin coregulated [Source:HGNC Symbol;Acc:HGNC:19152] | 14954 | -0.645 | -0.1273 | No |
| 111 | CFTR | CF transmembrane conductance regulator [Source:HGNC Symbol;Acc:HGNC:1884] | 15343 | -0.856 | -0.1350 | No |
| 112 | CLVS1 | clavesin 1 [Source:HGNC Symbol;Acc:HGNC:23139] | 15392 | -0.882 | -0.1203 | No |
| 113 | GSTM3 | glutathione S-transferase mu 3 [Source:HGNC Symbol;Acc:HGNC:4635] | 15393 | -0.884 | -0.1025 | No |
| 114 | GAD1 | glutamate decarboxylase 1 [Source:HGNC Symbol;Acc:HGNC:4092] | 15459 | -0.969 | -0.0872 | No |
| 115 | ADAM2 | ADAM metallopeptidase domain 2 [Source:HGNC Symbol;Acc:HGNC:198] | 15668 | -4.996 | -0.0000 | No |
Table: GSEA details [plain text format]

  

Fig 2: HALLMARK\_SPERMATOGENESIS      
 Blue-Pink O' Gram in the Space of the Analyzed GeneSet

  

Fig 3: HALLMARK\_SPERMATOGENESIS: Random ES distribution      
 Gene set null distribution of ES for **HALLMARK\_SPERMATOGENESIS**
