# Supplementary material for: In vivo CRISPR screens reveal Serpinb9 and Adam2 as regulators of immune therapy response in lung cancer
Source: Nat Commun. 2023 May 31;14:3150. doi: 10.1038/s41467-023-38841-7 (PMC10232477; doi:10.1038/s41467-023-38841-7)
Supplement: Supplementary file 13 — Source Data [file 41467_2023_38841_MOESM13_ESM.zip › Figure 4E.zip!/my_analysis.Gsea.1640146340255_Hallmark/index.html]

Index for xtools.gsea.Gsea my\_analysis.Gsea.1640146340255

### GSEA Report for Dataset December 21, 2021\_for GSEA run

#### Enrichment in phenotype: **CTLR\_NI (3 samples)**

- 33 / 50 gene sets are upregulated in phenotype **CTLR\_NI**- 5 gene sets are significant at FDR < 25%- 10 gene sets are significantly enriched at nominal pvalue < 1%- 10 gene sets are significantly enriched at nominal pvalue < 5%- Snapshot of enrichment results- Detailed enrichment results in html format- Detailed enrichment results in TSV format (tab delimited text)- Guide to interpret results

#### Enrichment in phenotype: **ADAM2\_NI (3 samples)**

- 17 / 50 gene sets are upregulated in phenotype **ADAM2\_NI**- 0 gene sets are significantly enriched at FDR < 25%- 2 gene sets are significantly enriched at nominal pvalue < 1%- 2 gene sets are significantly enriched at nominal pvalue < 5%- Snapshot of enrichment results- Detailed enrichment results in html format- Detailed enrichment results in TSV format (tab delimited text)- Guide to interpret results

#### Dataset details

- The dataset has 27358 native features- After collapsing features into gene symbols, there are: 15669 genes

#### Gene set details

- Gene set size filters (min=15, max=500) resulted in filtering out 0 / 50 gene sets- The remaining 50 gene sets were used in the analysis- List of gene sets used and their sizes (restricted to features in the specified dataset)

#### Gene markers for the **CTLR\_NI** *versus* **ADAM2\_NI** comparison

- The dataset has 15669 features (genes)- # of markers for phenotype **CTLR\_NI**: 7415 (47.3% ) with correlation area 55.6%- # of markers for phenotype **ADAM2\_NI**: 8254 (52.7% ) with correlation area 44.4%- Detailed rank ordered gene list for all features in the dataset- Heat map and gene list correlation  profile for all features in the dataset- Butterfly plot of significant genes

#### Global statistics and plots

- Plot of p-values *vs.* NES- Global ES histogram

#### Other

- Parameters used for this analysis

#### Comments

- Timestamp used as random seed: 1640146340255

#### Warnings

- Phenotype permutation was performed but the number of samples in class A is < 7, phenotype: GSEA\_B6\_NI.cls#CTLR\_NI\_versus\_ADAM2\_NI- Phenotype permutation was performed but the number of samples in class B is < 7, phenotype: GSEA\_B6\_NI.cls#CTLR\_NI\_versus\_ADAM2\_NI- With small datasets, there might not be enough random permutations of sample labels to generate a sufficient null distribution. In such cases, gene\_set randomization might be a better choice.

#### Citing GSEA and MSigDB

To cite your use of the GSEA software please reference the following:

- Subramanian, A., Tamayo, P., et al. (2005, PNAS). - Mootha, V. K., Lindgren, C. M., et al. (2003, Nature Genetics).

For use of the Molecular Signatures Database (MSigDB), to cite please reference   
one or more of the following as appropriate, along with the source for the gene set as listed on the gene set page:

- Liberzon A, et al. (Bioinformatics, 2011). - Liberzon A, et al. (Cell Systems 2015).

---

Report: my\_analysis.Gsea.1640146340255.rpt   by user: dzanadervovic

xtools.gsea.Gsea [Tue, Dec 21, '21 11 PM 12]

Website: www.gsea-msigdb.org/gsea
Questions & Suggestions: Contact page
