# Supplementary material for: In vivo CRISPR screens reveal Serpinb9 and Adam2 as regulators of immune therapy response in lung cancer
Source: Nat Commun. 2023 May 31;14:3150. doi: 10.1038/s41467-023-38841-7 (PMC10232477; doi:10.1038/s41467-023-38841-7)
Supplement: Supplementary file 13 — Source Data [file 41467_2023_38841_MOESM13_ESM.zip › Figure 4E.zip!/my_analysis.Gsea.1640146340255_Hallmark/HALLMARK_OXIDATIVE_PHOSPHORYLATION.html]

Details for gene set HALLMARK\_OXIDATIVE\_PHOSPHORYLATION[GSEA]

|  || Dataset | December 21, 2021\_for GSEA run\_collapsed\_to\_symbols.GSEA\_B6\_NI.cls #CTLR\_NI\_versus\_ADAM2\_NI |
| Phenotype | GSEA\_B6\_NI.cls#CTLR\_NI\_versus\_ADAM2\_NI |
| Upregulated in class | ADAM2\_NI |
| GeneSet | HALLMARK\_OXIDATIVE\_PHOSPHORYLATION |
| Enrichment Score (ES) | -0.30240968 |
| Normalized Enrichment Score (NES) | -0.88918847 |
| Nominal p-value | 0.68244576 |
| FDR q-value | 0.9648606 |
| FWER p-Value | 1.0 |
Table: GSEA Results Summary

  

Fig 1: Enrichment plot: HALLMARK\_OXIDATIVE\_PHOSPHORYLATION      
 Profile of the Running ES Score & Positions of GeneSet Members on the Rank Ordered List

  

| SYMBOL | TITLE | RANK IN GENE LIST | RANK METRIC SCORE | RUNNING ES | CORE ENRICHMENT || 1 | MAOB | monoamine oxidase B [Source:HGNC Symbol;Acc:HGNC:6834] | 535 | 1.034 | -0.0111 | No |
| 2 | PHYH | phytanoyl-CoA 2-hydroxylase [Source:HGNC Symbol;Acc:HGNC:8940] | 665 | 0.955 | 0.0021 | No |
| 3 | LDHB | lactate dehydrogenase B [Source:HGNC Symbol;Acc:HGNC:6541] | 687 | 0.938 | 0.0220 | No |
| 4 | COX17 | cytochrome c oxidase copper chaperone COX17 [Source:HGNC Symbol;Acc:HGNC:2264] | 723 | 0.920 | 0.0406 | No |
| 5 | CASP7 | caspase 7 [Source:HGNC Symbol;Acc:HGNC:1508] | 1011 | 0.810 | 0.0404 | No |
| 6 | PDK4 | pyruvate dehydrogenase kinase 4 [Source:HGNC Symbol;Acc:HGNC:8812] | 1755 | 0.554 | 0.0050 | No |
| 7 | TCIRG1 | "T cell immune regulator 1, ATPase H+ transporting V0 subunit a3 [Source:HGNC Symbol;Acc:HGNC:11647]" | 2822 | 0.412 | -0.0546 | No |
| 8 | MTRF1 | mitochondrial translation release factor 1 [Source:HGNC Symbol;Acc:HGNC:7469] | 2998 | 0.404 | -0.0567 | No |
| 9 | SUCLA2 | succinate-CoA ligase ADP-forming subunit beta [Source:HGNC Symbol;Acc:HGNC:11448] | 3562 | 0.320 | -0.0859 | No |
| 10 | NQO2 | N-ribosyldihydronicotinamide:quinone reductase 2 [Source:HGNC Symbol;Acc:HGNC:7856] | 3569 | 0.319 | -0.0790 | No |
| 11 | ATP6V1D | ATPase H+ transporting V1 subunit D [Source:HGNC Symbol;Acc:HGNC:13527] | 3675 | 0.304 | -0.0789 | No |
| 12 | DLD | dihydrolipoamide dehydrogenase [Source:HGNC Symbol;Acc:HGNC:2898] | 3738 | 0.298 | -0.0762 | No |
| 13 | VDAC2 | voltage dependent anion channel 2 [Source:HGNC Symbol;Acc:HGNC:12672] | 3934 | 0.275 | -0.0826 | No |
| 14 | DECR1 | "2,4-dienoyl-CoA reductase 1 [Source:HGNC Symbol;Acc:HGNC:2753]" | 3945 | 0.273 | -0.0770 | No |
| 15 | TOMM70 | translocase of outer mitochondrial membrane 70 [Source:HGNC Symbol;Acc:HGNC:11985] | 4118 | 0.252 | -0.0824 | No |
| 16 | OPA1 | OPA1 mitochondrial dynamin like GTPase [Source:HGNC Symbol;Acc:HGNC:8140] | 4163 | 0.248 | -0.0797 | No |
| 17 | PDHB | pyruvate dehydrogenase E1 subunit beta [Source:HGNC Symbol;Acc:HGNC:8808] | 4175 | 0.246 | -0.0748 | No |
| 18 | ETFA | electron transfer flavoprotein subunit alpha [Source:HGNC Symbol;Acc:HGNC:3481] | 4274 | 0.235 | -0.0758 | No |
| 19 | CYB5A | cytochrome b5 type A [Source:HGNC Symbol;Acc:HGNC:2570] | 4318 | 0.231 | -0.0734 | No |
| 20 | ATP5PO | ATP synthase peripheral stalk subunit OSCP [Source:HGNC Symbol;Acc:HGNC:850] | 4404 | 0.222 | -0.0738 | No |
| 21 | ATP5PF | ATP synthase peripheral stalk subunit F6 [Source:HGNC Symbol;Acc:HGNC:847] | 4567 | 0.207 | -0.0796 | No |
| 22 | HADHB | hydroxyacyl-CoA dehydrogenase trifunctional multienzyme complex subunit beta [Source:HGNC Symbol;Acc:HGNC:4803] | 4666 | 0.199 | -0.0814 | No |
| 23 | GLUD1 | glutamate dehydrogenase 1 [Source:HGNC Symbol;Acc:HGNC:4335] | 4885 | 0.180 | -0.0914 | No |
| 24 | ATP6V1H | ATPase H+ transporting V1 subunit H [Source:HGNC Symbol;Acc:HGNC:18303] | 5288 | 0.143 | -0.1142 | No |
| 25 | CYCS | "cytochrome c, somatic [Source:HGNC Symbol;Acc:HGNC:19986]" | 5529 | 0.125 | -0.1269 | No |
| 26 | NDUFS1 | NADH:ubiquinone oxidoreductase core subunit S1 [Source:HGNC Symbol;Acc:HGNC:7707] | 5607 | 0.119 | -0.1291 | No |
| 27 | OXA1L | OXA1L mitochondrial inner membrane protein [Source:HGNC Symbol;Acc:HGNC:8526] | 5639 | 0.116 | -0.1285 | No |
| 28 | NDUFB4 | NADH:ubiquinone oxidoreductase subunit B4 [Source:HGNC Symbol;Acc:HGNC:7699] | 5657 | 0.115 | -0.1270 | No |
| 29 | HSPA9 | heat shock protein family A (Hsp70) member 9 [Source:HGNC Symbol;Acc:HGNC:5244] | 5752 | 0.109 | -0.1306 | No |
| 30 | PDHX | pyruvate dehydrogenase complex component X [Source:HGNC Symbol;Acc:HGNC:21350] | 5862 | 0.102 | -0.1354 | No |
| 31 | NDUFS4 | NADH:ubiquinone oxidoreductase subunit S4 [Source:HGNC Symbol;Acc:HGNC:7711] | 5871 | 0.101 | -0.1336 | No |
| 32 | DLST | dihydrolipoamide S-succinyltransferase [Source:HGNC Symbol;Acc:HGNC:2911] | 6068 | 0.085 | -0.1443 | No |
| 33 | COX10 | cytochrome c oxidase assembly factor heme A:farnesyltransferase COX10 [Source:HGNC Symbol;Acc:HGNC:2260] | 6117 | 0.082 | -0.1456 | No |
| 34 | COX5A | cytochrome c oxidase subunit 5A [Source:HGNC Symbol;Acc:HGNC:2267] | 6129 | 0.081 | -0.1445 | No |
| 35 | LRPPRC | leucine rich pentatricopeptide repeat containing [Source:HGNC Symbol;Acc:HGNC:15714] | 6165 | 0.078 | -0.1450 | No |
| 36 | UQCRB | ubiquinol-cytochrome c reductase binding protein [Source:HGNC Symbol;Acc:HGNC:12582] | 6204 | 0.076 | -0.1457 | No |
| 37 | AFG3L2 | AFG3 like matrix AAA peptidase subunit 2 [Source:HGNC Symbol;Acc:HGNC:315] | 6410 | 0.062 | -0.1576 | No |
| 38 | ATP6V1C1 | ATPase H+ transporting V1 subunit C1 [Source:HGNC Symbol;Acc:HGNC:856] | 6517 | 0.054 | -0.1632 | No |
| 39 | OAT | ornithine aminotransferase [Source:HGNC Symbol;Acc:HGNC:8091] | 6568 | 0.051 | -0.1653 | No |
| 40 | UQCRQ | ubiquinol-cytochrome c reductase complex III subunit VII [Source:HGNC Symbol;Acc:HGNC:29594] | 6627 | 0.047 | -0.1679 | No |
| 41 | HCCS | holocytochrome c synthase [Source:HGNC Symbol;Acc:HGNC:4837] | 6769 | 0.038 | -0.1762 | No |
| 42 | SDHD | succinate dehydrogenase complex subunit D [Source:HGNC Symbol;Acc:HGNC:10683] | 6809 | 0.036 | -0.1779 | No |
| 43 | ATP5F1A | ATP synthase F1 subunit alpha [Source:HGNC Symbol;Acc:HGNC:823] | 6830 | 0.034 | -0.1784 | No |
| 44 | NDUFA9 | NADH:ubiquinone oxidoreductase subunit A9 [Source:HGNC Symbol;Acc:HGNC:7693] | 6850 | 0.033 | -0.1789 | No |
| 45 | COX6C | cytochrome c oxidase subunit 6C [Source:HGNC Symbol;Acc:HGNC:2285] | 6901 | 0.029 | -0.1815 | No |
| 46 | ECHS1 | "enoyl-CoA hydratase, short chain 1 [Source:HGNC Symbol;Acc:HGNC:3151]" | 6942 | 0.026 | -0.1834 | No |
| 47 | TIMM17A | translocase of inner mitochondrial membrane 17A [Source:HGNC Symbol;Acc:HGNC:17315] | 7068 | 0.020 | -0.1911 | No |
| 48 | MRPS22 | mitochondrial ribosomal protein S22 [Source:HGNC Symbol;Acc:HGNC:14508] | 7169 | 0.014 | -0.1972 | No |
| 49 | NDUFB3 | NADH:ubiquinone oxidoreductase subunit B3 [Source:HGNC Symbol;Acc:HGNC:7698] | 7364 | 0.003 | -0.2097 | No |
| 50 | NDUFS2 | NADH:ubiquinone oxidoreductase core subunit S2 [Source:HGNC Symbol;Acc:HGNC:7708] | 7431 | -0.001 | -0.2139 | No |
| 51 | SDHA | succinate dehydrogenase complex flavoprotein subunit A [Source:HGNC Symbol;Acc:HGNC:10680] | 7439 | -0.001 | -0.2144 | No |
| 52 | ALAS1 | 5'-aminolevulinate synthase 1 [Source:HGNC Symbol;Acc:HGNC:396] | 7482 | -0.004 | -0.2170 | No |
| 53 | ACAA2 | acetyl-CoA acyltransferase 2 [Source:HGNC Symbol;Acc:HGNC:83] | 7483 | -0.004 | -0.2169 | No |
| 54 | ATP5MG | ATP synthase membrane subunit g [Source:HGNC Symbol;Acc:HGNC:14247] | 7523 | -0.006 | -0.2193 | No |
| 55 | ATP6V1E1 | ATPase H+ transporting V1 subunit E1 [Source:HGNC Symbol;Acc:HGNC:857] | 7628 | -0.010 | -0.2258 | No |
| 56 | ABCB7 | ATP binding cassette subfamily B member 7 [Source:HGNC Symbol;Acc:HGNC:48] | 7641 | -0.010 | -0.2264 | No |
| 57 | SDHB | succinate dehydrogenase complex iron sulfur subunit B [Source:HGNC Symbol;Acc:HGNC:10681] | 7682 | -0.012 | -0.2287 | No |
| 58 | ATP5MC3 | ATP synthase membrane subunit c locus 3 [Source:HGNC Symbol;Acc:HGNC:843] | 7683 | -0.012 | -0.2284 | No |
| 59 | ATP6V0B | ATPase H+ transporting V0 subunit b [Source:HGNC Symbol;Acc:HGNC:861] | 7833 | -0.021 | -0.2376 | No |
| 60 | NDUFC2 | NADH:ubiquinone oxidoreductase subunit C2 [Source:HGNC Symbol;Acc:HGNC:7706] | 7924 | -0.025 | -0.2428 | No |
| 61 | IDH1 | isocitrate dehydrogenase (NADP(+)) 1 [Source:HGNC Symbol;Acc:HGNC:5382] | 8014 | -0.030 | -0.2479 | No |
| 62 | MFN2 | mitofusin 2 [Source:HGNC Symbol;Acc:HGNC:16877] | 8082 | -0.034 | -0.2515 | No |
| 63 | FH | fumarate hydratase [Source:HGNC Symbol;Acc:HGNC:3700] | 8093 | -0.034 | -0.2513 | No |
| 64 | NDUFB6 | NADH:ubiquinone oxidoreductase subunit B6 [Source:HGNC Symbol;Acc:HGNC:7701] | 8165 | -0.038 | -0.2550 | No |
| 65 | ATP5ME | ATP synthase membrane subunit e [Source:HGNC Symbol;Acc:HGNC:846] | 8289 | -0.043 | -0.2620 | No |
| 66 | MRPL35 | mitochondrial ribosomal protein L35 [Source:HGNC Symbol;Acc:HGNC:14489] | 8295 | -0.043 | -0.2614 | No |
| 67 | DLAT | dihydrolipoamide S-acetyltransferase [Source:HGNC Symbol;Acc:HGNC:2896] | 8308 | -0.044 | -0.2612 | No |
| 68 | OGDH | oxoglutarate dehydrogenase [Source:HGNC Symbol;Acc:HGNC:8124] | 8428 | -0.051 | -0.2677 | No |
| 69 | PMPCA | "peptidase, mitochondrial processing subunit alpha [Source:HGNC Symbol;Acc:HGNC:18667]" | 8480 | -0.054 | -0.2698 | No |
| 70 | UQCRC1 | ubiquinol-cytochrome c reductase core protein 1 [Source:HGNC Symbol;Acc:HGNC:12585] | 8551 | -0.058 | -0.2730 | No |
| 71 | RHOT2 | ras homolog family member T2 [Source:HGNC Symbol;Acc:HGNC:21169] | 8597 | -0.060 | -0.2745 | No |
| 72 | COX5B | cytochrome c oxidase subunit 5B [Source:HGNC Symbol;Acc:HGNC:2269] | 8665 | -0.064 | -0.2774 | No |
| 73 | POR | cytochrome p450 oxidoreductase [Source:HGNC Symbol;Acc:HGNC:9208] | 8699 | -0.066 | -0.2780 | No |
| 74 | VDAC3 | voltage dependent anion channel 3 [Source:HGNC Symbol;Acc:HGNC:12674] | 8705 | -0.067 | -0.2768 | No |
| 75 | ISCA1 | iron-sulfur cluster assembly 1 [Source:HGNC Symbol;Acc:HGNC:28660] | 8713 | -0.068 | -0.2757 | No |
| 76 | IDH3B | isocitrate dehydrogenase (NAD(+)) 3 non-catalytic subunit beta [Source:HGNC Symbol;Acc:HGNC:5385] | 8815 | -0.073 | -0.2806 | No |
| 77 | SURF1 | SURF1 cytochrome c oxidase assembly factor [Source:HGNC Symbol;Acc:HGNC:11474] | 8825 | -0.074 | -0.2795 | No |
| 78 | CYC1 | cytochrome c1 [Source:HGNC Symbol;Acc:HGNC:2579] | 8964 | -0.080 | -0.2866 | No |
| 79 | IMMT | inner membrane mitochondrial protein [Source:HGNC Symbol;Acc:HGNC:6047] | 9073 | -0.086 | -0.2916 | No |
| 80 | NDUFA3 | NADH:ubiquinone oxidoreductase subunit A3 [Source:HGNC Symbol;Acc:HGNC:7686] | 9157 | -0.091 | -0.2949 | No |
| 81 | ACADVL | acyl-CoA dehydrogenase very long chain [Source:HGNC Symbol;Acc:HGNC:92] | 9163 | -0.091 | -0.2932 | No |
| 82 | MRPL15 | mitochondrial ribosomal protein L15 [Source:HGNC Symbol;Acc:HGNC:14054] | 9166 | -0.091 | -0.2913 | No |
| 83 | TIMM9 | translocase of inner mitochondrial membrane 9 [Source:HGNC Symbol;Acc:HGNC:11819] | 9195 | -0.093 | -0.2910 | No |
| 84 | SDHC | succinate dehydrogenase complex subunit C [Source:HGNC Symbol;Acc:HGNC:10682] | 9365 | -0.102 | -0.2996 | No |
| 85 | COX7C | cytochrome c oxidase subunit 7C [Source:HGNC Symbol;Acc:HGNC:2292] | 9375 | -0.103 | -0.2978 | No |
| 86 | ISCU | iron-sulfur cluster assembly enzyme [Source:HGNC Symbol;Acc:HGNC:29882] | 9379 | -0.103 | -0.2957 | No |
| 87 | MPC1 | mitochondrial pyruvate carrier 1 [Source:HGNC Symbol;Acc:HGNC:21606] | 9387 | -0.104 | -0.2938 | No |
| 88 | MRPS11 | mitochondrial ribosomal protein S11 [Source:HGNC Symbol;Acc:HGNC:14050] | 9388 | -0.104 | -0.2914 | No |
| 89 | NDUFA5 | NADH:ubiquinone oxidoreductase subunit A5 [Source:HGNC Symbol;Acc:HGNC:7688] | 9559 | -0.115 | -0.2998 | Yes |
| 90 | NDUFS6 | NADH:ubiquinone oxidoreductase subunit S6 [Source:HGNC Symbol;Acc:HGNC:7713] | 9567 | -0.116 | -0.2976 | Yes |
| 91 | AIFM1 | apoptosis inducing factor mitochondria associated 1 [Source:HGNC Symbol;Acc:HGNC:8768] | 9570 | -0.116 | -0.2951 | Yes |
| 92 | COX6B1 | cytochrome c oxidase subunit 6B1 [Source:HGNC Symbol;Acc:HGNC:2280] | 9587 | -0.117 | -0.2935 | Yes |
| 93 | NDUFS7 | NADH:ubiquinone oxidoreductase core subunit S7 [Source:HGNC Symbol;Acc:HGNC:7714] | 9598 | -0.117 | -0.2915 | Yes |
| 94 | ATP6V0C | ATPase H+ transporting V0 subunit c [Source:HGNC Symbol;Acc:HGNC:855] | 9611 | -0.119 | -0.2896 | Yes |
| 95 | SUPV3L1 | Suv3 like RNA helicase [Source:HGNC Symbol;Acc:HGNC:11471] | 9675 | -0.123 | -0.2909 | Yes |
| 96 | COX7A2 | cytochrome c oxidase subunit 7A2 [Source:HGNC Symbol;Acc:HGNC:2288] | 9694 | -0.124 | -0.2892 | Yes |
| 97 | UQCRFS1 | "ubiquinol-cytochrome c reductase, Rieske iron-sulfur polypeptide 1 [Source:HGNC Symbol;Acc:HGNC:12587]" | 9705 | -0.125 | -0.2870 | Yes |
| 98 | ACO2 | aconitase 2 [Source:HGNC Symbol;Acc:HGNC:118] | 9744 | -0.127 | -0.2866 | Yes |
| 99 | PHB2 | prohibitin 2 [Source:HGNC Symbol;Acc:HGNC:30306] | 9754 | -0.128 | -0.2843 | Yes |
| 100 | TOMM22 | translocase of outer mitochondrial membrane 22 [Source:HGNC Symbol;Acc:HGNC:18002] | 9759 | -0.129 | -0.2816 | Yes |
| 101 | NDUFB8 | NADH:ubiquinone oxidoreductase subunit B8 [Source:HGNC Symbol;Acc:HGNC:7703] | 9779 | -0.130 | -0.2799 | Yes |
| 102 | ATP5PD | ATP synthase peripheral stalk subunit d [Source:HGNC Symbol;Acc:HGNC:845] | 9887 | -0.136 | -0.2838 | Yes |
| 103 | NDUFB2 | NADH:ubiquinone oxidoreductase subunit B2 [Source:HGNC Symbol;Acc:HGNC:7697] | 9928 | -0.138 | -0.2832 | Yes |
| 104 | ACAA1 | acetyl-CoA acyltransferase 1 [Source:HGNC Symbol;Acc:HGNC:82] | 9995 | -0.142 | -0.2843 | Yes |
| 105 | VDAC1 | voltage dependent anion channel 1 [Source:HGNC Symbol;Acc:HGNC:12669] | 10163 | -0.151 | -0.2916 | Yes |
| 106 | MTX2 | metaxin 2 [Source:HGNC Symbol;Acc:HGNC:7506] | 10183 | -0.153 | -0.2894 | Yes |
| 107 | MRPS15 | mitochondrial ribosomal protein S15 [Source:HGNC Symbol;Acc:HGNC:14504] | 10193 | -0.153 | -0.2865 | Yes |
| 108 | COX7B | cytochrome c oxidase subunit 7B [Source:HGNC Symbol;Acc:HGNC:2291] | 10215 | -0.155 | -0.2844 | Yes |
| 109 | HADHA | hydroxyacyl-CoA dehydrogenase trifunctional multienzyme complex subunit alpha [Source:HGNC Symbol;Acc:HGNC:4801] | 10266 | -0.157 | -0.2840 | Yes |
| 110 | ATP6V1G1 | ATPase H+ transporting V1 subunit G1 [Source:HGNC Symbol;Acc:HGNC:864] | 10284 | -0.158 | -0.2815 | Yes |
| 111 | GRPEL1 | "GrpE like 1, mitochondrial [Source:HGNC Symbol;Acc:HGNC:19696]" | 10295 | -0.159 | -0.2786 | Yes |
| 112 | ATP5PB | ATP synthase peripheral stalk-membrane subunit b [Source:HGNC Symbol;Acc:HGNC:840] | 10312 | -0.160 | -0.2760 | Yes |
| 113 | ATP6V0E1 | ATPase H+ transporting V0 subunit e1 [Source:HGNC Symbol;Acc:HGNC:863] | 10404 | -0.166 | -0.2781 | Yes |
| 114 | IDH3A | isocitrate dehydrogenase (NAD(+)) 3 catalytic subunit alpha [Source:HGNC Symbol;Acc:HGNC:5384] | 10505 | -0.172 | -0.2807 | Yes |
| 115 | NDUFV2 | NADH:ubiquinone oxidoreductase core subunit V2 [Source:HGNC Symbol;Acc:HGNC:7717] | 10527 | -0.173 | -0.2781 | Yes |
| 116 | NDUFS3 | NADH:ubiquinone oxidoreductase core subunit S3 [Source:HGNC Symbol;Acc:HGNC:7710] | 10647 | -0.180 | -0.2817 | Yes |
| 117 | POLR2F | "RNA polymerase II, I and III subunit F [Source:HGNC Symbol;Acc:HGNC:9193]" | 10655 | -0.180 | -0.2781 | Yes |
| 118 | MRPS30 | mitochondrial ribosomal protein S30 [Source:HGNC Symbol;Acc:HGNC:8769] | 10769 | -0.187 | -0.2812 | Yes |
| 119 | ATP5MF | ATP synthase membrane subunit f [Source:HGNC Symbol;Acc:HGNC:848] | 10777 | -0.187 | -0.2774 | Yes |
| 120 | TIMM50 | translocase of inner mitochondrial membrane 50 [Source:HGNC Symbol;Acc:HGNC:23656] | 10780 | -0.187 | -0.2733 | Yes |
| 121 | COX6A1 | cytochrome c oxidase subunit 6A1 [Source:HGNC Symbol;Acc:HGNC:2277] | 10799 | -0.188 | -0.2702 | Yes |
| 122 | ACAT1 | acetyl-CoA acetyltransferase 1 [Source:HGNC Symbol;Acc:HGNC:93] | 10829 | -0.190 | -0.2677 | Yes |
| 123 | TIMM10 | translocase of inner mitochondrial membrane 10 [Source:HGNC Symbol;Acc:HGNC:11814] | 10911 | -0.195 | -0.2686 | Yes |
| 124 | PDP1 | pyruvate dehyrogenase phosphatase catalytic subunit 1 [Source:HGNC Symbol;Acc:HGNC:9279] | 10941 | -0.197 | -0.2660 | Yes |
| 125 | FDX1 | ferredoxin 1 [Source:HGNC Symbol;Acc:HGNC:3638] | 10974 | -0.199 | -0.2635 | Yes |
| 126 | MGST3 | microsomal glutathione S-transferase 3 [Source:HGNC Symbol;Acc:HGNC:7064] | 10989 | -0.200 | -0.2599 | Yes |
| 127 | COX15 | cytochrome c oxidase assembly homolog COX15 [Source:HGNC Symbol;Acc:HGNC:2263] | 11052 | -0.203 | -0.2593 | Yes |
| 128 | NDUFAB1 | NADH:ubiquinone oxidoreductase subunit AB1 [Source:HGNC Symbol;Acc:HGNC:7694] | 11124 | -0.207 | -0.2592 | Yes |
| 129 | ATP5F1B | ATP synthase F1 subunit beta [Source:HGNC Symbol;Acc:HGNC:830] | 11144 | -0.209 | -0.2557 | Yes |
| 130 | NDUFA2 | NADH:ubiquinone oxidoreductase subunit A2 [Source:HGNC Symbol;Acc:HGNC:7685] | 11215 | -0.212 | -0.2554 | Yes |
| 131 | ATP5F1E | ATP synthase F1 subunit epsilon [Source:HGNC Symbol;Acc:HGNC:838] | 11271 | -0.215 | -0.2541 | Yes |
| 132 | MDH1 | malate dehydrogenase 1 [Source:HGNC Symbol;Acc:HGNC:6970] | 11276 | -0.216 | -0.2495 | Yes |
| 133 | IDH3G | isocitrate dehydrogenase (NAD(+)) 3 non-catalytic subunit gamma [Source:HGNC Symbol;Acc:HGNC:5386] | 11411 | -0.224 | -0.2530 | Yes |
| 134 | NDUFS8 | NADH:ubiquinone oxidoreductase core subunit S8 [Source:HGNC Symbol;Acc:HGNC:7715] | 11451 | -0.227 | -0.2504 | Yes |
| 135 | SLC25A20 | solute carrier family 25 member 20 [Source:HGNC Symbol;Acc:HGNC:1421] | 11456 | -0.227 | -0.2456 | Yes |
| 136 | SLC25A12 | solute carrier family 25 member 12 [Source:HGNC Symbol;Acc:HGNC:10982] | 11498 | -0.229 | -0.2430 | Yes |
| 137 | GPX4 | glutathione peroxidase 4 [Source:HGNC Symbol;Acc:HGNC:4556] | 11537 | -0.231 | -0.2402 | Yes |
| 138 | TIMM13 | translocase of inner mitochondrial membrane 13 [Source:HGNC Symbol;Acc:HGNC:11816] | 11583 | -0.234 | -0.2378 | Yes |
| 139 | CPT1A | carnitine palmitoyltransferase 1A [Source:HGNC Symbol;Acc:HGNC:2328] | 11637 | -0.237 | -0.2359 | Yes |
| 140 | ALDH6A1 | aldehyde dehydrogenase 6 family member A1 [Source:HGNC Symbol;Acc:HGNC:7179] | 11806 | -0.248 | -0.2411 | Yes |
| 141 | UQCRH | ubiquinol-cytochrome c reductase hinge protein [Source:HGNC Symbol;Acc:HGNC:12590] | 11886 | -0.253 | -0.2405 | Yes |
| 142 | UQCRC2 | ubiquinol-cytochrome c reductase core protein 2 [Source:HGNC Symbol;Acc:HGNC:12586] | 11893 | -0.253 | -0.2351 | Yes |
| 143 | ACADSB | acyl-CoA dehydrogenase short/branched chain [Source:HGNC Symbol;Acc:HGNC:91] | 11899 | -0.254 | -0.2297 | Yes |
| 144 | MTRR | 5-methyltetrahydrofolate-homocysteine methyltransferase reductase [Source:HGNC Symbol;Acc:HGNC:7473] | 11909 | -0.254 | -0.2246 | Yes |
| 145 | CS | citrate synthase [Source:HGNC Symbol;Acc:HGNC:2422] | 11935 | -0.256 | -0.2204 | Yes |
| 146 | BDH2 | 3-hydroxybutyrate dehydrogenase 2 [Source:HGNC Symbol;Acc:HGNC:32389] | 11936 | -0.256 | -0.2146 | Yes |
| 147 | SLC25A5 | solute carrier family 25 member 5 [Source:HGNC Symbol;Acc:HGNC:10991] | 12003 | -0.261 | -0.2129 | Yes |
| 148 | UQCR11 | "ubiquinol-cytochrome c reductase, complex III subunit XI [Source:HGNC Symbol;Acc:HGNC:30862]" | 12014 | -0.262 | -0.2076 | Yes |
| 149 | ATP6AP1 | ATPase H+ transporting accessory protein 1 [Source:HGNC Symbol;Acc:HGNC:868] | 12086 | -0.266 | -0.2062 | Yes |
| 150 | NDUFV1 | NADH:ubiquinone oxidoreductase core subunit V1 [Source:HGNC Symbol;Acc:HGNC:7716] | 12110 | -0.268 | -0.2016 | Yes |
| 151 | ECH1 | enoyl-CoA hydratase 1 [Source:HGNC Symbol;Acc:HGNC:3149] | 12129 | -0.269 | -0.1967 | Yes |
| 152 | NDUFA8 | NADH:ubiquinone oxidoreductase subunit A8 [Source:HGNC Symbol;Acc:HGNC:7692] | 12169 | -0.272 | -0.1930 | Yes |
| 153 | NDUFA6 | NADH:ubiquinone oxidoreductase subunit A6 [Source:HGNC Symbol;Acc:HGNC:7690] | 12180 | -0.272 | -0.1875 | Yes |
| 154 | RHOT1 | ras homolog family member T1 [Source:HGNC Symbol;Acc:HGNC:21168] | 12192 | -0.273 | -0.1820 | Yes |
| 155 | NDUFC1 | NADH:ubiquinone oxidoreductase subunit C1 [Source:HGNC Symbol;Acc:HGNC:7705] | 12265 | -0.278 | -0.1804 | Yes |
| 156 | ATP5MC2 | ATP synthase membrane subunit c locus 2 [Source:HGNC Symbol;Acc:HGNC:842] | 12291 | -0.280 | -0.1757 | Yes |
| 157 | ATP5F1D | ATP synthase F1 subunit delta [Source:HGNC Symbol;Acc:HGNC:837] | 12311 | -0.281 | -0.1705 | Yes |
| 158 | PRDX3 | peroxiredoxin 3 [Source:HGNC Symbol;Acc:HGNC:9354] | 12317 | -0.282 | -0.1645 | Yes |
| 159 | MDH2 | malate dehydrogenase 2 [Source:HGNC Symbol;Acc:HGNC:6971] | 12367 | -0.286 | -0.1611 | Yes |
| 160 | NNT | nicotinamide nucleotide transhydrogenase [Source:HGNC Symbol;Acc:HGNC:7863] | 12374 | -0.286 | -0.1550 | Yes |
| 161 | SUCLG1 | succinate-CoA ligase GDP/ADP-forming subunit alpha [Source:HGNC Symbol;Acc:HGNC:11449] | 12398 | -0.288 | -0.1500 | Yes |
| 162 | COX4I1 | cytochrome c oxidase subunit 4I1 [Source:HGNC Symbol;Acc:HGNC:2265] | 12431 | -0.291 | -0.1455 | Yes |
| 163 | TIMM8B | translocase of inner mitochondrial membrane 8 homolog B [Source:HGNC Symbol;Acc:HGNC:11818] | 12525 | -0.298 | -0.1447 | Yes |
| 164 | ATP5F1C | ATP synthase F1 subunit gamma [Source:HGNC Symbol;Acc:HGNC:833] | 12527 | -0.298 | -0.1380 | Yes |
| 165 | HTRA2 | HtrA serine peptidase 2 [Source:HGNC Symbol;Acc:HGNC:14348] | 12571 | -0.302 | -0.1340 | Yes |
| 166 | MRPS12 | mitochondrial ribosomal protein S12 [Source:HGNC Symbol;Acc:HGNC:10380] | 12666 | -0.309 | -0.1331 | Yes |
| 167 | GOT2 | glutamic-oxaloacetic transaminase 2 [Source:HGNC Symbol;Acc:HGNC:4433] | 12903 | -0.328 | -0.1409 | Yes |
| 168 | SLC25A3 | solute carrier family 25 member 3 [Source:HGNC Symbol;Acc:HGNC:10989] | 13002 | -0.338 | -0.1395 | Yes |
| 169 | ATP5MC1 | ATP synthase membrane subunit c locus 1 [Source:HGNC Symbol;Acc:HGNC:841] | 13021 | -0.340 | -0.1330 | Yes |
| 170 | NDUFB5 | NADH:ubiquinone oxidoreductase subunit B5 [Source:HGNC Symbol;Acc:HGNC:7700] | 13085 | -0.346 | -0.1292 | Yes |
| 171 | SLC25A4 | solute carrier family 25 member 4 [Source:HGNC Symbol;Acc:HGNC:10990] | 13135 | -0.350 | -0.1245 | Yes |
| 172 | MRPL11 | mitochondrial ribosomal protein L11 [Source:HGNC Symbol;Acc:HGNC:14042] | 13148 | -0.352 | -0.1173 | Yes |
| 173 | LDHA | lactate dehydrogenase A [Source:HGNC Symbol;Acc:HGNC:6535] | 13197 | -0.356 | -0.1123 | Yes |
| 174 | ATP1B1 | ATPase Na+/K+ transporting subunit beta 1 [Source:HGNC Symbol;Acc:HGNC:804] | 13275 | -0.364 | -0.1091 | Yes |
| 175 | COX11 | cytochrome c oxidase copper chaperone COX11 [Source:HGNC Symbol;Acc:HGNC:2261] | 13373 | -0.377 | -0.1068 | Yes |
| 176 | ACADM | acyl-CoA dehydrogenase medium chain [Source:HGNC Symbol;Acc:HGNC:89] | 13596 | -0.404 | -0.1120 | Yes |
| 177 | ETFDH | electron transfer flavoprotein dehydrogenase [Source:HGNC Symbol;Acc:HGNC:3483] | 13794 | -0.418 | -0.1153 | Yes |
| 178 | NDUFB7 | NADH:ubiquinone oxidoreductase subunit B7 [Source:HGNC Symbol;Acc:HGNC:7702] | 13798 | -0.418 | -0.1060 | Yes |
| 179 | IDH2 | isocitrate dehydrogenase (NADP(+)) 2 [Source:HGNC Symbol;Acc:HGNC:5383] | 13807 | -0.419 | -0.0970 | Yes |
| 180 | SLC25A11 | solute carrier family 25 member 11 [Source:HGNC Symbol;Acc:HGNC:10981] | 14005 | -0.433 | -0.0999 | Yes |
| 181 | COX7A2L | cytochrome c oxidase subunit 7A2 like [Source:HGNC Symbol;Acc:HGNC:2289] | 14051 | -0.439 | -0.0929 | Yes |
| 182 | COX8A | cytochrome c oxidase subunit 8A [Source:HGNC Symbol;Acc:HGNC:2294] | 14073 | -0.442 | -0.0842 | Yes |
| 183 | GPI | glucose-6-phosphate isomerase [Source:HGNC Symbol;Acc:HGNC:4458] | 14095 | -0.448 | -0.0754 | Yes |
| 184 | PDHA1 | pyruvate dehydrogenase E1 subunit alpha 1 [Source:HGNC Symbol;Acc:HGNC:8806] | 14152 | -0.458 | -0.0687 | Yes |
| 185 | FXN | frataxin [Source:HGNC Symbol;Acc:HGNC:3951] | 14206 | -0.466 | -0.0615 | Yes |
| 186 | NDUFA7 | NADH:ubiquinone oxidoreductase subunit A7 [Source:HGNC Symbol;Acc:HGNC:7691] | 14215 | -0.467 | -0.0515 | Yes |
| 187 | MRPL34 | mitochondrial ribosomal protein L34 [Source:HGNC Symbol;Acc:HGNC:14488] | 14255 | -0.472 | -0.0433 | Yes |
| 188 | CYB5R3 | cytochrome b5 reductase 3 [Source:HGNC Symbol;Acc:HGNC:2873] | 14268 | -0.475 | -0.0333 | Yes |
| 189 | NDUFA4 | NDUFA4 mitochondrial complex associated [Source:HGNC Symbol;Acc:HGNC:7687] | 14341 | -0.485 | -0.0270 | Yes |
| 190 | ATP6V1F | ATPase H+ transporting V1 subunit F [Source:HGNC Symbol;Acc:HGNC:16832] | 14350 | -0.486 | -0.0165 | Yes |
| 191 | UQCR10 | "ubiquinol-cytochrome c reductase, complex III subunit X [Source:HGNC Symbol;Acc:HGNC:30863]" | 14366 | -0.489 | -0.0064 | Yes |
| 192 | BAX | "BCL2 associated X, apoptosis regulator [Source:HGNC Symbol;Acc:HGNC:959]" | 14408 | -0.493 | 0.0022 | Yes |
| 193 | BCKDHA | branched chain keto acid dehydrogenase E1 subunit alpha [Source:HGNC Symbol;Acc:HGNC:986] | 14551 | -0.524 | 0.0049 | Yes |
| 194 | ECI1 | enoyl-CoA delta isomerase 1 [Source:HGNC Symbol;Acc:HGNC:2703] | 14616 | -0.536 | 0.0129 | Yes |
| 195 | ETFB | electron transfer flavoprotein subunit beta [Source:HGNC Symbol;Acc:HGNC:3482] | 14803 | -0.584 | 0.0141 | Yes |
| 196 | HSD17B10 | hydroxysteroid 17-beta dehydrogenase 10 [Source:HGNC Symbol;Acc:HGNC:4800] | 14872 | -0.607 | 0.0234 | Yes |
| 197 | RETSAT | retinol saturase [Source:HGNC Symbol;Acc:HGNC:25991] | 14885 | -0.613 | 0.0366 | Yes |
| 198 | NDUFA1 | NADH:ubiquinone oxidoreductase subunit A1 [Source:HGNC Symbol;Acc:HGNC:7683] | 14899 | -0.617 | 0.0497 | Yes |
Table: GSEA details [plain text format]

  

Fig 2: HALLMARK\_OXIDATIVE\_PHOSPHORYLATION      
 Blue-Pink O' Gram in the Space of the Analyzed GeneSet

  

Fig 3: HALLMARK\_OXIDATIVE\_PHOSPHORYLATION: Random ES distribution      
 Gene set null distribution of ES for **HALLMARK\_OXIDATIVE\_PHOSPHORYLATION**
