# Supplementary material for: In vivo CRISPR screens reveal Serpinb9 and Adam2 as regulators of immune therapy response in lung cancer
Source: Nat Commun. 2023 May 31;14:3150. doi: 10.1038/s41467-023-38841-7 (PMC10232477; doi:10.1038/s41467-023-38841-7)
Supplement: Supplementary file 13 — Source Data [file 41467_2023_38841_MOESM13_ESM.zip › Figure 4E.zip!/my_analysis.Gsea.1640146340255_Hallmark/HALLMARK_COMPLEMENT.html]

Details for gene set HALLMARK\_COMPLEMENT[GSEA]

|  || Dataset | December 21, 2021\_for GSEA run\_collapsed\_to\_symbols.GSEA\_B6\_NI.cls #CTLR\_NI\_versus\_ADAM2\_NI |
| Phenotype | GSEA\_B6\_NI.cls#CTLR\_NI\_versus\_ADAM2\_NI |
| Upregulated in class | CTLR\_NI |
| GeneSet | HALLMARK\_COMPLEMENT |
| Enrichment Score (ES) | 0.43944627 |
| Normalized Enrichment Score (NES) | 1.519624 |
| Nominal p-value | 0.0 |
| FDR q-value | 0.18757401 |
| FWER p-Value | 0.449 |
Table: GSEA Results Summary

  

Fig 1: Enrichment plot: HALLMARK\_COMPLEMENT      
 Profile of the Running ES Score & Positions of GeneSet Members on the Rank Ordered List

  

| SYMBOL | TITLE | RANK IN GENE LIST | RANK METRIC SCORE | RUNNING ES | CORE ENRICHMENT || 1 | LTF | lactotransferrin [Source:HGNC Symbol;Acc:HGNC:6720] | 42 | 2.154 | 0.0232 | Yes |
| 2 | C3 | complement C3 [Source:HGNC Symbol;Acc:HGNC:1318] | 66 | 1.929 | 0.0450 | Yes |
| 3 | PSMB9 | proteasome 20S subunit beta 9 [Source:HGNC Symbol;Acc:HGNC:9546] | 87 | 1.828 | 0.0657 | Yes |
| 4 | C2 | complement C2 [Source:HGNC Symbol;Acc:HGNC:1248] | 123 | 1.731 | 0.0843 | Yes |
| 5 | CCL5 | C-C motif chemokine ligand 5 [Source:HGNC Symbol;Acc:HGNC:10632] | 169 | 1.591 | 0.1006 | Yes |
| 6 | CFB | complement factor B [Source:HGNC Symbol;Acc:HGNC:1037] | 245 | 1.387 | 0.1124 | Yes |
| 7 | CASP1 | caspase 1 [Source:HGNC Symbol;Acc:HGNC:1499] | 248 | 1.383 | 0.1290 | Yes |
| 8 | LCK | "LCK proto-oncogene, Src family tyrosine kinase [Source:HGNC Symbol;Acc:HGNC:6524]" | 286 | 1.331 | 0.1426 | Yes |
| 9 | ACTN2 | actinin alpha 2 [Source:HGNC Symbol;Acc:HGNC:164] | 288 | 1.324 | 0.1585 | Yes |
| 10 | IRF1 | interferon regulatory factor 1 [Source:HGNC Symbol;Acc:HGNC:6116] | 295 | 1.310 | 0.1739 | Yes |
| 11 | DPP4 | dipeptidyl peptidase 4 [Source:HGNC Symbol;Acc:HGNC:3009] | 349 | 1.234 | 0.1853 | Yes |
| 12 | CASP4 | caspase 4 [Source:HGNC Symbol;Acc:HGNC:1505] | 358 | 1.219 | 0.1995 | Yes |
| 13 | IRF7 | interferon regulatory factor 7 [Source:HGNC Symbol;Acc:HGNC:6122] | 359 | 1.219 | 0.2142 | Yes |
| 14 | IL6 | interleukin 6 [Source:HGNC Symbol;Acc:HGNC:6018] | 378 | 1.200 | 0.2275 | Yes |
| 15 | CTSC | cathepsin C [Source:HGNC Symbol;Acc:HGNC:2528] | 566 | 1.018 | 0.2276 | Yes |
| 16 | F5 | coagulation factor V [Source:HGNC Symbol;Acc:HGNC:3542] | 592 | 1.000 | 0.2381 | Yes |
| 17 | TMPRSS6 | transmembrane serine protease 6 [Source:HGNC Symbol;Acc:HGNC:16517] | 668 | 0.953 | 0.2447 | Yes |
| 18 | LCP2 | lymphocyte cytosolic protein 2 [Source:HGNC Symbol;Acc:HGNC:6529] | 727 | 0.918 | 0.2520 | Yes |
| 19 | RASGRP1 | RAS guanyl releasing protein 1 [Source:HGNC Symbol;Acc:HGNC:9878] | 776 | 0.890 | 0.2596 | Yes |
| 20 | MMP13 | matrix metallopeptidase 13 [Source:HGNC Symbol;Acc:HGNC:7159] | 856 | 0.859 | 0.2649 | Yes |
| 21 | CD40LG | CD40 ligand [Source:HGNC Symbol;Acc:HGNC:11935] | 918 | 0.839 | 0.2711 | Yes |
| 22 | MMP12 | matrix metallopeptidase 12 [Source:HGNC Symbol;Acc:HGNC:7158] | 950 | 0.836 | 0.2791 | Yes |
| 23 | GCA | grancalcin [Source:HGNC Symbol;Acc:HGNC:15990] | 984 | 0.828 | 0.2870 | Yes |
| 24 | SERPINB2 | serpin family B member 2 [Source:HGNC Symbol;Acc:HGNC:8584] | 1010 | 0.810 | 0.2951 | Yes |
| 25 | CASP7 | caspase 7 [Source:HGNC Symbol;Acc:HGNC:1508] | 1011 | 0.810 | 0.3049 | Yes |
| 26 | GNGT2 | G protein subunit gamma transducin 2 [Source:HGNC Symbol;Acc:HGNC:4412] | 1020 | 0.805 | 0.3141 | Yes |
| 27 | DGKG | diacylglycerol kinase gamma [Source:HGNC Symbol;Acc:HGNC:2853] | 1056 | 0.788 | 0.3213 | Yes |
| 28 | TFPI2 | tissue factor pathway inhibitor 2 [Source:HGNC Symbol;Acc:HGNC:11761] | 1096 | 0.771 | 0.3281 | Yes |
| 29 | GZMA | granzyme A [Source:HGNC Symbol;Acc:HGNC:4708] | 1113 | 0.766 | 0.3362 | Yes |
| 30 | CTSH | cathepsin H [Source:HGNC Symbol;Acc:HGNC:2535] | 1156 | 0.747 | 0.3425 | Yes |
| 31 | ITGAM | integrin subunit alpha M [Source:HGNC Symbol;Acc:HGNC:6149] | 1185 | 0.737 | 0.3496 | Yes |
| 32 | CTSS | cathepsin S [Source:HGNC Symbol;Acc:HGNC:2545] | 1201 | 0.731 | 0.3574 | Yes |
| 33 | SERPING1 | serpin family G member 1 [Source:HGNC Symbol;Acc:HGNC:1228] | 1209 | 0.728 | 0.3657 | Yes |
| 34 | F10 | coagulation factor X [Source:HGNC Symbol;Acc:HGNC:3528] | 1225 | 0.723 | 0.3735 | Yes |
| 35 | DOCK9 | dedicator of cytokinesis 9 [Source:HGNC Symbol;Acc:HGNC:14132] | 1410 | 0.657 | 0.3695 | Yes |
| 36 | S100A9 | S100 calcium binding protein A9 [Source:HGNC Symbol;Acc:HGNC:10499] | 1434 | 0.650 | 0.3759 | Yes |
| 37 | DGKH | diacylglycerol kinase eta [Source:HGNC Symbol;Acc:HGNC:2854] | 1436 | 0.649 | 0.3836 | Yes |
| 38 | GZMK | granzyme K [Source:HGNC Symbol;Acc:HGNC:4711] | 1455 | 0.640 | 0.3902 | Yes |
| 39 | PIM1 | "Pim-1 proto-oncogene, serine/threonine kinase [Source:HGNC Symbol;Acc:HGNC:8986]" | 1567 | 0.607 | 0.3903 | Yes |
| 40 | WAS | WASP actin nucleation promoting factor [Source:HGNC Symbol;Acc:HGNC:12731] | 1584 | 0.599 | 0.3965 | Yes |
| 41 | VCPIP1 | valosin containing protein interacting protein 1 [Source:HGNC Symbol;Acc:HGNC:30897] | 1595 | 0.595 | 0.4030 | Yes |
| 42 | FCER1G | Fc fragment of IgE receptor Ig [Source:HGNC Symbol;Acc:HGNC:3611] | 1652 | 0.578 | 0.4063 | Yes |
| 43 | TNFAIP3 | TNF alpha induced protein 3 [Source:HGNC Symbol;Acc:HGNC:11896] | 1713 | 0.564 | 0.4093 | Yes |
| 44 | GP9 | glycoprotein IX platelet [Source:HGNC Symbol;Acc:HGNC:4444] | 1829 | 0.537 | 0.4083 | Yes |
| 45 | CR2 | complement C3d receptor 2 [Source:HGNC Symbol;Acc:HGNC:2336] | 1996 | 0.505 | 0.4037 | Yes |
| 46 | PLEK | pleckstrin [Source:HGNC Symbol;Acc:HGNC:9070] | 2033 | 0.502 | 0.4074 | Yes |
| 47 | LYN | "LYN proto-oncogene, Src family tyrosine kinase [Source:HGNC Symbol;Acc:HGNC:6735]" | 2037 | 0.501 | 0.4132 | Yes |
| 48 | C1R | complement C1r [Source:HGNC Symbol;Acc:HGNC:1246] | 2046 | 0.499 | 0.4187 | Yes |
| 49 | GNG2 | G protein subunit gamma 2 [Source:HGNC Symbol;Acc:HGNC:4404] | 2057 | 0.498 | 0.4241 | Yes |
| 50 | PCSK9 | proprotein convertase subtilisin/kexin type 9 [Source:HGNC Symbol;Acc:HGNC:20001] | 2166 | 0.486 | 0.4230 | Yes |
| 51 | FYN | "FYN proto-oncogene, Src family tyrosine kinase [Source:HGNC Symbol;Acc:HGNC:4037]" | 2288 | 0.469 | 0.4208 | Yes |
| 52 | APOBEC3F | apolipoprotein B mRNA editing enzyme catalytic subunit 3F [Source:HGNC Symbol;Acc:HGNC:17356] | 2289 | 0.469 | 0.4265 | Yes |
| 53 | PIK3R5 | phosphoinositide-3-kinase regulatory subunit 5 [Source:HGNC Symbol;Acc:HGNC:30035] | 2331 | 0.464 | 0.4294 | Yes |
| 54 | APOC1 | apolipoprotein C1 [Source:HGNC Symbol;Acc:HGNC:607] | 2413 | 0.449 | 0.4296 | Yes |
| 55 | PIK3CG | "phosphatidylinositol-4,5-bisphosphate 3-kinase catalytic subunit gamma [Source:HGNC Symbol;Acc:HGNC:8978]" | 2427 | 0.447 | 0.4341 | Yes |
| 56 | GMFB | glia maturation factor beta [Source:HGNC Symbol;Acc:HGNC:4373] | 2429 | 0.447 | 0.4394 | Yes |
| 57 | C9 | complement C9 [Source:HGNC Symbol;Acc:HGNC:1358] | 2652 | 0.426 | 0.4302 | No |
| 58 | PRKCD | protein kinase C delta [Source:HGNC Symbol;Acc:HGNC:9399] | 2749 | 0.426 | 0.4292 | No |
| 59 | DUSP5 | dual specificity phosphatase 5 [Source:HGNC Symbol;Acc:HGNC:3071] | 2753 | 0.425 | 0.4341 | No |
| 60 | IRF2 | interferon regulatory factor 2 [Source:HGNC Symbol;Acc:HGNC:6117] | 3080 | 0.391 | 0.4177 | No |
| 61 | C1QC | complement C1q C chain [Source:HGNC Symbol;Acc:HGNC:1245] | 3116 | 0.386 | 0.4201 | No |
| 62 | C1QA | complement C1q A chain [Source:HGNC Symbol;Acc:HGNC:1241] | 3162 | 0.378 | 0.4218 | No |
| 63 | ADRA2B | adrenoceptor alpha 2B [Source:HGNC Symbol;Acc:HGNC:282] | 3245 | 0.366 | 0.4209 | No |
| 64 | CXCL1 | C-X-C motif chemokine ligand 1 [Source:HGNC Symbol;Acc:HGNC:4602] | 3324 | 0.354 | 0.4201 | No |
| 65 | CTSB | cathepsin B [Source:HGNC Symbol;Acc:HGNC:2527] | 3373 | 0.346 | 0.4212 | No |
| 66 | PLA2G4A | phospholipase A2 group IVA [Source:HGNC Symbol;Acc:HGNC:9035] | 3396 | 0.342 | 0.4239 | No |
| 67 | PLA2G7 | phospholipase A2 group VII [Source:HGNC Symbol;Acc:HGNC:9040] | 3406 | 0.340 | 0.4274 | No |
| 68 | C1S | complement C1s [Source:HGNC Symbol;Acc:HGNC:1247] | 3521 | 0.324 | 0.4239 | No |
| 69 | JAK2 | Janus kinase 2 [Source:HGNC Symbol;Acc:HGNC:6192] | 3644 | 0.308 | 0.4198 | No |
| 70 | DOCK4 | dedicator of cytokinesis 4 [Source:HGNC Symbol;Acc:HGNC:19192] | 3733 | 0.299 | 0.4177 | No |
| 71 | CBLB | Cbl proto-oncogene B [Source:HGNC Symbol;Acc:HGNC:1542] | 3937 | 0.274 | 0.4079 | No |
| 72 | USP16 | ubiquitin specific peptidase 16 [Source:HGNC Symbol;Acc:HGNC:12614] | 3962 | 0.270 | 0.4096 | No |
| 73 | LGMN | legumain [Source:HGNC Symbol;Acc:HGNC:9472] | 4001 | 0.265 | 0.4103 | No |
| 74 | LGALS3 | galectin 3 [Source:HGNC Symbol;Acc:HGNC:6563] | 4079 | 0.256 | 0.4084 | No |
| 75 | USP14 | ubiquitin specific peptidase 14 [Source:HGNC Symbol;Acc:HGNC:12612] | 4092 | 0.255 | 0.4107 | No |
| 76 | KCNIP2 | potassium voltage-gated channel interacting protein 2 [Source:HGNC Symbol;Acc:HGNC:15522] | 4231 | 0.239 | 0.4047 | No |
| 77 | CSRP1 | cysteine and glycine rich protein 1 [Source:HGNC Symbol;Acc:HGNC:2469] | 4263 | 0.236 | 0.4055 | No |
| 78 | KLKB1 | kallikrein B1 [Source:HGNC Symbol;Acc:HGNC:6371] | 4362 | 0.226 | 0.4019 | No |
| 79 | CEBPB | CCAAT enhancer binding protein beta [Source:HGNC Symbol;Acc:HGNC:1834] | 4438 | 0.219 | 0.3997 | No |
| 80 | OLR1 | oxidized low density lipoprotein receptor 1 [Source:HGNC Symbol;Acc:HGNC:8133] | 4464 | 0.217 | 0.4007 | No |
| 81 | RHOG | ras homolog family member G [Source:HGNC Symbol;Acc:HGNC:672] | 4535 | 0.210 | 0.3987 | No |
| 82 | CD36 | CD36 molecule [Source:HGNC Symbol;Acc:HGNC:1663] | 4685 | 0.198 | 0.3915 | No |
| 83 | TIMP1 | TIMP metallopeptidase inhibitor 1 [Source:HGNC Symbol;Acc:HGNC:11820] | 4971 | 0.171 | 0.3751 | No |
| 84 | KYNU | kynureninase [Source:HGNC Symbol;Acc:HGNC:6469] | 5014 | 0.166 | 0.3744 | No |
| 85 | PLAUR | "plasminogen activator, urokinase receptor [Source:HGNC Symbol;Acc:HGNC:9053]" | 5260 | 0.146 | 0.3604 | No |
| 86 | DYRK2 | dual specificity tyrosine phosphorylation regulated kinase 2 [Source:HGNC Symbol;Acc:HGNC:3093] | 5298 | 0.142 | 0.3597 | No |
| 87 | GRB2 | growth factor receptor bound protein 2 [Source:HGNC Symbol;Acc:HGNC:4566] | 5321 | 0.141 | 0.3600 | No |
| 88 | CASP9 | caspase 9 [Source:HGNC Symbol;Acc:HGNC:1511] | 5704 | 0.112 | 0.3366 | No |
| 89 | KIF2A | kinesin family member 2A [Source:HGNC Symbol;Acc:HGNC:6318] | 5993 | 0.092 | 0.3191 | No |
| 90 | GP1BA | glycoprotein Ib platelet subunit alpha [Source:HGNC Symbol;Acc:HGNC:4439] | 6050 | 0.087 | 0.3166 | No |
| 91 | USP15 | ubiquitin specific peptidase 15 [Source:HGNC Symbol;Acc:HGNC:12613] | 6213 | 0.076 | 0.3070 | No |
| 92 | ANG | angiogenin [Source:HGNC Symbol;Acc:HGNC:483] | 6415 | 0.061 | 0.2948 | No |
| 93 | MSRB1 | methionine sulfoxide reductase B1 [Source:HGNC Symbol;Acc:HGNC:14133] | 6429 | 0.060 | 0.2946 | No |
| 94 | HSPA1A | heat shock protein family A (Hsp70) member 1A [Source:HGNC Symbol;Acc:HGNC:5232] | 6483 | 0.056 | 0.2919 | No |
| 95 | CASP3 | caspase 3 [Source:HGNC Symbol;Acc:HGNC:1504] | 6509 | 0.054 | 0.2909 | No |
| 96 | CTSV | cathepsin V [Source:HGNC Symbol;Acc:HGNC:2538] | 6538 | 0.053 | 0.2898 | No |
| 97 | RABIF | RAB interacting factor [Source:HGNC Symbol;Acc:HGNC:9797] | 6601 | 0.048 | 0.2863 | No |
| 98 | MMP8 | matrix metallopeptidase 8 [Source:HGNC Symbol;Acc:HGNC:7175] | 6643 | 0.046 | 0.2842 | No |
| 99 | CD59 | CD59 molecule (CD59 blood group) [Source:HGNC Symbol;Acc:HGNC:1689] | 6681 | 0.044 | 0.2824 | No |
| 100 | ZEB1 | zinc finger E-box binding homeobox 1 [Source:HGNC Symbol;Acc:HGNC:11642] | 6792 | 0.037 | 0.2757 | No |
| 101 | PPP4C | protein phosphatase 4 catalytic subunit [Source:HGNC Symbol;Acc:HGNC:9319] | 6838 | 0.034 | 0.2732 | No |
| 102 | ME1 | malic enzyme 1 [Source:HGNC Symbol;Acc:HGNC:6983] | 6909 | 0.029 | 0.2690 | No |
| 103 | F2 | "coagulation factor II, thrombin [Source:HGNC Symbol;Acc:HGNC:3535]" | 6945 | 0.026 | 0.2671 | No |
| 104 | SCG3 | secretogranin III [Source:HGNC Symbol;Acc:HGNC:13707] | 6953 | 0.026 | 0.2670 | No |
| 105 | LAMP2 | lysosomal associated membrane protein 2 [Source:HGNC Symbol;Acc:HGNC:6501] | 7018 | 0.023 | 0.2631 | No |
| 106 | PLSCR1 | phospholipid scramblase 1 [Source:HGNC Symbol;Acc:HGNC:9092] | 7180 | 0.013 | 0.2529 | No |
| 107 | CD46 | CD46 molecule [Source:HGNC Symbol;Acc:HGNC:6953] | 7302 | 0.006 | 0.2451 | No |
| 108 | PSEN1 | presenilin 1 [Source:HGNC Symbol;Acc:HGNC:9508] | 7304 | 0.006 | 0.2452 | No |
| 109 | PRDM4 | PR/SET domain 4 [Source:HGNC Symbol;Acc:HGNC:9348] | 7380 | 0.002 | 0.2403 | No |
| 110 | ITIH1 | inter-alpha-trypsin inhibitor heavy chain 1 [Source:HGNC Symbol;Acc:HGNC:6166] | 7544 | -0.006 | 0.2299 | No |
| 111 | CTSO | cathepsin O [Source:HGNC Symbol;Acc:HGNC:2542] | 7546 | -0.006 | 0.2299 | No |
| 112 | SRC | "SRC proto-oncogene, non-receptor tyrosine kinase [Source:HGNC Symbol;Acc:HGNC:11283]" | 7732 | -0.015 | 0.2181 | No |
| 113 | PFN1 | profilin 1 [Source:HGNC Symbol;Acc:HGNC:8881] | 7810 | -0.019 | 0.2134 | No |
| 114 | USP8 | ubiquitin specific peptidase 8 [Source:HGNC Symbol;Acc:HGNC:12631] | 7825 | -0.020 | 0.2127 | No |
| 115 | SIRT6 | sirtuin 6 [Source:HGNC Symbol;Acc:HGNC:14934] | 7848 | -0.021 | 0.2116 | No |
| 116 | GNB4 | G protein subunit beta 4 [Source:HGNC Symbol;Acc:HGNC:20731] | 7881 | -0.023 | 0.2098 | No |
| 117 | LRP1 | LDL receptor related protein 1 [Source:HGNC Symbol;Acc:HGNC:6692] | 7894 | -0.024 | 0.2093 | No |
| 118 | SPOCK2 | "SPARC (osteonectin), cwcv and kazal like domains proteoglycan 2 [Source:HGNC Symbol;Acc:HGNC:13564]" | 7910 | -0.024 | 0.2086 | No |
| 119 | GPD2 | glycerol-3-phosphate dehydrogenase 2 [Source:HGNC Symbol;Acc:HGNC:4456] | 7999 | -0.029 | 0.2033 | No |
| 120 | LAP3 | leucine aminopeptidase 3 [Source:HGNC Symbol;Acc:HGNC:18449] | 8033 | -0.031 | 0.2015 | No |
| 121 | CD55 | CD55 molecule (Cromer blood group) [Source:HGNC Symbol;Acc:HGNC:2665] | 8098 | -0.035 | 0.1978 | No |
| 122 | GNAI3 | G protein subunit alpha i3 [Source:HGNC Symbol;Acc:HGNC:4387] | 8386 | -0.048 | 0.1798 | No |
| 123 | AKAP10 | A-kinase anchoring protein 10 [Source:HGNC Symbol;Acc:HGNC:368] | 8430 | -0.051 | 0.1777 | No |
| 124 | STX4 | syntaxin 4 [Source:HGNC Symbol;Acc:HGNC:11439] | 8510 | -0.055 | 0.1732 | No |
| 125 | PRSS3 | serine protease 3 [Source:HGNC Symbol;Acc:HGNC:9486] | 8513 | -0.055 | 0.1738 | No |
| 126 | KLK1 | kallikrein 1 [Source:HGNC Symbol;Acc:HGNC:6357] | 8538 | -0.057 | 0.1729 | No |
| 127 | ADAM9 | ADAM metallopeptidase domain 9 [Source:HGNC Symbol;Acc:HGNC:216] | 8719 | -0.068 | 0.1621 | No |
| 128 | EHD1 | EH domain containing 1 [Source:HGNC Symbol;Acc:HGNC:3242] | 8788 | -0.071 | 0.1586 | No |
| 129 | RNF4 | ring finger protein 4 [Source:HGNC Symbol;Acc:HGNC:10067] | 8923 | -0.078 | 0.1508 | No |
| 130 | DOCK10 | dedicator of cytokinesis 10 [Source:HGNC Symbol;Acc:HGNC:23479] | 9043 | -0.084 | 0.1442 | No |
| 131 | PIK3CA | "phosphatidylinositol-4,5-bisphosphate 3-kinase catalytic subunit alpha [Source:HGNC Symbol;Acc:HGNC:8975]" | 9078 | -0.087 | 0.1430 | No |
| 132 | F8 | coagulation factor VIII [Source:HGNC Symbol;Acc:HGNC:3546] | 9135 | -0.090 | 0.1405 | No |
| 133 | PREP | prolyl endopeptidase [Source:HGNC Symbol;Acc:HGNC:9358] | 9307 | -0.099 | 0.1306 | No |
| 134 | LIPA | "lipase A, lysosomal acid type [Source:HGNC Symbol;Acc:HGNC:6617]" | 9313 | -0.099 | 0.1315 | No |
| 135 | CALM3 | calmodulin 3 [Source:HGNC Symbol;Acc:HGNC:1449] | 9382 | -0.103 | 0.1284 | No |
| 136 | RAF1 | "Raf-1 proto-oncogene, serine/threonine kinase [Source:HGNC Symbol;Acc:HGNC:9829]" | 9515 | -0.112 | 0.1212 | No |
| 137 | RBSN | "rabenosyn, RAB effector [Source:HGNC Symbol;Acc:HGNC:20759]" | 9530 | -0.113 | 0.1216 | No |
| 138 | CALM1 | calmodulin 1 [Source:HGNC Symbol;Acc:HGNC:1442] | 9602 | -0.118 | 0.1185 | No |
| 139 | PPP2CB | protein phosphatase 2 catalytic subunit beta [Source:HGNC Symbol;Acc:HGNC:9300] | 9637 | -0.121 | 0.1177 | No |
| 140 | MMP14 | matrix metallopeptidase 14 [Source:HGNC Symbol;Acc:HGNC:7160] | 9684 | -0.124 | 0.1162 | No |
| 141 | LTA4H | leukotriene A4 hydrolase [Source:HGNC Symbol;Acc:HGNC:6710] | 9826 | -0.132 | 0.1087 | No |
| 142 | GNAI2 | G protein subunit alpha i2 [Source:HGNC Symbol;Acc:HGNC:4385] | 9874 | -0.135 | 0.1073 | No |
| 143 | SERPINE1 | serpin family E member 1 [Source:HGNC Symbol;Acc:HGNC:8583] | 10052 | -0.145 | 0.0976 | No |
| 144 | KCNIP3 | potassium voltage-gated channel interacting protein 3 [Source:HGNC Symbol;Acc:HGNC:15523] | 10053 | -0.145 | 0.0994 | No |
| 145 | F3 | "coagulation factor III, tissue factor [Source:HGNC Symbol;Acc:HGNC:3541]" | 10575 | -0.175 | 0.0678 | No |
| 146 | F7 | coagulation factor VII [Source:HGNC Symbol;Acc:HGNC:3544] | 10577 | -0.176 | 0.0699 | No |
| 147 | PDGFB | platelet derived growth factor subunit B [Source:HGNC Symbol;Acc:HGNC:8800] | 10685 | -0.182 | 0.0652 | No |
| 148 | PRCP | prolylcarboxypeptidase [Source:HGNC Symbol;Acc:HGNC:9344] | 10705 | -0.183 | 0.0661 | No |
| 149 | PDP1 | pyruvate dehyrogenase phosphatase catalytic subunit 1 [Source:HGNC Symbol;Acc:HGNC:9279] | 10941 | -0.197 | 0.0533 | No |
| 150 | FDX1 | ferredoxin 1 [Source:HGNC Symbol;Acc:HGNC:3638] | 10974 | -0.199 | 0.0537 | No |
| 151 | CTSD | cathepsin D [Source:HGNC Symbol;Acc:HGNC:2529] | 11141 | -0.209 | 0.0455 | No |
| 152 | DUSP6 | dual specificity phosphatase 6 [Source:HGNC Symbol;Acc:HGNC:3072] | 11560 | -0.232 | 0.0213 | No |
| 153 | CLU | clusterin [Source:HGNC Symbol;Acc:HGNC:2095] | 11910 | -0.254 | 0.0018 | No |
| 154 | BRPF3 | bromodomain and PHD finger containing 3 [Source:HGNC Symbol;Acc:HGNC:14256] | 11913 | -0.254 | 0.0047 | No |
| 155 | RCE1 | Ras converting CAAX endopeptidase 1 [Source:HGNC Symbol;Acc:HGNC:13721] | 12049 | -0.264 | -0.0008 | No |
| 156 | PCLO | piccolo presynaptic cytomatrix protein [Source:HGNC Symbol;Acc:HGNC:13406] | 12112 | -0.268 | -0.0016 | No |
| 157 | SH2B3 | SH2B adaptor protein 3 [Source:HGNC Symbol;Acc:HGNC:29605] | 12116 | -0.268 | 0.0014 | No |
| 158 | ATOX1 | antioxidant 1 copper chaperone [Source:HGNC Symbol;Acc:HGNC:798] | 12252 | -0.277 | -0.0039 | No |
| 159 | HSPA5 | heat shock protein family A (Hsp70) member 5 [Source:HGNC Symbol;Acc:HGNC:5238] | 12586 | -0.303 | -0.0218 | No |
| 160 | MAFF | MAF bZIP transcription factor F [Source:HGNC Symbol;Acc:HGNC:6780] | 12675 | -0.309 | -0.0238 | No |
| 161 | XPNPEP1 | X-prolyl aminopeptidase 1 [Source:HGNC Symbol;Acc:HGNC:12822] | 12926 | -0.331 | -0.0359 | No |
| 162 | GZMB | granzyme B [Source:HGNC Symbol;Acc:HGNC:4709] | 13371 | -0.376 | -0.0601 | No |
| 163 | ZFPM2 | "zinc finger protein, FOG family member 2 [Source:HGNC Symbol;Acc:HGNC:16700]" | 13426 | -0.382 | -0.0590 | No |
| 164 | GNB2 | G protein subunit beta 2 [Source:HGNC Symbol;Acc:HGNC:4398] | 13592 | -0.404 | -0.0648 | No |
| 165 | SERPINA1 | serpin family A member 1 [Source:HGNC Symbol;Acc:HGNC:8941] | 13828 | -0.422 | -0.0749 | No |
| 166 | FCN1 | ficolin 1 [Source:HGNC Symbol;Acc:HGNC:3623] | 13941 | -0.428 | -0.0769 | No |
| 167 | CDK5R1 | cyclin dependent kinase 5 regulatory subunit 1 [Source:HGNC Symbol;Acc:HGNC:1775] | 14006 | -0.433 | -0.0759 | No |
| 168 | FN1 | fibronectin 1 [Source:HGNC Symbol;Acc:HGNC:3778] | 14037 | -0.437 | -0.0725 | No |
| 169 | CDA | cytidine deaminase [Source:HGNC Symbol;Acc:HGNC:1712] | 14101 | -0.449 | -0.0712 | No |
| 170 | CP | ceruloplasmin [Source:HGNC Symbol;Acc:HGNC:2295] | 14171 | -0.461 | -0.0701 | No |
| 171 | SERPINC1 | serpin family C member 1 [Source:HGNC Symbol;Acc:HGNC:775] | 14208 | -0.466 | -0.0668 | No |
| 172 | S100A13 | S100 calcium binding protein A13 [Source:HGNC Symbol;Acc:HGNC:10490] | 14224 | -0.468 | -0.0621 | No |
| 173 | PRSS36 | serine protease 36 [Source:HGNC Symbol;Acc:HGNC:26906] | 14354 | -0.488 | -0.0646 | No |
| 174 | PLAT | "plasminogen activator, tissue type [Source:HGNC Symbol;Acc:HGNC:9051]" | 14379 | -0.490 | -0.0602 | No |
| 175 | CPQ | carboxypeptidase Q [Source:HGNC Symbol;Acc:HGNC:16910] | 14651 | -0.542 | -0.0712 | No |
| 176 | ANXA5 | annexin A5 [Source:HGNC Symbol;Acc:HGNC:543] | 14759 | -0.571 | -0.0712 | No |
| 177 | TIMP2 | TIMP metallopeptidase inhibitor 2 [Source:HGNC Symbol;Acc:HGNC:11821] | 14863 | -0.603 | -0.0706 | No |
| 178 | COL4A2 | collagen type IV alpha 2 chain [Source:HGNC Symbol;Acc:HGNC:2203] | 14887 | -0.614 | -0.0647 | No |
| 179 | NOTCH4 | notch receptor 4 [Source:HGNC Symbol;Acc:HGNC:7884] | 15032 | -0.678 | -0.0658 | No |
| 180 | CPM | carboxypeptidase M [Source:HGNC Symbol;Acc:HGNC:2311] | 15150 | -0.752 | -0.0643 | No |
| 181 | CFH | complement factor H [Source:HGNC Symbol;Acc:HGNC:4883] | 15171 | -0.760 | -0.0565 | No |
| 182 | MT3 | metallothionein 3 [Source:HGNC Symbol;Acc:HGNC:7408] | 15277 | -0.824 | -0.0533 | No |
| 183 | PHEX | phosphate regulating endopeptidase homolog X-linked [Source:HGNC Symbol;Acc:HGNC:8918] | 15453 | -0.961 | -0.0531 | No |
| 184 | CDH13 | cadherin 13 [Source:HGNC Symbol;Acc:HGNC:1753] | 15514 | -1.064 | -0.0441 | No |
| 185 | CA2 | carbonic anhydrase 2 [Source:HGNC Symbol;Acc:HGNC:1373] | 15522 | -1.087 | -0.0315 | No |
| 186 | MMP15 | matrix metallopeptidase 15 [Source:HGNC Symbol;Acc:HGNC:7161] | 15583 | -1.234 | -0.0205 | No |
| 187 | GATA3 | GATA binding protein 3 [Source:HGNC Symbol;Acc:HGNC:4172] | 15661 | -2.152 | 0.0005 | No |
Table: GSEA details [plain text format]

  

Fig 2: HALLMARK\_COMPLEMENT      
 Blue-Pink O' Gram in the Space of the Analyzed GeneSet

  

Fig 3: HALLMARK\_COMPLEMENT: Random ES distribution      
 Gene set null distribution of ES for **HALLMARK\_COMPLEMENT**
