# Supplementary material for: In vivo CRISPR screens reveal Serpinb9 and Adam2 as regulators of immune therapy response in lung cancer
Source: Nat Commun. 2023 May 31;14:3150. doi: 10.1038/s41467-023-38841-7 (PMC10232477; doi:10.1038/s41467-023-38841-7)
Supplement: Supplementary file 13 — Source Data [file 41467_2023_38841_MOESM13_ESM.zip › Figure 4E.zip!/my_analysis.Gsea.1640146340255_Hallmark/HALLMARK_TGF_BETA_SIGNALING.html]

Details for gene set HALLMARK\_TGF\_BETA\_SIGNALING[GSEA]

|  || Dataset | December 21, 2021\_for GSEA run\_collapsed\_to\_symbols.GSEA\_B6\_NI.cls #CTLR\_NI\_versus\_ADAM2\_NI |
| Phenotype | GSEA\_B6\_NI.cls#CTLR\_NI\_versus\_ADAM2\_NI |
| Upregulated in class | ADAM2\_NI |
| GeneSet | HALLMARK\_TGF\_BETA\_SIGNALING |
| Enrichment Score (ES) | -0.30736277 |
| Normalized Enrichment Score (NES) | -1.157852 |
| Nominal p-value | 0.2989899 |
| FDR q-value | 0.8512557 |
| FWER p-Value | 1.0 |
Table: GSEA Results Summary

  

Fig 1: Enrichment plot: HALLMARK\_TGF\_BETA\_SIGNALING      
 Profile of the Running ES Score & Positions of GeneSet Members on the Rank Ordered List

  

| SYMBOL | TITLE | RANK IN GENE LIST | RANK METRIC SCORE | RUNNING ES | CORE ENRICHMENT || 1 | LEFTY2 | left-right determination factor 2 [Source:HGNC Symbol;Acc:HGNC:3122] | 893 | 0.845 | 0.0103 | No |
| 2 | NOG | noggin [Source:HGNC Symbol;Acc:HGNC:7866] | 2476 | 0.445 | -0.0555 | No |
| 3 | THBS1 | thrombospondin 1 [Source:HGNC Symbol;Acc:HGNC:11785] | 3316 | 0.355 | -0.0808 | No |
| 4 | SLC20A1 | solute carrier family 20 member 1 [Source:HGNC Symbol;Acc:HGNC:10946] | 3479 | 0.331 | -0.0648 | No |
| 5 | IFNGR2 | interferon gamma receptor 2 [Source:HGNC Symbol;Acc:HGNC:5440] | 3530 | 0.323 | -0.0422 | No |
| 6 | APC | APC regulator of WNT signaling pathway [Source:HGNC Symbol;Acc:HGNC:583] | 3689 | 0.302 | -0.0281 | No |
| 7 | SKIL | SKI like proto-oncogene [Source:HGNC Symbol;Acc:HGNC:10897] | 4386 | 0.225 | -0.0548 | No |
| 8 | BMPR2 | bone morphogenetic protein receptor type 2 [Source:HGNC Symbol;Acc:HGNC:1078] | 4766 | 0.192 | -0.0637 | No |
| 9 | HIPK2 | homeodomain interacting protein kinase 2 [Source:HGNC Symbol;Acc:HGNC:14402] | 5280 | 0.144 | -0.0851 | No |
| 10 | BMPR1A | bone morphogenetic protein receptor type 1A [Source:HGNC Symbol;Acc:HGNC:1076] | 5304 | 0.141 | -0.0752 | No |
| 11 | UBE2D3 | ubiquitin conjugating enzyme E2 D3 [Source:HGNC Symbol;Acc:HGNC:12476] | 5615 | 0.118 | -0.0857 | No |
| 12 | KLF10 | Kruppel like factor 10 [Source:HGNC Symbol;Acc:HGNC:11810] | 5683 | 0.114 | -0.0809 | No |
| 13 | SMAD7 | SMAD family member 7 [Source:HGNC Symbol;Acc:HGNC:6773] | 5911 | 0.098 | -0.0876 | No |
| 14 | BCAR3 | "BCAR3 adaptor protein, NSP family member [Source:HGNC Symbol;Acc:HGNC:973]" | 5937 | 0.096 | -0.0816 | No |
| 15 | TGFB1 | transforming growth factor beta 1 [Source:HGNC Symbol;Acc:HGNC:11766] | 6038 | 0.088 | -0.0809 | No |
| 16 | SMURF1 | SMAD specific E3 ubiquitin protein ligase 1 [Source:HGNC Symbol;Acc:HGNC:16807] | 6453 | 0.058 | -0.1028 | No |
| 17 | PPM1A | "protein phosphatase, Mg2+/Mn2+ dependent 1A [Source:HGNC Symbol;Acc:HGNC:9275]" | 6799 | 0.036 | -0.1220 | No |
| 18 | TRIM33 | tripartite motif containing 33 [Source:HGNC Symbol;Acc:HGNC:16290] | 6915 | 0.029 | -0.1271 | No |
| 19 | XIAP | X-linked inhibitor of apoptosis [Source:HGNC Symbol;Acc:HGNC:592] | 7316 | 0.005 | -0.1522 | No |
| 20 | SMAD1 | SMAD family member 1 [Source:HGNC Symbol;Acc:HGNC:6767] | 7847 | -0.021 | -0.1845 | No |
| 21 | PPP1R15A | protein phosphatase 1 regulatory subunit 15A [Source:HGNC Symbol;Acc:HGNC:14375] | 8019 | -0.030 | -0.1931 | No |
| 22 | TJP1 | tight junction protein 1 [Source:HGNC Symbol;Acc:HGNC:11827] | 8203 | -0.040 | -0.2016 | No |
| 23 | CDK9 | cyclin dependent kinase 9 [Source:HGNC Symbol;Acc:HGNC:1780] | 8359 | -0.047 | -0.2078 | No |
| 24 | ARID4B | AT-rich interaction domain 4B [Source:HGNC Symbol;Acc:HGNC:15550] | 8360 | -0.047 | -0.2041 | No |
| 25 | ID2 | inhibitor of DNA binding 2 [Source:HGNC Symbol;Acc:HGNC:5361] | 8387 | -0.048 | -0.2019 | No |
| 26 | HDAC1 | histone deacetylase 1 [Source:HGNC Symbol;Acc:HGNC:4852] | 8540 | -0.057 | -0.2070 | No |
| 27 | TGFBR1 | transforming growth factor beta receptor 1 [Source:HGNC Symbol;Acc:HGNC:11772] | 8664 | -0.064 | -0.2098 | No |
| 28 | RHOA | ras homolog family member A [Source:HGNC Symbol;Acc:HGNC:667] | 8727 | -0.069 | -0.2083 | No |
| 29 | SMURF2 | SMAD specific E3 ubiquitin protein ligase 2 [Source:HGNC Symbol;Acc:HGNC:16809] | 9085 | -0.087 | -0.2242 | No |
| 30 | SPTBN1 | "spectrin beta, non-erythrocytic 1 [Source:HGNC Symbol;Acc:HGNC:11275]" | 9235 | -0.095 | -0.2262 | No |
| 31 | CTNNB1 | catenin beta 1 [Source:HGNC Symbol;Acc:HGNC:2514] | 9410 | -0.105 | -0.2289 | No |
| 32 | SERPINE1 | serpin family E member 1 [Source:HGNC Symbol;Acc:HGNC:8583] | 10052 | -0.145 | -0.2584 | No |
| 33 | ID1 | "inhibitor of DNA binding 1, HLH protein [Source:HGNC Symbol;Acc:HGNC:5360]" | 10637 | -0.179 | -0.2815 | No |
| 34 | FKBP1A | FKBP prolyl isomerase 1A [Source:HGNC Symbol;Acc:HGNC:3711] | 10949 | -0.198 | -0.2856 | No |
| 35 | FURIN | "furin, paired basic amino acid cleaving enzyme [Source:HGNC Symbol;Acc:HGNC:8568]" | 11210 | -0.212 | -0.2853 | No |
| 36 | MAP3K7 | mitogen-activated protein kinase kinase kinase 7 [Source:HGNC Symbol;Acc:HGNC:6859] | 11555 | -0.232 | -0.2888 | Yes |
| 37 | CDKN1C | cyclin dependent kinase inhibitor 1C [Source:HGNC Symbol;Acc:HGNC:1786] | 11672 | -0.239 | -0.2772 | Yes |
| 38 | ACVR1 | activin A receptor type 1 [Source:HGNC Symbol;Acc:HGNC:171] | 11734 | -0.243 | -0.2617 | Yes |
| 39 | LTBP2 | latent transforming growth factor beta binding protein 2 [Source:HGNC Symbol;Acc:HGNC:6715] | 11803 | -0.248 | -0.2462 | Yes |
| 40 | JUNB | "JunB proto-oncogene, AP-1 transcription factor subunit [Source:HGNC Symbol;Acc:HGNC:6205]" | 11848 | -0.250 | -0.2290 | Yes |
| 41 | ID3 | "inhibitor of DNA binding 3, HLH protein [Source:HGNC Symbol;Acc:HGNC:5362]" | 11914 | -0.254 | -0.2129 | Yes |
| 42 | TGIF1 | TGFB induced factor homeobox 1 [Source:HGNC Symbol;Acc:HGNC:11776] | 12041 | -0.264 | -0.1999 | Yes |
| 43 | PPP1CA | protein phosphatase 1 catalytic subunit alpha [Source:HGNC Symbol;Acc:HGNC:9281] | 12176 | -0.272 | -0.1868 | Yes |
| 44 | CDH1 | cadherin 1 [Source:HGNC Symbol;Acc:HGNC:1748] | 12181 | -0.272 | -0.1653 | Yes |
| 45 | SKI | SKI proto-oncogene [Source:HGNC Symbol;Acc:HGNC:10896] | 12233 | -0.276 | -0.1465 | Yes |
| 46 | BMP2 | bone morphogenetic protein 2 [Source:HGNC Symbol;Acc:HGNC:1069] | 12618 | -0.305 | -0.1468 | Yes |
| 47 | RAB31 | "RAB31, member RAS oncogene family [Source:HGNC Symbol;Acc:HGNC:9771]" | 12683 | -0.310 | -0.1261 | Yes |
| 48 | WWTR1 | WW domain containing transcription regulator 1 [Source:HGNC Symbol;Acc:HGNC:24042] | 12884 | -0.327 | -0.1128 | Yes |
| 49 | NCOR2 | nuclear receptor corepressor 2 [Source:HGNC Symbol;Acc:HGNC:7673] | 13301 | -0.367 | -0.1101 | Yes |
| 50 | FNTA | "farnesyltransferase, CAAX box, alpha [Source:HGNC Symbol;Acc:HGNC:3782]" | 13461 | -0.387 | -0.0894 | Yes |
| 51 | PMEPA1 | "prostate transmembrane protein, androgen induced 1 [Source:HGNC Symbol;Acc:HGNC:14107]" | 14329 | -0.484 | -0.1063 | Yes |
| 52 | SMAD3 | SMAD family member 3 [Source:HGNC Symbol;Acc:HGNC:6769] | 15161 | -0.755 | -0.0992 | Yes |
| 53 | ENG | endoglin [Source:HGNC Symbol;Acc:HGNC:3349] | 15264 | -0.815 | -0.0406 | Yes |
| 54 | SMAD6 | SMAD family member 6 [Source:HGNC Symbol;Acc:HGNC:6772] | 15286 | -0.832 | 0.0245 | Yes |
Table: GSEA details [plain text format]

  

Fig 2: HALLMARK\_TGF\_BETA\_SIGNALING      
 Blue-Pink O' Gram in the Space of the Analyzed GeneSet

  

Fig 3: HALLMARK\_TGF\_BETA\_SIGNALING: Random ES distribution      
 Gene set null distribution of ES for **HALLMARK\_TGF\_BETA\_SIGNALING**
