# Supplementary material for: In vivo CRISPR screens reveal Serpinb9 and Adam2 as regulators of immune therapy response in lung cancer
Source: Nat Commun. 2023 May 31;14:3150. doi: 10.1038/s41467-023-38841-7 (PMC10232477; doi:10.1038/s41467-023-38841-7)
Supplement: Supplementary file 13 — Source Data [file 41467_2023_38841_MOESM13_ESM.zip › Figure 4E.zip!/my_analysis.Gsea.1640146340255_Hallmark/HALLMARK_MYC_TARGETS_V2.html]

Details for gene set HALLMARK\_MYC\_TARGETS\_V2[GSEA]

|  || Dataset | December 21, 2021\_for GSEA run\_collapsed\_to\_symbols.GSEA\_B6\_NI.cls #CTLR\_NI\_versus\_ADAM2\_NI |
| Phenotype | GSEA\_B6\_NI.cls#CTLR\_NI\_versus\_ADAM2\_NI |
| Upregulated in class | ADAM2\_NI |
| GeneSet | HALLMARK\_MYC\_TARGETS\_V2 |
| Enrichment Score (ES) | -0.50748795 |
| Normalized Enrichment Score (NES) | -1.2159233 |
| Nominal p-value | 0.22682446 |
| FDR q-value | 0.8350847 |
| FWER p-Value | 1.0 |
Table: GSEA Results Summary

  

Fig 1: Enrichment plot: HALLMARK\_MYC\_TARGETS\_V2      
 Profile of the Running ES Score & Positions of GeneSet Members on the Rank Ordered List

  

| SYMBOL | TITLE | RANK IN GENE LIST | RANK METRIC SCORE | RUNNING ES | CORE ENRICHMENT || 1 | MCM4 | minichromosome maintenance complex component 4 [Source:HGNC Symbol;Acc:HGNC:6947] | 3229 | 0.369 | -0.1798 | No |
| 2 | NOC4L | nucleolar complex associated 4 homolog [Source:HGNC Symbol;Acc:HGNC:28461] | 3549 | 0.321 | -0.1767 | No |
| 3 | PLK4 | polo like kinase 4 [Source:HGNC Symbol;Acc:HGNC:11397] | 5698 | 0.113 | -0.3060 | No |
| 4 | MAP3K6 | mitogen-activated protein kinase kinase kinase 6 [Source:HGNC Symbol;Acc:HGNC:6858] | 5792 | 0.105 | -0.3042 | No |
| 5 | GNL3 | G protein nucleolar 3 [Source:HGNC Symbol;Acc:HGNC:29931] | 5863 | 0.101 | -0.3013 | No |
| 6 | PLK1 | polo like kinase 1 [Source:HGNC Symbol;Acc:HGNC:9077] | 6259 | 0.073 | -0.3212 | No |
| 7 | MYC | "MYC proto-oncogene, bHLH transcription factor [Source:HGNC Symbol;Acc:HGNC:7553]" | 6504 | 0.055 | -0.3329 | No |
| 8 | HSPD1 | heat shock protein family D (Hsp60) member 1 [Source:HGNC Symbol;Acc:HGNC:5261] | 6586 | 0.050 | -0.3344 | No |
| 9 | TCOF1 | treacle ribosome biogenesis factor 1 [Source:HGNC Symbol;Acc:HGNC:11654] | 7801 | -0.019 | -0.4108 | No |
| 10 | NIP7 | nucleolar pre-rRNA processing protein NIP7 [Source:HGNC Symbol;Acc:HGNC:24328] | 7877 | -0.023 | -0.4140 | No |
| 11 | DDX18 | DEAD-box helicase 18 [Source:HGNC Symbol;Acc:HGNC:2741] | 7942 | -0.026 | -0.4162 | No |
| 12 | HSPE1 | heat shock protein family E (Hsp10) member 1 [Source:HGNC Symbol;Acc:HGNC:5269] | 8177 | -0.039 | -0.4283 | No |
| 13 | UTP20 | UTP20 small subunit processome component [Source:HGNC Symbol;Acc:HGNC:17897] | 8352 | -0.046 | -0.4361 | No |
| 14 | IMP4 | IMP U3 small nucleolar ribonucleoprotein 4 [Source:HGNC Symbol;Acc:HGNC:30856] | 8381 | -0.048 | -0.4344 | No |
| 15 | DUSP2 | dual specificity phosphatase 2 [Source:HGNC Symbol;Acc:HGNC:3068] | 8547 | -0.058 | -0.4407 | No |
| 16 | TFB2M | "transcription factor B2, mitochondrial [Source:HGNC Symbol;Acc:HGNC:18559]" | 8677 | -0.065 | -0.4442 | No |
| 17 | IPO4 | importin 4 [Source:HGNC Symbol;Acc:HGNC:19426] | 8932 | -0.078 | -0.4547 | No |
| 18 | RABEPK | Rab9 effector protein with kelch motifs [Source:HGNC Symbol;Acc:HGNC:16896] | 9233 | -0.095 | -0.4670 | No |
| 19 | MPHOSPH10 | M-phase phosphoprotein 10 [Source:HGNC Symbol;Acc:HGNC:7213] | 9491 | -0.110 | -0.4754 | No |
| 20 | SUPV3L1 | Suv3 like RNA helicase [Source:HGNC Symbol;Acc:HGNC:11471] | 9675 | -0.123 | -0.4781 | No |
| 21 | MCM5 | minichromosome maintenance complex component 5 [Source:HGNC Symbol;Acc:HGNC:6948] | 9727 | -0.126 | -0.4721 | No |
| 22 | PES1 | pescadillo ribosomal biogenesis factor 1 [Source:HGNC Symbol;Acc:HGNC:8848] | 9930 | -0.138 | -0.4749 | No |
| 23 | PA2G4 | proliferation-associated 2G4 [Source:HGNC Symbol;Acc:HGNC:8550] | 10108 | -0.148 | -0.4754 | No |
| 24 | NPM1 | nucleophosmin 1 [Source:HGNC Symbol;Acc:HGNC:7910] | 10355 | -0.163 | -0.4792 | No |
| 25 | NDUFAF4 | NADH:ubiquinone oxidoreductase complex assembly factor 4 [Source:HGNC Symbol;Acc:HGNC:21034] | 10439 | -0.168 | -0.4722 | No |
| 26 | AIMP2 | aminoacyl tRNA synthetase complex interacting multifunctional protein 2 [Source:HGNC Symbol;Acc:HGNC:20609] | 10991 | -0.200 | -0.4928 | Yes |
| 27 | PHB | prohibitin [Source:HGNC Symbol;Acc:HGNC:8912] | 11119 | -0.207 | -0.4858 | Yes |
| 28 | CBX3 | chromobox 3 [Source:HGNC Symbol;Acc:HGNC:1553] | 11298 | -0.217 | -0.4813 | Yes |
| 29 | TBRG4 | transforming growth factor beta regulator 4 [Source:HGNC Symbol;Acc:HGNC:17443] | 11363 | -0.221 | -0.4692 | Yes |
| 30 | HK2 | hexokinase 2 [Source:HGNC Symbol;Acc:HGNC:4923] | 11425 | -0.225 | -0.4566 | Yes |
| 31 | NOP16 | NOP16 nucleolar protein [Source:HGNC Symbol;Acc:HGNC:26934] | 11426 | -0.225 | -0.4401 | Yes |
| 32 | LAS1L | LAS1 like ribosome biogenesis factor [Source:HGNC Symbol;Acc:HGNC:25726] | 11558 | -0.232 | -0.4314 | Yes |
| 33 | EXOSC5 | exosome component 5 [Source:HGNC Symbol;Acc:HGNC:24662] | 11694 | -0.240 | -0.4225 | Yes |
| 34 | UNG | uracil DNA glycosylase [Source:HGNC Symbol;Acc:HGNC:12572] | 11714 | -0.242 | -0.4060 | Yes |
| 35 | SORD | sorbitol dehydrogenase [Source:HGNC Symbol;Acc:HGNC:11184] | 11863 | -0.252 | -0.3970 | Yes |
| 36 | WDR43 | WD repeat domain 43 [Source:HGNC Symbol;Acc:HGNC:28945] | 12187 | -0.273 | -0.3977 | Yes |
| 37 | MYBBP1A | MYB binding protein 1a [Source:HGNC Symbol;Acc:HGNC:7546] | 12198 | -0.273 | -0.3783 | Yes |
| 38 | SLC19A1 | solute carrier family 19 member 1 [Source:HGNC Symbol;Acc:HGNC:10937] | 12199 | -0.273 | -0.3583 | Yes |
| 39 | GRWD1 | glutamate rich WD repeat containing 1 [Source:HGNC Symbol;Acc:HGNC:21270] | 12361 | -0.285 | -0.3476 | Yes |
| 40 | MRTO4 | "MRT4 homolog, ribosome maturation factor [Source:HGNC Symbol;Acc:HGNC:18477]" | 12436 | -0.291 | -0.3310 | Yes |
| 41 | DCTPP1 | dCTP pyrophosphatase 1 [Source:HGNC Symbol;Acc:HGNC:28777] | 12471 | -0.294 | -0.3117 | Yes |
| 42 | TMEM97 | transmembrane protein 97 [Source:HGNC Symbol;Acc:HGNC:28106] | 12936 | -0.332 | -0.3171 | Yes |
| 43 | RRP9 | "ribosomal RNA processing 9, U3 small nucleolar RNA binding protein [Source:HGNC Symbol;Acc:HGNC:16829]" | 12955 | -0.334 | -0.2937 | Yes |
| 44 | NOP56 | NOP56 ribonucleoprotein [Source:HGNC Symbol;Acc:HGNC:15911] | 13004 | -0.338 | -0.2720 | Yes |
| 45 | PUS1 | pseudouridine synthase 1 [Source:HGNC Symbol;Acc:HGNC:15508] | 13066 | -0.344 | -0.2507 | Yes |
| 46 | WDR74 | WD repeat domain 74 [Source:HGNC Symbol;Acc:HGNC:25529] | 13280 | -0.365 | -0.2376 | Yes |
| 47 | PRMT3 | protein arginine methyltransferase 3 [Source:HGNC Symbol;Acc:HGNC:30163] | 13374 | -0.377 | -0.2159 | Yes |
| 48 | FARSA | phenylalanyl-tRNA synthetase subunit alpha [Source:HGNC Symbol;Acc:HGNC:3592] | 13418 | -0.381 | -0.1908 | Yes |
| 49 | RRP12 | ribosomal RNA processing 12 homolog [Source:HGNC Symbol;Acc:HGNC:29100] | 13536 | -0.397 | -0.1692 | Yes |
| 50 | CDK4 | cyclin dependent kinase 4 [Source:HGNC Symbol;Acc:HGNC:1773] | 13588 | -0.403 | -0.1429 | Yes |
| 51 | SLC29A2 | solute carrier family 29 member 2 [Source:HGNC Symbol;Acc:HGNC:11004] | 13975 | -0.428 | -0.1362 | Yes |
| 52 | BYSL | bystin like [Source:HGNC Symbol;Acc:HGNC:1157] | 14113 | -0.451 | -0.1119 | Yes |
| 53 | NOLC1 | nucleolar and coiled-body phosphoprotein 1 [Source:HGNC Symbol;Acc:HGNC:15608] | 14380 | -0.490 | -0.0930 | Yes |
| 54 | PPRC1 | PPARG related coactivator 1 [Source:HGNC Symbol;Acc:HGNC:30025] | 14445 | -0.503 | -0.0602 | Yes |
| 55 | NOP2 | NOP2 nucleolar protein [Source:HGNC Symbol;Acc:HGNC:7867] | 14568 | -0.527 | -0.0294 | Yes |
| 56 | SRM | spermidine synthase [Source:HGNC Symbol;Acc:HGNC:11296] | 14841 | -0.596 | -0.0031 | Yes |
| 57 | RCL1 | RNA terminal phosphate cyclase like 1 [Source:HGNC Symbol;Acc:HGNC:17687] | 15178 | -0.764 | 0.0314 | Yes |
Table: GSEA details [plain text format]

  

Fig 2: HALLMARK\_MYC\_TARGETS\_V2      
 Blue-Pink O' Gram in the Space of the Analyzed GeneSet

  

Fig 3: HALLMARK\_MYC\_TARGETS\_V2: Random ES distribution      
 Gene set null distribution of ES for **HALLMARK\_MYC\_TARGETS\_V2**
