# Supplementary material for: In vivo CRISPR screens reveal Serpinb9 and Adam2 as regulators of immune therapy response in lung cancer
Source: Nat Commun. 2023 May 31;14:3150. doi: 10.1038/s41467-023-38841-7 (PMC10232477; doi:10.1038/s41467-023-38841-7)
Supplement: Supplementary file 13 — Source Data [file 41467_2023_38841_MOESM13_ESM.zip › Figure 4E.zip!/my_analysis.Gsea.1640146340255_Hallmark/HALLMARK_ANGIOGENESIS.html]

Details for gene set HALLMARK\_ANGIOGENESIS[GSEA]

|  || Dataset | December 21, 2021\_for GSEA run\_collapsed\_to\_symbols.GSEA\_B6\_NI.cls #CTLR\_NI\_versus\_ADAM2\_NI |
| Phenotype | GSEA\_B6\_NI.cls#CTLR\_NI\_versus\_ADAM2\_NI |
| Upregulated in class | ADAM2\_NI |
| GeneSet | HALLMARK\_ANGIOGENESIS |
| Enrichment Score (ES) | -0.3671493 |
| Normalized Enrichment Score (NES) | -1.1070925 |
| Nominal p-value | 0.28395063 |
| FDR q-value | 0.86793065 |
| FWER p-Value | 1.0 |
Table: GSEA Results Summary

  

Fig 1: Enrichment plot: HALLMARK\_ANGIOGENESIS      
 Profile of the Running ES Score & Positions of GeneSet Members on the Rank Ordered List

  

| SYMBOL | TITLE | RANK IN GENE LIST | RANK METRIC SCORE | RUNNING ES | CORE ENRICHMENT || 1 | POSTN | periostin [Source:HGNC Symbol;Acc:HGNC:16953] | 814 | 0.871 | 0.0097 | No |
| 2 | LPL | lipoprotein lipase [Source:HGNC Symbol;Acc:HGNC:6677] | 819 | 0.867 | 0.0710 | No |
| 3 | FSTL1 | follistatin like 1 [Source:HGNC Symbol;Acc:HGNC:3972] | 1122 | 0.763 | 0.1058 | No |
| 4 | JAG1 | jagged canonical Notch ligand 1 [Source:HGNC Symbol;Acc:HGNC:6188] | 1788 | 0.546 | 0.1020 | No |
| 5 | LUM | lumican [Source:HGNC Symbol;Acc:HGNC:6724] | 1837 | 0.536 | 0.1370 | No |
| 6 | COL5A2 | collagen type V alpha 2 chain [Source:HGNC Symbol;Acc:HGNC:2210] | 2294 | 0.468 | 0.1411 | No |
| 7 | APP | amyloid beta precursor protein [Source:HGNC Symbol;Acc:HGNC:620] | 2788 | 0.418 | 0.1392 | No |
| 8 | SLCO2A1 | solute carrier organic anion transporter family member 2A1 [Source:HGNC Symbol;Acc:HGNC:10955] | 3331 | 0.352 | 0.1295 | No |
| 9 | OLR1 | oxidized low density lipoprotein receptor 1 [Source:HGNC Symbol;Acc:HGNC:8133] | 4464 | 0.217 | 0.0725 | No |
| 10 | CXCL6 | C-X-C motif chemokine ligand 6 [Source:HGNC Symbol;Acc:HGNC:10643] | 4655 | 0.200 | 0.0746 | No |
| 11 | VCAN | versican [Source:HGNC Symbol;Acc:HGNC:2464] | 4697 | 0.197 | 0.0859 | No |
| 12 | PF4 | platelet factor 4 [Source:HGNC Symbol;Acc:HGNC:8861] | 4836 | 0.184 | 0.0902 | No |
| 13 | TIMP1 | TIMP metallopeptidase inhibitor 1 [Source:HGNC Symbol;Acc:HGNC:11820] | 4971 | 0.171 | 0.0937 | No |
| 14 | TNFRSF21 | TNF receptor superfamily member 21 [Source:HGNC Symbol;Acc:HGNC:13469] | 6692 | 0.043 | -0.0132 | No |
| 15 | PTK2 | protein tyrosine kinase 2 [Source:HGNC Symbol;Acc:HGNC:9611] | 7196 | 0.012 | -0.0445 | No |
| 16 | VAV2 | vav guanine nucleotide exchange factor 2 [Source:HGNC Symbol;Acc:HGNC:12658] | 7575 | -0.008 | -0.0682 | No |
| 17 | VEGFA | vascular endothelial growth factor A [Source:HGNC Symbol;Acc:HGNC:12680] | 7671 | -0.012 | -0.0734 | No |
| 18 | CCND2 | cyclin D2 [Source:HGNC Symbol;Acc:HGNC:1583] | 8119 | -0.036 | -0.0994 | No |
| 19 | NRP1 | neuropilin 1 [Source:HGNC Symbol;Acc:HGNC:8004] | 9151 | -0.091 | -0.1589 | No |
| 20 | ITGAV | integrin subunit alpha V [Source:HGNC Symbol;Acc:HGNC:6150] | 9955 | -0.139 | -0.2004 | No |
| 21 | COL3A1 | collagen type III alpha 1 chain [Source:HGNC Symbol;Acc:HGNC:2201] | 10161 | -0.151 | -0.2028 | No |
| 22 | VTN | vitronectin [Source:HGNC Symbol;Acc:HGNC:12724] | 10896 | -0.194 | -0.2360 | No |
| 23 | LRPAP1 | LDL receptor related protein associated protein 1 [Source:HGNC Symbol;Acc:HGNC:6701] | 11327 | -0.219 | -0.2480 | No |
| 24 | APOH | apolipoprotein H [Source:HGNC Symbol;Acc:HGNC:616] | 11982 | -0.260 | -0.2714 | No |
| 25 | JAG2 | jagged canonical Notch ligand 2 [Source:HGNC Symbol;Acc:HGNC:6189] | 12440 | -0.291 | -0.2799 | No |
| 26 | PDGFA | platelet derived growth factor subunit A [Source:HGNC Symbol;Acc:HGNC:8799] | 12465 | -0.293 | -0.2606 | No |
| 27 | FGFR1 | fibroblast growth factor receptor 1 [Source:HGNC Symbol;Acc:HGNC:3688] | 13994 | -0.431 | -0.3278 | No |
| 28 | SPP1 | secreted phosphoprotein 1 [Source:HGNC Symbol;Acc:HGNC:11255] | 14610 | -0.535 | -0.3292 | Yes |
| 29 | PRG2 | "proteoglycan 2, pro eosinophil major basic protein [Source:HGNC Symbol;Acc:HGNC:9362]" | 14762 | -0.571 | -0.2983 | Yes |
| 30 | THBD | thrombomodulin [Source:HGNC Symbol;Acc:HGNC:11784] | 14835 | -0.594 | -0.2607 | Yes |
| 31 | PGLYRP1 | peptidoglycan recognition protein 1 [Source:HGNC Symbol;Acc:HGNC:8904] | 14990 | -0.660 | -0.2237 | Yes |
| 32 | S100A4 | S100 calcium binding protein A4 [Source:HGNC Symbol;Acc:HGNC:10494] | 15340 | -0.855 | -0.1854 | Yes |
| 33 | MSX1 | msh homeobox 1 [Source:HGNC Symbol;Acc:HGNC:7391] | 15355 | -0.863 | -0.1251 | Yes |
| 34 | STC1 | stanniocalcin 1 [Source:HGNC Symbol;Acc:HGNC:11373] | 15482 | -1.009 | -0.0615 | Yes |
| 35 | KCNJ8 | potassium inwardly rectifying channel subfamily J member 8 [Source:HGNC Symbol;Acc:HGNC:6269] | 15502 | -1.034 | 0.0106 | Yes |
Table: GSEA details [plain text format]

  

Fig 2: HALLMARK\_ANGIOGENESIS      
 Blue-Pink O' Gram in the Space of the Analyzed GeneSet

  

Fig 3: HALLMARK\_ANGIOGENESIS: Random ES distribution      
 Gene set null distribution of ES for **HALLMARK\_ANGIOGENESIS**
