# Supplementary material for: In vivo CRISPR screens reveal Serpinb9 and Adam2 as regulators of immune therapy response in lung cancer
Source: Nat Commun. 2023 May 31;14:3150. doi: 10.1038/s41467-023-38841-7 (PMC10232477; doi:10.1038/s41467-023-38841-7)
Supplement: Supplementary file 13 — Source Data [file 41467_2023_38841_MOESM13_ESM.zip › Figure 4E.zip!/my_analysis.Gsea.1640146340255_Hallmark/HALLMARK_UV_RESPONSE_DN.html]

Details for gene set HALLMARK\_UV\_RESPONSE\_DN[GSEA]

|  || Dataset | December 21, 2021\_for GSEA run\_collapsed\_to\_symbols.GSEA\_B6\_NI.cls #CTLR\_NI\_versus\_ADAM2\_NI |
| Phenotype | GSEA\_B6\_NI.cls#CTLR\_NI\_versus\_ADAM2\_NI |
| Upregulated in class | ADAM2\_NI |
| GeneSet | HALLMARK\_UV\_RESPONSE\_DN |
| Enrichment Score (ES) | -0.26747134 |
| Normalized Enrichment Score (NES) | -1.0584346 |
| Nominal p-value | 0.33778626 |
| FDR q-value | 0.8095754 |
| FWER p-Value | 1.0 |
Table: GSEA Results Summary

  

Fig 1: Enrichment plot: HALLMARK\_UV\_RESPONSE\_DN      
 Profile of the Running ES Score & Positions of GeneSet Members on the Rank Ordered List

  

| SYMBOL | TITLE | RANK IN GENE LIST | RANK METRIC SCORE | RUNNING ES | CORE ENRICHMENT || 1 | SCN8A | sodium voltage-gated channel alpha subunit 8 [Source:HGNC Symbol;Acc:HGNC:10596] | 57 | 1.981 | 0.0388 | No |
| 2 | PPARG | peroxisome proliferator activated receptor gamma [Source:HGNC Symbol;Acc:HGNC:9236] | 251 | 1.379 | 0.0560 | No |
| 3 | EFEMP1 | EGF containing fibulin extracellular matrix protein 1 [Source:HGNC Symbol;Acc:HGNC:3218] | 285 | 1.332 | 0.0824 | No |
| 4 | KCNMA1 | potassium calcium-activated channel subfamily M alpha 1 [Source:HGNC Symbol;Acc:HGNC:6284] | 373 | 1.205 | 0.1027 | No |
| 5 | HAS2 | hyaluronan synthase 2 [Source:HGNC Symbol;Acc:HGNC:4819] | 676 | 0.948 | 0.1035 | No |
| 6 | SLC22A18 | solute carrier family 22 member 18 [Source:HGNC Symbol;Acc:HGNC:10964] | 696 | 0.937 | 0.1224 | No |
| 7 | IGFBP5 | insulin like growth factor binding protein 5 [Source:HGNC Symbol;Acc:HGNC:5474] | 1124 | 0.762 | 0.1112 | No |
| 8 | INPP4B | inositol polyphosphate-4-phosphatase type II B [Source:HGNC Symbol;Acc:HGNC:6075] | 1292 | 0.698 | 0.1154 | No |
| 9 | PTGFR | prostaglandin F receptor [Source:HGNC Symbol;Acc:HGNC:9600] | 1304 | 0.695 | 0.1296 | No |
| 10 | COL1A2 | collagen type I alpha 2 chain [Source:HGNC Symbol;Acc:HGNC:2198] | 1673 | 0.574 | 0.1182 | No |
| 11 | ADORA2B | adenosine A2b receptor [Source:HGNC Symbol;Acc:HGNC:264] | 1789 | 0.546 | 0.1225 | No |
| 12 | ARHGEF9 | Cdc42 guanine nucleotide exchange factor 9 [Source:HGNC Symbol;Acc:HGNC:14561] | 1898 | 0.525 | 0.1269 | No |
| 13 | COL11A1 | collagen type XI alpha 1 chain [Source:HGNC Symbol;Acc:HGNC:2186] | 1935 | 0.516 | 0.1356 | No |
| 14 | COL1A1 | collagen type I alpha 1 chain [Source:HGNC Symbol;Acc:HGNC:2197] | 1960 | 0.511 | 0.1450 | No |
| 15 | FYN | "FYN proto-oncogene, Src family tyrosine kinase [Source:HGNC Symbol;Acc:HGNC:4037]" | 2288 | 0.469 | 0.1340 | No |
| 16 | COL5A2 | collagen type V alpha 2 chain [Source:HGNC Symbol;Acc:HGNC:2210] | 2294 | 0.468 | 0.1437 | No |
| 17 | DAB2 | DAB adaptor protein 2 [Source:HGNC Symbol;Acc:HGNC:2662] | 3069 | 0.393 | 0.1023 | No |
| 18 | WDR37 | WD repeat domain 37 [Source:HGNC Symbol;Acc:HGNC:31406] | 3075 | 0.392 | 0.1104 | No |
| 19 | MET | "MET proto-oncogene, receptor tyrosine kinase [Source:HGNC Symbol;Acc:HGNC:7029]" | 3190 | 0.374 | 0.1111 | No |
| 20 | PIK3CD | "phosphatidylinositol-4,5-bisphosphate 3-kinase catalytic subunit delta [Source:HGNC Symbol;Acc:HGNC:8977]" | 3392 | 0.343 | 0.1055 | No |
| 21 | CELF2 | CUGBP Elav-like family member 2 [Source:HGNC Symbol;Acc:HGNC:2550] | 3434 | 0.337 | 0.1101 | No |
| 22 | KALRN | kalirin RhoGEF kinase [Source:HGNC Symbol;Acc:HGNC:4814] | 3498 | 0.328 | 0.1130 | No |
| 23 | ATP2B4 | ATPase plasma membrane Ca2+ transporting 4 [Source:HGNC Symbol;Acc:HGNC:817] | 3648 | 0.308 | 0.1100 | No |
| 24 | SFMBT1 | Scm like with four mbt domains 1 [Source:HGNC Symbol;Acc:HGNC:20255] | 3820 | 0.289 | 0.1052 | No |
| 25 | AKT3 | AKT serine/threonine kinase 3 [Source:HGNC Symbol;Acc:HGNC:393] | 4169 | 0.247 | 0.0881 | No |
| 26 | IRS1 | insulin receptor substrate 1 [Source:HGNC Symbol;Acc:HGNC:6125] | 4220 | 0.241 | 0.0900 | No |
| 27 | RASA2 | RAS p21 protein activator 2 [Source:HGNC Symbol;Acc:HGNC:9872] | 4250 | 0.237 | 0.0932 | No |
| 28 | DYRK1A | dual specificity tyrosine phosphorylation regulated kinase 1A [Source:HGNC Symbol;Acc:HGNC:3091] | 4291 | 0.233 | 0.0957 | No |
| 29 | TOGARAM1 | TOG array regulator of axonemal microtubules 1 [Source:HGNC Symbol;Acc:HGNC:19959] | 4641 | 0.201 | 0.0775 | No |
| 30 | ACVR2A | activin A receptor type 2A [Source:HGNC Symbol;Acc:HGNC:173] | 4777 | 0.190 | 0.0729 | No |
| 31 | RUNX1 | RUNX family transcription factor 1 [Source:HGNC Symbol;Acc:HGNC:10471] | 4789 | 0.189 | 0.0762 | No |
| 32 | NIPBL | NIPBL cohesin loading factor [Source:HGNC Symbol;Acc:HGNC:28862] | 4807 | 0.187 | 0.0791 | No |
| 33 | PLCB4 | phospholipase C beta 4 [Source:HGNC Symbol;Acc:HGNC:9059] | 4876 | 0.181 | 0.0786 | No |
| 34 | CDKN1B | cyclin dependent kinase inhibitor 1B [Source:HGNC Symbol;Acc:HGNC:1785] | 4931 | 0.175 | 0.0789 | No |
| 35 | ATXN1 | ataxin 1 [Source:HGNC Symbol;Acc:HGNC:10548] | 5203 | 0.151 | 0.0647 | No |
| 36 | MAP1B | microtubule associated protein 1B [Source:HGNC Symbol;Acc:HGNC:6836] | 5244 | 0.147 | 0.0652 | No |
| 37 | BMPR1A | bone morphogenetic protein receptor type 1A [Source:HGNC Symbol;Acc:HGNC:1076] | 5304 | 0.141 | 0.0645 | No |
| 38 | PMP22 | peripheral myelin protein 22 [Source:HGNC Symbol;Acc:HGNC:9118] | 5531 | 0.125 | 0.0526 | No |
| 39 | KIT | "KIT proto-oncogene, receptor tyrosine kinase [Source:HGNC Symbol;Acc:HGNC:6342]" | 5564 | 0.122 | 0.0532 | No |
| 40 | ITGB3 | integrin subunit beta 3 [Source:HGNC Symbol;Acc:HGNC:6156] | 5695 | 0.113 | 0.0472 | No |
| 41 | NR1D2 | nuclear receptor subfamily 1 group D member 2 [Source:HGNC Symbol;Acc:HGNC:7963] | 5757 | 0.108 | 0.0456 | No |
| 42 | NR3C1 | nuclear receptor subfamily 3 group C member 1 [Source:HGNC Symbol;Acc:HGNC:7978] | 5843 | 0.102 | 0.0423 | No |
| 43 | SMAD7 | SMAD family member 7 [Source:HGNC Symbol;Acc:HGNC:6773] | 5911 | 0.098 | 0.0401 | No |
| 44 | ATRX | ATRX chromatin remodeler [Source:HGNC Symbol;Acc:HGNC:886] | 5944 | 0.095 | 0.0401 | No |
| 45 | BCKDHB | branched chain keto acid dehydrogenase E1 subunit beta [Source:HGNC Symbol;Acc:HGNC:987] | 6040 | 0.088 | 0.0359 | No |
| 46 | SNAI2 | snail family transcriptional repressor 2 [Source:HGNC Symbol;Acc:HGNC:11094] | 6041 | 0.088 | 0.0378 | No |
| 47 | CDK13 | cyclin dependent kinase 13 [Source:HGNC Symbol;Acc:HGNC:1733] | 6070 | 0.085 | 0.0378 | No |
| 48 | DLG1 | discs large MAGUK scaffold protein 1 [Source:HGNC Symbol;Acc:HGNC:2900] | 6093 | 0.083 | 0.0381 | No |
| 49 | PRKCE | protein kinase C epsilon [Source:HGNC Symbol;Acc:HGNC:9401] | 6431 | 0.060 | 0.0177 | No |
| 50 | MYC | "MYC proto-oncogene, bHLH transcription factor [Source:HGNC Symbol;Acc:HGNC:7553]" | 6504 | 0.055 | 0.0143 | No |
| 51 | YTHDC1 | YTH domain containing 1 [Source:HGNC Symbol;Acc:HGNC:30626] | 6537 | 0.053 | 0.0133 | No |
| 52 | PTPN21 | protein tyrosine phosphatase non-receptor type 21 [Source:HGNC Symbol;Acc:HGNC:9651] | 6717 | 0.041 | 0.0027 | No |
| 53 | NFKB1 | nuclear factor kappa B subunit 1 [Source:HGNC Symbol;Acc:HGNC:7794] | 6748 | 0.040 | 0.0016 | No |
| 54 | ATP2B1 | ATPase plasma membrane Ca2+ transporting 1 [Source:HGNC Symbol;Acc:HGNC:814] | 6837 | 0.034 | -0.0033 | No |
| 55 | GRK5 | G protein-coupled receptor kinase 5 [Source:HGNC Symbol;Acc:HGNC:4544] | 6898 | 0.029 | -0.0066 | No |
| 56 | ABCC1 | ATP binding cassette subfamily C member 1 [Source:HGNC Symbol;Acc:HGNC:51] | 6987 | 0.025 | -0.0117 | No |
| 57 | CDC42BPA | CDC42 binding protein kinase alpha [Source:HGNC Symbol;Acc:HGNC:1737] | 7022 | 0.023 | -0.0134 | No |
| 58 | SPOP | speckle type BTB/POZ protein [Source:HGNC Symbol;Acc:HGNC:11254] | 7113 | 0.017 | -0.0188 | No |
| 59 | VLDLR | very low density lipoprotein receptor [Source:HGNC Symbol;Acc:HGNC:12698] | 7123 | 0.017 | -0.0190 | No |
| 60 | PDLIM5 | PDZ and LIM domain 5 [Source:HGNC Symbol;Acc:HGNC:17468] | 7279 | 0.008 | -0.0289 | No |
| 61 | ATP2C1 | ATPase secretory pathway Ca2+ transporting 1 [Source:HGNC Symbol;Acc:HGNC:13211] | 7381 | 0.002 | -0.0353 | No |
| 62 | VAV2 | vav guanine nucleotide exchange factor 2 [Source:HGNC Symbol;Acc:HGNC:12658] | 7575 | -0.008 | -0.0476 | No |
| 63 | SRI | sorcin [Source:HGNC Symbol;Acc:HGNC:11292] | 7595 | -0.009 | -0.0486 | No |
| 64 | CAP2 | cyclase associated actin cytoskeleton regulatory protein 2 [Source:HGNC Symbol;Acc:HGNC:20039] | 7640 | -0.010 | -0.0512 | No |
| 65 | MAPK14 | mitogen-activated protein kinase 14 [Source:HGNC Symbol;Acc:HGNC:6876] | 7662 | -0.011 | -0.0524 | No |
| 66 | PHF3 | PHD finger protein 3 [Source:HGNC Symbol;Acc:HGNC:8921] | 7773 | -0.017 | -0.0591 | No |
| 67 | DLC1 | DLC1 Rho GTPase activating protein [Source:HGNC Symbol;Acc:HGNC:2897] | 7856 | -0.021 | -0.0639 | No |
| 68 | LAMC1 | laminin subunit gamma 1 [Source:HGNC Symbol;Acc:HGNC:6492] | 8012 | -0.029 | -0.0732 | No |
| 69 | AGGF1 | angiogenic factor with G-patch and FHA domains 1 [Source:HGNC Symbol;Acc:HGNC:24684] | 8067 | -0.032 | -0.0760 | No |
| 70 | ANXA4 | annexin A4 [Source:HGNC Symbol;Acc:HGNC:542] | 8076 | -0.033 | -0.0758 | No |
| 71 | NFIB | nuclear factor I B [Source:HGNC Symbol;Acc:HGNC:7785] | 8166 | -0.038 | -0.0807 | No |
| 72 | TJP1 | tight junction protein 1 [Source:HGNC Symbol;Acc:HGNC:11827] | 8203 | -0.040 | -0.0822 | No |
| 73 | AMPH | amphiphysin [Source:HGNC Symbol;Acc:HGNC:471] | 8254 | -0.042 | -0.0845 | No |
| 74 | TENT4A | terminal nucleotidyltransferase 4A [Source:HGNC Symbol;Acc:HGNC:16705] | 8320 | -0.045 | -0.0877 | No |
| 75 | FZD2 | frizzled class receptor 2 [Source:HGNC Symbol;Acc:HGNC:4040] | 8696 | -0.066 | -0.1105 | No |
| 76 | SIPA1L1 | signal induced proliferation associated 1 like 1 [Source:HGNC Symbol;Acc:HGNC:20284] | 8990 | -0.082 | -0.1276 | No |
| 77 | PRDM2 | PR/SET domain 2 [Source:HGNC Symbol;Acc:HGNC:9347] | 9144 | -0.090 | -0.1355 | No |
| 78 | NRP1 | neuropilin 1 [Source:HGNC Symbol;Acc:HGNC:8004] | 9151 | -0.091 | -0.1340 | No |
| 79 | GCNT1 | glucosaminyl (N-acetyl) transferase 1 [Source:HGNC Symbol;Acc:HGNC:4203] | 9214 | -0.094 | -0.1359 | No |
| 80 | PLPP3 | phospholipid phosphatase 3 [Source:HGNC Symbol;Acc:HGNC:9229] | 9319 | -0.099 | -0.1405 | No |
| 81 | ZMIZ1 | zinc finger MIZ-type containing 1 [Source:HGNC Symbol;Acc:HGNC:16493] | 9517 | -0.112 | -0.1508 | No |
| 82 | DDAH1 | dimethylarginine dimethylaminohydrolase 1 [Source:HGNC Symbol;Acc:HGNC:2715] | 9621 | -0.120 | -0.1549 | No |
| 83 | NEK7 | NIMA related kinase 7 [Source:HGNC Symbol;Acc:HGNC:13386] | 9706 | -0.125 | -0.1576 | No |
| 84 | DMAC2L | distal membrane arm assembly complex 2 like [Source:HGNC Symbol;Acc:HGNC:18799] | 9734 | -0.127 | -0.1566 | No |
| 85 | NOTCH2 | notch receptor 2 [Source:HGNC Symbol;Acc:HGNC:7882] | 9770 | -0.129 | -0.1561 | No |
| 86 | SERPINE1 | serpin family E member 1 [Source:HGNC Symbol;Acc:HGNC:8583] | 10052 | -0.145 | -0.1711 | No |
| 87 | MRPS31 | mitochondrial ribosomal protein S31 [Source:HGNC Symbol;Acc:HGNC:16632] | 10086 | -0.147 | -0.1701 | No |
| 88 | ANXA2 | annexin A2 [Source:HGNC Symbol;Acc:HGNC:537] | 10100 | -0.148 | -0.1677 | No |
| 89 | COL3A1 | collagen type III alpha 1 chain [Source:HGNC Symbol;Acc:HGNC:2201] | 10161 | -0.151 | -0.1684 | No |
| 90 | ATRN | attractin [Source:HGNC Symbol;Acc:HGNC:885] | 10170 | -0.152 | -0.1656 | No |
| 91 | SYNE1 | spectrin repeat containing nuclear envelope protein 1 [Source:HGNC Symbol;Acc:HGNC:17089] | 10195 | -0.154 | -0.1639 | No |
| 92 | TFPI | tissue factor pathway inhibitor [Source:HGNC Symbol;Acc:HGNC:11760] | 10316 | -0.160 | -0.1682 | No |
| 93 | MAP2K5 | mitogen-activated protein kinase kinase 5 [Source:HGNC Symbol;Acc:HGNC:6845] | 10493 | -0.171 | -0.1758 | No |
| 94 | PIK3R3 | phosphoinositide-3-kinase regulatory subunit 3 [Source:HGNC Symbol;Acc:HGNC:8981] | 10512 | -0.172 | -0.1733 | No |
| 95 | F3 | "coagulation factor III, tissue factor [Source:HGNC Symbol;Acc:HGNC:3541]" | 10575 | -0.175 | -0.1735 | No |
| 96 | ID1 | "inhibitor of DNA binding 1, HLH protein [Source:HGNC Symbol;Acc:HGNC:5360]" | 10637 | -0.179 | -0.1736 | No |
| 97 | SLC7A1 | solute carrier family 7 member 1 [Source:HGNC Symbol;Acc:HGNC:11057] | 10918 | -0.196 | -0.1874 | No |
| 98 | APBB2 | amyloid beta precursor protein binding family B member 2 [Source:HGNC Symbol;Acc:HGNC:582] | 11021 | -0.201 | -0.1897 | No |
| 99 | MIOS | meiosis regulator for oocyte development [Source:HGNC Symbol;Acc:HGNC:21905] | 11030 | -0.202 | -0.1859 | No |
| 100 | PTEN | phosphatase and tensin homolog [Source:HGNC Symbol;Acc:HGNC:9588] | 11136 | -0.208 | -0.1882 | No |
| 101 | PRKAR2B | protein kinase cAMP-dependent type II regulatory subunit beta [Source:HGNC Symbol;Acc:HGNC:9392] | 11261 | -0.215 | -0.1916 | No |
| 102 | IGF1R | insulin like growth factor 1 receptor [Source:HGNC Symbol;Acc:HGNC:5465] | 11329 | -0.219 | -0.1912 | No |
| 103 | MGLL | monoglyceride lipase [Source:HGNC Symbol;Acc:HGNC:17038] | 11367 | -0.222 | -0.1888 | No |
| 104 | PEX14 | peroxisomal biogenesis factor 14 [Source:HGNC Symbol;Acc:HGNC:8856] | 11500 | -0.229 | -0.1924 | No |
| 105 | SCAF8 | SR-related CTD associated factor 8 [Source:HGNC Symbol;Acc:HGNC:20959] | 11788 | -0.247 | -0.2056 | No |
| 106 | GJA1 | gap junction protein alpha 1 [Source:HGNC Symbol;Acc:HGNC:4274] | 12277 | -0.279 | -0.2310 | No |
| 107 | TGFBR3 | transforming growth factor beta receptor 3 [Source:HGNC Symbol;Acc:HGNC:11774] | 12373 | -0.286 | -0.2310 | No |
| 108 | RBPMS | "RNA binding protein, mRNA processing factor [Source:HGNC Symbol;Acc:HGNC:19097]" | 12410 | -0.289 | -0.2271 | No |
| 109 | CITED2 | Cbp/p300 interacting transactivator with Glu/Asp rich carboxy-terminal domain 2 [Source:HGNC Symbol;Acc:HGNC:1987] | 12508 | -0.296 | -0.2270 | No |
| 110 | TGFBR2 | transforming growth factor beta receptor 2 [Source:HGNC Symbol;Acc:HGNC:11773] | 12670 | -0.309 | -0.2308 | No |
| 111 | RND3 | Rho family GTPase 3 [Source:HGNC Symbol;Acc:HGNC:671] | 12679 | -0.310 | -0.2246 | No |
| 112 | ADD3 | adducin 3 [Source:HGNC Symbol;Acc:HGNC:245] | 12843 | -0.323 | -0.2282 | No |
| 113 | LTBP1 | latent transforming growth factor beta binding protein 1 [Source:HGNC Symbol;Acc:HGNC:6714] | 12878 | -0.326 | -0.2234 | No |
| 114 | MTA1 | metastasis associated 1 [Source:HGNC Symbol;Acc:HGNC:7410] | 12953 | -0.334 | -0.2210 | No |
| 115 | ERBB2 | erb-b2 receptor tyrosine kinase 2 [Source:HGNC Symbol;Acc:HGNC:3430] | 13227 | -0.359 | -0.2309 | No |
| 116 | CCN1 | cellular communication network factor 1 [Source:HGNC Symbol;Acc:HGNC:2654] | 13488 | -0.391 | -0.2393 | No |
| 117 | MMP16 | matrix metallopeptidase 16 [Source:HGNC Symbol;Acc:HGNC:7162] | 13927 | -0.428 | -0.2583 | Yes |
| 118 | INSIG1 | insulin induced gene 1 [Source:HGNC Symbol;Acc:HGNC:6083] | 13987 | -0.430 | -0.2529 | Yes |
| 119 | BHLHE40 | basic helix-loop-helix family member e40 [Source:HGNC Symbol;Acc:HGNC:1046] | 14032 | -0.437 | -0.2463 | Yes |
| 120 | LDLR | low density lipoprotein receptor [Source:HGNC Symbol;Acc:HGNC:6547] | 14103 | -0.450 | -0.2412 | Yes |
| 121 | MAGI2 | "membrane associated guanylate kinase, WW and PDZ domain containing 2 [Source:HGNC Symbol;Acc:HGNC:18957]" | 14130 | -0.454 | -0.2331 | Yes |
| 122 | PIAS3 | protein inhibitor of activated STAT 3 [Source:HGNC Symbol;Acc:HGNC:16861] | 14407 | -0.493 | -0.2403 | Yes |
| 123 | DUSP1 | dual specificity phosphatase 1 [Source:HGNC Symbol;Acc:HGNC:3064] | 14433 | -0.499 | -0.2313 | Yes |
| 124 | BDNF | brain derived neurotrophic factor [Source:HGNC Symbol;Acc:HGNC:1033] | 14450 | -0.504 | -0.2215 | Yes |
| 125 | PRKCA | protein kinase C alpha [Source:HGNC Symbol;Acc:HGNC:9393] | 14498 | -0.513 | -0.2135 | Yes |
| 126 | PDGFRB | platelet derived growth factor receptor beta [Source:HGNC Symbol;Acc:HGNC:8804] | 14530 | -0.521 | -0.2043 | Yes |
| 127 | RXRA | retinoid X receptor alpha [Source:HGNC Symbol;Acc:HGNC:10477] | 14541 | -0.522 | -0.1938 | Yes |
| 128 | SDC2 | syndecan 2 [Source:HGNC Symbol;Acc:HGNC:10659] | 14631 | -0.539 | -0.1880 | Yes |
| 129 | SYNJ2 | synaptojanin 2 [Source:HGNC Symbol;Acc:HGNC:11504] | 14827 | -0.592 | -0.1878 | Yes |
| 130 | SCHIP1 | schwannomin interacting protein 1 [Source:HGNC Symbol;Acc:HGNC:15678] | 14838 | -0.595 | -0.1757 | Yes |
| 131 | RGS4 | regulator of G protein signaling 4 [Source:HGNC Symbol;Acc:HGNC:10000] | 14914 | -0.625 | -0.1672 | Yes |
| 132 | CDON | "cell adhesion associated, oncogene regulated [Source:HGNC Symbol;Acc:HGNC:17104]" | 14957 | -0.646 | -0.1560 | Yes |
| 133 | FHL2 | four and a half LIM domains 2 [Source:HGNC Symbol;Acc:HGNC:3703] | 14993 | -0.661 | -0.1441 | Yes |
| 134 | ICA1 | islet cell autoantigen 1 [Source:HGNC Symbol;Acc:HGNC:5343] | 15045 | -0.684 | -0.1327 | Yes |
| 135 | CAV1 | caveolin 1 [Source:HGNC Symbol;Acc:HGNC:1527] | 15057 | -0.694 | -0.1185 | Yes |
| 136 | DBP | D-box binding PAR bZIP transcription factor [Source:HGNC Symbol;Acc:HGNC:2697] | 15157 | -0.754 | -0.1087 | Yes |
| 137 | SMAD3 | SMAD family member 3 [Source:HGNC Symbol;Acc:HGNC:6769] | 15161 | -0.755 | -0.0927 | Yes |
| 138 | LPAR1 | lysophosphatidic acid receptor 1 [Source:HGNC Symbol;Acc:HGNC:3166] | 15191 | -0.773 | -0.0780 | Yes |
| 139 | PTPRM | protein tyrosine phosphatase receptor type M [Source:HGNC Symbol;Acc:HGNC:9675] | 15362 | -0.866 | -0.0704 | Yes |
| 140 | CACNA1A | calcium voltage-gated channel subunit alpha1 A [Source:HGNC Symbol;Acc:HGNC:1388] | 15399 | -0.889 | -0.0536 | Yes |
| 141 | FBLN5 | fibulin 5 [Source:HGNC Symbol;Acc:HGNC:3602] | 15478 | -1.001 | -0.0372 | Yes |
| 142 | ADGRL2 | adhesion G protein-coupled receptor L2 [Source:HGNC Symbol;Acc:HGNC:18582] | 15519 | -1.081 | -0.0166 | Yes |
| 143 | MGMT | O-6-methylguanine-DNA methyltransferase [Source:HGNC Symbol;Acc:HGNC:7059] | 15574 | -1.217 | 0.0061 | Yes |
Table: GSEA details [plain text format]

  

Fig 2: HALLMARK\_UV\_RESPONSE\_DN      
 Blue-Pink O' Gram in the Space of the Analyzed GeneSet

  

Fig 3: HALLMARK\_UV\_RESPONSE\_DN: Random ES distribution      
 Gene set null distribution of ES for **HALLMARK\_UV\_RESPONSE\_DN**
