# Supplementary material for: In vivo CRISPR screens reveal Serpinb9 and Adam2 as regulators of immune therapy response in lung cancer
Source: Nat Commun. 2023 May 31;14:3150. doi: 10.1038/s41467-023-38841-7 (PMC10232477; doi:10.1038/s41467-023-38841-7)
Supplement: Supplementary file 13 — Source Data [file 41467_2023_38841_MOESM13_ESM.zip › Figure 4E.zip!/my_analysis.Gsea.1640146340255_Hallmark/gsea_report_for_ADAM2_NI_1640146340255.html]

Report for ADAM2\_NI 1640146340255 [GSEA]

| GS  follow link to MSigDB | GS DETAILS | SIZE | ES | NES | NOM p-val | FDR q-val | FWER p-val | RANK AT MAX | LEADING EDGE || 1 | HALLMARK\_UNFOLDED\_PROTEIN\_RESPONSE | Details ... | 111 | -0.33 | -1.58 | 0.000 | 0.435 | 0.332 | 5315 | tags=50%, list=34%, signal=76% |
| 2 | HALLMARK\_APICAL\_SURFACE | Details ... | 40 | -0.43 | -1.33 | 0.000 | 0.997 | 0.837 | 2062 | tags=35%, list=13%, signal=40% |
| 3 | HALLMARK\_GLYCOLYSIS | Details ... | 194 | -0.27 | -1.22 | 0.112 | 1.000 | 1.000 | 4607 | tags=41%, list=29%, signal=58% |
| 4 | HALLMARK\_MYC\_TARGETS\_V2 | Details ... | 57 | -0.51 | -1.22 | 0.227 | 0.835 | 1.000 | 4679 | tags=56%, list=30%, signal=80% |
| 5 | HALLMARK\_TGF\_BETA\_SIGNALING | Details ... | 54 | -0.31 | -1.16 | 0.299 | 0.851 | 1.000 | 4115 | tags=35%, list=26%, signal=48% |
| 6 | HALLMARK\_ANGIOGENESIS | Details ... | 35 | -0.37 | -1.11 | 0.284 | 0.868 | 1.000 | 1060 | tags=23%, list=7%, signal=24% |
| 7 | HALLMARK\_PEROXISOME | Details ... | 95 | -0.22 | -1.09 | 0.279 | 0.798 | 1.000 | 4693 | tags=34%, list=30%, signal=48% |
| 8 | HALLMARK\_UV\_RESPONSE\_DN | Details ... | 143 | -0.27 | -1.06 | 0.338 | 0.810 | 1.000 | 1743 | tags=19%, list=11%, signal=21% |
| 9 | HALLMARK\_MYC\_TARGETS\_V1 | Details ... | 200 | -0.33 | -0.93 | 0.613 | 1.000 | 1.000 | 5767 | tags=50%, list=37%, signal=78% |
| 10 | HALLMARK\_DNA\_REPAIR | Details ... | 148 | -0.25 | -0.90 | 0.602 | 1.000 | 1.000 | 5534 | tags=43%, list=35%, signal=66% |
| 11 | HALLMARK\_OXIDATIVE\_PHOSPHORYLATION | Details ... | 198 | -0.30 | -0.89 | 0.682 | 0.965 | 1.000 | 6111 | tags=56%, list=39%, signal=90% |
| 12 | HALLMARK\_NOTCH\_SIGNALING | Details ... | 32 | -0.24 | -0.85 | 0.786 | 0.956 | 1.000 | 1425 | tags=16%, list=9%, signal=17% |
| 13 | HALLMARK\_PROTEIN\_SECRETION | Details ... | 96 | -0.22 | -0.85 | 0.538 | 0.886 | 1.000 | 3614 | tags=26%, list=23%, signal=34% |
| 14 | HALLMARK\_P53\_PATHWAY | Details ... | 197 | -0.25 | -0.83 | 0.798 | 0.871 | 1.000 | 1340 | tags=18%, list=9%, signal=20% |
| 15 | HALLMARK\_HEME\_METABOLISM | Details ... | 184 | -0.19 | -0.83 | 0.899 | 0.816 | 1.000 | 3421 | tags=26%, list=22%, signal=32% |
| 16 | HALLMARK\_MTORC1\_SIGNALING | Details ... | 199 | -0.16 | -0.74 | 0.781 | 0.909 | 1.000 | 3431 | tags=24%, list=22%, signal=30% |
| 17 | HALLMARK\_WNT\_BETA\_CATENIN\_SIGNALING | Details ... | 39 | -0.23 | -0.72 | 0.891 | 0.881 | 1.000 | 3230 | tags=26%, list=21%, signal=32% |
Table: Gene sets enriched in phenotype **ADAM2\_NI (3 samples) **[plain text format]****
