# Supplementary material for: In vivo CRISPR screens reveal Serpinb9 and Adam2 as regulators of immune therapy response in lung cancer
Source: Nat Commun. 2023 May 31;14:3150. doi: 10.1038/s41467-023-38841-7 (PMC10232477; doi:10.1038/s41467-023-38841-7)
Supplement: Supplementary file 13 — Source Data [file 41467_2023_38841_MOESM13_ESM.zip › Figure 4E.zip!/my_analysis.Gsea.1640146340255_Hallmark/HALLMARK_INTERFERON_ALPHA_RESPONSE.html]

Details for gene set HALLMARK\_INTERFERON\_ALPHA\_RESPONSE[GSEA]

|  || Dataset | December 21, 2021\_for GSEA run\_collapsed\_to\_symbols.GSEA\_B6\_NI.cls #CTLR\_NI\_versus\_ADAM2\_NI |
| Phenotype | GSEA\_B6\_NI.cls#CTLR\_NI\_versus\_ADAM2\_NI |
| Upregulated in class | CTLR\_NI |
| GeneSet | HALLMARK\_INTERFERON\_ALPHA\_RESPONSE |
| Enrichment Score (ES) | 0.79065406 |
| Normalized Enrichment Score (NES) | 1.5732099 |
| Nominal p-value | 0.0 |
| FDR q-value | 0.19835128 |
| FWER p-Value | 0.336 |
Table: GSEA Results Summary

  

Fig 1: Enrichment plot: HALLMARK\_INTERFERON\_ALPHA\_RESPONSE      
 Profile of the Running ES Score & Positions of GeneSet Members on the Rank Ordered List

  

| SYMBOL | TITLE | RANK IN GENE LIST | RANK METRIC SCORE | RUNNING ES | CORE ENRICHMENT || 1 | CXCL11 | C-X-C motif chemokine ligand 11 [Source:HGNC Symbol;Acc:HGNC:10638] | 2 | 3.216 | 0.0357 | Yes |
| 2 | DDX60 | DExD/H-box helicase 60 [Source:HGNC Symbol;Acc:HGNC:25942] | 21 | 2.435 | 0.0616 | Yes |
| 3 | DHX58 | DExH-box helicase 58 [Source:HGNC Symbol;Acc:HGNC:29517] | 55 | 1.981 | 0.0816 | Yes |
| 4 | PARP9 | poly(ADP-ribose) polymerase family member 9 [Source:HGNC Symbol;Acc:HGNC:24118] | 69 | 1.910 | 0.1020 | Yes |
| 5 | RTP4 | receptor transporter protein 4 [Source:HGNC Symbol;Acc:HGNC:23992] | 80 | 1.848 | 0.1219 | Yes |
| 6 | STAT2 | signal transducer and activator of transcription 2 [Source:HGNC Symbol;Acc:HGNC:11363] | 86 | 1.830 | 0.1420 | Yes |
| 7 | PSMB9 | proteasome 20S subunit beta 9 [Source:HGNC Symbol;Acc:HGNC:9546] | 87 | 1.828 | 0.1623 | Yes |
| 8 | SP110 | SP110 nuclear body protein [Source:HGNC Symbol;Acc:HGNC:5401] | 89 | 1.822 | 0.1825 | Yes |
| 9 | UBE2L6 | ubiquitin conjugating enzyme E2 L6 [Source:HGNC Symbol;Acc:HGNC:12490] | 96 | 1.793 | 0.2021 | Yes |
| 10 | GBP4 | guanylate binding protein 4 [Source:HGNC Symbol;Acc:HGNC:20480] | 111 | 1.765 | 0.2209 | Yes |
| 11 | PARP14 | poly(ADP-ribose) polymerase family member 14 [Source:HGNC Symbol;Acc:HGNC:29232] | 124 | 1.728 | 0.2393 | Yes |
| 12 | IRF9 | interferon regulatory factor 9 [Source:HGNC Symbol;Acc:HGNC:6131] | 128 | 1.701 | 0.2581 | Yes |
| 13 | CXCL10 | C-X-C motif chemokine ligand 10 [Source:HGNC Symbol;Acc:HGNC:10637] | 142 | 1.666 | 0.2758 | Yes |
| 14 | ISG15 | ISG15 ubiquitin like modifier [Source:HGNC Symbol;Acc:HGNC:4053] | 144 | 1.658 | 0.2942 | Yes |
| 15 | HERC6 | HECT and RLD domain containing E3 ubiquitin protein ligase family member 6 [Source:HGNC Symbol;Acc:HGNC:26072] | 155 | 1.628 | 0.3117 | Yes |
| 16 | BATF2 | basic leucine zipper ATF-like transcription factor 2 [Source:HGNC Symbol;Acc:HGNC:25163] | 159 | 1.621 | 0.3295 | Yes |
| 17 | TRIM5 | tripartite motif containing 5 [Source:HGNC Symbol;Acc:HGNC:16276] | 178 | 1.539 | 0.3455 | Yes |
| 18 | IFIH1 | interferon induced with helicase C domain 1 [Source:HGNC Symbol;Acc:HGNC:18873] | 183 | 1.531 | 0.3623 | Yes |
| 19 | SELL | selectin L [Source:HGNC Symbol;Acc:HGNC:10720] | 206 | 1.467 | 0.3772 | Yes |
| 20 | OASL | 2'-5'-oligoadenylate synthetase like [Source:HGNC Symbol;Acc:HGNC:8090] | 214 | 1.455 | 0.3929 | Yes |
| 21 | CMPK2 | cytidine/uridine monophosphate kinase 2 [Source:HGNC Symbol;Acc:HGNC:27015] | 227 | 1.427 | 0.4081 | Yes |
| 22 | IFIT2 | interferon induced protein with tetratricopeptide repeats 2 [Source:HGNC Symbol;Acc:HGNC:5409] | 235 | 1.400 | 0.4232 | Yes |
| 23 | TAP1 | "transporter 1, ATP binding cassette subfamily B member [Source:HGNC Symbol;Acc:HGNC:43]" | 241 | 1.389 | 0.4383 | Yes |
| 24 | CASP1 | caspase 1 [Source:HGNC Symbol;Acc:HGNC:1499] | 248 | 1.383 | 0.4534 | Yes |
| 25 | GBP2 | guanylate binding protein 2 [Source:HGNC Symbol;Acc:HGNC:4183] | 250 | 1.381 | 0.4687 | Yes |
| 26 | LGALS3BP | galectin 3 binding protein [Source:HGNC Symbol;Acc:HGNC:6564] | 263 | 1.359 | 0.4830 | Yes |
| 27 | TRIM14 | tripartite motif containing 14 [Source:HGNC Symbol;Acc:HGNC:16283] | 265 | 1.353 | 0.4980 | Yes |
| 28 | UBA7 | ubiquitin like modifier activating enzyme 7 [Source:HGNC Symbol;Acc:HGNC:12471] | 272 | 1.342 | 0.5126 | Yes |
| 29 | TRIM21 | tripartite motif containing 21 [Source:HGNC Symbol;Acc:HGNC:11312] | 277 | 1.338 | 0.5272 | Yes |
| 30 | IFI44 | interferon induced protein 44 [Source:HGNC Symbol;Acc:HGNC:16938] | 278 | 1.338 | 0.5421 | Yes |
| 31 | USP18 | ubiquitin specific peptidase 18 [Source:HGNC Symbol;Acc:HGNC:12616] | 290 | 1.321 | 0.5561 | Yes |
| 32 | IRF1 | interferon regulatory factor 1 [Source:HGNC Symbol;Acc:HGNC:6116] | 295 | 1.310 | 0.5704 | Yes |
| 33 | BST2 | bone marrow stromal cell antigen 2 [Source:HGNC Symbol;Acc:HGNC:1119] | 306 | 1.293 | 0.5842 | Yes |
| 34 | IFIT3 | interferon induced protein with tetratricopeptide repeats 3 [Source:HGNC Symbol;Acc:HGNC:5411] | 314 | 1.278 | 0.5980 | Yes |
| 35 | PARP12 | poly(ADP-ribose) polymerase family member 12 [Source:HGNC Symbol;Acc:HGNC:21919] | 315 | 1.276 | 0.6122 | Yes |
| 36 | MX1 | MX dynamin like GTPase 1 [Source:HGNC Symbol;Acc:HGNC:7532] | 316 | 1.272 | 0.6263 | Yes |
| 37 | TENT5A | terminal nucleotidyltransferase 5A [Source:HGNC Symbol;Acc:HGNC:18345] | 321 | 1.268 | 0.6402 | Yes |
| 38 | EPSTI1 | epithelial stromal interaction 1 [Source:HGNC Symbol;Acc:HGNC:16465] | 346 | 1.240 | 0.6524 | Yes |
| 39 | RSAD2 | radical S-adenosyl methionine domain containing 2 [Source:HGNC Symbol;Acc:HGNC:30908] | 354 | 1.225 | 0.6656 | Yes |
| 40 | IRF7 | interferon regulatory factor 7 [Source:HGNC Symbol;Acc:HGNC:6122] | 359 | 1.219 | 0.6789 | Yes |
| 41 | SAMD9L | sterile alpha motif domain containing 9 like [Source:HGNC Symbol;Acc:HGNC:1349] | 385 | 1.195 | 0.6906 | Yes |
| 42 | CCRL2 | C-C motif chemokine receptor like 2 [Source:HGNC Symbol;Acc:HGNC:1612] | 427 | 1.145 | 0.7007 | Yes |
| 43 | B2M | beta-2-microglobulin [Source:HGNC Symbol;Acc:HGNC:914] | 441 | 1.131 | 0.7125 | Yes |
| 44 | PSMB8 | proteasome 20S subunit beta 8 [Source:HGNC Symbol;Acc:HGNC:9545] | 467 | 1.101 | 0.7231 | Yes |
| 45 | EIF2AK2 | eukaryotic translation initiation factor 2 alpha kinase 2 [Source:HGNC Symbol;Acc:HGNC:9437] | 472 | 1.098 | 0.7351 | Yes |
| 46 | PSME2 | proteasome activator subunit 2 [Source:HGNC Symbol;Acc:HGNC:9569] | 507 | 1.064 | 0.7448 | Yes |
| 47 | OAS1 | 2'-5'-oligoadenylate synthetase 1 [Source:HGNC Symbol;Acc:HGNC:8086] | 523 | 1.041 | 0.7554 | Yes |
| 48 | PSME1 | proteasome activator subunit 1 [Source:HGNC Symbol;Acc:HGNC:9568] | 569 | 1.013 | 0.7638 | Yes |
| 49 | NMI | N-myc and STAT interactor [Source:HGNC Symbol;Acc:HGNC:7854] | 579 | 1.008 | 0.7744 | Yes |
| 50 | TRAFD1 | TRAF-type zinc finger domain containing 1 [Source:HGNC Symbol;Acc:HGNC:24808] | 598 | 0.998 | 0.7844 | Yes |
| 51 | RNF31 | ring finger protein 31 [Source:HGNC Symbol;Acc:HGNC:16031] | 772 | 0.891 | 0.7832 | Yes |
| 52 | IFI35 | interferon induced protein 35 [Source:HGNC Symbol;Acc:HGNC:5399] | 998 | 0.820 | 0.7779 | Yes |
| 53 | TMEM140 | transmembrane protein 140 [Source:HGNC Symbol;Acc:HGNC:21870] | 1030 | 0.799 | 0.7848 | Yes |
| 54 | LPAR6 | lysophosphatidic acid receptor 6 [Source:HGNC Symbol;Acc:HGNC:15520] | 1075 | 0.782 | 0.7907 | Yes |
| 55 | IFI27 | interferon alpha inducible protein 27 [Source:HGNC Symbol;Acc:HGNC:5397] | 1395 | 0.662 | 0.7775 | No |
| 56 | HELZ2 | helicase with zinc finger 2 [Source:HGNC Symbol;Acc:HGNC:30021] | 1403 | 0.659 | 0.7844 | No |
| 57 | CD74 | CD74 molecule [Source:HGNC Symbol;Acc:HGNC:1697] | 1682 | 0.573 | 0.7730 | No |
| 58 | TRIM25 | tripartite motif containing 25 [Source:HGNC Symbol;Acc:HGNC:12932] | 1725 | 0.560 | 0.7765 | No |
| 59 | IFITM3 | interferon induced transmembrane protein 3 [Source:HGNC Symbol;Acc:HGNC:5414] | 1928 | 0.517 | 0.7693 | No |
| 60 | ELF1 | E74 like ETS transcription factor 1 [Source:HGNC Symbol;Acc:HGNC:3316] | 2249 | 0.476 | 0.7540 | No |
| 61 | CMTR1 | cap methyltransferase 1 [Source:HGNC Symbol;Acc:HGNC:21077] | 2326 | 0.465 | 0.7543 | No |
| 62 | ISG20 | interferon stimulated exonuclease gene 20 [Source:HGNC Symbol;Acc:HGNC:6130] | 2346 | 0.460 | 0.7582 | No |
| 63 | ADAR | adenosine deaminase RNA specific [Source:HGNC Symbol;Acc:HGNC:225] | 2405 | 0.451 | 0.7595 | No |
| 64 | CASP8 | caspase 8 [Source:HGNC Symbol;Acc:HGNC:1509] | 2434 | 0.446 | 0.7627 | No |
| 65 | CD47 | CD47 molecule [Source:HGNC Symbol;Acc:HGNC:1682] | 2439 | 0.445 | 0.7674 | No |
| 66 | IFITM1 | interferon induced transmembrane protein 1 [Source:HGNC Symbol;Acc:HGNC:5412] | 2571 | 0.440 | 0.7639 | No |
| 67 | IL15 | interleukin 15 [Source:HGNC Symbol;Acc:HGNC:5977] | 2791 | 0.418 | 0.7545 | No |
| 68 | IRF2 | interferon regulatory factor 2 [Source:HGNC Symbol;Acc:HGNC:6117] | 3080 | 0.391 | 0.7403 | No |
| 69 | IL4R | interleukin 4 receptor [Source:HGNC Symbol;Acc:HGNC:6015] | 3233 | 0.368 | 0.7347 | No |
| 70 | PSMA3 | proteasome 20S subunit alpha 3 [Source:HGNC Symbol;Acc:HGNC:9532] | 3335 | 0.352 | 0.7321 | No |
| 71 | IFI30 | IFI30 lysosomal thiol reductase [Source:HGNC Symbol;Acc:HGNC:5398] | 3485 | 0.330 | 0.7262 | No |
| 72 | C1S | complement C1s [Source:HGNC Symbol;Acc:HGNC:1247] | 3521 | 0.324 | 0.7276 | No |
| 73 | NUB1 | negative regulator of ubiquitin like proteins 1 [Source:HGNC Symbol;Acc:HGNC:17623] | 3887 | 0.280 | 0.7072 | No |
| 74 | IL7 | interleukin 7 [Source:HGNC Symbol;Acc:HGNC:6023] | 4006 | 0.265 | 0.7026 | No |
| 75 | PNPT1 | polyribonucleotide nucleotidyltransferase 1 [Source:HGNC Symbol;Acc:HGNC:23166] | 4303 | 0.232 | 0.6862 | No |
| 76 | TDRD7 | tudor domain containing 7 [Source:HGNC Symbol;Acc:HGNC:30831] | 4400 | 0.222 | 0.6825 | No |
| 77 | TRIM26 | tripartite motif containing 26 [Source:HGNC Symbol;Acc:HGNC:12962] | 4650 | 0.201 | 0.6687 | No |
| 78 | MOV10 | Mov10 RISC complex RNA helicase [Source:HGNC Symbol;Acc:HGNC:7200] | 4724 | 0.194 | 0.6662 | No |
| 79 | NCOA7 | nuclear receptor coactivator 7 [Source:HGNC Symbol;Acc:HGNC:21081] | 5360 | 0.138 | 0.6270 | No |
| 80 | RIPK2 | receptor interacting serine/threonine kinase 2 [Source:HGNC Symbol;Acc:HGNC:10020] | 5692 | 0.113 | 0.6070 | No |
| 81 | CNP | "2',3'-cyclic nucleotide 3' phosphodiesterase [Source:HGNC Symbol;Acc:HGNC:2158]" | 5754 | 0.109 | 0.6043 | No |
| 82 | TXNIP | thioredoxin interacting protein [Source:HGNC Symbol;Acc:HGNC:16952] | 5781 | 0.106 | 0.6038 | No |
| 83 | SLC25A28 | solute carrier family 25 member 28 [Source:HGNC Symbol;Acc:HGNC:23472] | 6127 | 0.081 | 0.5826 | No |
| 84 | WARS1 | tryptophanyl-tRNA synthetase 1 [Source:HGNC Symbol;Acc:HGNC:12729] | 6325 | 0.068 | 0.5707 | No |
| 85 | PLSCR1 | phospholipid scramblase 1 [Source:HGNC Symbol;Acc:HGNC:9092] | 7180 | 0.013 | 0.5160 | No |
| 86 | LY6E | lymphocyte antigen 6 family member E [Source:HGNC Symbol;Acc:HGNC:6727] | 7188 | 0.013 | 0.5157 | No |
| 87 | OGFR | opioid growth factor receptor [Source:HGNC Symbol;Acc:HGNC:15768] | 7450 | -0.002 | 0.4989 | No |
| 88 | LAP3 | leucine aminopeptidase 3 [Source:HGNC Symbol;Acc:HGNC:18449] | 8033 | -0.031 | 0.4619 | No |
| 89 | MVB12A | multivesicular body subunit 12A [Source:HGNC Symbol;Acc:HGNC:25153] | 8859 | -0.076 | 0.4098 | No |
| 90 | IFITM2 | interferon induced transmembrane protein 2 [Source:HGNC Symbol;Acc:HGNC:5413] | 10288 | -0.159 | 0.3199 | No |
| 91 | PROCR | protein C receptor [Source:HGNC Symbol;Acc:HGNC:9452] | 12467 | -0.293 | 0.1833 | No |
| 92 | LAMP3 | lysosomal associated membrane protein 3 [Source:HGNC Symbol;Acc:HGNC:14582] | 14483 | -0.511 | 0.0596 | No |
| 93 | CSF1 | colony stimulating factor 1 [Source:HGNC Symbol;Acc:HGNC:2432] | 14746 | -0.566 | 0.0491 | No |
| 94 | GMPR | guanosine monophosphate reductase [Source:HGNC Symbol;Acc:HGNC:4376] | 15406 | -0.902 | 0.0168 | No |
Table: GSEA details [plain text format]

  

Fig 2: HALLMARK\_INTERFERON\_ALPHA\_RESPONSE      
 Blue-Pink O' Gram in the Space of the Analyzed GeneSet

  

Fig 3: HALLMARK\_INTERFERON\_ALPHA\_RESPONSE: Random ES distribution      
 Gene set null distribution of ES for **HALLMARK\_INTERFERON\_ALPHA\_RESPONSE**
