# Supplementary material for: In vivo CRISPR screens reveal Serpinb9 and Adam2 as regulators of immune therapy response in lung cancer
Source: Nat Commun. 2023 May 31;14:3150. doi: 10.1038/s41467-023-38841-7 (PMC10232477; doi:10.1038/s41467-023-38841-7)
Supplement: Supplementary file 13 — Source Data [file 41467_2023_38841_MOESM13_ESM.zip › Figure 4E.zip!/my_analysis.Gsea.1640146340255_Hallmark/HALLMARK_DNA_REPAIR.html]

Details for gene set HALLMARK\_DNA\_REPAIR[GSEA]

|  || Dataset | December 21, 2021\_for GSEA run\_collapsed\_to\_symbols.GSEA\_B6\_NI.cls #CTLR\_NI\_versus\_ADAM2\_NI |
| Phenotype | GSEA\_B6\_NI.cls#CTLR\_NI\_versus\_ADAM2\_NI |
| Upregulated in class | ADAM2\_NI |
| GeneSet | HALLMARK\_DNA\_REPAIR |
| Enrichment Score (ES) | -0.24739712 |
| Normalized Enrichment Score (NES) | -0.8984459 |
| Nominal p-value | 0.60157794 |
| FDR q-value | 1.0 |
| FWER p-Value | 1.0 |
Table: GSEA Results Summary

  

Fig 1: Enrichment plot: HALLMARK\_DNA\_REPAIR      
 Profile of the Running ES Score & Positions of GeneSet Members on the Rank Ordered List

  

| SYMBOL | TITLE | RANK IN GENE LIST | RANK METRIC SCORE | RUNNING ES | CORE ENRICHMENT || 1 | CMPK2 | cytidine/uridine monophosphate kinase 2 [Source:HGNC Symbol;Acc:HGNC:27015] | 227 | 1.427 | 0.0312 | No |
| 2 | COX17 | cytochrome c oxidase copper chaperone COX17 [Source:HGNC Symbol;Acc:HGNC:2264] | 723 | 0.920 | 0.0289 | No |
| 3 | PNP | purine nucleoside phosphorylase [Source:HGNC Symbol;Acc:HGNC:7892] | 902 | 0.841 | 0.0445 | No |
| 4 | TAF9 | TATA-box binding protein associated factor 9 [Source:HGNC Symbol;Acc:HGNC:11542] | 1116 | 0.765 | 0.0553 | No |
| 5 | HCLS1 | hematopoietic cell-specific Lyn substrate 1 [Source:HGNC Symbol;Acc:HGNC:4844] | 1484 | 0.633 | 0.0520 | No |
| 6 | ARL6IP1 | ADP ribosylation factor like GTPase 6 interacting protein 1 [Source:HGNC Symbol;Acc:HGNC:697] | 1722 | 0.562 | 0.0548 | No |
| 7 | POLR2K | "RNA polymerase II, I and III subunit K [Source:HGNC Symbol;Acc:HGNC:9198]" | 2233 | 0.479 | 0.0373 | No |
| 8 | RFC4 | replication factor C subunit 4 [Source:HGNC Symbol;Acc:HGNC:9972] | 3006 | 0.403 | 0.0005 | No |
| 9 | RNMT | RNA guanine-7 methyltransferase [Source:HGNC Symbol;Acc:HGNC:10075] | 3436 | 0.337 | -0.0163 | No |
| 10 | CCNO | cyclin O [Source:HGNC Symbol;Acc:HGNC:18576] | 3620 | 0.312 | -0.0180 | No |
| 11 | SDCBP | syndecan binding protein [Source:HGNC Symbol;Acc:HGNC:10662] | 3750 | 0.296 | -0.0169 | No |
| 12 | DDB2 | damage specific DNA binding protein 2 [Source:HGNC Symbol;Acc:HGNC:2718] | 4125 | 0.251 | -0.0329 | No |
| 13 | PRIM1 | DNA primase subunit 1 [Source:HGNC Symbol;Acc:HGNC:9369] | 4433 | 0.219 | -0.0456 | No |
| 14 | GMPR2 | guanosine monophosphate reductase 2 [Source:HGNC Symbol;Acc:HGNC:4377] | 4580 | 0.206 | -0.0484 | No |
| 15 | TYMS | thymidylate synthetase [Source:HGNC Symbol;Acc:HGNC:12441] | 4624 | 0.203 | -0.0447 | No |
| 16 | FEN1 | flap structure-specific endonuclease 1 [Source:HGNC Symbol;Acc:HGNC:3650] | 5037 | 0.164 | -0.0659 | No |
| 17 | DCTN4 | dynactin subunit 4 [Source:HGNC Symbol;Acc:HGNC:15518] | 5121 | 0.156 | -0.0663 | No |
| 18 | REV3L | "REV3 like, DNA directed polymerase zeta catalytic subunit [Source:HGNC Symbol;Acc:HGNC:9968]" | 5192 | 0.151 | -0.0659 | No |
| 19 | RAD51 | RAD51 recombinase [Source:HGNC Symbol;Acc:HGNC:9817] | 5198 | 0.151 | -0.0614 | No |
| 20 | ERCC3 | "ERCC excision repair 3, TFIIH core complex helicase subunit [Source:HGNC Symbol;Acc:HGNC:3435]" | 5472 | 0.129 | -0.0748 | No |
| 21 | MRPL40 | mitochondrial ribosomal protein L40 [Source:HGNC Symbol;Acc:HGNC:14491] | 5483 | 0.128 | -0.0713 | No |
| 22 | PDE4B | phosphodiesterase 4B [Source:HGNC Symbol;Acc:HGNC:8781] | 5633 | 0.116 | -0.0772 | No |
| 23 | GTF2A2 | general transcription factor IIA subunit 2 [Source:HGNC Symbol;Acc:HGNC:4647] | 6049 | 0.087 | -0.1011 | No |
| 24 | CETN2 | centrin 2 [Source:HGNC Symbol;Acc:HGNC:1867] | 6131 | 0.081 | -0.1037 | No |
| 25 | PCNA | proliferating cell nuclear antigen [Source:HGNC Symbol;Acc:HGNC:8729] | 6214 | 0.075 | -0.1066 | No |
| 26 | CSTF3 | cleavage stimulation factor subunit 3 [Source:HGNC Symbol;Acc:HGNC:2485] | 6232 | 0.075 | -0.1053 | No |
| 27 | POLR2A | RNA polymerase II subunit A [Source:HGNC Symbol;Acc:HGNC:9187] | 6252 | 0.073 | -0.1042 | No |
| 28 | DGCR8 | DGCR8 microprocessor complex subunit [Source:HGNC Symbol;Acc:HGNC:2847] | 6281 | 0.071 | -0.1037 | No |
| 29 | GSDME | gasdermin E [Source:HGNC Symbol;Acc:HGNC:2810] | 6331 | 0.068 | -0.1047 | No |
| 30 | RALA | RAS like proto-oncogene A [Source:HGNC Symbol;Acc:HGNC:9839] | 6360 | 0.066 | -0.1044 | No |
| 31 | TARBP2 | TARBP2 subunit of RISC loading complex [Source:HGNC Symbol;Acc:HGNC:11569] | 6425 | 0.061 | -0.1065 | No |
| 32 | RFC3 | replication factor C subunit 3 [Source:HGNC Symbol;Acc:HGNC:9971] | 6497 | 0.055 | -0.1093 | No |
| 33 | POLA2 | "DNA polymerase alpha 2, accessory subunit [Source:HGNC Symbol;Acc:HGNC:30073]" | 6760 | 0.039 | -0.1250 | No |
| 34 | ERCC4 | "ERCC excision repair 4, endonuclease catalytic subunit [Source:HGNC Symbol;Acc:HGNC:3436]" | 6786 | 0.037 | -0.1254 | No |
| 35 | GTF2H3 | general transcription factor IIH subunit 3 [Source:HGNC Symbol;Acc:HGNC:4657] | 6798 | 0.036 | -0.1249 | No |
| 36 | RFC5 | replication factor C subunit 5 [Source:HGNC Symbol;Acc:HGNC:9973] | 6921 | 0.028 | -0.1319 | No |
| 37 | POLD4 | "DNA polymerase delta 4, accessory subunit [Source:HGNC Symbol;Acc:HGNC:14106]" | 6932 | 0.027 | -0.1317 | No |
| 38 | SRSF6 | serine and arginine rich splicing factor 6 [Source:HGNC Symbol;Acc:HGNC:10788] | 7074 | 0.020 | -0.1401 | No |
| 39 | SF3A3 | splicing factor 3a subunit 3 [Source:HGNC Symbol;Acc:HGNC:10767] | 7184 | 0.013 | -0.1467 | No |
| 40 | NCBP2 | nuclear cap binding protein subunit 2 [Source:HGNC Symbol;Acc:HGNC:7659] | 7268 | 0.009 | -0.1518 | No |
| 41 | AAAS | aladin WD repeat nucleoporin [Source:HGNC Symbol;Acc:HGNC:13666] | 7273 | 0.008 | -0.1517 | No |
| 42 | POLA1 | "DNA polymerase alpha 1, catalytic subunit [Source:HGNC Symbol;Acc:HGNC:9173]" | 7391 | 0.001 | -0.1592 | No |
| 43 | SMAD5 | SMAD family member 5 [Source:HGNC Symbol;Acc:HGNC:6771] | 7411 | 0.000 | -0.1605 | No |
| 44 | POLH | DNA polymerase eta [Source:HGNC Symbol;Acc:HGNC:9181] | 7443 | -0.001 | -0.1624 | No |
| 45 | POLD3 | "DNA polymerase delta 3, accessory subunit [Source:HGNC Symbol;Acc:HGNC:20932]" | 7459 | -0.002 | -0.1633 | No |
| 46 | CLP1 | cleavage factor polyribonucleotide kinase subunit 1 [Source:HGNC Symbol;Acc:HGNC:16999] | 7493 | -0.004 | -0.1653 | No |
| 47 | POLR2H | "RNA polymerase II, I and III subunit H [Source:HGNC Symbol;Acc:HGNC:9195]" | 7644 | -0.010 | -0.1746 | No |
| 48 | POLR1H | RNA polymerase I subunit H [Source:HGNC Symbol;Acc:HGNC:13182] | 7651 | -0.011 | -0.1747 | No |
| 49 | POLR2I | RNA polymerase II subunit I [Source:HGNC Symbol;Acc:HGNC:9196] | 7653 | -0.011 | -0.1744 | No |
| 50 | TK2 | thymidine kinase 2 [Source:HGNC Symbol;Acc:HGNC:11831] | 7658 | -0.011 | -0.1743 | No |
| 51 | DAD1 | defender against cell death 1 [Source:HGNC Symbol;Acc:HGNC:2664] | 7679 | -0.012 | -0.1752 | No |
| 52 | RRM2B | ribonucleotide reductase regulatory TP53 inducible subunit M2B [Source:HGNC Symbol;Acc:HGNC:17296] | 7837 | -0.021 | -0.1846 | No |
| 53 | SNAPC4 | small nuclear RNA activating complex polypeptide 4 [Source:HGNC Symbol;Acc:HGNC:11137] | 7843 | -0.021 | -0.1843 | No |
| 54 | NUDT9 | nudix hydrolase 9 [Source:HGNC Symbol;Acc:HGNC:8056] | 7845 | -0.021 | -0.1837 | No |
| 55 | RAD52 | "RAD52 homolog, DNA repair protein [Source:HGNC Symbol;Acc:HGNC:9824]" | 8034 | -0.031 | -0.1948 | No |
| 56 | ERCC2 | "ERCC excision repair 2, TFIIH core complex helicase subunit [Source:HGNC Symbol;Acc:HGNC:3434]" | 8048 | -0.031 | -0.1946 | No |
| 57 | ZWINT | ZW10 interacting kinetochore protein [Source:HGNC Symbol;Acc:HGNC:13195] | 8110 | -0.036 | -0.1974 | No |
| 58 | POLR2D | RNA polymerase II subunit D [Source:HGNC Symbol;Acc:HGNC:9191] | 8183 | -0.039 | -0.2008 | No |
| 59 | STX3 | syntaxin 3 [Source:HGNC Symbol;Acc:HGNC:11438] | 8243 | -0.042 | -0.2032 | No |
| 60 | MPC2 | mitochondrial pyruvate carrier 2 [Source:HGNC Symbol;Acc:HGNC:24515] | 8454 | -0.053 | -0.2151 | No |
| 61 | POLD1 | "DNA polymerase delta 1, catalytic subunit [Source:HGNC Symbol;Acc:HGNC:9175]" | 8640 | -0.063 | -0.2250 | No |
| 62 | NUDT21 | nudix hydrolase 21 [Source:HGNC Symbol;Acc:HGNC:13870] | 8669 | -0.065 | -0.2247 | No |
| 63 | TMED2 | transmembrane p24 trafficking protein 2 [Source:HGNC Symbol;Acc:HGNC:16996] | 8723 | -0.068 | -0.2259 | No |
| 64 | GTF2H1 | general transcription factor IIH subunit 1 [Source:HGNC Symbol;Acc:HGNC:4655] | 8752 | -0.069 | -0.2255 | No |
| 65 | DUT | deoxyuridine triphosphatase [Source:HGNC Symbol;Acc:HGNC:3078] | 8767 | -0.070 | -0.2241 | No |
| 66 | SURF1 | SURF1 cytochrome c oxidase assembly factor [Source:HGNC Symbol;Acc:HGNC:11474] | 8825 | -0.074 | -0.2254 | No |
| 67 | ELL | elongation factor for RNA polymerase II [Source:HGNC Symbol;Acc:HGNC:23114] | 8861 | -0.076 | -0.2252 | No |
| 68 | VPS37B | VPS37B subunit of ESCRT-I [Source:HGNC Symbol;Acc:HGNC:25754] | 8980 | -0.081 | -0.2302 | No |
| 69 | MPG | N-methylpurine DNA glycosylase [Source:HGNC Symbol;Acc:HGNC:7211] | 8994 | -0.082 | -0.2284 | No |
| 70 | UMPS | uridine monophosphate synthetase [Source:HGNC Symbol;Acc:HGNC:12563] | 9005 | -0.083 | -0.2264 | No |
| 71 | ITPA | inosine triphosphatase [Source:HGNC Symbol;Acc:HGNC:6176] | 9076 | -0.087 | -0.2281 | No |
| 72 | RPA2 | replication protein A2 [Source:HGNC Symbol;Acc:HGNC:10290] | 9154 | -0.091 | -0.2302 | No |
| 73 | ERCC8 | "ERCC excision repair 8, CSA ubiquitin ligase complex subunit [Source:HGNC Symbol;Acc:HGNC:3439]" | 9197 | -0.093 | -0.2299 | No |
| 74 | RFC2 | replication factor C subunit 2 [Source:HGNC Symbol;Acc:HGNC:9970] | 9249 | -0.095 | -0.2301 | No |
| 75 | NME1 | NME/NM23 nucleoside diphosphate kinase 1 [Source:HGNC Symbol;Acc:HGNC:7849] | 9314 | -0.099 | -0.2311 | No |
| 76 | GTF2F1 | general transcription factor IIF subunit 1 [Source:HGNC Symbol;Acc:HGNC:4652] | 9442 | -0.107 | -0.2358 | No |
| 77 | LIG1 | DNA ligase 1 [Source:HGNC Symbol;Acc:HGNC:6598] | 9533 | -0.113 | -0.2380 | No |
| 78 | HPRT1 | hypoxanthine phosphoribosyltransferase 1 [Source:HGNC Symbol;Acc:HGNC:5157] | 9568 | -0.116 | -0.2364 | No |
| 79 | AK3 | adenylate kinase 3 [Source:HGNC Symbol;Acc:HGNC:17376] | 9663 | -0.122 | -0.2386 | No |
| 80 | NT5C3A | "5'-nucleotidase, cytosolic IIIA [Source:HGNC Symbol;Acc:HGNC:17820]" | 9736 | -0.127 | -0.2391 | No |
| 81 | POM121 | POM121 transmembrane nucleoporin [Source:HGNC Symbol;Acc:HGNC:19702] | 9790 | -0.130 | -0.2384 | No |
| 82 | RBX1 | ring-box 1 [Source:HGNC Symbol;Acc:HGNC:9928] | 9819 | -0.132 | -0.2359 | No |
| 83 | NFX1 | "nuclear transcription factor, X-box binding 1 [Source:HGNC Symbol;Acc:HGNC:7803]" | 9847 | -0.133 | -0.2334 | No |
| 84 | RAE1 | ribonucleic acid export 1 [Source:HGNC Symbol;Acc:HGNC:9828] | 9966 | -0.140 | -0.2365 | No |
| 85 | NELFCD | negative elongation factor complex member C/D [Source:HGNC Symbol;Acc:HGNC:15934] | 10136 | -0.150 | -0.2426 | Yes |
| 86 | NELFB | negative elongation factor complex member B [Source:HGNC Symbol;Acc:HGNC:24324] | 10138 | -0.150 | -0.2378 | Yes |
| 87 | POLR2G | RNA polymerase II subunit G [Source:HGNC Symbol;Acc:HGNC:9194] | 10213 | -0.155 | -0.2376 | Yes |
| 88 | SNAPC5 | small nuclear RNA activating complex polypeptide 5 [Source:HGNC Symbol;Acc:HGNC:15484] | 10348 | -0.163 | -0.2410 | Yes |
| 89 | ALYREF | Aly/REF export factor [Source:HGNC Symbol;Acc:HGNC:19071] | 10377 | -0.164 | -0.2376 | Yes |
| 90 | TSG101 | tumor susceptibility 101 [Source:HGNC Symbol;Acc:HGNC:15971] | 10431 | -0.167 | -0.2356 | Yes |
| 91 | TAF13 | TATA-box binding protein associated factor 13 [Source:HGNC Symbol;Acc:HGNC:11546] | 10476 | -0.170 | -0.2330 | Yes |
| 92 | POLR3GL | RNA polymerase III subunit GL [Source:HGNC Symbol;Acc:HGNC:28466] | 10498 | -0.172 | -0.2288 | Yes |
| 93 | ADA | adenosine deaminase [Source:HGNC Symbol;Acc:HGNC:186] | 10516 | -0.173 | -0.2244 | Yes |
| 94 | SUPT4H1 | "SPT4 homolog, DSIF elongation factor subunit [Source:HGNC Symbol;Acc:HGNC:11467]" | 10618 | -0.178 | -0.2251 | Yes |
| 95 | RPA3 | replication protein A3 [Source:HGNC Symbol;Acc:HGNC:10291] | 10652 | -0.180 | -0.2215 | Yes |
| 96 | POLR2F | "RNA polymerase II, I and III subunit F [Source:HGNC Symbol;Acc:HGNC:9193]" | 10655 | -0.180 | -0.2158 | Yes |
| 97 | ERCC5 | "ERCC excision repair 5, endonuclease [Source:HGNC Symbol;Acc:HGNC:3437]" | 10670 | -0.181 | -0.2109 | Yes |
| 98 | AGO4 | argonaute RISC component 4 [Source:HGNC Symbol;Acc:HGNC:18424] | 10928 | -0.196 | -0.2212 | Yes |
| 99 | TAF12 | TATA-box binding protein associated factor 12 [Source:HGNC Symbol;Acc:HGNC:11545] | 10936 | -0.197 | -0.2153 | Yes |
| 100 | POLL | DNA polymerase lambda [Source:HGNC Symbol;Acc:HGNC:9184] | 10981 | -0.199 | -0.2117 | Yes |
| 101 | VPS28 | VPS28 subunit of ESCRT-I [Source:HGNC Symbol;Acc:HGNC:18178] | 11060 | -0.203 | -0.2102 | Yes |
| 102 | POLR3C | RNA polymerase III subunit C [Source:HGNC Symbol;Acc:HGNC:30076] | 11062 | -0.204 | -0.2037 | Yes |
| 103 | SUPT5H | "SPT5 homolog, DSIF elongation factor subunit [Source:HGNC Symbol;Acc:HGNC:11469]" | 11113 | -0.207 | -0.2003 | Yes |
| 104 | POLE4 | "DNA polymerase epsilon 4, accessory subunit [Source:HGNC Symbol;Acc:HGNC:18755]" | 11146 | -0.209 | -0.1957 | Yes |
| 105 | ADRM1 | adhesion regulating molecule 1 [Source:HGNC Symbol;Acc:HGNC:15759] | 11166 | -0.210 | -0.1901 | Yes |
| 106 | TP53 | tumor protein p53 [Source:HGNC Symbol;Acc:HGNC:11998] | 11287 | -0.216 | -0.1909 | Yes |
| 107 | SSRP1 | structure specific recognition protein 1 [Source:HGNC Symbol;Acc:HGNC:11327] | 11382 | -0.223 | -0.1898 | Yes |
| 108 | POLR2C | RNA polymerase II subunit C [Source:HGNC Symbol;Acc:HGNC:9189] | 11397 | -0.224 | -0.1835 | Yes |
| 109 | GPX4 | glutathione peroxidase 4 [Source:HGNC Symbol;Acc:HGNC:4556] | 11537 | -0.231 | -0.1851 | Yes |
| 110 | GTF2B | general transcription factor IIB [Source:HGNC Symbol;Acc:HGNC:4648] | 11609 | -0.236 | -0.1821 | Yes |
| 111 | VPS37D | VPS37D subunit of ESCRT-I [Source:HGNC Symbol;Acc:HGNC:18287] | 11639 | -0.237 | -0.1763 | Yes |
| 112 | POLR1D | RNA polymerase I and III subunit D [Source:HGNC Symbol;Acc:HGNC:20422] | 11641 | -0.237 | -0.1687 | Yes |
| 113 | ZNF707 | zinc finger protein 707 [Source:HGNC Symbol;Acc:HGNC:27815] | 11644 | -0.237 | -0.1612 | Yes |
| 114 | BRF2 | BRF2 RNA polymerase III transcription initiation factor subunit [Source:HGNC Symbol;Acc:HGNC:17298] | 11939 | -0.256 | -0.1720 | Yes |
| 115 | DDB1 | damage specific DNA binding protein 1 [Source:HGNC Symbol;Acc:HGNC:2717] | 11953 | -0.257 | -0.1645 | Yes |
| 116 | TAF6 | TATA-box binding protein associated factor 6 [Source:HGNC Symbol;Acc:HGNC:11540] | 12092 | -0.267 | -0.1648 | Yes |
| 117 | ERCC1 | "ERCC excision repair 1, endonuclease non-catalytic subunit [Source:HGNC Symbol;Acc:HGNC:3433]" | 12105 | -0.268 | -0.1570 | Yes |
| 118 | DGUOK | deoxyguanosine kinase [Source:HGNC Symbol;Acc:HGNC:2858] | 12121 | -0.268 | -0.1494 | Yes |
| 119 | CANT1 | calcium activated nucleotidase 1 [Source:HGNC Symbol;Acc:HGNC:19721] | 12195 | -0.273 | -0.1453 | Yes |
| 120 | GUK1 | guanylate kinase 1 [Source:HGNC Symbol;Acc:HGNC:4693] | 12241 | -0.277 | -0.1393 | Yes |
| 121 | ADCY6 | adenylate cyclase 6 [Source:HGNC Symbol;Acc:HGNC:237] | 12244 | -0.277 | -0.1305 | Yes |
| 122 | USP11 | ubiquitin specific peptidase 11 [Source:HGNC Symbol;Acc:HGNC:12609] | 12400 | -0.289 | -0.1312 | Yes |
| 123 | GTF3C5 | general transcription factor IIIC subunit 5 [Source:HGNC Symbol;Acc:HGNC:4668] | 12557 | -0.301 | -0.1316 | Yes |
| 124 | XPC | "XPC complex subunit, DNA damage recognition and repair factor [Source:HGNC Symbol;Acc:HGNC:12816]" | 12574 | -0.302 | -0.1229 | Yes |
| 125 | ELOA | elongin A [Source:HGNC Symbol;Acc:HGNC:11620] | 12627 | -0.306 | -0.1165 | Yes |
| 126 | POLR2J | RNA polymerase II subunit J [Source:HGNC Symbol;Acc:HGNC:9197] | 12790 | -0.319 | -0.1167 | Yes |
| 127 | UPF3B | UPF3B regulator of nonsense mediated mRNA decay [Source:HGNC Symbol;Acc:HGNC:20439] | 12811 | -0.320 | -0.1077 | Yes |
| 128 | PDE6G | phosphodiesterase 6G [Source:HGNC Symbol;Acc:HGNC:8789] | 12813 | -0.320 | -0.0974 | Yes |
| 129 | EIF1B | eukaryotic translation initiation factor 1B [Source:HGNC Symbol;Acc:HGNC:30792] | 12865 | -0.325 | -0.0903 | Yes |
| 130 | APRT | adenine phosphoribosyltransferase [Source:HGNC Symbol;Acc:HGNC:626] | 13134 | -0.350 | -0.0963 | Yes |
| 131 | TAF1C | "TATA-box binding protein associated factor, RNA polymerase I subunit C [Source:HGNC Symbol;Acc:HGNC:11534]" | 13165 | -0.353 | -0.0869 | Yes |
| 132 | BCAP31 | B cell receptor associated protein 31 [Source:HGNC Symbol;Acc:HGNC:16695] | 13210 | -0.357 | -0.0783 | Yes |
| 133 | SEC61A1 | SEC61 translocon subunit alpha 1 [Source:HGNC Symbol;Acc:HGNC:18276] | 13211 | -0.357 | -0.0668 | Yes |
| 134 | POLB | DNA polymerase beta [Source:HGNC Symbol;Acc:HGNC:9174] | 13384 | -0.378 | -0.0658 | Yes |
| 135 | BCAM | basal cell adhesion molecule (Lutheran blood group) [Source:HGNC Symbol;Acc:HGNC:6722] | 13487 | -0.391 | -0.0598 | Yes |
| 136 | GTF2H5 | general transcription factor IIH subunit 5 [Source:HGNC Symbol;Acc:HGNC:21157] | 13493 | -0.392 | -0.0475 | Yes |
| 137 | POLR1C | RNA polymerase I and III subunit C [Source:HGNC Symbol;Acc:HGNC:20194] | 13527 | -0.395 | -0.0369 | Yes |
| 138 | SAC3D1 | SAC3 domain containing 1 [Source:HGNC Symbol;Acc:HGNC:30179] | 13599 | -0.405 | -0.0285 | Yes |
| 139 | TAF10 | TATA-box binding protein associated factor 10 [Source:HGNC Symbol;Acc:HGNC:11543] | 13676 | -0.414 | -0.0201 | Yes |
| 140 | NELFE | negative elongation factor complex member E [Source:HGNC Symbol;Acc:HGNC:13974] | 13681 | -0.415 | -0.0070 | Yes |
| 141 | NT5C | "5', 3'-nucleotidase, cytosolic [Source:HGNC Symbol;Acc:HGNC:17144]" | 13822 | -0.421 | -0.0025 | Yes |
| 142 | POLR2E | "RNA polymerase II, I and III subunit E [Source:HGNC Symbol;Acc:HGNC:9192]" | 14024 | -0.436 | -0.0015 | Yes |
| 143 | EDF1 | endothelial differentiation related factor 1 [Source:HGNC Symbol;Acc:HGNC:3164] | 14033 | -0.437 | 0.0121 | Yes |
| 144 | CDA | cytidine deaminase [Source:HGNC Symbol;Acc:HGNC:1712] | 14101 | -0.449 | 0.0222 | Yes |
| 145 | NPR2 | natriuretic peptide receptor 2 [Source:HGNC Symbol;Acc:HGNC:7944] | 14188 | -0.464 | 0.0315 | Yes |
| 146 | IMPDH2 | inosine monophosphate dehydrogenase 2 [Source:HGNC Symbol;Acc:HGNC:6053] | 14589 | -0.530 | 0.0228 | Yes |
| 147 | NME4 | NME/NM23 nucleoside diphosphate kinase 4 [Source:HGNC Symbol;Acc:HGNC:7852] | 14715 | -0.557 | 0.0327 | Yes |
| 148 | AK1 | adenylate kinase 1 [Source:HGNC Symbol;Acc:HGNC:361] | 15402 | -0.892 | 0.0171 | Yes |
Table: GSEA details [plain text format]

  

Fig 2: HALLMARK\_DNA\_REPAIR      
 Blue-Pink O' Gram in the Space of the Analyzed GeneSet

  

Fig 3: HALLMARK\_DNA\_REPAIR: Random ES distribution      
 Gene set null distribution of ES for **HALLMARK\_DNA\_REPAIR**
