# Supplementary material for: In vivo CRISPR screens reveal Serpinb9 and Adam2 as regulators of immune therapy response in lung cancer
Source: Nat Commun. 2023 May 31;14:3150. doi: 10.1038/s41467-023-38841-7 (PMC10232477; doi:10.1038/s41467-023-38841-7)
Supplement: Supplementary file 13 — Source Data [file 41467_2023_38841_MOESM13_ESM.zip › Figure 4E.zip!/my_analysis.Gsea.1640146340255_Hallmark/HALLMARK_APOPTOSIS.html]

Details for gene set HALLMARK\_APOPTOSIS[GSEA]

|  || Dataset | December 21, 2021\_for GSEA run\_collapsed\_to\_symbols.GSEA\_B6\_NI.cls #CTLR\_NI\_versus\_ADAM2\_NI |
| Phenotype | GSEA\_B6\_NI.cls#CTLR\_NI\_versus\_ADAM2\_NI |
| Upregulated in class | CTLR\_NI |
| GeneSet | HALLMARK\_APOPTOSIS |
| Enrichment Score (ES) | 0.34437367 |
| Normalized Enrichment Score (NES) | 1.3909565 |
| Nominal p-value | 0.0 |
| FDR q-value | 0.28969762 |
| FWER p-Value | 0.748 |
Table: GSEA Results Summary

  

Fig 1: Enrichment plot: HALLMARK\_APOPTOSIS      
 Profile of the Running ES Score & Positions of GeneSet Members on the Rank Ordered List

  

| SYMBOL | TITLE | RANK IN GENE LIST | RANK METRIC SCORE | RUNNING ES | CORE ENRICHMENT || 1 | FASLG | Fas ligand [Source:HGNC Symbol;Acc:HGNC:11936] | 68 | 1.912 | 0.0247 | Yes |
| 2 | TNFSF10 | TNF superfamily member 10 [Source:HGNC Symbol;Acc:HGNC:11925] | 125 | 1.728 | 0.0474 | Yes |
| 3 | TAP1 | "transporter 1, ATP binding cassette subfamily B member [Source:HGNC Symbol;Acc:HGNC:43]" | 241 | 1.389 | 0.0611 | Yes |
| 4 | CASP1 | caspase 1 [Source:HGNC Symbol;Acc:HGNC:1499] | 248 | 1.383 | 0.0818 | Yes |
| 5 | CD2 | CD2 molecule [Source:HGNC Symbol;Acc:HGNC:1639] | 249 | 1.381 | 0.1028 | Yes |
| 6 | IRF1 | interferon regulatory factor 1 [Source:HGNC Symbol;Acc:HGNC:6116] | 295 | 1.310 | 0.1199 | Yes |
| 7 | SATB1 | SATB homeobox 1 [Source:HGNC Symbol;Acc:HGNC:10541] | 300 | 1.300 | 0.1394 | Yes |
| 8 | IL1B | interleukin 1 beta [Source:HGNC Symbol;Acc:HGNC:5992] | 303 | 1.298 | 0.1590 | Yes |
| 9 | CASP4 | caspase 4 [Source:HGNC Symbol;Acc:HGNC:1505] | 358 | 1.219 | 0.1741 | Yes |
| 10 | BIRC3 | baculoviral IAP repeat containing 3 [Source:HGNC Symbol;Acc:HGNC:591] | 369 | 1.208 | 0.1919 | Yes |
| 11 | IL6 | interleukin 6 [Source:HGNC Symbol;Acc:HGNC:6018] | 378 | 1.200 | 0.2096 | Yes |
| 12 | FAS | Fas cell surface death receptor [Source:HGNC Symbol;Acc:HGNC:11920] | 418 | 1.154 | 0.2247 | Yes |
| 13 | HGF | hepatocyte growth factor [Source:HGNC Symbol;Acc:HGNC:4893] | 431 | 1.141 | 0.2413 | Yes |
| 14 | ATF3 | activating transcription factor 3 [Source:HGNC Symbol;Acc:HGNC:785] | 453 | 1.117 | 0.2569 | Yes |
| 15 | CD69 | CD69 molecule [Source:HGNC Symbol;Acc:HGNC:1694] | 476 | 1.094 | 0.2722 | Yes |
| 16 | IL1A | interleukin 1 alpha [Source:HGNC Symbol;Acc:HGNC:5991] | 540 | 1.032 | 0.2838 | Yes |
| 17 | GADD45B | growth arrest and DNA damage inducible beta [Source:HGNC Symbol;Acc:HGNC:4096] | 667 | 0.954 | 0.2902 | Yes |
| 18 | TIMP3 | TIMP metallopeptidase inhibitor 3 [Source:HGNC Symbol;Acc:HGNC:11822] | 707 | 0.927 | 0.3018 | Yes |
| 19 | CASP7 | caspase 7 [Source:HGNC Symbol;Acc:HGNC:1508] | 1011 | 0.810 | 0.2946 | Yes |
| 20 | ERBB3 | erb-b2 receptor tyrosine kinase 3 [Source:HGNC Symbol;Acc:HGNC:3431] | 1107 | 0.770 | 0.3002 | Yes |
| 21 | LEF1 | lymphoid enhancer binding factor 1 [Source:HGNC Symbol;Acc:HGNC:6551] | 1114 | 0.765 | 0.3115 | Yes |
| 22 | RNASEL | ribonuclease L [Source:HGNC Symbol;Acc:HGNC:10050] | 1259 | 0.711 | 0.3130 | Yes |
| 23 | PSEN2 | presenilin 2 [Source:HGNC Symbol;Acc:HGNC:9509] | 1280 | 0.703 | 0.3224 | Yes |
| 24 | GPX3 | glutathione peroxidase 3 [Source:HGNC Symbol;Acc:HGNC:4555] | 1519 | 0.619 | 0.3165 | Yes |
| 25 | ROCK1 | Rho associated coiled-coil containing protein kinase 1 [Source:HGNC Symbol;Acc:HGNC:10251] | 1606 | 0.593 | 0.3200 | Yes |
| 26 | IL18 | interleukin 18 [Source:HGNC Symbol;Acc:HGNC:5986] | 1622 | 0.588 | 0.3280 | Yes |
| 27 | PLCB2 | phospholipase C beta 2 [Source:HGNC Symbol;Acc:HGNC:9055] | 1815 | 0.540 | 0.3238 | Yes |
| 28 | LUM | lumican [Source:HGNC Symbol;Acc:HGNC:6724] | 1837 | 0.536 | 0.3306 | Yes |
| 29 | TNF | tumor necrosis factor [Source:HGNC Symbol;Acc:HGNC:11892] | 1880 | 0.528 | 0.3360 | Yes |
| 30 | IFITM3 | interferon induced transmembrane protein 3 [Source:HGNC Symbol;Acc:HGNC:5414] | 1928 | 0.517 | 0.3408 | Yes |
| 31 | NEFH | neurofilament heavy [Source:HGNC Symbol;Acc:HGNC:7737] | 1993 | 0.505 | 0.3444 | Yes |
| 32 | MMP2 | matrix metallopeptidase 2 [Source:HGNC Symbol;Acc:HGNC:7166] | 2247 | 0.477 | 0.3353 | No |
| 33 | ISG20 | interferon stimulated exonuclease gene 20 [Source:HGNC Symbol;Acc:HGNC:6130] | 2346 | 0.460 | 0.3360 | No |
| 34 | CASP8 | caspase 8 [Source:HGNC Symbol;Acc:HGNC:1509] | 2434 | 0.446 | 0.3372 | No |
| 35 | GCH1 | GTP cyclohydrolase 1 [Source:HGNC Symbol;Acc:HGNC:4193] | 2566 | 0.441 | 0.3355 | No |
| 36 | APP | amyloid beta precursor protein [Source:HGNC Symbol;Acc:HGNC:620] | 2788 | 0.418 | 0.3276 | No |
| 37 | MADD | MAP kinase activating death domain [Source:HGNC Symbol;Acc:HGNC:6766] | 3062 | 0.393 | 0.3160 | No |
| 38 | BGN | biglycan [Source:HGNC Symbol;Acc:HGNC:1044] | 3138 | 0.382 | 0.3169 | No |
| 39 | CREBBP | CREB binding protein [Source:HGNC Symbol;Acc:HGNC:2348] | 3194 | 0.374 | 0.3191 | No |
| 40 | BCL2L2 | BCL2 like 2 [Source:HGNC Symbol;Acc:HGNC:995] | 3228 | 0.369 | 0.3226 | No |
| 41 | CTH | cystathionine gamma-lyase [Source:HGNC Symbol;Acc:HGNC:2501] | 3305 | 0.356 | 0.3231 | No |
| 42 | BTG2 | BTG anti-proliferation factor 2 [Source:HGNC Symbol;Acc:HGNC:1131] | 3319 | 0.354 | 0.3277 | No |
| 43 | SC5D | sterol-C5-desaturase [Source:HGNC Symbol;Acc:HGNC:10547] | 3356 | 0.349 | 0.3306 | No |
| 44 | AIFM3 | apoptosis inducing factor mitochondria associated 3 [Source:HGNC Symbol;Acc:HGNC:26398] | 3371 | 0.347 | 0.3350 | No |
| 45 | SLC20A1 | solute carrier family 20 member 1 [Source:HGNC Symbol;Acc:HGNC:10946] | 3479 | 0.331 | 0.3332 | No |
| 46 | TOP2A | DNA topoisomerase II alpha [Source:HGNC Symbol;Acc:HGNC:11989] | 3608 | 0.314 | 0.3297 | No |
| 47 | DNM1L | dynamin 1 like [Source:HGNC Symbol;Acc:HGNC:2973] | 3766 | 0.294 | 0.3240 | No |
| 48 | CFLAR | CASP8 and FADD like apoptosis regulator [Source:HGNC Symbol;Acc:HGNC:1876] | 3779 | 0.293 | 0.3277 | No |
| 49 | PRF1 | perforin 1 [Source:HGNC Symbol;Acc:HGNC:9360] | 3794 | 0.292 | 0.3313 | No |
| 50 | VDAC2 | voltage dependent anion channel 2 [Source:HGNC Symbol;Acc:HGNC:12672] | 3934 | 0.275 | 0.3265 | No |
| 51 | EGR3 | early growth response 3 [Source:HGNC Symbol;Acc:HGNC:3240] | 3940 | 0.274 | 0.3303 | No |
| 52 | HMGB2 | high mobility group box 2 [Source:HGNC Symbol;Acc:HGNC:5000] | 3990 | 0.267 | 0.3312 | No |
| 53 | BRCA1 | BRCA1 DNA repair associated [Source:HGNC Symbol;Acc:HGNC:1100] | 4003 | 0.265 | 0.3345 | No |
| 54 | ETF1 | eukaryotic translation termination factor 1 [Source:HGNC Symbol;Acc:HGNC:3477] | 4021 | 0.263 | 0.3374 | No |
| 55 | BCL2L11 | BCL2 like 11 [Source:HGNC Symbol;Acc:HGNC:994] | 4073 | 0.257 | 0.3380 | No |
| 56 | LGALS3 | galectin 3 [Source:HGNC Symbol;Acc:HGNC:6563] | 4079 | 0.256 | 0.3416 | No |
| 57 | PAK1 | p21 (RAC1) activated kinase 1 [Source:HGNC Symbol;Acc:HGNC:8590] | 4171 | 0.247 | 0.3395 | No |
| 58 | BID | BH3 interacting domain death agonist [Source:HGNC Symbol;Acc:HGNC:1050] | 4177 | 0.246 | 0.3429 | No |
| 59 | EREG | epiregulin [Source:HGNC Symbol;Acc:HGNC:3443] | 4321 | 0.231 | 0.3372 | No |
| 60 | CDK2 | cyclin dependent kinase 2 [Source:HGNC Symbol;Acc:HGNC:1771] | 4345 | 0.228 | 0.3392 | No |
| 61 | CASP6 | caspase 6 [Source:HGNC Symbol;Acc:HGNC:1507] | 4374 | 0.225 | 0.3408 | No |
| 62 | DDIT3 | DNA damage inducible transcript 3 [Source:HGNC Symbol;Acc:HGNC:2726] | 4509 | 0.212 | 0.3354 | No |
| 63 | GSR | glutathione-disulfide reductase [Source:HGNC Symbol;Acc:HGNC:4623] | 4657 | 0.200 | 0.3290 | No |
| 64 | CYLD | CYLD lysine 63 deubiquitinase [Source:HGNC Symbol;Acc:HGNC:2584] | 4705 | 0.196 | 0.3289 | No |
| 65 | CDKN1B | cyclin dependent kinase inhibitor 1B [Source:HGNC Symbol;Acc:HGNC:1785] | 4931 | 0.175 | 0.3171 | No |
| 66 | TIMP1 | TIMP metallopeptidase inhibitor 1 [Source:HGNC Symbol;Acc:HGNC:11820] | 4971 | 0.171 | 0.3172 | No |
| 67 | CD38 | CD38 molecule [Source:HGNC Symbol;Acc:HGNC:1667] | 5197 | 0.151 | 0.3050 | No |
| 68 | PEA15 | proliferation and apoptosis adaptor protein 15 [Source:HGNC Symbol;Acc:HGNC:8822] | 5227 | 0.148 | 0.3054 | No |
| 69 | CD14 | CD14 molecule [Source:HGNC Symbol;Acc:HGNC:1628] | 5473 | 0.129 | 0.2915 | No |
| 70 | EBP | EBP cholestenol delta-isomerase [Source:HGNC Symbol;Acc:HGNC:3133] | 5537 | 0.125 | 0.2894 | No |
| 71 | GNA15 | G protein subunit alpha 15 [Source:HGNC Symbol;Acc:HGNC:4383] | 5582 | 0.121 | 0.2884 | No |
| 72 | CASP9 | caspase 9 [Source:HGNC Symbol;Acc:HGNC:1511] | 5704 | 0.112 | 0.2823 | No |
| 73 | TXNIP | thioredoxin interacting protein [Source:HGNC Symbol;Acc:HGNC:16952] | 5781 | 0.106 | 0.2790 | No |
| 74 | SMAD7 | SMAD family member 7 [Source:HGNC Symbol;Acc:HGNC:6773] | 5911 | 0.098 | 0.2722 | No |
| 75 | PPT1 | palmitoyl-protein thioesterase 1 [Source:HGNC Symbol;Acc:HGNC:9325] | 5954 | 0.095 | 0.2709 | No |
| 76 | ENO2 | enolase 2 [Source:HGNC Symbol;Acc:HGNC:3353] | 5991 | 0.092 | 0.2700 | No |
| 77 | SAT1 | spermidine/spermine N1-acetyltransferase 1 [Source:HGNC Symbol;Acc:HGNC:10540] | 6202 | 0.076 | 0.2576 | No |
| 78 | DCN | decorin [Source:HGNC Symbol;Acc:HGNC:2705] | 6238 | 0.074 | 0.2565 | No |
| 79 | MCL1 | "MCL1 apoptosis regulator, BCL2 family member [Source:HGNC Symbol;Acc:HGNC:6943]" | 6249 | 0.073 | 0.2570 | No |
| 80 | CASP3 | caspase 3 [Source:HGNC Symbol;Acc:HGNC:1504] | 6509 | 0.054 | 0.2411 | No |
| 81 | HSPB1 | heat shock protein family B (small) member 1 [Source:HGNC Symbol;Acc:HGNC:5246] | 6615 | 0.048 | 0.2350 | No |
| 82 | BTG3 | BTG anti-proliferation factor 3 [Source:HGNC Symbol;Acc:HGNC:1132] | 6622 | 0.047 | 0.2354 | No |
| 83 | HMOX1 | heme oxygenase 1 [Source:HGNC Symbol;Acc:HGNC:5013] | 6937 | 0.027 | 0.2155 | No |
| 84 | F2 | "coagulation factor II, thrombin [Source:HGNC Symbol;Acc:HGNC:3535]" | 6945 | 0.026 | 0.2155 | No |
| 85 | IFNB1 | interferon beta 1 [Source:HGNC Symbol;Acc:HGNC:5434] | 6951 | 0.026 | 0.2156 | No |
| 86 | PLPPR4 | phospholipid phosphatase related 4 [Source:HGNC Symbol;Acc:HGNC:23496] | 6956 | 0.026 | 0.2157 | No |
| 87 | TSPO | translocator protein [Source:HGNC Symbol;Acc:HGNC:1158] | 7047 | 0.021 | 0.2102 | No |
| 88 | PTK2 | protein tyrosine kinase 2 [Source:HGNC Symbol;Acc:HGNC:9611] | 7196 | 0.012 | 0.2009 | No |
| 89 | PSEN1 | presenilin 1 [Source:HGNC Symbol;Acc:HGNC:9508] | 7304 | 0.006 | 0.1941 | No |
| 90 | XIAP | X-linked inhibitor of apoptosis [Source:HGNC Symbol;Acc:HGNC:592] | 7316 | 0.005 | 0.1935 | No |
| 91 | DPYD | dihydropyrimidine dehydrogenase [Source:HGNC Symbol;Acc:HGNC:3012] | 7433 | -0.001 | 0.1860 | No |
| 92 | CASP2 | caspase 2 [Source:HGNC Symbol;Acc:HGNC:1503] | 7445 | -0.001 | 0.1853 | No |
| 93 | EMP1 | epithelial membrane protein 1 [Source:HGNC Symbol;Acc:HGNC:3333] | 7529 | -0.006 | 0.1800 | No |
| 94 | IFNGR1 | interferon gamma receptor 1 [Source:HGNC Symbol;Acc:HGNC:5439] | 7599 | -0.009 | 0.1757 | No |
| 95 | BNIP3L | BCL2 interacting protein 3 like [Source:HGNC Symbol;Acc:HGNC:1085] | 7600 | -0.009 | 0.1759 | No |
| 96 | SOD2 | superoxide dismutase 2 [Source:HGNC Symbol;Acc:HGNC:11180] | 7867 | -0.022 | 0.1591 | No |
| 97 | CCND2 | cyclin D2 [Source:HGNC Symbol;Acc:HGNC:1583] | 8119 | -0.036 | 0.1434 | No |
| 98 | WEE1 | WEE1 G2 checkpoint kinase [Source:HGNC Symbol;Acc:HGNC:12761] | 8346 | -0.046 | 0.1296 | No |
| 99 | RHOT2 | ras homolog family member T2 [Source:HGNC Symbol;Acc:HGNC:21169] | 8597 | -0.060 | 0.1144 | No |
| 100 | DFFA | DNA fragmentation factor subunit alpha [Source:HGNC Symbol;Acc:HGNC:2772] | 9023 | -0.084 | 0.0882 | No |
| 101 | KRT18 | keratin 18 [Source:HGNC Symbol;Acc:HGNC:6430] | 9222 | -0.094 | 0.0769 | No |
| 102 | CTNNB1 | catenin beta 1 [Source:HGNC Symbol;Acc:HGNC:2514] | 9410 | -0.105 | 0.0664 | No |
| 103 | SPTAN1 | "spectrin alpha, non-erythrocytic 1 [Source:HGNC Symbol;Acc:HGNC:11273]" | 9660 | -0.122 | 0.0523 | No |
| 104 | PPP3R1 | "protein phosphatase 3 regulatory subunit B, alpha [Source:HGNC Symbol;Acc:HGNC:9317]" | 9692 | -0.124 | 0.0521 | No |
| 105 | CCND1 | cyclin D1 [Source:HGNC Symbol;Acc:HGNC:1582] | 9851 | -0.134 | 0.0440 | No |
| 106 | PPP2R5B | protein phosphatase 2 regulatory subunit B'beta [Source:HGNC Symbol;Acc:HGNC:9310] | 10018 | -0.143 | 0.0355 | No |
| 107 | DNAJA1 | DnaJ heat shock protein family (Hsp40) member A1 [Source:HGNC Symbol;Acc:HGNC:5229] | 10174 | -0.152 | 0.0278 | No |
| 108 | JUN | "Jun proto-oncogene, AP-1 transcription factor subunit [Source:HGNC Symbol;Acc:HGNC:6204]" | 10257 | -0.157 | 0.0249 | No |
| 109 | PMAIP1 | phorbol-12-myristate-13-acetate-induced protein 1 [Source:HGNC Symbol;Acc:HGNC:9108] | 10468 | -0.169 | 0.0139 | No |
| 110 | CD44 | CD44 molecule (Indian blood group) [Source:HGNC Symbol;Acc:HGNC:1681] | 10617 | -0.178 | 0.0071 | No |
| 111 | DNAJC3 | DnaJ heat shock protein family (Hsp40) member C3 [Source:HGNC Symbol;Acc:HGNC:9439] | 10973 | -0.199 | -0.0128 | No |
| 112 | SOD1 | superoxide dismutase 1 [Source:HGNC Symbol;Acc:HGNC:11179] | 10977 | -0.199 | -0.0099 | No |
| 113 | GPX1 | glutathione peroxidase 1 [Source:HGNC Symbol;Acc:HGNC:4553] | 11084 | -0.205 | -0.0136 | No |
| 114 | RHOB | ras homolog family member B [Source:HGNC Symbol;Acc:HGNC:668] | 11216 | -0.212 | -0.0189 | No |
| 115 | ADD1 | adducin 1 [Source:HGNC Symbol;Acc:HGNC:243] | 11225 | -0.213 | -0.0161 | No |
| 116 | GPX4 | glutathione peroxidase 4 [Source:HGNC Symbol;Acc:HGNC:4556] | 11537 | -0.231 | -0.0327 | No |
| 117 | RARA | retinoic acid receptor alpha [Source:HGNC Symbol;Acc:HGNC:9864] | 11774 | -0.246 | -0.0441 | No |
| 118 | CDC25B | cell division cycle 25B [Source:HGNC Symbol;Acc:HGNC:1726] | 11820 | -0.249 | -0.0432 | No |
| 119 | DAP3 | death associated protein 3 [Source:HGNC Symbol;Acc:HGNC:2673] | 11894 | -0.253 | -0.0441 | No |
| 120 | CLU | clusterin [Source:HGNC Symbol;Acc:HGNC:2095] | 11910 | -0.254 | -0.0412 | No |
| 121 | GADD45A | growth arrest and DNA damage inducible alpha [Source:HGNC Symbol;Acc:HGNC:4095] | 12093 | -0.267 | -0.0489 | No |
| 122 | BCL2L1 | BCL2 like 1 [Source:HGNC Symbol;Acc:HGNC:992] | 12157 | -0.271 | -0.0488 | No |
| 123 | FDXR | ferredoxin reductase [Source:HGNC Symbol;Acc:HGNC:3642] | 12240 | -0.277 | -0.0499 | No |
| 124 | TGFBR3 | transforming growth factor beta receptor 3 [Source:HGNC Symbol;Acc:HGNC:11774] | 12373 | -0.286 | -0.0540 | No |
| 125 | IGF2R | insulin like growth factor 2 receptor [Source:HGNC Symbol;Acc:HGNC:5467] | 12501 | -0.295 | -0.0577 | No |
| 126 | BMP2 | bone morphogenetic protein 2 [Source:HGNC Symbol;Acc:HGNC:1069] | 12618 | -0.305 | -0.0606 | No |
| 127 | RELA | "RELA proto-oncogene, NF-kB subunit [Source:HGNC Symbol;Acc:HGNC:9955]" | 12655 | -0.308 | -0.0582 | No |
| 128 | DIABLO | diablo IAP-binding mitochondrial protein [Source:HGNC Symbol;Acc:HGNC:21528] | 12789 | -0.318 | -0.0619 | No |
| 129 | IER3 | immediate early response 3 [Source:HGNC Symbol;Acc:HGNC:5392] | 12833 | -0.322 | -0.0598 | No |
| 130 | ANXA1 | annexin A1 [Source:HGNC Symbol;Acc:HGNC:533] | 12834 | -0.322 | -0.0549 | No |
| 131 | BCL10 | BCL10 immune signaling adaptor [Source:HGNC Symbol;Acc:HGNC:989] | 13120 | -0.349 | -0.0679 | No |
| 132 | BCAP31 | B cell receptor associated protein 31 [Source:HGNC Symbol;Acc:HGNC:16695] | 13210 | -0.357 | -0.0683 | No |
| 133 | ERBB2 | erb-b2 receptor tyrosine kinase 2 [Source:HGNC Symbol;Acc:HGNC:3430] | 13227 | -0.359 | -0.0638 | No |
| 134 | BIK | BCL2 interacting killer [Source:HGNC Symbol;Acc:HGNC:1051] | 13352 | -0.373 | -0.0661 | No |
| 135 | SQSTM1 | sequestosome 1 [Source:HGNC Symbol;Acc:HGNC:11280] | 13604 | -0.406 | -0.0762 | No |
| 136 | PDCD4 | programmed cell death 4 [Source:HGNC Symbol;Acc:HGNC:8763] | 13615 | -0.407 | -0.0706 | No |
| 137 | LMNA | lamin A/C [Source:HGNC Symbol;Acc:HGNC:6636] | 13663 | -0.413 | -0.0674 | No |
| 138 | IGFBP6 | insulin like growth factor binding protein 6 [Source:HGNC Symbol;Acc:HGNC:5475] | 13981 | -0.429 | -0.0813 | No |
| 139 | ANKH | ANKH inorganic pyrophosphate transport regulator [Source:HGNC Symbol;Acc:HGNC:15492] | 14048 | -0.439 | -0.0788 | No |
| 140 | TGFB2 | transforming growth factor beta 2 [Source:HGNC Symbol;Acc:HGNC:11768] | 14145 | -0.457 | -0.0781 | No |
| 141 | FEZ1 | fasciculation and elongation protein zeta 1 [Source:HGNC Symbol;Acc:HGNC:3659] | 14184 | -0.463 | -0.0735 | No |
| 142 | GSN | gelsolin [Source:HGNC Symbol;Acc:HGNC:4620] | 14267 | -0.475 | -0.0715 | No |
| 143 | AVPR1A | arginine vasopressin receptor 1A [Source:HGNC Symbol;Acc:HGNC:895] | 14293 | -0.481 | -0.0658 | No |
| 144 | PLAT | "plasminogen activator, tissue type [Source:HGNC Symbol;Acc:HGNC:9051]" | 14379 | -0.490 | -0.0638 | No |
| 145 | BAX | "BCL2 associated X, apoptosis regulator [Source:HGNC Symbol;Acc:HGNC:959]" | 14408 | -0.493 | -0.0581 | No |
| 146 | PDGFRB | platelet derived growth factor receptor beta [Source:HGNC Symbol;Acc:HGNC:8804] | 14530 | -0.521 | -0.0580 | No |
| 147 | H1-0 | H1.0 linker histone [Source:HGNC Symbol;Acc:HGNC:4714] | 14582 | -0.529 | -0.0532 | No |
| 148 | GSTM1 | glutathione S-transferase mu 1 [Source:HGNC Symbol;Acc:HGNC:4632] | 14659 | -0.544 | -0.0499 | No |
| 149 | DAP | death associated protein [Source:HGNC Symbol;Acc:HGNC:2672] | 14799 | -0.583 | -0.0500 | No |
| 150 | TNFRSF12A | TNF receptor superfamily member 12A [Source:HGNC Symbol;Acc:HGNC:18152] | 14839 | -0.595 | -0.0434 | No |
| 151 | TIMP2 | TIMP metallopeptidase inhibitor 2 [Source:HGNC Symbol;Acc:HGNC:11821] | 14863 | -0.603 | -0.0357 | No |
| 152 | RETSAT | retinol saturase [Source:HGNC Symbol;Acc:HGNC:25991] | 14885 | -0.613 | -0.0277 | No |
| 153 | BMF | Bcl2 modifying factor [Source:HGNC Symbol;Acc:HGNC:24132] | 15009 | -0.668 | -0.0255 | No |
| 154 | CAV1 | caveolin 1 [Source:HGNC Symbol;Acc:HGNC:1527] | 15057 | -0.694 | -0.0180 | No |
| 155 | NEDD9 | "neural precursor cell expressed, developmentally down-regulated 9 [Source:HGNC Symbol;Acc:HGNC:7733]" | 15128 | -0.734 | -0.0113 | No |
| 156 | F2R | coagulation factor II thrombin receptor [Source:HGNC Symbol;Acc:HGNC:3537] | 15375 | -0.870 | -0.0139 | No |
| 157 | CDKN1A | cyclin dependent kinase inhibitor 1A [Source:HGNC Symbol;Acc:HGNC:1784] | 15428 | -0.929 | -0.0031 | No |
| 158 | MGMT | O-6-methylguanine-DNA methyltransferase [Source:HGNC Symbol;Acc:HGNC:7059] | 15574 | -1.217 | 0.0061 | No |
Table: GSEA details [plain text format]

  

Fig 2: HALLMARK\_APOPTOSIS      
 Blue-Pink O' Gram in the Space of the Analyzed GeneSet

  

Fig 3: HALLMARK\_APOPTOSIS: Random ES distribution      
 Gene set null distribution of ES for **HALLMARK\_APOPTOSIS**
