# Supplementary material for: In vivo CRISPR screens reveal Serpinb9 and Adam2 as regulators of immune therapy response in lung cancer
Source: Nat Commun. 2023 May 31;14:3150. doi: 10.1038/s41467-023-38841-7 (PMC10232477; doi:10.1038/s41467-023-38841-7)
Supplement: Supplementary file 13 — Source Data [file 41467_2023_38841_MOESM13_ESM.zip › Figure 4E.zip!/my_analysis.Gsea.1640146340255_Hallmark/HALLMARK_APICAL_JUNCTION.html]

Details for gene set HALLMARK\_APICAL\_JUNCTION[GSEA]

|  || Dataset | December 21, 2021\_for GSEA run\_collapsed\_to\_symbols.GSEA\_B6\_NI.cls #CTLR\_NI\_versus\_ADAM2\_NI |
| Phenotype | GSEA\_B6\_NI.cls#CTLR\_NI\_versus\_ADAM2\_NI |
| Upregulated in class | CTLR\_NI |
| GeneSet | HALLMARK\_APICAL\_JUNCTION |
| Enrichment Score (ES) | 0.23900014 |
| Normalized Enrichment Score (NES) | 0.99592924 |
| Nominal p-value | 0.3833017 |
| FDR q-value | 0.79869473 |
| FWER p-Value | 1.0 |
Table: GSEA Results Summary

  

Fig 1: Enrichment plot: HALLMARK\_APICAL\_JUNCTION      
 Profile of the Running ES Score & Positions of GeneSet Members on the Rank Ordered List

  

| SYMBOL | TITLE | RANK IN GENE LIST | RANK METRIC SCORE | RUNNING ES | CORE ENRICHMENT || 1 | CD274 | CD274 molecule [Source:HGNC Symbol;Acc:HGNC:17635] | 90 | 1.821 | 0.0195 | Yes |
| 2 | ACTN2 | actinin alpha 2 [Source:HGNC Symbol;Acc:HGNC:164] | 288 | 1.324 | 0.0251 | Yes |
| 3 | NEGR1 | neuronal growth regulator 1 [Source:HGNC Symbol;Acc:HGNC:17302] | 294 | 1.313 | 0.0430 | Yes |
| 4 | MAPK13 | mitogen-activated protein kinase 13 [Source:HGNC Symbol;Acc:HGNC:6875] | 412 | 1.159 | 0.0516 | Yes |
| 5 | NLGN3 | neuroligin 3 [Source:HGNC Symbol;Acc:HGNC:14289] | 416 | 1.156 | 0.0675 | Yes |
| 6 | SDC3 | syndecan 3 [Source:HGNC Symbol;Acc:HGNC:10660] | 465 | 1.102 | 0.0797 | Yes |
| 7 | CD86 | CD86 molecule [Source:HGNC Symbol;Acc:HGNC:1705] | 518 | 1.048 | 0.0909 | Yes |
| 8 | FYB1 | FYN binding protein 1 [Source:HGNC Symbol;Acc:HGNC:4036] | 563 | 1.019 | 0.1022 | Yes |
| 9 | SYK | spleen associated tyrosine kinase [Source:HGNC Symbol;Acc:HGNC:11491] | 630 | 0.970 | 0.1114 | Yes |
| 10 | NECTIN4 | nectin cell adhesion molecule 4 [Source:HGNC Symbol;Acc:HGNC:19688] | 718 | 0.922 | 0.1186 | Yes |
| 11 | PTPRC | protein tyrosine phosphatase receptor type C [Source:HGNC Symbol;Acc:HGNC:9666] | 756 | 0.894 | 0.1286 | Yes |
| 12 | GRB7 | growth factor receptor bound protein 7 [Source:HGNC Symbol;Acc:HGNC:4567] | 762 | 0.892 | 0.1407 | Yes |
| 13 | MADCAM1 | mucosal vascular addressin cell adhesion molecule 1 [Source:HGNC Symbol;Acc:HGNC:6765] | 839 | 0.864 | 0.1478 | Yes |
| 14 | NFASC | neurofascin [Source:HGNC Symbol;Acc:HGNC:29866] | 882 | 0.845 | 0.1568 | Yes |
| 15 | ACTA1 | "actin alpha 1, skeletal muscle [Source:HGNC Symbol;Acc:HGNC:129]" | 1062 | 0.787 | 0.1562 | Yes |
| 16 | TRAF1 | TNF receptor associated factor 1 [Source:HGNC Symbol;Acc:HGNC:12031] | 1077 | 0.781 | 0.1661 | Yes |
| 17 | PDZD3 | PDZ domain containing 3 [Source:HGNC Symbol;Acc:HGNC:19891] | 1099 | 0.771 | 0.1755 | Yes |
| 18 | ITGA2 | integrin subunit alpha 2 [Source:HGNC Symbol;Acc:HGNC:6137] | 1109 | 0.769 | 0.1856 | Yes |
| 19 | ICAM1 | intercellular adhesion molecule 1 [Source:HGNC Symbol;Acc:HGNC:5344] | 1112 | 0.767 | 0.1961 | Yes |
| 20 | ARHGEF6 | Rac/Cdc42 guanine nucleotide exchange factor 6 [Source:HGNC Symbol;Acc:HGNC:685] | 1236 | 0.719 | 0.1981 | Yes |
| 21 | KCNH2 | potassium voltage-gated channel subfamily H member 2 [Source:HGNC Symbol;Acc:HGNC:6251] | 1277 | 0.704 | 0.2053 | Yes |
| 22 | LAMB3 | laminin subunit beta 3 [Source:HGNC Symbol;Acc:HGNC:6490] | 1282 | 0.701 | 0.2148 | Yes |
| 23 | CERCAM | cerebral endothelial cell adhesion molecule [Source:HGNC Symbol;Acc:HGNC:23723] | 1421 | 0.653 | 0.2149 | Yes |
| 24 | RAC2 | Rac family small GTPase 2 [Source:HGNC Symbol;Acc:HGNC:9802] | 1453 | 0.641 | 0.2218 | Yes |
| 25 | TGFBI | transforming growth factor beta induced [Source:HGNC Symbol;Acc:HGNC:11771] | 1655 | 0.577 | 0.2169 | Yes |
| 26 | ADAM23 | ADAM metallopeptidase domain 23 [Source:HGNC Symbol;Acc:HGNC:202] | 1678 | 0.574 | 0.2234 | Yes |
| 27 | SIRPA | signal regulatory protein alpha [Source:HGNC Symbol;Acc:HGNC:9662] | 1858 | 0.532 | 0.2193 | Yes |
| 28 | MPZL2 | myelin protein zero like 2 [Source:HGNC Symbol;Acc:HGNC:3496] | 1897 | 0.525 | 0.2241 | Yes |
| 29 | CDH11 | cadherin 11 [Source:HGNC Symbol;Acc:HGNC:1750] | 1959 | 0.511 | 0.2273 | Yes |
| 30 | ACTN3 | actinin alpha 3 [Source:HGNC Symbol;Acc:HGNC:165] | 2172 | 0.485 | 0.2203 | Yes |
| 31 | MMP2 | matrix metallopeptidase 2 [Source:HGNC Symbol;Acc:HGNC:7166] | 2247 | 0.477 | 0.2221 | Yes |
| 32 | NRAP | nebulin related anchoring protein [Source:HGNC Symbol;Acc:HGNC:7988] | 2258 | 0.475 | 0.2281 | Yes |
| 33 | TRO | trophinin [Source:HGNC Symbol;Acc:HGNC:12326] | 2342 | 0.461 | 0.2291 | Yes |
| 34 | AMH | anti-Mullerian hormone [Source:HGNC Symbol;Acc:HGNC:464] | 2423 | 0.448 | 0.2302 | Yes |
| 35 | CDH6 | cadherin 6 [Source:HGNC Symbol;Acc:HGNC:1765] | 2530 | 0.445 | 0.2295 | Yes |
| 36 | CLDN7 | claudin 7 [Source:HGNC Symbol;Acc:HGNC:2049] | 2548 | 0.443 | 0.2346 | Yes |
| 37 | PPP2R2C | protein phosphatase 2 regulatory subunit Bgamma [Source:HGNC Symbol;Acc:HGNC:9306] | 2663 | 0.426 | 0.2331 | Yes |
| 38 | COL9A1 | collagen type IX alpha 1 chain [Source:HGNC Symbol;Acc:HGNC:2217] | 2682 | 0.426 | 0.2379 | Yes |
| 39 | ADAMTS5 | ADAM metallopeptidase with thrombospondin type 1 motif 5 [Source:HGNC Symbol;Acc:HGNC:221] | 2757 | 0.425 | 0.2390 | Yes |
| 40 | ITGA9 | integrin subunit alpha 9 [Source:HGNC Symbol;Acc:HGNC:6145] | 2970 | 0.409 | 0.2310 | No |
| 41 | MMP9 | matrix metallopeptidase 9 [Source:HGNC Symbol;Acc:HGNC:7176] | 3018 | 0.401 | 0.2335 | No |
| 42 | CDH15 | cadherin 15 [Source:HGNC Symbol;Acc:HGNC:1754] | 3137 | 0.383 | 0.2312 | No |
| 43 | CD209 | CD209 molecule [Source:HGNC Symbol;Acc:HGNC:1641] | 3144 | 0.381 | 0.2361 | No |
| 44 | VCL | vinculin [Source:HGNC Symbol;Acc:HGNC:12665] | 3348 | 0.350 | 0.2279 | No |
| 45 | LAMA3 | laminin subunit alpha 3 [Source:HGNC Symbol;Acc:HGNC:6483] | 3398 | 0.341 | 0.2294 | No |
| 46 | WNK4 | WNK lysine deficient protein kinase 4 [Source:HGNC Symbol;Acc:HGNC:14544] | 3415 | 0.340 | 0.2331 | No |
| 47 | ATP1A3 | ATPase Na+/K+ transporting subunit alpha 3 [Source:HGNC Symbol;Acc:HGNC:801] | 3542 | 0.323 | 0.2295 | No |
| 48 | MPP5 | membrane palmitoylated protein 5 [Source:HGNC Symbol;Acc:HGNC:18669] | 3603 | 0.315 | 0.2300 | No |
| 49 | AKT3 | AKT serine/threonine kinase 3 [Source:HGNC Symbol;Acc:HGNC:393] | 4169 | 0.247 | 0.1969 | No |
| 50 | IRS1 | insulin receptor substrate 1 [Source:HGNC Symbol;Acc:HGNC:6125] | 4220 | 0.241 | 0.1970 | No |
| 51 | CNN2 | calponin 2 [Source:HGNC Symbol;Acc:HGNC:2156] | 4300 | 0.232 | 0.1951 | No |
| 52 | FBN1 | fibrillin 1 [Source:HGNC Symbol;Acc:HGNC:3603] | 4479 | 0.215 | 0.1866 | No |
| 53 | LDLRAP1 | low density lipoprotein receptor adaptor protein 1 [Source:HGNC Symbol;Acc:HGNC:18640] | 4522 | 0.211 | 0.1869 | No |
| 54 | CLDN6 | claudin 6 [Source:HGNC Symbol;Acc:HGNC:2048] | 4595 | 0.205 | 0.1851 | No |
| 55 | VCAN | versican [Source:HGNC Symbol;Acc:HGNC:2464] | 4697 | 0.197 | 0.1813 | No |
| 56 | CADM3 | cell adhesion molecule 3 [Source:HGNC Symbol;Acc:HGNC:17601] | 5022 | 0.166 | 0.1626 | No |
| 57 | ACTB | actin beta [Source:HGNC Symbol;Acc:HGNC:132] | 5029 | 0.165 | 0.1645 | No |
| 58 | CDK8 | cyclin dependent kinase 8 [Source:HGNC Symbol;Acc:HGNC:1779] | 5185 | 0.152 | 0.1566 | No |
| 59 | VASP | vasodilator stimulated phosphoprotein [Source:HGNC Symbol;Acc:HGNC:12652] | 5331 | 0.140 | 0.1492 | No |
| 60 | ARPC2 | actin related protein 2/3 complex subunit 2 [Source:HGNC Symbol;Acc:HGNC:705] | 5870 | 0.101 | 0.1159 | No |
| 61 | RASA1 | RAS p21 protein activator 1 [Source:HGNC Symbol;Acc:HGNC:9871] | 5873 | 0.101 | 0.1172 | No |
| 62 | RHOF | "ras homolog family member F, filopodia associated [Source:HGNC Symbol;Acc:HGNC:15703]" | 5961 | 0.095 | 0.1128 | No |
| 63 | CAP1 | cyclase associated actin cytoskeleton regulatory protein 1 [Source:HGNC Symbol;Acc:HGNC:20040] | 6005 | 0.091 | 0.1113 | No |
| 64 | TIAL1 | TIA1 cytotoxic granule associated RNA binding protein like 1 [Source:HGNC Symbol;Acc:HGNC:11804] | 6025 | 0.089 | 0.1113 | No |
| 65 | DLG1 | discs large MAGUK scaffold protein 1 [Source:HGNC Symbol;Acc:HGNC:2900] | 6093 | 0.083 | 0.1082 | No |
| 66 | TNFRSF11B | TNF receptor superfamily member 11b [Source:HGNC Symbol;Acc:HGNC:11909] | 6124 | 0.081 | 0.1074 | No |
| 67 | CDH4 | cadherin 4 [Source:HGNC Symbol;Acc:HGNC:1763] | 6187 | 0.078 | 0.1044 | No |
| 68 | VCAM1 | vascular cell adhesion molecule 1 [Source:HGNC Symbol;Acc:HGNC:12663] | 6218 | 0.075 | 0.1035 | No |
| 69 | NECTIN3 | nectin cell adhesion molecule 3 [Source:HGNC Symbol;Acc:HGNC:17664] | 6229 | 0.075 | 0.1039 | No |
| 70 | IKBKG | inhibitor of nuclear factor kappa B kinase regulatory subunit gamma [Source:HGNC Symbol;Acc:HGNC:5961] | 6343 | 0.067 | 0.0976 | No |
| 71 | MYH9 | myosin heavy chain 9 [Source:HGNC Symbol;Acc:HGNC:7579] | 6387 | 0.063 | 0.0957 | No |
| 72 | THY1 | Thy-1 cell surface antigen [Source:HGNC Symbol;Acc:HGNC:11801] | 6495 | 0.055 | 0.0895 | No |
| 73 | TSC1 | TSC complex subunit 1 [Source:HGNC Symbol;Acc:HGNC:12362] | 6594 | 0.049 | 0.0839 | No |
| 74 | CDH3 | cadherin 3 [Source:HGNC Symbol;Acc:HGNC:1762] | 6659 | 0.045 | 0.0804 | No |
| 75 | DMP1 | dentin matrix acidic phosphoprotein 1 [Source:HGNC Symbol;Acc:HGNC:2932] | 6694 | 0.043 | 0.0788 | No |
| 76 | B4GALT1 | "beta-1,4-galactosyltransferase 1 [Source:HGNC Symbol;Acc:HGNC:924]" | 6824 | 0.035 | 0.0709 | No |
| 77 | BMP1 | bone morphogenetic protein 1 [Source:HGNC Symbol;Acc:HGNC:1067] | 6929 | 0.027 | 0.0646 | No |
| 78 | NEXN | nexilin F-actin binding protein [Source:HGNC Symbol;Acc:HGNC:29557] | 7026 | 0.022 | 0.0587 | No |
| 79 | PIK3CB | "phosphatidylinositol-4,5-bisphosphate 3-kinase catalytic subunit beta [Source:HGNC Symbol;Acc:HGNC:8976]" | 7031 | 0.022 | 0.0587 | No |
| 80 | TAOK2 | TAO kinase 2 [Source:HGNC Symbol;Acc:HGNC:16835] | 7077 | 0.020 | 0.0561 | No |
| 81 | CD34 | CD34 molecule [Source:HGNC Symbol;Acc:HGNC:1662] | 7115 | 0.017 | 0.0540 | No |
| 82 | PTK2 | protein tyrosine kinase 2 [Source:HGNC Symbol;Acc:HGNC:9611] | 7196 | 0.012 | 0.0490 | No |
| 83 | VAV2 | vav guanine nucleotide exchange factor 2 [Source:HGNC Symbol;Acc:HGNC:12658] | 7575 | -0.008 | 0.0246 | No |
| 84 | MAPK14 | mitogen-activated protein kinase 14 [Source:HGNC Symbol;Acc:HGNC:6876] | 7662 | -0.011 | 0.0192 | No |
| 85 | CLDN18 | claudin 18 [Source:HGNC Symbol;Acc:HGNC:2039] | 7676 | -0.012 | 0.0186 | No |
| 86 | SRC | "SRC proto-oncogene, non-receptor tyrosine kinase [Source:HGNC Symbol;Acc:HGNC:11283]" | 7732 | -0.015 | 0.0152 | No |
| 87 | PFN1 | profilin 1 [Source:HGNC Symbol;Acc:HGNC:8881] | 7810 | -0.019 | 0.0105 | No |
| 88 | MSN | moesin [Source:HGNC Symbol;Acc:HGNC:7373] | 7875 | -0.022 | 0.0067 | No |
| 89 | NECTIN1 | nectin cell adhesion molecule 1 [Source:HGNC Symbol;Acc:HGNC:9706] | 8022 | -0.030 | -0.0023 | No |
| 90 | ZYX | zyxin [Source:HGNC Symbol;Acc:HGNC:13200] | 8151 | -0.038 | -0.0101 | No |
| 91 | SYMPK | symplekin [Source:HGNC Symbol;Acc:HGNC:22935] | 8176 | -0.039 | -0.0111 | No |
| 92 | TJP1 | tight junction protein 1 [Source:HGNC Symbol;Acc:HGNC:11827] | 8203 | -0.040 | -0.0122 | No |
| 93 | MAPK11 | mitogen-activated protein kinase 11 [Source:HGNC Symbol;Acc:HGNC:6873] | 8280 | -0.042 | -0.0165 | No |
| 94 | ACTN4 | actinin alpha 4 [Source:HGNC Symbol;Acc:HGNC:166] | 8441 | -0.052 | -0.0261 | No |
| 95 | STX4 | syntaxin 4 [Source:HGNC Symbol;Acc:HGNC:11439] | 8510 | -0.055 | -0.0297 | No |
| 96 | YWHAH | tyrosine 3-monooxygenase/tryptophan 5-monooxygenase activation protein eta [Source:HGNC Symbol;Acc:HGNC:12853] | 8556 | -0.058 | -0.0319 | No |
| 97 | ACTG1 | actin gamma 1 [Source:HGNC Symbol;Acc:HGNC:144] | 8595 | -0.060 | -0.0335 | No |
| 98 | COL17A1 | collagen type XVII alpha 1 chain [Source:HGNC Symbol;Acc:HGNC:2194] | 8615 | -0.062 | -0.0338 | No |
| 99 | CTNNA1 | catenin alpha 1 [Source:HGNC Symbol;Acc:HGNC:2509] | 8652 | -0.064 | -0.0353 | No |
| 100 | ADAM9 | ADAM metallopeptidase domain 9 [Source:HGNC Symbol;Acc:HGNC:216] | 8719 | -0.068 | -0.0386 | No |
| 101 | LIMA1 | LIM domain and actin binding 1 [Source:HGNC Symbol;Acc:HGNC:24636] | 8841 | -0.075 | -0.0454 | No |
| 102 | NLGN2 | neuroligin 2 [Source:HGNC Symbol;Acc:HGNC:14290] | 8963 | -0.080 | -0.0521 | No |
| 103 | EPB41L2 | erythrocyte membrane protein band 4.1 like 2 [Source:HGNC Symbol;Acc:HGNC:3379] | 9024 | -0.084 | -0.0548 | No |
| 104 | NRXN2 | neurexin 2 [Source:HGNC Symbol;Acc:HGNC:8009] | 9170 | -0.091 | -0.0629 | No |
| 105 | PARVA | parvin alpha [Source:HGNC Symbol;Acc:HGNC:14652] | 9213 | -0.094 | -0.0643 | No |
| 106 | ITGA3 | integrin subunit alpha 3 [Source:HGNC Symbol;Acc:HGNC:6139] | 9328 | -0.100 | -0.0703 | No |
| 107 | GTF2F1 | general transcription factor IIF subunit 1 [Source:HGNC Symbol;Acc:HGNC:4652] | 9442 | -0.107 | -0.0761 | No |
| 108 | EGFR | epidermal growth factor receptor [Source:HGNC Symbol;Acc:HGNC:3236] | 9513 | -0.111 | -0.0791 | No |
| 109 | MDK | midkine [Source:HGNC Symbol;Acc:HGNC:6972] | 9572 | -0.116 | -0.0812 | No |
| 110 | GNAI1 | G protein subunit alpha i1 [Source:HGNC Symbol;Acc:HGNC:4384] | 9635 | -0.121 | -0.0835 | No |
| 111 | CRAT | carnitine O-acetyltransferase [Source:HGNC Symbol;Acc:HGNC:2342] | 9657 | -0.122 | -0.0832 | No |
| 112 | NF1 | neurofibromin 1 [Source:HGNC Symbol;Acc:HGNC:7765] | 9703 | -0.125 | -0.0844 | No |
| 113 | ICAM5 | intercellular adhesion molecule 5 [Source:HGNC Symbol;Acc:HGNC:5348] | 9766 | -0.129 | -0.0866 | No |
| 114 | CTNND1 | catenin delta 1 [Source:HGNC Symbol;Acc:HGNC:2515] | 9869 | -0.134 | -0.0913 | No |
| 115 | GNAI2 | G protein subunit alpha i2 [Source:HGNC Symbol;Acc:HGNC:4385] | 9874 | -0.135 | -0.0897 | No |
| 116 | ACTN1 | actinin alpha 1 [Source:HGNC Symbol;Acc:HGNC:163] | 9983 | -0.141 | -0.0947 | No |
| 117 | RSU1 | Ras suppressor protein 1 [Source:HGNC Symbol;Acc:HGNC:10464] | 10062 | -0.145 | -0.0977 | No |
| 118 | DHX16 | DEAH-box helicase 16 [Source:HGNC Symbol;Acc:HGNC:2739] | 10076 | -0.146 | -0.0965 | No |
| 119 | SPEG | striated muscle enriched protein kinase [Source:HGNC Symbol;Acc:HGNC:16901] | 10130 | -0.149 | -0.0979 | No |
| 120 | EVL | Enah/Vasp-like [Source:HGNC Symbol;Acc:HGNC:20234] | 10175 | -0.152 | -0.0986 | No |
| 121 | HRAS | "HRas proto-oncogene, GTPase [Source:HGNC Symbol;Acc:HGNC:5173]" | 10331 | -0.161 | -0.1064 | No |
| 122 | PIK3R3 | phosphoinositide-3-kinase regulatory subunit 3 [Source:HGNC Symbol;Acc:HGNC:8981] | 10512 | -0.172 | -0.1156 | No |
| 123 | PBX2 | PBX homeobox 2 [Source:HGNC Symbol;Acc:HGNC:8633] | 10553 | -0.175 | -0.1158 | No |
| 124 | INPPL1 | inositol polyphosphate phosphatase like 1 [Source:HGNC Symbol;Acc:HGNC:6080] | 10729 | -0.184 | -0.1245 | No |
| 125 | WASL | WASP like actin nucleation promoting factor [Source:HGNC Symbol;Acc:HGNC:12735] | 10874 | -0.193 | -0.1312 | No |
| 126 | TSPAN4 | tetraspanin 4 [Source:HGNC Symbol;Acc:HGNC:11859] | 10880 | -0.193 | -0.1288 | No |
| 127 | ITGB1 | integrin subunit beta 1 [Source:HGNC Symbol;Acc:HGNC:6153] | 10916 | -0.196 | -0.1283 | No |
| 128 | AKT2 | AKT serine/threonine kinase 2 [Source:HGNC Symbol;Acc:HGNC:392] | 11023 | -0.201 | -0.1324 | No |
| 129 | PTEN | phosphatase and tensin homolog [Source:HGNC Symbol;Acc:HGNC:9588] | 11136 | -0.208 | -0.1367 | No |
| 130 | ADAM15 | ADAM metallopeptidase domain 15 [Source:HGNC Symbol;Acc:HGNC:193] | 11173 | -0.210 | -0.1361 | No |
| 131 | CX3CL1 | C-X3-C motif chemokine ligand 1 [Source:HGNC Symbol;Acc:HGNC:10647] | 11232 | -0.213 | -0.1369 | No |
| 132 | SKAP2 | src kinase associated phosphoprotein 2 [Source:HGNC Symbol;Acc:HGNC:15687] | 11269 | -0.215 | -0.1363 | No |
| 133 | HADH | hydroxyacyl-CoA dehydrogenase [Source:HGNC Symbol;Acc:HGNC:4799] | 11299 | -0.217 | -0.1351 | No |
| 134 | CLDN15 | claudin 15 [Source:HGNC Symbol;Acc:HGNC:2036] | 11622 | -0.236 | -0.1526 | No |
| 135 | ADRA1B | adrenoceptor alpha 1B [Source:HGNC Symbol;Acc:HGNC:278] | 11844 | -0.250 | -0.1634 | No |
| 136 | MAP3K20 | mitogen-activated protein kinase kinase kinase 20 [Source:HGNC Symbol;Acc:HGNC:17797] | 11882 | -0.253 | -0.1623 | No |
| 137 | MAP4K2 | mitogen-activated protein kinase kinase kinase kinase 2 [Source:HGNC Symbol;Acc:HGNC:6864] | 11965 | -0.258 | -0.1640 | No |
| 138 | RRAS | RAS related [Source:HGNC Symbol;Acc:HGNC:10447] | 12055 | -0.264 | -0.1661 | No |
| 139 | MPZL1 | myelin protein zero like 1 [Source:HGNC Symbol;Acc:HGNC:7226] | 12107 | -0.268 | -0.1657 | No |
| 140 | CLDN5 | claudin 5 [Source:HGNC Symbol;Acc:HGNC:2047] | 12149 | -0.270 | -0.1646 | No |
| 141 | ICAM2 | intercellular adhesion molecule 2 [Source:HGNC Symbol;Acc:HGNC:5345] | 12158 | -0.271 | -0.1613 | No |
| 142 | LAMC2 | laminin subunit gamma 2 [Source:HGNC Symbol;Acc:HGNC:6493] | 12173 | -0.272 | -0.1585 | No |
| 143 | CDH1 | cadherin 1 [Source:HGNC Symbol;Acc:HGNC:1748] | 12181 | -0.272 | -0.1551 | No |
| 144 | NF2 | neurofibromin 2 [Source:HGNC Symbol;Acc:HGNC:7773] | 12234 | -0.276 | -0.1547 | No |
| 145 | PECAM1 | platelet and endothelial cell adhesion molecule 1 [Source:HGNC Symbol;Acc:HGNC:8823] | 12443 | -0.292 | -0.1640 | No |
| 146 | FLNC | filamin C [Source:HGNC Symbol;Acc:HGNC:3756] | 12576 | -0.303 | -0.1684 | No |
| 147 | JAM3 | junctional adhesion molecule 3 [Source:HGNC Symbol;Acc:HGNC:15532] | 12587 | -0.303 | -0.1648 | No |
| 148 | LAYN | layilin [Source:HGNC Symbol;Acc:HGNC:29471] | 12619 | -0.305 | -0.1626 | No |
| 149 | COL16A1 | collagen type XVI alpha 1 chain [Source:HGNC Symbol;Acc:HGNC:2193] | 12641 | -0.307 | -0.1597 | No |
| 150 | PKD1 | "polycystin 1, transient receptor potential channel interacting [Source:HGNC Symbol;Acc:HGNC:9008]" | 12644 | -0.307 | -0.1555 | No |
| 151 | FSCN1 | fascin actin-bundling protein 1 [Source:HGNC Symbol;Acc:HGNC:11148] | 12688 | -0.311 | -0.1540 | No |
| 152 | BAIAP2 | BAR/IMD domain containing adaptor protein 2 [Source:HGNC Symbol;Acc:HGNC:947] | 12760 | -0.316 | -0.1542 | No |
| 153 | CD276 | CD276 molecule [Source:HGNC Symbol;Acc:HGNC:19137] | 12938 | -0.332 | -0.1610 | No |
| 154 | SHC1 | SHC adaptor protein 1 [Source:HGNC Symbol;Acc:HGNC:10840] | 12965 | -0.335 | -0.1580 | No |
| 155 | TUBG1 | tubulin gamma 1 [Source:HGNC Symbol;Acc:HGNC:12417] | 12998 | -0.338 | -0.1554 | No |
| 156 | PLCG1 | phospholipase C gamma 1 [Source:HGNC Symbol;Acc:HGNC:9065] | 13040 | -0.342 | -0.1533 | No |
| 157 | MVD | mevalonate diphosphate decarboxylase [Source:HGNC Symbol;Acc:HGNC:7529] | 13123 | -0.349 | -0.1538 | No |
| 158 | SGCE | sarcoglycan epsilon [Source:HGNC Symbol;Acc:HGNC:10808] | 13271 | -0.363 | -0.1582 | No |
| 159 | SORBS3 | sorbin and SH3 domain containing 3 [Source:HGNC Symbol;Acc:HGNC:30907] | 13408 | -0.380 | -0.1617 | No |
| 160 | GAMT | guanidinoacetate N-methyltransferase [Source:HGNC Symbol;Acc:HGNC:4136] | 13631 | -0.409 | -0.1704 | No |
| 161 | CADM2 | cell adhesion molecule 2 [Source:HGNC Symbol;Acc:HGNC:29849] | 13720 | -0.415 | -0.1703 | No |
| 162 | CALB2 | calbindin 2 [Source:HGNC Symbol;Acc:HGNC:1435] | 13749 | -0.415 | -0.1663 | No |
| 163 | INSIG1 | insulin induced gene 1 [Source:HGNC Symbol;Acc:HGNC:6083] | 13987 | -0.430 | -0.1757 | No |
| 164 | VWF | von Willebrand factor [Source:HGNC Symbol;Acc:HGNC:12726] | 14117 | -0.452 | -0.1777 | No |
| 165 | SLIT2 | slit guidance ligand 2 [Source:HGNC Symbol;Acc:HGNC:11086] | 14416 | -0.494 | -0.1901 | No |
| 166 | ACTC1 | actin alpha cardiac muscle 1 [Source:HGNC Symbol;Acc:HGNC:143] | 14458 | -0.505 | -0.1857 | No |
| 167 | TMEM8B | transmembrane protein 8B [Source:HGNC Symbol;Acc:HGNC:21427] | 14491 | -0.512 | -0.1807 | No |
| 168 | MYL9 | myosin light chain 9 [Source:HGNC Symbol;Acc:HGNC:15754] | 14619 | -0.537 | -0.1814 | No |
| 169 | MYH10 | myosin heavy chain 10 [Source:HGNC Symbol;Acc:HGNC:7568] | 14713 | -0.557 | -0.1797 | No |
| 170 | CDSN | corneodesmosin [Source:HGNC Symbol;Acc:HGNC:1802] | 14783 | -0.577 | -0.1762 | No |
| 171 | ACTG2 | "actin gamma 2, smooth muscle [Source:HGNC Symbol;Acc:HGNC:145]" | 14846 | -0.597 | -0.1719 | No |
| 172 | SHROOM2 | shroom family member 2 [Source:HGNC Symbol;Acc:HGNC:630] | 14850 | -0.599 | -0.1637 | No |
| 173 | EXOC4 | exocyst complex component 4 [Source:HGNC Symbol;Acc:HGNC:30389] | 14888 | -0.615 | -0.1576 | No |
| 174 | AMIGO1 | adhesion molecule with Ig like domain 1 [Source:HGNC Symbol;Acc:HGNC:20824] | 14919 | -0.627 | -0.1508 | No |
| 175 | CLDN4 | claudin 4 [Source:HGNC Symbol;Acc:HGNC:2046] | 15058 | -0.694 | -0.1501 | No |
| 176 | PCDH1 | protocadherin 1 [Source:HGNC Symbol;Acc:HGNC:8655] | 15112 | -0.727 | -0.1434 | No |
| 177 | ALOX15B | arachidonate 15-lipoxygenase type B [Source:HGNC Symbol;Acc:HGNC:434] | 15300 | -0.841 | -0.1438 | No |
| 178 | CNTN1 | contactin 1 [Source:HGNC Symbol;Acc:HGNC:2171] | 15346 | -0.858 | -0.1348 | No |
| 179 | THBS3 | thrombospondin 3 [Source:HGNC Symbol;Acc:HGNC:11787] | 15451 | -0.957 | -0.1282 | No |
| 180 | ITGB4 | integrin subunit beta 4 [Source:HGNC Symbol;Acc:HGNC:6158] | 15452 | -0.960 | -0.1149 | No |
| 181 | NECTIN2 | nectin cell adhesion molecule 2 [Source:HGNC Symbol;Acc:HGNC:9707] | 15471 | -0.987 | -0.1024 | No |
| 182 | JUP | junction plakoglobin [Source:HGNC Symbol;Acc:HGNC:6207] | 15477 | -1.001 | -0.0888 | No |
| 183 | NRTN | neurturin [Source:HGNC Symbol;Acc:HGNC:8007] | 15486 | -1.016 | -0.0752 | No |
| 184 | AMIGO2 | adhesion molecule with Ig like domain 2 [Source:HGNC Symbol;Acc:HGNC:24073] | 15505 | -1.040 | -0.0619 | No |
| 185 | CLDN9 | claudin 9 [Source:HGNC Symbol;Acc:HGNC:2051] | 15520 | -1.083 | -0.0478 | No |
| 186 | PARD6G | par-6 family cell polarity regulator gamma [Source:HGNC Symbol;Acc:HGNC:16076] | 15565 | -1.190 | -0.0341 | No |
| 187 | CLDN19 | claudin 19 [Source:HGNC Symbol;Acc:HGNC:2040] | 15620 | -1.422 | -0.0178 | No |
| 188 | ITGA10 | integrin subunit alpha 10 [Source:HGNC Symbol;Acc:HGNC:6135] | 15632 | -1.501 | 0.0023 | No |
Table: GSEA details [plain text format]

  

Fig 2: HALLMARK\_APICAL\_JUNCTION      
 Blue-Pink O' Gram in the Space of the Analyzed GeneSet

  

Fig 3: HALLMARK\_APICAL\_JUNCTION: Random ES distribution      
 Gene set null distribution of ES for **HALLMARK\_APICAL\_JUNCTION**
