# Supplementary material for: In vivo CRISPR screens reveal Serpinb9 and Adam2 as regulators of immune therapy response in lung cancer
Source: Nat Commun. 2023 May 31;14:3150. doi: 10.1038/s41467-023-38841-7 (PMC10232477; doi:10.1038/s41467-023-38841-7)
Supplement: Supplementary file 13 — Source Data [file 41467_2023_38841_MOESM13_ESM.zip › Figure 4E.zip!/my_analysis.Gsea.1640146340255_Hallmark/HALLMARK_INFLAMMATORY_RESPONSE.html]

Details for gene set HALLMARK\_INFLAMMATORY\_RESPONSE[GSEA]

|  || Dataset | December 21, 2021\_for GSEA run\_collapsed\_to\_symbols.GSEA\_B6\_NI.cls #CTLR\_NI\_versus\_ADAM2\_NI |
| Phenotype | GSEA\_B6\_NI.cls#CTLR\_NI\_versus\_ADAM2\_NI |
| Upregulated in class | CTLR\_NI |
| GeneSet | HALLMARK\_INFLAMMATORY\_RESPONSE |
| Enrichment Score (ES) | 0.5940148 |
| Normalized Enrichment Score (NES) | 1.6161464 |
| Nominal p-value | 0.0 |
| FDR q-value | 0.26889947 |
| FWER p-Value | 0.286 |
Table: GSEA Results Summary

  

Fig 1: Enrichment plot: HALLMARK\_INFLAMMATORY\_RESPONSE      
 Profile of the Running ES Score & Positions of GeneSet Members on the Rank Ordered List

  

| SYMBOL | TITLE | RANK IN GENE LIST | RANK METRIC SCORE | RUNNING ES | CORE ENRICHMENT || 1 | CXCL11 | C-X-C motif chemokine ligand 11 [Source:HGNC Symbol;Acc:HGNC:10638] | 2 | 3.216 | 0.0289 | Yes |
| 2 | CXCR6 | C-X-C motif chemokine receptor 6 [Source:HGNC Symbol;Acc:HGNC:16647] | 19 | 2.451 | 0.0500 | Yes |
| 3 | RTP4 | receptor transporter protein 4 [Source:HGNC Symbol;Acc:HGNC:23992] | 80 | 1.848 | 0.0628 | Yes |
| 4 | TNFSF10 | TNF superfamily member 10 [Source:HGNC Symbol;Acc:HGNC:11925] | 125 | 1.728 | 0.0755 | Yes |
| 5 | MEFV | "MEFV innate immuity regulator, pyrin [Source:HGNC Symbol;Acc:HGNC:6998]" | 126 | 1.727 | 0.0911 | Yes |
| 6 | IL2RB | interleukin 2 receptor subunit beta [Source:HGNC Symbol;Acc:HGNC:6009] | 138 | 1.679 | 0.1055 | Yes |
| 7 | CXCL10 | C-X-C motif chemokine ligand 10 [Source:HGNC Symbol;Acc:HGNC:10637] | 142 | 1.666 | 0.1204 | Yes |
| 8 | CCL5 | C-C motif chemokine ligand 5 [Source:HGNC Symbol;Acc:HGNC:10632] | 169 | 1.591 | 0.1330 | Yes |
| 9 | IL18R1 | interleukin 18 receptor 1 [Source:HGNC Symbol;Acc:HGNC:5988] | 179 | 1.535 | 0.1463 | Yes |
| 10 | SLC28A2 | solute carrier family 28 member 2 [Source:HGNC Symbol;Acc:HGNC:11002] | 182 | 1.532 | 0.1600 | Yes |
| 11 | BDKRB1 | bradykinin receptor B1 [Source:HGNC Symbol;Acc:HGNC:1029] | 190 | 1.511 | 0.1732 | Yes |
| 12 | CCL22 | C-C motif chemokine ligand 22 [Source:HGNC Symbol;Acc:HGNC:10621] | 196 | 1.500 | 0.1864 | Yes |
| 13 | SELL | selectin L [Source:HGNC Symbol;Acc:HGNC:10720] | 206 | 1.467 | 0.1991 | Yes |
| 14 | GPR132 | G protein-coupled receptor 132 [Source:HGNC Symbol;Acc:HGNC:17482] | 237 | 1.399 | 0.2097 | Yes |
| 15 | IL10 | interleukin 10 [Source:HGNC Symbol;Acc:HGNC:5962] | 246 | 1.386 | 0.2217 | Yes |
| 16 | PTAFR | platelet activating factor receptor [Source:HGNC Symbol;Acc:HGNC:9582] | 257 | 1.373 | 0.2335 | Yes |
| 17 | TLR3 | toll like receptor 3 [Source:HGNC Symbol;Acc:HGNC:11849] | 259 | 1.367 | 0.2458 | Yes |
| 18 | LCK | "LCK proto-oncogene, Src family tyrosine kinase [Source:HGNC Symbol;Acc:HGNC:6524]" | 286 | 1.331 | 0.2561 | Yes |
| 19 | IRF1 | interferon regulatory factor 1 [Source:HGNC Symbol;Acc:HGNC:6116] | 295 | 1.310 | 0.2674 | Yes |
| 20 | IL1B | interleukin 1 beta [Source:HGNC Symbol;Acc:HGNC:5992] | 303 | 1.298 | 0.2786 | Yes |
| 21 | BST2 | bone marrow stromal cell antigen 2 [Source:HGNC Symbol;Acc:HGNC:1119] | 306 | 1.293 | 0.2902 | Yes |
| 22 | CSF3R | colony stimulating factor 3 receptor [Source:HGNC Symbol;Acc:HGNC:2439] | 345 | 1.241 | 0.2989 | Yes |
| 23 | CCR7 | C-C motif chemokine receptor 7 [Source:HGNC Symbol;Acc:HGNC:1608] | 350 | 1.232 | 0.3098 | Yes |
| 24 | IRF7 | interferon regulatory factor 7 [Source:HGNC Symbol;Acc:HGNC:6122] | 359 | 1.219 | 0.3203 | Yes |
| 25 | IL6 | interleukin 6 [Source:HGNC Symbol;Acc:HGNC:6018] | 378 | 1.200 | 0.3299 | Yes |
| 26 | FFAR2 | free fatty acid receptor 2 [Source:HGNC Symbol;Acc:HGNC:4501] | 383 | 1.195 | 0.3404 | Yes |
| 27 | C3AR1 | complement C3a receptor 1 [Source:HGNC Symbol;Acc:HGNC:1319] | 387 | 1.191 | 0.3510 | Yes |
| 28 | NOD2 | nucleotide binding oligomerization domain containing 2 [Source:HGNC Symbol;Acc:HGNC:5331] | 410 | 1.161 | 0.3601 | Yes |
| 29 | CCRL2 | C-C motif chemokine receptor like 2 [Source:HGNC Symbol;Acc:HGNC:1612] | 427 | 1.145 | 0.3694 | Yes |
| 30 | EIF2AK2 | eukaryotic translation initiation factor 2 alpha kinase 2 [Source:HGNC Symbol;Acc:HGNC:9437] | 472 | 1.098 | 0.3764 | Yes |
| 31 | CXCL9 | C-X-C motif chemokine ligand 9 [Source:HGNC Symbol;Acc:HGNC:7098] | 473 | 1.098 | 0.3863 | Yes |
| 32 | CD69 | CD69 molecule [Source:HGNC Symbol;Acc:HGNC:1694] | 476 | 1.094 | 0.3961 | Yes |
| 33 | IL15RA | interleukin 15 receptor subunit alpha [Source:HGNC Symbol;Acc:HGNC:5978] | 494 | 1.080 | 0.4047 | Yes |
| 34 | IRAK2 | interleukin 1 receptor associated kinase 2 [Source:HGNC Symbol;Acc:HGNC:6113] | 533 | 1.036 | 0.4116 | Yes |
| 35 | IL1A | interleukin 1 alpha [Source:HGNC Symbol;Acc:HGNC:5991] | 540 | 1.032 | 0.4205 | Yes |
| 36 | SEMA4D | semaphorin 4D [Source:HGNC Symbol;Acc:HGNC:10732] | 559 | 1.020 | 0.4286 | Yes |
| 37 | NMI | N-myc and STAT interactor [Source:HGNC Symbol;Acc:HGNC:7854] | 579 | 1.008 | 0.4364 | Yes |
| 38 | HPN | hepsin [Source:HGNC Symbol;Acc:HGNC:5155] | 625 | 0.973 | 0.4423 | Yes |
| 39 | HAS2 | hyaluronan synthase 2 [Source:HGNC Symbol;Acc:HGNC:4819] | 676 | 0.948 | 0.4476 | Yes |
| 40 | ABCA1 | ATP binding cassette subfamily A member 1 [Source:HGNC Symbol;Acc:HGNC:29] | 717 | 0.923 | 0.4534 | Yes |
| 41 | CSF3 | colony stimulating factor 3 [Source:HGNC Symbol;Acc:HGNC:2438] | 720 | 0.922 | 0.4616 | Yes |
| 42 | LCP2 | lymphocyte cytosolic protein 2 [Source:HGNC Symbol;Acc:HGNC:6529] | 727 | 0.918 | 0.4695 | Yes |
| 43 | RASGRP1 | RAS guanyl releasing protein 1 [Source:HGNC Symbol;Acc:HGNC:9878] | 776 | 0.890 | 0.4744 | Yes |
| 44 | TNFSF15 | TNF superfamily member 15 [Source:HGNC Symbol;Acc:HGNC:11931] | 801 | 0.877 | 0.4807 | Yes |
| 45 | CYBB | cytochrome b-245 beta chain [Source:HGNC Symbol;Acc:HGNC:2578] | 806 | 0.875 | 0.4884 | Yes |
| 46 | IL7R | interleukin 7 receptor [Source:HGNC Symbol;Acc:HGNC:6024] | 816 | 0.869 | 0.4956 | Yes |
| 47 | TNFRSF1B | TNF receptor superfamily member 1B [Source:HGNC Symbol;Acc:HGNC:11917] | 845 | 0.864 | 0.5016 | Yes |
| 48 | EBI3 | Epstein-Barr virus induced 3 [Source:HGNC Symbol;Acc:HGNC:3129] | 852 | 0.862 | 0.5090 | Yes |
| 49 | TLR1 | toll like receptor 1 [Source:HGNC Symbol;Acc:HGNC:11847] | 873 | 0.850 | 0.5154 | Yes |
| 50 | NDP | norrin cystine knot growth factor NDP [Source:HGNC Symbol;Acc:HGNC:7678] | 890 | 0.845 | 0.5220 | Yes |
| 51 | MXD1 | MAX dimerization protein 1 [Source:HGNC Symbol;Acc:HGNC:6761] | 898 | 0.842 | 0.5291 | Yes |
| 52 | NLRP3 | NLR family pyrin domain containing 3 [Source:HGNC Symbol;Acc:HGNC:16400] | 899 | 0.841 | 0.5367 | Yes |
| 53 | CD70 | CD70 molecule [Source:HGNC Symbol;Acc:HGNC:11937] | 938 | 0.839 | 0.5418 | Yes |
| 54 | IL10RA | interleukin 10 receptor subunit alpha [Source:HGNC Symbol;Acc:HGNC:5964] | 1082 | 0.778 | 0.5396 | Yes |
| 55 | ICAM1 | intercellular adhesion molecule 1 [Source:HGNC Symbol;Acc:HGNC:5344] | 1112 | 0.767 | 0.5447 | Yes |
| 56 | CD48 | CD48 molecule [Source:HGNC Symbol;Acc:HGNC:1683] | 1137 | 0.756 | 0.5499 | Yes |
| 57 | NAMPT | nicotinamide phosphoribosyltransferase [Source:HGNC Symbol;Acc:HGNC:30092] | 1163 | 0.744 | 0.5550 | Yes |
| 58 | RGS16 | regulator of G protein signaling 16 [Source:HGNC Symbol;Acc:HGNC:9997] | 1189 | 0.735 | 0.5600 | Yes |
| 59 | SCARF1 | scavenger receptor class F member 1 [Source:HGNC Symbol;Acc:HGNC:16820] | 1196 | 0.733 | 0.5663 | Yes |
| 60 | PTGER2 | prostaglandin E receptor 2 [Source:HGNC Symbol;Acc:HGNC:9594] | 1267 | 0.707 | 0.5681 | Yes |
| 61 | CD40 | CD40 molecule [Source:HGNC Symbol;Acc:HGNC:11919] | 1287 | 0.700 | 0.5732 | Yes |
| 62 | SLAMF1 | signaling lymphocytic activation molecule family member 1 [Source:HGNC Symbol;Acc:HGNC:10903] | 1355 | 0.675 | 0.5750 | Yes |
| 63 | IL18RAP | interleukin 18 receptor accessory protein [Source:HGNC Symbol;Acc:HGNC:5989] | 1369 | 0.670 | 0.5802 | Yes |
| 64 | TAPBP | TAP binding protein [Source:HGNC Symbol;Acc:HGNC:11566] | 1384 | 0.665 | 0.5853 | Yes |
| 65 | MSR1 | macrophage scavenger receptor 1 [Source:HGNC Symbol;Acc:HGNC:7376] | 1419 | 0.654 | 0.5890 | Yes |
| 66 | ICOSLG | inducible T cell costimulator ligand [Source:HGNC Symbol;Acc:HGNC:17087] | 1433 | 0.651 | 0.5940 | Yes |
| 67 | IL18 | interleukin 18 [Source:HGNC Symbol;Acc:HGNC:5986] | 1622 | 0.588 | 0.5872 | No |
| 68 | ADORA2B | adenosine A2b receptor [Source:HGNC Symbol;Acc:HGNC:264] | 1789 | 0.546 | 0.5814 | No |
| 69 | SLC31A2 | solute carrier family 31 member 2 [Source:HGNC Symbol;Acc:HGNC:11017] | 1875 | 0.529 | 0.5807 | No |
| 70 | ADGRE1 | adhesion G protein-coupled receptor E1 [Source:HGNC Symbol;Acc:HGNC:3336] | 1891 | 0.526 | 0.5844 | No |
| 71 | P2RX7 | purinergic receptor P2X 7 [Source:HGNC Symbol;Acc:HGNC:8537] | 1990 | 0.505 | 0.5827 | No |
| 72 | MEP1A | meprin A subunit alpha [Source:HGNC Symbol;Acc:HGNC:7015] | 2019 | 0.503 | 0.5854 | No |
| 73 | LYN | "LYN proto-oncogene, Src family tyrosine kinase [Source:HGNC Symbol;Acc:HGNC:6735]" | 2037 | 0.501 | 0.5888 | No |
| 74 | IL12B | interleukin 12B [Source:HGNC Symbol;Acc:HGNC:5970] | 2102 | 0.491 | 0.5891 | No |
| 75 | SLC7A2 | solute carrier family 7 member 2 [Source:HGNC Symbol;Acc:HGNC:11060] | 2111 | 0.491 | 0.5930 | No |
| 76 | P2RX4 | purinergic receptor P2X 4 [Source:HGNC Symbol;Acc:HGNC:8535] | 2318 | 0.466 | 0.5839 | No |
| 77 | PIK3R5 | phosphoinositide-3-kinase regulatory subunit 5 [Source:HGNC Symbol;Acc:HGNC:30035] | 2331 | 0.464 | 0.5873 | No |
| 78 | KCNA3 | potassium voltage-gated channel subfamily A member 3 [Source:HGNC Symbol;Acc:HGNC:6221] | 2386 | 0.454 | 0.5879 | No |
| 79 | FPR1 | formyl peptide receptor 1 [Source:HGNC Symbol;Acc:HGNC:3826] | 2392 | 0.452 | 0.5917 | No |
| 80 | GCH1 | GTP cyclohydrolase 1 [Source:HGNC Symbol;Acc:HGNC:4193] | 2566 | 0.441 | 0.5845 | No |
| 81 | IFITM1 | interferon induced transmembrane protein 1 [Source:HGNC Symbol;Acc:HGNC:5412] | 2571 | 0.440 | 0.5882 | No |
| 82 | PTGER4 | prostaglandin E receptor 4 [Source:HGNC Symbol;Acc:HGNC:9596] | 2628 | 0.429 | 0.5885 | No |
| 83 | IL15 | interleukin 15 [Source:HGNC Symbol;Acc:HGNC:5977] | 2791 | 0.418 | 0.5818 | No |
| 84 | NMUR1 | neuromedin U receptor 1 [Source:HGNC Symbol;Acc:HGNC:4518] | 2792 | 0.417 | 0.5855 | No |
| 85 | CHST2 | carbohydrate sulfotransferase 2 [Source:HGNC Symbol;Acc:HGNC:1970] | 2824 | 0.412 | 0.5872 | No |
| 86 | KCNMB2 | potassium calcium-activated channel subfamily M regulatory beta subunit 2 [Source:HGNC Symbol;Acc:HGNC:6286] | 2882 | 0.411 | 0.5873 | No |
| 87 | NFKBIA | NFKB inhibitor alpha [Source:HGNC Symbol;Acc:HGNC:7797] | 2954 | 0.411 | 0.5864 | No |
| 88 | CCL7 | C-C motif chemokine ligand 7 [Source:HGNC Symbol;Acc:HGNC:10634] | 2997 | 0.404 | 0.5873 | No |
| 89 | MET | "MET proto-oncogene, receptor tyrosine kinase [Source:HGNC Symbol;Acc:HGNC:7029]" | 3190 | 0.374 | 0.5783 | No |
| 90 | IL4R | interleukin 4 receptor [Source:HGNC Symbol;Acc:HGNC:6015] | 3233 | 0.368 | 0.5789 | No |
| 91 | BTG2 | BTG anti-proliferation factor 2 [Source:HGNC Symbol;Acc:HGNC:1131] | 3319 | 0.354 | 0.5766 | No |
| 92 | INHBA | inhibin subunit beta A [Source:HGNC Symbol;Acc:HGNC:6066] | 3323 | 0.354 | 0.5796 | No |
| 93 | DCBLD2 | "discoidin, CUB and LCCL domain containing 2 [Source:HGNC Symbol;Acc:HGNC:24627]" | 3417 | 0.340 | 0.5767 | No |
| 94 | AQP9 | aquaporin 9 [Source:HGNC Symbol;Acc:HGNC:643] | 3493 | 0.329 | 0.5748 | No |
| 95 | IFNGR2 | interferon gamma receptor 2 [Source:HGNC Symbol;Acc:HGNC:5440] | 3530 | 0.323 | 0.5754 | No |
| 96 | CCL2 | C-C motif chemokine ligand 2 [Source:HGNC Symbol;Acc:HGNC:10618] | 3622 | 0.312 | 0.5723 | No |
| 97 | BEST1 | bestrophin 1 [Source:HGNC Symbol;Acc:HGNC:12703] | 3651 | 0.307 | 0.5733 | No |
| 98 | IFNAR1 | interferon alpha and beta receptor subunit 1 [Source:HGNC Symbol;Acc:HGNC:5432] | 3890 | 0.280 | 0.5604 | No |
| 99 | CMKLR1 | chemerin chemokine-like receptor 1 [Source:HGNC Symbol;Acc:HGNC:2121] | 3919 | 0.276 | 0.5611 | No |
| 100 | SLC31A1 | solute carrier family 31 member 1 [Source:HGNC Symbol;Acc:HGNC:11016] | 4120 | 0.252 | 0.5504 | No |
| 101 | HIF1A | hypoxia inducible factor 1 subunit alpha [Source:HGNC Symbol;Acc:HGNC:4910] | 4178 | 0.246 | 0.5490 | No |
| 102 | EREG | epiregulin [Source:HGNC Symbol;Acc:HGNC:3443] | 4321 | 0.231 | 0.5419 | No |
| 103 | PROK2 | prokineticin 2 [Source:HGNC Symbol;Acc:HGNC:18455] | 4332 | 0.229 | 0.5433 | No |
| 104 | ABI1 | abl interactor 1 [Source:HGNC Symbol;Acc:HGNC:11320] | 4361 | 0.226 | 0.5435 | No |
| 105 | OLR1 | oxidized low density lipoprotein receptor 1 [Source:HGNC Symbol;Acc:HGNC:8133] | 4464 | 0.217 | 0.5389 | No |
| 106 | SELE | selectin E [Source:HGNC Symbol;Acc:HGNC:10718] | 4465 | 0.217 | 0.5409 | No |
| 107 | RHOG | ras homolog family member G [Source:HGNC Symbol;Acc:HGNC:672] | 4535 | 0.210 | 0.5383 | No |
| 108 | ACVR1B | activin A receptor type 1B [Source:HGNC Symbol;Acc:HGNC:172] | 4621 | 0.203 | 0.5346 | No |
| 109 | CXCL6 | C-X-C motif chemokine ligand 6 [Source:HGNC Symbol;Acc:HGNC:10643] | 4655 | 0.200 | 0.5343 | No |
| 110 | PTPRE | protein tyrosine phosphatase receptor type E [Source:HGNC Symbol;Acc:HGNC:9669] | 4700 | 0.197 | 0.5333 | No |
| 111 | CCL17 | C-C motif chemokine ligand 17 [Source:HGNC Symbol;Acc:HGNC:10615] | 4736 | 0.193 | 0.5327 | No |
| 112 | OSM | oncostatin M [Source:HGNC Symbol;Acc:HGNC:8506] | 4752 | 0.192 | 0.5335 | No |
| 113 | ACVR2A | activin A receptor type 2A [Source:HGNC Symbol;Acc:HGNC:173] | 4777 | 0.190 | 0.5337 | No |
| 114 | SLC11A2 | solute carrier family 11 member 2 [Source:HGNC Symbol;Acc:HGNC:10908] | 4867 | 0.182 | 0.5296 | No |
| 115 | TIMP1 | TIMP metallopeptidase inhibitor 1 [Source:HGNC Symbol;Acc:HGNC:11820] | 4971 | 0.171 | 0.5244 | No |
| 116 | TPBG | trophoblast glycoprotein [Source:HGNC Symbol;Acc:HGNC:12004] | 5191 | 0.151 | 0.5117 | No |
| 117 | PLAUR | "plasminogen activator, urokinase receptor [Source:HGNC Symbol;Acc:HGNC:9053]" | 5260 | 0.146 | 0.5086 | No |
| 118 | MARCO | macrophage receptor with collagenous structure [Source:HGNC Symbol;Acc:HGNC:6895] | 5263 | 0.146 | 0.5098 | No |
| 119 | CD14 | CD14 molecule [Source:HGNC Symbol;Acc:HGNC:1628] | 5473 | 0.129 | 0.4974 | No |
| 120 | KLF6 | Kruppel like factor 6 [Source:HGNC Symbol;Acc:HGNC:2235] | 5555 | 0.123 | 0.4933 | No |
| 121 | GNA15 | G protein subunit alpha 15 [Source:HGNC Symbol;Acc:HGNC:4383] | 5582 | 0.121 | 0.4927 | No |
| 122 | PDE4B | phosphodiesterase 4B [Source:HGNC Symbol;Acc:HGNC:8781] | 5633 | 0.116 | 0.4905 | No |
| 123 | RIPK2 | receptor interacting serine/threonine kinase 2 [Source:HGNC Symbol;Acc:HGNC:10020] | 5692 | 0.113 | 0.4878 | No |
| 124 | ITGB3 | integrin subunit beta 3 [Source:HGNC Symbol;Acc:HGNC:6156] | 5695 | 0.113 | 0.4887 | No |
| 125 | GP1BA | glycoprotein Ib platelet subunit alpha [Source:HGNC Symbol;Acc:HGNC:4439] | 6050 | 0.087 | 0.4666 | No |
| 126 | ITGA5 | integrin subunit alpha 5 [Source:HGNC Symbol;Acc:HGNC:6141] | 6225 | 0.075 | 0.4560 | No |
| 127 | MYC | "MYC proto-oncogene, bHLH transcription factor [Source:HGNC Symbol;Acc:HGNC:7553]" | 6504 | 0.055 | 0.4386 | No |
| 128 | NFKB1 | nuclear factor kappa B subunit 1 [Source:HGNC Symbol;Acc:HGNC:7794] | 6748 | 0.040 | 0.4232 | No |
| 129 | ATP2B1 | ATPase plasma membrane Ca2+ transporting 1 [Source:HGNC Symbol;Acc:HGNC:814] | 6837 | 0.034 | 0.4179 | No |
| 130 | KCNJ2 | potassium inwardly rectifying channel subfamily J member 2 [Source:HGNC Symbol;Acc:HGNC:6263] | 6854 | 0.033 | 0.4171 | No |
| 131 | SGMS2 | sphingomyelin synthase 2 [Source:HGNC Symbol;Acc:HGNC:28395] | 7042 | 0.022 | 0.4052 | No |
| 132 | LY6E | lymphocyte antigen 6 family member E [Source:HGNC Symbol;Acc:HGNC:6727] | 7188 | 0.013 | 0.3960 | No |
| 133 | STAB1 | stabilin 1 [Source:HGNC Symbol;Acc:HGNC:18628] | 7202 | 0.012 | 0.3953 | No |
| 134 | RGS1 | regulator of G protein signaling 1 [Source:HGNC Symbol;Acc:HGNC:9991] | 7278 | 0.008 | 0.3905 | No |
| 135 | RNF144B | ring finger protein 144B [Source:HGNC Symbol;Acc:HGNC:21578] | 7303 | 0.006 | 0.3890 | No |
| 136 | PSEN1 | presenilin 1 [Source:HGNC Symbol;Acc:HGNC:9508] | 7304 | 0.006 | 0.3890 | No |
| 137 | LIF | LIF interleukin 6 family cytokine [Source:HGNC Symbol;Acc:HGNC:6596] | 7310 | 0.006 | 0.3888 | No |
| 138 | ATP2C1 | ATPase secretory pathway Ca2+ transporting 1 [Source:HGNC Symbol;Acc:HGNC:13211] | 7381 | 0.002 | 0.3843 | No |
| 139 | SRI | sorcin [Source:HGNC Symbol;Acc:HGNC:11292] | 7595 | -0.009 | 0.3706 | No |
| 140 | AHR | aryl hydrocarbon receptor [Source:HGNC Symbol;Acc:HGNC:348] | 7668 | -0.012 | 0.3660 | No |
| 141 | TNFRSF9 | TNF receptor superfamily member 9 [Source:HGNC Symbol;Acc:HGNC:11924] | 7797 | -0.019 | 0.3579 | No |
| 142 | CD55 | CD55 molecule (Cromer blood group) [Source:HGNC Symbol;Acc:HGNC:2665] | 8098 | -0.035 | 0.3389 | No |
| 143 | EMP3 | epithelial membrane protein 3 [Source:HGNC Symbol;Acc:HGNC:3335] | 8226 | -0.041 | 0.3310 | No |
| 144 | LTA | lymphotoxin alpha [Source:HGNC Symbol;Acc:HGNC:6709] | 8340 | -0.046 | 0.3242 | No |
| 145 | GNAI3 | G protein subunit alpha i3 [Source:HGNC Symbol;Acc:HGNC:4387] | 8386 | -0.048 | 0.3217 | No |
| 146 | PVR | PVR cell adhesion molecule [Source:HGNC Symbol;Acc:HGNC:9705] | 8624 | -0.062 | 0.3069 | No |
| 147 | CD82 | CD82 molecule [Source:HGNC Symbol;Acc:HGNC:6210] | 8645 | -0.063 | 0.3062 | No |
| 148 | HBEGF | heparin binding EGF like growth factor [Source:HGNC Symbol;Acc:HGNC:3059] | 8784 | -0.071 | 0.2979 | No |
| 149 | OSMR | oncostatin M receptor [Source:HGNC Symbol;Acc:HGNC:8507] | 8832 | -0.074 | 0.2956 | No |
| 150 | TLR2 | toll like receptor 2 [Source:HGNC Symbol;Acc:HGNC:11848] | 9404 | -0.105 | 0.2596 | No |
| 151 | RAF1 | "Raf-1 proto-oncogene, serine/threonine kinase [Source:HGNC Symbol;Acc:HGNC:9829]" | 9515 | -0.112 | 0.2535 | No |
| 152 | IL1R1 | interleukin 1 receptor type 1 [Source:HGNC Symbol;Acc:HGNC:5993] | 9556 | -0.115 | 0.2520 | No |
| 153 | CLEC5A | C-type lectin domain containing 5A [Source:HGNC Symbol;Acc:HGNC:2054] | 9610 | -0.119 | 0.2496 | No |
| 154 | MMP14 | matrix metallopeptidase 14 [Source:HGNC Symbol;Acc:HGNC:7160] | 9684 | -0.124 | 0.2460 | No |
| 155 | EDN1 | endothelin 1 [Source:HGNC Symbol;Acc:HGNC:3176] | 9964 | -0.140 | 0.2293 | No |
| 156 | SERPINE1 | serpin family E member 1 [Source:HGNC Symbol;Acc:HGNC:8583] | 10052 | -0.145 | 0.2249 | No |
| 157 | CCL24 | C-C motif chemokine ligand 24 [Source:HGNC Symbol;Acc:HGNC:10623] | 10380 | -0.165 | 0.2053 | No |
| 158 | TACR1 | tachykinin receptor 1 [Source:HGNC Symbol;Acc:HGNC:11526] | 10486 | -0.171 | 0.2001 | No |
| 159 | FZD5 | frizzled class receptor 5 [Source:HGNC Symbol;Acc:HGNC:4043] | 10504 | -0.172 | 0.2005 | No |
| 160 | F3 | "coagulation factor III, tissue factor [Source:HGNC Symbol;Acc:HGNC:3541]" | 10575 | -0.175 | 0.1976 | No |
| 161 | AXL | AXL receptor tyrosine kinase [Source:HGNC Symbol;Acc:HGNC:905] | 10819 | -0.189 | 0.1836 | No |
| 162 | GPR183 | G protein-coupled receptor 183 [Source:HGNC Symbol;Acc:HGNC:3128] | 10846 | -0.191 | 0.1836 | No |
| 163 | SLC7A1 | solute carrier family 7 member 1 [Source:HGNC Symbol;Acc:HGNC:11057] | 10918 | -0.196 | 0.1808 | No |
| 164 | ATP2A2 | ATPase sarcoplasmic/endoplasmic reticulum Ca2+ transporting 2 [Source:HGNC Symbol;Acc:HGNC:812] | 11020 | -0.201 | 0.1761 | No |
| 165 | SCN1B | sodium voltage-gated channel beta subunit 1 [Source:HGNC Symbol;Acc:HGNC:10586] | 11044 | -0.202 | 0.1764 | No |
| 166 | ADRM1 | adhesion regulating molecule 1 [Source:HGNC Symbol;Acc:HGNC:15759] | 11166 | -0.210 | 0.1705 | No |
| 167 | CX3CL1 | C-X3-C motif chemokine ligand 1 [Source:HGNC Symbol;Acc:HGNC:10647] | 11232 | -0.213 | 0.1682 | No |
| 168 | PCDH7 | protocadherin 7 [Source:HGNC Symbol;Acc:HGNC:8659] | 11395 | -0.224 | 0.1598 | No |
| 169 | KIF1B | kinesin family member 1B [Source:HGNC Symbol;Acc:HGNC:16636] | 11428 | -0.225 | 0.1597 | No |
| 170 | PDPN | podoplanin [Source:HGNC Symbol;Acc:HGNC:29602] | 11902 | -0.254 | 0.1315 | No |
| 171 | GABBR1 | gamma-aminobutyric acid type B receptor subunit 1 [Source:HGNC Symbol;Acc:HGNC:4070] | 12052 | -0.264 | 0.1242 | No |
| 172 | PTGIR | prostaglandin I2 receptor [Source:HGNC Symbol;Acc:HGNC:9602] | 12357 | -0.285 | 0.1072 | No |
| 173 | GPC3 | glypican 3 [Source:HGNC Symbol;Acc:HGNC:4451] | 12585 | -0.303 | 0.0952 | No |
| 174 | RELA | "RELA proto-oncogene, NF-kB subunit [Source:HGNC Symbol;Acc:HGNC:9955]" | 12655 | -0.308 | 0.0936 | No |
| 175 | HRH1 | histamine receptor H1 [Source:HGNC Symbol;Acc:HGNC:5182] | 13417 | -0.381 | 0.0478 | No |
| 176 | APLNR | apelin receptor [Source:HGNC Symbol;Acc:HGNC:339] | 13668 | -0.413 | 0.0354 | No |
| 177 | LDLR | low density lipoprotein receptor [Source:HGNC Symbol;Acc:HGNC:6547] | 14103 | -0.450 | 0.0114 | No |
| 178 | CALCRL | calcitonin receptor like receptor [Source:HGNC Symbol;Acc:HGNC:16709] | 14282 | -0.478 | 0.0042 | No |
| 179 | P2RY2 | purinergic receptor P2Y2 [Source:HGNC Symbol;Acc:HGNC:8541] | 14467 | -0.507 | -0.0031 | No |
| 180 | LAMP3 | lysosomal associated membrane protein 3 [Source:HGNC Symbol;Acc:HGNC:14582] | 14483 | -0.511 | 0.0006 | No |
| 181 | ITGB8 | integrin subunit beta 8 [Source:HGNC Symbol;Acc:HGNC:6163] | 14728 | -0.562 | -0.0101 | No |
| 182 | CSF1 | colony stimulating factor 1 [Source:HGNC Symbol;Acc:HGNC:2432] | 14746 | -0.566 | -0.0061 | No |
| 183 | SELENOS | selenoprotein S [Source:HGNC Symbol;Acc:HGNC:30396] | 14833 | -0.594 | -0.0063 | No |
| 184 | SLC4A4 | solute carrier family 4 member 4 [Source:HGNC Symbol;Acc:HGNC:11030] | 15182 | -0.768 | -0.0219 | No |
| 185 | LPAR1 | lysophosphatidic acid receptor 1 [Source:HGNC Symbol;Acc:HGNC:3166] | 15191 | -0.773 | -0.0154 | No |
| 186 | TNFAIP6 | TNF alpha induced protein 6 [Source:HGNC Symbol;Acc:HGNC:11898] | 15265 | -0.815 | -0.0128 | No |
| 187 | TNFSF9 | TNF superfamily member 9 [Source:HGNC Symbol;Acc:HGNC:11939] | 15266 | -0.818 | -0.0054 | No |
| 188 | ADM | adrenomedullin [Source:HGNC Symbol;Acc:HGNC:259] | 15283 | -0.829 | 0.0010 | No |
| 189 | SPHK1 | sphingosine kinase 1 [Source:HGNC Symbol;Acc:HGNC:11240] | 15316 | -0.845 | 0.0066 | No |
| 190 | SLC1A2 | solute carrier family 1 member 2 [Source:HGNC Symbol;Acc:HGNC:10940] | 15333 | -0.847 | 0.0132 | No |
| 191 | CDKN1A | cyclin dependent kinase inhibitor 1A [Source:HGNC Symbol;Acc:HGNC:1784] | 15428 | -0.929 | 0.0155 | No |
Table: GSEA details [plain text format]

  

Fig 2: HALLMARK\_INFLAMMATORY\_RESPONSE      
 Blue-Pink O' Gram in the Space of the Analyzed GeneSet

  

Fig 3: HALLMARK\_INFLAMMATORY\_RESPONSE: Random ES distribution      
 Gene set null distribution of ES for **HALLMARK\_INFLAMMATORY\_RESPONSE**
