# Supplementary material for: In vivo CRISPR screens reveal Serpinb9 and Adam2 as regulators of immune therapy response in lung cancer
Source: Nat Commun. 2023 May 31;14:3150. doi: 10.1038/s41467-023-38841-7 (PMC10232477; doi:10.1038/s41467-023-38841-7)
Supplement: Supplementary file 13 — Source Data [file 41467_2023_38841_MOESM13_ESM.zip › Figure 4E.zip!/my_analysis.Gsea.1640146340255_Hallmark/HALLMARK_GLYCOLYSIS.html]

Details for gene set HALLMARK\_GLYCOLYSIS[GSEA]

|  || Dataset | December 21, 2021\_for GSEA run\_collapsed\_to\_symbols.GSEA\_B6\_NI.cls #CTLR\_NI\_versus\_ADAM2\_NI |
| Phenotype | GSEA\_B6\_NI.cls#CTLR\_NI\_versus\_ADAM2\_NI |
| Upregulated in class | ADAM2\_NI |
| GeneSet | HALLMARK\_GLYCOLYSIS |
| Enrichment Score (ES) | -0.26752588 |
| Normalized Enrichment Score (NES) | -1.2209138 |
| Nominal p-value | 0.111561865 |
| FDR q-value | 1.0 |
| FWER p-Value | 1.0 |
Table: GSEA Results Summary

  

Fig 1: Enrichment plot: HALLMARK\_GLYCOLYSIS      
 Profile of the Running ES Score & Positions of GeneSet Members on the Rank Ordered List

  

| SYMBOL | TITLE | RANK IN GENE LIST | RANK METRIC SCORE | RUNNING ES | CORE ENRICHMENT || 1 | SDC3 | syndecan 3 [Source:HGNC Symbol;Acc:HGNC:10660] | 465 | 1.102 | -0.0117 | No |
| 2 | ANGPTL4 | angiopoietin like 4 [Source:HGNC Symbol;Acc:HGNC:16039] | 565 | 1.018 | -0.0012 | No |
| 3 | HS6ST2 | heparan sulfate 6-O-sulfotransferase 2 [Source:HGNC Symbol;Acc:HGNC:19133] | 627 | 0.972 | 0.0110 | No |
| 4 | GLRX | glutaredoxin [Source:HGNC Symbol;Acc:HGNC:4330] | 694 | 0.937 | 0.0223 | No |
| 5 | GYS2 | glycogen synthase 2 [Source:HGNC Symbol;Acc:HGNC:4707] | 758 | 0.892 | 0.0331 | No |
| 6 | ALDOB | "aldolase, fructose-bisphosphate B [Source:HGNC Symbol;Acc:HGNC:417]" | 827 | 0.864 | 0.0431 | No |
| 7 | GAPDHS | "glyceraldehyde-3-phosphate dehydrogenase, spermatogenic [Source:HGNC Symbol;Acc:HGNC:24864]" | 1244 | 0.717 | 0.0281 | No |
| 8 | LHPP | phospholysine phosphohistidine inorganic pyrophosphate phosphatase [Source:HGNC Symbol;Acc:HGNC:30042] | 1467 | 0.637 | 0.0244 | No |
| 9 | IL13RA1 | interleukin 13 receptor subunit alpha 1 [Source:HGNC Symbol;Acc:HGNC:5974] | 1511 | 0.623 | 0.0320 | No |
| 10 | DSC2 | desmocollin 2 [Source:HGNC Symbol;Acc:HGNC:3036] | 1570 | 0.605 | 0.0383 | No |
| 11 | B4GALT4 | "beta-1,4-galactosyltransferase 4 [Source:HGNC Symbol;Acc:HGNC:927]" | 1576 | 0.603 | 0.0480 | No |
| 12 | TGFBI | transforming growth factor beta induced [Source:HGNC Symbol;Acc:HGNC:11771] | 1655 | 0.577 | 0.0526 | No |
| 13 | AGRN | agrin [Source:HGNC Symbol;Acc:HGNC:329] | 1767 | 0.552 | 0.0546 | No |
| 14 | ADORA2B | adenosine A2b receptor [Source:HGNC Symbol;Acc:HGNC:264] | 1789 | 0.546 | 0.0623 | No |
| 15 | PYGL | glycogen phosphorylase L [Source:HGNC Symbol;Acc:HGNC:9725] | 1847 | 0.533 | 0.0675 | No |
| 16 | ARTN | artemin [Source:HGNC Symbol;Acc:HGNC:727] | 2251 | 0.476 | 0.0493 | No |
| 17 | ELF3 | E74 like ETS transcription factor 3 [Source:HGNC Symbol;Acc:HGNC:3318] | 2338 | 0.462 | 0.0515 | No |
| 18 | ISG20 | interferon stimulated exonuclease gene 20 [Source:HGNC Symbol;Acc:HGNC:6130] | 2346 | 0.460 | 0.0587 | No |
| 19 | KIF20A | kinesin family member 20A [Source:HGNC Symbol;Acc:HGNC:9787] | 2399 | 0.452 | 0.0628 | No |
| 20 | PFKFB1 | "6-phosphofructo-2-kinase/fructose-2,6-biphosphatase 1 [Source:HGNC Symbol;Acc:HGNC:8872]" | 2588 | 0.436 | 0.0579 | No |
| 21 | MIOX | myo-inositol oxygenase [Source:HGNC Symbol;Acc:HGNC:14522] | 2668 | 0.426 | 0.0599 | No |
| 22 | SOX9 | SRY-box transcription factor 9 [Source:HGNC Symbol;Acc:HGNC:11204] | 2780 | 0.419 | 0.0597 | No |
| 23 | ME2 | malic enzyme 2 [Source:HGNC Symbol;Acc:HGNC:6984] | 2821 | 0.413 | 0.0640 | No |
| 24 | CHST2 | carbohydrate sulfotransferase 2 [Source:HGNC Symbol;Acc:HGNC:1970] | 2824 | 0.412 | 0.0707 | No |
| 25 | LDHC | lactate dehydrogenase C [Source:HGNC Symbol;Acc:HGNC:6544] | 2845 | 0.411 | 0.0762 | No |
| 26 | GPC1 | glypican 1 [Source:HGNC Symbol;Acc:HGNC:4449] | 3143 | 0.382 | 0.0634 | No |
| 27 | MET | "MET proto-oncogene, receptor tyrosine kinase [Source:HGNC Symbol;Acc:HGNC:7029]" | 3190 | 0.374 | 0.0666 | No |
| 28 | CTH | cystathionine gamma-lyase [Source:HGNC Symbol;Acc:HGNC:2501] | 3305 | 0.356 | 0.0652 | No |
| 29 | NANP | N-acetylneuraminic acid phosphatase [Source:HGNC Symbol;Acc:HGNC:16140] | 3314 | 0.355 | 0.0706 | No |
| 30 | PMM2 | phosphomannomutase 2 [Source:HGNC Symbol;Acc:HGNC:9115] | 3615 | 0.313 | 0.0564 | No |
| 31 | DLD | dihydrolipoamide dehydrogenase [Source:HGNC Symbol;Acc:HGNC:2898] | 3738 | 0.298 | 0.0535 | No |
| 32 | DEPDC1 | DEP domain containing 1 [Source:HGNC Symbol;Acc:HGNC:22949] | 3792 | 0.292 | 0.0549 | No |
| 33 | SLC16A3 | solute carrier family 16 member 3 [Source:HGNC Symbol;Acc:HGNC:10924] | 3910 | 0.277 | 0.0519 | No |
| 34 | ECD | ecdysoneless cell cycle regulator [Source:HGNC Symbol;Acc:HGNC:17029] | 4101 | 0.254 | 0.0439 | No |
| 35 | RPE | ribulose-5-phosphate-3-epimerase [Source:HGNC Symbol;Acc:HGNC:10293] | 4216 | 0.242 | 0.0405 | No |
| 36 | CENPA | centromere protein A [Source:HGNC Symbol;Acc:HGNC:1851] | 4246 | 0.237 | 0.0426 | No |
| 37 | CYB5A | cytochrome b5 type A [Source:HGNC Symbol;Acc:HGNC:2570] | 4318 | 0.231 | 0.0419 | No |
| 38 | CASP6 | caspase 6 [Source:HGNC Symbol;Acc:HGNC:1507] | 4374 | 0.225 | 0.0421 | No |
| 39 | SLC35A3 | solute carrier family 35 member A3 [Source:HGNC Symbol;Acc:HGNC:11023] | 4484 | 0.215 | 0.0386 | No |
| 40 | PFKP | "phosphofructokinase, platelet [Source:HGNC Symbol;Acc:HGNC:8878]" | 4579 | 0.206 | 0.0359 | No |
| 41 | VCAN | versican [Source:HGNC Symbol;Acc:HGNC:2464] | 4697 | 0.197 | 0.0317 | No |
| 42 | RRAGD | Ras related GTP binding D [Source:HGNC Symbol;Acc:HGNC:19903] | 4762 | 0.192 | 0.0307 | No |
| 43 | NDST3 | N-deacetylase and N-sulfotransferase 3 [Source:HGNC Symbol;Acc:HGNC:7682] | 4771 | 0.191 | 0.0334 | No |
| 44 | GNPDA1 | glucosamine-6-phosphate deaminase 1 [Source:HGNC Symbol;Acc:HGNC:4417] | 4936 | 0.175 | 0.0257 | No |
| 45 | SDC1 | syndecan 1 [Source:HGNC Symbol;Acc:HGNC:10658] | 4954 | 0.172 | 0.0274 | No |
| 46 | ZNF292 | zinc finger protein 292 [Source:HGNC Symbol;Acc:HGNC:18410] | 4961 | 0.172 | 0.0299 | No |
| 47 | HMMR | hyaluronan mediated motility receptor [Source:HGNC Symbol;Acc:HGNC:5012] | 5069 | 0.161 | 0.0257 | No |
| 48 | TPBG | trophoblast glycoprotein [Source:HGNC Symbol;Acc:HGNC:12004] | 5191 | 0.151 | 0.0204 | No |
| 49 | NASP | nuclear autoantigenic sperm protein [Source:HGNC Symbol;Acc:HGNC:7644] | 5294 | 0.142 | 0.0162 | No |
| 50 | POLR3K | RNA polymerase III subunit K [Source:HGNC Symbol;Acc:HGNC:14121] | 5307 | 0.141 | 0.0177 | No |
| 51 | ALG1 | ALG1 chitobiosyldiphosphodolichol beta-mannosyltransferase [Source:HGNC Symbol;Acc:HGNC:18294] | 5488 | 0.128 | 0.0082 | No |
| 52 | RARS1 | arginyl-tRNA synthetase 1 [Source:HGNC Symbol;Acc:HGNC:9870] | 5716 | 0.111 | -0.0046 | No |
| 53 | PYGB | glycogen phosphorylase B [Source:HGNC Symbol;Acc:HGNC:9723] | 5858 | 0.102 | -0.0120 | No |
| 54 | GLCE | glucuronic acid epimerase [Source:HGNC Symbol;Acc:HGNC:17855] | 5914 | 0.098 | -0.0140 | No |
| 55 | AURKA | aurora kinase A [Source:HGNC Symbol;Acc:HGNC:11393] | 5931 | 0.097 | -0.0134 | No |
| 56 | ENO2 | enolase 2 [Source:HGNC Symbol;Acc:HGNC:3353] | 5991 | 0.092 | -0.0157 | No |
| 57 | KIF2A | kinesin family member 2A [Source:HGNC Symbol;Acc:HGNC:6318] | 5993 | 0.092 | -0.0142 | No |
| 58 | FUT8 | fucosyltransferase 8 [Source:HGNC Symbol;Acc:HGNC:4019] | 6077 | 0.085 | -0.0182 | No |
| 59 | UGP2 | UDP-glucose pyrophosphorylase 2 [Source:HGNC Symbol;Acc:HGNC:12527] | 6087 | 0.084 | -0.0174 | No |
| 60 | PKP2 | plakophilin 2 [Source:HGNC Symbol;Acc:HGNC:9024] | 6089 | 0.083 | -0.0160 | No |
| 61 | PPFIA4 | PTPRF interacting protein alpha 4 [Source:HGNC Symbol;Acc:HGNC:9248] | 6155 | 0.079 | -0.0189 | No |
| 62 | DCN | decorin [Source:HGNC Symbol;Acc:HGNC:2705] | 6238 | 0.074 | -0.0230 | No |
| 63 | CDK1 | cyclin dependent kinase 1 [Source:HGNC Symbol;Acc:HGNC:1722] | 6401 | 0.062 | -0.0324 | No |
| 64 | ANG | angiogenin [Source:HGNC Symbol;Acc:HGNC:483] | 6415 | 0.061 | -0.0322 | No |
| 65 | ALDH9A1 | aldehyde dehydrogenase 9 family member A1 [Source:HGNC Symbol;Acc:HGNC:412] | 6489 | 0.056 | -0.0360 | No |
| 66 | MPI | mannose phosphate isomerase [Source:HGNC Symbol;Acc:HGNC:7216] | 6540 | 0.053 | -0.0384 | No |
| 67 | TXN | thioredoxin [Source:HGNC Symbol;Acc:HGNC:12435] | 6549 | 0.052 | -0.0380 | No |
| 68 | MERTK | "MER proto-oncogene, tyrosine kinase [Source:HGNC Symbol;Acc:HGNC:7027]" | 6672 | 0.044 | -0.0452 | No |
| 69 | B4GALT1 | "beta-1,4-galactosyltransferase 1 [Source:HGNC Symbol;Acc:HGNC:924]" | 6824 | 0.035 | -0.0544 | No |
| 70 | RBCK1 | RANBP2-type and C3HC4-type zinc finger containing 1 [Source:HGNC Symbol;Acc:HGNC:15864] | 6883 | 0.030 | -0.0576 | No |
| 71 | ME1 | malic enzyme 1 [Source:HGNC Symbol;Acc:HGNC:6983] | 6909 | 0.029 | -0.0587 | No |
| 72 | SRD5A3 | steroid 5 alpha-reductase 3 [Source:HGNC Symbol;Acc:HGNC:25812] | 7046 | 0.021 | -0.0672 | No |
| 73 | VLDLR | very low density lipoprotein receptor [Source:HGNC Symbol;Acc:HGNC:12698] | 7123 | 0.017 | -0.0718 | No |
| 74 | ERO1A | endoplasmic reticulum oxidoreductase 1 alpha [Source:HGNC Symbol;Acc:HGNC:13280] | 7181 | 0.013 | -0.0753 | No |
| 75 | GFUS | GDP-L-fucose synthase [Source:HGNC Symbol;Acc:HGNC:12390] | 7233 | 0.010 | -0.0784 | No |
| 76 | PLOD2 | "procollagen-lysine,2-oxoglutarate 5-dioxygenase 2 [Source:HGNC Symbol;Acc:HGNC:9082]" | 7291 | 0.007 | -0.0820 | No |
| 77 | ANKZF1 | ankyrin repeat and zinc finger peptidyl tRNA hydrolase 1 [Source:HGNC Symbol;Acc:HGNC:25527] | 7410 | 0.000 | -0.0896 | No |
| 78 | PGAM2 | phosphoglycerate mutase 2 [Source:HGNC Symbol;Acc:HGNC:8889] | 7413 | 0.000 | -0.0897 | No |
| 79 | SLC37A4 | solute carrier family 37 member 4 [Source:HGNC Symbol;Acc:HGNC:4061] | 7567 | -0.008 | -0.0995 | No |
| 80 | HOMER1 | homer scaffold protein 1 [Source:HGNC Symbol;Acc:HGNC:17512] | 7638 | -0.010 | -0.1038 | No |
| 81 | B4GALT7 | "beta-1,4-galactosyltransferase 7 [Source:HGNC Symbol;Acc:HGNC:930]" | 7649 | -0.011 | -0.1043 | No |
| 82 | VEGFA | vascular endothelial growth factor A [Source:HGNC Symbol;Acc:HGNC:12680] | 7671 | -0.012 | -0.1055 | No |
| 83 | SLC25A13 | solute carrier family 25 member 13 [Source:HGNC Symbol;Acc:HGNC:10983] | 7948 | -0.026 | -0.1229 | No |
| 84 | IDH1 | isocitrate dehydrogenase (NADP(+)) 1 [Source:HGNC Symbol;Acc:HGNC:5382] | 8014 | -0.030 | -0.1266 | No |
| 85 | GUSB | glucuronidase beta [Source:HGNC Symbol;Acc:HGNC:4696] | 8096 | -0.035 | -0.1312 | No |
| 86 | B3GNT3 | "UDP-GlcNAc:betaGal beta-1,3-N-acetylglucosaminyltransferase 3 [Source:HGNC Symbol;Acc:HGNC:13528]" | 8101 | -0.035 | -0.1309 | No |
| 87 | ARPP19 | cAMP regulated phosphoprotein 19 [Source:HGNC Symbol;Acc:HGNC:16967] | 8116 | -0.036 | -0.1312 | No |
| 88 | MED24 | mediator complex subunit 24 [Source:HGNC Symbol;Acc:HGNC:22963] | 8334 | -0.045 | -0.1445 | No |
| 89 | FKBP4 | FKBP prolyl isomerase 4 [Source:HGNC Symbol;Acc:HGNC:3720] | 8505 | -0.055 | -0.1545 | No |
| 90 | CLN6 | CLN6 transmembrane ER protein [Source:HGNC Symbol;Acc:HGNC:2077] | 8562 | -0.058 | -0.1572 | No |
| 91 | AGL | "amylo-alpha-1, 6-glucosidase, 4-alpha-glucanotransferase [Source:HGNC Symbol;Acc:HGNC:321]" | 8757 | -0.070 | -0.1686 | No |
| 92 | PGM2 | phosphoglucomutase 2 [Source:HGNC Symbol;Acc:HGNC:8906] | 8792 | -0.072 | -0.1696 | No |
| 93 | GFPT1 | glutamine--fructose-6-phosphate transaminase 1 [Source:HGNC Symbol;Acc:HGNC:4241] | 8924 | -0.078 | -0.1767 | No |
| 94 | GMPPA | GDP-mannose pyrophosphorylase A [Source:HGNC Symbol;Acc:HGNC:22923] | 8983 | -0.082 | -0.1791 | No |
| 95 | IDUA | alpha-L-iduronidase [Source:HGNC Symbol;Acc:HGNC:5391] | 9056 | -0.085 | -0.1824 | No |
| 96 | COPB2 | COPI coat complex subunit beta 2 [Source:HGNC Symbol;Acc:HGNC:2232] | 9081 | -0.087 | -0.1825 | No |
| 97 | SDHC | succinate dehydrogenase complex subunit C [Source:HGNC Symbol;Acc:HGNC:10682] | 9365 | -0.102 | -0.1991 | No |
| 98 | BPNT1 | "3'(2'), 5'-bisphosphate nucleotidase 1 [Source:HGNC Symbol;Acc:HGNC:1096]" | 9416 | -0.105 | -0.2005 | No |
| 99 | STC2 | stanniocalcin 2 [Source:HGNC Symbol;Acc:HGNC:11374] | 9474 | -0.108 | -0.2024 | No |
| 100 | EGFR | epidermal growth factor receptor [Source:HGNC Symbol;Acc:HGNC:3236] | 9513 | -0.111 | -0.2030 | No |
| 101 | PPP2CB | protein phosphatase 2 catalytic subunit beta [Source:HGNC Symbol;Acc:HGNC:9300] | 9637 | -0.121 | -0.2090 | No |
| 102 | AK3 | adenylate kinase 3 [Source:HGNC Symbol;Acc:HGNC:17376] | 9663 | -0.122 | -0.2085 | No |
| 103 | CXCR4 | C-X-C motif chemokine receptor 4 [Source:HGNC Symbol;Acc:HGNC:2561] | 9672 | -0.123 | -0.2070 | No |
| 104 | LCT | lactase [Source:HGNC Symbol;Acc:HGNC:6530] | 9723 | -0.126 | -0.2082 | No |
| 105 | GCLC | glutamate-cysteine ligase catalytic subunit [Source:HGNC Symbol;Acc:HGNC:4311] | 9762 | -0.129 | -0.2085 | No |
| 106 | NOL3 | nucleolar protein 3 [Source:HGNC Symbol;Acc:HGNC:7869] | 9785 | -0.130 | -0.2077 | No |
| 107 | GMPPB | GDP-mannose pyrophosphorylase B [Source:HGNC Symbol;Acc:HGNC:22932] | 9958 | -0.140 | -0.2165 | No |
| 108 | HDLBP | high density lipoprotein binding protein [Source:HGNC Symbol;Acc:HGNC:4857] | 10016 | -0.143 | -0.2178 | No |
| 109 | ALDH7A1 | aldehyde dehydrogenase 7 family member A1 [Source:HGNC Symbol;Acc:HGNC:877] | 10297 | -0.159 | -0.2333 | No |
| 110 | PSMC4 | "proteasome 26S subunit, ATPase 4 [Source:HGNC Symbol;Acc:HGNC:9551]" | 10382 | -0.165 | -0.2360 | No |
| 111 | TPI1 | triosephosphate isomerase 1 [Source:HGNC Symbol;Acc:HGNC:12009] | 10457 | -0.169 | -0.2379 | No |
| 112 | CD44 | CD44 molecule (Indian blood group) [Source:HGNC Symbol;Acc:HGNC:1681] | 10617 | -0.178 | -0.2453 | No |
| 113 | G6PD | glucose-6-phosphate dehydrogenase [Source:HGNC Symbol;Acc:HGNC:4057] | 10710 | -0.183 | -0.2482 | No |
| 114 | SOD1 | superoxide dismutase 1 [Source:HGNC Symbol;Acc:HGNC:11179] | 10977 | -0.199 | -0.2620 | No |
| 115 | COG2 | component of oligomeric golgi complex 2 [Source:HGNC Symbol;Acc:HGNC:6546] | 11063 | -0.204 | -0.2641 | Yes |
| 116 | NDUFV3 | NADH:ubiquinone oxidoreductase subunit V3 [Source:HGNC Symbol;Acc:HGNC:7719] | 11075 | -0.204 | -0.2615 | Yes |
| 117 | LHX9 | LIM homeobox 9 [Source:HGNC Symbol;Acc:HGNC:14222] | 11086 | -0.205 | -0.2587 | Yes |
| 118 | CHPF | chondroitin polymerizing factor [Source:HGNC Symbol;Acc:HGNC:24291] | 11184 | -0.211 | -0.2615 | Yes |
| 119 | GALK2 | galactokinase 2 [Source:HGNC Symbol;Acc:HGNC:4119] | 11240 | -0.214 | -0.2615 | Yes |
| 120 | TPST1 | tyrosylprotein sulfotransferase 1 [Source:HGNC Symbol;Acc:HGNC:12020] | 11270 | -0.215 | -0.2598 | Yes |
| 121 | MDH1 | malate dehydrogenase 1 [Source:HGNC Symbol;Acc:HGNC:6970] | 11276 | -0.216 | -0.2565 | Yes |
| 122 | KDELR3 | KDEL endoplasmic reticulum protein retention receptor 3 [Source:HGNC Symbol;Acc:HGNC:6306] | 11322 | -0.219 | -0.2558 | Yes |
| 123 | PHKA2 | phosphorylase kinase regulatory subunit alpha 2 [Source:HGNC Symbol;Acc:HGNC:8926] | 11398 | -0.224 | -0.2569 | Yes |
| 124 | TKTL1 | transketolase like 1 [Source:HGNC Symbol;Acc:HGNC:11835] | 11399 | -0.224 | -0.2532 | Yes |
| 125 | HK2 | hexokinase 2 [Source:HGNC Symbol;Acc:HGNC:4923] | 11425 | -0.225 | -0.2510 | Yes |
| 126 | FBP2 | fructose-bisphosphatase 2 [Source:HGNC Symbol;Acc:HGNC:3607] | 11469 | -0.228 | -0.2500 | Yes |
| 127 | CHST6 | carbohydrate sulfotransferase 6 [Source:HGNC Symbol;Acc:HGNC:6938] | 11475 | -0.228 | -0.2466 | Yes |
| 128 | EXT1 | exostosin glycosyltransferase 1 [Source:HGNC Symbol;Acc:HGNC:3512] | 11477 | -0.228 | -0.2428 | Yes |
| 129 | QSOX1 | quiescin sulfhydryl oxidase 1 [Source:HGNC Symbol;Acc:HGNC:9756] | 11515 | -0.230 | -0.2414 | Yes |
| 130 | TALDO1 | transaldolase 1 [Source:HGNC Symbol;Acc:HGNC:11559] | 11575 | -0.234 | -0.2413 | Yes |
| 131 | MIF | macrophage migration inhibitory factor [Source:HGNC Symbol;Acc:HGNC:7097] | 11705 | -0.241 | -0.2456 | Yes |
| 132 | AKR1A1 | aldo-keto reductase family 1 member A1 [Source:HGNC Symbol;Acc:HGNC:380] | 11783 | -0.247 | -0.2465 | Yes |
| 133 | CHPF2 | chondroitin polymerizing factor 2 [Source:HGNC Symbol;Acc:HGNC:29270] | 11850 | -0.250 | -0.2466 | Yes |
| 134 | GNE | glucosamine (UDP-N-acetyl)-2-epimerase/N-acetylmannosamine kinase [Source:HGNC Symbol;Acc:HGNC:23657] | 12008 | -0.261 | -0.2524 | Yes |
| 135 | PAXIP1 | PAX interacting protein 1 [Source:HGNC Symbol;Acc:HGNC:8624] | 12071 | -0.265 | -0.2520 | Yes |
| 136 | XYLT2 | xylosyltransferase 2 [Source:HGNC Symbol;Acc:HGNC:15517] | 12078 | -0.266 | -0.2480 | Yes |
| 137 | AK4 | adenylate kinase 4 [Source:HGNC Symbol;Acc:HGNC:363] | 12258 | -0.278 | -0.2549 | Yes |
| 138 | PRPS1 | phosphoribosyl pyrophosphate synthetase 1 [Source:HGNC Symbol;Acc:HGNC:9462] | 12273 | -0.279 | -0.2512 | Yes |
| 139 | EFNA3 | ephrin A3 [Source:HGNC Symbol;Acc:HGNC:3223] | 12276 | -0.279 | -0.2467 | Yes |
| 140 | GALE | UDP-galactose-4-epimerase [Source:HGNC Symbol;Acc:HGNC:4116] | 12319 | -0.282 | -0.2447 | Yes |
| 141 | MDH2 | malate dehydrogenase 2 [Source:HGNC Symbol;Acc:HGNC:6971] | 12367 | -0.286 | -0.2430 | Yes |
| 142 | CITED2 | Cbp/p300 interacting transactivator with Glu/Asp rich carboxy-terminal domain 2 [Source:HGNC Symbol;Acc:HGNC:1987] | 12508 | -0.296 | -0.2471 | Yes |
| 143 | SAP30 | Sin3A associated protein 30 [Source:HGNC Symbol;Acc:HGNC:10532] | 12567 | -0.302 | -0.2458 | Yes |
| 144 | GPC3 | glypican 3 [Source:HGNC Symbol;Acc:HGNC:4451] | 12585 | -0.303 | -0.2419 | Yes |
| 145 | HSPA5 | heat shock protein family A (Hsp70) member 5 [Source:HGNC Symbol;Acc:HGNC:5238] | 12586 | -0.303 | -0.2369 | Yes |
| 146 | ALDOA | "aldolase, fructose-bisphosphate A [Source:HGNC Symbol;Acc:HGNC:414]" | 12634 | -0.306 | -0.2348 | Yes |
| 147 | PLOD1 | "procollagen-lysine,2-oxoglutarate 5-dioxygenase 1 [Source:HGNC Symbol;Acc:HGNC:9081]" | 12706 | -0.312 | -0.2342 | Yes |
| 148 | PPIA | peptidylprolyl isomerase A [Source:HGNC Symbol;Acc:HGNC:9253] | 12733 | -0.314 | -0.2307 | Yes |
| 149 | PDK3 | pyruvate dehydrogenase kinase 3 [Source:HGNC Symbol;Acc:HGNC:8811] | 12734 | -0.314 | -0.2255 | Yes |
| 150 | PKM | pyruvate kinase M1/2 [Source:HGNC Symbol;Acc:HGNC:9021] | 12770 | -0.317 | -0.2224 | Yes |
| 151 | FAM162A | family with sequence similarity 162 member A [Source:HGNC Symbol;Acc:HGNC:17865] | 12830 | -0.321 | -0.2209 | Yes |
| 152 | IER3 | immediate early response 3 [Source:HGNC Symbol;Acc:HGNC:5392] | 12833 | -0.322 | -0.2157 | Yes |
| 153 | GOT1 | glutamic-oxaloacetic transaminase 1 [Source:HGNC Symbol;Acc:HGNC:4432] | 12864 | -0.325 | -0.2122 | Yes |
| 154 | GOT2 | glutamic-oxaloacetic transaminase 2 [Source:HGNC Symbol;Acc:HGNC:4433] | 12903 | -0.328 | -0.2092 | Yes |
| 155 | B4GALT2 | "beta-1,4-galactosyltransferase 2 [Source:HGNC Symbol;Acc:HGNC:925]" | 12912 | -0.329 | -0.2043 | Yes |
| 156 | CAPN5 | calpain 5 [Source:HGNC Symbol;Acc:HGNC:1482] | 12933 | -0.332 | -0.2000 | Yes |
| 157 | HAX1 | HCLS1 associated protein X-1 [Source:HGNC Symbol;Acc:HGNC:16915] | 13055 | -0.343 | -0.2021 | Yes |
| 158 | ENO1 | enolase 1 [Source:HGNC Symbol;Acc:HGNC:3350] | 13088 | -0.346 | -0.1985 | Yes |
| 159 | SPAG4 | sperm associated antigen 4 [Source:HGNC Symbol;Acc:HGNC:11214] | 13138 | -0.350 | -0.1958 | Yes |
| 160 | LDHA | lactate dehydrogenase A [Source:HGNC Symbol;Acc:HGNC:6535] | 13197 | -0.356 | -0.1936 | Yes |
| 161 | ABCB6 | ATP binding cassette subfamily B member 6 (Langereis blood group) [Source:HGNC Symbol;Acc:HGNC:47] | 13232 | -0.359 | -0.1899 | Yes |
| 162 | P4HA1 | prolyl 4-hydroxylase subunit alpha 1 [Source:HGNC Symbol;Acc:HGNC:8546] | 13262 | -0.362 | -0.1857 | Yes |
| 163 | BIK | BCL2 interacting killer [Source:HGNC Symbol;Acc:HGNC:1051] | 13352 | -0.373 | -0.1853 | Yes |
| 164 | PGLS | 6-phosphogluconolactonase [Source:HGNC Symbol;Acc:HGNC:8903] | 13415 | -0.380 | -0.1829 | Yes |
| 165 | NSDHL | NAD(P) dependent steroid dehydrogenase-like [Source:HGNC Symbol;Acc:HGNC:13398] | 13437 | -0.384 | -0.1779 | Yes |
| 166 | P4HA2 | prolyl 4-hydroxylase subunit alpha 2 [Source:HGNC Symbol;Acc:HGNC:8547] | 13502 | -0.392 | -0.1755 | Yes |
| 167 | STMN1 | stathmin 1 [Source:HGNC Symbol;Acc:HGNC:6510] | 13782 | -0.416 | -0.1866 | Yes |
| 168 | GYS1 | glycogen synthase 1 [Source:HGNC Symbol;Acc:HGNC:4706] | 13783 | -0.416 | -0.1797 | Yes |
| 169 | HS2ST1 | heparan sulfate 2-O-sulfotransferase 1 [Source:HGNC Symbol;Acc:HGNC:5193] | 13811 | -0.420 | -0.1745 | Yes |
| 170 | EXT2 | exostosin glycosyltransferase 2 [Source:HGNC Symbol;Acc:HGNC:3513] | 13870 | -0.426 | -0.1711 | Yes |
| 171 | PAM | peptidylglycine alpha-amidating monooxygenase [Source:HGNC Symbol;Acc:HGNC:8596] | 13872 | -0.427 | -0.1641 | Yes |
| 172 | PGK1 | phosphoglycerate kinase 1 [Source:HGNC Symbol;Acc:HGNC:8896] | 14157 | -0.458 | -0.1748 | Yes |
| 173 | PGAM1 | phosphoglycerate mutase 1 [Source:HGNC Symbol;Acc:HGNC:8888] | 14332 | -0.484 | -0.1780 | Yes |
| 174 | COL5A1 | collagen type V alpha 1 chain [Source:HGNC Symbol;Acc:HGNC:2209] | 14349 | -0.486 | -0.1710 | Yes |
| 175 | IRS2 | insulin receptor substrate 2 [Source:HGNC Symbol;Acc:HGNC:6126] | 14481 | -0.511 | -0.1709 | Yes |
| 176 | GALK1 | galactokinase 1 [Source:HGNC Symbol;Acc:HGNC:4118] | 14488 | -0.512 | -0.1628 | Yes |
| 177 | PC | pyruvate carboxylase [Source:HGNC Symbol;Acc:HGNC:8636] | 14499 | -0.513 | -0.1549 | Yes |
| 178 | SLC25A10 | solute carrier family 25 member 10 [Source:HGNC Symbol;Acc:HGNC:10980] | 14617 | -0.536 | -0.1536 | Yes |
| 179 | SDC2 | syndecan 2 [Source:HGNC Symbol;Acc:HGNC:10659] | 14631 | -0.539 | -0.1455 | Yes |
| 180 | B3GALT6 | "beta-1,3-galactosyltransferase 6 [Source:HGNC Symbol;Acc:HGNC:17978]" | 14726 | -0.561 | -0.1422 | Yes |
| 181 | MXI1 | "MAX interactor 1, dimerization protein [Source:HGNC Symbol;Acc:HGNC:7534]" | 14822 | -0.589 | -0.1385 | Yes |
| 182 | B3GAT3 | "beta-1,3-glucuronyltransferase 3 [Source:HGNC Symbol;Acc:HGNC:923]" | 14897 | -0.617 | -0.1331 | Yes |
| 183 | EGLN3 | egl-9 family hypoxia inducible factor 3 [Source:HGNC Symbol;Acc:HGNC:14661] | 14996 | -0.663 | -0.1284 | Yes |
| 184 | CACNA1H | calcium voltage-gated channel subunit alpha1 H [Source:HGNC Symbol;Acc:HGNC:1395] | 15086 | -0.710 | -0.1223 | Yes |
| 185 | GPR87 | G protein-coupled receptor 87 [Source:HGNC Symbol;Acc:HGNC:4538] | 15118 | -0.730 | -0.1122 | Yes |
| 186 | CHST12 | carbohydrate sulfotransferase 12 [Source:HGNC Symbol;Acc:HGNC:17423] | 15122 | -0.731 | -0.1002 | Yes |
| 187 | GPC4 | glypican 4 [Source:HGNC Symbol;Acc:HGNC:4452] | 15147 | -0.751 | -0.0893 | Yes |
| 188 | DDIT4 | DNA damage inducible transcript 4 [Source:HGNC Symbol;Acc:HGNC:24944] | 15359 | -0.865 | -0.0885 | Yes |
| 189 | STC1 | stanniocalcin 1 [Source:HGNC Symbol;Acc:HGNC:11373] | 15482 | -1.009 | -0.0796 | Yes |
| 190 | NT5E | 5'-nucleotidase ecto [Source:HGNC Symbol;Acc:HGNC:8021] | 15487 | -1.022 | -0.0629 | Yes |
| 191 | CLDN9 | claudin 9 [Source:HGNC Symbol;Acc:HGNC:2051] | 15520 | -1.083 | -0.0470 | Yes |
| 192 | TGFA | transforming growth factor alpha [Source:HGNC Symbol;Acc:HGNC:11765] | 15521 | -1.084 | -0.0289 | Yes |
| 193 | IGFBP3 | insulin like growth factor binding protein 3 [Source:HGNC Symbol;Acc:HGNC:5472] | 15536 | -1.107 | -0.0114 | Yes |
| 194 | CHST1 | carbohydrate sulfotransferase 1 [Source:HGNC Symbol;Acc:HGNC:1969] | 15569 | -1.196 | 0.0064 | Yes |
Table: GSEA details [plain text format]

  

Fig 2: HALLMARK\_GLYCOLYSIS      
 Blue-Pink O' Gram in the Space of the Analyzed GeneSet

  

Fig 3: HALLMARK\_GLYCOLYSIS: Random ES distribution      
 Gene set null distribution of ES for **HALLMARK\_GLYCOLYSIS**
