# Supplementary material for: In vivo CRISPR screens reveal Serpinb9 and Adam2 as regulators of immune therapy response in lung cancer
Source: Nat Commun. 2023 May 31;14:3150. doi: 10.1038/s41467-023-38841-7 (PMC10232477; doi:10.1038/s41467-023-38841-7)
Supplement: Supplementary file 13 — Source Data [file 41467_2023_38841_MOESM13_ESM.zip › Figure 4E.zip!/my_analysis.Gsea.1640146340255_Hallmark/HALLMARK_TNFA_SIGNALING_VIA_NFKB.html]

Details for gene set HALLMARK\_TNFA\_SIGNALING\_VIA\_NFKB[GSEA]

|  || Dataset | December 21, 2021\_for GSEA run\_collapsed\_to\_symbols.GSEA\_B6\_NI.cls #CTLR\_NI\_versus\_ADAM2\_NI |
| Phenotype | GSEA\_B6\_NI.cls#CTLR\_NI\_versus\_ADAM2\_NI |
| Upregulated in class | CTLR\_NI |
| GeneSet | HALLMARK\_TNFA\_SIGNALING\_VIA\_NFKB |
| Enrichment Score (ES) | 0.43271732 |
| Normalized Enrichment Score (NES) | 1.4284118 |
| Nominal p-value | 0.09701493 |
| FDR q-value | 0.27549076 |
| FWER p-Value | 0.698 |
Table: GSEA Results Summary

  

Fig 1: Enrichment plot: HALLMARK\_TNFA\_SIGNALING\_VIA\_NFKB      
 Profile of the Running ES Score & Positions of GeneSet Members on the Rank Ordered List

  

| SYMBOL | TITLE | RANK IN GENE LIST | RANK METRIC SCORE | RUNNING ES | CORE ENRICHMENT || 1 | CXCL11 | C-X-C motif chemokine ligand 11 [Source:HGNC Symbol;Acc:HGNC:10638] | 2 | 3.216 | 0.0363 | Yes |
| 2 | CXCL10 | C-X-C motif chemokine ligand 10 [Source:HGNC Symbol;Acc:HGNC:10637] | 142 | 1.666 | 0.0462 | Yes |
| 3 | CCL5 | C-C motif chemokine ligand 5 [Source:HGNC Symbol;Acc:HGNC:10632] | 169 | 1.591 | 0.0626 | Yes |
| 4 | IFIH1 | interferon induced with helicase C domain 1 [Source:HGNC Symbol;Acc:HGNC:18873] | 183 | 1.531 | 0.0791 | Yes |
| 5 | MAP3K8 | mitogen-activated protein kinase kinase kinase 8 [Source:HGNC Symbol;Acc:HGNC:6860] | 202 | 1.482 | 0.0947 | Yes |
| 6 | NR4A3 | nuclear receptor subfamily 4 group A member 3 [Source:HGNC Symbol;Acc:HGNC:7982] | 230 | 1.414 | 0.1090 | Yes |
| 7 | IFIT2 | interferon induced protein with tetratricopeptide repeats 2 [Source:HGNC Symbol;Acc:HGNC:5409] | 235 | 1.400 | 0.1246 | Yes |
| 8 | TAP1 | "transporter 1, ATP binding cassette subfamily B member [Source:HGNC Symbol;Acc:HGNC:43]" | 241 | 1.389 | 0.1400 | Yes |
| 9 | PTX3 | pentraxin 3 [Source:HGNC Symbol;Acc:HGNC:9692] | 279 | 1.338 | 0.1528 | Yes |
| 10 | IRF1 | interferon regulatory factor 1 [Source:HGNC Symbol;Acc:HGNC:6116] | 295 | 1.310 | 0.1666 | Yes |
| 11 | IL1B | interleukin 1 beta [Source:HGNC Symbol;Acc:HGNC:5992] | 303 | 1.298 | 0.1809 | Yes |
| 12 | BIRC3 | baculoviral IAP repeat containing 3 [Source:HGNC Symbol;Acc:HGNC:591] | 369 | 1.208 | 0.1904 | Yes |
| 13 | IL6 | interleukin 6 [Source:HGNC Symbol;Acc:HGNC:6018] | 378 | 1.200 | 0.2035 | Yes |
| 14 | DRAM1 | DNA damage regulated autophagy modulator 1 [Source:HGNC Symbol;Acc:HGNC:25645] | 402 | 1.165 | 0.2152 | Yes |
| 15 | CCRL2 | C-C motif chemokine receptor like 2 [Source:HGNC Symbol;Acc:HGNC:1612] | 427 | 1.145 | 0.2266 | Yes |
| 16 | ATF3 | activating transcription factor 3 [Source:HGNC Symbol;Acc:HGNC:785] | 453 | 1.117 | 0.2377 | Yes |
| 17 | CD69 | CD69 molecule [Source:HGNC Symbol;Acc:HGNC:1694] | 476 | 1.094 | 0.2486 | Yes |
| 18 | IL15RA | interleukin 15 receptor subunit alpha [Source:HGNC Symbol;Acc:HGNC:5978] | 494 | 1.080 | 0.2598 | Yes |
| 19 | G0S2 | G0/G1 switch 2 [Source:HGNC Symbol;Acc:HGNC:30229] | 516 | 1.054 | 0.2704 | Yes |
| 20 | PLAU | "plasminogen activator, urokinase [Source:HGNC Symbol;Acc:HGNC:9052]" | 537 | 1.033 | 0.2808 | Yes |
| 21 | IL1A | interleukin 1 alpha [Source:HGNC Symbol;Acc:HGNC:5991] | 540 | 1.032 | 0.2923 | Yes |
| 22 | DDX58 | DExD/H-box helicase 58 [Source:HGNC Symbol;Acc:HGNC:19102] | 544 | 1.031 | 0.3038 | Yes |
| 23 | CD80 | CD80 molecule [Source:HGNC Symbol;Acc:HGNC:1700] | 608 | 0.992 | 0.3110 | Yes |
| 24 | GADD45B | growth arrest and DNA damage inducible beta [Source:HGNC Symbol;Acc:HGNC:4096] | 667 | 0.954 | 0.3180 | Yes |
| 25 | ABCA1 | ATP binding cassette subfamily A member 1 [Source:HGNC Symbol;Acc:HGNC:29] | 717 | 0.923 | 0.3253 | Yes |
| 26 | IL7R | interleukin 7 receptor [Source:HGNC Symbol;Acc:HGNC:6024] | 816 | 0.869 | 0.3289 | Yes |
| 27 | MSC | musculin [Source:HGNC Symbol;Acc:HGNC:7321] | 832 | 0.864 | 0.3377 | Yes |
| 28 | MXD1 | MAX dimerization protein 1 [Source:HGNC Symbol;Acc:HGNC:6761] | 898 | 0.842 | 0.3430 | Yes |
| 29 | BCL2A1 | BCL2 related protein A1 [Source:HGNC Symbol;Acc:HGNC:991] | 904 | 0.840 | 0.3522 | Yes |
| 30 | MARCKS | myristoylated alanine rich protein kinase C substrate [Source:HGNC Symbol;Acc:HGNC:6759] | 951 | 0.836 | 0.3587 | Yes |
| 31 | PFKFB3 | "6-phosphofructo-2-kinase/fructose-2,6-biphosphatase 3 [Source:HGNC Symbol;Acc:HGNC:8874]" | 994 | 0.822 | 0.3653 | Yes |
| 32 | SERPINB2 | serpin family B member 2 [Source:HGNC Symbol;Acc:HGNC:8584] | 1010 | 0.810 | 0.3735 | Yes |
| 33 | TRAF1 | TNF receptor associated factor 1 [Source:HGNC Symbol;Acc:HGNC:12031] | 1077 | 0.781 | 0.3781 | Yes |
| 34 | ICAM1 | intercellular adhesion molecule 1 [Source:HGNC Symbol;Acc:HGNC:5344] | 1112 | 0.767 | 0.3846 | Yes |
| 35 | NAMPT | nicotinamide phosphoribosyltransferase [Source:HGNC Symbol;Acc:HGNC:30092] | 1163 | 0.744 | 0.3898 | Yes |
| 36 | FUT4 | fucosyltransferase 4 [Source:HGNC Symbol;Acc:HGNC:4015] | 1169 | 0.743 | 0.3979 | Yes |
| 37 | SLC16A6 | solute carrier family 16 member 6 [Source:HGNC Symbol;Acc:HGNC:10927] | 1216 | 0.727 | 0.4032 | Yes |
| 38 | LAMB3 | laminin subunit beta 3 [Source:HGNC Symbol;Acc:HGNC:6490] | 1282 | 0.701 | 0.4069 | Yes |
| 39 | ICOSLG | inducible T cell costimulator ligand [Source:HGNC Symbol;Acc:HGNC:17087] | 1433 | 0.651 | 0.4046 | Yes |
| 40 | ZC3H12A | zinc finger CCCH-type containing 12A [Source:HGNC Symbol;Acc:HGNC:26259] | 1497 | 0.627 | 0.4076 | Yes |
| 41 | BCL6 | BCL6 transcription repressor [Source:HGNC Symbol;Acc:HGNC:1001] | 1565 | 0.607 | 0.4102 | Yes |
| 42 | IL18 | interleukin 18 [Source:HGNC Symbol;Acc:HGNC:5986] | 1622 | 0.588 | 0.4132 | Yes |
| 43 | TNFAIP8 | TNF alpha induced protein 8 [Source:HGNC Symbol;Acc:HGNC:17260] | 1675 | 0.574 | 0.4164 | Yes |
| 44 | TNFAIP3 | TNF alpha induced protein 3 [Source:HGNC Symbol;Acc:HGNC:11896] | 1713 | 0.564 | 0.4204 | Yes |
| 45 | JAG1 | jagged canonical Notch ligand 1 [Source:HGNC Symbol;Acc:HGNC:6188] | 1788 | 0.546 | 0.4218 | Yes |
| 46 | TNF | tumor necrosis factor [Source:HGNC Symbol;Acc:HGNC:11892] | 1880 | 0.528 | 0.4219 | Yes |
| 47 | CSF2 | colony stimulating factor 2 [Source:HGNC Symbol;Acc:HGNC:2434] | 1913 | 0.521 | 0.4257 | Yes |
| 48 | LITAF | lipopolysaccharide induced TNF factor [Source:HGNC Symbol;Acc:HGNC:16841] | 1953 | 0.512 | 0.4290 | Yes |
| 49 | PLEK | pleckstrin [Source:HGNC Symbol;Acc:HGNC:9070] | 2033 | 0.502 | 0.4296 | Yes |
| 50 | IL12B | interleukin 12B [Source:HGNC Symbol;Acc:HGNC:5970] | 2102 | 0.491 | 0.4307 | Yes |
| 51 | CEBPD | CCAAT enhancer binding protein delta [Source:HGNC Symbol;Acc:HGNC:1835] | 2158 | 0.488 | 0.4327 | Yes |
| 52 | RNF19B | ring finger protein 19B [Source:HGNC Symbol;Acc:HGNC:26886] | 2349 | 0.460 | 0.4256 | No |
| 53 | GCH1 | GTP cyclohydrolase 1 [Source:HGNC Symbol;Acc:HGNC:4193] | 2566 | 0.441 | 0.4167 | No |
| 54 | BIRC2 | baculoviral IAP repeat containing 2 [Source:HGNC Symbol;Acc:HGNC:590] | 2627 | 0.429 | 0.4177 | No |
| 55 | PTGER4 | prostaglandin E receptor 4 [Source:HGNC Symbol;Acc:HGNC:9596] | 2628 | 0.429 | 0.4225 | No |
| 56 | DUSP5 | dual specificity phosphatase 5 [Source:HGNC Symbol;Acc:HGNC:3071] | 2753 | 0.425 | 0.4193 | No |
| 57 | CLCF1 | cardiotrophin like cytokine factor 1 [Source:HGNC Symbol;Acc:HGNC:17412] | 2795 | 0.417 | 0.4214 | No |
| 58 | NFKBIA | NFKB inhibitor alpha [Source:HGNC Symbol;Acc:HGNC:7797] | 2954 | 0.411 | 0.4159 | No |
| 59 | STAT5A | signal transducer and activator of transcription 5A [Source:HGNC Symbol;Acc:HGNC:11366] | 2993 | 0.405 | 0.4180 | No |
| 60 | CD83 | CD83 molecule [Source:HGNC Symbol;Acc:HGNC:1703] | 3005 | 0.403 | 0.4219 | No |
| 61 | SERPINB8 | serpin family B member 8 [Source:HGNC Symbol;Acc:HGNC:8952] | 3310 | 0.356 | 0.4062 | No |
| 62 | BTG2 | BTG anti-proliferation factor 2 [Source:HGNC Symbol;Acc:HGNC:1131] | 3319 | 0.354 | 0.4097 | No |
| 63 | INHBA | inhibin subunit beta A [Source:HGNC Symbol;Acc:HGNC:6066] | 3323 | 0.354 | 0.4136 | No |
| 64 | CXCL1 | C-X-C motif chemokine ligand 1 [Source:HGNC Symbol;Acc:HGNC:4602] | 3324 | 0.354 | 0.4176 | No |
| 65 | SOCS3 | suppressor of cytokine signaling 3 [Source:HGNC Symbol;Acc:HGNC:19391] | 3330 | 0.352 | 0.4212 | No |
| 66 | SLC2A3 | solute carrier family 2 member 3 [Source:HGNC Symbol;Acc:HGNC:11007] | 3342 | 0.351 | 0.4245 | No |
| 67 | IFNGR2 | interferon gamma receptor 2 [Source:HGNC Symbol;Acc:HGNC:5440] | 3530 | 0.323 | 0.4161 | No |
| 68 | ETS2 | "ETS proto-oncogene 2, transcription factor [Source:HGNC Symbol;Acc:HGNC:3489]" | 3538 | 0.323 | 0.4193 | No |
| 69 | NFKBIE | NFKB inhibitor epsilon [Source:HGNC Symbol;Acc:HGNC:7799] | 3554 | 0.321 | 0.4219 | No |
| 70 | CCL2 | C-C motif chemokine ligand 2 [Source:HGNC Symbol;Acc:HGNC:10618] | 3622 | 0.312 | 0.4211 | No |
| 71 | TNC | tenascin C [Source:HGNC Symbol;Acc:HGNC:5318] | 3673 | 0.304 | 0.4214 | No |
| 72 | IL23A | interleukin 23 subunit alpha [Source:HGNC Symbol;Acc:HGNC:15488] | 3674 | 0.304 | 0.4248 | No |
| 73 | NFIL3 | "nuclear factor, interleukin 3 regulated [Source:HGNC Symbol;Acc:HGNC:7787]" | 3734 | 0.299 | 0.4244 | No |
| 74 | CFLAR | CASP8 and FADD like apoptosis regulator [Source:HGNC Symbol;Acc:HGNC:1876] | 3779 | 0.293 | 0.4248 | No |
| 75 | EGR3 | early growth response 3 [Source:HGNC Symbol;Acc:HGNC:3240] | 3940 | 0.274 | 0.4176 | No |
| 76 | TNFAIP2 | TNF alpha induced protein 2 [Source:HGNC Symbol;Acc:HGNC:11895] | 3997 | 0.266 | 0.4170 | No |
| 77 | PANX1 | pannexin 1 [Source:HGNC Symbol;Acc:HGNC:8599] | 4117 | 0.252 | 0.4122 | No |
| 78 | FOSL2 | "FOS like 2, AP-1 transcription factor subunit [Source:HGNC Symbol;Acc:HGNC:3798]" | 4207 | 0.243 | 0.4092 | No |
| 79 | CXCL3 | C-X-C motif chemokine ligand 3 [Source:HGNC Symbol;Acc:HGNC:4604] | 4256 | 0.236 | 0.4087 | No |
| 80 | BCL3 | BCL3 transcription coactivator [Source:HGNC Symbol;Acc:HGNC:998] | 4268 | 0.235 | 0.4107 | No |
| 81 | CEBPB | CCAAT enhancer binding protein beta [Source:HGNC Symbol;Acc:HGNC:1834] | 4438 | 0.219 | 0.4022 | No |
| 82 | SGK1 | serum/glucocorticoid regulated kinase 1 [Source:HGNC Symbol;Acc:HGNC:10810] | 4462 | 0.217 | 0.4032 | No |
| 83 | OLR1 | oxidized low density lipoprotein receptor 1 [Source:HGNC Symbol;Acc:HGNC:8133] | 4464 | 0.217 | 0.4056 | No |
| 84 | REL | "REL proto-oncogene, NF-kB subunit [Source:HGNC Symbol;Acc:HGNC:9954]" | 4616 | 0.204 | 0.3982 | No |
| 85 | CXCL6 | C-X-C motif chemokine ligand 6 [Source:HGNC Symbol;Acc:HGNC:10643] | 4655 | 0.200 | 0.3980 | No |
| 86 | CXCL2 | C-X-C motif chemokine ligand 2 [Source:HGNC Symbol;Acc:HGNC:4603] | 4696 | 0.197 | 0.3976 | No |
| 87 | PTPRE | protein tyrosine phosphatase receptor type E [Source:HGNC Symbol;Acc:HGNC:9669] | 4700 | 0.197 | 0.3997 | No |
| 88 | FOSB | "FosB proto-oncogene, AP-1 transcription factor subunit [Source:HGNC Symbol;Acc:HGNC:3797]" | 4908 | 0.177 | 0.3883 | No |
| 89 | B4GALT5 | "beta-1,4-galactosyltransferase 5 [Source:HGNC Symbol;Acc:HGNC:928]" | 4929 | 0.175 | 0.3890 | No |
| 90 | PER1 | period circadian regulator 1 [Source:HGNC Symbol;Acc:HGNC:8845] | 4948 | 0.173 | 0.3898 | No |
| 91 | KYNU | kynureninase [Source:HGNC Symbol;Acc:HGNC:6469] | 5014 | 0.166 | 0.3875 | No |
| 92 | IER5 | immediate early response 5 [Source:HGNC Symbol;Acc:HGNC:5393] | 5163 | 0.154 | 0.3796 | No |
| 93 | PLAUR | "plasminogen activator, urokinase receptor [Source:HGNC Symbol;Acc:HGNC:9053]" | 5260 | 0.146 | 0.3751 | No |
| 94 | HES1 | hes family bHLH transcription factor 1 [Source:HGNC Symbol;Acc:HGNC:5192] | 5322 | 0.141 | 0.3727 | No |
| 95 | CCNL1 | cyclin L1 [Source:HGNC Symbol;Acc:HGNC:20569] | 5468 | 0.130 | 0.3648 | No |
| 96 | TIPARP | TCDD inducible poly(ADP-ribose) polymerase [Source:HGNC Symbol;Acc:HGNC:23696] | 5482 | 0.128 | 0.3654 | No |
| 97 | GEM | GTP binding protein overexpressed in skeletal muscle [Source:HGNC Symbol;Acc:HGNC:4234] | 5511 | 0.127 | 0.3651 | No |
| 98 | KLF6 | Kruppel like factor 6 [Source:HGNC Symbol;Acc:HGNC:2235] | 5555 | 0.123 | 0.3637 | No |
| 99 | PDE4B | phosphodiesterase 4B [Source:HGNC Symbol;Acc:HGNC:8781] | 5633 | 0.116 | 0.3600 | No |
| 100 | KLF10 | Kruppel like factor 10 [Source:HGNC Symbol;Acc:HGNC:11810] | 5683 | 0.114 | 0.3581 | No |
| 101 | RIPK2 | receptor interacting serine/threonine kinase 2 [Source:HGNC Symbol;Acc:HGNC:10020] | 5692 | 0.113 | 0.3589 | No |
| 102 | TRIB1 | tribbles pseudokinase 1 [Source:HGNC Symbol;Acc:HGNC:16891] | 6002 | 0.091 | 0.3400 | No |
| 103 | TNIP2 | TNFAIP3 interacting protein 2 [Source:HGNC Symbol;Acc:HGNC:19118] | 6111 | 0.082 | 0.3339 | No |
| 104 | SAT1 | spermidine/spermine N1-acetyltransferase 1 [Source:HGNC Symbol;Acc:HGNC:10540] | 6202 | 0.076 | 0.3290 | No |
| 105 | MCL1 | "MCL1 apoptosis regulator, BCL2 family member [Source:HGNC Symbol;Acc:HGNC:6943]" | 6249 | 0.073 | 0.3268 | No |
| 106 | TANK | TRAF family member associated NFKB activator [Source:HGNC Symbol;Acc:HGNC:11562] | 6456 | 0.058 | 0.3142 | No |
| 107 | MYC | "MYC proto-oncogene, bHLH transcription factor [Source:HGNC Symbol;Acc:HGNC:7553]" | 6504 | 0.055 | 0.3117 | No |
| 108 | EGR2 | early growth response 2 [Source:HGNC Symbol;Acc:HGNC:3239] | 6612 | 0.048 | 0.3054 | No |
| 109 | BTG3 | BTG anti-proliferation factor 3 [Source:HGNC Symbol;Acc:HGNC:1132] | 6622 | 0.047 | 0.3053 | No |
| 110 | NFKB1 | nuclear factor kappa B subunit 1 [Source:HGNC Symbol;Acc:HGNC:7794] | 6748 | 0.040 | 0.2977 | No |
| 111 | B4GALT1 | "beta-1,4-galactosyltransferase 1 [Source:HGNC Symbol;Acc:HGNC:924]" | 6824 | 0.035 | 0.2932 | No |
| 112 | ATP2B1 | ATPase plasma membrane Ca2+ transporting 1 [Source:HGNC Symbol;Acc:HGNC:814] | 6837 | 0.034 | 0.2928 | No |
| 113 | SLC2A6 | solute carrier family 2 member 6 [Source:HGNC Symbol;Acc:HGNC:11011] | 7134 | 0.016 | 0.2739 | No |
| 114 | PTGS2 | prostaglandin-endoperoxide synthase 2 [Source:HGNC Symbol;Acc:HGNC:9605] | 7157 | 0.015 | 0.2726 | No |
| 115 | FOS | "Fos proto-oncogene, AP-1 transcription factor subunit [Source:HGNC Symbol;Acc:HGNC:3796]" | 7164 | 0.014 | 0.2724 | No |
| 116 | PDLIM5 | PDZ and LIM domain 5 [Source:HGNC Symbol;Acc:HGNC:17468] | 7279 | 0.008 | 0.2651 | No |
| 117 | LIF | LIF interleukin 6 family cytokine [Source:HGNC Symbol;Acc:HGNC:6596] | 7310 | 0.006 | 0.2633 | No |
| 118 | PHLDA1 | pleckstrin homology like domain family A member 1 [Source:HGNC Symbol;Acc:HGNC:8933] | 7553 | -0.007 | 0.2477 | No |
| 119 | VEGFA | vascular endothelial growth factor A [Source:HGNC Symbol;Acc:HGNC:12680] | 7671 | -0.012 | 0.2403 | No |
| 120 | TNFRSF9 | TNF receptor superfamily member 9 [Source:HGNC Symbol;Acc:HGNC:11924] | 7797 | -0.019 | 0.2324 | No |
| 121 | SOD2 | superoxide dismutase 2 [Source:HGNC Symbol;Acc:HGNC:11180] | 7867 | -0.022 | 0.2282 | No |
| 122 | GFPT2 | glutamine-fructose-6-phosphate transaminase 2 [Source:HGNC Symbol;Acc:HGNC:4242] | 7969 | -0.027 | 0.2220 | No |
| 123 | KLF9 | Kruppel like factor 9 [Source:HGNC Symbol;Acc:HGNC:1123] | 8000 | -0.029 | 0.2204 | No |
| 124 | PPP1R15A | protein phosphatase 1 regulatory subunit 15A [Source:HGNC Symbol;Acc:HGNC:14375] | 8019 | -0.030 | 0.2195 | No |
| 125 | BTG1 | BTG anti-proliferation factor 1 [Source:HGNC Symbol;Acc:HGNC:1130] | 8137 | -0.037 | 0.2124 | No |
| 126 | MAP2K3 | mitogen-activated protein kinase kinase 3 [Source:HGNC Symbol;Acc:HGNC:6843] | 8186 | -0.039 | 0.2097 | No |
| 127 | SDC4 | syndecan 4 [Source:HGNC Symbol;Acc:HGNC:10661] | 8318 | -0.044 | 0.2018 | No |
| 128 | ID2 | inhibitor of DNA binding 2 [Source:HGNC Symbol;Acc:HGNC:5361] | 8387 | -0.048 | 0.1979 | No |
| 129 | NFAT5 | nuclear factor of activated T cells 5 [Source:HGNC Symbol;Acc:HGNC:7774] | 8468 | -0.053 | 0.1934 | No |
| 130 | ZFP36 | ZFP36 ring finger protein [Source:HGNC Symbol;Acc:HGNC:12862] | 8519 | -0.056 | 0.1908 | No |
| 131 | DUSP2 | dual specificity phosphatase 2 [Source:HGNC Symbol;Acc:HGNC:3068] | 8547 | -0.058 | 0.1897 | No |
| 132 | DNAJB4 | DnaJ heat shock protein family (Hsp40) member B4 [Source:HGNC Symbol;Acc:HGNC:14886] | 8777 | -0.071 | 0.1757 | No |
| 133 | HBEGF | heparin binding EGF like growth factor [Source:HGNC Symbol;Acc:HGNC:3059] | 8784 | -0.071 | 0.1761 | No |
| 134 | EHD1 | EH domain containing 1 [Source:HGNC Symbol;Acc:HGNC:3242] | 8788 | -0.071 | 0.1767 | No |
| 135 | ZBTB10 | zinc finger and BTB domain containing 10 [Source:HGNC Symbol;Acc:HGNC:30953] | 9002 | -0.083 | 0.1639 | No |
| 136 | RELB | "RELB proto-oncogene, NF-kB subunit [Source:HGNC Symbol;Acc:HGNC:9956]" | 9175 | -0.092 | 0.1538 | No |
| 137 | PLPP3 | phospholipid phosphatase 3 [Source:HGNC Symbol;Acc:HGNC:9229] | 9319 | -0.099 | 0.1457 | No |
| 138 | TLR2 | toll like receptor 2 [Source:HGNC Symbol;Acc:HGNC:11848] | 9404 | -0.105 | 0.1414 | No |
| 139 | TSC22D1 | TSC22 domain family member 1 [Source:HGNC Symbol;Acc:HGNC:16826] | 9431 | -0.106 | 0.1410 | No |
| 140 | SPSB1 | splA/ryanodine receptor domain and SOCS box containing 1 [Source:HGNC Symbol;Acc:HGNC:30628] | 9531 | -0.113 | 0.1358 | No |
| 141 | NR4A2 | nuclear receptor subfamily 4 group A member 2 [Source:HGNC Symbol;Acc:HGNC:7981] | 9601 | -0.118 | 0.1327 | No |
| 142 | CCND1 | cyclin D1 [Source:HGNC Symbol;Acc:HGNC:1582] | 9851 | -0.134 | 0.1181 | No |
| 143 | NINJ1 | ninjurin 1 [Source:HGNC Symbol;Acc:HGNC:7824] | 9852 | -0.134 | 0.1197 | No |
| 144 | EDN1 | endothelin 1 [Source:HGNC Symbol;Acc:HGNC:3176] | 9964 | -0.140 | 0.1141 | No |
| 145 | SERPINE1 | serpin family E member 1 [Source:HGNC Symbol;Acc:HGNC:8583] | 10052 | -0.145 | 0.1101 | No |
| 146 | JUN | "Jun proto-oncogene, AP-1 transcription factor subunit [Source:HGNC Symbol;Acc:HGNC:6204]" | 10257 | -0.157 | 0.0987 | No |
| 147 | KLF4 | Kruppel like factor 4 [Source:HGNC Symbol;Acc:HGNC:6348] | 10415 | -0.167 | 0.0904 | No |
| 148 | F3 | "coagulation factor III, tissue factor [Source:HGNC Symbol;Acc:HGNC:3541]" | 10575 | -0.175 | 0.0821 | No |
| 149 | CD44 | CD44 molecule (Indian blood group) [Source:HGNC Symbol;Acc:HGNC:1681] | 10617 | -0.178 | 0.0815 | No |
| 150 | GPR183 | G protein-coupled receptor 183 [Source:HGNC Symbol;Acc:HGNC:3128] | 10846 | -0.191 | 0.0689 | No |
| 151 | RHOB | ras homolog family member B [Source:HGNC Symbol;Acc:HGNC:668] | 11216 | -0.212 | 0.0475 | No |
| 152 | TUBB2A | tubulin beta 2A class IIa [Source:HGNC Symbol;Acc:HGNC:12412] | 11519 | -0.230 | 0.0306 | No |
| 153 | KDM6B | lysine demethylase 6B [Source:HGNC Symbol;Acc:HGNC:29012] | 11539 | -0.231 | 0.0320 | No |
| 154 | EIF1 | eukaryotic translation initiation factor 1 [Source:HGNC Symbol;Acc:HGNC:3249] | 11559 | -0.232 | 0.0334 | No |
| 155 | FOSL1 | "FOS like 1, AP-1 transcription factor subunit [Source:HGNC Symbol;Acc:HGNC:13718]" | 11600 | -0.235 | 0.0334 | No |
| 156 | RCAN1 | regulator of calcineurin 1 [Source:HGNC Symbol;Acc:HGNC:3040] | 11628 | -0.237 | 0.0344 | No |
| 157 | YRDC | yrdC N6-threonylcarbamoyltransferase domain containing [Source:HGNC Symbol;Acc:HGNC:28905] | 11663 | -0.238 | 0.0349 | No |
| 158 | NFE2L2 | "nuclear factor, erythroid 2 like 2 [Source:HGNC Symbol;Acc:HGNC:7782]" | 11712 | -0.241 | 0.0345 | No |
| 159 | JUNB | "JunB proto-oncogene, AP-1 transcription factor subunit [Source:HGNC Symbol;Acc:HGNC:6205]" | 11848 | -0.250 | 0.0286 | No |
| 160 | TRIP10 | thyroid hormone receptor interactor 10 [Source:HGNC Symbol;Acc:HGNC:12304] | 11860 | -0.251 | 0.0308 | No |
| 161 | TGIF1 | TGFB induced factor homeobox 1 [Source:HGNC Symbol;Acc:HGNC:11776] | 12041 | -0.264 | 0.0221 | No |
| 162 | GADD45A | growth arrest and DNA damage inducible alpha [Source:HGNC Symbol;Acc:HGNC:4095] | 12093 | -0.267 | 0.0218 | No |
| 163 | IL6ST | interleukin 6 signal transducer [Source:HGNC Symbol;Acc:HGNC:6021] | 12152 | -0.270 | 0.0212 | No |
| 164 | KLF2 | Kruppel like factor 2 [Source:HGNC Symbol;Acc:HGNC:6347] | 12228 | -0.275 | 0.0194 | No |
| 165 | DENND5A | DENN domain containing 5A [Source:HGNC Symbol;Acc:HGNC:19344] | 12266 | -0.278 | 0.0202 | No |
| 166 | TNIP1 | TNFAIP3 interacting protein 1 [Source:HGNC Symbol;Acc:HGNC:16903] | 12511 | -0.297 | 0.0078 | No |
| 167 | BMP2 | bone morphogenetic protein 2 [Source:HGNC Symbol;Acc:HGNC:1069] | 12618 | -0.305 | 0.0044 | No |
| 168 | RELA | "RELA proto-oncogene, NF-kB subunit [Source:HGNC Symbol;Acc:HGNC:9955]" | 12655 | -0.308 | 0.0056 | No |
| 169 | MAFF | MAF bZIP transcription factor F [Source:HGNC Symbol;Acc:HGNC:6780] | 12675 | -0.309 | 0.0078 | No |
| 170 | NR4A1 | nuclear receptor subfamily 4 group A member 1 [Source:HGNC Symbol;Acc:HGNC:7980] | 12687 | -0.311 | 0.0106 | No |
| 171 | IER3 | immediate early response 3 [Source:HGNC Symbol;Acc:HGNC:5392] | 12833 | -0.322 | 0.0049 | No |
| 172 | AREG | amphiregulin [Source:HGNC Symbol;Acc:HGNC:651] | 13075 | -0.345 | -0.0068 | No |
| 173 | DUSP4 | dual specificity phosphatase 4 [Source:HGNC Symbol;Acc:HGNC:3070] | 13297 | -0.367 | -0.0169 | No |
| 174 | PNRC1 | proline rich nuclear receptor coactivator 1 [Source:HGNC Symbol;Acc:HGNC:17278] | 13342 | -0.372 | -0.0155 | No |
| 175 | CCN1 | cellular communication network factor 1 [Source:HGNC Symbol;Acc:HGNC:2654] | 13488 | -0.391 | -0.0205 | No |
| 176 | EFNA1 | ephrin A1 [Source:HGNC Symbol;Acc:HGNC:3221] | 13603 | -0.405 | -0.0232 | No |
| 177 | SQSTM1 | sequestosome 1 [Source:HGNC Symbol;Acc:HGNC:11280] | 13604 | -0.406 | -0.0186 | No |
| 178 | NFKB2 | nuclear factor kappa B subunit 2 [Source:HGNC Symbol;Acc:HGNC:7795] | 14002 | -0.432 | -0.0394 | No |
| 179 | BHLHE40 | basic helix-loop-helix family member e40 [Source:HGNC Symbol;Acc:HGNC:1046] | 14032 | -0.437 | -0.0363 | No |
| 180 | LDLR | low density lipoprotein receptor [Source:HGNC Symbol;Acc:HGNC:6547] | 14103 | -0.450 | -0.0357 | No |
| 181 | PMEPA1 | "prostate transmembrane protein, androgen induced 1 [Source:HGNC Symbol;Acc:HGNC:14107]" | 14329 | -0.484 | -0.0448 | No |
| 182 | DUSP1 | dual specificity phosphatase 1 [Source:HGNC Symbol;Acc:HGNC:3064] | 14433 | -0.499 | -0.0458 | No |
| 183 | PLK2 | polo like kinase 2 [Source:HGNC Symbol;Acc:HGNC:19699] | 14434 | -0.499 | -0.0402 | No |
| 184 | SNN | stannin [Source:HGNC Symbol;Acc:HGNC:11149] | 14474 | -0.509 | -0.0369 | No |
| 185 | IRS2 | insulin receptor substrate 2 [Source:HGNC Symbol;Acc:HGNC:6126] | 14481 | -0.511 | -0.0315 | No |
| 186 | EGR1 | early growth response 1 [Source:HGNC Symbol;Acc:HGNC:3238] | 14742 | -0.565 | -0.0419 | No |
| 187 | CSF1 | colony stimulating factor 1 [Source:HGNC Symbol;Acc:HGNC:2432] | 14746 | -0.566 | -0.0357 | No |
| 188 | F2RL1 | F2R like trypsin receptor 1 [Source:HGNC Symbol;Acc:HGNC:3538] | 14941 | -0.640 | -0.0410 | No |
| 189 | SMAD3 | SMAD family member 3 [Source:HGNC Symbol;Acc:HGNC:6769] | 15161 | -0.755 | -0.0466 | No |
| 190 | PHLDA2 | pleckstrin homology like domain family A member 2 [Source:HGNC Symbol;Acc:HGNC:12385] | 15209 | -0.784 | -0.0407 | No |
| 191 | IER2 | immediate early response 2 [Source:HGNC Symbol;Acc:HGNC:28871] | 15215 | -0.788 | -0.0321 | No |
| 192 | TNFAIP6 | TNF alpha induced protein 6 [Source:HGNC Symbol;Acc:HGNC:11898] | 15265 | -0.815 | -0.0260 | No |
| 193 | TNFSF9 | TNF superfamily member 9 [Source:HGNC Symbol;Acc:HGNC:11939] | 15266 | -0.818 | -0.0168 | No |
| 194 | SPHK1 | sphingosine kinase 1 [Source:HGNC Symbol;Acc:HGNC:11240] | 15316 | -0.845 | -0.0104 | No |
| 195 | FJX1 | four-jointed box kinase 1 [Source:HGNC Symbol;Acc:HGNC:17166] | 15391 | -0.882 | -0.0052 | No |
| 196 | CDKN1A | cyclin dependent kinase inhibitor 1A [Source:HGNC Symbol;Acc:HGNC:1784] | 15428 | -0.929 | 0.0030 | No |
| 197 | ACKR3 | atypical chemokine receptor 3 [Source:HGNC Symbol;Acc:HGNC:23692] | 15528 | -1.095 | 0.0090 | No |
Table: GSEA details [plain text format]

  

Fig 2: HALLMARK\_TNFA\_SIGNALING\_VIA\_NFKB      
 Blue-Pink O' Gram in the Space of the Analyzed GeneSet

  

Fig 3: HALLMARK\_TNFA\_SIGNALING\_VIA\_NFKB: Random ES distribution      
 Gene set null distribution of ES for **HALLMARK\_TNFA\_SIGNALING\_VIA\_NFKB**
