# Supplementary material for: In vivo CRISPR screens reveal Serpinb9 and Adam2 as regulators of immune therapy response in lung cancer
Source: Nat Commun. 2023 May 31;14:3150. doi: 10.1038/s41467-023-38841-7 (PMC10232477; doi:10.1038/s41467-023-38841-7)
Supplement: Supplementary file 13 — Source Data [file 41467_2023_38841_MOESM13_ESM.zip › Figure 4E.zip!/my_analysis.Gsea.1640146340255_Hallmark/HALLMARK_BILE_ACID_METABOLISM.html]

Details for gene set HALLMARK\_BILE\_ACID\_METABOLISM[GSEA]

|  || Dataset | December 21, 2021\_for GSEA run\_collapsed\_to\_symbols.GSEA\_B6\_NI.cls #CTLR\_NI\_versus\_ADAM2\_NI |
| Phenotype | GSEA\_B6\_NI.cls#CTLR\_NI\_versus\_ADAM2\_NI |
| Upregulated in class | CTLR\_NI |
| GeneSet | HALLMARK\_BILE\_ACID\_METABOLISM |
| Enrichment Score (ES) | 0.30078283 |
| Normalized Enrichment Score (NES) | 1.129101 |
| Nominal p-value | 0.11380597 |
| FDR q-value | 0.6319125 |
| FWER p-Value | 1.0 |
Table: GSEA Results Summary

  

Fig 1: Enrichment plot: HALLMARK\_BILE\_ACID\_METABOLISM      
 Profile of the Running ES Score & Positions of GeneSet Members on the Rank Ordered List

  

| SYMBOL | TITLE | RANK IN GENE LIST | RANK METRIC SCORE | RUNNING ES | CORE ENRICHMENT || 1 | LCK | "LCK proto-oncogene, Src family tyrosine kinase [Source:HGNC Symbol;Acc:HGNC:6524]" | 286 | 1.331 | 0.0167 | Yes |
| 2 | ABCA6 | ATP binding cassette subfamily A member 6 [Source:HGNC Symbol;Acc:HGNC:36] | 332 | 1.257 | 0.0469 | Yes |
| 3 | PHYH | phytanoyl-CoA 2-hydroxylase [Source:HGNC Symbol;Acc:HGNC:8940] | 665 | 0.955 | 0.0507 | Yes |
| 4 | ABCA9 | ATP binding cassette subfamily A member 9 [Source:HGNC Symbol;Acc:HGNC:39] | 680 | 0.945 | 0.0747 | Yes |
| 5 | SLC22A18 | solute carrier family 22 member 18 [Source:HGNC Symbol;Acc:HGNC:10964] | 696 | 0.937 | 0.0984 | Yes |
| 6 | ABCA1 | ATP binding cassette subfamily A member 1 [Source:HGNC Symbol;Acc:HGNC:29] | 717 | 0.923 | 0.1214 | Yes |
| 7 | SLC27A5 | solute carrier family 27 member 5 [Source:HGNC Symbol;Acc:HGNC:10999] | 803 | 0.875 | 0.1390 | Yes |
| 8 | CYP7B1 | cytochrome P450 family 7 subfamily B member 1 [Source:HGNC Symbol;Acc:HGNC:2652] | 948 | 0.837 | 0.1518 | Yes |
| 9 | ALDH1A1 | aldehyde dehydrogenase 1 family member A1 [Source:HGNC Symbol;Acc:HGNC:402] | 1061 | 0.787 | 0.1653 | Yes |
| 10 | HAO1 | hydroxyacid oxidase 1 [Source:HGNC Symbol;Acc:HGNC:4809] | 1148 | 0.750 | 0.1795 | Yes |
| 11 | SLC23A1 | solute carrier family 23 member 1 [Source:HGNC Symbol;Acc:HGNC:10974] | 1161 | 0.745 | 0.1984 | Yes |
| 12 | SLCO1A2 | solute carrier organic anion transporter family member 1A2 [Source:HGNC Symbol;Acc:HGNC:10956] | 1230 | 0.722 | 0.2130 | Yes |
| 13 | DIO2 | iodothyronine deiodinase 2 [Source:HGNC Symbol;Acc:HGNC:2884] | 1338 | 0.682 | 0.2241 | Yes |
| 14 | ACSL1 | acyl-CoA synthetase long chain family member 1 [Source:HGNC Symbol;Acc:HGNC:3569] | 1411 | 0.657 | 0.2367 | Yes |
| 15 | AR | androgen receptor [Source:HGNC Symbol;Acc:HGNC:644] | 1452 | 0.641 | 0.2510 | Yes |
| 16 | LIPE | "lipase E, hormone sensitive type [Source:HGNC Symbol;Acc:HGNC:6621]" | 1696 | 0.570 | 0.2504 | Yes |
| 17 | SLC27A2 | solute carrier family 27 member 2 [Source:HGNC Symbol;Acc:HGNC:10996] | 1720 | 0.562 | 0.2638 | Yes |
| 18 | ABCA8 | ATP binding cassette subfamily A member 8 [Source:HGNC Symbol;Acc:HGNC:38] | 1834 | 0.537 | 0.2706 | Yes |
| 19 | ABCD2 | ATP binding cassette subfamily D member 2 [Source:HGNC Symbol;Acc:HGNC:66] | 1973 | 0.508 | 0.2752 | Yes |
| 20 | RXRG | retinoid X receptor gamma [Source:HGNC Symbol;Acc:HGNC:10479] | 2013 | 0.503 | 0.2859 | Yes |
| 21 | SOAT2 | sterol O-acyltransferase 2 [Source:HGNC Symbol;Acc:HGNC:11178] | 2105 | 0.491 | 0.2930 | Yes |
| 22 | EPHX2 | epoxide hydrolase 2 [Source:HGNC Symbol;Acc:HGNC:3402] | 2182 | 0.482 | 0.3008 | Yes |
| 23 | ABCG8 | ATP binding cassette subfamily G member 8 [Source:HGNC Symbol;Acc:HGNC:13887] | 2949 | 0.411 | 0.2624 | No |
| 24 | ABCG4 | ATP binding cassette subfamily G member 4 [Source:HGNC Symbol;Acc:HGNC:13884] | 2971 | 0.409 | 0.2718 | No |
| 25 | GNMT | glycine N-methyltransferase [Source:HGNC Symbol;Acc:HGNC:4415] | 3059 | 0.394 | 0.2766 | No |
| 26 | NPC1 | NPC intracellular cholesterol transporter 1 [Source:HGNC Symbol;Acc:HGNC:7897] | 3270 | 0.363 | 0.2727 | No |
| 27 | PEX26 | peroxisomal biogenesis factor 26 [Source:HGNC Symbol;Acc:HGNC:22965] | 3369 | 0.347 | 0.2755 | No |
| 28 | AQP9 | aquaporin 9 [Source:HGNC Symbol;Acc:HGNC:643] | 3493 | 0.329 | 0.2763 | No |
| 29 | ABCA4 | ATP binding cassette subfamily A member 4 [Source:HGNC Symbol;Acc:HGNC:34] | 3559 | 0.321 | 0.2805 | No |
| 30 | ALDH8A1 | aldehyde dehydrogenase 8 family member A1 [Source:HGNC Symbol;Acc:HGNC:15471] | 4096 | 0.254 | 0.2528 | No |
| 31 | SULT2B1 | sulfotransferase family 2B member 1 [Source:HGNC Symbol;Acc:HGNC:11459] | 4193 | 0.245 | 0.2531 | No |
| 32 | PAOX | polyamine oxidase [Source:HGNC Symbol;Acc:HGNC:20837] | 4341 | 0.229 | 0.2496 | No |
| 33 | OPTN | optineurin [Source:HGNC Symbol;Acc:HGNC:17142] | 4423 | 0.220 | 0.2502 | No |
| 34 | ACSL5 | acyl-CoA synthetase long chain family member 5 [Source:HGNC Symbol;Acc:HGNC:16526] | 4458 | 0.217 | 0.2538 | No |
| 35 | HSD17B11 | hydroxysteroid 17-beta dehydrogenase 11 [Source:HGNC Symbol;Acc:HGNC:22960] | 4698 | 0.197 | 0.2436 | No |
| 36 | GSTK1 | glutathione S-transferase kappa 1 [Source:HGNC Symbol;Acc:HGNC:16906] | 5019 | 0.166 | 0.2274 | No |
| 37 | ATXN1 | ataxin 1 [Source:HGNC Symbol;Acc:HGNC:10548] | 5203 | 0.151 | 0.2196 | No |
| 38 | CROT | carnitine O-octanoyltransferase [Source:HGNC Symbol;Acc:HGNC:2366] | 5358 | 0.138 | 0.2134 | No |
| 39 | CYP27A1 | cytochrome P450 family 27 subfamily A member 1 [Source:HGNC Symbol;Acc:HGNC:2605] | 5408 | 0.134 | 0.2138 | No |
| 40 | BBOX1 | gamma-butyrobetaine hydroxylase 1 [Source:HGNC Symbol;Acc:HGNC:964] | 5419 | 0.134 | 0.2167 | No |
| 41 | BCAR3 | "BCAR3 adaptor protein, NSP family member [Source:HGNC Symbol;Acc:HGNC:973]" | 5937 | 0.096 | 0.1860 | No |
| 42 | PNPLA8 | patatin like phospholipase domain containing 8 [Source:HGNC Symbol;Acc:HGNC:28900] | 6164 | 0.078 | 0.1735 | No |
| 43 | PRDX5 | peroxiredoxin 5 [Source:HGNC Symbol;Acc:HGNC:9355] | 6408 | 0.062 | 0.1595 | No |
| 44 | ALDH9A1 | aldehyde dehydrogenase 9 family member A1 [Source:HGNC Symbol;Acc:HGNC:412] | 6489 | 0.056 | 0.1559 | No |
| 45 | PEX16 | peroxisomal biogenesis factor 16 [Source:HGNC Symbol;Acc:HGNC:8857] | 6520 | 0.054 | 0.1554 | No |
| 46 | ABCD1 | ATP binding cassette subfamily D member 1 [Source:HGNC Symbol;Acc:HGNC:61] | 6559 | 0.051 | 0.1543 | No |
| 47 | PEX7 | peroxisomal biogenesis factor 7 [Source:HGNC Symbol;Acc:HGNC:8860] | 6600 | 0.048 | 0.1530 | No |
| 48 | APOA1 | apolipoprotein A1 [Source:HGNC Symbol;Acc:HGNC:600] | 7013 | 0.023 | 0.1271 | No |
| 49 | CH25H | cholesterol 25-hydroxylase [Source:HGNC Symbol;Acc:HGNC:1907] | 7040 | 0.022 | 0.1260 | No |
| 50 | PEX6 | peroxisomal biogenesis factor 6 [Source:HGNC Symbol;Acc:HGNC:8859] | 7061 | 0.020 | 0.1253 | No |
| 51 | SCP2 | sterol carrier protein 2 [Source:HGNC Symbol;Acc:HGNC:10606] | 7266 | 0.009 | 0.1124 | No |
| 52 | ISOC1 | isochorismatase domain containing 1 [Source:HGNC Symbol;Acc:HGNC:24254] | 7415 | -0.000 | 0.1029 | No |
| 53 | SLC23A2 | solute carrier family 23 member 2 [Source:HGNC Symbol;Acc:HGNC:10973] | 7496 | -0.004 | 0.0979 | No |
| 54 | CYP8B1 | cytochrome P450 family 8 subfamily B member 1 [Source:HGNC Symbol;Acc:HGNC:2653] | 7624 | -0.009 | 0.0900 | No |
| 55 | EFHC1 | EF-hand domain containing 1 [Source:HGNC Symbol;Acc:HGNC:16406] | 7778 | -0.017 | 0.0806 | No |
| 56 | HSD17B4 | hydroxysteroid 17-beta dehydrogenase 4 [Source:HGNC Symbol;Acc:HGNC:5213] | 7943 | -0.026 | 0.0707 | No |
| 57 | GCLM | glutamate-cysteine ligase modifier subunit [Source:HGNC Symbol;Acc:HGNC:4312] | 7968 | -0.027 | 0.0699 | No |
| 58 | IDH1 | isocitrate dehydrogenase (NADP(+)) 1 [Source:HGNC Symbol;Acc:HGNC:5382] | 8014 | -0.030 | 0.0678 | No |
| 59 | ABCD3 | ATP binding cassette subfamily D member 3 [Source:HGNC Symbol;Acc:HGNC:67] | 8074 | -0.033 | 0.0649 | No |
| 60 | ABCA5 | ATP binding cassette subfamily A member 5 [Source:HGNC Symbol;Acc:HGNC:35] | 8671 | -0.065 | 0.0283 | No |
| 61 | PEX19 | peroxisomal biogenesis factor 19 [Source:HGNC Symbol;Acc:HGNC:9713] | 9045 | -0.085 | 0.0066 | No |
| 62 | CAT | catalase [Source:HGNC Symbol;Acc:HGNC:1516] | 9069 | -0.086 | 0.0074 | No |
| 63 | IDI1 | isopentenyl-diphosphate delta isomerase 1 [Source:HGNC Symbol;Acc:HGNC:5387] | 9590 | -0.117 | -0.0230 | No |
| 64 | GNPAT | glyceronephosphate O-acyltransferase [Source:HGNC Symbol;Acc:HGNC:4416] | 9820 | -0.132 | -0.0342 | No |
| 65 | PECR | peroxisomal trans-2-enoyl-CoA reductase [Source:HGNC Symbol;Acc:HGNC:18281] | 9823 | -0.132 | -0.0308 | No |
| 66 | PEX1 | peroxisomal biogenesis factor 1 [Source:HGNC Symbol;Acc:HGNC:8850] | 10328 | -0.161 | -0.0590 | No |
| 67 | NR3C2 | nuclear receptor subfamily 3 group C member 2 [Source:HGNC Symbol;Acc:HGNC:7979] | 10397 | -0.166 | -0.0590 | No |
| 68 | LONP2 | "lon peptidase 2, peroxisomal [Source:HGNC Symbol;Acc:HGNC:20598]" | 10542 | -0.174 | -0.0636 | No |
| 69 | SOD1 | superoxide dismutase 1 [Source:HGNC Symbol;Acc:HGNC:11179] | 10977 | -0.199 | -0.0863 | No |
| 70 | PEX13 | peroxisomal biogenesis factor 13 [Source:HGNC Symbol;Acc:HGNC:8855] | 11000 | -0.200 | -0.0824 | No |
| 71 | CYP39A1 | cytochrome P450 family 39 subfamily A member 1 [Source:HGNC Symbol;Acc:HGNC:17449] | 11047 | -0.203 | -0.0800 | No |
| 72 | ABCA2 | ATP binding cassette subfamily A member 2 [Source:HGNC Symbol;Acc:HGNC:32] | 11305 | -0.217 | -0.0908 | No |
| 73 | PFKM | "phosphofructokinase, muscle [Source:HGNC Symbol;Acc:HGNC:8877]" | 11673 | -0.239 | -0.1081 | No |
| 74 | NEDD4 | NEDD4 E3 ubiquitin protein ligase [Source:HGNC Symbol;Acc:HGNC:7727] | 12040 | -0.264 | -0.1247 | No |
| 75 | PEX11G | peroxisomal biogenesis factor 11 gamma [Source:HGNC Symbol;Acc:HGNC:20208] | 12061 | -0.265 | -0.1190 | No |
| 76 | DHCR24 | 24-dehydrocholesterol reductase [Source:HGNC Symbol;Acc:HGNC:2859] | 12239 | -0.276 | -0.1231 | No |
| 77 | FDXR | ferredoxin reductase [Source:HGNC Symbol;Acc:HGNC:3642] | 12240 | -0.277 | -0.1158 | No |
| 78 | MLYCD | malonyl-CoA decarboxylase [Source:HGNC Symbol;Acc:HGNC:7150] | 12390 | -0.288 | -0.1178 | No |
| 79 | SLC29A1 | solute carrier family 29 member 1 (Augustine blood group) [Source:HGNC Symbol;Acc:HGNC:11003] | 12506 | -0.296 | -0.1174 | No |
| 80 | KLF1 | Kruppel like factor 1 [Source:HGNC Symbol;Acc:HGNC:6345] | 13121 | -0.349 | -0.1476 | No |
| 81 | PEX12 | peroxisomal biogenesis factor 12 [Source:HGNC Symbol;Acc:HGNC:8854] | 13122 | -0.349 | -0.1384 | No |
| 82 | AMACR | alpha-methylacyl-CoA racemase [Source:HGNC Symbol;Acc:HGNC:451] | 13192 | -0.355 | -0.1335 | No |
| 83 | CYP46A1 | cytochrome P450 family 46 subfamily A member 1 [Source:HGNC Symbol;Acc:HGNC:2641] | 13451 | -0.385 | -0.1399 | No |
| 84 | FADS1 | fatty acid desaturase 1 [Source:HGNC Symbol;Acc:HGNC:3574] | 13531 | -0.396 | -0.1346 | No |
| 85 | FADS2 | fatty acid desaturase 2 [Source:HGNC Symbol;Acc:HGNC:3575] | 13558 | -0.400 | -0.1257 | No |
| 86 | SULT1B1 | sulfotransferase family 1B member 1 [Source:HGNC Symbol;Acc:HGNC:17845] | 13712 | -0.415 | -0.1246 | No |
| 87 | PIPOX | pipecolic acid and sarcosine oxidase [Source:HGNC Symbol;Acc:HGNC:17804] | 13723 | -0.415 | -0.1144 | No |
| 88 | ABCA3 | ATP binding cassette subfamily A member 3 [Source:HGNC Symbol;Acc:HGNC:33] | 13805 | -0.419 | -0.1085 | No |
| 89 | IDH2 | isocitrate dehydrogenase (NADP(+)) 2 [Source:HGNC Symbol;Acc:HGNC:5383] | 13807 | -0.419 | -0.0975 | No |
| 90 | HACL1 | 2-hydroxyacyl-CoA lyase 1 [Source:HGNC Symbol;Acc:HGNC:17856] | 14181 | -0.463 | -0.1093 | No |
| 91 | RXRA | retinoid X receptor alpha [Source:HGNC Symbol;Acc:HGNC:10477] | 14541 | -0.522 | -0.1186 | No |
| 92 | PEX11A | peroxisomal biogenesis factor 11 alpha [Source:HGNC Symbol;Acc:HGNC:8852] | 14698 | -0.553 | -0.1141 | No |
| 93 | NUDT12 | nudix hydrolase 12 [Source:HGNC Symbol;Acc:HGNC:18826] | 14767 | -0.572 | -0.1034 | No |
| 94 | PXMP2 | peroxisomal membrane protein 2 [Source:HGNC Symbol;Acc:HGNC:9716] | 14860 | -0.603 | -0.0935 | No |
| 95 | RETSAT | retinol saturase [Source:HGNC Symbol;Acc:HGNC:25991] | 14885 | -0.613 | -0.0789 | No |
| 96 | TFCP2L1 | transcription factor CP2 like 1 [Source:HGNC Symbol;Acc:HGNC:17925] | 14916 | -0.626 | -0.0643 | No |
| 97 | BMP6 | bone morphogenetic protein 6 [Source:HGNC Symbol;Acc:HGNC:1073] | 14991 | -0.661 | -0.0517 | No |
| 98 | RBP1 | retinol binding protein 1 [Source:HGNC Symbol;Acc:HGNC:9919] | 15143 | -0.747 | -0.0417 | No |
| 99 | SLC35B2 | solute carrier family 35 member B2 [Source:HGNC Symbol;Acc:HGNC:16872] | 15167 | -0.759 | -0.0232 | No |
| 100 | NR1H4 | nuclear receptor subfamily 1 group H member 4 [Source:HGNC Symbol;Acc:HGNC:7967] | 15660 | -2.101 | 0.0005 | No |
Table: GSEA details [plain text format]

  

Fig 2: HALLMARK\_BILE\_ACID\_METABOLISM      
 Blue-Pink O' Gram in the Space of the Analyzed GeneSet

  

Fig 3: HALLMARK\_BILE\_ACID\_METABOLISM: Random ES distribution      
 Gene set null distribution of ES for **HALLMARK\_BILE\_ACID\_METABOLISM**
