# Supplementary material for: In vivo CRISPR screens reveal Serpinb9 and Adam2 as regulators of immune therapy response in lung cancer
Source: Nat Commun. 2023 May 31;14:3150. doi: 10.1038/s41467-023-38841-7 (PMC10232477; doi:10.1038/s41467-023-38841-7)
Supplement: Supplementary file 13 — Source Data [file 41467_2023_38841_MOESM13_ESM.zip › Figure 4E.zip!/my_analysis.Gsea.1640146340255_Hallmark/HALLMARK_MTORC1_SIGNALING.html]

Details for gene set HALLMARK\_MTORC1\_SIGNALING[GSEA]

|  || Dataset | December 21, 2021\_for GSEA run\_collapsed\_to\_symbols.GSEA\_B6\_NI.cls #CTLR\_NI\_versus\_ADAM2\_NI |
| Phenotype | GSEA\_B6\_NI.cls#CTLR\_NI\_versus\_ADAM2\_NI |
| Upregulated in class | ADAM2\_NI |
| GeneSet | HALLMARK\_MTORC1\_SIGNALING |
| Enrichment Score (ES) | -0.1637146 |
| Normalized Enrichment Score (NES) | -0.74397975 |
| Nominal p-value | 0.78125 |
| FDR q-value | 0.9091442 |
| FWER p-Value | 1.0 |
Table: GSEA Results Summary

  

Fig 1: Enrichment plot: HALLMARK\_MTORC1\_SIGNALING      
 Profile of the Running ES Score & Positions of GeneSet Members on the Rank Ordered List

  

| SYMBOL | TITLE | RANK IN GENE LIST | RANK METRIC SCORE | RUNNING ES | CORE ENRICHMENT || 1 | FGL2 | fibrinogen like 2 [Source:HGNC Symbol;Acc:HGNC:3696] | 44 | 2.152 | 0.0370 | No |
| 2 | CTSC | cathepsin C [Source:HGNC Symbol;Acc:HGNC:2528] | 566 | 1.018 | 0.0222 | No |
| 3 | GLRX | glutaredoxin [Source:HGNC Symbol;Acc:HGNC:4330] | 694 | 0.937 | 0.0313 | No |
| 4 | TRIB3 | tribbles pseudokinase 3 [Source:HGNC Symbol;Acc:HGNC:16228] | 901 | 0.841 | 0.0336 | No |
| 5 | PNP | purine nucleoside phosphorylase [Source:HGNC Symbol;Acc:HGNC:7892] | 902 | 0.841 | 0.0492 | No |
| 6 | SYTL2 | synaptotagmin like 2 [Source:HGNC Symbol;Acc:HGNC:15585] | 1051 | 0.789 | 0.0542 | No |
| 7 | IGFBP5 | insulin like growth factor binding protein 5 [Source:HGNC Symbol;Acc:HGNC:5474] | 1124 | 0.762 | 0.0637 | No |
| 8 | NAMPT | nicotinamide phosphoribosyltransferase [Source:HGNC Symbol;Acc:HGNC:30092] | 1163 | 0.744 | 0.0750 | No |
| 9 | CFP | complement factor properdin [Source:HGNC Symbol;Acc:HGNC:8864] | 1425 | 0.652 | 0.0702 | No |
| 10 | ITGB2 | integrin subunit beta 2 [Source:HGNC Symbol;Acc:HGNC:6155] | 1632 | 0.585 | 0.0677 | No |
| 11 | PSMC6 | "proteasome 26S subunit, ATPase 6 [Source:HGNC Symbol;Acc:HGNC:9553]" | 1677 | 0.574 | 0.0755 | No |
| 12 | SLA | Src like adaptor [Source:HGNC Symbol;Acc:HGNC:10902] | 1841 | 0.535 | 0.0749 | No |
| 13 | IFRD1 | interferon related developmental regulator 1 [Source:HGNC Symbol;Acc:HGNC:5456] | 1881 | 0.528 | 0.0821 | No |
| 14 | CORO1A | coronin 1A [Source:HGNC Symbol;Acc:HGNC:2252] | 1901 | 0.525 | 0.0906 | No |
| 15 | BCAT1 | branched chain amino acid transaminase 1 [Source:HGNC Symbol;Acc:HGNC:976] | 1979 | 0.507 | 0.0950 | No |
| 16 | SLC9A3R1 | SLC9A3 regulator 1 [Source:HGNC Symbol;Acc:HGNC:11075] | 2348 | 0.460 | 0.0798 | No |
| 17 | ACTR3 | actin related protein 3 [Source:HGNC Symbol;Acc:HGNC:170] | 2582 | 0.436 | 0.0728 | No |
| 18 | NUFIP1 | nuclear FMR1 interacting protein 1 [Source:HGNC Symbol;Acc:HGNC:8057] | 2635 | 0.428 | 0.0773 | No |
| 19 | BUB1 | BUB1 mitotic checkpoint serine/threonine kinase [Source:HGNC Symbol;Acc:HGNC:1148] | 2974 | 0.409 | 0.0631 | No |
| 20 | PSMG1 | proteasome assembly chaperone 1 [Source:HGNC Symbol;Acc:HGNC:3043] | 3053 | 0.394 | 0.0653 | No |
| 21 | POLR3G | RNA polymerase III subunit G [Source:HGNC Symbol;Acc:HGNC:30075] | 3132 | 0.383 | 0.0674 | No |
| 22 | PSPH | phosphoserine phosphatase [Source:HGNC Symbol;Acc:HGNC:9577] | 3146 | 0.381 | 0.0736 | No |
| 23 | MCM4 | minichromosome maintenance complex component 4 [Source:HGNC Symbol;Acc:HGNC:6947] | 3229 | 0.369 | 0.0751 | No |
| 24 | CTH | cystathionine gamma-lyase [Source:HGNC Symbol;Acc:HGNC:2501] | 3305 | 0.356 | 0.0769 | No |
| 25 | BTG2 | BTG anti-proliferation factor 2 [Source:HGNC Symbol;Acc:HGNC:1131] | 3319 | 0.354 | 0.0826 | No |
| 26 | PSMA3 | proteasome 20S subunit alpha 3 [Source:HGNC Symbol;Acc:HGNC:9532] | 3335 | 0.352 | 0.0881 | No |
| 27 | SLC2A3 | solute carrier family 2 member 3 [Source:HGNC Symbol;Acc:HGNC:11007] | 3342 | 0.351 | 0.0942 | No |
| 28 | SC5D | sterol-C5-desaturase [Source:HGNC Symbol;Acc:HGNC:10547] | 3356 | 0.349 | 0.0998 | No |
| 29 | ACTR2 | actin related protein 2 [Source:HGNC Symbol;Acc:HGNC:169] | 3441 | 0.336 | 0.1006 | No |
| 30 | GLA | galactosidase alpha [Source:HGNC Symbol;Acc:HGNC:4296] | 3461 | 0.333 | 0.1056 | No |
| 31 | IFI30 | IFI30 lysosomal thiol reductase [Source:HGNC Symbol;Acc:HGNC:5398] | 3485 | 0.330 | 0.1102 | No |
| 32 | UCHL5 | ubiquitin C-terminal hydrolase L5 [Source:HGNC Symbol;Acc:HGNC:19678] | 3634 | 0.309 | 0.1064 | No |
| 33 | ATP6V1D | ATPase H+ transporting V1 subunit D [Source:HGNC Symbol;Acc:HGNC:13527] | 3675 | 0.304 | 0.1094 | No |
| 34 | NFIL3 | "nuclear factor, interleukin 3 regulated [Source:HGNC Symbol;Acc:HGNC:7787]" | 3734 | 0.299 | 0.1112 | No |
| 35 | GSK3B | glycogen synthase kinase 3 beta [Source:HGNC Symbol;Acc:HGNC:4617] | 3768 | 0.294 | 0.1145 | No |
| 36 | ASNS | asparagine synthetase (glutamine-hydrolyzing) [Source:HGNC Symbol;Acc:HGNC:753] | 3870 | 0.282 | 0.1132 | No |
| 37 | RRM2 | ribonucleotide reductase regulatory subunit M2 [Source:HGNC Symbol;Acc:HGNC:10452] | 3983 | 0.267 | 0.1109 | No |
| 38 | LGMN | legumain [Source:HGNC Symbol;Acc:HGNC:9472] | 4001 | 0.265 | 0.1147 | No |
| 39 | ETF1 | eukaryotic translation termination factor 1 [Source:HGNC Symbol;Acc:HGNC:3477] | 4021 | 0.263 | 0.1184 | No |
| 40 | CACYBP | calcyclin binding protein [Source:HGNC Symbol;Acc:HGNC:30423] | 4040 | 0.260 | 0.1220 | No |
| 41 | TBK1 | TANK binding kinase 1 [Source:HGNC Symbol;Acc:HGNC:11584] | 4121 | 0.252 | 0.1215 | No |
| 42 | PSMA4 | proteasome 20S subunit alpha 4 [Source:HGNC Symbol;Acc:HGNC:9533] | 4124 | 0.252 | 0.1260 | No |
| 43 | NIBAN1 | niban apoptosis regulator 1 [Source:HGNC Symbol;Acc:HGNC:16784] | 4191 | 0.245 | 0.1263 | No |
| 44 | TM7SF2 | transmembrane 7 superfamily member 2 [Source:HGNC Symbol;Acc:HGNC:11863] | 4337 | 0.229 | 0.1212 | No |
| 45 | DDIT3 | DNA damage inducible transcript 3 [Source:HGNC Symbol;Acc:HGNC:2726] | 4509 | 0.212 | 0.1140 | No |
| 46 | DAPP1 | dual adaptor of phosphotyrosine and 3-phosphoinositides 1 [Source:HGNC Symbol;Acc:HGNC:16500] | 4588 | 0.206 | 0.1128 | No |
| 47 | TUBA4A | tubulin alpha 4a [Source:HGNC Symbol;Acc:HGNC:12407] | 4589 | 0.206 | 0.1166 | No |
| 48 | GSR | glutathione-disulfide reductase [Source:HGNC Symbol;Acc:HGNC:4623] | 4657 | 0.200 | 0.1160 | No |
| 49 | CCNF | cyclin F [Source:HGNC Symbol;Acc:HGNC:1591] | 4750 | 0.192 | 0.1136 | No |
| 50 | SLC1A5 | solute carrier family 1 member 5 [Source:HGNC Symbol;Acc:HGNC:10943] | 5126 | 0.156 | 0.0923 | No |
| 51 | RDH11 | retinol dehydrogenase 11 [Source:HGNC Symbol;Acc:HGNC:17964] | 5129 | 0.155 | 0.0950 | No |
| 52 | COPS5 | COP9 signalosome subunit 5 [Source:HGNC Symbol;Acc:HGNC:2240] | 5186 | 0.152 | 0.0942 | No |
| 53 | M6PR | "mannose-6-phosphate receptor, cation dependent [Source:HGNC Symbol;Acc:HGNC:6752]" | 5200 | 0.151 | 0.0962 | No |
| 54 | PPA1 | inorganic pyrophosphatase 1 [Source:HGNC Symbol;Acc:HGNC:9226] | 5217 | 0.150 | 0.0979 | No |
| 55 | ELOVL6 | ELOVL fatty acid elongase 6 [Source:HGNC Symbol;Acc:HGNC:15829] | 5498 | 0.127 | 0.0822 | No |
| 56 | SLC7A11 | solute carrier family 7 member 11 [Source:HGNC Symbol;Acc:HGNC:11059] | 5535 | 0.125 | 0.0821 | No |
| 57 | EBP | EBP cholestenol delta-isomerase [Source:HGNC Symbol;Acc:HGNC:3133] | 5537 | 0.125 | 0.0844 | No |
| 58 | UBE2D3 | ubiquitin conjugating enzyme E2 D3 [Source:HGNC Symbol;Acc:HGNC:12476] | 5615 | 0.118 | 0.0816 | No |
| 59 | PSMB5 | proteasome 20S subunit beta 5 [Source:HGNC Symbol;Acc:HGNC:9542] | 5682 | 0.114 | 0.0794 | No |
| 60 | STARD4 | StAR related lipid transfer domain containing 4 [Source:HGNC Symbol;Acc:HGNC:18058] | 5748 | 0.109 | 0.0773 | No |
| 61 | HSPA9 | heat shock protein family A (Hsp70) member 9 [Source:HGNC Symbol;Acc:HGNC:5244] | 5752 | 0.109 | 0.0791 | No |
| 62 | EPRS1 | glutamyl-prolyl-tRNA synthetase 1 [Source:HGNC Symbol;Acc:HGNC:3418] | 5774 | 0.107 | 0.0797 | No |
| 63 | PSAT1 | phosphoserine aminotransferase 1 [Source:HGNC Symbol;Acc:HGNC:19129] | 5776 | 0.107 | 0.0816 | No |
| 64 | AURKA | aurora kinase A [Source:HGNC Symbol;Acc:HGNC:11393] | 5931 | 0.097 | 0.0734 | No |
| 65 | PSMD13 | "proteasome 26S subunit, non-ATPase 13 [Source:HGNC Symbol;Acc:HGNC:9558]" | 6112 | 0.082 | 0.0633 | No |
| 66 | PLK1 | polo like kinase 1 [Source:HGNC Symbol;Acc:HGNC:9077] | 6259 | 0.073 | 0.0552 | No |
| 67 | HSPA4 | heat shock protein family A (Hsp70) member 4 [Source:HGNC Symbol;Acc:HGNC:5237] | 6279 | 0.071 | 0.0553 | No |
| 68 | NFKBIB | NFKB inhibitor beta [Source:HGNC Symbol;Acc:HGNC:7798] | 6288 | 0.071 | 0.0561 | No |
| 69 | WARS1 | tryptophanyl-tRNA synthetase 1 [Source:HGNC Symbol;Acc:HGNC:12729] | 6325 | 0.068 | 0.0551 | No |
| 70 | TFRC | transferrin receptor [Source:HGNC Symbol;Acc:HGNC:11763] | 6327 | 0.068 | 0.0563 | No |
| 71 | TCEA1 | transcription elongation factor A1 [Source:HGNC Symbol;Acc:HGNC:11612] | 6416 | 0.061 | 0.0517 | No |
| 72 | HSPD1 | heat shock protein family D (Hsp60) member 1 [Source:HGNC Symbol;Acc:HGNC:5261] | 6586 | 0.050 | 0.0417 | No |
| 73 | PSMD14 | "proteasome 26S subunit, non-ATPase 14 [Source:HGNC Symbol;Acc:HGNC:16889]" | 6687 | 0.043 | 0.0360 | No |
| 74 | PHGDH | phosphoglycerate dehydrogenase [Source:HGNC Symbol;Acc:HGNC:8923] | 6699 | 0.043 | 0.0361 | No |
| 75 | ACACA | acetyl-CoA carboxylase alpha [Source:HGNC Symbol;Acc:HGNC:84] | 6836 | 0.034 | 0.0280 | No |
| 76 | PSMC2 | "proteasome 26S subunit, ATPase 2 [Source:HGNC Symbol;Acc:HGNC:9548]" | 6841 | 0.034 | 0.0283 | No |
| 77 | HMGCR | 3-hydroxy-3-methylglutaryl-CoA reductase [Source:HGNC Symbol;Acc:HGNC:5006] | 6855 | 0.033 | 0.0281 | No |
| 78 | HMGCS1 | 3-hydroxy-3-methylglutaryl-CoA synthase 1 [Source:HGNC Symbol;Acc:HGNC:5007] | 6875 | 0.031 | 0.0274 | No |
| 79 | ME1 | malic enzyme 1 [Source:HGNC Symbol;Acc:HGNC:6983] | 6909 | 0.029 | 0.0258 | No |
| 80 | EDEM1 | ER degradation enhancing alpha-mannosidase like protein 1 [Source:HGNC Symbol;Acc:HGNC:18967] | 7078 | 0.019 | 0.0153 | No |
| 81 | ACSL3 | acyl-CoA synthetase long chain family member 3 [Source:HGNC Symbol;Acc:HGNC:3570] | 7111 | 0.018 | 0.0136 | No |
| 82 | VLDLR | very low density lipoprotein receptor [Source:HGNC Symbol;Acc:HGNC:12698] | 7123 | 0.017 | 0.0132 | No |
| 83 | ERO1A | endoplasmic reticulum oxidoreductase 1 alpha [Source:HGNC Symbol;Acc:HGNC:13280] | 7181 | 0.013 | 0.0098 | No |
| 84 | MTHFD2L | methylenetetrahydrofolate dehydrogenase (NADP+ dependent) 2 like [Source:HGNC Symbol;Acc:HGNC:31865] | 7244 | 0.010 | 0.0059 | No |
| 85 | NUPR1 | "nuclear protein 1, transcriptional regulator [Source:HGNC Symbol;Acc:HGNC:29990]" | 7282 | 0.008 | 0.0037 | No |
| 86 | PLOD2 | "procollagen-lysine,2-oxoglutarate 5-dioxygenase 2 [Source:HGNC Symbol;Acc:HGNC:9082]" | 7291 | 0.007 | 0.0033 | No |
| 87 | SHMT2 | serine hydroxymethyltransferase 2 [Source:HGNC Symbol;Acc:HGNC:10852] | 7477 | -0.003 | -0.0086 | No |
| 88 | ARPC5L | actin related protein 2/3 complex subunit 5 like [Source:HGNC Symbol;Acc:HGNC:23366] | 7508 | -0.005 | -0.0104 | No |
| 89 | SLC37A4 | solute carrier family 37 member 4 [Source:HGNC Symbol;Acc:HGNC:4061] | 7567 | -0.008 | -0.0140 | No |
| 90 | YKT6 | YKT6 v-SNARE homolog [Source:HGNC Symbol;Acc:HGNC:16959] | 7588 | -0.008 | -0.0152 | No |
| 91 | MTHFD2 | "methylenetetrahydrofolate dehydrogenase (NADP+ dependent) 2, methenyltetrahydrofolate cyclohydrolase [Source:HGNC Symbol;Acc:HGNC:7434]" | 7932 | -0.025 | -0.0369 | No |
| 92 | SLC7A5 | solute carrier family 7 member 5 [Source:HGNC Symbol;Acc:HGNC:11063] | 7939 | -0.026 | -0.0368 | No |
| 93 | EIF2S2 | eukaryotic translation initiation factor 2 subunit beta [Source:HGNC Symbol;Acc:HGNC:3266] | 7960 | -0.027 | -0.0376 | No |
| 94 | PITPNB | phosphatidylinositol transfer protein beta [Source:HGNC Symbol;Acc:HGNC:9002] | 7964 | -0.027 | -0.0373 | No |
| 95 | TES | testin LIM domain protein [Source:HGNC Symbol;Acc:HGNC:14620] | 7996 | -0.029 | -0.0388 | No |
| 96 | IDH1 | isocitrate dehydrogenase (NADP(+)) 1 [Source:HGNC Symbol;Acc:HGNC:5382] | 8014 | -0.030 | -0.0393 | No |
| 97 | PPP1R15A | protein phosphatase 1 regulatory subunit 15A [Source:HGNC Symbol;Acc:HGNC:14375] | 8019 | -0.030 | -0.0390 | No |
| 98 | PNO1 | partner of NOB1 homolog [Source:HGNC Symbol;Acc:HGNC:32790] | 8121 | -0.036 | -0.0449 | No |
| 99 | RAB1A | "RAB1A, member RAS oncogene family [Source:HGNC Symbol;Acc:HGNC:9758]" | 8135 | -0.037 | -0.0450 | No |
| 100 | HSPE1 | heat shock protein family E (Hsp10) member 1 [Source:HGNC Symbol;Acc:HGNC:5269] | 8177 | -0.039 | -0.0470 | No |
| 101 | MAP2K3 | mitogen-activated protein kinase kinase 3 [Source:HGNC Symbol;Acc:HGNC:6843] | 8186 | -0.039 | -0.0467 | No |
| 102 | SDF2L1 | stromal cell derived factor 2 like 1 [Source:HGNC Symbol;Acc:HGNC:10676] | 8230 | -0.042 | -0.0488 | No |
| 103 | NUP205 | nucleoporin 205 [Source:HGNC Symbol;Acc:HGNC:18658] | 8568 | -0.058 | -0.0695 | No |
| 104 | CCT6A | chaperonin containing TCP1 subunit 6A [Source:HGNC Symbol;Acc:HGNC:1620] | 8611 | -0.061 | -0.0710 | No |
| 105 | PRDX1 | peroxiredoxin 1 [Source:HGNC Symbol;Acc:HGNC:9352] | 8653 | -0.064 | -0.0725 | No |
| 106 | PSMD12 | "proteasome 26S subunit, non-ATPase 12 [Source:HGNC Symbol;Acc:HGNC:9557]" | 8658 | -0.064 | -0.0716 | No |
| 107 | GTF2H1 | general transcription factor IIH subunit 1 [Source:HGNC Symbol;Acc:HGNC:4655] | 8752 | -0.069 | -0.0763 | No |
| 108 | CDC25A | cell division cycle 25A [Source:HGNC Symbol;Acc:HGNC:1725] | 8887 | -0.077 | -0.0835 | No |
| 109 | DHCR7 | 7-dehydrocholesterol reductase [Source:HGNC Symbol;Acc:HGNC:2860] | 8984 | -0.082 | -0.0882 | No |
| 110 | ACLY | ATP citrate lyase [Source:HGNC Symbol;Acc:HGNC:115] | 9034 | -0.084 | -0.0898 | No |
| 111 | IMMT | inner membrane mitochondrial protein [Source:HGNC Symbol;Acc:HGNC:6047] | 9073 | -0.086 | -0.0907 | No |
| 112 | GGA2 | "golgi associated, gamma adaptin ear containing, ARF binding protein 2 [Source:HGNC Symbol;Acc:HGNC:16064]" | 9082 | -0.087 | -0.0896 | No |
| 113 | ADIPOR2 | adiponectin receptor 2 [Source:HGNC Symbol;Acc:HGNC:24041] | 9231 | -0.094 | -0.0974 | No |
| 114 | PSME3 | proteasome activator subunit 3 [Source:HGNC Symbol;Acc:HGNC:9570] | 9390 | -0.104 | -0.1057 | No |
| 115 | HSP90B1 | heat shock protein 90 beta family member 1 [Source:HGNC Symbol;Acc:HGNC:12028] | 9406 | -0.105 | -0.1048 | No |
| 116 | HPRT1 | hypoxanthine phosphoribosyltransferase 1 [Source:HGNC Symbol;Acc:HGNC:5157] | 9568 | -0.116 | -0.1130 | No |
| 117 | IDI1 | isopentenyl-diphosphate delta isomerase 1 [Source:HGNC Symbol;Acc:HGNC:5387] | 9590 | -0.117 | -0.1122 | No |
| 118 | CYB5B | cytochrome b5 type B [Source:HGNC Symbol;Acc:HGNC:24374] | 9671 | -0.123 | -0.1151 | No |
| 119 | CXCR4 | C-X-C motif chemokine receptor 4 [Source:HGNC Symbol;Acc:HGNC:2561] | 9672 | -0.123 | -0.1128 | No |
| 120 | CYP51A1 | cytochrome P450 family 51 subfamily A member 1 [Source:HGNC Symbol;Acc:HGNC:2649] | 9718 | -0.125 | -0.1134 | No |
| 121 | GCLC | glutamate-cysteine ligase catalytic subunit [Source:HGNC Symbol;Acc:HGNC:4311] | 9762 | -0.129 | -0.1138 | No |
| 122 | DDX39A | DExD-box helicase 39A [Source:HGNC Symbol;Acc:HGNC:17821] | 9774 | -0.129 | -0.1121 | No |
| 123 | LTA4H | leukotriene A4 hydrolase [Source:HGNC Symbol;Acc:HGNC:6710] | 9826 | -0.132 | -0.1130 | No |
| 124 | UFM1 | ubiquitin fold modifier 1 [Source:HGNC Symbol;Acc:HGNC:20597] | 9965 | -0.140 | -0.1193 | No |
| 125 | TXNRD1 | thioredoxin reductase 1 [Source:HGNC Symbol;Acc:HGNC:12437] | 10031 | -0.144 | -0.1209 | No |
| 126 | RPA1 | replication protein A1 [Source:HGNC Symbol;Acc:HGNC:10289] | 10247 | -0.156 | -0.1319 | No |
| 127 | PSMC4 | "proteasome 26S subunit, ATPase 4 [Source:HGNC Symbol;Acc:HGNC:9551]" | 10382 | -0.165 | -0.1375 | No |
| 128 | TPI1 | triosephosphate isomerase 1 [Source:HGNC Symbol;Acc:HGNC:12009] | 10457 | -0.169 | -0.1391 | No |
| 129 | GMPS | guanine monophosphate synthase [Source:HGNC Symbol;Acc:HGNC:4378] | 10459 | -0.169 | -0.1361 | No |
| 130 | ABCF2 | ATP binding cassette subfamily F member 2 [Source:HGNC Symbol;Acc:HGNC:71] | 10485 | -0.171 | -0.1345 | No |
| 131 | PIK3R3 | phosphoinositide-3-kinase regulatory subunit 3 [Source:HGNC Symbol;Acc:HGNC:8981] | 10512 | -0.172 | -0.1330 | No |
| 132 | HMBS | hydroxymethylbilane synthase [Source:HGNC Symbol;Acc:HGNC:4982] | 10518 | -0.173 | -0.1301 | No |
| 133 | STIP1 | stress induced phosphoprotein 1 [Source:HGNC Symbol;Acc:HGNC:11387] | 10650 | -0.180 | -0.1353 | No |
| 134 | G6PD | glucose-6-phosphate dehydrogenase [Source:HGNC Symbol;Acc:HGNC:4057] | 10710 | -0.183 | -0.1357 | No |
| 135 | MCM2 | minichromosome maintenance complex component 2 [Source:HGNC Symbol;Acc:HGNC:6944] | 10713 | -0.183 | -0.1324 | No |
| 136 | NMT1 | N-myristoyltransferase 1 [Source:HGNC Symbol;Acc:HGNC:7857] | 10801 | -0.188 | -0.1346 | No |
| 137 | GBE1 | "1,4-alpha-glucan branching enzyme 1 [Source:HGNC Symbol;Acc:HGNC:4180]" | 10822 | -0.189 | -0.1324 | No |
| 138 | GAPDH | glyceraldehyde-3-phosphate dehydrogenase [Source:HGNC Symbol;Acc:HGNC:4141] | 10875 | -0.193 | -0.1322 | No |
| 139 | EEF1E1 | eukaryotic translation elongation factor 1 epsilon 1 [Source:HGNC Symbol;Acc:HGNC:3212] | 10881 | -0.193 | -0.1289 | No |
| 140 | SERP1 | stress associated endoplasmic reticulum protein 1 [Source:HGNC Symbol;Acc:HGNC:10759] | 10912 | -0.195 | -0.1272 | No |
| 141 | SQLE | squalene epoxidase [Source:HGNC Symbol;Acc:HGNC:11279] | 10986 | -0.200 | -0.1282 | No |
| 142 | ATP2A2 | ATPase sarcoplasmic/endoplasmic reticulum Ca2+ transporting 2 [Source:HGNC Symbol;Acc:HGNC:812] | 11020 | -0.201 | -0.1267 | No |
| 143 | SKAP2 | src kinase associated phosphoprotein 2 [Source:HGNC Symbol;Acc:HGNC:15687] | 11269 | -0.215 | -0.1387 | No |
| 144 | HK2 | hexokinase 2 [Source:HGNC Symbol;Acc:HGNC:4923] | 11425 | -0.225 | -0.1445 | No |
| 145 | UNG | uracil DNA glycosylase [Source:HGNC Symbol;Acc:HGNC:12572] | 11714 | -0.242 | -0.1587 | No |
| 146 | NFYC | nuclear transcription factor Y subunit gamma [Source:HGNC Symbol;Acc:HGNC:7806] | 11738 | -0.243 | -0.1557 | No |
| 147 | SORD | sorbitol dehydrogenase [Source:HGNC Symbol;Acc:HGNC:11184] | 11863 | -0.252 | -0.1590 | Yes |
| 148 | PDAP1 | PDGFA associated protein 1 [Source:HGNC Symbol;Acc:HGNC:14634] | 11879 | -0.252 | -0.1553 | Yes |
| 149 | SRD5A1 | steroid 5 alpha-reductase 1 [Source:HGNC Symbol;Acc:HGNC:11284] | 11962 | -0.258 | -0.1558 | Yes |
| 150 | SSR1 | signal sequence receptor subunit 1 [Source:HGNC Symbol;Acc:HGNC:11323] | 12057 | -0.264 | -0.1570 | Yes |
| 151 | PGM1 | phosphoglucomutase 1 [Source:HGNC Symbol;Acc:HGNC:8905] | 12099 | -0.267 | -0.1547 | Yes |
| 152 | DHCR24 | 24-dehydrocholesterol reductase [Source:HGNC Symbol;Acc:HGNC:2859] | 12239 | -0.276 | -0.1586 | Yes |
| 153 | FDXR | ferredoxin reductase [Source:HGNC Symbol;Acc:HGNC:3642] | 12240 | -0.277 | -0.1535 | Yes |
| 154 | AK4 | adenylate kinase 4 [Source:HGNC Symbol;Acc:HGNC:363] | 12258 | -0.278 | -0.1494 | Yes |
| 155 | SEC11A | "SEC11 homolog A, signal peptidase complex subunit [Source:HGNC Symbol;Acc:HGNC:17718]" | 12278 | -0.279 | -0.1455 | Yes |
| 156 | TOMM40 | translocase of outer mitochondrial membrane 40 [Source:HGNC Symbol;Acc:HGNC:18001] | 12284 | -0.280 | -0.1406 | Yes |
| 157 | CANX | calnexin [Source:HGNC Symbol;Acc:HGNC:1473] | 12536 | -0.299 | -0.1513 | Yes |
| 158 | HSPA5 | heat shock protein family A (Hsp70) member 5 [Source:HGNC Symbol;Acc:HGNC:5238] | 12586 | -0.303 | -0.1489 | Yes |
| 159 | ALDOA | "aldolase, fructose-bisphosphate A [Source:HGNC Symbol;Acc:HGNC:414]" | 12634 | -0.306 | -0.1462 | Yes |
| 160 | PPIA | peptidylprolyl isomerase A [Source:HGNC Symbol;Acc:HGNC:9253] | 12733 | -0.314 | -0.1468 | Yes |
| 161 | ADD3 | adducin 3 [Source:HGNC Symbol;Acc:HGNC:245] | 12843 | -0.323 | -0.1478 | Yes |
| 162 | CALR | calreticulin [Source:HGNC Symbol;Acc:HGNC:1455] | 12856 | -0.324 | -0.1426 | Yes |
| 163 | GOT1 | glutamic-oxaloacetic transaminase 1 [Source:HGNC Symbol;Acc:HGNC:4432] | 12864 | -0.325 | -0.1370 | Yes |
| 164 | ELOVL5 | ELOVL fatty acid elongase 5 [Source:HGNC Symbol;Acc:HGNC:21308] | 12900 | -0.328 | -0.1332 | Yes |
| 165 | TMEM97 | transmembrane protein 97 [Source:HGNC Symbol;Acc:HGNC:28106] | 12936 | -0.332 | -0.1293 | Yes |
| 166 | RRP9 | "ribosomal RNA processing 9, U3 small nucleolar RNA binding protein [Source:HGNC Symbol;Acc:HGNC:16829]" | 12955 | -0.334 | -0.1243 | Yes |
| 167 | TUBG1 | tubulin gamma 1 [Source:HGNC Symbol;Acc:HGNC:12417] | 12998 | -0.338 | -0.1208 | Yes |
| 168 | QDPR | quinoid dihydropteridine reductase [Source:HGNC Symbol;Acc:HGNC:9752] | 13019 | -0.339 | -0.1158 | Yes |
| 169 | ATP5MC1 | ATP synthase membrane subunit c locus 1 [Source:HGNC Symbol;Acc:HGNC:841] | 13021 | -0.340 | -0.1096 | Yes |
| 170 | SCD | stearoyl-CoA desaturase [Source:HGNC Symbol;Acc:HGNC:10571] | 13043 | -0.342 | -0.1046 | Yes |
| 171 | RPN1 | ribophorin I [Source:HGNC Symbol;Acc:HGNC:10381] | 13083 | -0.346 | -0.1007 | Yes |
| 172 | ENO1 | enolase 1 [Source:HGNC Symbol;Acc:HGNC:3350] | 13088 | -0.346 | -0.0945 | Yes |
| 173 | SERPINH1 | serpin family H member 1 [Source:HGNC Symbol;Acc:HGNC:1546] | 13150 | -0.352 | -0.0920 | Yes |
| 174 | LDHA | lactate dehydrogenase A [Source:HGNC Symbol;Acc:HGNC:6535] | 13197 | -0.356 | -0.0884 | Yes |
| 175 | USO1 | USO1 vesicle transport factor [Source:HGNC Symbol;Acc:HGNC:30904] | 13216 | -0.358 | -0.0829 | Yes |
| 176 | P4HA1 | prolyl 4-hydroxylase subunit alpha 1 [Source:HGNC Symbol;Acc:HGNC:8546] | 13262 | -0.362 | -0.0791 | Yes |
| 177 | SLC6A6 | solute carrier family 6 member 6 [Source:HGNC Symbol;Acc:HGNC:11052] | 13327 | -0.370 | -0.0764 | Yes |
| 178 | CD9 | CD9 molecule [Source:HGNC Symbol;Acc:HGNC:1709] | 13472 | -0.388 | -0.0785 | Yes |
| 179 | FADS1 | fatty acid desaturase 1 [Source:HGNC Symbol;Acc:HGNC:3574] | 13531 | -0.396 | -0.0749 | Yes |
| 180 | FADS2 | fatty acid desaturase 2 [Source:HGNC Symbol;Acc:HGNC:3575] | 13558 | -0.400 | -0.0692 | Yes |
| 181 | FKBP2 | FKBP prolyl isomerase 2 [Source:HGNC Symbol;Acc:HGNC:3718] | 13601 | -0.405 | -0.0644 | Yes |
| 182 | SQSTM1 | sequestosome 1 [Source:HGNC Symbol;Acc:HGNC:11280] | 13604 | -0.406 | -0.0570 | Yes |
| 183 | XBP1 | X-box binding protein 1 [Source:HGNC Symbol;Acc:HGNC:12801] | 13607 | -0.406 | -0.0497 | Yes |
| 184 | INSIG1 | insulin induced gene 1 [Source:HGNC Symbol;Acc:HGNC:6083] | 13987 | -0.430 | -0.0662 | Yes |
| 185 | BHLHE40 | basic helix-loop-helix family member e40 [Source:HGNC Symbol;Acc:HGNC:1046] | 14032 | -0.437 | -0.0609 | Yes |
| 186 | GPI | glucose-6-phosphate isomerase [Source:HGNC Symbol;Acc:HGNC:4458] | 14095 | -0.448 | -0.0567 | Yes |
| 187 | LDLR | low density lipoprotein receptor [Source:HGNC Symbol;Acc:HGNC:6547] | 14103 | -0.450 | -0.0488 | Yes |
| 188 | RIT1 | Ras like without CAAX 1 [Source:HGNC Symbol;Acc:HGNC:10023] | 14133 | -0.454 | -0.0422 | Yes |
| 189 | PGK1 | phosphoglycerate kinase 1 [Source:HGNC Symbol;Acc:HGNC:8896] | 14157 | -0.458 | -0.0352 | Yes |
| 190 | PFKL | "phosphofructokinase, liver type [Source:HGNC Symbol;Acc:HGNC:8876]" | 14344 | -0.485 | -0.0383 | Yes |
| 191 | PDK1 | pyruvate dehydrogenase kinase 1 [Source:HGNC Symbol;Acc:HGNC:8809] | 14661 | -0.544 | -0.0486 | Yes |
| 192 | CCNG1 | cyclin G1 [Source:HGNC Symbol;Acc:HGNC:1592] | 14944 | -0.640 | -0.0550 | Yes |
| 193 | SLC2A1 | solute carrier family 2 member 1 [Source:HGNC Symbol;Acc:HGNC:11005] | 14992 | -0.661 | -0.0458 | Yes |
| 194 | EGLN3 | egl-9 family hypoxia inducible factor 3 [Source:HGNC Symbol;Acc:HGNC:14661] | 14996 | -0.663 | -0.0337 | Yes |
| 195 | MLLT11 | MLLT11 transcription factor 7 cofactor [Source:HGNC Symbol;Acc:HGNC:16997] | 15011 | -0.668 | -0.0222 | Yes |
| 196 | SLC1A4 | solute carrier family 1 member 4 [Source:HGNC Symbol;Acc:HGNC:10942] | 15030 | -0.677 | -0.0109 | Yes |
| 197 | DDIT4 | DNA damage inducible transcript 4 [Source:HGNC Symbol;Acc:HGNC:24944] | 15359 | -0.865 | -0.0160 | Yes |
| 198 | CDKN1A | cyclin dependent kinase inhibitor 1A [Source:HGNC Symbol;Acc:HGNC:1784] | 15428 | -0.929 | -0.0032 | Yes |
| 199 | STC1 | stanniocalcin 1 [Source:HGNC Symbol;Acc:HGNC:11373] | 15482 | -1.009 | 0.0120 | Yes |
Table: GSEA details [plain text format]

  

Fig 2: HALLMARK\_MTORC1\_SIGNALING      
 Blue-Pink O' Gram in the Space of the Analyzed GeneSet

  

Fig 3: HALLMARK\_MTORC1\_SIGNALING: Random ES distribution      
 Gene set null distribution of ES for **HALLMARK\_MTORC1\_SIGNALING**
