# Supplementary material for: In vivo CRISPR screens reveal Serpinb9 and Adam2 as regulators of immune therapy response in lung cancer
Source: Nat Commun. 2023 May 31;14:3150. doi: 10.1038/s41467-023-38841-7 (PMC10232477; doi:10.1038/s41467-023-38841-7)
Supplement: Supplementary file 13 — Source Data [file 41467_2023_38841_MOESM13_ESM.zip › Figure 4E.zip!/my_analysis.Gsea.1640146340255_Hallmark/HALLMARK_PROTEIN_SECRETION.html]

Details for gene set HALLMARK\_PROTEIN\_SECRETION[GSEA]

|  || Dataset | December 21, 2021\_for GSEA run\_collapsed\_to\_symbols.GSEA\_B6\_NI.cls #CTLR\_NI\_versus\_ADAM2\_NI |
| Phenotype | GSEA\_B6\_NI.cls#CTLR\_NI\_versus\_ADAM2\_NI |
| Upregulated in class | ADAM2\_NI |
| GeneSet | HALLMARK\_PROTEIN\_SECRETION |
| Enrichment Score (ES) | -0.22148798 |
| Normalized Enrichment Score (NES) | -0.85073596 |
| Nominal p-value | 0.53831416 |
| FDR q-value | 0.88647383 |
| FWER p-Value | 1.0 |
Table: GSEA Results Summary

  

Fig 1: Enrichment plot: HALLMARK\_PROTEIN\_SECRETION      
 Profile of the Running ES Score & Positions of GeneSet Members on the Rank Ordered List

  

| SYMBOL | TITLE | RANK IN GENE LIST | RANK METRIC SCORE | RUNNING ES | CORE ENRICHMENT || 1 | CTSC | cathepsin C [Source:HGNC Symbol;Acc:HGNC:2528] | 566 | 1.018 | 0.0076 | No |
| 2 | ABCA1 | ATP binding cassette subfamily A member 1 [Source:HGNC Symbol;Acc:HGNC:29] | 717 | 0.923 | 0.0378 | No |
| 3 | SSPN | sarcospan [Source:HGNC Symbol;Acc:HGNC:11322] | 1331 | 0.684 | 0.0280 | No |
| 4 | CLN5 | CLN5 intracellular trafficking protein [Source:HGNC Symbol;Acc:HGNC:2076] | 1975 | 0.508 | 0.0086 | No |
| 5 | ATP7A | ATPase copper transporting alpha [Source:HGNC Symbol;Acc:HGNC:869] | 2350 | 0.460 | 0.0044 | No |
| 6 | NAPG | NSF attachment protein gamma [Source:HGNC Symbol;Acc:HGNC:7642] | 2541 | 0.444 | 0.0114 | No |
| 7 | TOM1L1 | target of myb1 like 1 membrane trafficking protein [Source:HGNC Symbol;Acc:HGNC:11983] | 3125 | 0.384 | -0.0094 | No |
| 8 | ATP6V1B1 | ATPase H+ transporting V1 subunit B1 [Source:HGNC Symbol;Acc:HGNC:853] | 3295 | 0.358 | -0.0048 | No |
| 9 | VPS4B | vacuolar protein sorting 4 homolog B [Source:HGNC Symbol;Acc:HGNC:10895] | 3343 | 0.351 | 0.0073 | No |
| 10 | GLA | galactosidase alpha [Source:HGNC Symbol;Acc:HGNC:4296] | 3461 | 0.333 | 0.0142 | No |
| 11 | DNM1L | dynamin 1 like [Source:HGNC Symbol;Acc:HGNC:2973] | 3766 | 0.294 | 0.0073 | No |
| 12 | SNX2 | sorting nexin 2 [Source:HGNC Symbol;Acc:HGNC:11173] | 3825 | 0.288 | 0.0161 | No |
| 13 | TMX1 | thioredoxin related transmembrane protein 1 [Source:HGNC Symbol;Acc:HGNC:15487] | 4100 | 0.254 | 0.0094 | No |
| 14 | ARFGEF1 | ADP ribosylation factor guanine nucleotide exchange factor 1 [Source:HGNC Symbol;Acc:HGNC:15772] | 4217 | 0.241 | 0.0124 | No |
| 15 | TPD52 | tumor protein D52 [Source:HGNC Symbol;Acc:HGNC:12005] | 4259 | 0.236 | 0.0199 | No |
| 16 | STX7 | syntaxin 7 [Source:HGNC Symbol;Acc:HGNC:11442] | 4601 | 0.205 | 0.0069 | No |
| 17 | SNAP23 | synaptosome associated protein 23 [Source:HGNC Symbol;Acc:HGNC:11131] | 4900 | 0.178 | -0.0046 | No |
| 18 | AP1G1 | adaptor related protein complex 1 subunit gamma 1 [Source:HGNC Symbol;Acc:HGNC:555] | 5044 | 0.164 | -0.0067 | No |
| 19 | M6PR | "mannose-6-phosphate receptor, cation dependent [Source:HGNC Symbol;Acc:HGNC:6752]" | 5200 | 0.151 | -0.0101 | No |
| 20 | ATP6V1H | ATPase H+ transporting V1 subunit H [Source:HGNC Symbol;Acc:HGNC:18303] | 5288 | 0.143 | -0.0096 | No |
| 21 | CLTC | clathrin heavy chain [Source:HGNC Symbol;Acc:HGNC:2092] | 5312 | 0.141 | -0.0049 | No |
| 22 | ZW10 | zw10 kinetochore protein [Source:HGNC Symbol;Acc:HGNC:13194] | 5417 | 0.134 | -0.0059 | No |
| 23 | MAPK1 | mitogen-activated protein kinase 1 [Source:HGNC Symbol;Acc:HGNC:6871] | 5638 | 0.116 | -0.0150 | No |
| 24 | GNAS | GNAS complex locus [Source:HGNC Symbol;Acc:HGNC:4392] | 5922 | 0.097 | -0.0289 | No |
| 25 | PPT1 | palmitoyl-protein thioesterase 1 [Source:HGNC Symbol;Acc:HGNC:9325] | 5954 | 0.095 | -0.0268 | No |
| 26 | STAM | signal transducing adaptor molecule [Source:HGNC Symbol;Acc:HGNC:11357] | 5969 | 0.094 | -0.0237 | No |
| 27 | DST | dystonin [Source:HGNC Symbol;Acc:HGNC:1090] | 6192 | 0.077 | -0.0346 | No |
| 28 | AP3S1 | adaptor related protein complex 3 subunit sigma 1 [Source:HGNC Symbol;Acc:HGNC:2013] | 6312 | 0.069 | -0.0393 | No |
| 29 | VAMP4 | vesicle associated membrane protein 4 [Source:HGNC Symbol;Acc:HGNC:12645] | 6503 | 0.055 | -0.0491 | No |
| 30 | STX16 | syntaxin 16 [Source:HGNC Symbol;Acc:HGNC:11431] | 6701 | 0.043 | -0.0599 | No |
| 31 | LAMP2 | lysosomal associated membrane protein 2 [Source:HGNC Symbol;Acc:HGNC:6501] | 7018 | 0.023 | -0.0792 | No |
| 32 | RAB22A | "RAB22A, member RAS oncogene family [Source:HGNC Symbol;Acc:HGNC:9764]" | 7201 | 0.012 | -0.0904 | No |
| 33 | GOSR2 | golgi SNAP receptor complex member 2 [Source:HGNC Symbol;Acc:HGNC:4431] | 7476 | -0.003 | -0.1078 | No |
| 34 | YKT6 | YKT6 v-SNARE homolog [Source:HGNC Symbol;Acc:HGNC:16959] | 7588 | -0.008 | -0.1146 | No |
| 35 | ARFGEF2 | ADP ribosylation factor guanine nucleotide exchange factor 2 [Source:HGNC Symbol;Acc:HGNC:15853] | 7637 | -0.010 | -0.1172 | No |
| 36 | YIPF6 | Yip1 domain family member 6 [Source:HGNC Symbol;Acc:HGNC:28304] | 7719 | -0.015 | -0.1218 | No |
| 37 | COPB1 | COPI coat complex subunit beta 1 [Source:HGNC Symbol;Acc:HGNC:2231] | 8008 | -0.029 | -0.1390 | No |
| 38 | ADAM10 | ADAM metallopeptidase domain 10 [Source:HGNC Symbol;Acc:HGNC:188] | 8009 | -0.029 | -0.1378 | No |
| 39 | TSPAN8 | tetraspanin 8 [Source:HGNC Symbol;Acc:HGNC:11855] | 8180 | -0.039 | -0.1470 | No |
| 40 | RPS6KA3 | ribosomal protein S6 kinase A3 [Source:HGNC Symbol;Acc:HGNC:10432] | 8182 | -0.039 | -0.1454 | No |
| 41 | AP2S1 | adaptor related protein complex 2 subunit sigma 1 [Source:HGNC Symbol;Acc:HGNC:565] | 8497 | -0.054 | -0.1632 | No |
| 42 | GALC | galactosylceramidase [Source:HGNC Symbol;Acc:HGNC:4115] | 8607 | -0.061 | -0.1675 | No |
| 43 | AP3B1 | adaptor related protein complex 3 subunit beta 1 [Source:HGNC Symbol;Acc:HGNC:566] | 8655 | -0.064 | -0.1678 | No |
| 44 | TMED10 | transmembrane p24 trafficking protein 10 [Source:HGNC Symbol;Acc:HGNC:16998] | 8661 | -0.064 | -0.1654 | No |
| 45 | STX12 | syntaxin 12 [Source:HGNC Symbol;Acc:HGNC:11430] | 8710 | -0.068 | -0.1655 | No |
| 46 | TMED2 | transmembrane p24 trafficking protein 2 [Source:HGNC Symbol;Acc:HGNC:16996] | 8723 | -0.068 | -0.1633 | No |
| 47 | CLCN3 | chloride voltage-gated channel 3 [Source:HGNC Symbol;Acc:HGNC:2021] | 8758 | -0.070 | -0.1625 | No |
| 48 | LMAN1 | "lectin, mannose binding 1 [Source:HGNC Symbol;Acc:HGNC:6631]" | 8976 | -0.081 | -0.1729 | No |
| 49 | RAB5A | "RAB5A, member RAS oncogene family [Source:HGNC Symbol;Acc:HGNC:9783]" | 9039 | -0.084 | -0.1733 | No |
| 50 | COPB2 | COPI coat complex subunit beta 2 [Source:HGNC Symbol;Acc:HGNC:2232] | 9081 | -0.087 | -0.1722 | No |
| 51 | AP2M1 | adaptor related protein complex 2 subunit mu 1 [Source:HGNC Symbol;Acc:HGNC:564] | 9180 | -0.092 | -0.1745 | No |
| 52 | VAMP3 | vesicle associated membrane protein 3 [Source:HGNC Symbol;Acc:HGNC:12644] | 9217 | -0.094 | -0.1727 | No |
| 53 | KRT18 | keratin 18 [Source:HGNC Symbol;Acc:HGNC:6430] | 9222 | -0.094 | -0.1689 | No |
| 54 | EGFR | epidermal growth factor receptor [Source:HGNC Symbol;Acc:HGNC:3236] | 9513 | -0.111 | -0.1827 | No |
| 55 | CLTA | clathrin light chain A [Source:HGNC Symbol;Acc:HGNC:2090] | 9648 | -0.121 | -0.1861 | No |
| 56 | BET1 | Bet1 golgi vesicular membrane trafficking protein [Source:HGNC Symbol;Acc:HGNC:14562] | 9652 | -0.122 | -0.1810 | No |
| 57 | MON2 | "MON2 homolog, regulator of endosome-to-Golgi trafficking [Source:HGNC Symbol;Acc:HGNC:29177]" | 9840 | -0.133 | -0.1873 | No |
| 58 | ARFIP1 | ADP ribosylation factor interacting protein 1 [Source:HGNC Symbol;Acc:HGNC:21496] | 9944 | -0.139 | -0.1879 | No |
| 59 | RAB14 | "RAB14, member RAS oncogene family [Source:HGNC Symbol;Acc:HGNC:16524]" | 9976 | -0.141 | -0.1838 | No |
| 60 | ATP1A1 | ATPase Na+/K+ transporting subunit alpha 1 [Source:HGNC Symbol;Acc:HGNC:799] | 10045 | -0.145 | -0.1820 | No |
| 61 | ANP32E | acidic nuclear phosphoprotein 32 family member E [Source:HGNC Symbol;Acc:HGNC:16673] | 10109 | -0.149 | -0.1796 | No |
| 62 | ARCN1 | archain 1 [Source:HGNC Symbol;Acc:HGNC:649] | 10374 | -0.164 | -0.1895 | No |
| 63 | GOLGA4 | golgin A4 [Source:HGNC Symbol;Acc:HGNC:4427] | 10394 | -0.166 | -0.1835 | No |
| 64 | DOP1A | DOP1 leucine zipper like protein A [Source:HGNC Symbol;Acc:HGNC:21194] | 10400 | -0.166 | -0.1767 | No |
| 65 | TSG101 | tumor susceptibility 101 [Source:HGNC Symbol;Acc:HGNC:15971] | 10431 | -0.167 | -0.1714 | No |
| 66 | RAB9A | "RAB9A, member RAS oncogene family [Source:HGNC Symbol;Acc:HGNC:9792]" | 10576 | -0.176 | -0.1731 | No |
| 67 | SOD1 | superoxide dismutase 1 [Source:HGNC Symbol;Acc:HGNC:11179] | 10977 | -0.199 | -0.1902 | No |
| 68 | COG2 | component of oligomeric golgi complex 2 [Source:HGNC Symbol;Acc:HGNC:6546] | 11063 | -0.204 | -0.1868 | No |
| 69 | AP2B1 | adaptor related protein complex 2 subunit beta 1 [Source:HGNC Symbol;Acc:HGNC:563] | 11234 | -0.213 | -0.1885 | No |
| 70 | KIF1B | kinesin family member 1B [Source:HGNC Symbol;Acc:HGNC:16636] | 11428 | -0.225 | -0.1912 | No |
| 71 | CD63 | CD63 molecule [Source:HGNC Symbol;Acc:HGNC:1692] | 11507 | -0.230 | -0.1863 | No |
| 72 | ARF1 | ADP ribosylation factor 1 [Source:HGNC Symbol;Acc:HGNC:652] | 12056 | -0.264 | -0.2101 | Yes |
| 73 | RER1 | retention in endoplasmic reticulum sorting receptor 1 [Source:HGNC Symbol;Acc:HGNC:30309] | 12137 | -0.269 | -0.2036 | Yes |
| 74 | SEC22B | "SEC22 homolog B, vesicle trafficking protein [Source:HGNC Symbol;Acc:HGNC:10700]" | 12247 | -0.277 | -0.1986 | Yes |
| 75 | CAV2 | caveolin 2 [Source:HGNC Symbol;Acc:HGNC:1528] | 12448 | -0.292 | -0.1989 | Yes |
| 76 | IGF2R | insulin like growth factor 2 receptor [Source:HGNC Symbol;Acc:HGNC:5467] | 12501 | -0.295 | -0.1894 | Yes |
| 77 | RAB2A | "RAB2A, member RAS oncogene family [Source:HGNC Symbol;Acc:HGNC:9763]" | 12539 | -0.300 | -0.1789 | Yes |
| 78 | NAPA | NSF attachment protein alpha [Source:HGNC Symbol;Acc:HGNC:7641] | 12979 | -0.336 | -0.1926 | Yes |
| 79 | SEC24D | "SEC24 homolog D, COPII coat complex component [Source:HGNC Symbol;Acc:HGNC:10706]" | 13032 | -0.341 | -0.1812 | Yes |
| 80 | SEC31A | "SEC31 homolog A, COPII coat complex component [Source:HGNC Symbol;Acc:HGNC:17052]" | 13053 | -0.343 | -0.1677 | Yes |
| 81 | OCRL | OCRL inositol polyphosphate-5-phosphatase [Source:HGNC Symbol;Acc:HGNC:8108] | 13214 | -0.357 | -0.1625 | Yes |
| 82 | USO1 | USO1 vesicle transport factor [Source:HGNC Symbol;Acc:HGNC:30904] | 13216 | -0.358 | -0.1471 | Yes |
| 83 | VPS45 | vacuolar protein sorting 45 homolog [Source:HGNC Symbol;Acc:HGNC:14579] | 13319 | -0.369 | -0.1378 | Yes |
| 84 | SH3GL2 | "SH3 domain containing GRB2 like 2, endophilin A1 [Source:HGNC Symbol;Acc:HGNC:10831]" | 13388 | -0.378 | -0.1258 | Yes |
| 85 | SCAMP3 | secretory carrier membrane protein 3 [Source:HGNC Symbol;Acc:HGNC:10565] | 13522 | -0.394 | -0.1173 | Yes |
| 86 | VAMP7 | vesicle associated membrane protein 7 [Source:HGNC Symbol;Acc:HGNC:11486] | 13602 | -0.405 | -0.1049 | Yes |
| 87 | ERGIC3 | ERGIC and golgi 3 [Source:HGNC Symbol;Acc:HGNC:15927] | 13863 | -0.425 | -0.1032 | Yes |
| 88 | PAM | peptidylglycine alpha-amidating monooxygenase [Source:HGNC Symbol;Acc:HGNC:8596] | 13872 | -0.427 | -0.0853 | Yes |
| 89 | ARFGAP3 | ADP ribosylation factor GTPase activating protein 3 [Source:HGNC Symbol;Acc:HGNC:661] | 14098 | -0.449 | -0.0804 | Yes |
| 90 | GBF1 | golgi brefeldin A resistant guanine nucleotide exchange factor 1 [Source:HGNC Symbol;Acc:HGNC:4181] | 14102 | -0.450 | -0.0612 | Yes |
| 91 | COPE | COPI coat complex subunit epsilon [Source:HGNC Symbol;Acc:HGNC:2234] | 14422 | -0.496 | -0.0602 | Yes |
| 92 | SGMS1 | sphingomyelin synthase 1 [Source:HGNC Symbol;Acc:HGNC:29799] | 14429 | -0.498 | -0.0391 | Yes |
| 93 | BNIP3 | BCL2 interacting protein 3 [Source:HGNC Symbol;Acc:HGNC:1084] | 14859 | -0.602 | -0.0407 | Yes |
| 94 | ICA1 | islet cell autoantigen 1 [Source:HGNC Symbol;Acc:HGNC:5343] | 15045 | -0.684 | -0.0230 | Yes |
| 95 | SCAMP1 | secretory carrier membrane protein 1 [Source:HGNC Symbol;Acc:HGNC:10563] | 15081 | -0.706 | 0.0052 | Yes |
| 96 | SCRN1 | secernin 1 [Source:HGNC Symbol;Acc:HGNC:22192] | 15145 | -0.750 | 0.0336 | Yes |
Table: GSEA details [plain text format]

  

Fig 2: HALLMARK\_PROTEIN\_SECRETION      
 Blue-Pink O' Gram in the Space of the Analyzed GeneSet

  

Fig 3: HALLMARK\_PROTEIN\_SECRETION: Random ES distribution      
 Gene set null distribution of ES for **HALLMARK\_PROTEIN\_SECRETION**
