# Supplementary material for: In vivo CRISPR screens reveal Serpinb9 and Adam2 as regulators of immune therapy response in lung cancer
Source: Nat Commun. 2023 May 31;14:3150. doi: 10.1038/s41467-023-38841-7 (PMC10232477; doi:10.1038/s41467-023-38841-7)
Supplement: Supplementary file 13 — Source Data [file 41467_2023_38841_MOESM13_ESM.zip › Figure 4E.zip!/my_analysis.Gsea.1640146340255_Hallmark/HALLMARK_APICAL_SURFACE.html]

Details for gene set HALLMARK\_APICAL\_SURFACE[GSEA]

|  || Dataset | December 21, 2021\_for GSEA run\_collapsed\_to\_symbols.GSEA\_B6\_NI.cls #CTLR\_NI\_versus\_ADAM2\_NI |
| Phenotype | GSEA\_B6\_NI.cls#CTLR\_NI\_versus\_ADAM2\_NI |
| Upregulated in class | ADAM2\_NI |
| GeneSet | HALLMARK\_APICAL\_SURFACE |
| Enrichment Score (ES) | -0.4263686 |
| Normalized Enrichment Score (NES) | -1.3288654 |
| Nominal p-value | 0.0 |
| FDR q-value | 0.99676645 |
| FWER p-Value | 0.837 |
Table: GSEA Results Summary

  

Fig 1: Enrichment plot: HALLMARK\_APICAL\_SURFACE      
 Profile of the Running ES Score & Positions of GeneSet Members on the Rank Ordered List

  

| SYMBOL | TITLE | RANK IN GENE LIST | RANK METRIC SCORE | RUNNING ES | CORE ENRICHMENT || 1 | IL2RB | interleukin 2 receptor subunit beta [Source:HGNC Symbol;Acc:HGNC:6009] | 138 | 1.679 | 0.0751 | No |
| 2 | IL2RG | interleukin 2 receptor subunit gamma [Source:HGNC Symbol;Acc:HGNC:6010] | 432 | 1.141 | 0.1134 | No |
| 3 | CRYBG1 | crystallin beta-gamma domain containing 1 [Source:HGNC Symbol;Acc:HGNC:356] | 798 | 0.879 | 0.1340 | No |
| 4 | SLC2A4 | solute carrier family 2 member 4 [Source:HGNC Symbol;Acc:HGNC:11009] | 1684 | 0.572 | 0.1060 | No |
| 5 | LYN | "LYN proto-oncogene, Src family tyrosine kinase [Source:HGNC Symbol;Acc:HGNC:6735]" | 2037 | 0.501 | 0.1085 | No |
| 6 | RHCG | Rh family C glycoprotein [Source:HGNC Symbol;Acc:HGNC:18140] | 2139 | 0.490 | 0.1266 | No |
| 7 | PCSK9 | proprotein convertase subtilisin/kexin type 9 [Source:HGNC Symbol;Acc:HGNC:20001] | 2166 | 0.486 | 0.1492 | No |
| 8 | APP | amyloid beta precursor protein [Source:HGNC Symbol;Acc:HGNC:620] | 2788 | 0.418 | 0.1303 | No |
| 9 | ATP8B1 | ATPase phospholipid transporting 8B1 [Source:HGNC Symbol;Acc:HGNC:3706] | 3000 | 0.404 | 0.1370 | No |
| 10 | DCBLD2 | "discoidin, CUB and LCCL domain containing 2 [Source:HGNC Symbol;Acc:HGNC:24627]" | 3417 | 0.340 | 0.1274 | No |
| 11 | BRCA1 | BRCA1 DNA repair associated [Source:HGNC Symbol;Acc:HGNC:1100] | 4003 | 0.265 | 0.1032 | No |
| 12 | CD160 | CD160 molecule [Source:HGNC Symbol;Acc:HGNC:17013] | 4218 | 0.241 | 0.1016 | No |
| 13 | AKAP7 | A-kinase anchoring protein 7 [Source:HGNC Symbol;Acc:HGNC:377] | 5180 | 0.152 | 0.0477 | No |
| 14 | PLAUR | "plasminogen activator, urokinase receptor [Source:HGNC Symbol;Acc:HGNC:9053]" | 5260 | 0.146 | 0.0500 | No |
| 15 | SRPX | sushi repeat containing protein X-linked [Source:HGNC Symbol;Acc:HGNC:11309] | 6145 | 0.079 | -0.0026 | No |
| 16 | THY1 | Thy-1 cell surface antigen [Source:HGNC Symbol;Acc:HGNC:11801] | 6495 | 0.055 | -0.0222 | No |
| 17 | HSPB1 | heat shock protein family B (small) member 1 [Source:HGNC Symbol;Acc:HGNC:5246] | 6615 | 0.048 | -0.0274 | No |
| 18 | B4GALT1 | "beta-1,4-galactosyltransferase 1 [Source:HGNC Symbol;Acc:HGNC:924]" | 6824 | 0.035 | -0.0390 | No |
| 19 | ATP6V0A4 | ATPase H+ transporting V0 subunit a4 [Source:HGNC Symbol;Acc:HGNC:866] | 6989 | 0.025 | -0.0482 | No |
| 20 | ADAM10 | ADAM metallopeptidase domain 10 [Source:HGNC Symbol;Acc:HGNC:188] | 8009 | -0.029 | -0.1120 | No |
| 21 | FLOT2 | flotillin 2 [Source:HGNC Symbol;Acc:HGNC:3758] | 8992 | -0.082 | -0.1707 | No |
| 22 | NCOA6 | nuclear receptor coactivator 6 [Source:HGNC Symbol;Acc:HGNC:15936] | 9177 | -0.092 | -0.1779 | No |
| 23 | ADIPOR2 | adiponectin receptor 2 [Source:HGNC Symbol;Acc:HGNC:24041] | 9231 | -0.094 | -0.1765 | No |
| 24 | PKHD1 | PKHD1 ciliary IPT domain containing fibrocystin/polyductin [Source:HGNC Symbol;Acc:HGNC:9016] | 10155 | -0.151 | -0.2281 | No |
| 25 | CX3CL1 | C-X3-C motif chemokine ligand 1 [Source:HGNC Symbol;Acc:HGNC:10647] | 11232 | -0.213 | -0.2862 | No |
| 26 | CROCC | "ciliary rootlet coiled-coil, rootletin [Source:HGNC Symbol;Acc:HGNC:21299]" | 11604 | -0.235 | -0.2982 | No |
| 27 | EPHB4 | EPH receptor B4 [Source:HGNC Symbol;Acc:HGNC:3395] | 13608 | -0.406 | -0.4061 | Yes |
| 28 | NTNG1 | netrin G1 [Source:HGNC Symbol;Acc:HGNC:23319] | 13685 | -0.415 | -0.3902 | Yes |
| 29 | RTN4RL1 | reticulon 4 receptor like 1 [Source:HGNC Symbol;Acc:HGNC:21329] | 13780 | -0.416 | -0.3754 | Yes |
| 30 | TMEM8B | transmembrane protein 8B [Source:HGNC Symbol;Acc:HGNC:21427] | 14491 | -0.512 | -0.3952 | Yes |
| 31 | GHRL | ghrelin and obestatin prepropeptide [Source:HGNC Symbol;Acc:HGNC:18129] | 14816 | -0.588 | -0.3866 | Yes |
| 32 | SHROOM2 | shroom family member 2 [Source:HGNC Symbol;Acc:HGNC:630] | 14850 | -0.599 | -0.3587 | Yes |
| 33 | GAS1 | growth arrest specific 1 [Source:HGNC Symbol;Acc:HGNC:4165] | 15129 | -0.734 | -0.3398 | Yes |
| 34 | MDGA1 | MAM domain containing glycosylphosphatidylinositol anchor 1 [Source:HGNC Symbol;Acc:HGNC:19267] | 15134 | -0.739 | -0.3031 | Yes |
| 35 | EFNA5 | ephrin A5 [Source:HGNC Symbol;Acc:HGNC:3225] | 15347 | -0.859 | -0.2738 | Yes |
| 36 | MAL | "mal, T cell differentiation protein [Source:HGNC Symbol;Acc:HGNC:6817]" | 15354 | -0.863 | -0.2310 | Yes |
| 37 | GSTM3 | glutathione S-transferase mu 3 [Source:HGNC Symbol;Acc:HGNC:4635] | 15393 | -0.884 | -0.1892 | Yes |
| 38 | AFAP1L2 | actin filament associated protein 1 like 2 [Source:HGNC Symbol;Acc:HGNC:25901] | 15448 | -0.953 | -0.1450 | Yes |
| 39 | SULF2 | sulfatase 2 [Source:HGNC Symbol;Acc:HGNC:20392] | 15491 | -1.028 | -0.0963 | Yes |
| 40 | GATA3 | GATA binding protein 3 [Source:HGNC Symbol;Acc:HGNC:4172] | 15661 | -2.152 | 0.0004 | Yes |
Table: GSEA details [plain text format]

  

Fig 2: HALLMARK\_APICAL\_SURFACE      
 Blue-Pink O' Gram in the Space of the Analyzed GeneSet

  

Fig 3: HALLMARK\_APICAL\_SURFACE: Random ES distribution      
 Gene set null distribution of ES for **HALLMARK\_APICAL\_SURFACE**
