# Supplementary material for: In vivo CRISPR screens reveal Serpinb9 and Adam2 as regulators of immune therapy response in lung cancer
Source: Nat Commun. 2023 May 31;14:3150. doi: 10.1038/s41467-023-38841-7 (PMC10232477; doi:10.1038/s41467-023-38841-7)
Supplement: Supplementary file 13 — Source Data [file 41467_2023_38841_MOESM13_ESM.zip › Figure 4E.zip!/my_analysis.Gsea.1640146340255_Hallmark/HALLMARK_EPITHELIAL_MESENCHYMAL_TRANSITION.html]

Details for gene set HALLMARK\_EPITHELIAL\_MESENCHYMAL\_TRANSITION[GSEA]

|  || Dataset | December 21, 2021\_for GSEA run\_collapsed\_to\_symbols.GSEA\_B6\_NI.cls #CTLR\_NI\_versus\_ADAM2\_NI |
| Phenotype | GSEA\_B6\_NI.cls#CTLR\_NI\_versus\_ADAM2\_NI |
| Upregulated in class | CTLR\_NI |
| GeneSet | HALLMARK\_EPITHELIAL\_MESENCHYMAL\_TRANSITION |
| Enrichment Score (ES) | 0.24809392 |
| Normalized Enrichment Score (NES) | 1.0407457 |
| Nominal p-value | 0.29310346 |
| FDR q-value | 0.70496655 |
| FWER p-Value | 1.0 |
Table: GSEA Results Summary

  

Fig 1: Enrichment plot: HALLMARK\_EPITHELIAL\_MESENCHYMAL\_TRANSITION      
 Profile of the Running ES Score & Positions of GeneSet Members on the Rank Ordered List

  

| SYMBOL | TITLE | RANK IN GENE LIST | RANK METRIC SCORE | RUNNING ES | CORE ENRICHMENT || 1 | FMOD | fibromodulin [Source:HGNC Symbol;Acc:HGNC:3774] | 10 | 2.618 | 0.0308 | Yes |
| 2 | MMP3 | matrix metallopeptidase 3 [Source:HGNC Symbol;Acc:HGNC:7173] | 223 | 1.434 | 0.0343 | Yes |
| 3 | PTX3 | pentraxin 3 [Source:HGNC Symbol;Acc:HGNC:9692] | 279 | 1.338 | 0.0468 | Yes |
| 4 | TGM2 | transglutaminase 2 [Source:HGNC Symbol;Acc:HGNC:11778] | 305 | 1.293 | 0.0606 | Yes |
| 5 | IL6 | interleukin 6 [Source:HGNC Symbol;Acc:HGNC:6018] | 378 | 1.200 | 0.0704 | Yes |
| 6 | ABI3BP | ABI family member 3 binding protein [Source:HGNC Symbol;Acc:HGNC:17265] | 395 | 1.176 | 0.0835 | Yes |
| 7 | FAS | Fas cell surface death receptor [Source:HGNC Symbol;Acc:HGNC:11920] | 418 | 1.154 | 0.0959 | Yes |
| 8 | FBLN2 | fibulin 2 [Source:HGNC Symbol;Acc:HGNC:3601] | 430 | 1.143 | 0.1089 | Yes |
| 9 | COL6A3 | collagen type VI alpha 3 chain [Source:HGNC Symbol;Acc:HGNC:2213] | 515 | 1.054 | 0.1161 | Yes |
| 10 | MMP1 | matrix metallopeptidase 1 [Source:HGNC Symbol;Acc:HGNC:7155] | 528 | 1.037 | 0.1278 | Yes |
| 11 | GADD45B | growth arrest and DNA damage inducible beta [Source:HGNC Symbol;Acc:HGNC:4096] | 667 | 0.954 | 0.1303 | Yes |
| 12 | TIMP3 | TIMP metallopeptidase inhibitor 3 [Source:HGNC Symbol;Acc:HGNC:11822] | 707 | 0.927 | 0.1389 | Yes |
| 13 | LOX | lysyl oxidase [Source:HGNC Symbol;Acc:HGNC:6664] | 731 | 0.914 | 0.1484 | Yes |
| 14 | FOXC2 | forkhead box C2 [Source:HGNC Symbol;Acc:HGNC:3801] | 777 | 0.889 | 0.1561 | Yes |
| 15 | COL8A2 | collagen type VIII alpha 2 chain [Source:HGNC Symbol;Acc:HGNC:2216] | 813 | 0.871 | 0.1643 | Yes |
| 16 | POSTN | periostin [Source:HGNC Symbol;Acc:HGNC:16953] | 814 | 0.871 | 0.1747 | Yes |
| 17 | CXCL12 | C-X-C motif chemokine ligand 12 [Source:HGNC Symbol;Acc:HGNC:10672] | 1092 | 0.774 | 0.1661 | Yes |
| 18 | TFPI2 | tissue factor pathway inhibitor 2 [Source:HGNC Symbol;Acc:HGNC:11761] | 1096 | 0.771 | 0.1752 | Yes |
| 19 | ITGA2 | integrin subunit alpha 2 [Source:HGNC Symbol;Acc:HGNC:6137] | 1109 | 0.769 | 0.1836 | Yes |
| 20 | FSTL1 | follistatin like 1 [Source:HGNC Symbol;Acc:HGNC:3972] | 1122 | 0.763 | 0.1920 | Yes |
| 21 | NNMT | nicotinamide N-methyltransferase [Source:HGNC Symbol;Acc:HGNC:7861] | 1154 | 0.747 | 0.1990 | Yes |
| 22 | SGCD | sarcoglycan delta [Source:HGNC Symbol;Acc:HGNC:10807] | 1229 | 0.722 | 0.2029 | Yes |
| 23 | SFRP1 | secreted frizzled related protein 1 [Source:HGNC Symbol;Acc:HGNC:10776] | 1232 | 0.721 | 0.2114 | Yes |
| 24 | LAMA1 | laminin subunit alpha 1 [Source:HGNC Symbol;Acc:HGNC:6481] | 1256 | 0.712 | 0.2184 | Yes |
| 25 | WNT5A | Wnt family member 5A [Source:HGNC Symbol;Acc:HGNC:12784] | 1464 | 0.638 | 0.2127 | Yes |
| 26 | COL12A1 | collagen type XII alpha 1 chain [Source:HGNC Symbol;Acc:HGNC:2188] | 1545 | 0.611 | 0.2149 | Yes |
| 27 | TGFBI | transforming growth factor beta induced [Source:HGNC Symbol;Acc:HGNC:11771] | 1655 | 0.577 | 0.2147 | Yes |
| 28 | ECM2 | extracellular matrix protein 2 [Source:HGNC Symbol;Acc:HGNC:3154] | 1670 | 0.575 | 0.2207 | Yes |
| 29 | COL1A2 | collagen type I alpha 2 chain [Source:HGNC Symbol;Acc:HGNC:2198] | 1673 | 0.574 | 0.2275 | Yes |
| 30 | TNFAIP3 | TNF alpha induced protein 3 [Source:HGNC Symbol;Acc:HGNC:11896] | 1713 | 0.564 | 0.2317 | Yes |
| 31 | MATN3 | matrilin 3 [Source:HGNC Symbol;Acc:HGNC:6909] | 1780 | 0.550 | 0.2341 | Yes |
| 32 | LUM | lumican [Source:HGNC Symbol;Acc:HGNC:6724] | 1837 | 0.536 | 0.2369 | Yes |
| 33 | COL11A1 | collagen type XI alpha 1 chain [Source:HGNC Symbol;Acc:HGNC:2186] | 1935 | 0.516 | 0.2368 | Yes |
| 34 | CDH11 | cadherin 11 [Source:HGNC Symbol;Acc:HGNC:1750] | 1959 | 0.511 | 0.2415 | Yes |
| 35 | COL1A1 | collagen type I alpha 1 chain [Source:HGNC Symbol;Acc:HGNC:2197] | 1960 | 0.511 | 0.2476 | Yes |
| 36 | IGFBP4 | insulin like growth factor binding protein 4 [Source:HGNC Symbol;Acc:HGNC:5473] | 2183 | 0.482 | 0.2390 | Yes |
| 37 | MMP2 | matrix metallopeptidase 2 [Source:HGNC Symbol;Acc:HGNC:7166] | 2247 | 0.477 | 0.2407 | Yes |
| 38 | COL5A2 | collagen type V alpha 2 chain [Source:HGNC Symbol;Acc:HGNC:2210] | 2294 | 0.468 | 0.2433 | Yes |
| 39 | BASP1 | brain abundant membrane attached signal protein 1 [Source:HGNC Symbol;Acc:HGNC:957] | 2366 | 0.457 | 0.2442 | Yes |
| 40 | ANPEP | "alanyl aminopeptidase, membrane [Source:HGNC Symbol;Acc:HGNC:500]" | 2391 | 0.452 | 0.2481 | Yes |
| 41 | CDH6 | cadherin 6 [Source:HGNC Symbol;Acc:HGNC:1765] | 2530 | 0.445 | 0.2445 | No |
| 42 | IGFBP2 | insulin like growth factor binding protein 2 [Source:HGNC Symbol;Acc:HGNC:5471] | 2671 | 0.426 | 0.2406 | No |
| 43 | IL15 | interleukin 15 [Source:HGNC Symbol;Acc:HGNC:5977] | 2791 | 0.418 | 0.2379 | No |
| 44 | NTM | neurotrimin [Source:HGNC Symbol;Acc:HGNC:17941] | 2871 | 0.411 | 0.2377 | No |
| 45 | MFAP5 | microfibril associated protein 5 [Source:HGNC Symbol;Acc:HGNC:29673] | 2973 | 0.409 | 0.2361 | No |
| 46 | DAB2 | DAB adaptor protein 2 [Source:HGNC Symbol;Acc:HGNC:2662] | 3069 | 0.393 | 0.2347 | No |
| 47 | BGN | biglycan [Source:HGNC Symbol;Acc:HGNC:1044] | 3138 | 0.382 | 0.2349 | No |
| 48 | GPC1 | glypican 1 [Source:HGNC Symbol;Acc:HGNC:4449] | 3143 | 0.382 | 0.2392 | No |
| 49 | THBS1 | thrombospondin 1 [Source:HGNC Symbol;Acc:HGNC:11785] | 3316 | 0.355 | 0.2323 | No |
| 50 | INHBA | inhibin subunit beta A [Source:HGNC Symbol;Acc:HGNC:6066] | 3323 | 0.354 | 0.2362 | No |
| 51 | CXCL1 | C-X-C motif chemokine ligand 1 [Source:HGNC Symbol;Acc:HGNC:4602] | 3324 | 0.354 | 0.2404 | No |
| 52 | LAMA3 | laminin subunit alpha 3 [Source:HGNC Symbol;Acc:HGNC:6483] | 3398 | 0.341 | 0.2398 | No |
| 53 | LAMA2 | laminin subunit alpha 2 [Source:HGNC Symbol;Acc:HGNC:6482] | 3500 | 0.328 | 0.2372 | No |
| 54 | COL7A1 | collagen type VII alpha 1 chain [Source:HGNC Symbol;Acc:HGNC:2214] | 3606 | 0.314 | 0.2342 | No |
| 55 | TNC | tenascin C [Source:HGNC Symbol;Acc:HGNC:5318] | 3673 | 0.304 | 0.2336 | No |
| 56 | FBLN1 | fibulin 1 [Source:HGNC Symbol;Acc:HGNC:3600] | 3838 | 0.286 | 0.2264 | No |
| 57 | GPX7 | glutathione peroxidase 7 [Source:HGNC Symbol;Acc:HGNC:4559] | 4016 | 0.264 | 0.2181 | No |
| 58 | PRRX1 | paired related homeobox 1 [Source:HGNC Symbol;Acc:HGNC:9142] | 4018 | 0.264 | 0.2212 | No |
| 59 | SGCG | sarcoglycan gamma [Source:HGNC Symbol;Acc:HGNC:10809] | 4417 | 0.221 | 0.1982 | No |
| 60 | FBN1 | fibrillin 1 [Source:HGNC Symbol;Acc:HGNC:3603] | 4479 | 0.215 | 0.1968 | No |
| 61 | CXCL6 | C-X-C motif chemokine ligand 6 [Source:HGNC Symbol;Acc:HGNC:10643] | 4655 | 0.200 | 0.1879 | No |
| 62 | VCAN | versican [Source:HGNC Symbol;Acc:HGNC:2464] | 4697 | 0.197 | 0.1876 | No |
| 63 | FERMT2 | fermitin family member 2 [Source:HGNC Symbol;Acc:HGNC:15767] | 4888 | 0.179 | 0.1775 | No |
| 64 | FAP | fibroblast activation protein alpha [Source:HGNC Symbol;Acc:HGNC:3590] | 4895 | 0.178 | 0.1792 | No |
| 65 | SDC1 | syndecan 1 [Source:HGNC Symbol;Acc:HGNC:10658] | 4954 | 0.172 | 0.1776 | No |
| 66 | TIMP1 | TIMP metallopeptidase inhibitor 1 [Source:HGNC Symbol;Acc:HGNC:11820] | 4971 | 0.171 | 0.1786 | No |
| 67 | PLAUR | "plasminogen activator, urokinase receptor [Source:HGNC Symbol;Acc:HGNC:9053]" | 5260 | 0.146 | 0.1617 | No |
| 68 | GEM | GTP binding protein overexpressed in skeletal muscle [Source:HGNC Symbol;Acc:HGNC:4234] | 5511 | 0.127 | 0.1471 | No |
| 69 | PMP22 | peripheral myelin protein 22 [Source:HGNC Symbol;Acc:HGNC:9118] | 5531 | 0.125 | 0.1474 | No |
| 70 | ACTA2 | "actin alpha 2, smooth muscle [Source:HGNC Symbol;Acc:HGNC:130]" | 5628 | 0.117 | 0.1426 | No |
| 71 | ITGB3 | integrin subunit beta 3 [Source:HGNC Symbol;Acc:HGNC:6156] | 5695 | 0.113 | 0.1396 | No |
| 72 | ENO2 | enolase 2 [Source:HGNC Symbol;Acc:HGNC:3353] | 5991 | 0.092 | 0.1217 | No |
| 73 | TGFB1 | transforming growth factor beta 1 [Source:HGNC Symbol;Acc:HGNC:11766] | 6038 | 0.088 | 0.1198 | No |
| 74 | SNAI2 | snail family transcriptional repressor 2 [Source:HGNC Symbol;Acc:HGNC:11094] | 6041 | 0.088 | 0.1207 | No |
| 75 | TNFRSF11B | TNF receptor superfamily member 11b [Source:HGNC Symbol;Acc:HGNC:11909] | 6124 | 0.081 | 0.1164 | No |
| 76 | DST | dystonin [Source:HGNC Symbol;Acc:HGNC:1090] | 6192 | 0.077 | 0.1130 | No |
| 77 | SAT1 | spermidine/spermine N1-acetyltransferase 1 [Source:HGNC Symbol;Acc:HGNC:10540] | 6202 | 0.076 | 0.1133 | No |
| 78 | VCAM1 | vascular cell adhesion molecule 1 [Source:HGNC Symbol;Acc:HGNC:12663] | 6218 | 0.075 | 0.1132 | No |
| 79 | ITGA5 | integrin subunit alpha 5 [Source:HGNC Symbol;Acc:HGNC:6141] | 6225 | 0.075 | 0.1137 | No |
| 80 | DCN | decorin [Source:HGNC Symbol;Acc:HGNC:2705] | 6238 | 0.074 | 0.1139 | No |
| 81 | THY1 | Thy-1 cell surface antigen [Source:HGNC Symbol;Acc:HGNC:11801] | 6495 | 0.055 | 0.0980 | No |
| 82 | LOXL1 | lysyl oxidase like 1 [Source:HGNC Symbol;Acc:HGNC:6665] | 6511 | 0.054 | 0.0977 | No |
| 83 | FSTL3 | follistatin like 3 [Source:HGNC Symbol;Acc:HGNC:3973] | 6619 | 0.047 | 0.0913 | No |
| 84 | CD59 | CD59 molecule (CD59 blood group) [Source:HGNC Symbol;Acc:HGNC:1689] | 6681 | 0.044 | 0.0879 | No |
| 85 | WIPF1 | WAS/WASL interacting protein family member 1 [Source:HGNC Symbol;Acc:HGNC:12736] | 6886 | 0.030 | 0.0751 | No |
| 86 | BMP1 | bone morphogenetic protein 1 [Source:HGNC Symbol;Acc:HGNC:1067] | 6929 | 0.027 | 0.0727 | No |
| 87 | TPM1 | tropomyosin 1 [Source:HGNC Symbol;Acc:HGNC:12010] | 7124 | 0.017 | 0.0604 | No |
| 88 | FLNA | filamin A [Source:HGNC Symbol;Acc:HGNC:3754] | 7185 | 0.013 | 0.0566 | No |
| 89 | PLOD2 | "procollagen-lysine,2-oxoglutarate 5-dioxygenase 2 [Source:HGNC Symbol;Acc:HGNC:9082]" | 7291 | 0.007 | 0.0500 | No |
| 90 | LRRC15 | leucine rich repeat containing 15 [Source:HGNC Symbol;Acc:HGNC:20818] | 7425 | -0.001 | 0.0414 | No |
| 91 | FGF2 | fibroblast growth factor 2 [Source:HGNC Symbol;Acc:HGNC:3676] | 7478 | -0.003 | 0.0380 | No |
| 92 | CAP2 | cyclase associated actin cytoskeleton regulatory protein 2 [Source:HGNC Symbol;Acc:HGNC:20039] | 7640 | -0.010 | 0.0278 | No |
| 93 | VEGFA | vascular endothelial growth factor A [Source:HGNC Symbol;Acc:HGNC:12680] | 7671 | -0.012 | 0.0260 | No |
| 94 | CALD1 | caldesmon 1 [Source:HGNC Symbol;Acc:HGNC:1441] | 7872 | -0.022 | 0.0133 | No |
| 95 | LRP1 | LDL receptor related protein 1 [Source:HGNC Symbol;Acc:HGNC:6692] | 7894 | -0.024 | 0.0122 | No |
| 96 | LAMC1 | laminin subunit gamma 1 [Source:HGNC Symbol;Acc:HGNC:6492] | 8012 | -0.029 | 0.0050 | No |
| 97 | CAPG | "capping actin protein, gelsolin like [Source:HGNC Symbol;Acc:HGNC:1474]" | 8049 | -0.031 | 0.0031 | No |
| 98 | DPYSL3 | dihydropyrimidinase like 3 [Source:HGNC Symbol;Acc:HGNC:3015] | 8161 | -0.038 | -0.0036 | No |
| 99 | SLIT3 | slit guidance ligand 3 [Source:HGNC Symbol;Acc:HGNC:11087] | 8199 | -0.040 | -0.0055 | No |
| 100 | EMP3 | epithelial membrane protein 3 [Source:HGNC Symbol;Acc:HGNC:3335] | 8226 | -0.041 | -0.0067 | No |
| 101 | SDC4 | syndecan 4 [Source:HGNC Symbol;Acc:HGNC:10661] | 8318 | -0.044 | -0.0121 | No |
| 102 | PDLIM4 | PDZ and LIM domain 4 [Source:HGNC Symbol;Acc:HGNC:16501] | 8371 | -0.047 | -0.0149 | No |
| 103 | ID2 | inhibitor of DNA binding 2 [Source:HGNC Symbol;Acc:HGNC:5361] | 8387 | -0.048 | -0.0153 | No |
| 104 | COPA | COPI coat complex subunit alpha [Source:HGNC Symbol;Acc:HGNC:2230] | 8420 | -0.050 | -0.0167 | No |
| 105 | SNTB1 | syntrophin beta 1 [Source:HGNC Symbol;Acc:HGNC:11168] | 8572 | -0.059 | -0.0258 | No |
| 106 | PVR | PVR cell adhesion molecule [Source:HGNC Symbol;Acc:HGNC:9705] | 8624 | -0.062 | -0.0283 | No |
| 107 | CDH2 | cadherin 2 [Source:HGNC Symbol;Acc:HGNC:1759] | 9057 | -0.085 | -0.0552 | No |
| 108 | CADM1 | cell adhesion molecule 1 [Source:HGNC Symbol;Acc:HGNC:5951] | 9550 | -0.114 | -0.0856 | No |
| 109 | TPM4 | tropomyosin 4 [Source:HGNC Symbol;Acc:HGNC:12013] | 9583 | -0.116 | -0.0863 | No |
| 110 | MMP14 | matrix metallopeptidase 14 [Source:HGNC Symbol;Acc:HGNC:7160] | 9684 | -0.124 | -0.0913 | No |
| 111 | COMP | cartilage oligomeric matrix protein [Source:HGNC Symbol;Acc:HGNC:2227] | 9749 | -0.128 | -0.0939 | No |
| 112 | NOTCH2 | notch receptor 2 [Source:HGNC Symbol;Acc:HGNC:7882] | 9770 | -0.129 | -0.0936 | No |
| 113 | ITGAV | integrin subunit alpha V [Source:HGNC Symbol;Acc:HGNC:6150] | 9955 | -0.139 | -0.1039 | No |
| 114 | TAGLN | transgelin [Source:HGNC Symbol;Acc:HGNC:11553] | 10037 | -0.144 | -0.1074 | No |
| 115 | SERPINE1 | serpin family E member 1 [Source:HGNC Symbol;Acc:HGNC:8583] | 10052 | -0.145 | -0.1065 | No |
| 116 | COL3A1 | collagen type III alpha 1 chain [Source:HGNC Symbol;Acc:HGNC:2201] | 10161 | -0.151 | -0.1117 | No |
| 117 | SGCB | sarcoglycan beta [Source:HGNC Symbol;Acc:HGNC:10806] | 10189 | -0.153 | -0.1116 | No |
| 118 | OXTR | oxytocin receptor [Source:HGNC Symbol;Acc:HGNC:8529] | 10208 | -0.155 | -0.1109 | No |
| 119 | SERPINE2 | serpin family E member 2 [Source:HGNC Symbol;Acc:HGNC:8951] | 10253 | -0.157 | -0.1119 | No |
| 120 | JUN | "Jun proto-oncogene, AP-1 transcription factor subunit [Source:HGNC Symbol;Acc:HGNC:6204]" | 10257 | -0.157 | -0.1102 | No |
| 121 | MCM7 | minichromosome maintenance complex component 7 [Source:HGNC Symbol;Acc:HGNC:6950] | 10302 | -0.160 | -0.1111 | No |
| 122 | PTHLH | parathyroid hormone like hormone [Source:HGNC Symbol;Acc:HGNC:9607] | 10611 | -0.178 | -0.1289 | No |
| 123 | CD44 | CD44 molecule (Indian blood group) [Source:HGNC Symbol;Acc:HGNC:1681] | 10617 | -0.178 | -0.1271 | No |
| 124 | ITGB1 | integrin subunit beta 1 [Source:HGNC Symbol;Acc:HGNC:6153] | 10916 | -0.196 | -0.1440 | No |
| 125 | RHOB | ras homolog family member B [Source:HGNC Symbol;Acc:HGNC:668] | 11216 | -0.212 | -0.1608 | No |
| 126 | ADAM12 | ADAM metallopeptidase domain 12 [Source:HGNC Symbol;Acc:HGNC:190] | 11245 | -0.214 | -0.1600 | No |
| 127 | FUCA1 | alpha-L-fucosidase 1 [Source:HGNC Symbol;Acc:HGNC:4006] | 11365 | -0.221 | -0.1650 | No |
| 128 | VIM | vimentin [Source:HGNC Symbol;Acc:HGNC:12692] | 11393 | -0.223 | -0.1641 | No |
| 129 | QSOX1 | quiescin sulfhydryl oxidase 1 [Source:HGNC Symbol;Acc:HGNC:9756] | 11515 | -0.230 | -0.1692 | No |
| 130 | COL5A3 | collagen type V alpha 3 chain [Source:HGNC Symbol;Acc:HGNC:14864] | 11711 | -0.241 | -0.1789 | No |
| 131 | TPM2 | tropomyosin 2 [Source:HGNC Symbol;Acc:HGNC:12011] | 11761 | -0.245 | -0.1791 | No |
| 132 | PPIB | peptidylprolyl isomerase B [Source:HGNC Symbol;Acc:HGNC:9255] | 11857 | -0.251 | -0.1822 | No |
| 133 | GREM1 | "gremlin 1, DAN family BMP antagonist [Source:HGNC Symbol;Acc:HGNC:2001]" | 11883 | -0.253 | -0.1808 | No |
| 134 | GADD45A | growth arrest and DNA damage inducible alpha [Source:HGNC Symbol;Acc:HGNC:4095] | 12093 | -0.267 | -0.1911 | No |
| 135 | CALU | calumenin [Source:HGNC Symbol;Acc:HGNC:1458] | 12125 | -0.269 | -0.1899 | No |
| 136 | PLOD3 | "procollagen-lysine,2-oxoglutarate 5-dioxygenase 3 [Source:HGNC Symbol;Acc:HGNC:9083]" | 12165 | -0.271 | -0.1892 | No |
| 137 | LAMC2 | laminin subunit gamma 2 [Source:HGNC Symbol;Acc:HGNC:6493] | 12173 | -0.272 | -0.1863 | No |
| 138 | ECM1 | extracellular matrix protein 1 [Source:HGNC Symbol;Acc:HGNC:3153] | 12213 | -0.274 | -0.1856 | No |
| 139 | GJA1 | gap junction protein alpha 1 [Source:HGNC Symbol;Acc:HGNC:4274] | 12277 | -0.279 | -0.1863 | No |
| 140 | TGFBR3 | transforming growth factor beta receptor 3 [Source:HGNC Symbol;Acc:HGNC:11774] | 12373 | -0.286 | -0.1890 | No |
| 141 | LGALS1 | galectin 1 [Source:HGNC Symbol;Acc:HGNC:6561] | 12490 | -0.295 | -0.1930 | No |
| 142 | COL16A1 | collagen type XVI alpha 1 chain [Source:HGNC Symbol;Acc:HGNC:2193] | 12641 | -0.307 | -0.1990 | No |
| 143 | SLC6A8 | solute carrier family 6 member 8 [Source:HGNC Symbol;Acc:HGNC:11055] | 12660 | -0.308 | -0.1964 | No |
| 144 | PLOD1 | "procollagen-lysine,2-oxoglutarate 5-dioxygenase 1 [Source:HGNC Symbol;Acc:HGNC:9081]" | 12706 | -0.312 | -0.1956 | No |
| 145 | CTHRC1 | collagen triple helix repeat containing 1 [Source:HGNC Symbol;Acc:HGNC:18831] | 12715 | -0.313 | -0.1924 | No |
| 146 | FBN2 | fibrillin 2 [Source:HGNC Symbol;Acc:HGNC:3604] | 12814 | -0.320 | -0.1949 | No |
| 147 | MATN2 | matrilin 2 [Source:HGNC Symbol;Acc:HGNC:6908] | 12837 | -0.322 | -0.1924 | No |
| 148 | CRLF1 | cytokine receptor like factor 1 [Source:HGNC Symbol;Acc:HGNC:2364] | 13059 | -0.344 | -0.2026 | No |
| 149 | AREG | amphiregulin [Source:HGNC Symbol;Acc:HGNC:651] | 13075 | -0.345 | -0.1994 | No |
| 150 | SERPINH1 | serpin family H member 1 [Source:HGNC Symbol;Acc:HGNC:1546] | 13150 | -0.352 | -0.2000 | No |
| 151 | CCN1 | cellular communication network factor 1 [Source:HGNC Symbol;Acc:HGNC:2654] | 13488 | -0.391 | -0.2171 | No |
| 152 | HTRA1 | HtrA serine peptidase 1 [Source:HGNC Symbol;Acc:HGNC:9476] | 13525 | -0.394 | -0.2147 | No |
| 153 | MAGEE1 | MAGE family member E1 [Source:HGNC Symbol;Acc:HGNC:24934] | 13627 | -0.409 | -0.2163 | No |
| 154 | FN1 | fibronectin 1 [Source:HGNC Symbol;Acc:HGNC:3778] | 14037 | -0.437 | -0.2375 | No |
| 155 | NID2 | nidogen 2 [Source:HGNC Symbol;Acc:HGNC:13389] | 14161 | -0.459 | -0.2399 | No |
| 156 | EDIL3 | EGF like repeats and discoidin domains 3 [Source:HGNC Symbol;Acc:HGNC:3173] | 14173 | -0.461 | -0.2351 | No |
| 157 | PCOLCE2 | procollagen C-endopeptidase enhancer 2 [Source:HGNC Symbol;Acc:HGNC:8739] | 14187 | -0.464 | -0.2304 | No |
| 158 | MEST | mesoderm specific transcript [Source:HGNC Symbol;Acc:HGNC:7028] | 14218 | -0.467 | -0.2267 | No |
| 159 | THBS2 | thrombospondin 2 [Source:HGNC Symbol;Acc:HGNC:11786] | 14262 | -0.473 | -0.2238 | No |
| 160 | ITGB5 | integrin subunit beta 5 [Source:HGNC Symbol;Acc:HGNC:6160] | 14269 | -0.475 | -0.2185 | No |
| 161 | SPARC | secreted protein acidic and cysteine rich [Source:HGNC Symbol;Acc:HGNC:11219] | 14281 | -0.478 | -0.2135 | No |
| 162 | PMEPA1 | "prostate transmembrane protein, androgen induced 1 [Source:HGNC Symbol;Acc:HGNC:14107]" | 14329 | -0.484 | -0.2107 | No |
| 163 | COL5A1 | collagen type V alpha 1 chain [Source:HGNC Symbol;Acc:HGNC:2209] | 14349 | -0.486 | -0.2061 | No |
| 164 | SLIT2 | slit guidance ligand 2 [Source:HGNC Symbol;Acc:HGNC:11086] | 14416 | -0.494 | -0.2044 | No |
| 165 | COLGALT1 | collagen beta(1-O)galactosyltransferase 1 [Source:HGNC Symbol;Acc:HGNC:26182] | 14442 | -0.502 | -0.2000 | No |
| 166 | BDNF | brain derived neurotrophic factor [Source:HGNC Symbol;Acc:HGNC:1033] | 14450 | -0.504 | -0.1944 | No |
| 167 | EFEMP2 | EGF containing fibulin extracellular matrix protein 2 [Source:HGNC Symbol;Acc:HGNC:3219] | 14495 | -0.513 | -0.1911 | No |
| 168 | PDGFRB | platelet derived growth factor receptor beta [Source:HGNC Symbol;Acc:HGNC:8804] | 14530 | -0.521 | -0.1871 | No |
| 169 | LOXL2 | lysyl oxidase like 2 [Source:HGNC Symbol;Acc:HGNC:6666] | 14546 | -0.523 | -0.1818 | No |
| 170 | GLIPR1 | GLI pathogenesis related 1 [Source:HGNC Symbol;Acc:HGNC:17001] | 14560 | -0.526 | -0.1763 | No |
| 171 | SPP1 | secreted phosphoprotein 1 [Source:HGNC Symbol;Acc:HGNC:11255] | 14610 | -0.535 | -0.1730 | No |
| 172 | MYL9 | myosin light chain 9 [Source:HGNC Symbol;Acc:HGNC:15754] | 14619 | -0.537 | -0.1671 | No |
| 173 | APLP1 | amyloid beta precursor like protein 1 [Source:HGNC Symbol;Acc:HGNC:597] | 14707 | -0.556 | -0.1661 | No |
| 174 | TNFRSF12A | TNF receptor superfamily member 12A [Source:HGNC Symbol;Acc:HGNC:18152] | 14839 | -0.595 | -0.1674 | No |
| 175 | P3H1 | prolyl 3-hydroxylase 1 [Source:HGNC Symbol;Acc:HGNC:19316] | 14877 | -0.609 | -0.1625 | No |
| 176 | CCN2 | cellular communication network factor 2 [Source:HGNC Symbol;Acc:HGNC:2500] | 14883 | -0.612 | -0.1555 | No |
| 177 | COL4A2 | collagen type IV alpha 2 chain [Source:HGNC Symbol;Acc:HGNC:2203] | 14887 | -0.614 | -0.1483 | No |
| 178 | RGS4 | regulator of G protein signaling 4 [Source:HGNC Symbol;Acc:HGNC:10000] | 14914 | -0.625 | -0.1425 | No |
| 179 | COL4A1 | collagen type IV alpha 1 chain [Source:HGNC Symbol;Acc:HGNC:2202] | 14958 | -0.646 | -0.1375 | No |
| 180 | GAS1 | growth arrest specific 1 [Source:HGNC Symbol;Acc:HGNC:4165] | 15129 | -0.734 | -0.1397 | No |
| 181 | PCOLCE | procollagen C-endopeptidase enhancer [Source:HGNC Symbol;Acc:HGNC:8738] | 15192 | -0.774 | -0.1344 | No |
| 182 | MYLK | myosin light chain kinase [Source:HGNC Symbol;Acc:HGNC:7590] | 15292 | -0.837 | -0.1308 | No |
| 183 | MSX1 | msh homeobox 1 [Source:HGNC Symbol;Acc:HGNC:7391] | 15355 | -0.863 | -0.1244 | No |
| 184 | FZD8 | frizzled class receptor 8 [Source:HGNC Symbol;Acc:HGNC:4046] | 15435 | -0.934 | -0.1183 | No |
| 185 | FBLN5 | fibulin 5 [Source:HGNC Symbol;Acc:HGNC:3602] | 15478 | -1.001 | -0.1090 | No |
| 186 | SFRP4 | secreted frizzled related protein 4 [Source:HGNC Symbol;Acc:HGNC:10778] | 15483 | -1.009 | -0.0972 | No |
| 187 | NT5E | 5'-nucleotidase ecto [Source:HGNC Symbol;Acc:HGNC:8021] | 15487 | -1.022 | -0.0851 | No |
| 188 | IGFBP3 | insulin like growth factor binding protein 3 [Source:HGNC Symbol;Acc:HGNC:5472] | 15536 | -1.107 | -0.0749 | No |
| 189 | VEGFC | vascular endothelial growth factor C [Source:HGNC Symbol;Acc:HGNC:12682] | 15542 | -1.121 | -0.0618 | No |
| 190 | PFN2 | profilin 2 [Source:HGNC Symbol;Acc:HGNC:8882] | 15589 | -1.240 | -0.0499 | No |
| 191 | COL6A2 | collagen type VI alpha 2 chain [Source:HGNC Symbol;Acc:HGNC:2212] | 15592 | -1.260 | -0.0349 | No |
| 192 | MGP | matrix Gla protein [Source:HGNC Symbol;Acc:HGNC:7060] | 15641 | -1.606 | -0.0188 | No |
| 193 | ELN | elastin [Source:HGNC Symbol;Acc:HGNC:3327] | 15649 | -1.705 | 0.0012 | No |
Table: GSEA details [plain text format]

  

Fig 2: HALLMARK\_EPITHELIAL\_MESENCHYMAL\_TRANSITION      
 Blue-Pink O' Gram in the Space of the Analyzed GeneSet

  

Fig 3: HALLMARK\_EPITHELIAL\_MESENCHYMAL\_TRANSITION: Random ES distribution      
 Gene set null distribution of ES for **HALLMARK\_EPITHELIAL\_MESENCHYMAL\_TRANSITION**
