# Supplementary material for: In vivo CRISPR screens reveal Serpinb9 and Adam2 as regulators of immune therapy response in lung cancer
Source: Nat Commun. 2023 May 31;14:3150. doi: 10.1038/s41467-023-38841-7 (PMC10232477; doi:10.1038/s41467-023-38841-7)
Supplement: Supplementary file 13 — Source Data [file 41467_2023_38841_MOESM13_ESM.zip › Figure 4E.zip!/my_analysis.Gsea.1640146340255_Hallmark/HALLMARK_NOTCH_SIGNALING.html]

Details for gene set HALLMARK\_NOTCH\_SIGNALING[GSEA]

|  || Dataset | December 21, 2021\_for GSEA run\_collapsed\_to\_symbols.GSEA\_B6\_NI.cls #CTLR\_NI\_versus\_ADAM2\_NI |
| Phenotype | GSEA\_B6\_NI.cls#CTLR\_NI\_versus\_ADAM2\_NI |
| Upregulated in class | ADAM2\_NI |
| GeneSet | HALLMARK\_NOTCH\_SIGNALING |
| Enrichment Score (ES) | -0.24178724 |
| Normalized Enrichment Score (NES) | -0.8546474 |
| Nominal p-value | 0.785567 |
| FDR q-value | 0.95617926 |
| FWER p-Value | 1.0 |
Table: GSEA Results Summary

  

Fig 1: Enrichment plot: HALLMARK\_NOTCH\_SIGNALING      
 Profile of the Running ES Score & Positions of GeneSet Members on the Rank Ordered List

  

| SYMBOL | TITLE | RANK IN GENE LIST | RANK METRIC SCORE | RUNNING ES | CORE ENRICHMENT || 1 | PSEN2 | presenilin 2 [Source:HGNC Symbol;Acc:HGNC:9509] | 1280 | 0.703 | -0.0119 | Yes |
| 2 | LFNG | LFNG O-fucosylpeptide 3-beta-N-acetylglucosaminyltransferase [Source:HGNC Symbol;Acc:HGNC:6560] | 1295 | 0.697 | 0.0565 | Yes |
| 3 | WNT5A | Wnt family member 5A [Source:HGNC Symbol;Acc:HGNC:12784] | 1464 | 0.638 | 0.1092 | Yes |
| 4 | WNT2 | Wnt family member 2 [Source:HGNC Symbol;Acc:HGNC:12780] | 1714 | 0.564 | 0.1493 | Yes |
| 5 | JAG1 | jagged canonical Notch ligand 1 [Source:HGNC Symbol;Acc:HGNC:6188] | 1788 | 0.546 | 0.1990 | Yes |
| 6 | NOTCH1 | notch receptor 1 [Source:HGNC Symbol;Acc:HGNC:7881] | 2327 | 0.465 | 0.2108 | Yes |
| 7 | FZD1 | frizzled class receptor 1 [Source:HGNC Symbol;Acc:HGNC:4038] | 2620 | 0.430 | 0.2349 | Yes |
| 8 | DTX4 | deltex E3 ubiquitin ligase 4 [Source:HGNC Symbol;Acc:HGNC:29151] | 4540 | 0.210 | 0.1331 | No |
| 9 | HES1 | hes family bHLH transcription factor 1 [Source:HGNC Symbol;Acc:HGNC:5192] | 5322 | 0.141 | 0.0971 | No |
| 10 | FZD7 | frizzled class receptor 7 [Source:HGNC Symbol;Acc:HGNC:4045] | 6696 | 0.043 | 0.0136 | No |
| 11 | FBXW11 | F-box and WD repeat domain containing 11 [Source:HGNC Symbol;Acc:HGNC:13607] | 7106 | 0.018 | -0.0108 | No |
| 12 | ARRB1 | arrestin beta 1 [Source:HGNC Symbol;Acc:HGNC:711] | 7178 | 0.013 | -0.0140 | No |
| 13 | DTX1 | deltex E3 ubiquitin ligase 1 [Source:HGNC Symbol;Acc:HGNC:3060] | 7473 | -0.003 | -0.0325 | No |
| 14 | DTX2 | deltex E3 ubiquitin ligase 2 [Source:HGNC Symbol;Acc:HGNC:15973] | 7562 | -0.007 | -0.0374 | No |
| 15 | PPARD | peroxisome proliferator activated receptor delta [Source:HGNC Symbol;Acc:HGNC:9235] | 8546 | -0.058 | -0.0945 | No |
| 16 | NOTCH2 | notch receptor 2 [Source:HGNC Symbol;Acc:HGNC:7882] | 9770 | -0.129 | -0.1599 | No |
| 17 | RBX1 | ring-box 1 [Source:HGNC Symbol;Acc:HGNC:9928] | 9819 | -0.132 | -0.1498 | No |
| 18 | CCND1 | cyclin D1 [Source:HGNC Symbol;Acc:HGNC:1582] | 9851 | -0.134 | -0.1385 | No |
| 19 | TCF7L2 | transcription factor 7 like 2 [Source:HGNC Symbol;Acc:HGNC:11641] | 10124 | -0.149 | -0.1411 | No |
| 20 | APH1A | "aph-1 homolog A, gamma-secretase subunit [Source:HGNC Symbol;Acc:HGNC:29509]" | 10226 | -0.156 | -0.1321 | No |
| 21 | ST3GAL6 | "ST3 beta-galactoside alpha-2,3-sialyltransferase 6 [Source:HGNC Symbol;Acc:HGNC:18080]" | 10227 | -0.156 | -0.1166 | No |
| 22 | CUL1 | cullin 1 [Source:HGNC Symbol;Acc:HGNC:2551] | 10236 | -0.156 | -0.1016 | No |
| 23 | FZD5 | frizzled class receptor 5 [Source:HGNC Symbol;Acc:HGNC:4043] | 10504 | -0.172 | -0.1016 | No |
| 24 | SKP1 | S-phase kinase associated protein 1 [Source:HGNC Symbol;Acc:HGNC:10899] | 10544 | -0.174 | -0.0867 | No |
| 25 | PSENEN | "presenilin enhancer, gamma-secretase subunit [Source:HGNC Symbol;Acc:HGNC:30100]" | 11065 | -0.204 | -0.0998 | No |
| 26 | SAP30 | Sin3A associated protein 30 [Source:HGNC Symbol;Acc:HGNC:10532] | 12567 | -0.302 | -0.1657 | No |
| 27 | KAT2A | lysine acetyltransferase 2A [Source:HGNC Symbol;Acc:HGNC:4201] | 12723 | -0.313 | -0.1445 | No |
| 28 | DLL1 | delta like canonical Notch ligand 1 [Source:HGNC Symbol;Acc:HGNC:2908] | 14245 | -0.471 | -0.1949 | No |
| 29 | MAML2 | mastermind like transcriptional coactivator 2 [Source:HGNC Symbol;Acc:HGNC:16259] | 14494 | -0.512 | -0.1598 | No |
| 30 | PRKCA | protein kinase C alpha [Source:HGNC Symbol;Acc:HGNC:9393] | 14498 | -0.513 | -0.1090 | No |
| 31 | HEYL | hes related family bHLH transcription factor with YRPW motif like [Source:HGNC Symbol;Acc:HGNC:4882] | 14804 | -0.585 | -0.0704 | No |
| 32 | NOTCH3 | notch receptor 3 [Source:HGNC Symbol;Acc:HGNC:7883] | 15593 | -1.262 | 0.0048 | No |
Table: GSEA details [plain text format]

  

Fig 2: HALLMARK\_NOTCH\_SIGNALING      
 Blue-Pink O' Gram in the Space of the Analyzed GeneSet

  

Fig 3: HALLMARK\_NOTCH\_SIGNALING: Random ES distribution      
 Gene set null distribution of ES for **HALLMARK\_NOTCH\_SIGNALING**
