# Supplementary material for: In vivo CRISPR screens reveal Serpinb9 and Adam2 as regulators of immune therapy response in lung cancer
Source: Nat Commun. 2023 May 31;14:3150. doi: 10.1038/s41467-023-38841-7 (PMC10232477; doi:10.1038/s41467-023-38841-7)
Supplement: Supplementary file 13 — Source Data [file 41467_2023_38841_MOESM13_ESM.zip › Figure 4E.zip!/my_analysis.Gsea.1640146340255_Hallmark/HALLMARK_WNT_BETA_CATENIN_SIGNALING.html]

Details for gene set HALLMARK\_WNT\_BETA\_CATENIN\_SIGNALING[GSEA]

|  || Dataset | December 21, 2021\_for GSEA run\_collapsed\_to\_symbols.GSEA\_B6\_NI.cls #CTLR\_NI\_versus\_ADAM2\_NI |
| Phenotype | GSEA\_B6\_NI.cls#CTLR\_NI\_versus\_ADAM2\_NI |
| Upregulated in class | ADAM2\_NI |
| GeneSet | HALLMARK\_WNT\_BETA\_CATENIN\_SIGNALING |
| Enrichment Score (ES) | -0.23172852 |
| Normalized Enrichment Score (NES) | -0.72259575 |
| Nominal p-value | 0.89106756 |
| FDR q-value | 0.8812524 |
| FWER p-Value | 1.0 |
Table: GSEA Results Summary

  

Fig 1: Enrichment plot: HALLMARK\_WNT\_BETA\_CATENIN\_SIGNALING      
 Profile of the Running ES Score & Positions of GeneSet Members on the Rank Ordered List

  

| SYMBOL | TITLE | RANK IN GENE LIST | RANK METRIC SCORE | RUNNING ES | CORE ENRICHMENT || 1 | LEF1 | lymphoid enhancer binding factor 1 [Source:HGNC Symbol;Acc:HGNC:6551] | 1114 | 0.765 | -0.0100 | No |
| 2 | HEY2 | hes related family bHLH transcription factor with YRPW motif 2 [Source:HGNC Symbol;Acc:HGNC:4881] | 1121 | 0.763 | 0.0507 | No |
| 3 | PSEN2 | presenilin 2 [Source:HGNC Symbol;Acc:HGNC:9509] | 1280 | 0.703 | 0.0969 | No |
| 4 | JAG1 | jagged canonical Notch ligand 1 [Source:HGNC Symbol;Acc:HGNC:6188] | 1788 | 0.546 | 0.1082 | No |
| 5 | NOTCH1 | notch receptor 1 [Source:HGNC Symbol;Acc:HGNC:7881] | 2327 | 0.465 | 0.1110 | No |
| 6 | FZD1 | frizzled class receptor 1 [Source:HGNC Symbol;Acc:HGNC:4038] | 2620 | 0.430 | 0.1267 | No |
| 7 | WNT1 | Wnt family member 1 [Source:HGNC Symbol;Acc:HGNC:12774] | 3090 | 0.390 | 0.1279 | No |
| 8 | PTCH1 | patched 1 [Source:HGNC Symbol;Acc:HGNC:9585] | 3244 | 0.367 | 0.1475 | No |
| 9 | TCF7 | transcription factor 7 [Source:HGNC Symbol;Acc:HGNC:11639] | 4430 | 0.219 | 0.0892 | No |
| 10 | ADAM17 | ADAM metallopeptidase domain 17 [Source:HGNC Symbol;Acc:HGNC:195] | 5273 | 0.145 | 0.0469 | No |
| 11 | WNT5B | Wnt family member 5B [Source:HGNC Symbol;Acc:HGNC:16265] | 5746 | 0.109 | 0.0255 | No |
| 12 | HDAC11 | histone deacetylase 11 [Source:HGNC Symbol;Acc:HGNC:19086] | 6039 | 0.088 | 0.0138 | No |
| 13 | AXIN2 | axin 2 [Source:HGNC Symbol;Acc:HGNC:904] | 6264 | 0.073 | 0.0053 | No |
| 14 | NUMB | NUMB endocytic adaptor protein [Source:HGNC Symbol;Acc:HGNC:8060] | 6278 | 0.071 | 0.0102 | No |
| 15 | HEY1 | hes related family bHLH transcription factor with YRPW motif 1 [Source:HGNC Symbol;Acc:HGNC:4880] | 6301 | 0.070 | 0.0144 | No |
| 16 | SKP2 | S-phase kinase associated protein 2 [Source:HGNC Symbol;Acc:HGNC:10901] | 6330 | 0.068 | 0.0180 | No |
| 17 | MYC | "MYC proto-oncogene, bHLH transcription factor [Source:HGNC Symbol;Acc:HGNC:7553]" | 6504 | 0.055 | 0.0113 | No |
| 18 | CCND2 | cyclin D2 [Source:HGNC Symbol;Acc:HGNC:1583] | 8119 | -0.036 | -0.0890 | No |
| 19 | HDAC2 | histone deacetylase 2 [Source:HGNC Symbol;Acc:HGNC:4853] | 8194 | -0.040 | -0.0906 | No |
| 20 | PPARD | peroxisome proliferator activated receptor delta [Source:HGNC Symbol;Acc:HGNC:9235] | 8546 | -0.058 | -0.1084 | No |
| 21 | CTNNB1 | catenin beta 1 [Source:HGNC Symbol;Acc:HGNC:2514] | 9410 | -0.105 | -0.1552 | No |
| 22 | GNAI1 | G protein subunit alpha i1 [Source:HGNC Symbol;Acc:HGNC:4384] | 9635 | -0.121 | -0.1599 | No |
| 23 | NCSTN | nicastrin [Source:HGNC Symbol;Acc:HGNC:17091] | 9864 | -0.134 | -0.1638 | No |
| 24 | CUL1 | cullin 1 [Source:HGNC Symbol;Acc:HGNC:2551] | 10236 | -0.156 | -0.1750 | No |
| 25 | RBPJ | recombination signal binding protein for immunoglobulin kappa J region [Source:HGNC Symbol;Acc:HGNC:5724] | 10616 | -0.178 | -0.1850 | No |
| 26 | DVL2 | dishevelled segment polarity protein 2 [Source:HGNC Symbol;Acc:HGNC:3086] | 10800 | -0.188 | -0.1816 | No |
| 27 | MAML1 | mastermind like transcriptional coactivator 1 [Source:HGNC Symbol;Acc:HGNC:13632] | 11085 | -0.205 | -0.1834 | No |
| 28 | TP53 | tumor protein p53 [Source:HGNC Symbol;Acc:HGNC:11998] | 11287 | -0.216 | -0.1790 | No |
| 29 | AXIN1 | axin 1 [Source:HGNC Symbol;Acc:HGNC:903] | 11998 | -0.261 | -0.2035 | No |
| 30 | JAG2 | jagged canonical Notch ligand 2 [Source:HGNC Symbol;Acc:HGNC:6189] | 12440 | -0.291 | -0.2084 | Yes |
| 31 | KAT2A | lysine acetyltransferase 2A [Source:HGNC Symbol;Acc:HGNC:4201] | 12723 | -0.313 | -0.2014 | Yes |
| 32 | HDAC5 | histone deacetylase 5 [Source:HGNC Symbol;Acc:HGNC:14068] | 13082 | -0.346 | -0.1966 | Yes |
| 33 | WNT6 | Wnt family member 6 [Source:HGNC Symbol;Acc:HGNC:12785] | 13198 | -0.356 | -0.1755 | Yes |
| 34 | NCOR2 | nuclear receptor corepressor 2 [Source:HGNC Symbol;Acc:HGNC:7673] | 13301 | -0.367 | -0.1526 | Yes |
| 35 | DLL1 | delta like canonical Notch ligand 1 [Source:HGNC Symbol;Acc:HGNC:2908] | 14245 | -0.471 | -0.1752 | Yes |
| 36 | NOTCH4 | notch receptor 4 [Source:HGNC Symbol;Acc:HGNC:7884] | 15032 | -0.678 | -0.1712 | Yes |
| 37 | FRAT1 | FRAT regulator of WNT signaling pathway 1 [Source:HGNC Symbol;Acc:HGNC:3944] | 15110 | -0.725 | -0.1180 | Yes |
| 38 | FZD8 | frizzled class receptor 8 [Source:HGNC Symbol;Acc:HGNC:4046] | 15435 | -0.934 | -0.0640 | Yes |
| 39 | NKD1 | NKD inhibitor of WNT signaling pathway 1 [Source:HGNC Symbol;Acc:HGNC:17045] | 15469 | -0.985 | 0.0127 | Yes |
Table: GSEA details [plain text format]

  

Fig 2: HALLMARK\_WNT\_BETA\_CATENIN\_SIGNALING      
 Blue-Pink O' Gram in the Space of the Analyzed GeneSet

  

Fig 3: HALLMARK\_WNT\_BETA\_CATENIN\_SIGNALING: Random ES distribution      
 Gene set null distribution of ES for **HALLMARK\_WNT\_BETA\_CATENIN\_SIGNALING**
