# Supplementary material for: In vivo CRISPR screens reveal Serpinb9 and Adam2 as regulators of immune therapy response in lung cancer
Source: Nat Commun. 2023 May 31;14:3150. doi: 10.1038/s41467-023-38841-7 (PMC10232477; doi:10.1038/s41467-023-38841-7)
Supplement: Supplementary file 13 — Source Data [file 41467_2023_38841_MOESM13_ESM.zip › Figure 4E.zip!/my_analysis.Gsea.1640146340255_Hallmark/HALLMARK_IL6_JAK_STAT3_SIGNALING.html]

Details for gene set HALLMARK\_IL6\_JAK\_STAT3\_SIGNALING[GSEA]

|  || Dataset | December 21, 2021\_for GSEA run\_collapsed\_to\_symbols.GSEA\_B6\_NI.cls #CTLR\_NI\_versus\_ADAM2\_NI |
| Phenotype | GSEA\_B6\_NI.cls#CTLR\_NI\_versus\_ADAM2\_NI |
| Upregulated in class | CTLR\_NI |
| GeneSet | HALLMARK\_IL6\_JAK\_STAT3\_SIGNALING |
| Enrichment Score (ES) | 0.64556885 |
| Normalized Enrichment Score (NES) | 1.5607724 |
| Nominal p-value | 0.0 |
| FDR q-value | 0.18051566 |
| FWER p-Value | 0.336 |
Table: GSEA Results Summary

  

Fig 1: Enrichment plot: HALLMARK\_IL6\_JAK\_STAT3\_SIGNALING      
 Profile of the Running ES Score & Positions of GeneSet Members on the Rank Ordered List

  

| SYMBOL | TITLE | RANK IN GENE LIST | RANK METRIC SCORE | RUNNING ES | CORE ENRICHMENT || 1 | CXCL11 | C-X-C motif chemokine ligand 11 [Source:HGNC Symbol;Acc:HGNC:10638] | 2 | 3.216 | 0.0601 | Yes |
| 2 | STAT1 | signal transducer and activator of transcription 1 [Source:HGNC Symbol;Acc:HGNC:11362] | 45 | 2.090 | 0.0966 | Yes |
| 3 | STAT2 | signal transducer and activator of transcription 2 [Source:HGNC Symbol;Acc:HGNC:11363] | 86 | 1.830 | 0.1283 | Yes |
| 4 | IRF9 | interferon regulatory factor 9 [Source:HGNC Symbol;Acc:HGNC:6131] | 128 | 1.701 | 0.1575 | Yes |
| 5 | CXCL10 | C-X-C motif chemokine ligand 10 [Source:HGNC Symbol;Acc:HGNC:10637] | 142 | 1.666 | 0.1879 | Yes |
| 6 | IL18R1 | interleukin 18 receptor 1 [Source:HGNC Symbol;Acc:HGNC:5988] | 179 | 1.535 | 0.2143 | Yes |
| 7 | MAP3K8 | mitogen-activated protein kinase kinase kinase 8 [Source:HGNC Symbol;Acc:HGNC:6860] | 202 | 1.482 | 0.2406 | Yes |
| 8 | IL2RA | interleukin 2 receptor subunit alpha [Source:HGNC Symbol;Acc:HGNC:6008] | 205 | 1.469 | 0.2680 | Yes |
| 9 | IRF1 | interferon regulatory factor 1 [Source:HGNC Symbol;Acc:HGNC:6116] | 295 | 1.310 | 0.2868 | Yes |
| 10 | IL1B | interleukin 1 beta [Source:HGNC Symbol;Acc:HGNC:5992] | 303 | 1.298 | 0.3107 | Yes |
| 11 | IL1R2 | interleukin 1 receptor type 2 [Source:HGNC Symbol;Acc:HGNC:5994] | 318 | 1.270 | 0.3336 | Yes |
| 12 | CSF3R | colony stimulating factor 3 receptor [Source:HGNC Symbol;Acc:HGNC:2439] | 345 | 1.241 | 0.3552 | Yes |
| 13 | IL9R | interleukin 9 receptor [Source:HGNC Symbol;Acc:HGNC:6030] | 367 | 1.209 | 0.3765 | Yes |
| 14 | IL6 | interleukin 6 [Source:HGNC Symbol;Acc:HGNC:6018] | 378 | 1.200 | 0.3983 | Yes |
| 15 | FAS | Fas cell surface death receptor [Source:HGNC Symbol;Acc:HGNC:11920] | 418 | 1.154 | 0.4174 | Yes |
| 16 | IL2RG | interleukin 2 receptor subunit gamma [Source:HGNC Symbol;Acc:HGNC:6010] | 432 | 1.141 | 0.4379 | Yes |
| 17 | CXCL9 | C-X-C motif chemokine ligand 9 [Source:HGNC Symbol;Acc:HGNC:7098] | 473 | 1.098 | 0.4559 | Yes |
| 18 | IL15RA | interleukin 15 receptor subunit alpha [Source:HGNC Symbol;Acc:HGNC:5978] | 494 | 1.080 | 0.4749 | Yes |
| 19 | ITGA4 | integrin subunit alpha 4 [Source:HGNC Symbol;Acc:HGNC:6140] | 551 | 1.029 | 0.4906 | Yes |
| 20 | CSF2RB | colony stimulating factor 2 receptor subunit beta [Source:HGNC Symbol;Acc:HGNC:2436] | 561 | 1.019 | 0.5091 | Yes |
| 21 | LTB | lymphotoxin beta [Source:HGNC Symbol;Acc:HGNC:6711] | 593 | 1.000 | 0.5258 | Yes |
| 22 | IL12RB1 | interleukin 12 receptor subunit beta 1 [Source:HGNC Symbol;Acc:HGNC:5971] | 705 | 0.928 | 0.5361 | Yes |
| 23 | CXCL13 | C-X-C motif chemokine ligand 13 [Source:HGNC Symbol;Acc:HGNC:10639] | 710 | 0.927 | 0.5532 | Yes |
| 24 | CNTFR | ciliary neurotrophic factor receptor [Source:HGNC Symbol;Acc:HGNC:2170] | 760 | 0.892 | 0.5668 | Yes |
| 25 | TNFRSF1B | TNF receptor superfamily member 1B [Source:HGNC Symbol;Acc:HGNC:11917] | 845 | 0.864 | 0.5775 | Yes |
| 26 | EBI3 | Epstein-Barr virus induced 3 [Source:HGNC Symbol;Acc:HGNC:3129] | 852 | 0.862 | 0.5933 | Yes |
| 27 | CCR1 | C-C motif chemokine receptor 1 [Source:HGNC Symbol;Acc:HGNC:1602] | 866 | 0.853 | 0.6084 | Yes |
| 28 | REG1A | regenerating family member 1 alpha [Source:HGNC Symbol;Acc:HGNC:9951] | 947 | 0.837 | 0.6190 | Yes |
| 29 | SOCS1 | suppressor of cytokine signaling 1 [Source:HGNC Symbol;Acc:HGNC:19383] | 1359 | 0.674 | 0.6052 | Yes |
| 30 | IL13RA1 | interleukin 13 receptor subunit alpha 1 [Source:HGNC Symbol;Acc:HGNC:5974] | 1511 | 0.623 | 0.6072 | Yes |
| 31 | ACVRL1 | activin A receptor like type 1 [Source:HGNC Symbol;Acc:HGNC:175] | 1555 | 0.608 | 0.6158 | Yes |
| 32 | PIM1 | "Pim-1 proto-oncogene, serine/threonine kinase [Source:HGNC Symbol;Acc:HGNC:8986]" | 1567 | 0.607 | 0.6265 | Yes |
| 33 | A2M | alpha-2-macroglobulin [Source:HGNC Symbol;Acc:HGNC:7] | 1612 | 0.591 | 0.6347 | Yes |
| 34 | IL10RB | interleukin 10 receptor subunit beta [Source:HGNC Symbol;Acc:HGNC:5965] | 1772 | 0.551 | 0.6348 | Yes |
| 35 | TNF | tumor necrosis factor [Source:HGNC Symbol;Acc:HGNC:11892] | 1880 | 0.528 | 0.6379 | Yes |
| 36 | CSF2 | colony stimulating factor 2 [Source:HGNC Symbol;Acc:HGNC:2434] | 1913 | 0.521 | 0.6456 | Yes |
| 37 | PIK3R5 | phosphoinositide-3-kinase regulatory subunit 5 [Source:HGNC Symbol;Acc:HGNC:30035] | 2331 | 0.464 | 0.6275 | No |
| 38 | BAK1 | BCL2 antagonist/killer 1 [Source:HGNC Symbol;Acc:HGNC:949] | 2363 | 0.457 | 0.6341 | No |
| 39 | INHBE | inhibin subunit beta E [Source:HGNC Symbol;Acc:HGNC:24029] | 2487 | 0.445 | 0.6345 | No |
| 40 | CCL7 | C-C motif chemokine ligand 7 [Source:HGNC Symbol;Acc:HGNC:10634] | 2997 | 0.404 | 0.6094 | No |
| 41 | MYD88 | MYD88 innate immune signal transduction adaptor [Source:HGNC Symbol;Acc:HGNC:7562] | 3202 | 0.373 | 0.6033 | No |
| 42 | IL4R | interleukin 4 receptor [Source:HGNC Symbol;Acc:HGNC:6015] | 3233 | 0.368 | 0.6083 | No |
| 43 | CXCL1 | C-X-C motif chemokine ligand 1 [Source:HGNC Symbol;Acc:HGNC:4602] | 3324 | 0.354 | 0.6091 | No |
| 44 | SOCS3 | suppressor of cytokine signaling 3 [Source:HGNC Symbol;Acc:HGNC:19391] | 3330 | 0.352 | 0.6154 | No |
| 45 | CSF2RA | colony stimulating factor 2 receptor subunit alpha [Source:HGNC Symbol;Acc:HGNC:2435] | 3459 | 0.333 | 0.6134 | No |
| 46 | IFNGR2 | interferon gamma receptor 2 [Source:HGNC Symbol;Acc:HGNC:5440] | 3530 | 0.323 | 0.6150 | No |
| 47 | IFNAR1 | interferon alpha and beta receptor subunit 1 [Source:HGNC Symbol;Acc:HGNC:5432] | 3890 | 0.280 | 0.5972 | No |
| 48 | IL7 | interleukin 7 [Source:HGNC Symbol;Acc:HGNC:6023] | 4006 | 0.265 | 0.5948 | No |
| 49 | CRLF2 | cytokine receptor like factor 2 [Source:HGNC Symbol;Acc:HGNC:14281] | 4085 | 0.256 | 0.5946 | No |
| 50 | IL3RA | interleukin 3 receptor subunit alpha [Source:HGNC Symbol;Acc:HGNC:6012] | 4139 | 0.250 | 0.5958 | No |
| 51 | CXCL3 | C-X-C motif chemokine ligand 3 [Source:HGNC Symbol;Acc:HGNC:4604] | 4256 | 0.236 | 0.5928 | No |
| 52 | STAT3 | signal transducer and activator of transcription 3 [Source:HGNC Symbol;Acc:HGNC:11364] | 4571 | 0.207 | 0.5766 | No |
| 53 | ACVR1B | activin A receptor type 1B [Source:HGNC Symbol;Acc:HGNC:172] | 4621 | 0.203 | 0.5772 | No |
| 54 | CD36 | CD36 molecule [Source:HGNC Symbol;Acc:HGNC:1663] | 4685 | 0.198 | 0.5769 | No |
| 55 | PF4 | platelet factor 4 [Source:HGNC Symbol;Acc:HGNC:8861] | 4836 | 0.184 | 0.5707 | No |
| 56 | PTPN2 | protein tyrosine phosphatase non-receptor type 2 [Source:HGNC Symbol;Acc:HGNC:9650] | 5042 | 0.164 | 0.5606 | No |
| 57 | CD38 | CD38 molecule [Source:HGNC Symbol;Acc:HGNC:1667] | 5197 | 0.151 | 0.5536 | No |
| 58 | PTPN1 | protein tyrosine phosphatase non-receptor type 1 [Source:HGNC Symbol;Acc:HGNC:9642] | 5216 | 0.150 | 0.5552 | No |
| 59 | GRB2 | growth factor receptor bound protein 2 [Source:HGNC Symbol;Acc:HGNC:4566] | 5321 | 0.141 | 0.5512 | No |
| 60 | CD14 | CD14 molecule [Source:HGNC Symbol;Acc:HGNC:1628] | 5473 | 0.129 | 0.5439 | No |
| 61 | TYK2 | tyrosine kinase 2 [Source:HGNC Symbol;Acc:HGNC:12440] | 5521 | 0.126 | 0.5433 | No |
| 62 | ITGB3 | integrin subunit beta 3 [Source:HGNC Symbol;Acc:HGNC:6156] | 5695 | 0.113 | 0.5343 | No |
| 63 | CBL | Cbl proto-oncogene [Source:HGNC Symbol;Acc:HGNC:1541] | 5833 | 0.103 | 0.5274 | No |
| 64 | TGFB1 | transforming growth factor beta 1 [Source:HGNC Symbol;Acc:HGNC:11766] | 6038 | 0.088 | 0.5160 | No |
| 65 | TNFRSF21 | TNF receptor superfamily member 21 [Source:HGNC Symbol;Acc:HGNC:13469] | 6692 | 0.043 | 0.4749 | No |
| 66 | HMOX1 | heme oxygenase 1 [Source:HGNC Symbol;Acc:HGNC:5013] | 6937 | 0.027 | 0.4597 | No |
| 67 | TNFRSF1A | TNF receptor superfamily member 1A [Source:HGNC Symbol;Acc:HGNC:11916] | 7339 | 0.004 | 0.4341 | No |
| 68 | IFNGR1 | interferon gamma receptor 1 [Source:HGNC Symbol;Acc:HGNC:5439] | 7599 | -0.009 | 0.4176 | No |
| 69 | IL17RA | interleukin 17 receptor A [Source:HGNC Symbol;Acc:HGNC:5985] | 7685 | -0.013 | 0.4124 | No |
| 70 | PTPN11 | protein tyrosine phosphatase non-receptor type 11 [Source:HGNC Symbol;Acc:HGNC:9644] | 8451 | -0.053 | 0.3643 | No |
| 71 | OSMR | oncostatin M receptor [Source:HGNC Symbol;Acc:HGNC:8507] | 8832 | -0.074 | 0.3413 | No |
| 72 | TLR2 | toll like receptor 2 [Source:HGNC Symbol;Acc:HGNC:11848] | 9404 | -0.105 | 0.3066 | No |
| 73 | IL1R1 | interleukin 1 receptor type 1 [Source:HGNC Symbol;Acc:HGNC:5993] | 9556 | -0.115 | 0.2991 | No |
| 74 | STAM2 | signal transducing adaptor molecule 2 [Source:HGNC Symbol;Acc:HGNC:11358] | 10125 | -0.149 | 0.2654 | No |
| 75 | JUN | "Jun proto-oncogene, AP-1 transcription factor subunit [Source:HGNC Symbol;Acc:HGNC:6204]" | 10257 | -0.157 | 0.2599 | No |
| 76 | DNTT | DNA nucleotidylexotransferase [Source:HGNC Symbol;Acc:HGNC:2983] | 10602 | -0.177 | 0.2412 | No |
| 77 | CD44 | CD44 molecule (Indian blood group) [Source:HGNC Symbol;Acc:HGNC:1681] | 10617 | -0.178 | 0.2436 | No |
| 78 | IL6ST | interleukin 6 signal transducer [Source:HGNC Symbol;Acc:HGNC:6021] | 12152 | -0.270 | 0.1502 | No |
| 79 | LTBR | lymphotoxin beta receptor [Source:HGNC Symbol;Acc:HGNC:6718] | 12413 | -0.289 | 0.1390 | No |
| 80 | HAX1 | HCLS1 associated protein X-1 [Source:HGNC Symbol;Acc:HGNC:16915] | 13055 | -0.343 | 0.1043 | No |
| 81 | IL17RB | interleukin 17 receptor B [Source:HGNC Symbol;Acc:HGNC:18015] | 13115 | -0.348 | 0.1070 | No |
| 82 | CD9 | CD9 molecule [Source:HGNC Symbol;Acc:HGNC:1709] | 13472 | -0.388 | 0.0914 | No |
| 83 | LEPR | leptin receptor [Source:HGNC Symbol;Acc:HGNC:6554] | 14583 | -0.529 | 0.0301 | No |
| 84 | CSF1 | colony stimulating factor 1 [Source:HGNC Symbol;Acc:HGNC:2432] | 14746 | -0.566 | 0.0303 | No |
| 85 | TNFRSF12A | TNF receptor superfamily member 12A [Source:HGNC Symbol;Acc:HGNC:18152] | 14839 | -0.595 | 0.0356 | No |
| 86 | PDGFC | platelet derived growth factor C [Source:HGNC Symbol;Acc:HGNC:8801] | 15439 | -0.939 | 0.0147 | No |
Table: GSEA details [plain text format]

  

Fig 2: HALLMARK\_IL6\_JAK\_STAT3\_SIGNALING      
 Blue-Pink O' Gram in the Space of the Analyzed GeneSet

  

Fig 3: HALLMARK\_IL6\_JAK\_STAT3\_SIGNALING: Random ES distribution      
 Gene set null distribution of ES for **HALLMARK\_IL6\_JAK\_STAT3\_SIGNALING**
