# Supplementary material for: In vivo CRISPR screens reveal Serpinb9 and Adam2 as regulators of immune therapy response in lung cancer
Source: Nat Commun. 2023 May 31;14:3150. doi: 10.1038/s41467-023-38841-7 (PMC10232477; doi:10.1038/s41467-023-38841-7)
Supplement: Supplementary file 13 — Source Data [file 41467_2023_38841_MOESM13_ESM.zip › Figure 4E.zip!/my_analysis.Gsea.1640146340255_Hallmark/HALLMARK_XENOBIOTIC_METABOLISM.html]

Details for gene set HALLMARK\_XENOBIOTIC\_METABOLISM[GSEA]

|  || Dataset | December 21, 2021\_for GSEA run\_collapsed\_to\_symbols.GSEA\_B6\_NI.cls #CTLR\_NI\_versus\_ADAM2\_NI |
| Phenotype | GSEA\_B6\_NI.cls#CTLR\_NI\_versus\_ADAM2\_NI |
| Upregulated in class | CTLR\_NI |
| GeneSet | HALLMARK\_XENOBIOTIC\_METABOLISM |
| Enrichment Score (ES) | 0.31249046 |
| Normalized Enrichment Score (NES) | 1.1450998 |
| Nominal p-value | 0.3258427 |
| FDR q-value | 0.6484542 |
| FWER p-Value | 1.0 |
Table: GSEA Results Summary

  

Fig 1: Enrichment plot: HALLMARK\_XENOBIOTIC\_METABOLISM      
 Profile of the Running ES Score & Positions of GeneSet Members on the Rank Ordered List

  

| SYMBOL | TITLE | RANK IN GENE LIST | RANK METRIC SCORE | RUNNING ES | CORE ENRICHMENT || 1 | TDO2 | "tryptophan 2,3-dioxygenase [Source:HGNC Symbol;Acc:HGNC:11708]" | 170 | 1.587 | 0.0120 | Yes |
| 2 | UPP1 | uridine phosphorylase 1 [Source:HGNC Symbol;Acc:HGNC:12576] | 191 | 1.509 | 0.0326 | Yes |
| 3 | GSTT2 | glutathione S-transferase theta 2 (gene/pseudogene) [Source:HGNC Symbol;Acc:HGNC:4642] | 229 | 1.414 | 0.0508 | Yes |
| 4 | CFB | complement factor B [Source:HGNC Symbol;Acc:HGNC:1037] | 245 | 1.387 | 0.0699 | Yes |
| 5 | ARG2 | arginase 2 [Source:HGNC Symbol;Acc:HGNC:664] | 280 | 1.338 | 0.0871 | Yes |
| 6 | HSD11B1 | hydroxysteroid 11-beta dehydrogenase 1 [Source:HGNC Symbol;Acc:HGNC:5208] | 291 | 1.319 | 0.1056 | Yes |
| 7 | CYFIP2 | cytoplasmic FMR1 interacting protein 2 [Source:HGNC Symbol;Acc:HGNC:13760] | 304 | 1.294 | 0.1236 | Yes |
| 8 | PSMB10 | proteasome 20S subunit beta 10 [Source:HGNC Symbol;Acc:HGNC:9538] | 326 | 1.261 | 0.1405 | Yes |
| 9 | FAS | Fas cell surface death receptor [Source:HGNC Symbol;Acc:HGNC:11920] | 418 | 1.154 | 0.1514 | Yes |
| 10 | XDH | xanthine dehydrogenase [Source:HGNC Symbol;Acc:HGNC:12805] | 449 | 1.119 | 0.1657 | Yes |
| 11 | LEAP2 | liver enriched antimicrobial peptide 2 [Source:HGNC Symbol;Acc:HGNC:29571] | 582 | 1.007 | 0.1717 | Yes |
| 12 | GSTM4 | glutathione S-transferase mu 4 [Source:HGNC Symbol;Acc:HGNC:4636] | 646 | 0.966 | 0.1817 | Yes |
| 13 | TTPA | alpha tocopherol transfer protein [Source:HGNC Symbol;Acc:HGNC:12404] | 768 | 0.892 | 0.1868 | Yes |
| 14 | REG1A | regenerating family member 1 alpha [Source:HGNC Symbol;Acc:HGNC:9951] | 947 | 0.837 | 0.1874 | Yes |
| 15 | PAPSS2 | 3'-phosphoadenosine 5'-phosphosulfate synthase 2 [Source:HGNC Symbol;Acc:HGNC:8604] | 985 | 0.828 | 0.1971 | Yes |
| 16 | CYP2J2 | cytochrome P450 family 2 subfamily J member 2 [Source:HGNC Symbol;Acc:HGNC:2634] | 1117 | 0.765 | 0.1997 | Yes |
| 17 | CYP26A1 | cytochrome P450 family 26 subfamily A member 1 [Source:HGNC Symbol;Acc:HGNC:2603] | 1126 | 0.761 | 0.2102 | Yes |
| 18 | CNDP2 | carnosine dipeptidase 2 [Source:HGNC Symbol;Acc:HGNC:24437] | 1199 | 0.732 | 0.2162 | Yes |
| 19 | F10 | coagulation factor X [Source:HGNC Symbol;Acc:HGNC:3528] | 1225 | 0.723 | 0.2251 | Yes |
| 20 | ADH7 | "alcohol dehydrogenase 7 (class IV), mu or sigma polypeptide [Source:HGNC Symbol;Acc:HGNC:256]" | 1273 | 0.704 | 0.2322 | Yes |
| 21 | IRF8 | interferon regulatory factor 8 [Source:HGNC Symbol;Acc:HGNC:5358] | 1378 | 0.667 | 0.2352 | Yes |
| 22 | DDC | dopa decarboxylase [Source:HGNC Symbol;Acc:HGNC:2719] | 1468 | 0.637 | 0.2387 | Yes |
| 23 | ARG1 | arginase 1 [Source:HGNC Symbol;Acc:HGNC:663] | 1507 | 0.624 | 0.2453 | Yes |
| 24 | CES1 | carboxylesterase 1 [Source:HGNC Symbol;Acc:HGNC:1863] | 1509 | 0.624 | 0.2543 | Yes |
| 25 | ANGPTL3 | angiopoietin like 3 [Source:HGNC Symbol;Acc:HGNC:491] | 1518 | 0.620 | 0.2627 | Yes |
| 26 | SLC46A3 | solute carrier family 46 member 3 [Source:HGNC Symbol;Acc:HGNC:27501] | 1548 | 0.610 | 0.2697 | Yes |
| 27 | CDO1 | cysteine dioxygenase type 1 [Source:HGNC Symbol;Acc:HGNC:1795] | 1550 | 0.610 | 0.2785 | Yes |
| 28 | PDK4 | pyruvate dehydrogenase kinase 4 [Source:HGNC Symbol;Acc:HGNC:8812] | 1755 | 0.554 | 0.2734 | Yes |
| 29 | SLC6A12 | solute carrier family 6 member 12 [Source:HGNC Symbol;Acc:HGNC:11045] | 1761 | 0.553 | 0.2811 | Yes |
| 30 | NDRG2 | NDRG family member 2 [Source:HGNC Symbol;Acc:HGNC:14460] | 1803 | 0.543 | 0.2863 | Yes |
| 31 | CYP2E1 | cytochrome P450 family 2 subfamily E member 1 [Source:HGNC Symbol;Acc:HGNC:2631] | 1945 | 0.515 | 0.2846 | Yes |
| 32 | ABCD2 | ATP binding cassette subfamily D member 2 [Source:HGNC Symbol;Acc:HGNC:66] | 1973 | 0.508 | 0.2903 | Yes |
| 33 | BCAT1 | branched chain amino acid transaminase 1 [Source:HGNC Symbol;Acc:HGNC:976] | 1979 | 0.507 | 0.2973 | Yes |
| 34 | ABCC2 | ATP binding cassette subfamily C member 2 [Source:HGNC Symbol;Acc:HGNC:53] | 2147 | 0.490 | 0.2936 | Yes |
| 35 | IGFBP4 | insulin like growth factor binding protein 4 [Source:HGNC Symbol;Acc:HGNC:5473] | 2183 | 0.482 | 0.2984 | Yes |
| 36 | ITIH4 | inter-alpha-trypsin inhibitor heavy chain 4 [Source:HGNC Symbol;Acc:HGNC:6169] | 2265 | 0.474 | 0.3000 | Yes |
| 37 | SMOX | spermine oxidase [Source:HGNC Symbol;Acc:HGNC:15862] | 2328 | 0.465 | 0.3027 | Yes |
| 38 | ACOX2 | acyl-CoA oxidase 2 [Source:HGNC Symbol;Acc:HGNC:120] | 2464 | 0.445 | 0.3005 | Yes |
| 39 | CYP4F2 | cytochrome P450 family 4 subfamily F member 2 [Source:HGNC Symbol;Acc:HGNC:2645] | 2525 | 0.445 | 0.3030 | Yes |
| 40 | GCH1 | GTP cyclohydrolase 1 [Source:HGNC Symbol;Acc:HGNC:4193] | 2566 | 0.441 | 0.3069 | Yes |
| 41 | FMO1 | flavin containing dimethylaniline monoxygenase 1 [Source:HGNC Symbol;Acc:HGNC:3769] | 2578 | 0.437 | 0.3125 | Yes |
| 42 | F11 | coagulation factor XI [Source:HGNC Symbol;Acc:HGNC:3529] | 2839 | 0.411 | 0.3017 | No |
| 43 | GNMT | glycine N-methyltransferase [Source:HGNC Symbol;Acc:HGNC:4415] | 3059 | 0.394 | 0.2932 | No |
| 44 | PTS | 6-pyruvoyltetrahydropterin synthase [Source:HGNC Symbol;Acc:HGNC:9689] | 3114 | 0.386 | 0.2954 | No |
| 45 | NPC1 | NPC intracellular cholesterol transporter 1 [Source:HGNC Symbol;Acc:HGNC:7897] | 3270 | 0.363 | 0.2906 | No |
| 46 | ENPEP | glutamyl aminopeptidase [Source:HGNC Symbol;Acc:HGNC:3355] | 3359 | 0.348 | 0.2900 | No |
| 47 | LCAT | lecithin-cholesterol acyltransferase [Source:HGNC Symbol;Acc:HGNC:6522] | 3393 | 0.343 | 0.2928 | No |
| 48 | GCNT2 | glucosaminyl (N-acetyl) transferase 2 (I blood group) [Source:HGNC Symbol;Acc:HGNC:4204] | 3433 | 0.337 | 0.2952 | No |
| 49 | ACP1 | acid phosphatase 1 [Source:HGNC Symbol;Acc:HGNC:122] | 3457 | 0.334 | 0.2985 | No |
| 50 | AQP9 | aquaporin 9 [Source:HGNC Symbol;Acc:HGNC:643] | 3493 | 0.329 | 0.3010 | No |
| 51 | ETS2 | "ETS proto-oncogene 2, transcription factor [Source:HGNC Symbol;Acc:HGNC:3489]" | 3538 | 0.323 | 0.3029 | No |
| 52 | CBR1 | carbonyl reductase 1 [Source:HGNC Symbol;Acc:HGNC:1548] | 3640 | 0.309 | 0.3008 | No |
| 53 | FBLN1 | fibulin 1 [Source:HGNC Symbol;Acc:HGNC:3600] | 3838 | 0.286 | 0.2923 | No |
| 54 | GART | "phosphoribosylglycinamide formyltransferase, phosphoribosylglycinamide synthetase, phosphoribosylaminoimidazole synthetase [Source:HGNC Symbol;Acc:HGNC:4163]" | 4180 | 0.246 | 0.2738 | No |
| 55 | CYB5A | cytochrome b5 type A [Source:HGNC Symbol;Acc:HGNC:2570] | 4318 | 0.231 | 0.2683 | No |
| 56 | CASP6 | caspase 6 [Source:HGNC Symbol;Acc:HGNC:1507] | 4374 | 0.225 | 0.2680 | No |
| 57 | MARCHF6 | membrane associated ring-CH-type finger 6 [Source:HGNC Symbol;Acc:HGNC:30550] | 4487 | 0.214 | 0.2639 | No |
| 58 | GSR | glutathione-disulfide reductase [Source:HGNC Symbol;Acc:HGNC:4623] | 4657 | 0.200 | 0.2559 | No |
| 59 | CD36 | CD36 molecule [Source:HGNC Symbol;Acc:HGNC:1663] | 4685 | 0.198 | 0.2570 | No |
| 60 | ACOX3 | "acyl-CoA oxidase 3, pristanoyl [Source:HGNC Symbol;Acc:HGNC:121]" | 4903 | 0.177 | 0.2456 | No |
| 61 | APOE | apolipoprotein E [Source:HGNC Symbol;Acc:HGNC:613] | 4918 | 0.176 | 0.2472 | No |
| 62 | KYNU | kynureninase [Source:HGNC Symbol;Acc:HGNC:6469] | 5014 | 0.166 | 0.2435 | No |
| 63 | COMT | catechol-O-methyltransferase [Source:HGNC Symbol;Acc:HGNC:2228] | 5059 | 0.162 | 0.2430 | No |
| 64 | SLC1A5 | solute carrier family 1 member 5 [Source:HGNC Symbol;Acc:HGNC:10943] | 5126 | 0.156 | 0.2410 | No |
| 65 | TMBIM6 | transmembrane BAX inhibitor motif containing 6 [Source:HGNC Symbol;Acc:HGNC:11723] | 5276 | 0.144 | 0.2335 | No |
| 66 | CROT | carnitine O-octanoyltransferase [Source:HGNC Symbol;Acc:HGNC:2366] | 5358 | 0.138 | 0.2303 | No |
| 67 | CYP27A1 | cytochrome P450 family 27 subfamily A member 1 [Source:HGNC Symbol;Acc:HGNC:2605] | 5408 | 0.134 | 0.2291 | No |
| 68 | RBP4 | retinol binding protein 4 [Source:HGNC Symbol;Acc:HGNC:9922] | 5444 | 0.132 | 0.2287 | No |
| 69 | DHRS7 | dehydrogenase/reductase 7 [Source:HGNC Symbol;Acc:HGNC:21524] | 5629 | 0.117 | 0.2185 | No |
| 70 | DHRS1 | dehydrogenase/reductase 1 [Source:HGNC Symbol;Acc:HGNC:16445] | 5779 | 0.107 | 0.2105 | No |
| 71 | FMO3 | flavin containing dimethylaniline monoxygenase 3 [Source:HGNC Symbol;Acc:HGNC:3771] | 5815 | 0.104 | 0.2097 | No |
| 72 | LPIN2 | lipin 2 [Source:HGNC Symbol;Acc:HGNC:14450] | 5857 | 0.102 | 0.2085 | No |
| 73 | PROS1 | protein S [Source:HGNC Symbol;Acc:HGNC:9456] | 5860 | 0.102 | 0.2099 | No |
| 74 | ACP2 | "acid phosphatase 2, lysosomal [Source:HGNC Symbol;Acc:HGNC:123]" | 5908 | 0.098 | 0.2083 | No |
| 75 | DDT | D-dopachrome tautomerase [Source:HGNC Symbol;Acc:HGNC:2732] | 5988 | 0.092 | 0.2045 | No |
| 76 | ABCC3 | ATP binding cassette subfamily C member 3 [Source:HGNC Symbol;Acc:HGNC:54] | 6098 | 0.083 | 0.1987 | No |
| 77 | ABHD6 | "abhydrolase domain containing 6, acylglycerol lipase [Source:HGNC Symbol;Acc:HGNC:21398]" | 6230 | 0.075 | 0.1913 | No |
| 78 | FAH | fumarylacetoacetate hydrolase [Source:HGNC Symbol;Acc:HGNC:3579] | 6356 | 0.066 | 0.1842 | No |
| 79 | MTHFD1 | "methylenetetrahydrofolate dehydrogenase, cyclohydrolase and formyltetrahydrofolate synthetase 1 [Source:HGNC Symbol;Acc:HGNC:7432]" | 6407 | 0.062 | 0.1819 | No |
| 80 | RAP1GAP | RAP1 GTPase activating protein [Source:HGNC Symbol;Acc:HGNC:9858] | 6458 | 0.057 | 0.1795 | No |
| 81 | ALDH9A1 | aldehyde dehydrogenase 9 family member A1 [Source:HGNC Symbol;Acc:HGNC:412] | 6489 | 0.056 | 0.1783 | No |
| 82 | HMOX1 | heme oxygenase 1 [Source:HGNC Symbol;Acc:HGNC:5013] | 6937 | 0.027 | 0.1499 | No |
| 83 | FBP1 | fructose-bisphosphatase 1 [Source:HGNC Symbol;Acc:HGNC:3606] | 6968 | 0.026 | 0.1483 | No |
| 84 | VNN1 | vanin 1 [Source:HGNC Symbol;Acc:HGNC:12705] | 7087 | 0.019 | 0.1410 | No |
| 85 | PDLIM5 | PDZ and LIM domain 5 [Source:HGNC Symbol;Acc:HGNC:17468] | 7279 | 0.008 | 0.1287 | No |
| 86 | SERTAD1 | SERTA domain containing 1 [Source:HGNC Symbol;Acc:HGNC:17932] | 7308 | 0.006 | 0.1270 | No |
| 87 | TNFRSF1A | TNF receptor superfamily member 1A [Source:HGNC Symbol;Acc:HGNC:11916] | 7339 | 0.004 | 0.1251 | No |
| 88 | BLVRB | biliverdin reductase B [Source:HGNC Symbol;Acc:HGNC:1063] | 7346 | 0.004 | 0.1248 | No |
| 89 | SHMT2 | serine hydroxymethyltransferase 2 [Source:HGNC Symbol;Acc:HGNC:10852] | 7477 | -0.003 | 0.1165 | No |
| 90 | ALAS1 | 5'-aminolevulinate synthase 1 [Source:HGNC Symbol;Acc:HGNC:396] | 7482 | -0.004 | 0.1163 | No |
| 91 | ITIH1 | inter-alpha-trypsin inhibitor heavy chain 1 [Source:HGNC Symbol;Acc:HGNC:6166] | 7544 | -0.006 | 0.1124 | No |
| 92 | CYP2S1 | cytochrome P450 family 2 subfamily S member 1 [Source:HGNC Symbol;Acc:HGNC:15654] | 7745 | -0.016 | 0.0997 | No |
| 93 | FETUB | fetuin B [Source:HGNC Symbol;Acc:HGNC:3658] | 7799 | -0.019 | 0.0966 | No |
| 94 | MAN1A1 | mannosidase alpha class 1A member 1 [Source:HGNC Symbol;Acc:HGNC:6821] | 7884 | -0.023 | 0.0915 | No |
| 95 | IDH1 | isocitrate dehydrogenase (NADP(+)) 1 [Source:HGNC Symbol;Acc:HGNC:5382] | 8014 | -0.030 | 0.0836 | No |
| 96 | ARPP19 | cAMP regulated phosphoprotein 19 [Source:HGNC Symbol;Acc:HGNC:16967] | 8116 | -0.036 | 0.0776 | No |
| 97 | UGDH | UDP-glucose 6-dehydrogenase [Source:HGNC Symbol;Acc:HGNC:12525] | 8375 | -0.047 | 0.0616 | No |
| 98 | ID2 | inhibitor of DNA binding 2 [Source:HGNC Symbol;Acc:HGNC:5361] | 8387 | -0.048 | 0.0616 | No |
| 99 | AP4B1 | adaptor related protein complex 4 subunit beta 1 [Source:HGNC Symbol;Acc:HGNC:572] | 8465 | -0.053 | 0.0574 | No |
| 100 | SLC35D1 | solute carrier family 35 member D1 [Source:HGNC Symbol;Acc:HGNC:20800] | 8512 | -0.055 | 0.0553 | No |
| 101 | PPARD | peroxisome proliferator activated receptor delta [Source:HGNC Symbol;Acc:HGNC:9235] | 8546 | -0.058 | 0.0540 | No |
| 102 | POR | cytochrome p450 oxidoreductase [Source:HGNC Symbol;Acc:HGNC:9208] | 8699 | -0.066 | 0.0451 | No |
| 103 | SAR1B | secretion associated Ras related GTPase 1B [Source:HGNC Symbol;Acc:HGNC:10535] | 8838 | -0.075 | 0.0373 | No |
| 104 | IGF1 | insulin like growth factor 1 [Source:HGNC Symbol;Acc:HGNC:5464] | 8926 | -0.078 | 0.0328 | No |
| 105 | ASL | argininosuccinate lyase [Source:HGNC Symbol;Acc:HGNC:746] | 8934 | -0.079 | 0.0335 | No |
| 106 | CAT | catalase [Source:HGNC Symbol;Acc:HGNC:1516] | 9069 | -0.086 | 0.0261 | No |
| 107 | PTGES3 | prostaglandin E synthase 3 [Source:HGNC Symbol;Acc:HGNC:16049] | 9118 | -0.089 | 0.0243 | No |
| 108 | KARS1 | lysyl-tRNA synthetase 1 [Source:HGNC Symbol;Acc:HGNC:6215] | 9351 | -0.101 | 0.0108 | No |
| 109 | PTGR1 | prostaglandin reductase 1 [Source:HGNC Symbol;Acc:HGNC:18429] | 9371 | -0.103 | 0.0110 | No |
| 110 | LONP1 | "lon peptidase 1, mitochondrial [Source:HGNC Symbol;Acc:HGNC:9479]" | 9471 | -0.108 | 0.0062 | No |
| 111 | IL1R1 | interleukin 1 receptor type 1 [Source:HGNC Symbol;Acc:HGNC:5993] | 9556 | -0.115 | 0.0024 | No |
| 112 | HPRT1 | hypoxanthine phosphoribosyltransferase 1 [Source:HGNC Symbol;Acc:HGNC:5157] | 9568 | -0.116 | 0.0034 | No |
| 113 | ACO2 | aconitase 2 [Source:HGNC Symbol;Acc:HGNC:118] | 9744 | -0.127 | -0.0060 | No |
| 114 | GCLC | glutamate-cysteine ligase catalytic subunit [Source:HGNC Symbol;Acc:HGNC:4311] | 9762 | -0.129 | -0.0053 | No |
| 115 | NINJ1 | ninjurin 1 [Source:HGNC Symbol;Acc:HGNC:7824] | 9852 | -0.134 | -0.0091 | No |
| 116 | ATOH8 | atonal bHLH transcription factor 8 [Source:HGNC Symbol;Acc:HGNC:24126] | 9891 | -0.136 | -0.0096 | No |
| 117 | PEMT | phosphatidylethanolamine N-methyltransferase [Source:HGNC Symbol;Acc:HGNC:8830] | 9918 | -0.137 | -0.0092 | No |
| 118 | SPINT2 | "serine peptidase inhibitor, Kunitz type 2 [Source:HGNC Symbol;Acc:HGNC:11247]" | 9981 | -0.141 | -0.0112 | No |
| 119 | SERPINE1 | serpin family E member 1 [Source:HGNC Symbol;Acc:HGNC:8583] | 10052 | -0.145 | -0.0136 | No |
| 120 | GSS | glutathione synthetase [Source:HGNC Symbol;Acc:HGNC:4624] | 10461 | -0.169 | -0.0375 | No |
| 121 | DHPS | deoxyhypusine synthase [Source:HGNC Symbol;Acc:HGNC:2869] | 10462 | -0.169 | -0.0351 | No |
| 122 | GABARAPL1 | GABA type A receptor associated protein like 1 [Source:HGNC Symbol;Acc:HGNC:4068] | 10566 | -0.175 | -0.0392 | No |
| 123 | NMT1 | N-myristoyltransferase 1 [Source:HGNC Symbol;Acc:HGNC:7857] | 10801 | -0.188 | -0.0515 | No |
| 124 | NFS1 | NFS1 cysteine desulfurase [Source:HGNC Symbol;Acc:HGNC:15910] | 10802 | -0.188 | -0.0488 | No |
| 125 | CSAD | cysteine sulfinic acid decarboxylase [Source:HGNC Symbol;Acc:HGNC:18966] | 10890 | -0.193 | -0.0516 | No |
| 126 | VTN | vitronectin [Source:HGNC Symbol;Acc:HGNC:12724] | 10896 | -0.194 | -0.0491 | No |
| 127 | ATP2A2 | ATPase sarcoplasmic/endoplasmic reticulum Ca2+ transporting 2 [Source:HGNC Symbol;Acc:HGNC:812] | 11020 | -0.201 | -0.0542 | No |
| 128 | MCCC2 | methylcrotonoyl-CoA carboxylase 2 [Source:HGNC Symbol;Acc:HGNC:6937] | 11151 | -0.209 | -0.0595 | No |
| 129 | TPST1 | tyrosylprotein sulfotransferase 1 [Source:HGNC Symbol;Acc:HGNC:12020] | 11270 | -0.215 | -0.0640 | No |
| 130 | PYCR1 | pyrroline-5-carboxylate reductase 1 [Source:HGNC Symbol;Acc:HGNC:9721] | 11284 | -0.216 | -0.0617 | No |
| 131 | ADH5 | "alcohol dehydrogenase 5 (class III), chi polypeptide [Source:HGNC Symbol;Acc:HGNC:253]" | 11350 | -0.220 | -0.0627 | No |
| 132 | GCKR | glucokinase regulator [Source:HGNC Symbol;Acc:HGNC:4196] | 11491 | -0.229 | -0.0684 | No |
| 133 | SLC12A4 | solute carrier family 12 member 4 [Source:HGNC Symbol;Acc:HGNC:10913] | 11615 | -0.236 | -0.0730 | No |
| 134 | NQO1 | NAD(P)H quinone dehydrogenase 1 [Source:HGNC Symbol;Acc:HGNC:2874] | 11633 | -0.237 | -0.0706 | No |
| 135 | MAOA | monoamine oxidase A [Source:HGNC Symbol;Acc:HGNC:6833] | 11749 | -0.244 | -0.0745 | No |
| 136 | SLC35B1 | solute carrier family 35 member B1 [Source:HGNC Symbol;Acc:HGNC:20798] | 11784 | -0.247 | -0.0731 | No |
| 137 | ENTPD5 | ectonucleoside triphosphate diphosphohydrolase 5 (inactive) [Source:HGNC Symbol;Acc:HGNC:3367] | 11873 | -0.252 | -0.0752 | No |
| 138 | ACOX1 | acyl-CoA oxidase 1 [Source:HGNC Symbol;Acc:HGNC:119] | 11941 | -0.257 | -0.0758 | No |
| 139 | PTGES | prostaglandin E synthase [Source:HGNC Symbol;Acc:HGNC:9599] | 11951 | -0.257 | -0.0726 | No |
| 140 | AOX1 | aldehyde oxidase 1 [Source:HGNC Symbol;Acc:HGNC:553] | 11955 | -0.258 | -0.0691 | No |
| 141 | ECH1 | enoyl-CoA hydratase 1 [Source:HGNC Symbol;Acc:HGNC:3149] | 12129 | -0.269 | -0.0763 | No |
| 142 | MPP2 | membrane palmitoylated protein 2 [Source:HGNC Symbol;Acc:HGNC:7220] | 12242 | -0.277 | -0.0796 | No |
| 143 | AHCY | adenosylhomocysteinase [Source:HGNC Symbol;Acc:HGNC:343] | 12331 | -0.283 | -0.0811 | No |
| 144 | HGFAC | HGF activator [Source:HGNC Symbol;Acc:HGNC:4894] | 12507 | -0.296 | -0.0881 | No |
| 145 | ELOVL5 | ELOVL fatty acid elongase 5 [Source:HGNC Symbol;Acc:HGNC:21308] | 12900 | -0.328 | -0.1087 | No |
| 146 | TMEM97 | transmembrane protein 97 [Source:HGNC Symbol;Acc:HGNC:28106] | 12936 | -0.332 | -0.1061 | No |
| 147 | BPHL | biphenyl hydrolase like [Source:HGNC Symbol;Acc:HGNC:1094] | 12977 | -0.336 | -0.1039 | No |
| 148 | BCAR1 | "BCAR1 scaffold protein, Cas family member [Source:HGNC Symbol;Acc:HGNC:971]" | 13086 | -0.346 | -0.1058 | No |
| 149 | PGRMC1 | progesterone receptor membrane component 1 [Source:HGNC Symbol;Acc:HGNC:16090] | 13136 | -0.350 | -0.1039 | No |
| 150 | SLC6A6 | solute carrier family 6 member 6 [Source:HGNC Symbol;Acc:HGNC:11052] | 13327 | -0.370 | -0.1108 | No |
| 151 | SSR3 | signal sequence receptor subunit 3 [Source:HGNC Symbol;Acc:HGNC:11325] | 13336 | -0.371 | -0.1059 | No |
| 152 | CCL25 | C-C motif chemokine ligand 25 [Source:HGNC Symbol;Acc:HGNC:10624] | 13369 | -0.376 | -0.1025 | No |
| 153 | PGD | phosphogluconate dehydrogenase [Source:HGNC Symbol;Acc:HGNC:8891] | 13398 | -0.379 | -0.0989 | No |
| 154 | ALDH2 | aldehyde dehydrogenase 2 family member [Source:HGNC Symbol;Acc:HGNC:404] | 13434 | -0.383 | -0.0956 | No |
| 155 | PMM1 | phosphomannomutase 1 [Source:HGNC Symbol;Acc:HGNC:9114] | 13600 | -0.405 | -0.1003 | No |
| 156 | CYP17A1 | cytochrome P450 family 17 subfamily A member 1 [Source:HGNC Symbol;Acc:HGNC:2593] | 13714 | -0.415 | -0.1016 | No |
| 157 | ETFDH | electron transfer flavoprotein dehydrogenase [Source:HGNC Symbol;Acc:HGNC:3483] | 13794 | -0.418 | -0.1007 | No |
| 158 | GSTO1 | glutathione S-transferase omega 1 [Source:HGNC Symbol;Acc:HGNC:13312] | 13804 | -0.419 | -0.0952 | No |
| 159 | CYP1A1 | cytochrome P450 family 1 subfamily A member 1 [Source:HGNC Symbol;Acc:HGNC:2595] | 13890 | -0.428 | -0.0944 | No |
| 160 | ALDH3A1 | aldehyde dehydrogenase 3 family member A1 [Source:HGNC Symbol;Acc:HGNC:405] | 13964 | -0.428 | -0.0929 | No |
| 161 | HES6 | hes family bHLH transcription factor 6 [Source:HGNC Symbol;Acc:HGNC:18254] | 14021 | -0.435 | -0.0903 | No |
| 162 | CDA | cytidine deaminase [Source:HGNC Symbol;Acc:HGNC:1712] | 14101 | -0.449 | -0.0888 | No |
| 163 | TGFB2 | transforming growth factor beta 2 [Source:HGNC Symbol;Acc:HGNC:11768] | 14145 | -0.457 | -0.0850 | No |
| 164 | HACL1 | 2-hydroxyacyl-CoA lyase 1 [Source:HGNC Symbol;Acc:HGNC:17856] | 14181 | -0.463 | -0.0805 | No |
| 165 | PC | pyruvate carboxylase [Source:HGNC Symbol;Acc:HGNC:8636] | 14499 | -0.513 | -0.0936 | No |
| 166 | CYP2C18 | cytochrome P450 family 2 subfamily C member 18 [Source:HGNC Symbol;Acc:HGNC:2620] | 14598 | -0.531 | -0.0922 | No |
| 167 | DCXR | dicarbonyl and L-xylulose reductase [Source:HGNC Symbol;Acc:HGNC:18985] | 14777 | -0.576 | -0.0953 | No |
| 168 | TKFC | triokinase and FMN cyclase [Source:HGNC Symbol;Acc:HGNC:24552] | 14819 | -0.589 | -0.0894 | No |
| 169 | GSTA3 | glutathione S-transferase alpha 3 [Source:HGNC Symbol;Acc:HGNC:4628] | 14856 | -0.601 | -0.0831 | No |
| 170 | RETSAT | retinol saturase [Source:HGNC Symbol;Acc:HGNC:25991] | 14885 | -0.613 | -0.0760 | No |
| 171 | PINK1 | PTEN induced kinase 1 [Source:HGNC Symbol;Acc:HGNC:14581] | 14900 | -0.618 | -0.0679 | No |
| 172 | EPHA2 | EPH receptor A2 [Source:HGNC Symbol;Acc:HGNC:3386] | 15102 | -0.719 | -0.0705 | No |
| 173 | ESR1 | estrogen receptor 1 [Source:HGNC Symbol;Acc:HGNC:3467] | 15139 | -0.744 | -0.0620 | No |
| 174 | GAD1 | glutamate decarboxylase 1 [Source:HGNC Symbol;Acc:HGNC:4092] | 15459 | -0.969 | -0.0686 | No |
| 175 | JUP | junction plakoglobin [Source:HGNC Symbol;Acc:HGNC:6207] | 15477 | -1.001 | -0.0551 | No |
| 176 | TMEM176B | transmembrane protein 176B [Source:HGNC Symbol;Acc:HGNC:29596] | 15507 | -1.048 | -0.0418 | No |
| 177 | CA2 | carbonic anhydrase 2 [Source:HGNC Symbol;Acc:HGNC:1373] | 15522 | -1.087 | -0.0270 | No |
| 178 | DDAH2 | dimethylarginine dimethylaminohydrolase 2 [Source:HGNC Symbol;Acc:HGNC:2716] | 15552 | -1.145 | -0.0122 | No |
| 179 | EPHX1 | epoxide hydrolase 1 [Source:HGNC Symbol;Acc:HGNC:3401] | 15605 | -1.355 | 0.0041 | No |
Table: GSEA details [plain text format]

  

Fig 2: HALLMARK\_XENOBIOTIC\_METABOLISM      
 Blue-Pink O' Gram in the Space of the Analyzed GeneSet

  

Fig 3: HALLMARK\_XENOBIOTIC\_METABOLISM: Random ES distribution      
 Gene set null distribution of ES for **HALLMARK\_XENOBIOTIC\_METABOLISM**
