# Supplementary material for: In vivo CRISPR screens reveal Serpinb9 and Adam2 as regulators of immune therapy response in lung cancer
Source: Nat Commun. 2023 May 31;14:3150. doi: 10.1038/s41467-023-38841-7 (PMC10232477; doi:10.1038/s41467-023-38841-7)
Supplement: Supplementary file 13 — Source Data [file 41467_2023_38841_MOESM13_ESM.zip › Figure 4E.zip!/my_analysis.Gsea.1640146340255_Hallmark/HALLMARK_ALLOGRAFT_REJECTION.html]

Details for gene set HALLMARK\_ALLOGRAFT\_REJECTION[GSEA]

|  || Dataset | December 21, 2021\_for GSEA run\_collapsed\_to\_symbols.GSEA\_B6\_NI.cls #CTLR\_NI\_versus\_ADAM2\_NI |
| Phenotype | GSEA\_B6\_NI.cls#CTLR\_NI\_versus\_ADAM2\_NI |
| Upregulated in class | CTLR\_NI |
| GeneSet | HALLMARK\_ALLOGRAFT\_REJECTION |
| Enrichment Score (ES) | 0.6652 |
| Normalized Enrichment Score (NES) | 1.6435096 |
| Nominal p-value | 0.0 |
| FDR q-value | 0.42925063 |
| FWER p-Value | 0.181 |
Table: GSEA Results Summary

  

Fig 1: Enrichment plot: HALLMARK\_ALLOGRAFT\_REJECTION      
 Profile of the Running ES Score & Positions of GeneSet Members on the Rank Ordered List

  

| SYMBOL | TITLE | RANK IN GENE LIST | RANK METRIC SCORE | RUNNING ES | CORE ENRICHMENT || 1 | HLA-E | "major histocompatibility complex, class I, E [Source:HGNC Symbol;Acc:HGNC:4962]" | 11 | 2.600 | 0.0205 | Yes |
| 2 | KLRD1 | killer cell lectin like receptor D1 [Source:HGNC Symbol;Acc:HGNC:6378] | 26 | 2.367 | 0.0390 | Yes |
| 3 | CD3D | CD3d molecule [Source:HGNC Symbol;Acc:HGNC:1673] | 28 | 2.343 | 0.0581 | Yes |
| 4 | CD3G | CD3g molecule [Source:HGNC Symbol;Acc:HGNC:1675] | 43 | 2.153 | 0.0748 | Yes |
| 5 | STAT1 | signal transducer and activator of transcription 1 [Source:HGNC Symbol;Acc:HGNC:11362] | 45 | 2.090 | 0.0918 | Yes |
| 6 | ZAP70 | zeta chain of T cell receptor associated protein kinase 70 [Source:HGNC Symbol;Acc:HGNC:12858] | 56 | 1.981 | 0.1073 | Yes |
| 7 | CD3E | CD3e molecule [Source:HGNC Symbol;Acc:HGNC:1674] | 62 | 1.950 | 0.1230 | Yes |
| 8 | FASLG | Fas ligand [Source:HGNC Symbol;Acc:HGNC:11936] | 68 | 1.912 | 0.1383 | Yes |
| 9 | ITK | IL2 inducible T cell kinase [Source:HGNC Symbol;Acc:HGNC:6171] | 74 | 1.893 | 0.1534 | Yes |
| 10 | IRF4 | interferon regulatory factor 4 [Source:HGNC Symbol;Acc:HGNC:6119] | 93 | 1.799 | 0.1670 | Yes |
| 11 | CD8A | CD8a molecule [Source:HGNC Symbol;Acc:HGNC:1706] | 99 | 1.789 | 0.1813 | Yes |
| 12 | C2 | complement C2 [Source:HGNC Symbol;Acc:HGNC:1248] | 123 | 1.731 | 0.1939 | Yes |
| 13 | IL2RB | interleukin 2 receptor subunit beta [Source:HGNC Symbol;Acc:HGNC:6009] | 138 | 1.679 | 0.2067 | Yes |
| 14 | CCL5 | C-C motif chemokine ligand 5 [Source:HGNC Symbol;Acc:HGNC:10632] | 169 | 1.591 | 0.2178 | Yes |
| 15 | CD8B | CD8b molecule [Source:HGNC Symbol;Acc:HGNC:1707] | 185 | 1.522 | 0.2293 | Yes |
| 16 | STAT4 | signal transducer and activator of transcription 4 [Source:HGNC Symbol;Acc:HGNC:11365] | 186 | 1.521 | 0.2417 | Yes |
| 17 | CCL22 | C-C motif chemokine ligand 22 [Source:HGNC Symbol;Acc:HGNC:10621] | 196 | 1.500 | 0.2534 | Yes |
| 18 | IL2RA | interleukin 2 receptor subunit alpha [Source:HGNC Symbol;Acc:HGNC:6008] | 205 | 1.469 | 0.2649 | Yes |
| 19 | TAP1 | "transporter 1, ATP binding cassette subfamily B member [Source:HGNC Symbol;Acc:HGNC:43]" | 241 | 1.389 | 0.2740 | Yes |
| 20 | IL10 | interleukin 10 [Source:HGNC Symbol;Acc:HGNC:5962] | 246 | 1.386 | 0.2851 | Yes |
| 21 | CD2 | CD2 molecule [Source:HGNC Symbol;Acc:HGNC:1639] | 249 | 1.381 | 0.2962 | Yes |
| 22 | GBP2 | guanylate binding protein 2 [Source:HGNC Symbol;Acc:HGNC:4183] | 250 | 1.381 | 0.3075 | Yes |
| 23 | TLR3 | toll like receptor 3 [Source:HGNC Symbol;Acc:HGNC:11849] | 259 | 1.367 | 0.3182 | Yes |
| 24 | LCK | "LCK proto-oncogene, Src family tyrosine kinase [Source:HGNC Symbol;Acc:HGNC:6524]" | 286 | 1.331 | 0.3274 | Yes |
| 25 | IL1B | interleukin 1 beta [Source:HGNC Symbol;Acc:HGNC:5992] | 303 | 1.298 | 0.3369 | Yes |
| 26 | PSMB10 | proteasome 20S subunit beta 10 [Source:HGNC Symbol;Acc:HGNC:9538] | 326 | 1.261 | 0.3458 | Yes |
| 27 | IRF7 | interferon regulatory factor 7 [Source:HGNC Symbol;Acc:HGNC:6122] | 359 | 1.219 | 0.3537 | Yes |
| 28 | CD7 | CD7 molecule [Source:HGNC Symbol;Acc:HGNC:1695] | 374 | 1.204 | 0.3627 | Yes |
| 29 | IL6 | interleukin 6 [Source:HGNC Symbol;Acc:HGNC:6018] | 378 | 1.200 | 0.3723 | Yes |
| 30 | FAS | Fas cell surface death receptor [Source:HGNC Symbol;Acc:HGNC:11920] | 418 | 1.154 | 0.3792 | Yes |
| 31 | IL2RG | interleukin 2 receptor subunit gamma [Source:HGNC Symbol;Acc:HGNC:6010] | 432 | 1.141 | 0.3877 | Yes |
| 32 | B2M | beta-2-microglobulin [Source:HGNC Symbol;Acc:HGNC:914] | 441 | 1.131 | 0.3964 | Yes |
| 33 | CXCL9 | C-X-C motif chemokine ligand 9 [Source:HGNC Symbol;Acc:HGNC:7098] | 473 | 1.098 | 0.4034 | Yes |
| 34 | CD86 | CD86 molecule [Source:HGNC Symbol;Acc:HGNC:1705] | 518 | 1.048 | 0.4091 | Yes |
| 35 | TLR6 | toll like receptor 6 [Source:HGNC Symbol;Acc:HGNC:16711] | 542 | 1.032 | 0.4161 | Yes |
| 36 | FYB1 | FYN binding protein 1 [Source:HGNC Symbol;Acc:HGNC:4036] | 563 | 1.019 | 0.4231 | Yes |
| 37 | LTB | lymphotoxin beta [Source:HGNC Symbol;Acc:HGNC:6711] | 593 | 1.000 | 0.4294 | Yes |
| 38 | CD80 | CD80 molecule [Source:HGNC Symbol;Acc:HGNC:1700] | 608 | 0.992 | 0.4366 | Yes |
| 39 | CD96 | CD96 molecule [Source:HGNC Symbol;Acc:HGNC:16892] | 620 | 0.979 | 0.4439 | Yes |
| 40 | CD28 | CD28 molecule [Source:HGNC Symbol;Acc:HGNC:1653] | 634 | 0.969 | 0.4510 | Yes |
| 41 | IL12RB1 | interleukin 12 receptor subunit beta 1 [Source:HGNC Symbol;Acc:HGNC:5971] | 705 | 0.928 | 0.4541 | Yes |
| 42 | CXCL13 | C-X-C motif chemokine ligand 13 [Source:HGNC Symbol;Acc:HGNC:10639] | 710 | 0.927 | 0.4614 | Yes |
| 43 | LCP2 | lymphocyte cytosolic protein 2 [Source:HGNC Symbol;Acc:HGNC:6529] | 727 | 0.918 | 0.4678 | Yes |
| 44 | PTPRC | protein tyrosine phosphatase receptor type C [Source:HGNC Symbol;Acc:HGNC:9666] | 756 | 0.894 | 0.4733 | Yes |
| 45 | SIT1 | signaling threshold regulating transmembrane adaptor 1 [Source:HGNC Symbol;Acc:HGNC:17710] | 786 | 0.886 | 0.4787 | Yes |
| 46 | CCR1 | C-C motif chemokine receptor 1 [Source:HGNC Symbol;Acc:HGNC:1602] | 866 | 0.853 | 0.4806 | Yes |
| 47 | TLR1 | toll like receptor 1 [Source:HGNC Symbol;Acc:HGNC:11847] | 873 | 0.850 | 0.4871 | Yes |
| 48 | NLRP3 | NLR family pyrin domain containing 3 [Source:HGNC Symbol;Acc:HGNC:16400] | 899 | 0.841 | 0.4924 | Yes |
| 49 | IFNG | interferon gamma [Source:HGNC Symbol;Acc:HGNC:5438] | 906 | 0.840 | 0.4989 | Yes |
| 50 | CD40LG | CD40 ligand [Source:HGNC Symbol;Acc:HGNC:11935] | 918 | 0.839 | 0.5050 | Yes |
| 51 | CCR5 | C-C motif chemokine receptor 5 [Source:HGNC Symbol;Acc:HGNC:1606] | 1014 | 0.806 | 0.5055 | Yes |
| 52 | IGSF6 | immunoglobulin superfamily member 6 [Source:HGNC Symbol;Acc:HGNC:5953] | 1027 | 0.801 | 0.5112 | Yes |
| 53 | PTPN6 | protein tyrosine phosphatase non-receptor type 6 [Source:HGNC Symbol;Acc:HGNC:9658] | 1049 | 0.790 | 0.5163 | Yes |
| 54 | CD247 | CD247 molecule [Source:HGNC Symbol;Acc:HGNC:1677] | 1050 | 0.789 | 0.5228 | Yes |
| 55 | TAP2 | "transporter 2, ATP binding cassette subfamily B member [Source:HGNC Symbol;Acc:HGNC:44]" | 1094 | 0.772 | 0.5263 | Yes |
| 56 | ICAM1 | intercellular adhesion molecule 1 [Source:HGNC Symbol;Acc:HGNC:5344] | 1112 | 0.767 | 0.5315 | Yes |
| 57 | GZMA | granzyme A [Source:HGNC Symbol;Acc:HGNC:4708] | 1113 | 0.766 | 0.5378 | Yes |
| 58 | HLA-A | "major histocompatibility complex, class I, A [Source:HGNC Symbol;Acc:HGNC:4931]" | 1127 | 0.761 | 0.5431 | Yes |
| 59 | CTSS | cathepsin S [Source:HGNC Symbol;Acc:HGNC:2545] | 1201 | 0.731 | 0.5444 | Yes |
| 60 | HLA-DMA | "major histocompatibility complex, class II, DM alpha [Source:HGNC Symbol;Acc:HGNC:4934]" | 1238 | 0.719 | 0.5480 | Yes |
| 61 | TRAT1 | T cell receptor associated transmembrane adaptor 1 [Source:HGNC Symbol;Acc:HGNC:30698] | 1250 | 0.713 | 0.5531 | Yes |
| 62 | CD40 | CD40 molecule [Source:HGNC Symbol;Acc:HGNC:11919] | 1287 | 0.700 | 0.5565 | Yes |
| 63 | MAP4K1 | mitogen-activated protein kinase kinase kinase kinase 1 [Source:HGNC Symbol;Acc:HGNC:6863] | 1305 | 0.695 | 0.5610 | Yes |
| 64 | PRKCG | protein kinase C gamma [Source:HGNC Symbol;Acc:HGNC:9402] | 1319 | 0.687 | 0.5658 | Yes |
| 65 | SRGN | serglycin [Source:HGNC Symbol;Acc:HGNC:9361] | 1322 | 0.687 | 0.5713 | Yes |
| 66 | SOCS1 | suppressor of cytokine signaling 1 [Source:HGNC Symbol;Acc:HGNC:19383] | 1359 | 0.674 | 0.5745 | Yes |
| 67 | IL18RAP | interleukin 18 receptor accessory protein [Source:HGNC Symbol;Acc:HGNC:5989] | 1369 | 0.670 | 0.5794 | Yes |
| 68 | IRF8 | interferon regulatory factor 8 [Source:HGNC Symbol;Acc:HGNC:5358] | 1378 | 0.667 | 0.5843 | Yes |
| 69 | TAPBP | TAP binding protein [Source:HGNC Symbol;Acc:HGNC:11566] | 1384 | 0.665 | 0.5894 | Yes |
| 70 | CFP | complement factor properdin [Source:HGNC Symbol;Acc:HGNC:8864] | 1425 | 0.652 | 0.5922 | Yes |
| 71 | ICOSLG | inducible T cell costimulator ligand [Source:HGNC Symbol;Acc:HGNC:17087] | 1433 | 0.651 | 0.5971 | Yes |
| 72 | HLA-DQA1 | "major histocompatibility complex, class II, DQ alpha 1 [Source:HGNC Symbol;Acc:HGNC:4942]" | 1442 | 0.645 | 0.6018 | Yes |
| 73 | CCL11 | C-C motif chemokine ligand 11 [Source:HGNC Symbol;Acc:HGNC:10610] | 1443 | 0.643 | 0.6071 | Yes |
| 74 | HCLS1 | hematopoietic cell-specific Lyn substrate 1 [Source:HGNC Symbol;Acc:HGNC:4844] | 1484 | 0.633 | 0.6097 | Yes |
| 75 | CCR2 | C-C motif chemokine receptor 2 [Source:HGNC Symbol;Acc:HGNC:1603] | 1516 | 0.621 | 0.6127 | Yes |
| 76 | WAS | WASP actin nucleation promoting factor [Source:HGNC Symbol;Acc:HGNC:12731] | 1584 | 0.599 | 0.6133 | Yes |
| 77 | CD4 | CD4 molecule [Source:HGNC Symbol;Acc:HGNC:1678] | 1591 | 0.596 | 0.6178 | Yes |
| 78 | IL18 | interleukin 18 [Source:HGNC Symbol;Acc:HGNC:5986] | 1622 | 0.588 | 0.6206 | Yes |
| 79 | ITGB2 | integrin subunit beta 2 [Source:HGNC Symbol;Acc:HGNC:6155] | 1632 | 0.585 | 0.6248 | Yes |
| 80 | GPR65 | G protein-coupled receptor 65 [Source:HGNC Symbol;Acc:HGNC:4517] | 1679 | 0.574 | 0.6266 | Yes |
| 81 | CD74 | CD74 molecule [Source:HGNC Symbol;Acc:HGNC:1697] | 1682 | 0.573 | 0.6311 | Yes |
| 82 | HLA-DOB | "major histocompatibility complex, class II, DO beta [Source:HGNC Symbol;Acc:HGNC:4937]" | 1709 | 0.565 | 0.6341 | Yes |
| 83 | CRTAM | cytotoxic and regulatory T cell molecule [Source:HGNC Symbol;Acc:HGNC:24313] | 1743 | 0.557 | 0.6365 | Yes |
| 84 | ITGAL | integrin subunit alpha L [Source:HGNC Symbol;Acc:HGNC:6148] | 1749 | 0.556 | 0.6407 | Yes |
| 85 | CDKN2A | cyclin dependent kinase inhibitor 2A [Source:HGNC Symbol;Acc:HGNC:1787] | 1779 | 0.550 | 0.6433 | Yes |
| 86 | LY86 | lymphocyte antigen 86 [Source:HGNC Symbol;Acc:HGNC:16837] | 1784 | 0.548 | 0.6475 | Yes |
| 87 | NOS2 | nitric oxide synthase 2 [Source:HGNC Symbol;Acc:HGNC:7873] | 1838 | 0.536 | 0.6485 | Yes |
| 88 | TNF | tumor necrosis factor [Source:HGNC Symbol;Acc:HGNC:11892] | 1880 | 0.528 | 0.6502 | Yes |
| 89 | IL27RA | interleukin 27 receptor subunit alpha [Source:HGNC Symbol;Acc:HGNC:17290] | 1887 | 0.527 | 0.6541 | Yes |
| 90 | CXCR3 | C-X-C motif chemokine receptor 3 [Source:HGNC Symbol;Acc:HGNC:4540] | 1918 | 0.519 | 0.6564 | Yes |
| 91 | BCAT1 | branched chain amino acid transaminase 1 [Source:HGNC Symbol;Acc:HGNC:976] | 1979 | 0.507 | 0.6567 | Yes |
| 92 | LYN | "LYN proto-oncogene, Src family tyrosine kinase [Source:HGNC Symbol;Acc:HGNC:6735]" | 2037 | 0.501 | 0.6571 | Yes |
| 93 | HLA-DMB | "major histocompatibility complex, class II, DM beta [Source:HGNC Symbol;Acc:HGNC:4935]" | 2045 | 0.500 | 0.6607 | Yes |
| 94 | IFNAR2 | interferon alpha and beta receptor subunit 2 [Source:HGNC Symbol;Acc:HGNC:5433] | 2095 | 0.493 | 0.6616 | Yes |
| 95 | IL12B | interleukin 12B [Source:HGNC Symbol;Acc:HGNC:5970] | 2102 | 0.491 | 0.6652 | Yes |
| 96 | FCGR2B | Fc fragment of IgG receptor IIb [Source:HGNC Symbol;Acc:HGNC:3618] | 2339 | 0.462 | 0.6537 | No |
| 97 | CD47 | CD47 molecule [Source:HGNC Symbol;Acc:HGNC:1682] | 2439 | 0.445 | 0.6510 | No |
| 98 | CCL19 | C-C motif chemokine ligand 19 [Source:HGNC Symbol;Acc:HGNC:10617] | 2489 | 0.445 | 0.6514 | No |
| 99 | RPL3L | ribosomal protein L3 like [Source:HGNC Symbol;Acc:HGNC:10351] | 2610 | 0.433 | 0.6472 | No |
| 100 | IL15 | interleukin 15 [Source:HGNC Symbol;Acc:HGNC:5977] | 2791 | 0.418 | 0.6390 | No |
| 101 | CCL7 | C-C motif chemokine ligand 7 [Source:HGNC Symbol;Acc:HGNC:10634] | 2997 | 0.404 | 0.6291 | No |
| 102 | MMP9 | matrix metallopeptidase 9 [Source:HGNC Symbol;Acc:HGNC:7176] | 3018 | 0.401 | 0.6311 | No |
| 103 | IL4R | interleukin 4 receptor [Source:HGNC Symbol;Acc:HGNC:6015] | 3233 | 0.368 | 0.6203 | No |
| 104 | INHBA | inhibin subunit beta A [Source:HGNC Symbol;Acc:HGNC:6066] | 3323 | 0.354 | 0.6174 | No |
| 105 | IFNGR2 | interferon gamma receptor 2 [Source:HGNC Symbol;Acc:HGNC:5440] | 3530 | 0.323 | 0.6067 | No |
| 106 | ELF4 | E74 like ETS transcription factor 4 [Source:HGNC Symbol;Acc:HGNC:3319] | 3534 | 0.323 | 0.6092 | No |
| 107 | CCL2 | C-C motif chemokine ligand 2 [Source:HGNC Symbol;Acc:HGNC:10618] | 3622 | 0.312 | 0.6061 | No |
| 108 | JAK2 | Janus kinase 2 [Source:HGNC Symbol;Acc:HGNC:6192] | 3644 | 0.308 | 0.6073 | No |
| 109 | HDAC9 | histone deacetylase 9 [Source:HGNC Symbol;Acc:HGNC:14065] | 3665 | 0.306 | 0.6085 | No |
| 110 | GALNT1 | polypeptide N-acetylgalactosaminyltransferase 1 [Source:HGNC Symbol;Acc:HGNC:4123] | 3751 | 0.296 | 0.6054 | No |
| 111 | PRF1 | perforin 1 [Source:HGNC Symbol;Acc:HGNC:9360] | 3794 | 0.292 | 0.6051 | No |
| 112 | SPI1 | Spi-1 proto-oncogene [Source:HGNC Symbol;Acc:HGNC:11241] | 3979 | 0.268 | 0.5954 | No |
| 113 | BRCA1 | BRCA1 DNA repair associated [Source:HGNC Symbol;Acc:HGNC:1100] | 4003 | 0.265 | 0.5960 | No |
| 114 | IL7 | interleukin 7 [Source:HGNC Symbol;Acc:HGNC:6023] | 4006 | 0.265 | 0.5981 | No |
| 115 | NCF4 | neutrophil cytosolic factor 4 [Source:HGNC Symbol;Acc:HGNC:7662] | 4090 | 0.255 | 0.5948 | No |
| 116 | CD79A | CD79a molecule [Source:HGNC Symbol;Acc:HGNC:1698] | 4119 | 0.252 | 0.5951 | No |
| 117 | HLA-DOA | "major histocompatibility complex, class II, DO alpha [Source:HGNC Symbol;Acc:HGNC:4936]" | 4168 | 0.247 | 0.5940 | No |
| 118 | HIF1A | hypoxia inducible factor 1 subunit alpha [Source:HGNC Symbol;Acc:HGNC:4910] | 4178 | 0.246 | 0.5954 | No |
| 119 | MTIF2 | mitochondrial translational initiation factor 2 [Source:HGNC Symbol;Acc:HGNC:7441] | 4179 | 0.246 | 0.5974 | No |
| 120 | TPD52 | tumor protein D52 [Source:HGNC Symbol;Acc:HGNC:12005] | 4259 | 0.236 | 0.5942 | No |
| 121 | BCL3 | BCL3 transcription coactivator [Source:HGNC Symbol;Acc:HGNC:998] | 4268 | 0.235 | 0.5956 | No |
| 122 | EREG | epiregulin [Source:HGNC Symbol;Acc:HGNC:3443] | 4321 | 0.231 | 0.5942 | No |
| 123 | ST8SIA4 | "ST8 alpha-N-acetyl-neuraminide alpha-2,8-sialyltransferase 4 [Source:HGNC Symbol;Acc:HGNC:10871]" | 4356 | 0.227 | 0.5938 | No |
| 124 | ABI1 | abl interactor 1 [Source:HGNC Symbol;Acc:HGNC:11320] | 4361 | 0.226 | 0.5954 | No |
| 125 | FGR | "FGR proto-oncogene, Src family tyrosine kinase [Source:HGNC Symbol;Acc:HGNC:3697]" | 4489 | 0.214 | 0.5890 | No |
| 126 | PRKCB | protein kinase C beta [Source:HGNC Symbol;Acc:HGNC:9395] | 4572 | 0.207 | 0.5854 | No |
| 127 | ACVR2A | activin A receptor type 2A [Source:HGNC Symbol;Acc:HGNC:173] | 4777 | 0.190 | 0.5737 | No |
| 128 | PF4 | platelet factor 4 [Source:HGNC Symbol;Acc:HGNC:8861] | 4836 | 0.184 | 0.5715 | No |
| 129 | TIMP1 | TIMP metallopeptidase inhibitor 1 [Source:HGNC Symbol;Acc:HGNC:11820] | 4971 | 0.171 | 0.5642 | No |
| 130 | GLMN | "glomulin, FKBP associated protein [Source:HGNC Symbol;Acc:HGNC:14373]" | 5034 | 0.165 | 0.5616 | No |
| 131 | IL16 | interleukin 16 [Source:HGNC Symbol;Acc:HGNC:5980] | 5170 | 0.153 | 0.5541 | No |
| 132 | AARS1 | alanyl-tRNA synthetase 1 [Source:HGNC Symbol;Acc:HGNC:20] | 5466 | 0.130 | 0.5361 | No |
| 133 | RIPK2 | receptor interacting serine/threonine kinase 2 [Source:HGNC Symbol;Acc:HGNC:10020] | 5692 | 0.113 | 0.5225 | No |
| 134 | RARS1 | arginyl-tRNA synthetase 1 [Source:HGNC Symbol;Acc:HGNC:9870] | 5716 | 0.111 | 0.5219 | No |
| 135 | IL12A | interleukin 12A [Source:HGNC Symbol;Acc:HGNC:5969] | 5766 | 0.108 | 0.5196 | No |
| 136 | TGFB1 | transforming growth factor beta 1 [Source:HGNC Symbol;Acc:HGNC:11766] | 6038 | 0.088 | 0.5028 | No |
| 137 | DARS1 | aspartyl-tRNA synthetase 1 [Source:HGNC Symbol;Acc:HGNC:2678] | 6269 | 0.072 | 0.4886 | No |
| 138 | WARS1 | tryptophanyl-tRNA synthetase 1 [Source:HGNC Symbol;Acc:HGNC:12729] | 6325 | 0.068 | 0.4856 | No |
| 139 | CSK | C-terminal Src kinase [Source:HGNC Symbol;Acc:HGNC:2444] | 6350 | 0.066 | 0.4846 | No |
| 140 | THY1 | Thy-1 cell surface antigen [Source:HGNC Symbol;Acc:HGNC:11801] | 6495 | 0.055 | 0.4757 | No |
| 141 | F2 | "coagulation factor II, thrombin [Source:HGNC Symbol;Acc:HGNC:3535]" | 6945 | 0.026 | 0.4469 | No |
| 142 | CARTPT | CART prepropeptide [Source:HGNC Symbol;Acc:HGNC:24323] | 6954 | 0.026 | 0.4466 | No |
| 143 | DEGS1 | "delta 4-desaturase, sphingolipid 1 [Source:HGNC Symbol;Acc:HGNC:13709]" | 7049 | 0.021 | 0.4407 | No |
| 144 | MRPL3 | mitochondrial ribosomal protein L3 [Source:HGNC Symbol;Acc:HGNC:10379] | 7053 | 0.021 | 0.4407 | No |
| 145 | FLNA | filamin A [Source:HGNC Symbol;Acc:HGNC:3754] | 7185 | 0.013 | 0.4323 | No |
| 146 | STAB1 | stabilin 1 [Source:HGNC Symbol;Acc:HGNC:18628] | 7202 | 0.012 | 0.4314 | No |
| 147 | CD1D | CD1d molecule [Source:HGNC Symbol;Acc:HGNC:1637] | 7204 | 0.012 | 0.4314 | No |
| 148 | LIF | LIF interleukin 6 family cytokine [Source:HGNC Symbol;Acc:HGNC:6596] | 7310 | 0.006 | 0.4247 | No |
| 149 | IKBKB | inhibitor of nuclear factor kappa B kinase subunit beta [Source:HGNC Symbol;Acc:HGNC:5960] | 7325 | 0.005 | 0.4238 | No |
| 150 | NCK1 | NCK adaptor protein 1 [Source:HGNC Symbol;Acc:HGNC:7664] | 7446 | -0.001 | 0.4161 | No |
| 151 | IFNGR1 | interferon gamma receptor 1 [Source:HGNC Symbol;Acc:HGNC:5439] | 7599 | -0.009 | 0.4063 | No |
| 152 | EIF3J | eukaryotic translation initiation factor 3 subunit J [Source:HGNC Symbol;Acc:HGNC:3270] | 7794 | -0.018 | 0.3940 | No |
| 153 | ETS1 | "ETS proto-oncogene 1, transcription factor [Source:HGNC Symbol;Acc:HGNC:3488]" | 7841 | -0.021 | 0.3912 | No |
| 154 | CAPG | "capping actin protein, gelsolin like [Source:HGNC Symbol;Acc:HGNC:1474]" | 8049 | -0.031 | 0.3780 | No |
| 155 | CCND2 | cyclin D2 [Source:HGNC Symbol;Acc:HGNC:1583] | 8119 | -0.036 | 0.3739 | No |
| 156 | UBE2N | ubiquitin conjugating enzyme E2 N [Source:HGNC Symbol;Acc:HGNC:12492] | 9173 | -0.092 | 0.3066 | No |
| 157 | GCNT1 | glucosaminyl (N-acetyl) transferase 1 [Source:HGNC Symbol;Acc:HGNC:4203] | 9214 | -0.094 | 0.3048 | No |
| 158 | TRAF2 | TNF receptor associated factor 2 [Source:HGNC Symbol;Acc:HGNC:12032] | 9218 | -0.094 | 0.3053 | No |
| 159 | NME1 | NME/NM23 nucleoside diphosphate kinase 1 [Source:HGNC Symbol;Acc:HGNC:7849] | 9314 | -0.099 | 0.3000 | No |
| 160 | TLR2 | toll like receptor 2 [Source:HGNC Symbol;Acc:HGNC:11848] | 9404 | -0.105 | 0.2951 | No |
| 161 | EGFR | epidermal growth factor receptor [Source:HGNC Symbol;Acc:HGNC:3236] | 9513 | -0.111 | 0.2891 | No |
| 162 | EIF5A | eukaryotic translation initiation factor 5A [Source:HGNC Symbol;Acc:HGNC:3300] | 9741 | -0.127 | 0.2754 | No |
| 163 | ABCE1 | ATP binding cassette subfamily E member 1 [Source:HGNC Symbol;Acc:HGNC:69] | 10033 | -0.144 | 0.2578 | No |
| 164 | NCR1 | natural cytotoxicity triggering receptor 1 [Source:HGNC Symbol;Acc:HGNC:6731] | 10296 | -0.159 | 0.2422 | No |
| 165 | NPM1 | nucleophosmin 1 [Source:HGNC Symbol;Acc:HGNC:7910] | 10355 | -0.163 | 0.2398 | No |
| 166 | CCND3 | cyclin D3 [Source:HGNC Symbol;Acc:HGNC:1585] | 10446 | -0.168 | 0.2353 | No |
| 167 | SOCS5 | suppressor of cytokine signaling 5 [Source:HGNC Symbol;Acc:HGNC:16852] | 11218 | -0.212 | 0.1872 | No |
| 168 | MAP3K7 | mitogen-activated protein kinase kinase kinase 7 [Source:HGNC Symbol;Acc:HGNC:6859] | 11555 | -0.232 | 0.1674 | No |
| 169 | AKT1 | AKT serine/threonine kinase 1 [Source:HGNC Symbol;Acc:HGNC:391] | 11570 | -0.233 | 0.1684 | No |
| 170 | EIF3D | eukaryotic translation initiation factor 3 subunit D [Source:HGNC Symbol;Acc:HGNC:3278] | 11922 | -0.255 | 0.1478 | No |
| 171 | UBE2D1 | ubiquitin conjugating enzyme E2 D1 [Source:HGNC Symbol;Acc:HGNC:12474] | 12897 | -0.327 | 0.0876 | No |
| 172 | BCL10 | BCL10 immune signaling adaptor [Source:HGNC Symbol;Acc:HGNC:989] | 13120 | -0.349 | 0.0761 | No |
| 173 | EIF4G3 | eukaryotic translation initiation factor 4 gamma 3 [Source:HGNC Symbol;Acc:HGNC:3298] | 13236 | -0.359 | 0.0716 | No |
| 174 | EIF3A | eukaryotic translation initiation factor 3 subunit A [Source:HGNC Symbol;Acc:HGNC:3271] | 13320 | -0.369 | 0.0693 | No |
| 175 | GZMB | granzyme B [Source:HGNC Symbol;Acc:HGNC:4709] | 13371 | -0.376 | 0.0691 | No |
| 176 | IL4 | interleukin 4 [Source:HGNC Symbol;Acc:HGNC:6014] | 13785 | -0.416 | 0.0458 | No |
| 177 | KRT1 | keratin 1 [Source:HGNC Symbol;Acc:HGNC:6412] | 13940 | -0.428 | 0.0394 | No |
| 178 | TGFB2 | transforming growth factor beta 2 [Source:HGNC Symbol;Acc:HGNC:11768] | 14145 | -0.457 | 0.0299 | No |
| 179 | IL2 | interleukin 2 [Source:HGNC Symbol;Acc:HGNC:6001] | 14273 | -0.476 | 0.0256 | No |
| 180 | RPL9 | ribosomal protein L9 [Source:HGNC Symbol;Acc:HGNC:10369] | 14378 | -0.490 | 0.0229 | No |
| 181 | APBB1 | amyloid beta precursor protein binding family B member 1 [Source:HGNC Symbol;Acc:HGNC:581] | 14512 | -0.515 | 0.0185 | No |
| 182 | IL11 | interleukin 11 [Source:HGNC Symbol;Acc:HGNC:5966] | 14584 | -0.529 | 0.0183 | No |
| 183 | RPL39 | ribosomal protein L39 [Source:HGNC Symbol;Acc:HGNC:10350] | 14615 | -0.536 | 0.0207 | No |
| 184 | RPS3A | ribosomal protein S3A [Source:HGNC Symbol;Acc:HGNC:10421] | 14665 | -0.547 | 0.0220 | No |
| 185 | RPS9 | ribosomal protein S9 [Source:HGNC Symbol;Acc:HGNC:10442] | 14697 | -0.553 | 0.0245 | No |
| 186 | DYRK3 | dual specificity tyrosine phosphorylation regulated kinase 3 [Source:HGNC Symbol;Acc:HGNC:3094] | 14703 | -0.554 | 0.0287 | No |
| 187 | CSF1 | colony stimulating factor 1 [Source:HGNC Symbol;Acc:HGNC:2432] | 14746 | -0.566 | 0.0307 | No |
| 188 | ACHE | acetylcholinesterase (Cartwright blood group) [Source:HGNC Symbol;Acc:HGNC:108] | 14808 | -0.585 | 0.0315 | No |
| 189 | RPS19 | ribosomal protein S19 [Source:HGNC Symbol;Acc:HGNC:10402] | 15054 | -0.693 | 0.0213 | No |
| 190 | F2R | coagulation factor II thrombin receptor [Source:HGNC Symbol;Acc:HGNC:3537] | 15375 | -0.870 | 0.0078 | No |
| 191 | INHBB | inhibin subunit beta B [Source:HGNC Symbol;Acc:HGNC:6067] | 15606 | -1.356 | 0.0040 | No |
Table: GSEA details [plain text format]

  

Fig 2: HALLMARK\_ALLOGRAFT\_REJECTION      
 Blue-Pink O' Gram in the Space of the Analyzed GeneSet

  

Fig 3: HALLMARK\_ALLOGRAFT\_REJECTION: Random ES distribution      
 Gene set null distribution of ES for **HALLMARK\_ALLOGRAFT\_REJECTION**
