# Supplementary material for: In vivo CRISPR screens reveal Serpinb9 and Adam2 as regulators of immune therapy response in lung cancer
Source: Nat Commun. 2023 May 31;14:3150. doi: 10.1038/s41467-023-38841-7 (PMC10232477; doi:10.1038/s41467-023-38841-7)
Supplement: Supplementary file 13 — Source Data [file 41467_2023_38841_MOESM13_ESM.zip › Figure 4E.zip!/my_analysis.Gsea.1640146340255_Hallmark/HALLMARK_COAGULATION.html]

Details for gene set HALLMARK\_COAGULATION[GSEA]

|  || Dataset | December 21, 2021\_for GSEA run\_collapsed\_to\_symbols.GSEA\_B6\_NI.cls #CTLR\_NI\_versus\_ADAM2\_NI |
| Phenotype | GSEA\_B6\_NI.cls#CTLR\_NI\_versus\_ADAM2\_NI |
| Upregulated in class | CTLR\_NI |
| GeneSet | HALLMARK\_COAGULATION |
| Enrichment Score (ES) | 0.37572253 |
| Normalized Enrichment Score (NES) | 1.3451984 |
| Nominal p-value | 0.0 |
| FDR q-value | 0.31006795 |
| FWER p-Value | 0.844 |
Table: GSEA Results Summary

  

Fig 1: Enrichment plot: HALLMARK\_COAGULATION      
 Profile of the Running ES Score & Positions of GeneSet Members on the Rank Ordered List

  

| SYMBOL | TITLE | RANK IN GENE LIST | RANK METRIC SCORE | RUNNING ES | CORE ENRICHMENT || 1 | MMP10 | matrix metallopeptidase 10 [Source:HGNC Symbol;Acc:HGNC:7156] | 48 | 2.057 | 0.0351 | Yes |
| 2 | C3 | complement C3 [Source:HGNC Symbol;Acc:HGNC:1318] | 66 | 1.929 | 0.0699 | Yes |
| 3 | C2 | complement C2 [Source:HGNC Symbol;Acc:HGNC:1248] | 123 | 1.731 | 0.0985 | Yes |
| 4 | MMP3 | matrix metallopeptidase 3 [Source:HGNC Symbol;Acc:HGNC:7173] | 223 | 1.434 | 0.1188 | Yes |
| 5 | CFB | complement factor B [Source:HGNC Symbol;Acc:HGNC:1037] | 245 | 1.387 | 0.1432 | Yes |
| 6 | DPP4 | dipeptidyl peptidase 4 [Source:HGNC Symbol;Acc:HGNC:3009] | 349 | 1.234 | 0.1595 | Yes |
| 7 | MMP1 | matrix metallopeptidase 1 [Source:HGNC Symbol;Acc:HGNC:7155] | 528 | 1.037 | 0.1673 | Yes |
| 8 | PLAU | "plasminogen activator, urokinase [Source:HGNC Symbol;Acc:HGNC:9052]" | 537 | 1.033 | 0.1860 | Yes |
| 9 | CTSE | cathepsin E [Source:HGNC Symbol;Acc:HGNC:2530] | 590 | 1.003 | 0.2013 | Yes |
| 10 | HPN | hepsin [Source:HGNC Symbol;Acc:HGNC:5155] | 625 | 0.973 | 0.2172 | Yes |
| 11 | PROZ | "protein Z, vitamin K dependent plasma glycoprotein [Source:HGNC Symbol;Acc:HGNC:9460]" | 662 | 0.956 | 0.2327 | Yes |
| 12 | TMPRSS6 | transmembrane serine protease 6 [Source:HGNC Symbol;Acc:HGNC:16517] | 668 | 0.953 | 0.2500 | Yes |
| 13 | F9 | coagulation factor IX [Source:HGNC Symbol;Acc:HGNC:3551] | 697 | 0.936 | 0.2656 | Yes |
| 14 | TIMP3 | TIMP metallopeptidase inhibitor 3 [Source:HGNC Symbol;Acc:HGNC:11822] | 707 | 0.927 | 0.2823 | Yes |
| 15 | LEFTY2 | left-right determination factor 2 [Source:HGNC Symbol;Acc:HGNC:3122] | 893 | 0.845 | 0.2861 | Yes |
| 16 | SERPINB2 | serpin family B member 2 [Source:HGNC Symbol;Acc:HGNC:8584] | 1010 | 0.810 | 0.2937 | Yes |
| 17 | TFPI2 | tissue factor pathway inhibitor 2 [Source:HGNC Symbol;Acc:HGNC:11761] | 1096 | 0.771 | 0.3026 | Yes |
| 18 | ITGA2 | integrin subunit alpha 2 [Source:HGNC Symbol;Acc:HGNC:6137] | 1109 | 0.769 | 0.3161 | Yes |
| 19 | CTSH | cathepsin H [Source:HGNC Symbol;Acc:HGNC:2535] | 1156 | 0.747 | 0.3270 | Yes |
| 20 | SERPING1 | serpin family G member 1 [Source:HGNC Symbol;Acc:HGNC:1228] | 1209 | 0.728 | 0.3372 | Yes |
| 21 | F10 | coagulation factor X [Source:HGNC Symbol;Acc:HGNC:3528] | 1225 | 0.723 | 0.3497 | Yes |
| 22 | GDA | guanine deaminase [Source:HGNC Symbol;Acc:HGNC:4212] | 1363 | 0.672 | 0.3534 | Yes |
| 23 | A2M | alpha-2-macroglobulin [Source:HGNC Symbol;Acc:HGNC:7] | 1612 | 0.591 | 0.3484 | Yes |
| 24 | GP9 | glycoprotein IX platelet [Source:HGNC Symbol;Acc:HGNC:4444] | 1829 | 0.537 | 0.3445 | Yes |
| 25 | CFD | complement factor D [Source:HGNC Symbol;Acc:HGNC:2771] | 1997 | 0.504 | 0.3431 | Yes |
| 26 | MEP1A | meprin A subunit alpha [Source:HGNC Symbol;Acc:HGNC:7015] | 2019 | 0.503 | 0.3511 | Yes |
| 27 | PLEK | pleckstrin [Source:HGNC Symbol;Acc:HGNC:9070] | 2033 | 0.502 | 0.3596 | Yes |
| 28 | C1R | complement C1r [Source:HGNC Symbol;Acc:HGNC:1246] | 2046 | 0.499 | 0.3681 | Yes |
| 29 | MMP2 | matrix metallopeptidase 2 [Source:HGNC Symbol;Acc:HGNC:7166] | 2247 | 0.477 | 0.3641 | Yes |
| 30 | FYN | "FYN proto-oncogene, Src family tyrosine kinase [Source:HGNC Symbol;Acc:HGNC:4037]" | 2288 | 0.469 | 0.3703 | Yes |
| 31 | APOC1 | apolipoprotein C1 [Source:HGNC Symbol;Acc:HGNC:607] | 2413 | 0.449 | 0.3707 | Yes |
| 32 | ACOX2 | acyl-CoA oxidase 2 [Source:HGNC Symbol;Acc:HGNC:120] | 2464 | 0.445 | 0.3757 | Yes |
| 33 | C9 | complement C9 [Source:HGNC Symbol;Acc:HGNC:1358] | 2652 | 0.426 | 0.3716 | No |
| 34 | F11 | coagulation factor XI [Source:HGNC Symbol;Acc:HGNC:3529] | 2839 | 0.411 | 0.3673 | No |
| 35 | KLF7 | Kruppel like factor 7 [Source:HGNC Symbol;Acc:HGNC:6350] | 3002 | 0.404 | 0.3644 | No |
| 36 | MMP9 | matrix metallopeptidase 9 [Source:HGNC Symbol;Acc:HGNC:7176] | 3018 | 0.401 | 0.3709 | No |
| 37 | C1QA | complement C1q A chain [Source:HGNC Symbol;Acc:HGNC:1241] | 3162 | 0.378 | 0.3687 | No |
| 38 | TF | transferrin [Source:HGNC Symbol;Acc:HGNC:11740] | 3227 | 0.369 | 0.3714 | No |
| 39 | THBS1 | thrombospondin 1 [Source:HGNC Symbol;Acc:HGNC:11785] | 3316 | 0.355 | 0.3724 | No |
| 40 | CTSB | cathepsin B [Source:HGNC Symbol;Acc:HGNC:2527] | 3373 | 0.346 | 0.3752 | No |
| 41 | C1S | complement C1s [Source:HGNC Symbol;Acc:HGNC:1247] | 3521 | 0.324 | 0.3718 | No |
| 42 | LGMN | legumain [Source:HGNC Symbol;Acc:HGNC:9472] | 4001 | 0.265 | 0.3459 | No |
| 43 | C8G | complement C8 gamma chain [Source:HGNC Symbol;Acc:HGNC:1354] | 4154 | 0.249 | 0.3408 | No |
| 44 | CSRP1 | cysteine and glycine rich protein 1 [Source:HGNC Symbol;Acc:HGNC:2469] | 4263 | 0.236 | 0.3382 | No |
| 45 | KLKB1 | kallikrein B1 [Source:HGNC Symbol;Acc:HGNC:6371] | 4362 | 0.226 | 0.3361 | No |
| 46 | ARF4 | ADP ribosylation factor 4 [Source:HGNC Symbol;Acc:HGNC:655] | 4393 | 0.223 | 0.3383 | No |
| 47 | WDR1 | WD repeat domain 1 [Source:HGNC Symbol;Acc:HGNC:12754] | 4463 | 0.217 | 0.3379 | No |
| 48 | OLR1 | oxidized low density lipoprotein receptor 1 [Source:HGNC Symbol;Acc:HGNC:8133] | 4464 | 0.217 | 0.3419 | No |
| 49 | FBN1 | fibrillin 1 [Source:HGNC Symbol;Acc:HGNC:3603] | 4479 | 0.215 | 0.3450 | No |
| 50 | PF4 | platelet factor 4 [Source:HGNC Symbol;Acc:HGNC:8861] | 4836 | 0.184 | 0.3255 | No |
| 51 | TIMP1 | TIMP metallopeptidase inhibitor 1 [Source:HGNC Symbol;Acc:HGNC:11820] | 4971 | 0.171 | 0.3201 | No |
| 52 | PROC | "protein C, inactivator of coagulation factors Va and VIIIa [Source:HGNC Symbol;Acc:HGNC:9451]" | 5251 | 0.146 | 0.3049 | No |
| 53 | F2RL2 | coagulation factor II thrombin receptor like 2 [Source:HGNC Symbol;Acc:HGNC:3539] | 5339 | 0.140 | 0.3019 | No |
| 54 | ITGB3 | integrin subunit beta 3 [Source:HGNC Symbol;Acc:HGNC:6156] | 5695 | 0.113 | 0.2811 | No |
| 55 | CASP9 | caspase 9 [Source:HGNC Symbol;Acc:HGNC:1511] | 5704 | 0.112 | 0.2827 | No |
| 56 | PROS1 | protein S [Source:HGNC Symbol;Acc:HGNC:9456] | 5860 | 0.102 | 0.2746 | No |
| 57 | GP1BA | glycoprotein Ib platelet subunit alpha [Source:HGNC Symbol;Acc:HGNC:4439] | 6050 | 0.087 | 0.2641 | No |
| 58 | ANG | angiogenin [Source:HGNC Symbol;Acc:HGNC:483] | 6415 | 0.061 | 0.2418 | No |
| 59 | CTSV | cathepsin V [Source:HGNC Symbol;Acc:HGNC:2538] | 6538 | 0.053 | 0.2350 | No |
| 60 | RABIF | RAB interacting factor [Source:HGNC Symbol;Acc:HGNC:9797] | 6601 | 0.048 | 0.2319 | No |
| 61 | MMP8 | matrix metallopeptidase 8 [Source:HGNC Symbol;Acc:HGNC:7175] | 6643 | 0.046 | 0.2301 | No |
| 62 | BMP1 | bone morphogenetic protein 1 [Source:HGNC Symbol;Acc:HGNC:1067] | 6929 | 0.027 | 0.2123 | No |
| 63 | F2 | "coagulation factor II, thrombin [Source:HGNC Symbol;Acc:HGNC:3535]" | 6945 | 0.026 | 0.2118 | No |
| 64 | APOA1 | apolipoprotein A1 [Source:HGNC Symbol;Acc:HGNC:600] | 7013 | 0.023 | 0.2079 | No |
| 65 | LAMP2 | lysosomal associated membrane protein 2 [Source:HGNC Symbol;Acc:HGNC:6501] | 7018 | 0.023 | 0.2081 | No |
| 66 | SH2B2 | SH2B adaptor protein 2 [Source:HGNC Symbol;Acc:HGNC:17381] | 7039 | 0.022 | 0.2072 | No |
| 67 | ITIH1 | inter-alpha-trypsin inhibitor heavy chain 1 [Source:HGNC Symbol;Acc:HGNC:6166] | 7544 | -0.006 | 0.1749 | No |
| 68 | CTSO | cathepsin O [Source:HGNC Symbol;Acc:HGNC:2542] | 7546 | -0.006 | 0.1749 | No |
| 69 | CFI | complement factor I [Source:HGNC Symbol;Acc:HGNC:5394] | 7729 | -0.015 | 0.1635 | No |
| 70 | MSRB2 | methionine sulfoxide reductase B2 [Source:HGNC Symbol;Acc:HGNC:17061] | 7834 | -0.021 | 0.1572 | No |
| 71 | LRP1 | LDL receptor related protein 1 [Source:HGNC Symbol;Acc:HGNC:6692] | 7894 | -0.024 | 0.1538 | No |
| 72 | RAPGEF3 | Rap guanine nucleotide exchange factor 3 [Source:HGNC Symbol;Acc:HGNC:16629] | 8218 | -0.041 | 0.1338 | No |
| 73 | MST1 | macrophage stimulating 1 [Source:HGNC Symbol;Acc:HGNC:7380] | 8232 | -0.042 | 0.1338 | No |
| 74 | ADAM9 | ADAM metallopeptidase domain 9 [Source:HGNC Symbol;Acc:HGNC:216] | 8719 | -0.068 | 0.1038 | No |
| 75 | CAPN2 | calpain 2 [Source:HGNC Symbol;Acc:HGNC:1479] | 8734 | -0.069 | 0.1042 | No |
| 76 | RAC1 | Rac family small GTPase 1 [Source:HGNC Symbol;Acc:HGNC:9801] | 9083 | -0.087 | 0.0834 | No |
| 77 | F8 | coagulation factor VIII [Source:HGNC Symbol;Acc:HGNC:3546] | 9135 | -0.090 | 0.0818 | No |
| 78 | PREP | prolyl endopeptidase [Source:HGNC Symbol;Acc:HGNC:9358] | 9307 | -0.099 | 0.0726 | No |
| 79 | ISCU | iron-sulfur cluster assembly enzyme [Source:HGNC Symbol;Acc:HGNC:29882] | 9379 | -0.103 | 0.0700 | No |
| 80 | MMP14 | matrix metallopeptidase 14 [Source:HGNC Symbol;Acc:HGNC:7160] | 9684 | -0.124 | 0.0527 | No |
| 81 | COMP | cartilage oligomeric matrix protein [Source:HGNC Symbol;Acc:HGNC:2227] | 9749 | -0.128 | 0.0510 | No |
| 82 | LTA4H | leukotriene A4 hydrolase [Source:HGNC Symbol;Acc:HGNC:6710] | 9826 | -0.132 | 0.0485 | No |
| 83 | SERPINE1 | serpin family E member 1 [Source:HGNC Symbol;Acc:HGNC:8583] | 10052 | -0.145 | 0.0367 | No |
| 84 | CTSK | cathepsin K [Source:HGNC Symbol;Acc:HGNC:2536] | 10091 | -0.147 | 0.0370 | No |
| 85 | DUSP14 | dual specificity phosphatase 14 [Source:HGNC Symbol;Acc:HGNC:17007] | 10146 | -0.151 | 0.0364 | No |
| 86 | F3 | "coagulation factor III, tissue factor [Source:HGNC Symbol;Acc:HGNC:3541]" | 10575 | -0.175 | 0.0121 | No |
| 87 | PDGFB | platelet derived growth factor subunit B [Source:HGNC Symbol;Acc:HGNC:8800] | 10685 | -0.182 | 0.0085 | No |
| 88 | GNG12 | G protein subunit gamma 12 [Source:HGNC Symbol;Acc:HGNC:19663] | 11209 | -0.212 | -0.0212 | No |
| 89 | FURIN | "furin, paired basic amino acid cleaving enzyme [Source:HGNC Symbol;Acc:HGNC:8568]" | 11210 | -0.212 | -0.0173 | No |
| 90 | DUSP6 | dual specificity phosphatase 6 [Source:HGNC Symbol;Acc:HGNC:3072] | 11560 | -0.232 | -0.0354 | No |
| 91 | CLU | clusterin [Source:HGNC Symbol;Acc:HGNC:2095] | 11910 | -0.254 | -0.0532 | No |
| 92 | USP11 | ubiquitin specific peptidase 11 [Source:HGNC Symbol;Acc:HGNC:12609] | 12400 | -0.289 | -0.0793 | No |
| 93 | PECAM1 | platelet and endothelial cell adhesion molecule 1 [Source:HGNC Symbol;Acc:HGNC:8823] | 12443 | -0.292 | -0.0765 | No |
| 94 | PEF1 | penta-EF-hand domain containing 1 [Source:HGNC Symbol;Acc:HGNC:30009] | 12563 | -0.301 | -0.0786 | No |
| 95 | MAFF | MAF bZIP transcription factor F [Source:HGNC Symbol;Acc:HGNC:6780] | 12675 | -0.309 | -0.0800 | No |
| 96 | ANXA1 | annexin A1 [Source:HGNC Symbol;Acc:HGNC:533] | 12834 | -0.322 | -0.0842 | No |
| 97 | CAPN5 | calpain 5 [Source:HGNC Symbol;Acc:HGNC:1482] | 12933 | -0.332 | -0.0843 | No |
| 98 | SIRT2 | sirtuin 2 [Source:HGNC Symbol;Acc:HGNC:10886] | 12993 | -0.337 | -0.0818 | No |
| 99 | P2RY1 | purinergic receptor P2Y1 [Source:HGNC Symbol;Acc:HGNC:8539] | 13283 | -0.365 | -0.0936 | No |
| 100 | CD9 | CD9 molecule [Source:HGNC Symbol;Acc:HGNC:1709] | 13472 | -0.388 | -0.0985 | No |
| 101 | HTRA1 | HtrA serine peptidase 1 [Source:HGNC Symbol;Acc:HGNC:9476] | 13525 | -0.394 | -0.0945 | No |
| 102 | GNB2 | G protein subunit beta 2 [Source:HGNC Symbol;Acc:HGNC:4398] | 13592 | -0.404 | -0.0912 | No |
| 103 | APOC2 | apolipoprotein C2 [Source:HGNC Symbol;Acc:HGNC:609] | 13689 | -0.415 | -0.0897 | No |
| 104 | SERPINA1 | serpin family A member 1 [Source:HGNC Symbol;Acc:HGNC:8941] | 13828 | -0.422 | -0.0907 | No |
| 105 | CPN1 | carboxypeptidase N subunit 1 [Source:HGNC Symbol;Acc:HGNC:2312] | 13910 | -0.428 | -0.0880 | No |
| 106 | FN1 | fibronectin 1 [Source:HGNC Symbol;Acc:HGNC:3778] | 14037 | -0.437 | -0.0880 | No |
| 107 | MASP2 | mannan binding lectin serine peptidase 2 [Source:HGNC Symbol;Acc:HGNC:6902] | 14111 | -0.451 | -0.0843 | No |
| 108 | VWF | von Willebrand factor [Source:HGNC Symbol;Acc:HGNC:12726] | 14117 | -0.452 | -0.0762 | No |
| 109 | SERPINC1 | serpin family C member 1 [Source:HGNC Symbol;Acc:HGNC:775] | 14208 | -0.466 | -0.0733 | No |
| 110 | S100A13 | S100 calcium binding protein A13 [Source:HGNC Symbol;Acc:HGNC:10490] | 14224 | -0.468 | -0.0656 | No |
| 111 | GSN | gelsolin [Source:HGNC Symbol;Acc:HGNC:4620] | 14267 | -0.475 | -0.0595 | No |
| 112 | SPARC | secreted protein acidic and cysteine rich [Source:HGNC Symbol;Acc:HGNC:11219] | 14281 | -0.478 | -0.0514 | No |
| 113 | PLAT | "plasminogen activator, tissue type [Source:HGNC Symbol;Acc:HGNC:9051]" | 14379 | -0.490 | -0.0486 | No |
| 114 | KLK8 | kallikrein related peptidase 8 [Source:HGNC Symbol;Acc:HGNC:6369] | 14414 | -0.493 | -0.0416 | No |
| 115 | CPQ | carboxypeptidase Q [Source:HGNC Symbol;Acc:HGNC:16910] | 14651 | -0.542 | -0.0467 | No |
| 116 | PRSS23 | serine protease 23 [Source:HGNC Symbol;Acc:HGNC:14370] | 14750 | -0.567 | -0.0424 | No |
| 117 | THBD | thrombomodulin [Source:HGNC Symbol;Acc:HGNC:11784] | 14835 | -0.594 | -0.0368 | No |
| 118 | MMP11 | matrix metallopeptidase 11 [Source:HGNC Symbol;Acc:HGNC:7157] | 14904 | -0.621 | -0.0296 | No |
| 119 | CRIP2 | cysteine rich protein 2 [Source:HGNC Symbol;Acc:HGNC:2361] | 15023 | -0.674 | -0.0247 | No |
| 120 | CFH | complement factor H [Source:HGNC Symbol;Acc:HGNC:4883] | 15171 | -0.760 | -0.0200 | No |
| 121 | S100A1 | S100 calcium binding protein A1 [Source:HGNC Symbol;Acc:HGNC:10486] | 15184 | -0.768 | -0.0065 | No |
| 122 | F12 | coagulation factor XII [Source:HGNC Symbol;Acc:HGNC:3530] | 15205 | -0.784 | 0.0068 | No |
| 123 | MMP15 | matrix metallopeptidase 15 [Source:HGNC Symbol;Acc:HGNC:7161] | 15583 | -1.234 | 0.0055 | No |
Table: GSEA details [plain text format]

  

Fig 2: HALLMARK\_COAGULATION      
 Blue-Pink O' Gram in the Space of the Analyzed GeneSet

  

Fig 3: HALLMARK\_COAGULATION: Random ES distribution      
 Gene set null distribution of ES for **HALLMARK\_COAGULATION**
