# Supplementary material for: In vivo CRISPR screens reveal Serpinb9 and Adam2 as regulators of immune therapy response in lung cancer
Source: Nat Commun. 2023 May 31;14:3150. doi: 10.1038/s41467-023-38841-7 (PMC10232477; doi:10.1038/s41467-023-38841-7)
Supplement: Supplementary file 13 — Source Data [file 41467_2023_38841_MOESM13_ESM.zip › Figure 4E.zip!/my_analysis.Gsea.1640146340255_Hallmark/HALLMARK_ESTROGEN_RESPONSE_LATE.html]

Details for gene set HALLMARK\_ESTROGEN\_RESPONSE\_LATE[GSEA]

|  || Dataset | December 21, 2021\_for GSEA run\_collapsed\_to\_symbols.GSEA\_B6\_NI.cls #CTLR\_NI\_versus\_ADAM2\_NI |
| Phenotype | GSEA\_B6\_NI.cls#CTLR\_NI\_versus\_ADAM2\_NI |
| Upregulated in class | CTLR\_NI |
| GeneSet | HALLMARK\_ESTROGEN\_RESPONSE\_LATE |
| Enrichment Score (ES) | 0.30859253 |
| Normalized Enrichment Score (NES) | 1.0718157 |
| Nominal p-value | 0.33778626 |
| FDR q-value | 0.75472075 |
| FWER p-Value | 1.0 |
Table: GSEA Results Summary

  

Fig 1: Enrichment plot: HALLMARK\_ESTROGEN\_RESPONSE\_LATE      
 Profile of the Running ES Score & Positions of GeneSet Members on the Rank Ordered List

  

| SYMBOL | TITLE | RANK IN GENE LIST | RANK METRIC SCORE | RUNNING ES | CORE ENRICHMENT || 1 | HOMER2 | homer scaffold protein 2 [Source:HGNC Symbol;Acc:HGNC:17513] | 37 | 2.189 | 0.0269 | Yes |
| 2 | LTF | lactotransferrin [Source:HGNC Symbol;Acc:HGNC:6720] | 42 | 2.154 | 0.0554 | Yes |
| 3 | SERPINA3 | serpin family A member 3 [Source:HGNC Symbol;Acc:HGNC:16] | 50 | 2.046 | 0.0823 | Yes |
| 4 | ST14 | ST14 transmembrane serine protease matriptase [Source:HGNC Symbol;Acc:HGNC:11344] | 136 | 1.687 | 0.0994 | Yes |
| 5 | MYB | "MYB proto-oncogene, transcription factor [Source:HGNC Symbol;Acc:HGNC:7545]" | 181 | 1.533 | 0.1170 | Yes |
| 6 | PLAC1 | placenta enriched 1 [Source:HGNC Symbol;Acc:HGNC:9044] | 217 | 1.451 | 0.1342 | Yes |
| 7 | CCN5 | cellular communication network factor 5 [Source:HGNC Symbol;Acc:HGNC:12770] | 398 | 1.171 | 0.1382 | Yes |
| 8 | MAPK13 | mitogen-activated protein kinase 13 [Source:HGNC Symbol;Acc:HGNC:6875] | 412 | 1.159 | 0.1528 | Yes |
| 9 | LSR | lipolysis stimulated lipoprotein receptor [Source:HGNC Symbol;Acc:HGNC:29572] | 481 | 1.090 | 0.1630 | Yes |
| 10 | TNNC1 | "troponin C1, slow skeletal and cardiac type [Source:HGNC Symbol;Acc:HGNC:11943]" | 648 | 0.963 | 0.1652 | Yes |
| 11 | BATF | basic leucine zipper ATF-like transcription factor [Source:HGNC Symbol;Acc:HGNC:958] | 653 | 0.960 | 0.1777 | Yes |
| 12 | PERP | p53 apoptosis effector related to PMP22 [Source:HGNC Symbol;Acc:HGNC:17637] | 657 | 0.957 | 0.1903 | Yes |
| 13 | PAPSS2 | 3'-phosphoadenosine 5'-phosphosulfate synthase 2 [Source:HGNC Symbol;Acc:HGNC:8604] | 985 | 0.828 | 0.1803 | Yes |
| 14 | PTPN6 | protein tyrosine phosphatase non-receptor type 6 [Source:HGNC Symbol;Acc:HGNC:9658] | 1049 | 0.790 | 0.1868 | Yes |
| 15 | CXCL12 | C-X-C motif chemokine ligand 12 [Source:HGNC Symbol;Acc:HGNC:10672] | 1092 | 0.774 | 0.1944 | Yes |
| 16 | TFPI2 | tissue factor pathway inhibitor 2 [Source:HGNC Symbol;Acc:HGNC:11761] | 1096 | 0.771 | 0.2045 | Yes |
| 17 | TPSAB1 | tryptase alpha/beta 1 [Source:HGNC Symbol;Acc:HGNC:12019] | 1147 | 0.751 | 0.2114 | Yes |
| 18 | SFN | stratifin [Source:HGNC Symbol;Acc:HGNC:10773] | 1200 | 0.732 | 0.2178 | Yes |
| 19 | PDLIM3 | PDZ and LIM domain 3 [Source:HGNC Symbol;Acc:HGNC:20767] | 1272 | 0.704 | 0.2226 | Yes |
| 20 | TIAM1 | TIAM Rac1 associated GEF 1 [Source:HGNC Symbol;Acc:HGNC:11805] | 1317 | 0.689 | 0.2290 | Yes |
| 21 | S100A9 | S100 calcium binding protein A9 [Source:HGNC Symbol;Acc:HGNC:10499] | 1434 | 0.650 | 0.2302 | Yes |
| 22 | SLC26A2 | solute carrier family 26 member 2 [Source:HGNC Symbol;Acc:HGNC:10994] | 1515 | 0.621 | 0.2333 | Yes |
| 23 | NRIP1 | nuclear receptor interacting protein 1 [Source:HGNC Symbol;Acc:HGNC:8001] | 1582 | 0.599 | 0.2371 | Yes |
| 24 | PLAAT3 | phospholipase A and acyltransferase 3 [Source:HGNC Symbol;Acc:HGNC:17825] | 1617 | 0.589 | 0.2427 | Yes |
| 25 | CA12 | carbonic anhydrase 12 [Source:HGNC Symbol;Acc:HGNC:1371] | 1688 | 0.571 | 0.2459 | Yes |
| 26 | SLC27A2 | solute carrier family 27 member 2 [Source:HGNC Symbol;Acc:HGNC:10996] | 1720 | 0.562 | 0.2514 | Yes |
| 27 | ALDH3B1 | aldehyde dehydrogenase 3 family member B1 [Source:HGNC Symbol;Acc:HGNC:410] | 1728 | 0.560 | 0.2584 | Yes |
| 28 | SNX10 | sorting nexin 10 [Source:HGNC Symbol;Acc:HGNC:14974] | 1751 | 0.555 | 0.2644 | Yes |
| 29 | UNC13B | unc-13 homolog B [Source:HGNC Symbol;Acc:HGNC:12566] | 1823 | 0.538 | 0.2670 | Yes |
| 30 | TMPRSS3 | transmembrane serine protease 3 [Source:HGNC Symbol;Acc:HGNC:11877] | 2012 | 0.503 | 0.2616 | Yes |
| 31 | PRLR | prolactin receptor [Source:HGNC Symbol;Acc:HGNC:9446] | 2014 | 0.503 | 0.2682 | Yes |
| 32 | NMU | neuromedin U [Source:HGNC Symbol;Acc:HGNC:7859] | 2021 | 0.503 | 0.2746 | Yes |
| 33 | TFF1 | trefoil factor 1 [Source:HGNC Symbol;Acc:HGNC:11755] | 2024 | 0.503 | 0.2812 | Yes |
| 34 | STIL | STIL centriolar assembly protein [Source:HGNC Symbol;Acc:HGNC:10879] | 2175 | 0.484 | 0.2779 | Yes |
| 35 | IGFBP4 | insulin like growth factor binding protein 4 [Source:HGNC Symbol;Acc:HGNC:5473] | 2183 | 0.482 | 0.2839 | Yes |
| 36 | ISG20 | interferon stimulated exonuclease gene 20 [Source:HGNC Symbol;Acc:HGNC:6130] | 2346 | 0.460 | 0.2796 | Yes |
| 37 | SLC9A3R1 | SLC9A3 regulator 1 [Source:HGNC Symbol;Acc:HGNC:11075] | 2348 | 0.460 | 0.2857 | Yes |
| 38 | RBBP8 | "RB binding protein 8, endonuclease [Source:HGNC Symbol;Acc:HGNC:9891]" | 2369 | 0.456 | 0.2905 | Yes |
| 39 | CISH | cytokine inducible SH2 containing protein [Source:HGNC Symbol;Acc:HGNC:1984] | 2374 | 0.455 | 0.2963 | Yes |
| 40 | KIF20A | kinesin family member 20A [Source:HGNC Symbol;Acc:HGNC:9787] | 2399 | 0.452 | 0.3008 | Yes |
| 41 | ACOX2 | acyl-CoA oxidase 2 [Source:HGNC Symbol;Acc:HGNC:120] | 2464 | 0.445 | 0.3026 | Yes |
| 42 | ASCL1 | achaete-scute family bHLH transcription factor 1 [Source:HGNC Symbol;Acc:HGNC:738] | 2483 | 0.445 | 0.3074 | Yes |
| 43 | TSPAN13 | tetraspanin 13 [Source:HGNC Symbol;Acc:HGNC:21643] | 2557 | 0.441 | 0.3086 | Yes |
| 44 | CACNA2D2 | calcium voltage-gated channel auxiliary subunit alpha2delta 2 [Source:HGNC Symbol;Acc:HGNC:1400] | 2725 | 0.426 | 0.3035 | No |
| 45 | GJB3 | gap junction protein beta 3 [Source:HGNC Symbol;Acc:HGNC:4285] | 3035 | 0.398 | 0.2889 | No |
| 46 | DYNLT3 | dynein light chain Tctex-type 3 [Source:HGNC Symbol;Acc:HGNC:11694] | 3186 | 0.375 | 0.2842 | No |
| 47 | AFF1 | AF4/FMR2 family member 1 [Source:HGNC Symbol;Acc:HGNC:7135] | 3231 | 0.369 | 0.2863 | No |
| 48 | TJP3 | tight junction protein 3 [Source:HGNC Symbol;Acc:HGNC:11829] | 3271 | 0.363 | 0.2886 | No |
| 49 | CHPT1 | choline phosphotransferase 1 [Source:HGNC Symbol;Acc:HGNC:17852] | 3289 | 0.359 | 0.2923 | No |
| 50 | GLA | galactosidase alpha [Source:HGNC Symbol;Acc:HGNC:4296] | 3461 | 0.333 | 0.2857 | No |
| 51 | TOP2A | DNA topoisomerase II alpha [Source:HGNC Symbol;Acc:HGNC:11989] | 3608 | 0.314 | 0.2805 | No |
| 52 | JAK2 | Janus kinase 2 [Source:HGNC Symbol;Acc:HGNC:6192] | 3644 | 0.308 | 0.2823 | No |
| 53 | ATP2B4 | ATPase plasma membrane Ca2+ transporting 4 [Source:HGNC Symbol;Acc:HGNC:817] | 3648 | 0.308 | 0.2863 | No |
| 54 | KLK10 | kallikrein related peptidase 10 [Source:HGNC Symbol;Acc:HGNC:6358] | 3861 | 0.283 | 0.2764 | No |
| 55 | CXCL14 | C-X-C motif chemokine ligand 14 [Source:HGNC Symbol;Acc:HGNC:10640] | 3880 | 0.281 | 0.2789 | No |
| 56 | CKB | creatine kinase B [Source:HGNC Symbol;Acc:HGNC:1991] | 3922 | 0.276 | 0.2800 | No |
| 57 | EGR3 | early growth response 3 [Source:HGNC Symbol;Acc:HGNC:3240] | 3940 | 0.274 | 0.2826 | No |
| 58 | ST6GALNAC2 | "ST6 N-acetylgalactosaminide alpha-2,6-sialyltransferase 2 [Source:HGNC Symbol;Acc:HGNC:10867]" | 3950 | 0.273 | 0.2856 | No |
| 59 | HR | HR lysine demethylase and nuclear receptor corepressor [Source:HGNC Symbol;Acc:HGNC:5172] | 3960 | 0.271 | 0.2887 | No |
| 60 | SLC16A1 | solute carrier family 16 member 1 [Source:HGNC Symbol;Acc:HGNC:10922] | 4007 | 0.265 | 0.2892 | No |
| 61 | SULT2B1 | sulfotransferase family 2B member 1 [Source:HGNC Symbol;Acc:HGNC:11459] | 4193 | 0.245 | 0.2806 | No |
| 62 | TFAP2C | transcription factor AP-2 gamma [Source:HGNC Symbol;Acc:HGNC:11744] | 4387 | 0.225 | 0.2711 | No |
| 63 | SGK1 | serum/glucocorticoid regulated kinase 1 [Source:HGNC Symbol;Acc:HGNC:10810] | 4462 | 0.217 | 0.2692 | No |
| 64 | OPN3 | opsin 3 [Source:HGNC Symbol;Acc:HGNC:14007] | 4749 | 0.193 | 0.2533 | No |
| 65 | FGFR3 | fibroblast growth factor receptor 3 [Source:HGNC Symbol;Acc:HGNC:3690] | 4951 | 0.172 | 0.2426 | No |
| 66 | METTL3 | methyltransferase like 3 [Source:HGNC Symbol;Acc:HGNC:17563] | 5033 | 0.165 | 0.2396 | No |
| 67 | PTGER3 | prostaglandin E receptor 3 [Source:HGNC Symbol;Acc:HGNC:9595] | 5089 | 0.159 | 0.2382 | No |
| 68 | PKP3 | plakophilin 3 [Source:HGNC Symbol;Acc:HGNC:9025] | 5128 | 0.156 | 0.2378 | No |
| 69 | TPBG | trophoblast glycoprotein [Source:HGNC Symbol;Acc:HGNC:12004] | 5191 | 0.151 | 0.2358 | No |
| 70 | TRIM29 | tripartite motif containing 29 [Source:HGNC Symbol;Acc:HGNC:17274] | 5286 | 0.143 | 0.2317 | No |
| 71 | PLK4 | polo like kinase 4 [Source:HGNC Symbol;Acc:HGNC:11397] | 5698 | 0.113 | 0.2066 | No |
| 72 | ANXA9 | annexin A9 [Source:HGNC Symbol;Acc:HGNC:547] | 5869 | 0.101 | 0.1970 | No |
| 73 | FLNB | filamin B [Source:HGNC Symbol;Acc:HGNC:3755] | 6035 | 0.088 | 0.1875 | No |
| 74 | SLC24A3 | solute carrier family 24 member 3 [Source:HGNC Symbol;Acc:HGNC:10977] | 6372 | 0.065 | 0.1667 | No |
| 75 | FDFT1 | farnesyl-diphosphate farnesyltransferase 1 [Source:HGNC Symbol;Acc:HGNC:3629] | 6427 | 0.060 | 0.1640 | No |
| 76 | TPD52L1 | TPD52 like 1 [Source:HGNC Symbol;Acc:HGNC:12006] | 6452 | 0.058 | 0.1632 | No |
| 77 | HSPB8 | heat shock protein family B (small) member 8 [Source:HGNC Symbol;Acc:HGNC:30171] | 6506 | 0.055 | 0.1606 | No |
| 78 | BTG3 | BTG anti-proliferation factor 3 [Source:HGNC Symbol;Acc:HGNC:1132] | 6622 | 0.047 | 0.1538 | No |
| 79 | FARP1 | "FERM, ARH/RhoGEF and pleckstrin domain protein 1 [Source:HGNC Symbol;Acc:HGNC:3591]" | 6834 | 0.034 | 0.1406 | No |
| 80 | MOCS2 | molybdenum cofactor synthesis 2 [Source:HGNC Symbol;Acc:HGNC:7193] | 6884 | 0.030 | 0.1378 | No |
| 81 | COX6C | cytochrome c oxidase subunit 6C [Source:HGNC Symbol;Acc:HGNC:2285] | 6901 | 0.029 | 0.1372 | No |
| 82 | PPIF | peptidylprolyl isomerase F [Source:HGNC Symbol;Acc:HGNC:9259] | 7150 | 0.015 | 0.1214 | No |
| 83 | JAK1 | Janus kinase 1 [Source:HGNC Symbol;Acc:HGNC:6190] | 7159 | 0.014 | 0.1211 | No |
| 84 | FOS | "Fos proto-oncogene, AP-1 transcription factor subunit [Source:HGNC Symbol;Acc:HGNC:3796]" | 7164 | 0.014 | 0.1210 | No |
| 85 | GFUS | GDP-L-fucose synthase [Source:HGNC Symbol;Acc:HGNC:12390] | 7233 | 0.010 | 0.1167 | No |
| 86 | BLVRB | biliverdin reductase B [Source:HGNC Symbol;Acc:HGNC:1063] | 7346 | 0.004 | 0.1095 | No |
| 87 | DLG5 | discs large MAGUK scaffold protein 5 [Source:HGNC Symbol;Acc:HGNC:2904] | 7386 | 0.001 | 0.1070 | No |
| 88 | SIAH2 | siah E3 ubiquitin protein ligase 2 [Source:HGNC Symbol;Acc:HGNC:10858] | 7444 | -0.001 | 0.1034 | No |
| 89 | FRK | fyn related Src family tyrosine kinase [Source:HGNC Symbol;Acc:HGNC:3955] | 7572 | -0.008 | 0.0953 | No |
| 90 | ITPK1 | inositol-tetrakisphosphate 1-kinase [Source:HGNC Symbol;Acc:HGNC:6177] | 7597 | -0.009 | 0.0939 | No |
| 91 | SLC7A5 | solute carrier family 7 member 5 [Source:HGNC Symbol;Acc:HGNC:11063] | 7939 | -0.026 | 0.0722 | No |
| 92 | CDC20 | cell division cycle 20 [Source:HGNC Symbol;Acc:HGNC:1723] | 7952 | -0.026 | 0.0718 | No |
| 93 | CDC6 | cell division cycle 6 [Source:HGNC Symbol;Acc:HGNC:1744] | 8163 | -0.038 | 0.0587 | No |
| 94 | RABEP1 | "rabaptin, RAB GTPase binding effector protein 1 [Source:HGNC Symbol;Acc:HGNC:17677]" | 8374 | -0.047 | 0.0458 | No |
| 95 | UGDH | UDP-glucose 6-dehydrogenase [Source:HGNC Symbol;Acc:HGNC:12525] | 8375 | -0.047 | 0.0464 | No |
| 96 | ID2 | inhibitor of DNA binding 2 [Source:HGNC Symbol;Acc:HGNC:5361] | 8387 | -0.048 | 0.0463 | No |
| 97 | SCNN1A | sodium channel epithelial 1 subunit alpha [Source:HGNC Symbol;Acc:HGNC:10599] | 8424 | -0.050 | 0.0447 | No |
| 98 | ALDH3A2 | aldehyde dehydrogenase 3 family member A2 [Source:HGNC Symbol;Acc:HGNC:403] | 8489 | -0.054 | 0.0413 | No |
| 99 | RET | ret proto-oncogene [Source:HGNC Symbol;Acc:HGNC:9967] | 8496 | -0.054 | 0.0416 | No |
| 100 | FKBP4 | FKBP prolyl isomerase 4 [Source:HGNC Symbol;Acc:HGNC:3720] | 8505 | -0.055 | 0.0418 | No |
| 101 | ZFP36 | ZFP36 ring finger protein [Source:HGNC Symbol;Acc:HGNC:12862] | 8519 | -0.056 | 0.0417 | No |
| 102 | IMPA2 | inositol monophosphatase 2 [Source:HGNC Symbol;Acc:HGNC:6051] | 8521 | -0.056 | 0.0424 | No |
| 103 | DUSP2 | dual specificity phosphatase 2 [Source:HGNC Symbol;Acc:HGNC:3068] | 8547 | -0.058 | 0.0416 | No |
| 104 | XRCC3 | X-ray repair cross complementing 3 [Source:HGNC Symbol;Acc:HGNC:12830] | 8566 | -0.058 | 0.0412 | No |
| 105 | DHCR7 | 7-dehydrocholesterol reductase [Source:HGNC Symbol;Acc:HGNC:2860] | 8984 | -0.082 | 0.0154 | No |
| 106 | KCNK5 | potassium two pore domain channel subfamily K member 5 [Source:HGNC Symbol;Acc:HGNC:6280] | 9343 | -0.100 | -0.0064 | No |
| 107 | HPRT1 | hypoxanthine phosphoribosyltransferase 1 [Source:HGNC Symbol;Acc:HGNC:5157] | 9568 | -0.116 | -0.0193 | No |
| 108 | MDK | midkine [Source:HGNC Symbol;Acc:HGNC:6972] | 9572 | -0.116 | -0.0180 | No |
| 109 | TOB1 | "transducer of ERBB2, 1 [Source:HGNC Symbol;Acc:HGNC:11979]" | 9783 | -0.130 | -0.0298 | No |
| 110 | CCND1 | cyclin D1 [Source:HGNC Symbol;Acc:HGNC:1582] | 9851 | -0.134 | -0.0324 | No |
| 111 | SLC22A5 | solute carrier family 22 member 5 [Source:HGNC Symbol;Acc:HGNC:10969] | 9853 | -0.134 | -0.0306 | No |
| 112 | ASS1 | argininosuccinate synthase 1 [Source:HGNC Symbol;Acc:HGNC:758] | 9933 | -0.138 | -0.0339 | No |
| 113 | SEMA3B | semaphorin 3B [Source:HGNC Symbol;Acc:HGNC:10724] | 9978 | -0.141 | -0.0348 | No |
| 114 | BAG1 | BAG cochaperone 1 [Source:HGNC Symbol;Acc:HGNC:937] | 10341 | -0.162 | -0.0561 | No |
| 115 | SCARB1 | scavenger receptor class B member 1 [Source:HGNC Symbol;Acc:HGNC:1664] | 10347 | -0.163 | -0.0542 | No |
| 116 | FKBP5 | FKBP prolyl isomerase 5 [Source:HGNC Symbol;Acc:HGNC:3721] | 10352 | -0.163 | -0.0523 | No |
| 117 | MYOF | myoferlin [Source:HGNC Symbol;Acc:HGNC:3656] | 10378 | -0.165 | -0.0517 | No |
| 118 | GINS2 | GINS complex subunit 2 [Source:HGNC Symbol;Acc:HGNC:24575] | 10398 | -0.166 | -0.0507 | No |
| 119 | KLF4 | Kruppel like factor 4 [Source:HGNC Symbol;Acc:HGNC:6348] | 10415 | -0.167 | -0.0495 | No |
| 120 | CD44 | CD44 molecule (Indian blood group) [Source:HGNC Symbol;Acc:HGNC:1681] | 10617 | -0.178 | -0.0601 | No |
| 121 | CYP26B1 | cytochrome P450 family 26 subfamily B member 1 [Source:HGNC Symbol;Acc:HGNC:20581] | 10745 | -0.185 | -0.0658 | No |
| 122 | DNAJC1 | DnaJ heat shock protein family (Hsp40) member C1 [Source:HGNC Symbol;Acc:HGNC:20090] | 10856 | -0.192 | -0.0704 | No |
| 123 | ABHD2 | "abhydrolase domain containing 2, acylglycerol lipase [Source:HGNC Symbol;Acc:HGNC:18717]" | 11126 | -0.208 | -0.0850 | No |
| 124 | PRKAR2B | protein kinase cAMP-dependent type II regulatory subunit beta [Source:HGNC Symbol;Acc:HGNC:9392] | 11261 | -0.215 | -0.0908 | No |
| 125 | EMP2 | epithelial membrane protein 2 [Source:HGNC Symbol;Acc:HGNC:3334] | 11520 | -0.230 | -0.1044 | No |
| 126 | HSPA4L | heat shock protein family A (Hsp70) member 4 like [Source:HGNC Symbol;Acc:HGNC:17041] | 11561 | -0.233 | -0.1038 | No |
| 127 | BCL2 | BCL2 apoptosis regulator [Source:HGNC Symbol;Acc:HGNC:990] | 11610 | -0.236 | -0.1038 | No |
| 128 | FABP5 | fatty acid binding protein 5 [Source:HGNC Symbol;Acc:HGNC:3560] | 11684 | -0.240 | -0.1053 | No |
| 129 | MAPT | microtubule associated protein tau [Source:HGNC Symbol;Acc:HGNC:6893] | 11822 | -0.249 | -0.1108 | No |
| 130 | SORD | sorbitol dehydrogenase [Source:HGNC Symbol;Acc:HGNC:11184] | 11863 | -0.252 | -0.1100 | No |
| 131 | AMFR | autocrine motility factor receptor [Source:HGNC Symbol;Acc:HGNC:463] | 11871 | -0.252 | -0.1071 | No |
| 132 | PTGES | prostaglandin E synthase [Source:HGNC Symbol;Acc:HGNC:9599] | 11951 | -0.257 | -0.1088 | No |
| 133 | NXT1 | nuclear transport factor 2 like export factor 1 [Source:HGNC Symbol;Acc:HGNC:15913] | 12073 | -0.265 | -0.1130 | No |
| 134 | SLC2A8 | solute carrier family 2 member 8 [Source:HGNC Symbol;Acc:HGNC:13812] | 12087 | -0.266 | -0.1103 | No |
| 135 | IL6ST | interleukin 6 signal transducer [Source:HGNC Symbol;Acc:HGNC:6021] | 12152 | -0.270 | -0.1108 | No |
| 136 | LAMC2 | laminin subunit gamma 2 [Source:HGNC Symbol;Acc:HGNC:6493] | 12173 | -0.272 | -0.1085 | No |
| 137 | CDH1 | cadherin 1 [Source:HGNC Symbol;Acc:HGNC:1748] | 12181 | -0.272 | -0.1053 | No |
| 138 | SCUBE2 | "signal peptide, CUB domain and EGF like domain containing 2 [Source:HGNC Symbol;Acc:HGNC:30425]" | 12205 | -0.273 | -0.1031 | No |
| 139 | GALE | UDP-galactose-4-epimerase [Source:HGNC Symbol;Acc:HGNC:4116] | 12319 | -0.282 | -0.1067 | No |
| 140 | TST | thiosulfate sulfurtransferase [Source:HGNC Symbol;Acc:HGNC:12388] | 12466 | -0.293 | -0.1122 | No |
| 141 | SLC29A1 | solute carrier family 29 member 1 (Augustine blood group) [Source:HGNC Symbol;Acc:HGNC:11003] | 12506 | -0.296 | -0.1107 | No |
| 142 | RAB31 | "RAB31, member RAS oncogene family [Source:HGNC Symbol;Acc:HGNC:9771]" | 12683 | -0.310 | -0.1180 | No |
| 143 | ADD3 | adducin 3 [Source:HGNC Symbol;Acc:HGNC:245] | 12843 | -0.323 | -0.1239 | No |
| 144 | NAB2 | NGFI-A binding protein 2 [Source:HGNC Symbol;Acc:HGNC:7627] | 12889 | -0.327 | -0.1225 | No |
| 145 | ELOVL5 | ELOVL fatty acid elongase 5 [Source:HGNC Symbol;Acc:HGNC:21308] | 12900 | -0.328 | -0.1187 | No |
| 146 | RNASEH2A | ribonuclease H2 subunit A [Source:HGNC Symbol;Acc:HGNC:18518] | 12960 | -0.334 | -0.1181 | No |
| 147 | LARGE1 | LARGE xylosyl- and glucuronyltransferase 1 [Source:HGNC Symbol;Acc:HGNC:6511] | 12999 | -0.338 | -0.1160 | No |
| 148 | AREG | amphiregulin [Source:HGNC Symbol;Acc:HGNC:651] | 13075 | -0.345 | -0.1162 | No |
| 149 | PLXNB1 | plexin B1 [Source:HGNC Symbol;Acc:HGNC:9103] | 13111 | -0.348 | -0.1138 | No |
| 150 | IL17RB | interleukin 17 receptor B [Source:HGNC Symbol;Acc:HGNC:18015] | 13115 | -0.348 | -0.1094 | No |
| 151 | NCOR2 | nuclear receptor corepressor 2 [Source:HGNC Symbol;Acc:HGNC:7673] | 13301 | -0.367 | -0.1164 | No |
| 152 | CD9 | CD9 molecule [Source:HGNC Symbol;Acc:HGNC:1709] | 13472 | -0.388 | -0.1222 | No |
| 153 | RAPGEFL1 | Rap guanine nucleotide exchange factor like 1 [Source:HGNC Symbol;Acc:HGNC:17428] | 13567 | -0.400 | -0.1229 | No |
| 154 | FAM102A | family with sequence similarity 102 member A [Source:HGNC Symbol;Acc:HGNC:31419] | 13578 | -0.402 | -0.1182 | No |
| 155 | XBP1 | X-box binding protein 1 [Source:HGNC Symbol;Acc:HGNC:12801] | 13607 | -0.406 | -0.1146 | No |
| 156 | PDCD4 | programmed cell death 4 [Source:HGNC Symbol;Acc:HGNC:8763] | 13615 | -0.407 | -0.1096 | No |
| 157 | CALCR | calcitonin receptor [Source:HGNC Symbol;Acc:HGNC:1440] | 13619 | -0.407 | -0.1043 | No |
| 158 | CLIC3 | chloride intracellular channel 3 [Source:HGNC Symbol;Acc:HGNC:2064] | 13632 | -0.409 | -0.0996 | No |
| 159 | OVOL2 | ovo like zinc finger 2 [Source:HGNC Symbol;Acc:HGNC:15804] | 13694 | -0.415 | -0.0980 | No |
| 160 | KRT13 | keratin 13 [Source:HGNC Symbol;Acc:HGNC:6415] | 13797 | -0.418 | -0.0990 | No |
| 161 | ABCA3 | ATP binding cassette subfamily A member 3 [Source:HGNC Symbol;Acc:HGNC:33] | 13805 | -0.419 | -0.0939 | No |
| 162 | IDH2 | isocitrate dehydrogenase (NADP(+)) 2 [Source:HGNC Symbol;Acc:HGNC:5383] | 13807 | -0.419 | -0.0883 | No |
| 163 | SERPINA1 | serpin family A member 1 [Source:HGNC Symbol;Acc:HGNC:8941] | 13828 | -0.422 | -0.0840 | No |
| 164 | NPY1R | neuropeptide Y receptor Y1 [Source:HGNC Symbol;Acc:HGNC:7956] | 13947 | -0.428 | -0.0859 | No |
| 165 | KRT19 | keratin 19 [Source:HGNC Symbol;Acc:HGNC:6436] | 13989 | -0.430 | -0.0828 | No |
| 166 | MEST | mesoderm specific transcript [Source:HGNC Symbol;Acc:HGNC:7028] | 14218 | -0.467 | -0.0912 | No |
| 167 | OLFM1 | olfactomedin 1 [Source:HGNC Symbol;Acc:HGNC:17187] | 14426 | -0.498 | -0.0980 | No |
| 168 | RPS6KA2 | ribosomal protein S6 kinase A2 [Source:HGNC Symbol;Acc:HGNC:10431] | 14545 | -0.523 | -0.0986 | No |
| 169 | LLGL2 | LLGL scribble cell polarity complex component 2 [Source:HGNC Symbol;Acc:HGNC:6629] | 14557 | -0.525 | -0.0923 | No |
| 170 | FOXC1 | forkhead box C1 [Source:HGNC Symbol;Acc:HGNC:3800] | 14579 | -0.528 | -0.0866 | No |
| 171 | ARL3 | ADP ribosylation factor like GTPase 3 [Source:HGNC Symbol;Acc:HGNC:694] | 14621 | -0.537 | -0.0820 | No |
| 172 | WFS1 | wolframin ER transmembrane glycoprotein [Source:HGNC Symbol;Acc:HGNC:12762] | 14644 | -0.541 | -0.0762 | No |
| 173 | PRSS23 | serine protease 23 [Source:HGNC Symbol;Acc:HGNC:14370] | 14750 | -0.567 | -0.0754 | No |
| 174 | DCXR | dicarbonyl and L-xylulose reductase [Source:HGNC Symbol;Acc:HGNC:18985] | 14777 | -0.576 | -0.0694 | No |
| 175 | ETFB | electron transfer flavoprotein subunit beta [Source:HGNC Symbol;Acc:HGNC:3482] | 14803 | -0.584 | -0.0632 | No |
| 176 | SLC1A4 | solute carrier family 1 member 4 [Source:HGNC Symbol;Acc:HGNC:10942] | 15030 | -0.677 | -0.0687 | No |
| 177 | CAV1 | caveolin 1 [Source:HGNC Symbol;Acc:HGNC:1527] | 15057 | -0.694 | -0.0612 | No |
| 178 | GAL | galanin and GMAP prepropeptide [Source:HGNC Symbol;Acc:HGNC:4114] | 15108 | -0.723 | -0.0547 | No |
| 179 | GPER1 | G protein-coupled estrogen receptor 1 [Source:HGNC Symbol;Acc:HGNC:4485] | 15159 | -0.754 | -0.0479 | No |
| 180 | CPE | carboxypeptidase E [Source:HGNC Symbol;Acc:HGNC:2303] | 15202 | -0.784 | -0.0401 | No |
| 181 | DNAJC12 | DnaJ heat shock protein family (Hsp40) member C12 [Source:HGNC Symbol;Acc:HGNC:28908] | 15248 | -0.805 | -0.0322 | No |
| 182 | CHST8 | carbohydrate sulfotransferase 8 [Source:HGNC Symbol;Acc:HGNC:15993] | 15394 | -0.885 | -0.0298 | No |
| 183 | CELSR2 | cadherin EGF LAG seven-pass G-type receptor 2 [Source:HGNC Symbol;Acc:HGNC:3231] | 15484 | -1.013 | -0.0220 | No |
| 184 | CA2 | carbonic anhydrase 2 [Source:HGNC Symbol;Acc:HGNC:1373] | 15522 | -1.087 | -0.0098 | No |
| 185 | PDZK1 | PDZ domain containing 1 [Source:HGNC Symbol;Acc:HGNC:8821] | 15624 | -1.437 | 0.0028 | No |
Table: GSEA details [plain text format]

  

Fig 2: HALLMARK\_ESTROGEN\_RESPONSE\_LATE      
 Blue-Pink O' Gram in the Space of the Analyzed GeneSet

  

Fig 3: HALLMARK\_ESTROGEN\_RESPONSE\_LATE: Random ES distribution      
 Gene set null distribution of ES for **HALLMARK\_ESTROGEN\_RESPONSE\_LATE**
