# Supplementary material for: In vivo CRISPR screens reveal Serpinb9 and Adam2 as regulators of immune therapy response in lung cancer
Source: Nat Commun. 2023 May 31;14:3150. doi: 10.1038/s41467-023-38841-7 (PMC10232477; doi:10.1038/s41467-023-38841-7)
Supplement: Supplementary file 13 — Source Data [file 41467_2023_38841_MOESM13_ESM.zip › Figure 4E.zip!/my_analysis.Gsea.1640146340255_Hallmark/HALLMARK_UNFOLDED_PROTEIN_RESPONSE.html]

Details for gene set HALLMARK\_UNFOLDED\_PROTEIN\_RESPONSE[GSEA]

|  || Dataset | December 21, 2021\_for GSEA run\_collapsed\_to\_symbols.GSEA\_B6\_NI.cls #CTLR\_NI\_versus\_ADAM2\_NI |
| Phenotype | GSEA\_B6\_NI.cls#CTLR\_NI\_versus\_ADAM2\_NI |
| Upregulated in class | ADAM2\_NI |
| GeneSet | HALLMARK\_UNFOLDED\_PROTEIN\_RESPONSE |
| Enrichment Score (ES) | -0.33004543 |
| Normalized Enrichment Score (NES) | -1.575278 |
| Nominal p-value | 0.0 |
| FDR q-value | 0.4345125 |
| FWER p-Value | 0.332 |
Table: GSEA Results Summary

  

Fig 1: Enrichment plot: HALLMARK\_UNFOLDED\_PROTEIN\_RESPONSE      
 Profile of the Running ES Score & Positions of GeneSet Members on the Rank Ordered List

  

| SYMBOL | TITLE | RANK IN GENE LIST | RANK METRIC SCORE | RUNNING ES | CORE ENRICHMENT || 1 | ATF3 | activating transcription factor 3 [Source:HGNC Symbol;Acc:HGNC:785] | 453 | 1.117 | 0.0127 | No |
| 2 | DCP2 | decapping mRNA 2 [Source:HGNC Symbol;Acc:HGNC:24452] | 660 | 0.957 | 0.0353 | No |
| 3 | NABP1 | nucleic acid binding protein 1 [Source:HGNC Symbol;Acc:HGNC:26232] | 2253 | 0.476 | -0.0492 | No |
| 4 | KIF5B | kinesin family member 5B [Source:HGNC Symbol;Acc:HGNC:6324] | 3329 | 0.353 | -0.1051 | No |
| 5 | CEBPG | CCAAT enhancer binding protein gamma [Source:HGNC Symbol;Acc:HGNC:1837] | 3361 | 0.348 | -0.0940 | No |
| 6 | CHAC1 | ChaC glutathione specific gamma-glutamylcyclotransferase 1 [Source:HGNC Symbol;Acc:HGNC:28680] | 3416 | 0.340 | -0.0848 | No |
| 7 | CCL2 | C-C motif chemokine ligand 2 [Source:HGNC Symbol;Acc:HGNC:10618] | 3622 | 0.312 | -0.0863 | No |
| 8 | DCP1A | decapping mRNA 1A [Source:HGNC Symbol;Acc:HGNC:18714] | 3827 | 0.288 | -0.0886 | No |
| 9 | ASNS | asparagine synthetase (glutamine-hydrolyzing) [Source:HGNC Symbol;Acc:HGNC:753] | 3870 | 0.282 | -0.0807 | No |
| 10 | GEMIN4 | gem nuclear organelle associated protein 4 [Source:HGNC Symbol;Acc:HGNC:15717] | 4128 | 0.251 | -0.0878 | No |
| 11 | ATF4 | activating transcription factor 4 [Source:HGNC Symbol;Acc:HGNC:786] | 4130 | 0.251 | -0.0785 | No |
| 12 | CEBPB | CCAAT enhancer binding protein beta [Source:HGNC Symbol;Acc:HGNC:1834] | 4438 | 0.219 | -0.0900 | No |
| 13 | SPCS1 | signal peptidase complex subunit 1 [Source:HGNC Symbol;Acc:HGNC:23401] | 4529 | 0.211 | -0.0879 | No |
| 14 | EIF2S1 | eukaryotic translation initiation factor 2 subunit alpha [Source:HGNC Symbol;Acc:HGNC:3265] | 4656 | 0.200 | -0.0885 | No |
| 15 | TATDN2 | TatD DNase domain containing 2 [Source:HGNC Symbol;Acc:HGNC:28988] | 4669 | 0.199 | -0.0818 | No |
| 16 | CXXC1 | CXXC finger protein 1 [Source:HGNC Symbol;Acc:HGNC:24343] | 4800 | 0.188 | -0.0832 | No |
| 17 | FUS | FUS RNA binding protein [Source:HGNC Symbol;Acc:HGNC:4010] | 4808 | 0.187 | -0.0766 | No |
| 18 | DNAJA4 | DnaJ heat shock protein family (Hsp40) member A4 [Source:HGNC Symbol;Acc:HGNC:14885] | 4946 | 0.174 | -0.0789 | No |
| 19 | CNOT6 | CCR4-NOT transcription complex subunit 6 [Source:HGNC Symbol;Acc:HGNC:14099] | 5088 | 0.159 | -0.0820 | No |
| 20 | SDAD1 | SDA1 domain containing 1 [Source:HGNC Symbol;Acc:HGNC:25537] | 5600 | 0.119 | -0.1104 | No |
| 21 | HSPA9 | heat shock protein family A (Hsp70) member 9 [Source:HGNC Symbol;Acc:HGNC:5244] | 5752 | 0.109 | -0.1160 | No |
| 22 | NFYB | nuclear transcription factor Y subunit beta [Source:HGNC Symbol;Acc:HGNC:7805] | 5775 | 0.107 | -0.1134 | No |
| 23 | PSAT1 | phosphoserine aminotransferase 1 [Source:HGNC Symbol;Acc:HGNC:19129] | 5776 | 0.107 | -0.1094 | No |
| 24 | EIF4A2 | eukaryotic translation initiation factor 4A2 [Source:HGNC Symbol;Acc:HGNC:3284] | 6055 | 0.087 | -0.1241 | No |
| 25 | TARS1 | threonyl-tRNA synthetase 1 [Source:HGNC Symbol;Acc:HGNC:11572] | 6091 | 0.083 | -0.1232 | No |
| 26 | EIF4G1 | eukaryotic translation initiation factor 4 gamma 1 [Source:HGNC Symbol;Acc:HGNC:3296] | 6104 | 0.082 | -0.1209 | No |
| 27 | H2AX | H2A.X variant histone [Source:HGNC Symbol;Acc:HGNC:4739] | 6134 | 0.080 | -0.1197 | No |
| 28 | EXOSC9 | exosome component 9 [Source:HGNC Symbol;Acc:HGNC:9137] | 6464 | 0.057 | -0.1387 | No |
| 29 | TTC37 | tetratricopeptide repeat domain 37 [Source:HGNC Symbol;Acc:HGNC:23639] | 6718 | 0.041 | -0.1534 | No |
| 30 | EXOC2 | exocyst complex component 2 [Source:HGNC Symbol;Acc:HGNC:24968] | 6810 | 0.036 | -0.1580 | No |
| 31 | MTREX | Mtr4 exosome RNA helicase [Source:HGNC Symbol;Acc:HGNC:18734] | 6913 | 0.029 | -0.1634 | No |
| 32 | PARN | poly(A)-specific ribonuclease [Source:HGNC Symbol;Acc:HGNC:8609] | 7050 | 0.021 | -0.1714 | No |
| 33 | EDEM1 | ER degradation enhancing alpha-mannosidase like protein 1 [Source:HGNC Symbol;Acc:HGNC:18967] | 7078 | 0.019 | -0.1724 | No |
| 34 | ERO1A | endoplasmic reticulum oxidoreductase 1 alpha [Source:HGNC Symbol;Acc:HGNC:13280] | 7181 | 0.013 | -0.1785 | No |
| 35 | GOSR2 | golgi SNAP receptor complex member 2 [Source:HGNC Symbol;Acc:HGNC:4431] | 7476 | -0.003 | -0.1972 | No |
| 36 | VEGFA | vascular endothelial growth factor A [Source:HGNC Symbol;Acc:HGNC:12680] | 7671 | -0.012 | -0.2092 | No |
| 37 | FKBP14 | FKBP prolyl isomerase 14 [Source:HGNC Symbol;Acc:HGNC:18625] | 7714 | -0.014 | -0.2114 | No |
| 38 | PREB | prolactin regulatory element binding [Source:HGNC Symbol;Acc:HGNC:9356] | 7852 | -0.021 | -0.2194 | No |
| 39 | POP4 | "POP4 homolog, ribonuclease P/MRP subunit [Source:HGNC Symbol;Acc:HGNC:30081]" | 7891 | -0.023 | -0.2210 | No |
| 40 | MTHFD2 | "methylenetetrahydrofolate dehydrogenase (NADP+ dependent) 2, methenyltetrahydrofolate cyclohydrolase [Source:HGNC Symbol;Acc:HGNC:7434]" | 7932 | -0.025 | -0.2226 | No |
| 41 | SLC7A5 | solute carrier family 7 member 5 [Source:HGNC Symbol;Acc:HGNC:11063] | 7939 | -0.026 | -0.2220 | No |
| 42 | IARS1 | isoleucyl-tRNA synthetase 1 [Source:HGNC Symbol;Acc:HGNC:5330] | 8025 | -0.030 | -0.2264 | No |
| 43 | DDX10 | DEAD-box helicase 10 [Source:HGNC Symbol;Acc:HGNC:2735] | 8206 | -0.040 | -0.2364 | No |
| 44 | WIPI1 | "WD repeat domain, phosphoinositide interacting 1 [Source:HGNC Symbol;Acc:HGNC:25471]" | 8329 | -0.045 | -0.2426 | No |
| 45 | RPS14 | ribosomal protein S14 [Source:HGNC Symbol;Acc:HGNC:10387] | 8405 | -0.049 | -0.2456 | No |
| 46 | YWHAZ | tyrosine 3-monooxygenase/tryptophan 5-monooxygenase activation protein zeta [Source:HGNC Symbol;Acc:HGNC:12855] | 8694 | -0.066 | -0.2616 | No |
| 47 | EIF4E | eukaryotic translation initiation factor 4E [Source:HGNC Symbol;Acc:HGNC:3287] | 8798 | -0.073 | -0.2655 | No |
| 48 | EDC4 | enhancer of mRNA decapping 4 [Source:HGNC Symbol;Acc:HGNC:17157] | 9291 | -0.098 | -0.2935 | No |
| 49 | HSP90B1 | heat shock protein 90 beta family member 1 [Source:HGNC Symbol;Acc:HGNC:12028] | 9406 | -0.105 | -0.2969 | No |
| 50 | STC2 | stanniocalcin 2 [Source:HGNC Symbol;Acc:HGNC:11374] | 9474 | -0.108 | -0.2971 | No |
| 51 | EIF4A1 | eukaryotic translation initiation factor 4A1 [Source:HGNC Symbol;Acc:HGNC:3282] | 9488 | -0.110 | -0.2939 | No |
| 52 | NOP14 | NOP14 nucleolar protein [Source:HGNC Symbol;Acc:HGNC:16821] | 9499 | -0.110 | -0.2904 | No |
| 53 | EXOSC2 | exosome component 2 [Source:HGNC Symbol;Acc:HGNC:17097] | 9796 | -0.131 | -0.3045 | No |
| 54 | ERN1 | endoplasmic reticulum to nucleus signaling 1 [Source:HGNC Symbol;Acc:HGNC:3449] | 9894 | -0.136 | -0.3056 | No |
| 55 | CNOT2 | CCR4-NOT transcription complex subunit 2 [Source:HGNC Symbol;Acc:HGNC:7878] | 9895 | -0.136 | -0.3005 | No |
| 56 | NPM1 | nucleophosmin 1 [Source:HGNC Symbol;Acc:HGNC:7910] | 10355 | -0.163 | -0.3239 | Yes |
| 57 | XPOT | exportin for tRNA [Source:HGNC Symbol;Acc:HGNC:12826] | 10363 | -0.164 | -0.3183 | Yes |
| 58 | IMP3 | IMP U3 small nucleolar ribonucleoprotein 3 [Source:HGNC Symbol;Acc:HGNC:14497] | 10423 | -0.167 | -0.3158 | Yes |
| 59 | ARFGAP1 | ADP ribosylation factor GTPase activating protein 1 [Source:HGNC Symbol;Acc:HGNC:15852] | 10549 | -0.174 | -0.3173 | Yes |
| 60 | NFYA | nuclear transcription factor Y subunit alpha [Source:HGNC Symbol;Acc:HGNC:7804] | 10568 | -0.175 | -0.3119 | Yes |
| 61 | CNOT4 | CCR4-NOT transcription complex subunit 4 [Source:HGNC Symbol;Acc:HGNC:7880] | 10619 | -0.179 | -0.3084 | Yes |
| 62 | ATF6 | activating transcription factor 6 [Source:HGNC Symbol;Acc:HGNC:791] | 10784 | -0.188 | -0.3119 | Yes |
| 63 | PAIP1 | poly(A) binding protein interacting protein 1 [Source:HGNC Symbol;Acc:HGNC:16945] | 10844 | -0.191 | -0.3086 | Yes |
| 64 | SERP1 | stress associated endoplasmic reticulum protein 1 [Source:HGNC Symbol;Acc:HGNC:10759] | 10912 | -0.195 | -0.3056 | Yes |
| 65 | PDIA6 | protein disulfide isomerase family A member 6 [Source:HGNC Symbol;Acc:HGNC:30168] | 10956 | -0.198 | -0.3009 | Yes |
| 66 | DNAJC3 | DnaJ heat shock protein family (Hsp40) member C3 [Source:HGNC Symbol;Acc:HGNC:9439] | 10973 | -0.199 | -0.2945 | Yes |
| 67 | EXOSC10 | exosome component 10 [Source:HGNC Symbol;Acc:HGNC:9138] | 11188 | -0.211 | -0.3004 | Yes |
| 68 | CKS1B | CDC28 protein kinase regulatory subunit 1B [Source:HGNC Symbol;Acc:HGNC:19083] | 11243 | -0.214 | -0.2958 | Yes |
| 69 | HERPUD1 | homocysteine inducible ER protein with ubiquitin like domain 1 [Source:HGNC Symbol;Acc:HGNC:13744] | 11319 | -0.218 | -0.2924 | Yes |
| 70 | KDELR3 | KDEL endoplasmic reticulum protein retention receptor 3 [Source:HGNC Symbol;Acc:HGNC:6306] | 11322 | -0.219 | -0.2844 | Yes |
| 71 | NHP2 | NHP2 ribonucleoprotein [Source:HGNC Symbol;Acc:HGNC:14377] | 11362 | -0.221 | -0.2786 | Yes |
| 72 | PDIA5 | protein disulfide isomerase family A member 5 [Source:HGNC Symbol;Acc:HGNC:24811] | 11438 | -0.226 | -0.2750 | Yes |
| 73 | TUBB2A | tubulin beta 2A class IIa [Source:HGNC Symbol;Acc:HGNC:12412] | 11519 | -0.230 | -0.2715 | Yes |
| 74 | EXOSC5 | exosome component 5 [Source:HGNC Symbol;Acc:HGNC:24662] | 11694 | -0.240 | -0.2737 | Yes |
| 75 | EXOSC1 | exosome component 1 [Source:HGNC Symbol;Acc:HGNC:17286] | 11704 | -0.241 | -0.2652 | Yes |
| 76 | EIF4A3 | eukaryotic translation initiation factor 4A3 [Source:HGNC Symbol;Acc:HGNC:18683] | 11786 | -0.247 | -0.2612 | Yes |
| 77 | EIF4EBP1 | eukaryotic translation initiation factor 4E binding protein 1 [Source:HGNC Symbol;Acc:HGNC:3288] | 11846 | -0.250 | -0.2556 | Yes |
| 78 | KHSRP | KH-type splicing regulatory protein [Source:HGNC Symbol;Acc:HGNC:6316] | 11878 | -0.252 | -0.2481 | Yes |
| 79 | DKC1 | dyskerin pseudouridine synthase 1 [Source:HGNC Symbol;Acc:HGNC:2890] | 11911 | -0.254 | -0.2407 | Yes |
| 80 | SSR1 | signal sequence receptor subunit 1 [Source:HGNC Symbol;Acc:HGNC:11323] | 12057 | -0.264 | -0.2401 | Yes |
| 81 | ALDH18A1 | aldehyde dehydrogenase 18 family member A1 [Source:HGNC Symbol;Acc:HGNC:9722] | 12069 | -0.265 | -0.2309 | Yes |
| 82 | SEC11A | "SEC11 homolog A, signal peptidase complex subunit [Source:HGNC Symbol;Acc:HGNC:17718]" | 12278 | -0.279 | -0.2338 | Yes |
| 83 | SLC30A5 | solute carrier family 30 member 5 [Source:HGNC Symbol;Acc:HGNC:19089] | 12279 | -0.279 | -0.2233 | Yes |
| 84 | BANF1 | BAF nuclear assembly factor 1 [Source:HGNC Symbol;Acc:HGNC:17397] | 12292 | -0.280 | -0.2136 | Yes |
| 85 | BAG3 | BAG cochaperone 3 [Source:HGNC Symbol;Acc:HGNC:939] | 12384 | -0.288 | -0.2087 | Yes |
| 86 | EXOSC4 | exosome component 4 [Source:HGNC Symbol;Acc:HGNC:18189] | 12554 | -0.301 | -0.2083 | Yes |
| 87 | HSPA5 | heat shock protein family A (Hsp70) member 5 [Source:HGNC Symbol;Acc:HGNC:5238] | 12586 | -0.303 | -0.1989 | Yes |
| 88 | LSM1 | "LSM1 homolog, mRNA degradation associated [Source:HGNC Symbol;Acc:HGNC:20472]" | 12689 | -0.311 | -0.1938 | Yes |
| 89 | SRPRA | SRP receptor subunit alpha [Source:HGNC Symbol;Acc:HGNC:11307] | 12693 | -0.311 | -0.1824 | Yes |
| 90 | ATP6V0D1 | ATPase H+ transporting V0 subunit d1 [Source:HGNC Symbol;Acc:HGNC:13724] | 12761 | -0.316 | -0.1748 | Yes |
| 91 | TSPYL2 | TSPY like 2 [Source:HGNC Symbol;Acc:HGNC:24358] | 12840 | -0.323 | -0.1678 | Yes |
| 92 | CALR | calreticulin [Source:HGNC Symbol;Acc:HGNC:1455] | 12856 | -0.324 | -0.1566 | Yes |
| 93 | RRP9 | "ribosomal RNA processing 9, U3 small nucleolar RNA binding protein [Source:HGNC Symbol;Acc:HGNC:16829]" | 12955 | -0.334 | -0.1504 | Yes |
| 94 | SHC1 | SHC adaptor protein 1 [Source:HGNC Symbol;Acc:HGNC:10840] | 12965 | -0.335 | -0.1384 | Yes |
| 95 | SPCS3 | signal peptidase complex subunit 3 [Source:HGNC Symbol;Acc:HGNC:26212] | 12968 | -0.335 | -0.1260 | Yes |
| 96 | NOP56 | NOP56 ribonucleoprotein [Source:HGNC Symbol;Acc:HGNC:15911] | 13004 | -0.338 | -0.1156 | Yes |
| 97 | SEC31A | "SEC31 homolog A, COPII coat complex component [Source:HGNC Symbol;Acc:HGNC:17052]" | 13053 | -0.343 | -0.1058 | Yes |
| 98 | LSM4 | "LSM4 homolog, U6 small nuclear RNA and mRNA degradation associated [Source:HGNC Symbol;Acc:HGNC:17259]" | 13302 | -0.368 | -0.1080 | Yes |
| 99 | ZBTB17 | zinc finger and BTB domain containing 17 [Source:HGNC Symbol;Acc:HGNC:12936] | 13467 | -0.388 | -0.1040 | Yes |
| 100 | EIF2AK3 | eukaryotic translation initiation factor 2 alpha kinase 3 [Source:HGNC Symbol;Acc:HGNC:3255] | 13549 | -0.398 | -0.0943 | Yes |
| 101 | XBP1 | X-box binding protein 1 [Source:HGNC Symbol;Acc:HGNC:12801] | 13607 | -0.406 | -0.0828 | Yes |
| 102 | HYOU1 | hypoxia up-regulated 1 [Source:HGNC Symbol;Acc:HGNC:16931] | 14143 | -0.457 | -0.1000 | Yes |
| 103 | DNAJB9 | DnaJ heat shock protein family (Hsp40) member B9 [Source:HGNC Symbol;Acc:HGNC:6968] | 14214 | -0.467 | -0.0871 | Yes |
| 104 | DCTN1 | dynactin subunit 1 [Source:HGNC Symbol;Acc:HGNC:2711] | 14298 | -0.481 | -0.0744 | Yes |
| 105 | NOLC1 | nucleolar and coiled-body phosphoprotein 1 [Source:HGNC Symbol;Acc:HGNC:15608] | 14380 | -0.490 | -0.0612 | Yes |
| 106 | YIF1A | "Yip1 interacting factor homolog A, membrane trafficking protein [Source:HGNC Symbol;Acc:HGNC:16688]" | 14436 | -0.500 | -0.0460 | Yes |
| 107 | WFS1 | wolframin ER transmembrane glycoprotein [Source:HGNC Symbol;Acc:HGNC:12762] | 14644 | -0.541 | -0.0391 | Yes |
| 108 | EEF2 | eukaryotic translation elongation factor 2 [Source:HGNC Symbol;Acc:HGNC:3214] | 14826 | -0.592 | -0.0285 | Yes |
| 109 | SRPRB | SRP receptor subunit beta [Source:HGNC Symbol;Acc:HGNC:24085] | 14987 | -0.659 | -0.0141 | Yes |
| 110 | SLC1A4 | solute carrier family 1 member 4 [Source:HGNC Symbol;Acc:HGNC:10942] | 15030 | -0.677 | 0.0085 | Yes |
| 111 | DDIT4 | DNA damage inducible transcript 4 [Source:HGNC Symbol;Acc:HGNC:24944] | 15359 | -0.865 | 0.0199 | Yes |
Table: GSEA details [plain text format]

  

Fig 2: HALLMARK\_UNFOLDED\_PROTEIN\_RESPONSE      
 Blue-Pink O' Gram in the Space of the Analyzed GeneSet

  

Fig 3: HALLMARK\_UNFOLDED\_PROTEIN\_RESPONSE: Random ES distribution      
 Gene set null distribution of ES for **HALLMARK\_UNFOLDED\_PROTEIN\_RESPONSE**
