# Supplementary material for: In vivo CRISPR screens reveal Serpinb9 and Adam2 as regulators of immune therapy response in lung cancer
Source: Nat Commun. 2023 May 31;14:3150. doi: 10.1038/s41467-023-38841-7 (PMC10232477; doi:10.1038/s41467-023-38841-7)
Supplement: Supplementary file 13 — Source Data [file 41467_2023_38841_MOESM13_ESM.zip › Figure 4E.zip!/my_analysis.Gsea.1640146340255_Hallmark/HALLMARK_HEDGEHOG_SIGNALING.html]

Details for gene set HALLMARK\_HEDGEHOG\_SIGNALING[GSEA]

|  || Dataset | December 21, 2021\_for GSEA run\_collapsed\_to\_symbols.GSEA\_B6\_NI.cls #CTLR\_NI\_versus\_ADAM2\_NI |
| Phenotype | GSEA\_B6\_NI.cls#CTLR\_NI\_versus\_ADAM2\_NI |
| Upregulated in class | CTLR\_NI |
| GeneSet | HALLMARK\_HEDGEHOG\_SIGNALING |
| Enrichment Score (ES) | 0.35559395 |
| Normalized Enrichment Score (NES) | 1.0608103 |
| Nominal p-value | 0.3007663 |
| FDR q-value | 0.6982789 |
| FWER p-Value | 1.0 |
Table: GSEA Results Summary

  

Fig 1: Enrichment plot: HALLMARK\_HEDGEHOG\_SIGNALING      
 Profile of the Running ES Score & Positions of GeneSet Members on the Rank Ordered List

  

| SYMBOL | TITLE | RANK IN GENE LIST | RANK METRIC SCORE | RUNNING ES | CORE ENRICHMENT || 1 | CRMP1 | collapsin response mediator protein 1 [Source:HGNC Symbol;Acc:HGNC:2365] | 106 | 1.775 | 0.1544 | Yes |
| 2 | CNTFR | ciliary neurotrophic factor receptor [Source:HGNC Symbol;Acc:HGNC:2170] | 760 | 0.892 | 0.1937 | Yes |
| 3 | GLI1 | GLI family zinc finger 1 [Source:HGNC Symbol;Acc:HGNC:4317] | 976 | 0.832 | 0.2554 | Yes |
| 4 | HEY2 | hes related family bHLH transcription factor with YRPW motif 2 [Source:HGNC Symbol;Acc:HGNC:4881] | 1121 | 0.763 | 0.3155 | Yes |
| 5 | UNC5C | unc-5 netrin receptor C [Source:HGNC Symbol;Acc:HGNC:12569] | 1699 | 0.568 | 0.3302 | Yes |
| 6 | DPYSL2 | dihydropyrimidinase like 2 [Source:HGNC Symbol;Acc:HGNC:3014] | 2273 | 0.472 | 0.3364 | Yes |
| 7 | SHH | sonic hedgehog signaling molecule [Source:HGNC Symbol;Acc:HGNC:10848] | 2893 | 0.411 | 0.3341 | Yes |
| 8 | PTCH1 | patched 1 [Source:HGNC Symbol;Acc:HGNC:9585] | 3244 | 0.367 | 0.3450 | Yes |
| 9 | ETS2 | "ETS proto-oncogene 2, transcription factor [Source:HGNC Symbol;Acc:HGNC:3489]" | 3538 | 0.323 | 0.3556 | Yes |
| 10 | OPHN1 | oligophrenin 1 [Source:HGNC Symbol;Acc:HGNC:8148] | 5077 | 0.160 | 0.2718 | No |
| 11 | CELSR1 | cadherin EGF LAG seven-pass G-type receptor 1 [Source:HGNC Symbol;Acc:HGNC:1850] | 5431 | 0.133 | 0.2613 | No |
| 12 | RASA1 | RAS p21 protein activator 1 [Source:HGNC Symbol;Acc:HGNC:9871] | 5873 | 0.101 | 0.2422 | No |
| 13 | HEY1 | hes related family bHLH transcription factor with YRPW motif 1 [Source:HGNC Symbol;Acc:HGNC:4880] | 6301 | 0.070 | 0.2213 | No |
| 14 | MYH9 | myosin heavy chain 9 [Source:HGNC Symbol;Acc:HGNC:7579] | 6387 | 0.063 | 0.2216 | No |
| 15 | THY1 | Thy-1 cell surface antigen [Source:HGNC Symbol;Acc:HGNC:11801] | 6495 | 0.055 | 0.2198 | No |
| 16 | PML | PML nuclear body scaffold [Source:HGNC Symbol;Acc:HGNC:9113] | 6860 | 0.032 | 0.1994 | No |
| 17 | VLDLR | very low density lipoprotein receptor [Source:HGNC Symbol;Acc:HGNC:12698] | 7123 | 0.017 | 0.1842 | No |
| 18 | CDK6 | cyclin dependent kinase 6 [Source:HGNC Symbol;Acc:HGNC:1777] | 7351 | 0.004 | 0.1700 | No |
| 19 | L1CAM | L1 cell adhesion molecule [Source:HGNC Symbol;Acc:HGNC:6470] | 7545 | -0.006 | 0.1582 | No |
| 20 | VEGFA | vascular endothelial growth factor A [Source:HGNC Symbol;Acc:HGNC:12680] | 7671 | -0.012 | 0.1513 | No |
| 21 | RTN1 | reticulon 1 [Source:HGNC Symbol;Acc:HGNC:10467] | 8105 | -0.035 | 0.1268 | No |
| 22 | ADGRG1 | adhesion G protein-coupled receptor G1 [Source:HGNC Symbol;Acc:HGNC:4512] | 9016 | -0.084 | 0.0762 | No |
| 23 | NRP1 | neuropilin 1 [Source:HGNC Symbol;Acc:HGNC:8004] | 9151 | -0.091 | 0.0759 | No |
| 24 | TLE3 | "TLE family member 3, transcriptional corepressor [Source:HGNC Symbol;Acc:HGNC:11839]" | 9266 | -0.096 | 0.0774 | No |
| 25 | NF1 | neurofibromin 1 [Source:HGNC Symbol;Acc:HGNC:7765] | 9703 | -0.125 | 0.0608 | No |
| 26 | LDB1 | LIM domain binding 1 [Source:HGNC Symbol;Acc:HGNC:6532] | 10228 | -0.156 | 0.0414 | No |
| 27 | NRP2 | neuropilin 2 [Source:HGNC Symbol;Acc:HGNC:8005] | 11760 | -0.245 | -0.0343 | No |
| 28 | NRCAM | neuronal cell adhesion molecule [Source:HGNC Symbol;Acc:HGNC:7994] | 11877 | -0.252 | -0.0188 | No |
| 29 | CDK5R1 | cyclin dependent kinase 5 regulatory subunit 1 [Source:HGNC Symbol;Acc:HGNC:1775] | 14006 | -0.433 | -0.1156 | No |
| 30 | AMOT | angiomotin [Source:HGNC Symbol;Acc:HGNC:17810] | 14287 | -0.480 | -0.0899 | No |
| 31 | ACHE | acetylcholinesterase (Cartwright blood group) [Source:HGNC Symbol;Acc:HGNC:108] | 14808 | -0.585 | -0.0700 | No |
| 32 | SLIT1 | slit guidance ligand 1 [Source:HGNC Symbol;Acc:HGNC:11085] | 14886 | -0.614 | -0.0192 | No |
| 33 | TLE1 | "TLE family member 1, transcriptional corepressor [Source:HGNC Symbol;Acc:HGNC:11837]" | 15174 | -0.762 | 0.0316 | No |
Table: GSEA details [plain text format]

  

Fig 2: HALLMARK\_HEDGEHOG\_SIGNALING      
 Blue-Pink O' Gram in the Space of the Analyzed GeneSet

  

Fig 3: HALLMARK\_HEDGEHOG\_SIGNALING: Random ES distribution      
 Gene set null distribution of ES for **HALLMARK\_HEDGEHOG\_SIGNALING**
