# Supplementary material for: In vivo CRISPR screens reveal Serpinb9 and Adam2 as regulators of immune therapy response in lung cancer
Source: Nat Commun. 2023 May 31;14:3150. doi: 10.1038/s41467-023-38841-7 (PMC10232477; doi:10.1038/s41467-023-38841-7)
Supplement: Supplementary file 13 — Source Data [file 41467_2023_38841_MOESM13_ESM.zip › Figure 4E.zip!/my_analysis.Gsea.1640146340255_Hallmark/HALLMARK_MYC_TARGETS_V1.html]

Details for gene set HALLMARK\_MYC\_TARGETS\_V1[GSEA]

|  || Dataset | December 21, 2021\_for GSEA run\_collapsed\_to\_symbols.GSEA\_B6\_NI.cls #CTLR\_NI\_versus\_ADAM2\_NI |
| Phenotype | GSEA\_B6\_NI.cls#CTLR\_NI\_versus\_ADAM2\_NI |
| Upregulated in class | ADAM2\_NI |
| GeneSet | HALLMARK\_MYC\_TARGETS\_V1 |
| Enrichment Score (ES) | -0.33397493 |
| Normalized Enrichment Score (NES) | -0.92982805 |
| Nominal p-value | 0.6134122 |
| FDR q-value | 1.0 |
| FWER p-Value | 1.0 |
Table: GSEA Results Summary

  

Fig 1: Enrichment plot: HALLMARK\_MYC\_TARGETS\_V1      
 Profile of the Running ES Score & Positions of GeneSet Members on the Rank Ordered List

  

| SYMBOL | TITLE | RANK IN GENE LIST | RANK METRIC SCORE | RUNNING ES | CORE ENRICHMENT || 1 | PSMC6 | "proteasome 26S subunit, ATPase 6 [Source:HGNC Symbol;Acc:HGNC:9553]" | 1677 | 0.574 | -0.0951 | No |
| 2 | IFRD1 | interferon related developmental regulator 1 [Source:HGNC Symbol;Acc:HGNC:5456] | 1881 | 0.528 | -0.0960 | No |
| 3 | ODC1 | ornithine decarboxylase 1 [Source:HGNC Symbol;Acc:HGNC:8109] | 2154 | 0.489 | -0.1023 | No |
| 4 | PSMA6 | proteasome 20S subunit alpha 6 [Source:HGNC Symbol;Acc:HGNC:9535] | 2549 | 0.443 | -0.1175 | No |
| 5 | RFC4 | replication factor C subunit 4 [Source:HGNC Symbol;Acc:HGNC:9972] | 3006 | 0.403 | -0.1376 | No |
| 6 | XPO1 | exportin 1 [Source:HGNC Symbol;Acc:HGNC:12825] | 3047 | 0.395 | -0.1311 | No |
| 7 | CDC45 | cell division cycle 45 [Source:HGNC Symbol;Acc:HGNC:1739] | 3085 | 0.390 | -0.1244 | No |
| 8 | MCM4 | minichromosome maintenance complex component 4 [Source:HGNC Symbol;Acc:HGNC:6947] | 3229 | 0.369 | -0.1251 | No |
| 9 | ACP1 | acid phosphatase 1 [Source:HGNC Symbol;Acc:HGNC:122] | 3457 | 0.334 | -0.1321 | No |
| 10 | ETF1 | eukaryotic translation termination factor 1 [Source:HGNC Symbol;Acc:HGNC:3477] | 4021 | 0.263 | -0.1624 | No |
| 11 | TOMM70 | translocase of outer mitochondrial membrane 70 [Source:HGNC Symbol;Acc:HGNC:11985] | 4118 | 0.252 | -0.1628 | No |
| 12 | PSMA4 | proteasome 20S subunit alpha 4 [Source:HGNC Symbol;Acc:HGNC:9533] | 4124 | 0.252 | -0.1573 | No |
| 13 | SNRPB2 | small nuclear ribonucleoprotein polypeptide B2 [Source:HGNC Symbol;Acc:HGNC:11155] | 4245 | 0.238 | -0.1595 | No |
| 14 | SNRPD1 | small nuclear ribonucleoprotein D1 polypeptide [Source:HGNC Symbol;Acc:HGNC:11158] | 4329 | 0.230 | -0.1596 | No |
| 15 | CDK2 | cyclin dependent kinase 2 [Source:HGNC Symbol;Acc:HGNC:1771] | 4345 | 0.228 | -0.1553 | No |
| 16 | DEK | DEK proto-oncogene [Source:HGNC Symbol;Acc:HGNC:2768] | 4368 | 0.226 | -0.1515 | No |
| 17 | RANBP1 | RAN binding protein 1 [Source:HGNC Symbol;Acc:HGNC:9847] | 4441 | 0.219 | -0.1511 | No |
| 18 | H2AZ1 | H2A.Z variant histone 1 [Source:HGNC Symbol;Acc:HGNC:4741] | 4447 | 0.218 | -0.1463 | No |
| 19 | SRSF1 | serine and arginine rich splicing factor 1 [Source:HGNC Symbol;Acc:HGNC:10780] | 4515 | 0.212 | -0.1458 | No |
| 20 | PSMA2 | proteasome 20S subunit alpha 2 [Source:HGNC Symbol;Acc:HGNC:9531] | 4594 | 0.205 | -0.1460 | No |
| 21 | GSPT1 | G1 to S phase transition 1 [Source:HGNC Symbol;Acc:HGNC:4621] | 4622 | 0.203 | -0.1431 | No |
| 22 | TYMS | thymidylate synthetase [Source:HGNC Symbol;Acc:HGNC:12441] | 4624 | 0.203 | -0.1385 | No |
| 23 | EIF2S1 | eukaryotic translation initiation factor 2 subunit alpha [Source:HGNC Symbol;Acc:HGNC:3265] | 4656 | 0.200 | -0.1358 | No |
| 24 | HNRNPA2B1 | heterogeneous nuclear ribonucleoprotein A2/B1 [Source:HGNC Symbol;Acc:HGNC:5033] | 4684 | 0.198 | -0.1330 | No |
| 25 | KPNA2 | karyopherin subunit alpha 2 [Source:HGNC Symbol;Acc:HGNC:6395] | 4831 | 0.185 | -0.1382 | No |
| 26 | HNRNPD | heterogeneous nuclear ribonucleoprotein D [Source:HGNC Symbol;Acc:HGNC:5036] | 4894 | 0.178 | -0.1380 | No |
| 27 | RPS10 | ribosomal protein S10 [Source:HGNC Symbol;Acc:HGNC:10383] | 4987 | 0.169 | -0.1401 | No |
| 28 | DHX15 | DEAH-box helicase 15 [Source:HGNC Symbol;Acc:HGNC:2738] | 5013 | 0.166 | -0.1378 | No |
| 29 | MAD2L1 | mitotic arrest deficient 2 like 1 [Source:HGNC Symbol;Acc:HGNC:6763] | 5028 | 0.165 | -0.1349 | No |
| 30 | COPS5 | COP9 signalosome subunit 5 [Source:HGNC Symbol;Acc:HGNC:2240] | 5186 | 0.152 | -0.1415 | No |
| 31 | TRA2B | transformer 2 beta homolog [Source:HGNC Symbol;Acc:HGNC:10781] | 5272 | 0.145 | -0.1437 | No |
| 32 | SRSF2 | serine and arginine rich splicing factor 2 [Source:HGNC Symbol;Acc:HGNC:10783] | 5308 | 0.141 | -0.1427 | No |
| 33 | PSMD1 | "proteasome 26S subunit, non-ATPase 1 [Source:HGNC Symbol;Acc:HGNC:9554]" | 5558 | 0.123 | -0.1559 | No |
| 34 | ORC2 | origin recognition complex subunit 2 [Source:HGNC Symbol;Acc:HGNC:8488] | 5652 | 0.115 | -0.1593 | No |
| 35 | RRM1 | ribonucleotide reductase catalytic subunit M1 [Source:HGNC Symbol;Acc:HGNC:10451] | 5711 | 0.111 | -0.1604 | No |
| 36 | PSMA1 | proteasome 20S subunit alpha 1 [Source:HGNC Symbol;Acc:HGNC:9530] | 5719 | 0.111 | -0.1583 | No |
| 37 | EPRS1 | glutamyl-prolyl-tRNA synthetase 1 [Source:HGNC Symbol;Acc:HGNC:3418] | 5774 | 0.107 | -0.1593 | No |
| 38 | SSB | small RNA binding exonuclease protection factor La [Source:HGNC Symbol;Acc:HGNC:11316] | 5808 | 0.105 | -0.1591 | No |
| 39 | GNL3 | G protein nucleolar 3 [Source:HGNC Symbol;Acc:HGNC:29931] | 5863 | 0.101 | -0.1602 | No |
| 40 | HNRNPA3 | heterogeneous nuclear ribonucleoprotein A3 [Source:HGNC Symbol;Acc:HGNC:24941] | 5895 | 0.099 | -0.1599 | No |
| 41 | SNRPD3 | small nuclear ribonucleoprotein D3 polypeptide [Source:HGNC Symbol;Acc:HGNC:11160] | 5899 | 0.099 | -0.1578 | No |
| 42 | RSL1D1 | ribosomal L1 domain containing 1 [Source:HGNC Symbol;Acc:HGNC:24534] | 5923 | 0.097 | -0.1571 | No |
| 43 | HNRNPU | heterogeneous nuclear ribonucleoprotein U [Source:HGNC Symbol;Acc:HGNC:5048] | 5992 | 0.092 | -0.1593 | No |
| 44 | CCNA2 | cyclin A2 [Source:HGNC Symbol;Acc:HGNC:1578] | 6083 | 0.084 | -0.1632 | No |
| 45 | PSMA7 | proteasome 20S subunit alpha 7 [Source:HGNC Symbol;Acc:HGNC:9536] | 6090 | 0.083 | -0.1617 | No |
| 46 | COX5A | cytochrome c oxidase subunit 5A [Source:HGNC Symbol;Acc:HGNC:2267] | 6129 | 0.081 | -0.1622 | No |
| 47 | PCNA | proliferating cell nuclear antigen [Source:HGNC Symbol;Acc:HGNC:8729] | 6214 | 0.075 | -0.1659 | No |
| 48 | AP3S1 | adaptor related protein complex 3 subunit sigma 1 [Source:HGNC Symbol;Acc:HGNC:2013] | 6312 | 0.069 | -0.1706 | No |
| 49 | MYC | "MYC proto-oncogene, bHLH transcription factor [Source:HGNC Symbol;Acc:HGNC:7553]" | 6504 | 0.055 | -0.1817 | No |
| 50 | LSM7 | "LSM7 homolog, U6 small nuclear RNA and mRNA degradation associated [Source:HGNC Symbol;Acc:HGNC:20470]" | 6551 | 0.052 | -0.1835 | No |
| 51 | HSPD1 | heat shock protein family D (Hsp60) member 1 [Source:HGNC Symbol;Acc:HGNC:5261] | 6586 | 0.050 | -0.1845 | No |
| 52 | RAD23B | "RAD23 homolog B, nucleotide excision repair protein [Source:HGNC Symbol;Acc:HGNC:9813]" | 6677 | 0.044 | -0.1893 | No |
| 53 | PSMD14 | "proteasome 26S subunit, non-ATPase 14 [Source:HGNC Symbol;Acc:HGNC:16889]" | 6687 | 0.043 | -0.1889 | No |
| 54 | FAM120A | family with sequence similarity 120A [Source:HGNC Symbol;Acc:HGNC:13247] | 6741 | 0.041 | -0.1914 | No |
| 55 | TXNL4A | thioredoxin like 4A [Source:HGNC Symbol;Acc:HGNC:30551] | 6778 | 0.038 | -0.1928 | No |
| 56 | SRSF3 | serine and arginine rich splicing factor 3 [Source:HGNC Symbol;Acc:HGNC:10785] | 6787 | 0.037 | -0.1925 | No |
| 57 | YWHAQ | tyrosine 3-monooxygenase/tryptophan 5-monooxygenase activation protein theta [Source:HGNC Symbol;Acc:HGNC:12854] | 6822 | 0.035 | -0.1939 | No |
| 58 | UBE2L3 | ubiquitin conjugating enzyme E2 L3 [Source:HGNC Symbol;Acc:HGNC:12488] | 6920 | 0.028 | -0.1995 | No |
| 59 | TARDBP | TAR DNA binding protein [Source:HGNC Symbol;Acc:HGNC:11571] | 7060 | 0.020 | -0.2080 | No |
| 60 | UBE2E1 | ubiquitin conjugating enzyme E2 E1 [Source:HGNC Symbol;Acc:HGNC:12477] | 7131 | 0.016 | -0.2122 | No |
| 61 | NCBP2 | nuclear cap binding protein subunit 2 [Source:HGNC Symbol;Acc:HGNC:7659] | 7268 | 0.009 | -0.2207 | No |
| 62 | SYNCRIP | synaptotagmin binding cytoplasmic RNA interacting protein [Source:HGNC Symbol;Acc:HGNC:16918] | 7274 | 0.008 | -0.2209 | No |
| 63 | HNRNPC | heterogeneous nuclear ribonucleoprotein C [Source:HGNC Symbol;Acc:HGNC:5035] | 7442 | -0.001 | -0.2316 | No |
| 64 | USP1 | ubiquitin specific peptidase 1 [Source:HGNC Symbol;Acc:HGNC:12607] | 7744 | -0.016 | -0.2507 | No |
| 65 | APEX1 | apurinic/apyrimidinic endodeoxyribonuclease 1 [Source:HGNC Symbol;Acc:HGNC:587] | 7768 | -0.017 | -0.2518 | No |
| 66 | EIF3J | eukaryotic translation initiation factor 3 subunit J [Source:HGNC Symbol;Acc:HGNC:3270] | 7794 | -0.018 | -0.2530 | No |
| 67 | NCBP1 | nuclear cap binding protein subunit 1 [Source:HGNC Symbol;Acc:HGNC:7658] | 7863 | -0.022 | -0.2569 | No |
| 68 | HNRNPR | heterogeneous nuclear ribonucleoprotein R [Source:HGNC Symbol;Acc:HGNC:5047] | 7869 | -0.022 | -0.2567 | No |
| 69 | CNBP | CCHC-type zinc finger nucleic acid binding protein [Source:HGNC Symbol;Acc:HGNC:13164] | 7912 | -0.025 | -0.2589 | No |
| 70 | DDX18 | DEAD-box helicase 18 [Source:HGNC Symbol;Acc:HGNC:2741] | 7942 | -0.026 | -0.2602 | No |
| 71 | KPNB1 | karyopherin subunit beta 1 [Source:HGNC Symbol;Acc:HGNC:6400] | 7950 | -0.026 | -0.2600 | No |
| 72 | CDC20 | cell division cycle 20 [Source:HGNC Symbol;Acc:HGNC:1723] | 7952 | -0.026 | -0.2595 | No |
| 73 | SRSF7 | serine and arginine rich splicing factor 7 [Source:HGNC Symbol;Acc:HGNC:10789] | 7956 | -0.027 | -0.2590 | No |
| 74 | EIF2S2 | eukaryotic translation initiation factor 2 subunit beta [Source:HGNC Symbol;Acc:HGNC:3266] | 7960 | -0.027 | -0.2586 | No |
| 75 | IARS1 | isoleucyl-tRNA synthetase 1 [Source:HGNC Symbol;Acc:HGNC:5330] | 8025 | -0.030 | -0.2620 | No |
| 76 | HSPE1 | heat shock protein family E (Hsp10) member 1 [Source:HGNC Symbol;Acc:HGNC:5269] | 8177 | -0.039 | -0.2709 | No |
| 77 | HDAC2 | histone deacetylase 2 [Source:HGNC Symbol;Acc:HGNC:4853] | 8194 | -0.040 | -0.2710 | No |
| 78 | VBP1 | VHL binding protein 1 [Source:HGNC Symbol;Acc:HGNC:12662] | 8325 | -0.045 | -0.2784 | No |
| 79 | SRPK1 | SRSF protein kinase 1 [Source:HGNC Symbol;Acc:HGNC:11305] | 8383 | -0.048 | -0.2810 | No |
| 80 | SSBP1 | single stranded DNA binding protein 1 [Source:HGNC Symbol;Acc:HGNC:11317] | 8432 | -0.051 | -0.2829 | No |
| 81 | PSMD8 | "proteasome 26S subunit, non-ATPase 8 [Source:HGNC Symbol;Acc:HGNC:9566]" | 8584 | -0.059 | -0.2913 | No |
| 82 | U2AF1 | U2 small nuclear RNA auxiliary factor 1 [Source:HGNC Symbol;Acc:HGNC:12453] | 8698 | -0.066 | -0.2970 | No |
| 83 | VDAC3 | voltage dependent anion channel 3 [Source:HGNC Symbol;Acc:HGNC:12674] | 8705 | -0.067 | -0.2959 | No |
| 84 | SET | SET nuclear proto-oncogene [Source:HGNC Symbol;Acc:HGNC:10760] | 8751 | -0.069 | -0.2972 | No |
| 85 | DUT | deoxyuridine triphosphatase [Source:HGNC Symbol;Acc:HGNC:3078] | 8767 | -0.070 | -0.2965 | No |
| 86 | EIF4E | eukaryotic translation initiation factor 4E [Source:HGNC Symbol;Acc:HGNC:3287] | 8798 | -0.073 | -0.2968 | No |
| 87 | UBA2 | ubiquitin like modifier activating enzyme 2 [Source:HGNC Symbol;Acc:HGNC:30661] | 8880 | -0.077 | -0.3002 | No |
| 88 | PSMB2 | proteasome 20S subunit beta 2 [Source:HGNC Symbol;Acc:HGNC:9539] | 8925 | -0.078 | -0.3013 | No |
| 89 | CYC1 | cytochrome c1 [Source:HGNC Symbol;Acc:HGNC:2579] | 8964 | -0.080 | -0.3019 | No |
| 90 | ERH | ERH mRNA splicing and mitosis factor [Source:HGNC Symbol;Acc:HGNC:3447] | 8972 | -0.081 | -0.3004 | No |
| 91 | PTGES3 | prostaglandin E synthase 3 [Source:HGNC Symbol;Acc:HGNC:16049] | 9118 | -0.089 | -0.3078 | No |
| 92 | NME1 | NME/NM23 nucleoside diphosphate kinase 1 [Source:HGNC Symbol;Acc:HGNC:7849] | 9314 | -0.099 | -0.3181 | No |
| 93 | KARS1 | lysyl-tRNA synthetase 1 [Source:HGNC Symbol;Acc:HGNC:6215] | 9351 | -0.101 | -0.3181 | No |
| 94 | CCT4 | chaperonin containing TCP1 subunit 4 [Source:HGNC Symbol;Acc:HGNC:1617] | 9461 | -0.107 | -0.3226 | No |
| 95 | EIF4A1 | eukaryotic translation initiation factor 4A1 [Source:HGNC Symbol;Acc:HGNC:3282] | 9488 | -0.110 | -0.3218 | No |
| 96 | EIF4G2 | eukaryotic translation initiation factor 4 gamma 2 [Source:HGNC Symbol;Acc:HGNC:3297] | 9525 | -0.113 | -0.3215 | No |
| 97 | HPRT1 | hypoxanthine phosphoribosyltransferase 1 [Source:HGNC Symbol;Acc:HGNC:5157] | 9568 | -0.116 | -0.3215 | No |
| 98 | MCM5 | minichromosome maintenance complex component 5 [Source:HGNC Symbol;Acc:HGNC:6948] | 9727 | -0.126 | -0.3288 | No |
| 99 | PHB2 | prohibitin 2 [Source:HGNC Symbol;Acc:HGNC:30306] | 9754 | -0.128 | -0.3275 | No |
| 100 | NAP1L1 | nucleosome assembly protein 1 like 1 [Source:HGNC Symbol;Acc:HGNC:7637] | 9805 | -0.131 | -0.3277 | No |
| 101 | PRPF31 | pre-mRNA processing factor 31 [Source:HGNC Symbol;Acc:HGNC:15446] | 9903 | -0.137 | -0.3308 | Yes |
| 102 | PWP1 | "PWP1 homolog, endonuclein [Source:HGNC Symbol;Acc:HGNC:17015]" | 9934 | -0.138 | -0.3295 | Yes |
| 103 | HNRNPA1 | heterogeneous nuclear ribonucleoprotein A1 [Source:HGNC Symbol;Acc:HGNC:5031] | 9962 | -0.140 | -0.3281 | Yes |
| 104 | CCT5 | chaperonin containing TCP1 subunit 5 [Source:HGNC Symbol;Acc:HGNC:1618] | 10014 | -0.143 | -0.3280 | Yes |
| 105 | CSTF2 | cleavage stimulation factor subunit 2 [Source:HGNC Symbol;Acc:HGNC:2484] | 10023 | -0.144 | -0.3252 | Yes |
| 106 | ABCE1 | ATP binding cassette subfamily E member 1 [Source:HGNC Symbol;Acc:HGNC:69] | 10033 | -0.144 | -0.3225 | Yes |
| 107 | PA2G4 | proliferation-associated 2G4 [Source:HGNC Symbol;Acc:HGNC:8550] | 10108 | -0.148 | -0.3238 | Yes |
| 108 | VDAC1 | voltage dependent anion channel 1 [Source:HGNC Symbol;Acc:HGNC:12669] | 10163 | -0.151 | -0.3238 | Yes |
| 109 | SNRPA1 | small nuclear ribonucleoprotein polypeptide A' [Source:HGNC Symbol;Acc:HGNC:11152] | 10167 | -0.152 | -0.3205 | Yes |
| 110 | MCM6 | minichromosome maintenance complex component 6 [Source:HGNC Symbol;Acc:HGNC:6949] | 10218 | -0.155 | -0.3201 | Yes |
| 111 | CUL1 | cullin 1 [Source:HGNC Symbol;Acc:HGNC:2551] | 10236 | -0.156 | -0.3176 | Yes |
| 112 | MCM7 | minichromosome maintenance complex component 7 [Source:HGNC Symbol;Acc:HGNC:6950] | 10302 | -0.160 | -0.3181 | Yes |
| 113 | NPM1 | nucleophosmin 1 [Source:HGNC Symbol;Acc:HGNC:7910] | 10355 | -0.163 | -0.3177 | Yes |
| 114 | XPOT | exportin for tRNA [Source:HGNC Symbol;Acc:HGNC:12826] | 10363 | -0.164 | -0.3144 | Yes |
| 115 | YWHAE | tyrosine 3-monooxygenase/tryptophan 5-monooxygenase activation protein epsilon [Source:HGNC Symbol;Acc:HGNC:12851] | 10366 | -0.164 | -0.3107 | Yes |
| 116 | PSMC4 | "proteasome 26S subunit, ATPase 4 [Source:HGNC Symbol;Acc:HGNC:9551]" | 10382 | -0.165 | -0.3079 | Yes |
| 117 | EXOSC7 | exosome component 7 [Source:HGNC Symbol;Acc:HGNC:28112] | 10425 | -0.167 | -0.3067 | Yes |
| 118 | CAD | "carbamoyl-phosphate synthetase 2, aspartate transcarbamylase, and dihydroorotase [Source:HGNC Symbol;Acc:HGNC:1424]" | 10443 | -0.168 | -0.3039 | Yes |
| 119 | RAN | "RAN, member RAS oncogene family [Source:HGNC Symbol;Acc:HGNC:9846]" | 10509 | -0.172 | -0.3041 | Yes |
| 120 | SF3A1 | splicing factor 3a subunit 1 [Source:HGNC Symbol;Acc:HGNC:10765] | 10584 | -0.176 | -0.3049 | Yes |
| 121 | POLE3 | "DNA polymerase epsilon 3, accessory subunit [Source:HGNC Symbol;Acc:HGNC:13546]" | 10614 | -0.178 | -0.3026 | Yes |
| 122 | SMARCC1 | "SWI/SNF related, matrix associated, actin dependent regulator of chromatin subfamily c member 1 [Source:HGNC Symbol;Acc:HGNC:11104]" | 10675 | -0.181 | -0.3023 | Yes |
| 123 | MCM2 | minichromosome maintenance complex component 2 [Source:HGNC Symbol;Acc:HGNC:6944] | 10713 | -0.183 | -0.3005 | Yes |
| 124 | SF3B3 | splicing factor 3b subunit 3 [Source:HGNC Symbol;Acc:HGNC:10770] | 10750 | -0.186 | -0.2985 | Yes |
| 125 | AIMP2 | aminoacyl tRNA synthetase complex interacting multifunctional protein 2 [Source:HGNC Symbol;Acc:HGNC:20609] | 10991 | -0.200 | -0.3094 | Yes |
| 126 | PSMD3 | "proteasome 26S subunit, non-ATPase 3 [Source:HGNC Symbol;Acc:HGNC:9560]" | 11071 | -0.204 | -0.3098 | Yes |
| 127 | PHB | prohibitin [Source:HGNC Symbol;Acc:HGNC:8912] | 11119 | -0.207 | -0.3080 | Yes |
| 128 | NDUFAB1 | NADH:ubiquinone oxidoreductase subunit AB1 [Source:HGNC Symbol;Acc:HGNC:7694] | 11124 | -0.207 | -0.3035 | Yes |
| 129 | PABPC1 | poly(A) binding protein cytoplasmic 1 [Source:HGNC Symbol;Acc:HGNC:8554] | 11145 | -0.209 | -0.2999 | Yes |
| 130 | CCT2 | chaperonin containing TCP1 subunit 2 [Source:HGNC Symbol;Acc:HGNC:1615] | 11157 | -0.210 | -0.2958 | Yes |
| 131 | MRPS18B | mitochondrial ribosomal protein S18B [Source:HGNC Symbol;Acc:HGNC:14516] | 11180 | -0.210 | -0.2923 | Yes |
| 132 | DDX21 | DExD-box helicase 21 [Source:HGNC Symbol;Acc:HGNC:2744] | 11268 | -0.215 | -0.2930 | Yes |
| 133 | G3BP1 | G3BP stress granule assembly factor 1 [Source:HGNC Symbol;Acc:HGNC:30292] | 11272 | -0.215 | -0.2882 | Yes |
| 134 | CBX3 | chromobox 3 [Source:HGNC Symbol;Acc:HGNC:1553] | 11298 | -0.217 | -0.2848 | Yes |
| 135 | LSM2 | "LSM2 homolog, U6 small nuclear RNA and mRNA degradation associated [Source:HGNC Symbol;Acc:HGNC:13940]" | 11336 | -0.219 | -0.2821 | Yes |
| 136 | NHP2 | NHP2 ribonucleoprotein [Source:HGNC Symbol;Acc:HGNC:14377] | 11362 | -0.221 | -0.2786 | Yes |
| 137 | SNRPG | small nuclear ribonucleoprotein polypeptide G [Source:HGNC Symbol;Acc:HGNC:11163] | 11389 | -0.223 | -0.2751 | Yes |
| 138 | EIF4H | eukaryotic translation initiation factor 4H [Source:HGNC Symbol;Acc:HGNC:12741] | 11409 | -0.224 | -0.2711 | Yes |
| 139 | NOP16 | NOP16 nucleolar protein [Source:HGNC Symbol;Acc:HGNC:26934] | 11426 | -0.225 | -0.2670 | Yes |
| 140 | BUB3 | BUB3 mitotic checkpoint protein [Source:HGNC Symbol;Acc:HGNC:1151] | 11432 | -0.225 | -0.2621 | Yes |
| 141 | CCT7 | chaperonin containing TCP1 subunit 7 [Source:HGNC Symbol;Acc:HGNC:1622] | 11447 | -0.227 | -0.2577 | Yes |
| 142 | PSMD7 | "proteasome 26S subunit, non-ATPase 7 [Source:HGNC Symbol;Acc:HGNC:9565]" | 11496 | -0.229 | -0.2555 | Yes |
| 143 | PSMB3 | proteasome 20S subunit beta 3 [Source:HGNC Symbol;Acc:HGNC:9540] | 11569 | -0.233 | -0.2548 | Yes |
| 144 | RUVBL2 | RuvB like AAA ATPase 2 [Source:HGNC Symbol;Acc:HGNC:10475] | 11582 | -0.234 | -0.2501 | Yes |
| 145 | EIF1AX | eukaryotic translation initiation factor 1A X-linked [Source:HGNC Symbol;Acc:HGNC:3250] | 11691 | -0.240 | -0.2515 | Yes |
| 146 | RPL34 | ribosomal protein L34 [Source:HGNC Symbol;Acc:HGNC:10340] | 11791 | -0.247 | -0.2522 | Yes |
| 147 | ILF2 | interleukin enhancer binding factor 2 [Source:HGNC Symbol;Acc:HGNC:6037] | 11815 | -0.249 | -0.2479 | Yes |
| 148 | EIF3D | eukaryotic translation initiation factor 3 subunit D [Source:HGNC Symbol;Acc:HGNC:3278] | 11922 | -0.255 | -0.2489 | Yes |
| 149 | CLNS1A | chloride nucleotide-sensitive channel 1A [Source:HGNC Symbol;Acc:HGNC:2080] | 12089 | -0.267 | -0.2535 | Yes |
| 150 | TRIM28 | tripartite motif containing 28 [Source:HGNC Symbol;Acc:HGNC:16384] | 12122 | -0.268 | -0.2493 | Yes |
| 151 | FBL | fibrillarin [Source:HGNC Symbol;Acc:HGNC:3599] | 12177 | -0.272 | -0.2465 | Yes |
| 152 | MRPL9 | mitochondrial ribosomal protein L9 [Source:HGNC Symbol;Acc:HGNC:14277] | 12194 | -0.273 | -0.2412 | Yes |
| 153 | RNPS1 | RNA binding protein with serine rich domain 1 [Source:HGNC Symbol;Acc:HGNC:10080] | 12251 | -0.277 | -0.2384 | Yes |
| 154 | CTPS1 | CTP synthase 1 [Source:HGNC Symbol;Acc:HGNC:2519] | 12310 | -0.281 | -0.2357 | Yes |
| 155 | PRDX3 | peroxiredoxin 3 [Source:HGNC Symbol;Acc:HGNC:9354] | 12317 | -0.282 | -0.2295 | Yes |
| 156 | TFDP1 | transcription factor Dp-1 [Source:HGNC Symbol;Acc:HGNC:11749] | 12351 | -0.285 | -0.2251 | Yes |
| 157 | XRCC6 | X-ray repair cross complementing 6 [Source:HGNC Symbol;Acc:HGNC:4055] | 12394 | -0.288 | -0.2211 | Yes |
| 158 | CANX | calnexin [Source:HGNC Symbol;Acc:HGNC:1473] | 12536 | -0.299 | -0.2233 | Yes |
| 159 | HDGF | heparin binding growth factor [Source:HGNC Symbol;Acc:HGNC:4856] | 12538 | -0.299 | -0.2164 | Yes |
| 160 | HSP90AB1 | heat shock protein 90 alpha family class B member 1 [Source:HGNC Symbol;Acc:HGNC:5258] | 12665 | -0.309 | -0.2174 | Yes |
| 161 | SERBP1 | SERPINE1 mRNA binding protein 1 [Source:HGNC Symbol;Acc:HGNC:17860] | 12669 | -0.309 | -0.2105 | Yes |
| 162 | PPM1G | "protein phosphatase, Mg2+/Mn2+ dependent 1G [Source:HGNC Symbol;Acc:HGNC:9278]" | 12718 | -0.313 | -0.2063 | Yes |
| 163 | PPIA | peptidylprolyl isomerase A [Source:HGNC Symbol;Acc:HGNC:9253] | 12733 | -0.314 | -0.2000 | Yes |
| 164 | C1QBP | complement C1q binding protein [Source:HGNC Symbol;Acc:HGNC:1243] | 12800 | -0.319 | -0.1968 | Yes |
| 165 | GOT2 | glutamic-oxaloacetic transaminase 2 [Source:HGNC Symbol;Acc:HGNC:4433] | 12903 | -0.328 | -0.1958 | Yes |
| 166 | TUFM | "Tu translation elongation factor, mitochondrial [Source:HGNC Symbol;Acc:HGNC:12420]" | 12914 | -0.330 | -0.1888 | Yes |
| 167 | STARD7 | StAR related lipid transfer domain containing 7 [Source:HGNC Symbol;Acc:HGNC:18063] | 12916 | -0.330 | -0.1813 | Yes |
| 168 | RRP9 | "ribosomal RNA processing 9, U3 small nucleolar RNA binding protein [Source:HGNC Symbol;Acc:HGNC:16829]" | 12955 | -0.334 | -0.1760 | Yes |
| 169 | SLC25A3 | solute carrier family 25 member 3 [Source:HGNC Symbol;Acc:HGNC:10989] | 13002 | -0.338 | -0.1711 | Yes |
| 170 | NOP56 | NOP56 ribonucleoprotein [Source:HGNC Symbol;Acc:HGNC:15911] | 13004 | -0.338 | -0.1634 | Yes |
| 171 | EIF3B | eukaryotic translation initiation factor 3 subunit B [Source:HGNC Symbol;Acc:HGNC:3280] | 13011 | -0.338 | -0.1559 | Yes |
| 172 | LDHA | lactate dehydrogenase A [Source:HGNC Symbol;Acc:HGNC:6535] | 13197 | -0.356 | -0.1597 | Yes |
| 173 | PRPS2 | phosphoribosyl pyrophosphate synthetase 2 [Source:HGNC Symbol;Acc:HGNC:9465] | 13205 | -0.356 | -0.1519 | Yes |
| 174 | SNRPA | small nuclear ribonucleoprotein polypeptide A [Source:HGNC Symbol;Acc:HGNC:11151] | 13284 | -0.365 | -0.1484 | Yes |
| 175 | RPL22 | ribosomal protein L22 [Source:HGNC Symbol;Acc:HGNC:10315] | 13294 | -0.366 | -0.1405 | Yes |
| 176 | TCP1 | t-complex 1 [Source:HGNC Symbol;Acc:HGNC:11655] | 13396 | -0.379 | -0.1383 | Yes |
| 177 | PCBP1 | poly(rC) binding protein 1 [Source:HGNC Symbol;Acc:HGNC:8647] | 13419 | -0.381 | -0.1309 | Yes |
| 178 | POLD2 | "DNA polymerase delta 2, accessory subunit [Source:HGNC Symbol;Acc:HGNC:9176]" | 13550 | -0.398 | -0.1301 | Yes |
| 179 | CDK4 | cyclin dependent kinase 4 [Source:HGNC Symbol;Acc:HGNC:1773] | 13588 | -0.403 | -0.1232 | Yes |
| 180 | CCT3 | chaperonin containing TCP1 subunit 3 [Source:HGNC Symbol;Acc:HGNC:1616] | 13635 | -0.409 | -0.1167 | Yes |
| 181 | HDDC2 | HD domain containing 2 [Source:HGNC Symbol;Acc:HGNC:21078] | 13861 | -0.425 | -0.1214 | Yes |
| 182 | RPS2 | ribosomal protein S2 [Source:HGNC Symbol;Acc:HGNC:10404] | 14019 | -0.435 | -0.1214 | Yes |
| 183 | SNRPD2 | small nuclear ribonucleoprotein D2 polypeptide [Source:HGNC Symbol;Acc:HGNC:11159] | 14096 | -0.448 | -0.1160 | Yes |
| 184 | RPL14 | ribosomal protein L14 [Source:HGNC Symbol;Acc:HGNC:10305] | 14121 | -0.453 | -0.1070 | Yes |
| 185 | PGK1 | phosphoglycerate kinase 1 [Source:HGNC Symbol;Acc:HGNC:8896] | 14157 | -0.458 | -0.0987 | Yes |
| 186 | RPL6 | ribosomal protein L6 [Source:HGNC Symbol;Acc:HGNC:10362] | 14230 | -0.469 | -0.0925 | Yes |
| 187 | RPS3 | ribosomal protein S3 [Source:HGNC Symbol;Acc:HGNC:10420] | 14348 | -0.486 | -0.0888 | Yes |
| 188 | RACK1 | receptor for activated C kinase 1 [Source:HGNC Symbol;Acc:HGNC:4399] | 14370 | -0.489 | -0.0788 | Yes |
| 189 | NOLC1 | nucleolar and coiled-body phosphoprotein 1 [Source:HGNC Symbol;Acc:HGNC:15608] | 14380 | -0.490 | -0.0681 | Yes |
| 190 | RPS6 | ribosomal protein S6 [Source:HGNC Symbol;Acc:HGNC:10429] | 14506 | -0.515 | -0.0642 | Yes |
| 191 | IMPDH2 | inosine monophosphate dehydrogenase 2 [Source:HGNC Symbol;Acc:HGNC:6053] | 14589 | -0.530 | -0.0573 | Yes |
| 192 | GLO1 | glyoxalase I [Source:HGNC Symbol;Acc:HGNC:4323] | 14603 | -0.532 | -0.0458 | Yes |
| 193 | RPS5 | ribosomal protein S5 [Source:HGNC Symbol;Acc:HGNC:10426] | 14664 | -0.546 | -0.0370 | Yes |
| 194 | EEF1B2 | eukaryotic translation elongation factor 1 beta 2 [Source:HGNC Symbol;Acc:HGNC:3208] | 14753 | -0.568 | -0.0296 | Yes |
| 195 | MRPL23 | mitochondrial ribosomal protein L23 [Source:HGNC Symbol;Acc:HGNC:10322] | 14796 | -0.582 | -0.0188 | Yes |
| 196 | PABPC4 | poly(A) binding protein cytoplasmic 4 [Source:HGNC Symbol;Acc:HGNC:8557] | 14798 | -0.582 | -0.0054 | Yes |
| 197 | SRM | spermidine synthase [Source:HGNC Symbol;Acc:HGNC:11296] | 14841 | -0.596 | 0.0057 | Yes |
| 198 | RPL18 | ribosomal protein L18 [Source:HGNC Symbol;Acc:HGNC:10310] | 14911 | -0.624 | 0.0157 | Yes |
| 199 | PRDX4 | peroxiredoxin 4 [Source:HGNC Symbol;Acc:HGNC:17169] | 15089 | -0.713 | 0.0207 | Yes |
| 200 | RPLP0 | ribosomal protein lateral stalk subunit P0 [Source:HGNC Symbol;Acc:HGNC:10371] | 15100 | -0.718 | 0.0367 | Yes |
Table: GSEA details [plain text format]

  

Fig 2: HALLMARK\_MYC\_TARGETS\_V1      
 Blue-Pink O' Gram in the Space of the Analyzed GeneSet

  

Fig 3: HALLMARK\_MYC\_TARGETS\_V1: Random ES distribution      
 Gene set null distribution of ES for **HALLMARK\_MYC\_TARGETS\_V1**
