# Supplementary material for: In vivo CRISPR screens reveal Serpinb9 and Adam2 as regulators of immune therapy response in lung cancer
Source: Nat Commun. 2023 May 31;14:3150. doi: 10.1038/s41467-023-38841-7 (PMC10232477; doi:10.1038/s41467-023-38841-7)
Supplement: Supplementary file 13 — Source Data [file 41467_2023_38841_MOESM13_ESM.zip › Figure 4E.zip!/my_analysis.Gsea.1640146340255_Hallmark/HALLMARK_INTERFERON_GAMMA_RESPONSE.html]

Details for gene set HALLMARK\_INTERFERON\_GAMMA\_RESPONSE[GSEA]

|  || Dataset | December 21, 2021\_for GSEA run\_collapsed\_to\_symbols.GSEA\_B6\_NI.cls #CTLR\_NI\_versus\_ADAM2\_NI |
| Phenotype | GSEA\_B6\_NI.cls#CTLR\_NI\_versus\_ADAM2\_NI |
| Upregulated in class | CTLR\_NI |
| GeneSet | HALLMARK\_INTERFERON\_GAMMA\_RESPONSE |
| Enrichment Score (ES) | 0.7617147 |
| Normalized Enrichment Score (NES) | 1.5732398 |
| Nominal p-value | 0.0 |
| FDR q-value | 0.24780194 |
| FWER p-Value | 0.336 |
Table: GSEA Results Summary

  

Fig 1: Enrichment plot: HALLMARK\_INTERFERON\_GAMMA\_RESPONSE      
 Profile of the Running ES Score & Positions of GeneSet Members on the Rank Ordered List

  

| SYMBOL | TITLE | RANK IN GENE LIST | RANK METRIC SCORE | RUNNING ES | CORE ENRICHMENT || 1 | CXCL11 | C-X-C motif chemokine ligand 11 [Source:HGNC Symbol;Acc:HGNC:10638] | 2 | 3.216 | 0.0197 | Yes |
| 2 | APOL6 | apolipoprotein L6 [Source:HGNC Symbol;Acc:HGNC:14870] | 5 | 2.971 | 0.0380 | Yes |
| 3 | DDX60 | DExD/H-box helicase 60 [Source:HGNC Symbol;Acc:HGNC:25942] | 21 | 2.435 | 0.0520 | Yes |
| 4 | FGL2 | fibrinogen like 2 [Source:HGNC Symbol;Acc:HGNC:3696] | 44 | 2.152 | 0.0639 | Yes |
| 5 | STAT1 | signal transducer and activator of transcription 1 [Source:HGNC Symbol;Acc:HGNC:11362] | 45 | 2.090 | 0.0768 | Yes |
| 6 | IL18BP | interleukin 18 binding protein [Source:HGNC Symbol;Acc:HGNC:5987] | 52 | 2.005 | 0.0888 | Yes |
| 7 | DHX58 | DExH-box helicase 58 [Source:HGNC Symbol;Acc:HGNC:29517] | 55 | 1.981 | 0.1009 | Yes |
| 8 | XAF1 | XIAP associated factor 1 [Source:HGNC Symbol;Acc:HGNC:30932] | 59 | 1.960 | 0.1128 | Yes |
| 9 | RTP4 | receptor transporter protein 4 [Source:HGNC Symbol;Acc:HGNC:23992] | 80 | 1.848 | 0.1230 | Yes |
| 10 | SLAMF7 | SLAM family member 7 [Source:HGNC Symbol;Acc:HGNC:21394] | 85 | 1.832 | 0.1340 | Yes |
| 11 | STAT2 | signal transducer and activator of transcription 2 [Source:HGNC Symbol;Acc:HGNC:11363] | 86 | 1.830 | 0.1453 | Yes |
| 12 | PSMB9 | proteasome 20S subunit beta 9 [Source:HGNC Symbol;Acc:HGNC:9546] | 87 | 1.828 | 0.1566 | Yes |
| 13 | GBP6 | guanylate binding protein family member 6 [Source:HGNC Symbol;Acc:HGNC:25395] | 88 | 1.824 | 0.1679 | Yes |
| 14 | SP110 | SP110 nuclear body protein [Source:HGNC Symbol;Acc:HGNC:5401] | 89 | 1.822 | 0.1792 | Yes |
| 15 | CD274 | CD274 molecule [Source:HGNC Symbol;Acc:HGNC:17635] | 90 | 1.821 | 0.1904 | Yes |
| 16 | IRF4 | interferon regulatory factor 4 [Source:HGNC Symbol;Acc:HGNC:6119] | 93 | 1.799 | 0.2014 | Yes |
| 17 | UBE2L6 | ubiquitin conjugating enzyme E2 L6 [Source:HGNC Symbol;Acc:HGNC:12490] | 96 | 1.793 | 0.2123 | Yes |
| 18 | OAS3 | 2'-5'-oligoadenylate synthetase 3 [Source:HGNC Symbol;Acc:HGNC:8088] | 107 | 1.774 | 0.2226 | Yes |
| 19 | GBP4 | guanylate binding protein 4 [Source:HGNC Symbol;Acc:HGNC:20480] | 111 | 1.765 | 0.2334 | Yes |
| 20 | PARP14 | poly(ADP-ribose) polymerase family member 14 [Source:HGNC Symbol;Acc:HGNC:29232] | 124 | 1.728 | 0.2433 | Yes |
| 21 | TNFSF10 | TNF superfamily member 10 [Source:HGNC Symbol;Acc:HGNC:11925] | 125 | 1.728 | 0.2539 | Yes |
| 22 | IRF9 | interferon regulatory factor 9 [Source:HGNC Symbol;Acc:HGNC:6131] | 128 | 1.701 | 0.2643 | Yes |
| 23 | SECTM1 | secreted and transmembrane 1 [Source:HGNC Symbol;Acc:HGNC:10707] | 137 | 1.682 | 0.2742 | Yes |
| 24 | IL2RB | interleukin 2 receptor subunit beta [Source:HGNC Symbol;Acc:HGNC:6009] | 138 | 1.679 | 0.2846 | Yes |
| 25 | CXCL10 | C-X-C motif chemokine ligand 10 [Source:HGNC Symbol;Acc:HGNC:10637] | 142 | 1.666 | 0.2947 | Yes |
| 26 | GPR18 | G protein-coupled receptor 18 [Source:HGNC Symbol;Acc:HGNC:4472] | 143 | 1.664 | 0.3049 | Yes |
| 27 | ISG15 | ISG15 ubiquitin like modifier [Source:HGNC Symbol;Acc:HGNC:4053] | 144 | 1.658 | 0.3152 | Yes |
| 28 | IDO1 | "indoleamine 2,3-dioxygenase 1 [Source:HGNC Symbol;Acc:HGNC:6059]" | 148 | 1.654 | 0.3252 | Yes |
| 29 | HERC6 | HECT and RLD domain containing E3 ubiquitin protein ligase family member 6 [Source:HGNC Symbol;Acc:HGNC:26072] | 155 | 1.628 | 0.3349 | Yes |
| 30 | BATF2 | basic leucine zipper ATF-like transcription factor 2 [Source:HGNC Symbol;Acc:HGNC:25163] | 159 | 1.621 | 0.3447 | Yes |
| 31 | NLRC5 | NLR family CARD domain containing 5 [Source:HGNC Symbol;Acc:HGNC:29933] | 165 | 1.607 | 0.3543 | Yes |
| 32 | CCL5 | C-C motif chemokine ligand 5 [Source:HGNC Symbol;Acc:HGNC:10632] | 169 | 1.591 | 0.3639 | Yes |
| 33 | IFIH1 | interferon induced with helicase C domain 1 [Source:HGNC Symbol;Acc:HGNC:18873] | 183 | 1.531 | 0.3725 | Yes |
| 34 | STAT4 | signal transducer and activator of transcription 4 [Source:HGNC Symbol;Acc:HGNC:11365] | 186 | 1.521 | 0.3818 | Yes |
| 35 | FCGR1A | Fc fragment of IgG receptor Ia [Source:HGNC Symbol;Acc:HGNC:3613] | 187 | 1.518 | 0.3912 | Yes |
| 36 | UPP1 | uridine phosphorylase 1 [Source:HGNC Symbol;Acc:HGNC:12576] | 191 | 1.509 | 0.4003 | Yes |
| 37 | OASL | 2'-5'-oligoadenylate synthetase like [Source:HGNC Symbol;Acc:HGNC:8090] | 214 | 1.455 | 0.4079 | Yes |
| 38 | CMPK2 | cytidine/uridine monophosphate kinase 2 [Source:HGNC Symbol;Acc:HGNC:27015] | 227 | 1.427 | 0.4159 | Yes |
| 39 | IFIT2 | interferon induced protein with tetratricopeptide repeats 2 [Source:HGNC Symbol;Acc:HGNC:5409] | 235 | 1.400 | 0.4241 | Yes |
| 40 | TAP1 | "transporter 1, ATP binding cassette subfamily B member [Source:HGNC Symbol;Acc:HGNC:43]" | 241 | 1.389 | 0.4324 | Yes |
| 41 | CFB | complement factor B [Source:HGNC Symbol;Acc:HGNC:1037] | 245 | 1.387 | 0.4408 | Yes |
| 42 | CASP1 | caspase 1 [Source:HGNC Symbol;Acc:HGNC:1499] | 248 | 1.383 | 0.4492 | Yes |
| 43 | LGALS3BP | galectin 3 binding protein [Source:HGNC Symbol;Acc:HGNC:6564] | 263 | 1.359 | 0.4567 | Yes |
| 44 | TRIM14 | tripartite motif containing 14 [Source:HGNC Symbol;Acc:HGNC:16283] | 265 | 1.353 | 0.4650 | Yes |
| 45 | TRIM21 | tripartite motif containing 21 [Source:HGNC Symbol;Acc:HGNC:11312] | 277 | 1.338 | 0.4725 | Yes |
| 46 | IFI44 | interferon induced protein 44 [Source:HGNC Symbol;Acc:HGNC:16938] | 278 | 1.338 | 0.4808 | Yes |
| 47 | USP18 | ubiquitin specific peptidase 18 [Source:HGNC Symbol;Acc:HGNC:12616] | 290 | 1.321 | 0.4882 | Yes |
| 48 | IRF1 | interferon regulatory factor 1 [Source:HGNC Symbol;Acc:HGNC:6116] | 295 | 1.310 | 0.4961 | Yes |
| 49 | BST2 | bone marrow stromal cell antigen 2 [Source:HGNC Symbol;Acc:HGNC:1119] | 306 | 1.293 | 0.5034 | Yes |
| 50 | IFIT3 | interferon induced protein with tetratricopeptide repeats 3 [Source:HGNC Symbol;Acc:HGNC:5411] | 314 | 1.278 | 0.5109 | Yes |
| 51 | PARP12 | poly(ADP-ribose) polymerase family member 12 [Source:HGNC Symbol;Acc:HGNC:21919] | 315 | 1.276 | 0.5187 | Yes |
| 52 | MX1 | MX dynamin like GTPase 1 [Source:HGNC Symbol;Acc:HGNC:7532] | 316 | 1.272 | 0.5266 | Yes |
| 53 | PSMB10 | proteasome 20S subunit beta 10 [Source:HGNC Symbol;Acc:HGNC:9538] | 326 | 1.261 | 0.5338 | Yes |
| 54 | EPSTI1 | epithelial stromal interaction 1 [Source:HGNC Symbol;Acc:HGNC:16465] | 346 | 1.240 | 0.5402 | Yes |
| 55 | RSAD2 | radical S-adenosyl methionine domain containing 2 [Source:HGNC Symbol;Acc:HGNC:30908] | 354 | 1.225 | 0.5474 | Yes |
| 56 | CASP4 | caspase 4 [Source:HGNC Symbol;Acc:HGNC:1505] | 358 | 1.219 | 0.5547 | Yes |
| 57 | IRF7 | interferon regulatory factor 7 [Source:HGNC Symbol;Acc:HGNC:6122] | 359 | 1.219 | 0.5622 | Yes |
| 58 | SAMHD1 | SAM and HD domain containing deoxynucleoside triphosphate triphosphohydrolase 1 [Source:HGNC Symbol;Acc:HGNC:15925] | 362 | 1.214 | 0.5696 | Yes |
| 59 | HLA-B | "major histocompatibility complex, class I, B [Source:HGNC Symbol;Acc:HGNC:4932]" | 371 | 1.206 | 0.5765 | Yes |
| 60 | IL6 | interleukin 6 [Source:HGNC Symbol;Acc:HGNC:6018] | 378 | 1.200 | 0.5835 | Yes |
| 61 | SAMD9L | sterile alpha motif domain containing 9 like [Source:HGNC Symbol;Acc:HGNC:1349] | 385 | 1.195 | 0.5905 | Yes |
| 62 | ZBP1 | Z-DNA binding protein 1 [Source:HGNC Symbol;Acc:HGNC:16176] | 393 | 1.181 | 0.5974 | Yes |
| 63 | RNF213 | ring finger protein 213 [Source:HGNC Symbol;Acc:HGNC:14539] | 407 | 1.162 | 0.6037 | Yes |
| 64 | FAS | Fas cell surface death receptor [Source:HGNC Symbol;Acc:HGNC:11920] | 418 | 1.154 | 0.6102 | Yes |
| 65 | B2M | beta-2-microglobulin [Source:HGNC Symbol;Acc:HGNC:914] | 441 | 1.131 | 0.6158 | Yes |
| 66 | PSMB8 | proteasome 20S subunit beta 8 [Source:HGNC Symbol;Acc:HGNC:9545] | 467 | 1.101 | 0.6210 | Yes |
| 67 | EIF2AK2 | eukaryotic translation initiation factor 2 alpha kinase 2 [Source:HGNC Symbol;Acc:HGNC:9437] | 472 | 1.098 | 0.6275 | Yes |
| 68 | CXCL9 | C-X-C motif chemokine ligand 9 [Source:HGNC Symbol;Acc:HGNC:7098] | 473 | 1.098 | 0.6343 | Yes |
| 69 | CD69 | CD69 molecule [Source:HGNC Symbol;Acc:HGNC:1694] | 476 | 1.094 | 0.6409 | Yes |
| 70 | IL15RA | interleukin 15 receptor subunit alpha [Source:HGNC Symbol;Acc:HGNC:5978] | 494 | 1.080 | 0.6465 | Yes |
| 71 | PSME2 | proteasome activator subunit 2 [Source:HGNC Symbol;Acc:HGNC:9569] | 507 | 1.064 | 0.6523 | Yes |
| 72 | CD86 | CD86 molecule [Source:HGNC Symbol;Acc:HGNC:1705] | 518 | 1.048 | 0.6581 | Yes |
| 73 | NOD1 | nucleotide binding oligomerization domain containing 1 [Source:HGNC Symbol;Acc:HGNC:16390] | 536 | 1.034 | 0.6634 | Yes |
| 74 | IRF5 | interferon regulatory factor 5 [Source:HGNC Symbol;Acc:HGNC:6120] | 539 | 1.032 | 0.6696 | Yes |
| 75 | DDX58 | DExD/H-box helicase 58 [Source:HGNC Symbol;Acc:HGNC:19102] | 544 | 1.031 | 0.6757 | Yes |
| 76 | OAS2 | 2'-5'-oligoadenylate synthetase 2 [Source:HGNC Symbol;Acc:HGNC:8087] | 560 | 1.020 | 0.6811 | Yes |
| 77 | CSF2RB | colony stimulating factor 2 receptor subunit beta [Source:HGNC Symbol;Acc:HGNC:2436] | 561 | 1.019 | 0.6874 | Yes |
| 78 | PSME1 | proteasome activator subunit 1 [Source:HGNC Symbol;Acc:HGNC:9568] | 569 | 1.013 | 0.6932 | Yes |
| 79 | NMI | N-myc and STAT interactor [Source:HGNC Symbol;Acc:HGNC:7854] | 579 | 1.008 | 0.6988 | Yes |
| 80 | TRAFD1 | TRAF-type zinc finger domain containing 1 [Source:HGNC Symbol;Acc:HGNC:24808] | 598 | 0.998 | 0.7038 | Yes |
| 81 | LCP2 | lymphocyte cytosolic protein 2 [Source:HGNC Symbol;Acc:HGNC:6529] | 727 | 0.918 | 0.7012 | Yes |
| 82 | RNF31 | ring finger protein 31 [Source:HGNC Symbol;Acc:HGNC:16031] | 772 | 0.891 | 0.7039 | Yes |
| 83 | ZNFX1 | zinc finger NFX1-type containing 1 [Source:HGNC Symbol;Acc:HGNC:29271] | 878 | 0.848 | 0.7023 | Yes |
| 84 | PNP | purine nucleoside phosphorylase [Source:HGNC Symbol;Acc:HGNC:7892] | 902 | 0.841 | 0.7061 | Yes |
| 85 | IFI35 | interferon induced protein 35 [Source:HGNC Symbol;Acc:HGNC:5399] | 998 | 0.820 | 0.7050 | Yes |
| 86 | CASP7 | caspase 7 [Source:HGNC Symbol;Acc:HGNC:1508] | 1011 | 0.810 | 0.7092 | Yes |
| 87 | PTPN6 | protein tyrosine phosphatase non-receptor type 6 [Source:HGNC Symbol;Acc:HGNC:9658] | 1049 | 0.790 | 0.7117 | Yes |
| 88 | IL10RA | interleukin 10 receptor subunit alpha [Source:HGNC Symbol;Acc:HGNC:5964] | 1082 | 0.778 | 0.7144 | Yes |
| 89 | ICAM1 | intercellular adhesion molecule 1 [Source:HGNC Symbol;Acc:HGNC:5344] | 1112 | 0.767 | 0.7173 | Yes |
| 90 | GZMA | granzyme A [Source:HGNC Symbol;Acc:HGNC:4708] | 1113 | 0.766 | 0.7220 | Yes |
| 91 | HLA-A | "major histocompatibility complex, class I, A [Source:HGNC Symbol;Acc:HGNC:4931]" | 1127 | 0.761 | 0.7259 | Yes |
| 92 | NAMPT | nicotinamide phosphoribosyltransferase [Source:HGNC Symbol;Acc:HGNC:30092] | 1163 | 0.744 | 0.7282 | Yes |
| 93 | SERPING1 | serpin family G member 1 [Source:HGNC Symbol;Acc:HGNC:1228] | 1209 | 0.728 | 0.7298 | Yes |
| 94 | HLA-DMA | "major histocompatibility complex, class II, DM alpha [Source:HGNC Symbol;Acc:HGNC:4934]" | 1238 | 0.719 | 0.7324 | Yes |
| 95 | CIITA | class II major histocompatibility complex transactivator [Source:HGNC Symbol;Acc:HGNC:7067] | 1242 | 0.718 | 0.7367 | Yes |
| 96 | CD40 | CD40 molecule [Source:HGNC Symbol;Acc:HGNC:11919] | 1287 | 0.700 | 0.7382 | Yes |
| 97 | SSPN | sarcospan [Source:HGNC Symbol;Acc:HGNC:11322] | 1331 | 0.684 | 0.7396 | Yes |
| 98 | MARCHF1 | membrane associated ring-CH-type finger 1 [Source:HGNC Symbol;Acc:HGNC:26077] | 1333 | 0.684 | 0.7438 | Yes |
| 99 | SOCS1 | suppressor of cytokine signaling 1 [Source:HGNC Symbol;Acc:HGNC:19383] | 1359 | 0.674 | 0.7463 | Yes |
| 100 | IRF8 | interferon regulatory factor 8 [Source:HGNC Symbol;Acc:HGNC:5358] | 1378 | 0.667 | 0.7493 | Yes |
| 101 | TAPBP | TAP binding protein [Source:HGNC Symbol;Acc:HGNC:11566] | 1384 | 0.665 | 0.7531 | Yes |
| 102 | IFI27 | interferon alpha inducible protein 27 [Source:HGNC Symbol;Acc:HGNC:5397] | 1395 | 0.662 | 0.7565 | Yes |
| 103 | HELZ2 | helicase with zinc finger 2 [Source:HGNC Symbol;Acc:HGNC:30021] | 1403 | 0.659 | 0.7601 | Yes |
| 104 | HLA-DQA1 | "major histocompatibility complex, class II, DQ alpha 1 [Source:HGNC Symbol;Acc:HGNC:4942]" | 1442 | 0.645 | 0.7617 | Yes |
| 105 | PELI1 | pellino E3 ubiquitin protein ligase 1 [Source:HGNC Symbol;Acc:HGNC:8827] | 1532 | 0.614 | 0.7597 | Yes |
| 106 | PIM1 | "Pim-1 proto-oncogene, serine/threonine kinase [Source:HGNC Symbol;Acc:HGNC:8986]" | 1567 | 0.607 | 0.7612 | Yes |
| 107 | CD74 | CD74 molecule [Source:HGNC Symbol;Acc:HGNC:1697] | 1682 | 0.573 | 0.7574 | Yes |
| 108 | TNFAIP3 | TNF alpha induced protein 3 [Source:HGNC Symbol;Acc:HGNC:11896] | 1713 | 0.564 | 0.7590 | Yes |
| 109 | TRIM25 | tripartite motif containing 25 [Source:HGNC Symbol;Acc:HGNC:12932] | 1725 | 0.560 | 0.7617 | Yes |
| 110 | IFITM3 | interferon induced transmembrane protein 3 [Source:HGNC Symbol;Acc:HGNC:5414] | 1928 | 0.517 | 0.7519 | No |
| 111 | C1R | complement C1r [Source:HGNC Symbol;Acc:HGNC:1246] | 2046 | 0.499 | 0.7474 | No |
| 112 | HLA-DRB1 | "major histocompatibility complex, class II, DR beta 1 [Source:HGNC Symbol;Acc:HGNC:4948]" | 2055 | 0.498 | 0.7499 | No |
| 113 | IFNAR2 | interferon alpha and beta receptor subunit 2 [Source:HGNC Symbol;Acc:HGNC:5433] | 2095 | 0.493 | 0.7505 | No |
| 114 | CMTR1 | cap methyltransferase 1 [Source:HGNC Symbol;Acc:HGNC:21077] | 2326 | 0.465 | 0.7385 | No |
| 115 | ISG20 | interferon stimulated exonuclease gene 20 [Source:HGNC Symbol;Acc:HGNC:6130] | 2346 | 0.460 | 0.7401 | No |
| 116 | FPR1 | formyl peptide receptor 1 [Source:HGNC Symbol;Acc:HGNC:3826] | 2392 | 0.452 | 0.7400 | No |
| 117 | SPPL2A | signal peptide peptidase like 2A [Source:HGNC Symbol;Acc:HGNC:30227] | 2404 | 0.451 | 0.7420 | No |
| 118 | ADAR | adenosine deaminase RNA specific [Source:HGNC Symbol;Acc:HGNC:225] | 2405 | 0.451 | 0.7448 | No |
| 119 | LATS2 | large tumor suppressor kinase 2 [Source:HGNC Symbol;Acc:HGNC:6515] | 2407 | 0.450 | 0.7475 | No |
| 120 | CASP8 | caspase 8 [Source:HGNC Symbol;Acc:HGNC:1509] | 2434 | 0.446 | 0.7486 | No |
| 121 | GCH1 | GTP cyclohydrolase 1 [Source:HGNC Symbol;Acc:HGNC:4193] | 2566 | 0.441 | 0.7429 | No |
| 122 | IL15 | interleukin 15 [Source:HGNC Symbol;Acc:HGNC:5977] | 2791 | 0.418 | 0.7310 | No |
| 123 | NFKBIA | NFKB inhibitor alpha [Source:HGNC Symbol;Acc:HGNC:7797] | 2954 | 0.411 | 0.7231 | No |
| 124 | CCL7 | C-C motif chemokine ligand 7 [Source:HGNC Symbol;Acc:HGNC:10634] | 2997 | 0.404 | 0.7228 | No |
| 125 | IRF2 | interferon regulatory factor 2 [Source:HGNC Symbol;Acc:HGNC:6117] | 3080 | 0.391 | 0.7200 | No |
| 126 | MYD88 | MYD88 innate immune signal transduction adaptor [Source:HGNC Symbol;Acc:HGNC:7562] | 3202 | 0.373 | 0.7144 | No |
| 127 | IL4R | interleukin 4 receptor [Source:HGNC Symbol;Acc:HGNC:6015] | 3233 | 0.368 | 0.7148 | No |
| 128 | SOCS3 | suppressor of cytokine signaling 3 [Source:HGNC Symbol;Acc:HGNC:19391] | 3330 | 0.352 | 0.7108 | No |
| 129 | PSMA3 | proteasome 20S subunit alpha 3 [Source:HGNC Symbol;Acc:HGNC:9532] | 3335 | 0.352 | 0.7127 | No |
| 130 | VAMP8 | vesicle associated membrane protein 8 [Source:HGNC Symbol;Acc:HGNC:12647] | 3355 | 0.349 | 0.7136 | No |
| 131 | PLA2G4A | phospholipase A2 group IVA [Source:HGNC Symbol;Acc:HGNC:9035] | 3396 | 0.342 | 0.7131 | No |
| 132 | AUTS2 | activator of transcription and developmental regulator AUTS2 [Source:HGNC Symbol;Acc:HGNC:14262] | 3442 | 0.336 | 0.7123 | No |
| 133 | IFI30 | IFI30 lysosomal thiol reductase [Source:HGNC Symbol;Acc:HGNC:5398] | 3485 | 0.330 | 0.7116 | No |
| 134 | C1S | complement C1s [Source:HGNC Symbol;Acc:HGNC:1247] | 3521 | 0.324 | 0.7113 | No |
| 135 | CCL2 | C-C motif chemokine ligand 2 [Source:HGNC Symbol;Acc:HGNC:10618] | 3622 | 0.312 | 0.7068 | No |
| 136 | JAK2 | Janus kinase 2 [Source:HGNC Symbol;Acc:HGNC:6192] | 3644 | 0.308 | 0.7074 | No |
| 137 | METTL7B | methyltransferase like 7B [Source:HGNC Symbol;Acc:HGNC:28276] | 3720 | 0.300 | 0.7044 | No |
| 138 | ITGB7 | integrin subunit beta 7 [Source:HGNC Symbol;Acc:HGNC:6162] | 3723 | 0.300 | 0.7061 | No |
| 139 | P2RY14 | purinergic receptor P2Y14 [Source:HGNC Symbol;Acc:HGNC:16442] | 3761 | 0.295 | 0.7055 | No |
| 140 | ARL4A | ADP ribosylation factor like GTPase 4A [Source:HGNC Symbol;Acc:HGNC:695] | 3817 | 0.289 | 0.7038 | No |
| 141 | CMKLR1 | chemerin chemokine-like receptor 1 [Source:HGNC Symbol;Acc:HGNC:2121] | 3919 | 0.276 | 0.6989 | No |
| 142 | TNFAIP2 | TNF alpha induced protein 2 [Source:HGNC Symbol;Acc:HGNC:11895] | 3997 | 0.266 | 0.6956 | No |
| 143 | IL7 | interleukin 7 [Source:HGNC Symbol;Acc:HGNC:6023] | 4006 | 0.265 | 0.6967 | No |
| 144 | ST3GAL5 | "ST3 beta-galactoside alpha-2,3-sialyltransferase 5 [Source:HGNC Symbol;Acc:HGNC:10872]" | 4104 | 0.254 | 0.6920 | No |
| 145 | HIF1A | hypoxia inducible factor 1 subunit alpha [Source:HGNC Symbol;Acc:HGNC:4910] | 4178 | 0.246 | 0.6888 | No |
| 146 | PNPT1 | polyribonucleotide nucleotidyltransferase 1 [Source:HGNC Symbol;Acc:HGNC:23166] | 4303 | 0.232 | 0.6822 | No |
| 147 | ST8SIA4 | "ST8 alpha-N-acetyl-neuraminide alpha-2,8-sialyltransferase 4 [Source:HGNC Symbol;Acc:HGNC:10871]" | 4356 | 0.227 | 0.6803 | No |
| 148 | TDRD7 | tudor domain containing 7 [Source:HGNC Symbol;Acc:HGNC:30831] | 4400 | 0.222 | 0.6789 | No |
| 149 | EIF4E3 | eukaryotic translation initiation factor 4E family member 3 [Source:HGNC Symbol;Acc:HGNC:31837] | 4426 | 0.220 | 0.6786 | No |
| 150 | NUP93 | nucleoporin 93 [Source:HGNC Symbol;Acc:HGNC:28958] | 4476 | 0.215 | 0.6768 | No |
| 151 | NCOA3 | nuclear receptor coactivator 3 [Source:HGNC Symbol;Acc:HGNC:7670] | 4525 | 0.211 | 0.6750 | No |
| 152 | VAMP5 | vesicle associated membrane protein 5 [Source:HGNC Symbol;Acc:HGNC:12646] | 4557 | 0.209 | 0.6743 | No |
| 153 | STAT3 | signal transducer and activator of transcription 3 [Source:HGNC Symbol;Acc:HGNC:11364] | 4571 | 0.207 | 0.6747 | No |
| 154 | PFKP | "phosphofructokinase, platelet [Source:HGNC Symbol;Acc:HGNC:8878]" | 4579 | 0.206 | 0.6755 | No |
| 155 | PSMA2 | proteasome 20S subunit alpha 2 [Source:HGNC Symbol;Acc:HGNC:9531] | 4594 | 0.205 | 0.6759 | No |
| 156 | TRIM26 | tripartite motif containing 26 [Source:HGNC Symbol;Acc:HGNC:12962] | 4650 | 0.201 | 0.6736 | No |
| 157 | RIPK1 | receptor interacting serine/threonine kinase 1 [Source:HGNC Symbol;Acc:HGNC:10019] | 4756 | 0.192 | 0.6680 | No |
| 158 | LYSMD2 | LysM domain containing 2 [Source:HGNC Symbol;Acc:HGNC:28571] | 4821 | 0.186 | 0.6650 | No |
| 159 | PTPN2 | protein tyrosine phosphatase non-receptor type 2 [Source:HGNC Symbol;Acc:HGNC:9650] | 5042 | 0.164 | 0.6518 | No |
| 160 | CD38 | CD38 molecule [Source:HGNC Symbol;Acc:HGNC:1667] | 5197 | 0.151 | 0.6428 | No |
| 161 | PTPN1 | protein tyrosine phosphatase non-receptor type 1 [Source:HGNC Symbol;Acc:HGNC:9642] | 5216 | 0.150 | 0.6425 | No |
| 162 | PDE4B | phosphodiesterase 4B [Source:HGNC Symbol;Acc:HGNC:8781] | 5633 | 0.116 | 0.6164 | No |
| 163 | RIPK2 | receptor interacting serine/threonine kinase 2 [Source:HGNC Symbol;Acc:HGNC:10020] | 5692 | 0.113 | 0.6133 | No |
| 164 | TXNIP | thioredoxin interacting protein [Source:HGNC Symbol;Acc:HGNC:16952] | 5781 | 0.106 | 0.6083 | No |
| 165 | RAPGEF6 | Rap guanine nucleotide exchange factor 6 [Source:HGNC Symbol;Acc:HGNC:20655] | 6082 | 0.084 | 0.5894 | No |
| 166 | SLC25A28 | solute carrier family 25 member 28 [Source:HGNC Symbol;Acc:HGNC:23472] | 6127 | 0.081 | 0.5871 | No |
| 167 | VCAM1 | vascular cell adhesion molecule 1 [Source:HGNC Symbol;Acc:HGNC:12663] | 6218 | 0.075 | 0.5817 | No |
| 168 | WARS1 | tryptophanyl-tRNA synthetase 1 [Source:HGNC Symbol;Acc:HGNC:12729] | 6325 | 0.068 | 0.5753 | No |
| 169 | XCL1 | X-C motif chemokine ligand 1 [Source:HGNC Symbol;Acc:HGNC:10645] | 6339 | 0.067 | 0.5749 | No |
| 170 | CASP3 | caspase 3 [Source:HGNC Symbol;Acc:HGNC:1504] | 6509 | 0.054 | 0.5643 | No |
| 171 | NFKB1 | nuclear factor kappa B subunit 1 [Source:HGNC Symbol;Acc:HGNC:7794] | 6748 | 0.040 | 0.5492 | No |
| 172 | PML | PML nuclear body scaffold [Source:HGNC Symbol;Acc:HGNC:9113] | 6860 | 0.032 | 0.5422 | No |
| 173 | RBCK1 | RANBP2-type and C3HC4-type zinc finger containing 1 [Source:HGNC Symbol;Acc:HGNC:15864] | 6883 | 0.030 | 0.5409 | No |
| 174 | PTGS2 | prostaglandin-endoperoxide synthase 2 [Source:HGNC Symbol;Acc:HGNC:9605] | 7157 | 0.015 | 0.5234 | No |
| 175 | PLSCR1 | phospholipid scramblase 1 [Source:HGNC Symbol;Acc:HGNC:9092] | 7180 | 0.013 | 0.5221 | No |
| 176 | LY6E | lymphocyte antigen 6 family member E [Source:HGNC Symbol;Acc:HGNC:6727] | 7188 | 0.013 | 0.5217 | No |
| 177 | ISOC1 | isochorismatase domain containing 1 [Source:HGNC Symbol;Acc:HGNC:24254] | 7415 | -0.000 | 0.5071 | No |
| 178 | OGFR | opioid growth factor receptor [Source:HGNC Symbol;Acc:HGNC:15768] | 7450 | -0.002 | 0.5049 | No |
| 179 | SRI | sorcin [Source:HGNC Symbol;Acc:HGNC:11292] | 7595 | -0.009 | 0.4956 | No |
| 180 | SOD2 | superoxide dismutase 2 [Source:HGNC Symbol;Acc:HGNC:11180] | 7867 | -0.022 | 0.4783 | No |
| 181 | MTHFD2 | "methylenetetrahydrofolate dehydrogenase (NADP+ dependent) 2, methenyltetrahydrofolate cyclohydrolase [Source:HGNC Symbol;Acc:HGNC:7434]" | 7932 | -0.025 | 0.4743 | No |
| 182 | LAP3 | leucine aminopeptidase 3 [Source:HGNC Symbol;Acc:HGNC:18449] | 8033 | -0.031 | 0.4680 | No |
| 183 | BTG1 | BTG anti-proliferation factor 1 [Source:HGNC Symbol;Acc:HGNC:1130] | 8137 | -0.037 | 0.4616 | No |
| 184 | ARID5B | AT-rich interaction domain 5B [Source:HGNC Symbol;Acc:HGNC:17362] | 8765 | -0.070 | 0.4215 | No |
| 185 | PSMB2 | proteasome 20S subunit beta 2 [Source:HGNC Symbol;Acc:HGNC:9539] | 8925 | -0.078 | 0.4117 | No |
| 186 | MVP | major vault protein [Source:HGNC Symbol;Acc:HGNC:7531] | 9551 | -0.114 | 0.3720 | No |
| 187 | IFITM2 | interferon induced transmembrane protein 2 [Source:HGNC Symbol;Acc:HGNC:5413] | 10288 | -0.159 | 0.3255 | No |
| 188 | BANK1 | B cell scaffold protein with ankyrin repeats 1 [Source:HGNC Symbol;Acc:HGNC:18233] | 10466 | -0.169 | 0.3151 | No |
| 189 | SELP | selectin P [Source:HGNC Symbol;Acc:HGNC:10721] | 11056 | -0.203 | 0.2783 | No |
| 190 | TOR1B | torsin family 1 member B [Source:HGNC Symbol;Acc:HGNC:11995] | 11057 | -0.203 | 0.2795 | No |
| 191 | BPGM | bisphosphoglycerate mutase [Source:HGNC Symbol;Acc:HGNC:1093] | 14058 | -0.441 | 0.0884 | No |
| 192 | CFH | complement factor H [Source:HGNC Symbol;Acc:HGNC:4883] | 15171 | -0.760 | 0.0212 | No |
| 193 | TNFAIP6 | TNF alpha induced protein 6 [Source:HGNC Symbol;Acc:HGNC:11898] | 15265 | -0.815 | 0.0202 | No |
| 194 | CDKN1A | cyclin dependent kinase inhibitor 1A [Source:HGNC Symbol;Acc:HGNC:1784] | 15428 | -0.929 | 0.0155 | No |
Table: GSEA details [plain text format]

  

Fig 2: HALLMARK\_INTERFERON\_GAMMA\_RESPONSE      
 Blue-Pink O' Gram in the Space of the Analyzed GeneSet

  

Fig 3: HALLMARK\_INTERFERON\_GAMMA\_RESPONSE: Random ES distribution      
 Gene set null distribution of ES for **HALLMARK\_INTERFERON\_GAMMA\_RESPONSE**
