# Supplementary material for: In vivo CRISPR screens reveal Serpinb9 and Adam2 as regulators of immune therapy response in lung cancer
Source: Nat Commun. 2023 May 31;14:3150. doi: 10.1038/s41467-023-38841-7 (PMC10232477; doi:10.1038/s41467-023-38841-7)
Supplement: Supplementary file 13 — Source Data [file 41467_2023_38841_MOESM13_ESM.zip › Figure 4E.zip!/my_analysis.Gsea.1640146340255_Hallmark/HALLMARK_IL2_STAT5_SIGNALING.html]

Details for gene set HALLMARK\_IL2\_STAT5\_SIGNALING[GSEA]

|  || Dataset | December 21, 2021\_for GSEA run\_collapsed\_to\_symbols.GSEA\_B6\_NI.cls #CTLR\_NI\_versus\_ADAM2\_NI |
| Phenotype | GSEA\_B6\_NI.cls#CTLR\_NI\_versus\_ADAM2\_NI |
| Upregulated in class | CTLR\_NI |
| GeneSet | HALLMARK\_IL2\_STAT5\_SIGNALING |
| Enrichment Score (ES) | 0.4710563 |
| Normalized Enrichment Score (NES) | 1.5558585 |
| Nominal p-value | 0.0 |
| FDR q-value | 0.15876293 |
| FWER p-Value | 0.336 |
Table: GSEA Results Summary

  

Fig 1: Enrichment plot: HALLMARK\_IL2\_STAT5\_SIGNALING      
 Profile of the Running ES Score & Positions of GeneSet Members on the Rank Ordered List

  

| SYMBOL | TITLE | RANK IN GENE LIST | RANK METRIC SCORE | RUNNING ES | CORE ENRICHMENT || 1 | ICOS | inducible T cell costimulator [Source:HGNC Symbol;Acc:HGNC:5351] | 3 | 3.209 | 0.0340 | Yes |
| 2 | FGL2 | fibrinogen like 2 [Source:HGNC Symbol;Acc:HGNC:3696] | 44 | 2.152 | 0.0543 | Yes |
| 3 | IRF4 | interferon regulatory factor 4 [Source:HGNC Symbol;Acc:HGNC:6119] | 93 | 1.799 | 0.0704 | Yes |
| 4 | GBP4 | guanylate binding protein 4 [Source:HGNC Symbol;Acc:HGNC:20480] | 111 | 1.765 | 0.0881 | Yes |
| 5 | TNFSF10 | TNF superfamily member 10 [Source:HGNC Symbol;Acc:HGNC:11925] | 125 | 1.728 | 0.1056 | Yes |
| 6 | IL2RB | interleukin 2 receptor subunit beta [Source:HGNC Symbol;Acc:HGNC:6009] | 138 | 1.679 | 0.1227 | Yes |
| 7 | CXCL10 | C-X-C motif chemokine ligand 10 [Source:HGNC Symbol;Acc:HGNC:10637] | 142 | 1.666 | 0.1403 | Yes |
| 8 | IL18R1 | interleukin 18 receptor 1 [Source:HGNC Symbol;Acc:HGNC:5988] | 179 | 1.535 | 0.1543 | Yes |
| 9 | MAP3K8 | mitogen-activated protein kinase kinase kinase 8 [Source:HGNC Symbol;Acc:HGNC:6860] | 202 | 1.482 | 0.1687 | Yes |
| 10 | IL2RA | interleukin 2 receptor subunit alpha [Source:HGNC Symbol;Acc:HGNC:6008] | 205 | 1.469 | 0.1842 | Yes |
| 11 | SELL | selectin L [Source:HGNC Symbol;Acc:HGNC:10720] | 206 | 1.467 | 0.1998 | Yes |
| 12 | IL10 | interleukin 10 [Source:HGNC Symbol;Acc:HGNC:5962] | 246 | 1.386 | 0.2121 | Yes |
| 13 | CTLA4 | cytotoxic T-lymphocyte associated protein 4 [Source:HGNC Symbol;Acc:HGNC:2505] | 275 | 1.340 | 0.2245 | Yes |
| 14 | TGM2 | transglutaminase 2 [Source:HGNC Symbol;Acc:HGNC:11778] | 305 | 1.293 | 0.2364 | Yes |
| 15 | IL1R2 | interleukin 1 receptor type 2 [Source:HGNC Symbol;Acc:HGNC:5994] | 318 | 1.270 | 0.2492 | Yes |
| 16 | CD79B | CD79b molecule [Source:HGNC Symbol;Acc:HGNC:1699] | 352 | 1.230 | 0.2601 | Yes |
| 17 | RHOH | ras homolog family member H [Source:HGNC Symbol;Acc:HGNC:686] | 413 | 1.159 | 0.2686 | Yes |
| 18 | CD86 | CD86 molecule [Source:HGNC Symbol;Acc:HGNC:1705] | 518 | 1.048 | 0.2731 | Yes |
| 19 | LTB | lymphotoxin beta [Source:HGNC Symbol;Acc:HGNC:6711] | 593 | 1.000 | 0.2789 | Yes |
| 20 | AGER | advanced glycosylation end-product specific receptor [Source:HGNC Symbol;Acc:HGNC:320] | 632 | 0.970 | 0.2868 | Yes |
| 21 | BATF | basic leucine zipper ATF-like transcription factor [Source:HGNC Symbol;Acc:HGNC:958] | 653 | 0.960 | 0.2957 | Yes |
| 22 | GADD45B | growth arrest and DNA damage inducible beta [Source:HGNC Symbol;Acc:HGNC:4096] | 667 | 0.954 | 0.3050 | Yes |
| 23 | PTH1R | parathyroid hormone 1 receptor [Source:HGNC Symbol;Acc:HGNC:9608] | 674 | 0.949 | 0.3148 | Yes |
| 24 | TNFRSF4 | TNF receptor superfamily member 4 [Source:HGNC Symbol;Acc:HGNC:11918] | 770 | 0.891 | 0.3181 | Yes |
| 25 | TNFRSF1B | TNF receptor superfamily member 1B [Source:HGNC Symbol;Acc:HGNC:11917] | 845 | 0.864 | 0.3225 | Yes |
| 26 | MXD1 | MAX dimerization protein 1 [Source:HGNC Symbol;Acc:HGNC:6761] | 898 | 0.842 | 0.3281 | Yes |
| 27 | PNP | purine nucleoside phosphorylase [Source:HGNC Symbol;Acc:HGNC:7892] | 902 | 0.841 | 0.3369 | Yes |
| 28 | GATA1 | GATA binding protein 1 [Source:HGNC Symbol;Acc:HGNC:4170] | 928 | 0.839 | 0.3442 | Yes |
| 29 | TLR7 | toll like receptor 7 [Source:HGNC Symbol;Acc:HGNC:15631] | 973 | 0.833 | 0.3503 | Yes |
| 30 | TNFRSF18 | TNF receptor superfamily member 18 [Source:HGNC Symbol;Acc:HGNC:11914] | 980 | 0.831 | 0.3587 | Yes |
| 31 | SMPDL3A | sphingomyelin phosphodiesterase acid like 3A [Source:HGNC Symbol;Acc:HGNC:17389] | 1005 | 0.812 | 0.3658 | Yes |
| 32 | TRAF1 | TNF receptor associated factor 1 [Source:HGNC Symbol;Acc:HGNC:12031] | 1077 | 0.781 | 0.3695 | Yes |
| 33 | IL10RA | interleukin 10 receptor subunit alpha [Source:HGNC Symbol;Acc:HGNC:5964] | 1082 | 0.778 | 0.3776 | Yes |
| 34 | NFKBIZ | NFKB inhibitor zeta [Source:HGNC Symbol;Acc:HGNC:29805] | 1093 | 0.773 | 0.3852 | Yes |
| 35 | CD48 | CD48 molecule [Source:HGNC Symbol;Acc:HGNC:1683] | 1137 | 0.756 | 0.3904 | Yes |
| 36 | ITGAE | integrin subunit alpha E [Source:HGNC Symbol;Acc:HGNC:6147] | 1146 | 0.752 | 0.3979 | Yes |
| 37 | RGS16 | regulator of G protein signaling 16 [Source:HGNC Symbol;Acc:HGNC:9997] | 1189 | 0.735 | 0.4030 | Yes |
| 38 | TTC39B | tetratricopeptide repeat domain 39B [Source:HGNC Symbol;Acc:HGNC:23704] | 1264 | 0.708 | 0.4058 | Yes |
| 39 | PTGER2 | prostaglandin E receptor 2 [Source:HGNC Symbol;Acc:HGNC:9594] | 1267 | 0.707 | 0.4132 | Yes |
| 40 | IKZF2 | IKAROS family zinc finger 2 [Source:HGNC Symbol;Acc:HGNC:13177] | 1312 | 0.693 | 0.4177 | Yes |
| 41 | TIAM1 | TIAM Rac1 associated GEF 1 [Source:HGNC Symbol;Acc:HGNC:11805] | 1317 | 0.689 | 0.4248 | Yes |
| 42 | SOCS1 | suppressor of cytokine signaling 1 [Source:HGNC Symbol;Acc:HGNC:19383] | 1359 | 0.674 | 0.4293 | Yes |
| 43 | IRF8 | interferon regulatory factor 8 [Source:HGNC Symbol;Acc:HGNC:5358] | 1378 | 0.667 | 0.4353 | Yes |
| 44 | IL1RL1 | interleukin 1 receptor like 1 [Source:HGNC Symbol;Acc:HGNC:5998] | 1475 | 0.635 | 0.4358 | Yes |
| 45 | ALCAM | activated leukocyte cell adhesion molecule [Source:HGNC Symbol;Acc:HGNC:400] | 1534 | 0.614 | 0.4386 | Yes |
| 46 | PIM1 | "Pim-1 proto-oncogene, serine/threonine kinase [Source:HGNC Symbol;Acc:HGNC:8986]" | 1567 | 0.607 | 0.4430 | Yes |
| 47 | BATF3 | basic leucine zipper ATF-like transcription factor 3 [Source:HGNC Symbol;Acc:HGNC:28915] | 1585 | 0.599 | 0.4483 | Yes |
| 48 | FAM126B | family with sequence similarity 126 member B [Source:HGNC Symbol;Acc:HGNC:28593] | 1607 | 0.593 | 0.4532 | Yes |
| 49 | PENK | proenkephalin [Source:HGNC Symbol;Acc:HGNC:8831] | 1668 | 0.576 | 0.4555 | Yes |
| 50 | GPR65 | G protein-coupled receptor 65 [Source:HGNC Symbol;Acc:HGNC:4517] | 1679 | 0.574 | 0.4610 | Yes |
| 51 | ETFBKMT | electron transfer flavoprotein subunit beta lysine methyltransferase [Source:HGNC Symbol;Acc:HGNC:28739] | 1820 | 0.539 | 0.4576 | Yes |
| 52 | CSF2 | colony stimulating factor 2 [Source:HGNC Symbol;Acc:HGNC:2434] | 1913 | 0.521 | 0.4572 | Yes |
| 53 | IFITM3 | interferon induced transmembrane protein 3 [Source:HGNC Symbol;Acc:HGNC:5414] | 1928 | 0.517 | 0.4618 | Yes |
| 54 | IKZF4 | IKAROS family zinc finger 4 [Source:HGNC Symbol;Acc:HGNC:13179] | 1955 | 0.511 | 0.4656 | Yes |
| 55 | CST7 | cystatin F [Source:HGNC Symbol;Acc:HGNC:2479] | 1956 | 0.511 | 0.4711 | Yes |
| 56 | ODC1 | ornithine decarboxylase 1 [Source:HGNC Symbol;Acc:HGNC:8109] | 2154 | 0.489 | 0.4635 | No |
| 57 | PLAGL1 | PLAG1 like zinc finger 1 [Source:HGNC Symbol;Acc:HGNC:9046] | 2305 | 0.467 | 0.4588 | No |
| 58 | PRKCH | protein kinase C eta [Source:HGNC Symbol;Acc:HGNC:9403] | 2306 | 0.467 | 0.4638 | No |
| 59 | P2RX4 | purinergic receptor P2X 4 [Source:HGNC Symbol;Acc:HGNC:8535] | 2318 | 0.466 | 0.4680 | No |
| 60 | CISH | cytokine inducible SH2 containing protein [Source:HGNC Symbol;Acc:HGNC:1984] | 2374 | 0.455 | 0.4693 | No |
| 61 | SLC39A8 | solute carrier family 39 member 8 [Source:HGNC Symbol;Acc:HGNC:20862] | 2608 | 0.434 | 0.4589 | No |
| 62 | CD83 | CD83 molecule [Source:HGNC Symbol;Acc:HGNC:1703] | 3005 | 0.403 | 0.4376 | No |
| 63 | FLT3LG | fms related receptor tyrosine kinase 3 ligand [Source:HGNC Symbol;Acc:HGNC:3766] | 3152 | 0.380 | 0.4322 | No |
| 64 | IL4R | interleukin 4 receptor [Source:HGNC Symbol;Acc:HGNC:6015] | 3233 | 0.368 | 0.4310 | No |
| 65 | PTCH1 | patched 1 [Source:HGNC Symbol;Acc:HGNC:9585] | 3244 | 0.367 | 0.4342 | No |
| 66 | SLC2A3 | solute carrier family 2 member 3 [Source:HGNC Symbol;Acc:HGNC:11007] | 3342 | 0.351 | 0.4317 | No |
| 67 | POU2F1 | POU class 2 homeobox 1 [Source:HGNC Symbol;Acc:HGNC:9212] | 3412 | 0.340 | 0.4308 | No |
| 68 | IRF6 | interferon regulatory factor 6 [Source:HGNC Symbol;Acc:HGNC:6121] | 3509 | 0.326 | 0.4281 | No |
| 69 | NFIL3 | "nuclear factor, interleukin 3 regulated [Source:HGNC Symbol;Acc:HGNC:7787]" | 3734 | 0.299 | 0.4168 | No |
| 70 | ARL4A | ADP ribosylation factor like GTPase 4A [Source:HGNC Symbol;Acc:HGNC:695] | 3817 | 0.289 | 0.4146 | No |
| 71 | CCR4 | C-C motif chemokine receptor 4 [Source:HGNC Symbol;Acc:HGNC:1605] | 3834 | 0.287 | 0.4166 | No |
| 72 | DRC1 | dynein regulatory complex subunit 1 [Source:HGNC Symbol;Acc:HGNC:24245] | 4080 | 0.256 | 0.4035 | No |
| 73 | MYO1E | myosin IE [Source:HGNC Symbol;Acc:HGNC:7599] | 4081 | 0.256 | 0.4062 | No |
| 74 | IL3RA | interleukin 3 receptor subunit alpha [Source:HGNC Symbol;Acc:HGNC:6012] | 4139 | 0.250 | 0.4052 | No |
| 75 | NCOA3 | nuclear receptor coactivator 3 [Source:HGNC Symbol;Acc:HGNC:7670] | 4525 | 0.211 | 0.3826 | No |
| 76 | MAP6 | microtubule associated protein 6 [Source:HGNC Symbol;Acc:HGNC:6868] | 4582 | 0.206 | 0.3812 | No |
| 77 | GLIPR2 | GLI pathogenesis related 2 [Source:HGNC Symbol;Acc:HGNC:18007] | 4636 | 0.202 | 0.3799 | No |
| 78 | RRAGD | Ras related GTP binding D [Source:HGNC Symbol;Acc:HGNC:19903] | 4762 | 0.192 | 0.3738 | No |
| 79 | BMPR2 | bone morphogenetic protein receptor type 2 [Source:HGNC Symbol;Acc:HGNC:1078] | 4766 | 0.192 | 0.3757 | No |
| 80 | GPR83 | G protein-coupled receptor 83 [Source:HGNC Symbol;Acc:HGNC:4523] | 4842 | 0.184 | 0.3728 | No |
| 81 | LRRC8C | leucine rich repeat containing 8 VRAC subunit C [Source:HGNC Symbol;Acc:HGNC:25075] | 4921 | 0.175 | 0.3696 | No |
| 82 | EOMES | eomesodermin [Source:HGNC Symbol;Acc:HGNC:3372] | 4947 | 0.173 | 0.3699 | No |
| 83 | PLIN2 | perilipin 2 [Source:HGNC Symbol;Acc:HGNC:248] | 5003 | 0.168 | 0.3681 | No |
| 84 | SLC1A5 | solute carrier family 1 member 5 [Source:HGNC Symbol;Acc:HGNC:10943] | 5126 | 0.156 | 0.3619 | No |
| 85 | MAPKAPK2 | MAPK activated protein kinase 2 [Source:HGNC Symbol;Acc:HGNC:6887] | 5177 | 0.153 | 0.3603 | No |
| 86 | HIPK2 | homeodomain interacting protein kinase 2 [Source:HGNC Symbol;Acc:HGNC:14402] | 5280 | 0.144 | 0.3552 | No |
| 87 | F2RL2 | coagulation factor II thrombin receptor like 2 [Source:HGNC Symbol;Acc:HGNC:3539] | 5339 | 0.140 | 0.3529 | No |
| 88 | CYFIP1 | cytoplasmic FMR1 interacting protein 1 [Source:HGNC Symbol;Acc:HGNC:13759] | 5353 | 0.138 | 0.3536 | No |
| 89 | SCN9A | sodium voltage-gated channel alpha subunit 9 [Source:HGNC Symbol;Acc:HGNC:10597] | 5386 | 0.136 | 0.3529 | No |
| 90 | EEF1AKMT1 | EEF1A lysine methyltransferase 1 [Source:HGNC Symbol;Acc:HGNC:27351] | 5554 | 0.124 | 0.3435 | No |
| 91 | KLF6 | Kruppel like factor 6 [Source:HGNC Symbol;Acc:HGNC:2235] | 5555 | 0.123 | 0.3448 | No |
| 92 | CDC42SE2 | CDC42 small effector 2 [Source:HGNC Symbol;Acc:HGNC:18547] | 5573 | 0.121 | 0.3450 | No |
| 93 | MUC1 | "mucin 1, cell surface associated [Source:HGNC Symbol;Acc:HGNC:7508]" | 5819 | 0.104 | 0.3302 | No |
| 94 | HOPX | HOP homeobox [Source:HGNC Symbol;Acc:HGNC:24961] | 5882 | 0.100 | 0.3273 | No |
| 95 | PHTF2 | putative homeodomain transcription factor 2 [Source:HGNC Symbol;Acc:HGNC:13411] | 6022 | 0.089 | 0.3193 | No |
| 96 | FAH | fumarylacetoacetate hydrolase [Source:HGNC Symbol;Acc:HGNC:3579] | 6356 | 0.066 | 0.2985 | No |
| 97 | MYC | "MYC proto-oncogene, bHLH transcription factor [Source:HGNC Symbol;Acc:HGNC:7553]" | 6504 | 0.055 | 0.2895 | No |
| 98 | CASP3 | caspase 3 [Source:HGNC Symbol;Acc:HGNC:1504] | 6509 | 0.054 | 0.2899 | No |
| 99 | TNFSF11 | TNF superfamily member 11 [Source:HGNC Symbol;Acc:HGNC:11926] | 6556 | 0.052 | 0.2874 | No |
| 100 | ADAM19 | ADAM metallopeptidase domain 19 [Source:HGNC Symbol;Acc:HGNC:197] | 6675 | 0.044 | 0.2803 | No |
| 101 | TNFRSF21 | TNF receptor superfamily member 21 [Source:HGNC Symbol;Acc:HGNC:13469] | 6692 | 0.043 | 0.2797 | No |
| 102 | ABCB1 | ATP binding cassette subfamily B member 1 [Source:HGNC Symbol;Acc:HGNC:40] | 6707 | 0.042 | 0.2792 | No |
| 103 | DCPS | "decapping enzyme, scavenger [Source:HGNC Symbol;Acc:HGNC:29812]" | 6801 | 0.036 | 0.2736 | No |
| 104 | PLSCR1 | phospholipid scramblase 1 [Source:HGNC Symbol;Acc:HGNC:9092] | 7180 | 0.013 | 0.2493 | No |
| 105 | RNH1 | ribonuclease/angiogenin inhibitor 1 [Source:HGNC Symbol;Acc:HGNC:10074] | 7225 | 0.010 | 0.2466 | No |
| 106 | LIF | LIF interleukin 6 family cytokine [Source:HGNC Symbol;Acc:HGNC:6596] | 7310 | 0.006 | 0.2412 | No |
| 107 | SH3BGRL2 | SH3 domain binding glutamate rich protein like 2 [Source:HGNC Symbol;Acc:HGNC:15567] | 7502 | -0.005 | 0.2289 | No |
| 108 | EMP1 | epithelial membrane protein 1 [Source:HGNC Symbol;Acc:HGNC:3333] | 7529 | -0.006 | 0.2273 | No |
| 109 | PHLDA1 | pleckstrin homology like domain family A member 1 [Source:HGNC Symbol;Acc:HGNC:8933] | 7553 | -0.007 | 0.2259 | No |
| 110 | RORA | RAR related orphan receptor A [Source:HGNC Symbol;Acc:HGNC:10258] | 7568 | -0.008 | 0.2251 | No |
| 111 | IFNGR1 | interferon gamma receptor 1 [Source:HGNC Symbol;Acc:HGNC:5439] | 7599 | -0.009 | 0.2232 | No |
| 112 | AHR | aryl hydrocarbon receptor [Source:HGNC Symbol;Acc:HGNC:348] | 7668 | -0.012 | 0.2190 | No |
| 113 | TNFRSF9 | TNF receptor superfamily member 9 [Source:HGNC Symbol;Acc:HGNC:11924] | 7797 | -0.019 | 0.2109 | No |
| 114 | CTSZ | cathepsin Z [Source:HGNC Symbol;Acc:HGNC:2547] | 7896 | -0.024 | 0.2048 | No |
| 115 | DHRS3 | dehydrogenase/reductase 3 [Source:HGNC Symbol;Acc:HGNC:17693] | 7935 | -0.025 | 0.2026 | No |
| 116 | CAPG | "capping actin protein, gelsolin like [Source:HGNC Symbol;Acc:HGNC:1474]" | 8049 | -0.031 | 0.1957 | No |
| 117 | ANXA4 | annexin A4 [Source:HGNC Symbol;Acc:HGNC:542] | 8076 | -0.033 | 0.1943 | No |
| 118 | CCND2 | cyclin D2 [Source:HGNC Symbol;Acc:HGNC:1583] | 8119 | -0.036 | 0.1920 | No |
| 119 | CDC6 | cell division cycle 6 [Source:HGNC Symbol;Acc:HGNC:1744] | 8163 | -0.038 | 0.1896 | No |
| 120 | LCLAT1 | lysocardiolipin acyltransferase 1 [Source:HGNC Symbol;Acc:HGNC:26756] | 8326 | -0.045 | 0.1796 | No |
| 121 | SNX14 | sorting nexin 14 [Source:HGNC Symbol;Acc:HGNC:14977] | 8370 | -0.047 | 0.1774 | No |
| 122 | AHNAK | AHNAK nucleoprotein [Source:HGNC Symbol;Acc:HGNC:347] | 8789 | -0.072 | 0.1511 | No |
| 123 | PRAF2 | PRA1 domain family member 2 [Source:HGNC Symbol;Acc:HGNC:28911] | 8852 | -0.076 | 0.1479 | No |
| 124 | SWAP70 | switching B cell complex subunit SWAP70 [Source:HGNC Symbol;Acc:HGNC:17070] | 8854 | -0.076 | 0.1486 | No |
| 125 | UMPS | uridine monophosphate synthetase [Source:HGNC Symbol;Acc:HGNC:12563] | 9005 | -0.083 | 0.1398 | No |
| 126 | NRP1 | neuropilin 1 [Source:HGNC Symbol;Acc:HGNC:8004] | 9151 | -0.091 | 0.1314 | No |
| 127 | CCNE1 | cyclin E1 [Source:HGNC Symbol;Acc:HGNC:1589] | 9202 | -0.093 | 0.1292 | No |
| 128 | UCK2 | uridine-cytidine kinase 2 [Source:HGNC Symbol;Acc:HGNC:12562] | 9334 | -0.100 | 0.1218 | No |
| 129 | SERPINB6 | serpin family B member 6 [Source:HGNC Symbol;Acc:HGNC:8950] | 9508 | -0.111 | 0.1118 | No |
| 130 | ST3GAL4 | "ST3 beta-galactoside alpha-2,3-sialyltransferase 4 [Source:HGNC Symbol;Acc:HGNC:10864]" | 9575 | -0.116 | 0.1088 | No |
| 131 | ENO3 | enolase 3 [Source:HGNC Symbol;Acc:HGNC:3354] | 9636 | -0.121 | 0.1062 | No |
| 132 | ITGAV | integrin subunit alpha V [Source:HGNC Symbol;Acc:HGNC:6150] | 9955 | -0.139 | 0.0871 | No |
| 133 | PLEC | plectin [Source:HGNC Symbol;Acc:HGNC:9069] | 10344 | -0.162 | 0.0637 | No |
| 134 | CCND3 | cyclin D3 [Source:HGNC Symbol;Acc:HGNC:1585] | 10446 | -0.168 | 0.0590 | No |
| 135 | GABARAPL1 | GABA type A receptor associated protein like 1 [Source:HGNC Symbol;Acc:HGNC:4068] | 10566 | -0.175 | 0.0532 | No |
| 136 | CD44 | CD44 molecule (Indian blood group) [Source:HGNC Symbol;Acc:HGNC:1681] | 10617 | -0.178 | 0.0518 | No |
| 137 | RABGAP1L | RAB GTPase activating protein 1 like [Source:HGNC Symbol;Acc:HGNC:24663] | 10642 | -0.180 | 0.0522 | No |
| 138 | PTRH2 | peptidyl-tRNA hydrolase 2 [Source:HGNC Symbol;Acc:HGNC:24265] | 10746 | -0.185 | 0.0475 | No |
| 139 | SELP | selectin P [Source:HGNC Symbol;Acc:HGNC:10721] | 11056 | -0.203 | 0.0297 | No |
| 140 | HUWE1 | "HECT, UBA and WWE domain containing E3 ubiquitin protein ligase 1 [Source:HGNC Symbol;Acc:HGNC:30892]" | 11205 | -0.212 | 0.0224 | No |
| 141 | FURIN | "furin, paired basic amino acid cleaving enzyme [Source:HGNC Symbol;Acc:HGNC:8568]" | 11210 | -0.212 | 0.0244 | No |
| 142 | RHOB | ras homolog family member B [Source:HGNC Symbol;Acc:HGNC:668] | 11216 | -0.212 | 0.0263 | No |
| 143 | LRIG1 | leucine rich repeats and immunoglobulin like domains 1 [Source:HGNC Symbol;Acc:HGNC:17360] | 11236 | -0.213 | 0.0274 | No |
| 144 | IGF1R | insulin like growth factor 1 receptor [Source:HGNC Symbol;Acc:HGNC:5465] | 11329 | -0.219 | 0.0238 | No |
| 145 | HK2 | hexokinase 2 [Source:HGNC Symbol;Acc:HGNC:4923] | 11425 | -0.225 | 0.0200 | No |
| 146 | SYT11 | synaptotagmin 11 [Source:HGNC Symbol;Acc:HGNC:19239] | 11450 | -0.227 | 0.0209 | No |
| 147 | CDCP1 | CUB domain containing protein 1 [Source:HGNC Symbol;Acc:HGNC:24357] | 11486 | -0.229 | 0.0211 | No |
| 148 | GPX4 | glutathione peroxidase 4 [Source:HGNC Symbol;Acc:HGNC:4556] | 11537 | -0.231 | 0.0203 | No |
| 149 | SPRY4 | sprouty RTK signaling antagonist 4 [Source:HGNC Symbol;Acc:HGNC:15533] | 11588 | -0.235 | 0.0196 | No |
| 150 | SYNGR2 | synaptogyrin 2 [Source:HGNC Symbol;Acc:HGNC:11499] | 11596 | -0.235 | 0.0216 | No |
| 151 | BCL2 | BCL2 apoptosis regulator [Source:HGNC Symbol;Acc:HGNC:990] | 11610 | -0.236 | 0.0233 | No |
| 152 | SPRED2 | sprouty related EVH1 domain containing 2 [Source:HGNC Symbol;Acc:HGNC:17722] | 11611 | -0.236 | 0.0258 | No |
| 153 | CDKN1C | cyclin dependent kinase inhibitor 1C [Source:HGNC Symbol;Acc:HGNC:1786] | 11672 | -0.239 | 0.0245 | No |
| 154 | GALM | galactose mutarotase [Source:HGNC Symbol;Acc:HGNC:24063] | 11864 | -0.252 | 0.0148 | No |
| 155 | BCL2L1 | BCL2 like 1 [Source:HGNC Symbol;Acc:HGNC:992] | 12157 | -0.271 | -0.0012 | No |
| 156 | ECM1 | extracellular matrix protein 1 [Source:HGNC Symbol;Acc:HGNC:3153] | 12213 | -0.274 | -0.0018 | No |
| 157 | DENND5A | DENN domain containing 5A [Source:HGNC Symbol;Acc:HGNC:19344] | 12266 | -0.278 | -0.0022 | No |
| 158 | AHCY | adenosylhomocysteinase [Source:HGNC Symbol;Acc:HGNC:343] | 12331 | -0.283 | -0.0034 | No |
| 159 | ETV4 | ETS variant transcription factor 4 [Source:HGNC Symbol;Acc:HGNC:3493] | 12369 | -0.286 | -0.0027 | No |
| 160 | PRNP | prion protein [Source:HGNC Symbol;Acc:HGNC:9449] | 12403 | -0.289 | -0.0018 | No |
| 161 | IGF2R | insulin like growth factor 2 receptor [Source:HGNC Symbol;Acc:HGNC:5467] | 12501 | -0.295 | -0.0049 | No |
| 162 | COCH | cochlin [Source:HGNC Symbol;Acc:HGNC:2180] | 12545 | -0.300 | -0.0045 | No |
| 163 | MYO1C | myosin IC [Source:HGNC Symbol;Acc:HGNC:7597] | 12593 | -0.303 | -0.0043 | No |
| 164 | PDCD2L | programmed cell death 2 like [Source:HGNC Symbol;Acc:HGNC:28194] | 12602 | -0.304 | -0.0016 | No |
| 165 | BMP2 | bone morphogenetic protein 2 [Source:HGNC Symbol;Acc:HGNC:1069] | 12618 | -0.305 | 0.0007 | No |
| 166 | MAFF | MAF bZIP transcription factor F [Source:HGNC Symbol;Acc:HGNC:6780] | 12675 | -0.309 | 0.0004 | No |
| 167 | NDRG1 | N-myc downstream regulated 1 [Source:HGNC Symbol;Acc:HGNC:7679] | 12779 | -0.318 | -0.0029 | No |
| 168 | SHE | Src homology 2 domain containing E [Source:HGNC Symbol;Acc:HGNC:27004] | 12935 | -0.332 | -0.0094 | No |
| 169 | PUS1 | pseudouridine synthase 1 [Source:HGNC Symbol;Acc:HGNC:15508] | 13066 | -0.344 | -0.0141 | No |
| 170 | AMACR | alpha-methylacyl-CoA racemase [Source:HGNC Symbol;Acc:HGNC:451] | 13192 | -0.355 | -0.0184 | No |
| 171 | PLPP1 | phospholipid phosphatase 1 [Source:HGNC Symbol;Acc:HGNC:9228] | 13203 | -0.356 | -0.0152 | No |
| 172 | P4HA1 | prolyl 4-hydroxylase subunit alpha 1 [Source:HGNC Symbol;Acc:HGNC:8546] | 13262 | -0.362 | -0.0151 | No |
| 173 | TWSG1 | twisted gastrulation BMP signaling modulator 1 [Source:HGNC Symbol;Acc:HGNC:12429] | 13337 | -0.371 | -0.0160 | No |
| 174 | XBP1 | X-box binding protein 1 [Source:HGNC Symbol;Acc:HGNC:12801] | 13607 | -0.406 | -0.0290 | No |
| 175 | ENPP1 | ectonucleotide pyrophosphatase/phosphodiesterase 1 [Source:HGNC Symbol;Acc:HGNC:3356] | 13655 | -0.412 | -0.0277 | No |
| 176 | GUCY1B1 | guanylate cyclase 1 soluble subunit beta 1 [Source:HGNC Symbol;Acc:HGNC:4687] | 13786 | -0.416 | -0.0316 | No |
| 177 | GSTO1 | glutathione S-transferase omega 1 [Source:HGNC Symbol;Acc:HGNC:13312] | 13804 | -0.419 | -0.0283 | No |
| 178 | SLC29A2 | solute carrier family 29 member 2 [Source:HGNC Symbol;Acc:HGNC:11004] | 13975 | -0.428 | -0.0347 | No |
| 179 | BHLHE40 | basic helix-loop-helix family member e40 [Source:HGNC Symbol;Acc:HGNC:1046] | 14032 | -0.437 | -0.0337 | No |
| 180 | CKAP4 | cytoskeleton associated protein 4 [Source:HGNC Symbol;Acc:HGNC:16991] | 14093 | -0.447 | -0.0328 | No |
| 181 | NCS1 | neuronal calcium sensor 1 [Source:HGNC Symbol;Acc:HGNC:3953] | 14105 | -0.450 | -0.0287 | No |
| 182 | SERPINC1 | serpin family C member 1 [Source:HGNC Symbol;Acc:HGNC:775] | 14208 | -0.466 | -0.0303 | No |
| 183 | SOCS2 | suppressor of cytokine signaling 2 [Source:HGNC Symbol;Acc:HGNC:19382] | 14286 | -0.480 | -0.0302 | No |
| 184 | ITGA6 | integrin subunit alpha 6 [Source:HGNC Symbol;Acc:HGNC:6142] | 14340 | -0.485 | -0.0285 | No |
| 185 | NOP2 | NOP2 nucleolar protein [Source:HGNC Symbol;Acc:HGNC:7867] | 14568 | -0.527 | -0.0375 | No |
| 186 | SPP1 | secreted phosphoprotein 1 [Source:HGNC Symbol;Acc:HGNC:11255] | 14610 | -0.535 | -0.0345 | No |
| 187 | APLP1 | amyloid beta precursor like protein 1 [Source:HGNC Symbol;Acc:HGNC:597] | 14707 | -0.556 | -0.0348 | No |
| 188 | CSF1 | colony stimulating factor 1 [Source:HGNC Symbol;Acc:HGNC:2432] | 14746 | -0.566 | -0.0312 | No |
| 189 | SNX9 | sorting nexin 9 [Source:HGNC Symbol;Acc:HGNC:14973] | 14801 | -0.584 | -0.0285 | No |
| 190 | WLS | Wnt ligand secretion mediator [Source:HGNC Symbol;Acc:HGNC:30238] | 14960 | -0.648 | -0.0318 | No |
| 191 | S100A1 | S100 calcium binding protein A1 [Source:HGNC Symbol;Acc:HGNC:10486] | 15184 | -0.768 | -0.0380 | No |
| 192 | TNFRSF8 | TNF receptor superfamily member 8 [Source:HGNC Symbol;Acc:HGNC:11923] | 15348 | -0.860 | -0.0394 | No |
| 193 | CD81 | CD81 molecule [Source:HGNC Symbol;Acc:HGNC:1701] | 15353 | -0.863 | -0.0305 | No |
| 194 | NT5E | 5'-nucleotidase ecto [Source:HGNC Symbol;Acc:HGNC:8021] | 15487 | -1.022 | -0.0282 | No |
| 195 | CA2 | carbonic anhydrase 2 [Source:HGNC Symbol;Acc:HGNC:1373] | 15522 | -1.087 | -0.0188 | No |
| 196 | ITIH5 | inter-alpha-trypsin inhibitor heavy chain 5 [Source:HGNC Symbol;Acc:HGNC:21449] | 15585 | -1.236 | -0.0096 | No |
| 197 | COL6A1 | collagen type VI alpha 1 chain [Source:HGNC Symbol;Acc:HGNC:2211] | 15612 | -1.403 | 0.0036 | No |
Table: GSEA details [plain text format]

  

Fig 2: HALLMARK\_IL2\_STAT5\_SIGNALING      
 Blue-Pink O' Gram in the Space of the Analyzed GeneSet

  

Fig 3: HALLMARK\_IL2\_STAT5\_SIGNALING: Random ES distribution      
 Gene set null distribution of ES for **HALLMARK\_IL2\_STAT5\_SIGNALING**
