# Supplementary material for: In vivo CRISPR screens reveal Serpinb9 and Adam2 as regulators of immune therapy response in lung cancer
Source: Nat Commun. 2023 May 31;14:3150. doi: 10.1038/s41467-023-38841-7 (PMC10232477; doi:10.1038/s41467-023-38841-7)
Supplement: Supplementary file 13 — Source Data [file 41467_2023_38841_MOESM13_ESM.zip › Figure 4E.zip!/my_analysis.Gsea.1640146340255_Hallmark/heat_map_corr_plot.html]

Heat map and correlation plot for December 21, 2021\_for GSEA run\_collapsed\_to\_symbols  

Fig 1: heat\_map      
 Heat Map of the top 50 features for each phenotype in December 21, 2021\_for GSEA run\_collapsed\_to\_symbols

  
  

Fig 2: Ranked Gene List Correlation Profile      
 Ranked list correlations for December 21, 2021\_for GSEA run\_collapsed\_to\_symbols

  
  
    
