# Supplementary material for: In vivo CRISPR screens reveal Serpinb9 and Adam2 as regulators of immune therapy response in lung cancer
Source: Nat Commun. 2023 May 31;14:3150. doi: 10.1038/s41467-023-38841-7 (PMC10232477; doi:10.1038/s41467-023-38841-7)
Supplement: Supplementary file 13 — Source Data [file 41467_2023_38841_MOESM13_ESM.zip › Figure 4E.zip!/my_analysis.Gsea.1640146340255_Hallmark/HALLMARK_PEROXISOME.html]

Details for gene set HALLMARK\_PEROXISOME[GSEA]

|  || Dataset | December 21, 2021\_for GSEA run\_collapsed\_to\_symbols.GSEA\_B6\_NI.cls #CTLR\_NI\_versus\_ADAM2\_NI |
| Phenotype | GSEA\_B6\_NI.cls#CTLR\_NI\_versus\_ADAM2\_NI |
| Upregulated in class | ADAM2\_NI |
| GeneSet | HALLMARK\_PEROXISOME |
| Enrichment Score (ES) | -0.2210222 |
| Normalized Enrichment Score (NES) | -1.0866034 |
| Nominal p-value | 0.27868852 |
| FDR q-value | 0.79780936 |
| FWER p-Value | 1.0 |
Table: GSEA Results Summary

  

Fig 1: Enrichment plot: HALLMARK\_PEROXISOME      
 Profile of the Running ES Score & Positions of GeneSet Members on the Rank Ordered List

  

| SYMBOL | TITLE | RANK IN GENE LIST | RANK METRIC SCORE | RUNNING ES | CORE ENRICHMENT || 1 | ALDH1A1 | aldehyde dehydrogenase 1 family member A1 [Source:HGNC Symbol;Acc:HGNC:402] | 1061 | 0.787 | -0.0350 | No |
| 2 | ACSL1 | acyl-CoA synthetase long chain family member 1 [Source:HGNC Symbol;Acc:HGNC:3569] | 1411 | 0.657 | -0.0298 | No |
| 3 | SLC27A2 | solute carrier family 27 member 2 [Source:HGNC Symbol;Acc:HGNC:10996] | 1720 | 0.562 | -0.0259 | No |
| 4 | ABCD2 | ATP binding cassette subfamily D member 2 [Source:HGNC Symbol;Acc:HGNC:66] | 1973 | 0.508 | -0.0207 | No |
| 5 | RXRG | retinoid X receptor gamma [Source:HGNC Symbol;Acc:HGNC:10479] | 2013 | 0.503 | -0.0021 | No |
| 6 | HSD11B2 | hydroxysteroid 11-beta dehydrogenase 2 [Source:HGNC Symbol;Acc:HGNC:5209] | 2085 | 0.494 | 0.0142 | No |
| 7 | EPHX2 | epoxide hydrolase 2 [Source:HGNC Symbol;Acc:HGNC:3402] | 2182 | 0.482 | 0.0283 | No |
| 8 | CEL | carboxyl ester lipase [Source:HGNC Symbol;Acc:HGNC:1848] | 2834 | 0.411 | 0.0038 | No |
| 9 | ALB | albumin [Source:HGNC Symbol;Acc:HGNC:399] | 2947 | 0.411 | 0.0139 | No |
| 10 | ABCC8 | ATP binding cassette subfamily C member 8 [Source:HGNC Symbol;Acc:HGNC:59] | 2951 | 0.411 | 0.0310 | No |
| 11 | CLN8 | CLN8 transmembrane ER and ERGIC protein [Source:HGNC Symbol;Acc:HGNC:2079] | 3021 | 0.401 | 0.0435 | No |
| 12 | VPS4B | vacuolar protein sorting 4 homolog B [Source:HGNC Symbol;Acc:HGNC:10895] | 3343 | 0.351 | 0.0376 | No |
| 13 | ABCB9 | ATP binding cassette subfamily B member 9 [Source:HGNC Symbol;Acc:HGNC:50] | 3381 | 0.345 | 0.0497 | No |
| 14 | TOP2A | DNA topoisomerase II alpha [Source:HGNC Symbol;Acc:HGNC:11989] | 3608 | 0.314 | 0.0484 | No |
| 15 | CRABP2 | cellular retinoic acid binding protein 2 [Source:HGNC Symbol;Acc:HGNC:2339] | 3828 | 0.288 | 0.0465 | No |
| 16 | SULT2B1 | sulfotransferase family 2B member 1 [Source:HGNC Symbol;Acc:HGNC:11459] | 4193 | 0.245 | 0.0334 | No |
| 17 | ACSL5 | acyl-CoA synthetase long chain family member 5 [Source:HGNC Symbol;Acc:HGNC:16526] | 4458 | 0.217 | 0.0256 | No |
| 18 | ACOT8 | acyl-CoA thioesterase 8 [Source:HGNC Symbol;Acc:HGNC:15919] | 4635 | 0.202 | 0.0228 | No |
| 19 | CRABP1 | cellular retinoic acid binding protein 1 [Source:HGNC Symbol;Acc:HGNC:2338] | 4639 | 0.202 | 0.0311 | No |
| 20 | HSD17B11 | hydroxysteroid 17-beta dehydrogenase 11 [Source:HGNC Symbol;Acc:HGNC:22960] | 4698 | 0.197 | 0.0356 | No |
| 21 | ACSL4 | acyl-CoA synthetase long chain family member 4 [Source:HGNC Symbol;Acc:HGNC:3571] | 4768 | 0.191 | 0.0393 | No |
| 22 | GSTK1 | glutathione S-transferase kappa 1 [Source:HGNC Symbol;Acc:HGNC:16906] | 5019 | 0.166 | 0.0302 | No |
| 23 | RDH11 | retinol dehydrogenase 11 [Source:HGNC Symbol;Acc:HGNC:17964] | 5129 | 0.155 | 0.0297 | No |
| 24 | ATXN1 | ataxin 1 [Source:HGNC Symbol;Acc:HGNC:10548] | 5203 | 0.151 | 0.0314 | No |
| 25 | ERCC3 | "ERCC excision repair 3, TFIIH core complex helicase subunit [Source:HGNC Symbol;Acc:HGNC:3435]" | 5472 | 0.129 | 0.0196 | No |
| 26 | ABCC5 | ATP binding cassette subfamily C member 5 [Source:HGNC Symbol;Acc:HGNC:56] | 5602 | 0.119 | 0.0163 | No |
| 27 | EHHADH | enoyl-CoA hydratase and 3-hydroxyacyl CoA dehydrogenase [Source:HGNC Symbol;Acc:HGNC:3247] | 5669 | 0.114 | 0.0169 | No |
| 28 | ESR2 | estrogen receptor 2 [Source:HGNC Symbol;Acc:HGNC:3468] | 6176 | 0.078 | -0.0123 | No |
| 29 | PRDX5 | peroxiredoxin 5 [Source:HGNC Symbol;Acc:HGNC:9355] | 6408 | 0.062 | -0.0245 | No |
| 30 | ALDH9A1 | aldehyde dehydrogenase 9 family member A1 [Source:HGNC Symbol;Acc:HGNC:412] | 6489 | 0.056 | -0.0273 | No |
| 31 | ABCD1 | ATP binding cassette subfamily D member 1 [Source:HGNC Symbol;Acc:HGNC:61] | 6559 | 0.051 | -0.0296 | No |
| 32 | ABCB1 | ATP binding cassette subfamily B member 1 [Source:HGNC Symbol;Acc:HGNC:40] | 6707 | 0.042 | -0.0373 | No |
| 33 | TSPO | translocator protein [Source:HGNC Symbol;Acc:HGNC:1158] | 7047 | 0.021 | -0.0581 | No |
| 34 | PEX6 | peroxisomal biogenesis factor 6 [Source:HGNC Symbol;Acc:HGNC:8859] | 7061 | 0.020 | -0.0581 | No |
| 35 | SCP2 | sterol carrier protein 2 [Source:HGNC Symbol;Acc:HGNC:10606] | 7266 | 0.009 | -0.0708 | No |
| 36 | MSH2 | mutS homolog 2 [Source:HGNC Symbol;Acc:HGNC:7325] | 7311 | 0.006 | -0.0734 | No |
| 37 | ISOC1 | isochorismatase domain containing 1 [Source:HGNC Symbol;Acc:HGNC:24254] | 7415 | -0.000 | -0.0800 | No |
| 38 | SLC23A2 | solute carrier family 23 member 2 [Source:HGNC Symbol;Acc:HGNC:10973] | 7496 | -0.004 | -0.0850 | No |
| 39 | CDK7 | cyclin dependent kinase 7 [Source:HGNC Symbol;Acc:HGNC:1778] | 7503 | -0.005 | -0.0852 | No |
| 40 | SOD2 | superoxide dismutase 2 [Source:HGNC Symbol;Acc:HGNC:11180] | 7867 | -0.022 | -0.1076 | No |
| 41 | CNBP | CCHC-type zinc finger nucleic acid binding protein [Source:HGNC Symbol;Acc:HGNC:13164] | 7912 | -0.025 | -0.1094 | No |
| 42 | DHRS3 | dehydrogenase/reductase 3 [Source:HGNC Symbol;Acc:HGNC:17693] | 7935 | -0.025 | -0.1097 | No |
| 43 | HSD17B4 | hydroxysteroid 17-beta dehydrogenase 4 [Source:HGNC Symbol;Acc:HGNC:5213] | 7943 | -0.026 | -0.1091 | No |
| 44 | IDH1 | isocitrate dehydrogenase (NADP(+)) 1 [Source:HGNC Symbol;Acc:HGNC:5382] | 8014 | -0.030 | -0.1123 | No |
| 45 | ABCD3 | ATP binding cassette subfamily D member 3 [Source:HGNC Symbol;Acc:HGNC:67] | 8074 | -0.033 | -0.1147 | No |
| 46 | SCGB1A1 | secretoglobin family 1A member 1 [Source:HGNC Symbol;Acc:HGNC:12523] | 8269 | -0.042 | -0.1254 | No |
| 47 | ITGB1BP1 | integrin subunit beta 1 binding protein 1 [Source:HGNC Symbol;Acc:HGNC:23927] | 8327 | -0.045 | -0.1272 | No |
| 48 | YWHAH | tyrosine 3-monooxygenase/tryptophan 5-monooxygenase activation protein eta [Source:HGNC Symbol;Acc:HGNC:12853] | 8556 | -0.058 | -0.1394 | No |
| 49 | PEX5 | peroxisomal biogenesis factor 5 [Source:HGNC Symbol;Acc:HGNC:9719] | 8557 | -0.058 | -0.1369 | No |
| 50 | CLN6 | CLN6 transmembrane ER protein [Source:HGNC Symbol;Acc:HGNC:2077] | 8562 | -0.058 | -0.1347 | No |
| 51 | PRDX1 | peroxiredoxin 1 [Source:HGNC Symbol;Acc:HGNC:9352] | 8653 | -0.064 | -0.1378 | No |
| 52 | CAT | catalase [Source:HGNC Symbol;Acc:HGNC:1516] | 9069 | -0.086 | -0.1609 | No |
| 53 | SLC25A19 | solute carrier family 25 member 19 [Source:HGNC Symbol;Acc:HGNC:14409] | 9145 | -0.090 | -0.1619 | No |
| 54 | CADM1 | cell adhesion molecule 1 [Source:HGNC Symbol;Acc:HGNC:5951] | 9550 | -0.114 | -0.1830 | No |
| 55 | MVP | major vault protein [Source:HGNC Symbol;Acc:HGNC:7531] | 9551 | -0.114 | -0.1782 | No |
| 56 | NUDT19 | nudix hydrolase 19 [Source:HGNC Symbol;Acc:HGNC:32036] | 9564 | -0.116 | -0.1741 | No |
| 57 | IDI1 | isopentenyl-diphosphate delta isomerase 1 [Source:HGNC Symbol;Acc:HGNC:5387] | 9590 | -0.117 | -0.1708 | No |
| 58 | CRAT | carnitine O-acetyltransferase [Source:HGNC Symbol;Acc:HGNC:2342] | 9657 | -0.122 | -0.1699 | No |
| 59 | GNPAT | glyceronephosphate O-acyltransferase [Source:HGNC Symbol;Acc:HGNC:4416] | 9820 | -0.132 | -0.1747 | No |
| 60 | ACAA1 | acetyl-CoA acyltransferase 1 [Source:HGNC Symbol;Acc:HGNC:82] | 9995 | -0.142 | -0.1799 | No |
| 61 | HRAS | "HRas proto-oncogene, GTPase [Source:HGNC Symbol;Acc:HGNC:5173]" | 10331 | -0.161 | -0.1947 | No |
| 62 | LONP2 | "lon peptidase 2, peroxisomal [Source:HGNC Symbol;Acc:HGNC:20598]" | 10542 | -0.174 | -0.2008 | No |
| 63 | SMARCC1 | "SWI/SNF related, matrix associated, actin dependent regulator of chromatin subfamily c member 1 [Source:HGNC Symbol;Acc:HGNC:11104]" | 10675 | -0.181 | -0.2017 | No |
| 64 | SOD1 | superoxide dismutase 1 [Source:HGNC Symbol;Acc:HGNC:11179] | 10977 | -0.199 | -0.2126 | Yes |
| 65 | PEX2 | peroxisomal biogenesis factor 2 [Source:HGNC Symbol;Acc:HGNC:9717] | 10995 | -0.200 | -0.2053 | Yes |
| 66 | PEX13 | peroxisomal biogenesis factor 13 [Source:HGNC Symbol;Acc:HGNC:8855] | 11000 | -0.200 | -0.1971 | Yes |
| 67 | SLC25A17 | solute carrier family 25 member 17 [Source:HGNC Symbol;Acc:HGNC:10987] | 11011 | -0.201 | -0.1893 | Yes |
| 68 | PABPC1 | poly(A) binding protein cytoplasmic 1 [Source:HGNC Symbol;Acc:HGNC:8554] | 11145 | -0.209 | -0.1891 | Yes |
| 69 | HMGCL | 3-hydroxy-3-methylglutaryl-CoA lyase [Source:HGNC Symbol;Acc:HGNC:5005] | 11164 | -0.210 | -0.1814 | Yes |
| 70 | PEX14 | peroxisomal biogenesis factor 14 [Source:HGNC Symbol;Acc:HGNC:8856] | 11500 | -0.229 | -0.1933 | Yes |
| 71 | ECI2 | enoyl-CoA delta isomerase 2 [Source:HGNC Symbol;Acc:HGNC:14601] | 11800 | -0.248 | -0.2020 | Yes |
| 72 | ACOX1 | acyl-CoA oxidase 1 [Source:HGNC Symbol;Acc:HGNC:119] | 11941 | -0.257 | -0.2002 | Yes |
| 73 | ERCC1 | "ERCC excision repair 1, endonuclease non-catalytic subunit [Source:HGNC Symbol;Acc:HGNC:3433]" | 12105 | -0.268 | -0.1994 | Yes |
| 74 | ECH1 | enoyl-CoA hydratase 1 [Source:HGNC Symbol;Acc:HGNC:3149] | 12129 | -0.269 | -0.1896 | Yes |
| 75 | FIS1 | "fission, mitochondrial 1 [Source:HGNC Symbol;Acc:HGNC:21689]" | 12171 | -0.272 | -0.1808 | Yes |
| 76 | CTBP1 | C-terminal binding protein 1 [Source:HGNC Symbol;Acc:HGNC:2494] | 12200 | -0.273 | -0.1711 | Yes |
| 77 | DHCR24 | 24-dehydrocholesterol reductase [Source:HGNC Symbol;Acc:HGNC:2859] | 12239 | -0.276 | -0.1619 | Yes |
| 78 | CTPS1 | CTP synthase 1 [Source:HGNC Symbol;Acc:HGNC:2519] | 12310 | -0.281 | -0.1546 | Yes |
| 79 | DLG4 | discs large MAGUK scaffold protein 4 [Source:HGNC Symbol;Acc:HGNC:2903] | 12320 | -0.282 | -0.1433 | Yes |
| 80 | MLYCD | malonyl-CoA decarboxylase [Source:HGNC Symbol;Acc:HGNC:7150] | 12390 | -0.288 | -0.1356 | Yes |
| 81 | IDE | insulin degrading enzyme [Source:HGNC Symbol;Acc:HGNC:5381] | 12846 | -0.323 | -0.1512 | Yes |
| 82 | PEX11B | peroxisomal biogenesis factor 11 beta [Source:HGNC Symbol;Acc:HGNC:8853] | 12858 | -0.324 | -0.1383 | Yes |
| 83 | ELOVL5 | ELOVL fatty acid elongase 5 [Source:HGNC Symbol;Acc:HGNC:21308] | 12900 | -0.328 | -0.1271 | Yes |
| 84 | BCL10 | BCL10 immune signaling adaptor [Source:HGNC Symbol;Acc:HGNC:989] | 13120 | -0.349 | -0.1265 | Yes |
| 85 | SLC25A4 | solute carrier family 25 member 4 [Source:HGNC Symbol;Acc:HGNC:10990] | 13135 | -0.350 | -0.1127 | Yes |
| 86 | FADS1 | fatty acid desaturase 1 [Source:HGNC Symbol;Acc:HGNC:3574] | 13531 | -0.396 | -0.1214 | Yes |
| 87 | SIAH1 | siah E3 ubiquitin protein ligase 1 [Source:HGNC Symbol;Acc:HGNC:10857] | 13669 | -0.413 | -0.1128 | Yes |
| 88 | IDH2 | isocitrate dehydrogenase (NADP(+)) 2 [Source:HGNC Symbol;Acc:HGNC:5383] | 13807 | -0.419 | -0.1040 | Yes |
| 89 | FDPS | farnesyl diphosphate synthase [Source:HGNC Symbol;Acc:HGNC:3631] | 14135 | -0.455 | -0.1058 | Yes |
| 90 | PEX11A | peroxisomal biogenesis factor 11 alpha [Source:HGNC Symbol;Acc:HGNC:8852] | 14698 | -0.553 | -0.1186 | Yes |
| 91 | RETSAT | retinol saturase [Source:HGNC Symbol;Acc:HGNC:25991] | 14885 | -0.613 | -0.1048 | Yes |
| 92 | ABCB4 | ATP binding cassette subfamily B member 4 [Source:HGNC Symbol;Acc:HGNC:45] | 14931 | -0.634 | -0.0810 | Yes |
| 93 | SEMA3C | semaphorin 3C [Source:HGNC Symbol;Acc:HGNC:10725] | 14972 | -0.653 | -0.0561 | Yes |
| 94 | SLC35B2 | solute carrier family 35 member B2 [Source:HGNC Symbol;Acc:HGNC:16872] | 15167 | -0.759 | -0.0366 | Yes |
| 95 | CACNA1B | calcium voltage-gated channel subunit alpha1 B [Source:HGNC Symbol;Acc:HGNC:1389] | 15644 | -1.634 | 0.0015 | Yes |
Table: GSEA details [plain text format]

  

Fig 2: HALLMARK\_PEROXISOME      
 Blue-Pink O' Gram in the Space of the Analyzed GeneSet

  

Fig 3: HALLMARK\_PEROXISOME: Random ES distribution      
 Gene set null distribution of ES for **HALLMARK\_PEROXISOME**
