# Supplementary material for: In vivo CRISPR screens reveal Serpinb9 and Adam2 as regulators of immune therapy response in lung cancer
Source: Nat Commun. 2023 May 31;14:3150. doi: 10.1038/s41467-023-38841-7 (PMC10232477; doi:10.1038/s41467-023-38841-7)
Supplement: Supplementary file 13 — Source Data [file 41467_2023_38841_MOESM13_ESM.zip › Figure 4E.zip!/my_analysis.Gsea.1640146340255_Hallmark/HALLMARK_HEME_METABOLISM.html]

Details for gene set HALLMARK\_HEME\_METABOLISM[GSEA]

|  || Dataset | December 21, 2021\_for GSEA run\_collapsed\_to\_symbols.GSEA\_B6\_NI.cls #CTLR\_NI\_versus\_ADAM2\_NI |
| Phenotype | GSEA\_B6\_NI.cls#CTLR\_NI\_versus\_ADAM2\_NI |
| Upregulated in class | ADAM2\_NI |
| GeneSet | HALLMARK\_HEME\_METABOLISM |
| Enrichment Score (ES) | -0.19450904 |
| Normalized Enrichment Score (NES) | -0.8300503 |
| Nominal p-value | 0.8989899 |
| FDR q-value | 0.8159049 |
| FWER p-Value | 1.0 |
Table: GSEA Results Summary

  

Fig 1: Enrichment plot: HALLMARK\_HEME\_METABOLISM      
 Profile of the Running ES Score & Positions of GeneSet Members on the Rank Ordered List

  

| SYMBOL | TITLE | RANK IN GENE LIST | RANK METRIC SCORE | RUNNING ES | CORE ENRICHMENT || 1 | C3 | complement C3 [Source:HGNC Symbol;Acc:HGNC:1318] | 66 | 1.929 | 0.0340 | No |
| 2 | XK | X-linked Kx blood group [Source:HGNC Symbol;Acc:HGNC:12811] | 175 | 1.565 | 0.0580 | No |
| 3 | SLC30A1 | solute carrier family 30 member 1 [Source:HGNC Symbol;Acc:HGNC:11012] | 529 | 1.036 | 0.0558 | No |
| 4 | CTSE | cathepsin E [Source:HGNC Symbol;Acc:HGNC:2530] | 590 | 1.003 | 0.0718 | No |
| 5 | GATA1 | GATA binding protein 1 [Source:HGNC Symbol;Acc:HGNC:4170] | 928 | 0.839 | 0.0667 | No |
| 6 | SYNJ1 | synaptojanin 1 [Source:HGNC Symbol;Acc:HGNC:11503] | 1177 | 0.740 | 0.0653 | No |
| 7 | MOCOS | molybdenum cofactor sulfurase [Source:HGNC Symbol;Acc:HGNC:18234] | 1192 | 0.735 | 0.0790 | No |
| 8 | SLC25A37 | solute carrier family 25 member 37 [Source:HGNC Symbol;Acc:HGNC:29786] | 1405 | 0.659 | 0.0784 | No |
| 9 | ACKR1 | atypical chemokine receptor 1 (Duffy blood group) [Source:HGNC Symbol;Acc:HGNC:4035] | 1494 | 0.628 | 0.0852 | No |
| 10 | SPTB | "spectrin beta, erythrocytic [Source:HGNC Symbol;Acc:HGNC:11274]" | 1810 | 0.541 | 0.0755 | No |
| 11 | SMOX | spermine oxidase [Source:HGNC Symbol;Acc:HGNC:15862] | 2328 | 0.465 | 0.0514 | No |
| 12 | TENT5C | terminal nucleotidyltransferase 5C [Source:HGNC Symbol;Acc:HGNC:24712] | 2415 | 0.448 | 0.0547 | No |
| 13 | BACH1 | BTB domain and CNC homolog 1 [Source:HGNC Symbol;Acc:HGNC:935] | 2762 | 0.424 | 0.0408 | No |
| 14 | KHNYN | KH and NYN domain containing [Source:HGNC Symbol;Acc:HGNC:20166] | 2800 | 0.415 | 0.0466 | No |
| 15 | E2F2 | E2F transcription factor 2 [Source:HGNC Symbol;Acc:HGNC:3114] | 2809 | 0.414 | 0.0543 | No |
| 16 | ELL2 | elongation factor for RNA polymerase II 2 [Source:HGNC Symbol;Acc:HGNC:17064] | 3031 | 0.399 | 0.0479 | No |
| 17 | RIOK3 | RIO kinase 3 [Source:HGNC Symbol;Acc:HGNC:11451] | 3093 | 0.389 | 0.0517 | No |
| 18 | LMO2 | LIM domain only 2 [Source:HGNC Symbol;Acc:HGNC:6642] | 3196 | 0.374 | 0.0525 | No |
| 19 | BTG2 | BTG anti-proliferation factor 2 [Source:HGNC Symbol;Acc:HGNC:1131] | 3319 | 0.354 | 0.0517 | No |
| 20 | CTSB | cathepsin B [Source:HGNC Symbol;Acc:HGNC:2527] | 3373 | 0.346 | 0.0551 | No |
| 21 | NCOA4 | nuclear receptor coactivator 4 [Source:HGNC Symbol;Acc:HGNC:7671] | 3376 | 0.345 | 0.0618 | No |
| 22 | ABCG2 | ATP binding cassette subfamily G member 2 (Junior blood group) [Source:HGNC Symbol;Acc:HGNC:74] | 3426 | 0.338 | 0.0654 | No |
| 23 | MYL4 | myosin light chain 4 [Source:HGNC Symbol;Acc:HGNC:7585] | 3480 | 0.330 | 0.0685 | No |
| 24 | BMP2K | BMP2 inducible kinase [Source:HGNC Symbol;Acc:HGNC:18041] | 3609 | 0.314 | 0.0664 | No |
| 25 | KDM7A | lysine demethylase 7A [Source:HGNC Symbol;Acc:HGNC:22224] | 3706 | 0.301 | 0.0662 | No |
| 26 | FN3K | fructosamine 3 kinase [Source:HGNC Symbol;Acc:HGNC:24822] | 3717 | 0.300 | 0.0715 | No |
| 27 | SDCBP | syndecan binding protein [Source:HGNC Symbol;Acc:HGNC:10662] | 3750 | 0.296 | 0.0753 | No |
| 28 | PICALM | phosphatidylinositol binding clathrin assembly protein [Source:HGNC Symbol;Acc:HGNC:15514] | 3844 | 0.286 | 0.0750 | No |
| 29 | ASNS | asparagine synthetase (glutamine-hydrolyzing) [Source:HGNC Symbol;Acc:HGNC:753] | 3870 | 0.282 | 0.0790 | No |
| 30 | KAT2B | lysine acetyltransferase 2B [Source:HGNC Symbol;Acc:HGNC:8638] | 3873 | 0.282 | 0.0844 | No |
| 31 | TNRC6B | trinucleotide repeat containing adaptor 6B [Source:HGNC Symbol;Acc:HGNC:29190] | 3888 | 0.280 | 0.0891 | No |
| 32 | DAAM1 | dishevelled associated activator of morphogenesis 1 [Source:HGNC Symbol;Acc:HGNC:18142] | 3964 | 0.270 | 0.0896 | No |
| 33 | KLF3 | Kruppel like factor 3 [Source:HGNC Symbol;Acc:HGNC:16516] | 4062 | 0.258 | 0.0884 | No |
| 34 | CCDC28A | coiled-coil domain containing 28A [Source:HGNC Symbol;Acc:HGNC:21098] | 4075 | 0.257 | 0.0928 | No |
| 35 | SLC22A4 | solute carrier family 22 member 4 [Source:HGNC Symbol;Acc:HGNC:10968] | 4373 | 0.225 | 0.0781 | No |
| 36 | TRAK2 | trafficking kinesin protein 2 [Source:HGNC Symbol;Acc:HGNC:13206] | 4392 | 0.223 | 0.0813 | No |
| 37 | OPTN | optineurin [Source:HGNC Symbol;Acc:HGNC:17142] | 4423 | 0.220 | 0.0837 | No |
| 38 | SLC66A2 | solute carrier family 66 member 2 [Source:HGNC Symbol;Acc:HGNC:26188] | 4593 | 0.205 | 0.0769 | No |
| 39 | DCAF11 | DDB1 and CUL4 associated factor 11 [Source:HGNC Symbol;Acc:HGNC:20258] | 4609 | 0.204 | 0.0800 | No |
| 40 | MOSPD1 | motile sperm domain containing 1 [Source:HGNC Symbol;Acc:HGNC:25235] | 4732 | 0.194 | 0.0759 | No |
| 41 | ERMAP | erythroblast membrane associated protein (Scianna blood group) [Source:HGNC Symbol;Acc:HGNC:15743] | 4735 | 0.193 | 0.0797 | No |
| 42 | DMTN | dematin actin binding protein [Source:HGNC Symbol;Acc:HGNC:3382] | 4792 | 0.188 | 0.0798 | No |
| 43 | SLC11A2 | solute carrier family 11 member 2 [Source:HGNC Symbol;Acc:HGNC:10908] | 4867 | 0.182 | 0.0786 | No |
| 44 | UROS | uroporphyrinogen III synthase [Source:HGNC Symbol;Acc:HGNC:12592] | 4869 | 0.182 | 0.0821 | No |
| 45 | FBXO34 | F-box protein 34 [Source:HGNC Symbol;Acc:HGNC:20201] | 5081 | 0.160 | 0.0717 | No |
| 46 | XPO7 | exportin 7 [Source:HGNC Symbol;Acc:HGNC:14108] | 5101 | 0.158 | 0.0736 | No |
| 47 | CDC27 | cell division cycle 27 [Source:HGNC Symbol;Acc:HGNC:1728] | 5109 | 0.157 | 0.0762 | No |
| 48 | FOXJ2 | forkhead box J2 [Source:HGNC Symbol;Acc:HGNC:24818] | 5209 | 0.150 | 0.0728 | No |
| 49 | UCP2 | uncoupling protein 2 [Source:HGNC Symbol;Acc:HGNC:12518] | 5351 | 0.139 | 0.0665 | No |
| 50 | HEBP1 | heme binding protein 1 [Source:HGNC Symbol;Acc:HGNC:17176] | 5530 | 0.125 | 0.0575 | No |
| 51 | SLC7A11 | solute carrier family 7 member 11 [Source:HGNC Symbol;Acc:HGNC:11059] | 5535 | 0.125 | 0.0597 | No |
| 52 | SELENBP1 | selenium binding protein 1 [Source:HGNC Symbol;Acc:HGNC:10719] | 5559 | 0.123 | 0.0606 | No |
| 53 | MFHAS1 | malignant fibrous histiocytoma amplified sequence 1 [Source:HGNC Symbol;Acc:HGNC:16982] | 5569 | 0.122 | 0.0625 | No |
| 54 | SIDT2 | SID1 transmembrane family member 2 [Source:HGNC Symbol;Acc:HGNC:24272] | 5583 | 0.121 | 0.0640 | No |
| 55 | ENDOD1 | endonuclease domain containing 1 [Source:HGNC Symbol;Acc:HGNC:29129] | 5807 | 0.105 | 0.0517 | No |
| 56 | NR3C1 | nuclear receptor subfamily 3 group C member 1 [Source:HGNC Symbol;Acc:HGNC:7978] | 5843 | 0.102 | 0.0515 | No |
| 57 | LPIN2 | lipin 2 [Source:HGNC Symbol;Acc:HGNC:14450] | 5857 | 0.102 | 0.0526 | No |
| 58 | USP15 | ubiquitin specific peptidase 15 [Source:HGNC Symbol;Acc:HGNC:12613] | 6213 | 0.076 | 0.0312 | No |
| 59 | TFRC | transferrin receptor [Source:HGNC Symbol;Acc:HGNC:11763] | 6327 | 0.068 | 0.0253 | No |
| 60 | TCEA1 | transcription elongation factor A1 [Source:HGNC Symbol;Acc:HGNC:11612] | 6416 | 0.061 | 0.0208 | No |
| 61 | BLVRA | biliverdin reductase A [Source:HGNC Symbol;Acc:HGNC:1062] | 6450 | 0.058 | 0.0198 | No |
| 62 | RAP1GAP | RAP1 GTPase activating protein [Source:HGNC Symbol;Acc:HGNC:9858] | 6458 | 0.057 | 0.0205 | No |
| 63 | CIR1 | "corepressor interacting with RBPJ, CIR1 [Source:HGNC Symbol;Acc:HGNC:24217]" | 6486 | 0.056 | 0.0199 | No |
| 64 | LAMP2 | lysosomal associated membrane protein 2 [Source:HGNC Symbol;Acc:HGNC:6501] | 7018 | 0.023 | -0.0140 | No |
| 65 | RANBP10 | RAN binding protein 10 [Source:HGNC Symbol;Acc:HGNC:29285] | 7035 | 0.022 | -0.0146 | No |
| 66 | LRP10 | LDL receptor related protein 10 [Source:HGNC Symbol;Acc:HGNC:14553] | 7063 | 0.020 | -0.0159 | No |
| 67 | ATG4A | autophagy related 4A cysteine peptidase [Source:HGNC Symbol;Acc:HGNC:16489] | 7084 | 0.019 | -0.0168 | No |
| 68 | DCAF10 | DDB1 and CUL4 associated factor 10 [Source:HGNC Symbol;Acc:HGNC:23686] | 7221 | 0.011 | -0.0254 | No |
| 69 | FECH | ferrochelatase [Source:HGNC Symbol;Acc:HGNC:3647] | 7305 | 0.006 | -0.0306 | No |
| 70 | GAPVD1 | GTPase activating protein and VPS9 domains 1 [Source:HGNC Symbol;Acc:HGNC:23375] | 7336 | 0.004 | -0.0325 | No |
| 71 | BLVRB | biliverdin reductase B [Source:HGNC Symbol;Acc:HGNC:1063] | 7346 | 0.004 | -0.0330 | No |
| 72 | NFE2 | "nuclear factor, erythroid 2 [Source:HGNC Symbol;Acc:HGNC:7780]" | 7372 | 0.002 | -0.0346 | No |
| 73 | ADD2 | adducin 2 [Source:HGNC Symbol;Acc:HGNC:244] | 7557 | -0.007 | -0.0463 | No |
| 74 | BNIP3L | BCL2 interacting protein 3 like [Source:HGNC Symbol;Acc:HGNC:1085] | 7600 | -0.009 | -0.0488 | No |
| 75 | EPB42 | erythrocyte membrane protein band 4.2 [Source:HGNC Symbol;Acc:HGNC:3381] | 7615 | -0.009 | -0.0495 | No |
| 76 | FBXO7 | F-box protein 7 [Source:HGNC Symbol;Acc:HGNC:13586] | 7634 | -0.010 | -0.0505 | No |
| 77 | ALAD | aminolevulinate dehydratase [Source:HGNC Symbol;Acc:HGNC:395] | 7699 | -0.014 | -0.0544 | No |
| 78 | ARHGEF12 | Rho guanine nucleotide exchange factor 12 [Source:HGNC Symbol;Acc:HGNC:14193] | 7717 | -0.014 | -0.0552 | No |
| 79 | PPOX | protoporphyrinogen oxidase [Source:HGNC Symbol;Acc:HGNC:9280] | 7861 | -0.021 | -0.0640 | No |
| 80 | RBM5 | RNA binding motif protein 5 [Source:HGNC Symbol;Acc:HGNC:9902] | 7906 | -0.024 | -0.0664 | No |
| 81 | GCLM | glutamate-cysteine ligase modifier subunit [Source:HGNC Symbol;Acc:HGNC:4312] | 7968 | -0.027 | -0.0698 | No |
| 82 | ACSL6 | acyl-CoA synthetase long chain family member 6 [Source:HGNC Symbol;Acc:HGNC:16496] | 7982 | -0.028 | -0.0701 | No |
| 83 | CTNS | "cystinosin, lysosomal cystine transporter [Source:HGNC Symbol;Acc:HGNC:2518]" | 8149 | -0.038 | -0.0800 | No |
| 84 | MAP2K3 | mitogen-activated protein kinase kinase 3 [Source:HGNC Symbol;Acc:HGNC:6843] | 8186 | -0.039 | -0.0816 | No |
| 85 | PSMD9 | "proteasome 26S subunit, non-ATPase 9 [Source:HGNC Symbol;Acc:HGNC:9567]" | 8216 | -0.041 | -0.0826 | No |
| 86 | ARL2BP | ADP ribosylation factor like GTPase 2 binding protein [Source:HGNC Symbol;Acc:HGNC:17146] | 8234 | -0.042 | -0.0829 | No |
| 87 | SPTA1 | "spectrin alpha, erythrocytic 1 [Source:HGNC Symbol;Acc:HGNC:11272]" | 8271 | -0.042 | -0.0844 | No |
| 88 | GYPC | glycophorin C (Gerbich blood group) [Source:HGNC Symbol;Acc:HGNC:4704] | 8394 | -0.048 | -0.0913 | No |
| 89 | MKRN1 | makorin ring finger protein 1 [Source:HGNC Symbol;Acc:HGNC:7112] | 8406 | -0.049 | -0.0910 | No |
| 90 | AQP3 | aquaporin 3 (Gill blood group) [Source:HGNC Symbol;Acc:HGNC:636] | 8455 | -0.053 | -0.0931 | No |
| 91 | ALDH1L1 | aldehyde dehydrogenase 1 family member L1 [Source:HGNC Symbol;Acc:HGNC:3978] | 8610 | -0.061 | -0.1018 | No |
| 92 | SLC25A38 | solute carrier family 25 member 38 [Source:HGNC Symbol;Acc:HGNC:26054] | 8691 | -0.066 | -0.1057 | No |
| 93 | CAST | calpastatin [Source:HGNC Symbol;Acc:HGNC:1515] | 8697 | -0.066 | -0.1047 | No |
| 94 | ISCA1 | iron-sulfur cluster assembly 1 [Source:HGNC Symbol;Acc:HGNC:28660] | 8713 | -0.068 | -0.1043 | No |
| 95 | RNF123 | ring finger protein 123 [Source:HGNC Symbol;Acc:HGNC:21148] | 8715 | -0.068 | -0.1031 | No |
| 96 | RNF19A | "ring finger protein 19A, RBR E3 ubiquitin protein ligase [Source:HGNC Symbol;Acc:HGNC:13432]" | 8746 | -0.069 | -0.1036 | No |
| 97 | CLCN3 | chloride voltage-gated channel 3 [Source:HGNC Symbol;Acc:HGNC:2021] | 8758 | -0.070 | -0.1029 | No |
| 98 | SLC6A9 | solute carrier family 6 member 9 [Source:HGNC Symbol;Acc:HGNC:11056] | 8874 | -0.077 | -0.1089 | No |
| 99 | PDZK1IP1 | PDZK1 interacting protein 1 [Source:HGNC Symbol;Acc:HGNC:16887] | 8899 | -0.078 | -0.1089 | No |
| 100 | UBAC1 | UBA domain containing 1 [Source:HGNC Symbol;Acc:HGNC:30221] | 9000 | -0.082 | -0.1137 | No |
| 101 | CAT | catalase [Source:HGNC Symbol;Acc:HGNC:1516] | 9069 | -0.086 | -0.1164 | No |
| 102 | MARK3 | microtubule affinity regulating kinase 3 [Source:HGNC Symbol;Acc:HGNC:6897] | 9124 | -0.089 | -0.1181 | No |
| 103 | EPB41 | erythrocyte membrane protein band 4.1 [Source:HGNC Symbol;Acc:HGNC:3377] | 9260 | -0.096 | -0.1249 | No |
| 104 | EIF2AK1 | eukaryotic translation initiation factor 2 alpha kinase 1 [Source:HGNC Symbol;Acc:HGNC:24921] | 9278 | -0.097 | -0.1241 | No |
| 105 | YPEL5 | yippee like 5 [Source:HGNC Symbol;Acc:HGNC:18329] | 9347 | -0.101 | -0.1265 | No |
| 106 | ADIPOR1 | adiponectin receptor 1 [Source:HGNC Symbol;Acc:HGNC:24040] | 9523 | -0.112 | -0.1355 | No |
| 107 | NEK7 | NIMA related kinase 7 [Source:HGNC Symbol;Acc:HGNC:13386] | 9706 | -0.125 | -0.1448 | No |
| 108 | EZH1 | enhancer of zeste 1 polycomb repressive complex 2 subunit [Source:HGNC Symbol;Acc:HGNC:3526] | 9755 | -0.128 | -0.1454 | No |
| 109 | GCLC | glutamate-cysteine ligase catalytic subunit [Source:HGNC Symbol;Acc:HGNC:4311] | 9762 | -0.129 | -0.1432 | No |
| 110 | TOP1 | DNA topoisomerase I [Source:HGNC Symbol;Acc:HGNC:11986] | 9863 | -0.134 | -0.1470 | No |
| 111 | DCUN1D1 | defective in cullin neddylation 1 domain containing 1 [Source:HGNC Symbol;Acc:HGNC:18184] | 9872 | -0.135 | -0.1448 | No |
| 112 | TAL1 | "TAL bHLH transcription factor 1, erythroid differentiation factor [Source:HGNC Symbol;Acc:HGNC:11556]" | 9886 | -0.136 | -0.1430 | No |
| 113 | MBOAT2 | membrane bound O-acyltransferase domain containing 2 [Source:HGNC Symbol;Acc:HGNC:25193] | 9929 | -0.138 | -0.1430 | No |
| 114 | PPP2R5B | protein phosphatase 2 regulatory subunit B'beta [Source:HGNC Symbol;Acc:HGNC:9310] | 10018 | -0.143 | -0.1458 | No |
| 115 | CPOX | coproporphyrinogen oxidase [Source:HGNC Symbol;Acc:HGNC:2321] | 10034 | -0.144 | -0.1439 | No |
| 116 | MARCHF8 | membrane associated ring-CH-type finger 8 [Source:HGNC Symbol;Acc:HGNC:23356] | 10338 | -0.162 | -0.1603 | No |
| 117 | GLRX5 | glutaredoxin 5 [Source:HGNC Symbol;Acc:HGNC:20134] | 10365 | -0.164 | -0.1587 | No |
| 118 | CCND3 | cyclin D3 [Source:HGNC Symbol;Acc:HGNC:1585] | 10446 | -0.168 | -0.1605 | No |
| 119 | GMPS | guanine monophosphate synthase [Source:HGNC Symbol;Acc:HGNC:4378] | 10459 | -0.169 | -0.1580 | No |
| 120 | HMBS | hydroxymethylbilane synthase [Source:HGNC Symbol;Acc:HGNC:4982] | 10518 | -0.173 | -0.1583 | No |
| 121 | RAD23A | "RAD23 homolog A, nucleotide excision repair protein [Source:HGNC Symbol;Acc:HGNC:9812]" | 10595 | -0.177 | -0.1597 | No |
| 122 | VEZF1 | vascular endothelial zinc finger 1 [Source:HGNC Symbol;Acc:HGNC:12949] | 10785 | -0.188 | -0.1682 | No |
| 123 | BTRC | beta-transducin repeat containing E3 ubiquitin protein ligase [Source:HGNC Symbol;Acc:HGNC:1144] | 10894 | -0.194 | -0.1713 | No |
| 124 | MGST3 | microsomal glutathione S-transferase 3 [Source:HGNC Symbol;Acc:HGNC:7064] | 10989 | -0.200 | -0.1734 | No |
| 125 | NUDT4 | nudix hydrolase 4 [Source:HGNC Symbol;Acc:HGNC:8051] | 10997 | -0.200 | -0.1699 | No |
| 126 | SEC14L1 | SEC14 like lipid binding 1 [Source:HGNC Symbol;Acc:HGNC:10698] | 11028 | -0.202 | -0.1679 | No |
| 127 | FBXO9 | F-box protein 9 [Source:HGNC Symbol;Acc:HGNC:13588] | 11051 | -0.203 | -0.1653 | No |
| 128 | TFDP2 | transcription factor Dp-2 [Source:HGNC Symbol;Acc:HGNC:11751] | 11175 | -0.210 | -0.1690 | No |
| 129 | ADD1 | adducin 1 [Source:HGNC Symbol;Acc:HGNC:243] | 11225 | -0.213 | -0.1680 | No |
| 130 | ANK1 | ankyrin 1 [Source:HGNC Symbol;Acc:HGNC:492] | 11593 | -0.235 | -0.1870 | No |
| 131 | ACP5 | "acid phosphatase 5, tartrate resistant [Source:HGNC Symbol;Acc:HGNC:124]" | 11597 | -0.235 | -0.1825 | No |
| 132 | TMEM9B | TMEM9 domain family member B [Source:HGNC Symbol;Acc:HGNC:1168] | 11678 | -0.239 | -0.1830 | No |
| 133 | RBM38 | RNA binding motif protein 38 [Source:HGNC Symbol;Acc:HGNC:15818] | 11686 | -0.240 | -0.1787 | No |
| 134 | ALDH6A1 | aldehyde dehydrogenase 6 family member A1 [Source:HGNC Symbol;Acc:HGNC:7179] | 11806 | -0.248 | -0.1814 | No |
| 135 | HBD | hemoglobin subunit delta [Source:HGNC Symbol;Acc:HGNC:4829] | 11916 | -0.254 | -0.1834 | No |
| 136 | GDE1 | glycerophosphodiester phosphodiesterase 1 [Source:HGNC Symbol;Acc:HGNC:29644] | 11947 | -0.257 | -0.1803 | No |
| 137 | CA1 | carbonic anhydrase 1 [Source:HGNC Symbol;Acc:HGNC:1368] | 11970 | -0.259 | -0.1766 | No |
| 138 | PIGQ | phosphatidylinositol glycan anchor biosynthesis class Q [Source:HGNC Symbol;Acc:HGNC:14135] | 12249 | -0.277 | -0.1890 | Yes |
| 139 | FOXO3 | forkhead box O3 [Source:HGNC Symbol;Acc:HGNC:3821] | 12304 | -0.281 | -0.1869 | Yes |
| 140 | ATP6V0A1 | ATPase H+ transporting V0 subunit a1 [Source:HGNC Symbol;Acc:HGNC:865] | 12334 | -0.283 | -0.1832 | Yes |
| 141 | MPP1 | membrane palmitoylated protein 1 [Source:HGNC Symbol;Acc:HGNC:7219] | 12348 | -0.284 | -0.1784 | Yes |
| 142 | NNT | nicotinamide nucleotide transhydrogenase [Source:HGNC Symbol;Acc:HGNC:7863] | 12374 | -0.286 | -0.1743 | Yes |
| 143 | AGPAT4 | 1-acylglycerol-3-phosphate O-acyltransferase 4 [Source:HGNC Symbol;Acc:HGNC:20885] | 12505 | -0.296 | -0.1769 | Yes |
| 144 | HDGF | heparin binding growth factor [Source:HGNC Symbol;Acc:HGNC:4856] | 12538 | -0.299 | -0.1730 | Yes |
| 145 | BSG | basigin (Ok blood group) [Source:HGNC Symbol;Acc:HGNC:1116] | 12543 | -0.300 | -0.1673 | Yes |
| 146 | HTRA2 | HtrA serine peptidase 2 [Source:HGNC Symbol;Acc:HGNC:14348] | 12571 | -0.302 | -0.1630 | Yes |
| 147 | SLC6A8 | solute carrier family 6 member 8 [Source:HGNC Symbol;Acc:HGNC:11055] | 12660 | -0.308 | -0.1626 | Yes |
| 148 | ALAS2 | 5'-aminolevulinate synthase 2 [Source:HGNC Symbol;Acc:HGNC:397] | 12662 | -0.309 | -0.1566 | Yes |
| 149 | MARCHF2 | membrane associated ring-CH-type finger 2 [Source:HGNC Symbol;Acc:HGNC:28038] | 12799 | -0.319 | -0.1590 | Yes |
| 150 | SLC4A1 | solute carrier family 4 member 1 (Diego blood group) [Source:HGNC Symbol;Acc:HGNC:11027] | 13047 | -0.343 | -0.1682 | Yes |
| 151 | KLF1 | Kruppel like factor 1 [Source:HGNC Symbol;Acc:HGNC:6345] | 13121 | -0.349 | -0.1660 | Yes |
| 152 | ABCB6 | ATP binding cassette subfamily B member 6 (Langereis blood group) [Source:HGNC Symbol;Acc:HGNC:47] | 13232 | -0.359 | -0.1659 | Yes |
| 153 | NARF | nuclear prelamin A recognition factor [Source:HGNC Symbol;Acc:HGNC:29916] | 13392 | -0.378 | -0.1687 | Yes |
| 154 | PGLS | 6-phosphogluconolactonase [Source:HGNC Symbol;Acc:HGNC:8903] | 13415 | -0.380 | -0.1626 | Yes |
| 155 | OSBP2 | oxysterol binding protein 2 [Source:HGNC Symbol;Acc:HGNC:8504] | 13433 | -0.383 | -0.1561 | Yes |
| 156 | BCAM | basal cell adhesion molecule (Lutheran blood group) [Source:HGNC Symbol;Acc:HGNC:6722] | 13487 | -0.391 | -0.1518 | Yes |
| 157 | P4HA2 | prolyl 4-hydroxylase subunit alpha 2 [Source:HGNC Symbol;Acc:HGNC:8547] | 13502 | -0.392 | -0.1449 | Yes |
| 158 | TSPAN5 | tetraspanin 5 [Source:HGNC Symbol;Acc:HGNC:17753] | 13505 | -0.393 | -0.1372 | Yes |
| 159 | UROD | uroporphyrinogen decarboxylase [Source:HGNC Symbol;Acc:HGNC:12591] | 13545 | -0.398 | -0.1319 | Yes |
| 160 | EPOR | erythropoietin receptor [Source:HGNC Symbol;Acc:HGNC:3416] | 13559 | -0.400 | -0.1248 | Yes |
| 161 | HTATIP2 | HIV-1 Tat interactive protein 2 [Source:HGNC Symbol;Acc:HGNC:16637] | 13612 | -0.407 | -0.1201 | Yes |
| 162 | GYPA | glycophorin A (MNS blood group) [Source:HGNC Symbol;Acc:HGNC:4702] | 13621 | -0.408 | -0.1125 | Yes |
| 163 | NFE2L1 | "nuclear factor, erythroid 2 like 1 [Source:HGNC Symbol;Acc:HGNC:7781]" | 13623 | -0.408 | -0.1045 | Yes |
| 164 | MINPP1 | multiple inositol-polyphosphate phosphatase 1 [Source:HGNC Symbol;Acc:HGNC:7102] | 13630 | -0.409 | -0.0968 | Yes |
| 165 | SNCA | synuclein alpha [Source:HGNC Symbol;Acc:HGNC:11138] | 13646 | -0.410 | -0.0896 | Yes |
| 166 | HAGH | hydroxyacylglutathione hydrolase [Source:HGNC Symbol;Acc:HGNC:4805] | 13779 | -0.416 | -0.0899 | Yes |
| 167 | BPGM | bisphosphoglycerate mutase [Source:HGNC Symbol;Acc:HGNC:1093] | 14058 | -0.441 | -0.0991 | Yes |
| 168 | CDR2 | cerebellar degeneration related protein 2 [Source:HGNC Symbol;Acc:HGNC:1799] | 14064 | -0.441 | -0.0907 | Yes |
| 169 | IGSF3 | immunoglobulin superfamily member 3 [Source:HGNC Symbol;Acc:HGNC:5950] | 14129 | -0.453 | -0.0858 | Yes |
| 170 | PRDX2 | peroxiredoxin 2 [Source:HGNC Symbol;Acc:HGNC:9353] | 14285 | -0.480 | -0.0863 | Yes |
| 171 | TNS1 | tensin 1 [Source:HGNC Symbol;Acc:HGNC:11973] | 14358 | -0.488 | -0.0813 | Yes |
| 172 | RHAG | Rh associated glycoprotein [Source:HGNC Symbol;Acc:HGNC:10006] | 14375 | -0.490 | -0.0726 | Yes |
| 173 | FTCD | formimidoyltransferase cyclodeaminase [Source:HGNC Symbol;Acc:HGNC:3974] | 14461 | -0.505 | -0.0681 | Yes |
| 174 | PC | pyruvate carboxylase [Source:HGNC Symbol;Acc:HGNC:8636] | 14499 | -0.513 | -0.0603 | Yes |
| 175 | TRIM10 | tripartite motif containing 10 [Source:HGNC Symbol;Acc:HGNC:10072] | 14549 | -0.524 | -0.0531 | Yes |
| 176 | TMCC2 | transmembrane and coiled-coil domain family 2 [Source:HGNC Symbol;Acc:HGNC:24239] | 14575 | -0.528 | -0.0442 | Yes |
| 177 | H1-0 | H1.0 linker histone [Source:HGNC Symbol;Acc:HGNC:4714] | 14582 | -0.529 | -0.0341 | Yes |
| 178 | MXI1 | "MAX interactor 1, dimerization protein [Source:HGNC Symbol;Acc:HGNC:7534]" | 14822 | -0.589 | -0.0379 | Yes |
| 179 | RHD | Rh blood group D antigen [Source:HGNC Symbol;Acc:HGNC:10009] | 14840 | -0.596 | -0.0271 | Yes |
| 180 | SLC2A1 | solute carrier family 2 member 1 [Source:HGNC Symbol;Acc:HGNC:11005] | 14992 | -0.661 | -0.0238 | Yes |
| 181 | SLC10A3 | solute carrier family 10 member 3 [Source:HGNC Symbol;Acc:HGNC:22979] | 15019 | -0.671 | -0.0122 | Yes |
| 182 | RCL1 | RNA terminal phosphate cyclase like 1 [Source:HGNC Symbol;Acc:HGNC:17687] | 15178 | -0.764 | -0.0072 | Yes |
| 183 | HBQ1 | hemoglobin subunit theta 1 [Source:HGNC Symbol;Acc:HGNC:4833] | 15364 | -0.866 | -0.0020 | Yes |
| 184 | CA2 | carbonic anhydrase 2 [Source:HGNC Symbol;Acc:HGNC:1373] | 15522 | -1.087 | 0.0094 | Yes |
Table: GSEA details [plain text format]

  

Fig 2: HALLMARK\_HEME\_METABOLISM      
 Blue-Pink O' Gram in the Space of the Analyzed GeneSet

  

Fig 3: HALLMARK\_HEME\_METABOLISM: Random ES distribution      
 Gene set null distribution of ES for **HALLMARK\_HEME\_METABOLISM**
