# Supplementary material for: In vivo CRISPR screens reveal Serpinb9 and Adam2 as regulators of immune therapy response in lung cancer
Source: Nat Commun. 2023 May 31;14:3150. doi: 10.1038/s41467-023-38841-7 (PMC10232477; doi:10.1038/s41467-023-38841-7)
Supplement: Supplementary file 13 — Source Data [file 41467_2023_38841_MOESM13_ESM.zip › Figure 4E.zip!/my_analysis.Gsea.1640146340255_Hallmark/gsea_report_for_CTLR_NI_1640146340255.html]

Report for CTLR\_NI 1640146340255 [GSEA]

| GS  follow link to MSigDB | GS DETAILS | SIZE | ES | NES | NOM p-val | FDR q-val | FWER p-val | RANK AT MAX | LEADING EDGE || 1 | HALLMARK\_ALLOGRAFT\_REJECTION | Details ... | 191 | 0.67 | 1.64 | 0.000 | 0.429 | 0.181 | 2102 | tags=50%, list=13%, signal=57% |
| 2 | HALLMARK\_INFLAMMATORY\_RESPONSE | Details ... | 191 | 0.59 | 1.62 | 0.000 | 0.269 | 0.286 | 1433 | tags=35%, list=9%, signal=38% |
| 3 | HALLMARK\_INTERFERON\_GAMMA\_RESPONSE | Details ... | 194 | 0.76 | 1.57 | 0.000 | 0.248 | 0.336 | 1725 | tags=56%, list=11%, signal=62% |
| 4 | HALLMARK\_INTERFERON\_ALPHA\_RESPONSE | Details ... | 94 | 0.79 | 1.57 | 0.000 | 0.198 | 0.336 | 1075 | tags=57%, list=7%, signal=61% |
| 5 | HALLMARK\_IL6\_JAK\_STAT3\_SIGNALING | Details ... | 86 | 0.65 | 1.56 | 0.000 | 0.181 | 0.336 | 1913 | tags=42%, list=12%, signal=47% |
| 6 | HALLMARK\_IL2\_STAT5\_SIGNALING | Details ... | 197 | 0.47 | 1.56 | 0.000 | 0.159 | 0.336 | 1956 | tags=28%, list=12%, signal=31% |
| 7 | HALLMARK\_COMPLEMENT | Details ... | 187 | 0.44 | 1.52 | 0.000 | 0.188 | 0.449 | 2429 | tags=30%, list=16%, signal=35% |
| 8 | HALLMARK\_TNFA\_SIGNALING\_VIA\_NFKB | Details ... | 197 | 0.43 | 1.43 | 0.097 | 0.275 | 0.698 | 2158 | tags=26%, list=14%, signal=30% |
| 9 | HALLMARK\_APOPTOSIS | Details ... | 158 | 0.34 | 1.39 | 0.000 | 0.290 | 0.748 | 1993 | tags=20%, list=13%, signal=22% |
| 10 | HALLMARK\_KRAS\_SIGNALING\_UP | Details ... | 192 | 0.43 | 1.38 | 0.000 | 0.291 | 0.802 | 1988 | tags=28%, list=13%, signal=32% |
| 11 | HALLMARK\_COAGULATION | Details ... | 123 | 0.38 | 1.35 | 0.000 | 0.310 | 0.844 | 2464 | tags=26%, list=16%, signal=31% |
| 12 | HALLMARK\_PI3K\_AKT\_MTOR\_SIGNALING | Details ... | 104 | 0.28 | 1.23 | 0.122 | 0.486 | 1.000 | 1490 | tags=12%, list=10%, signal=13% |
| 13 | HALLMARK\_SPERMATOGENESIS | Details ... | 115 | 0.32 | 1.17 | 0.108 | 0.623 | 1.000 | 3358 | tags=32%, list=21%, signal=41% |
| 14 | HALLMARK\_XENOBIOTIC\_METABOLISM | Details ... | 179 | 0.31 | 1.15 | 0.326 | 0.648 | 1.000 | 2578 | tags=23%, list=16%, signal=27% |
| 15 | HALLMARK\_BILE\_ACID\_METABOLISM | Details ... | 100 | 0.30 | 1.13 | 0.114 | 0.632 | 1.000 | 2182 | tags=22%, list=14%, signal=25% |
| 16 | HALLMARK\_ESTROGEN\_RESPONSE\_LATE | Details ... | 185 | 0.31 | 1.07 | 0.338 | 0.755 | 1.000 | 2557 | tags=23%, list=16%, signal=27% |
| 17 | HALLMARK\_ANDROGEN\_RESPONSE | Details ... | 95 | 0.26 | 1.07 | 0.437 | 0.724 | 1.000 | 3729 | tags=23%, list=24%, signal=30% |
| 18 | HALLMARK\_HEDGEHOG\_SIGNALING | Details ... | 33 | 0.36 | 1.06 | 0.301 | 0.698 | 1.000 | 3538 | tags=27%, list=23%, signal=35% |
| 19 | HALLMARK\_EPITHELIAL\_MESENCHYMAL\_TRANSITION | Details ... | 193 | 0.25 | 1.04 | 0.293 | 0.705 | 1.000 | 2391 | tags=21%, list=15%, signal=24% |
| 20 | HALLMARK\_APICAL\_JUNCTION | Details ... | 188 | 0.24 | 1.00 | 0.383 | 0.799 | 1.000 | 2757 | tags=21%, list=18%, signal=25% |
| 21 | HALLMARK\_MYOGENESIS |  | 199 | 0.33 | 0.97 | 0.418 | 0.809 | 1.000 | 3117 | tags=32%, list=20%, signal=40% |
| 22 | HALLMARK\_PANCREAS\_BETA\_CELLS |  | 28 | 0.33 | 0.96 | 0.508 | 0.794 | 1.000 | 3196 | tags=36%, list=20%, signal=45% |
| 23 | HALLMARK\_ESTROGEN\_RESPONSE\_EARLY |  | 191 | 0.26 | 0.93 | 0.624 | 0.816 | 1.000 | 2548 | tags=21%, list=16%, signal=25% |
| 24 | HALLMARK\_KRAS\_SIGNALING\_DN |  | 158 | 0.28 | 0.91 | 0.613 | 0.819 | 1.000 | 3595 | tags=35%, list=23%, signal=46% |
| 25 | HALLMARK\_HYPOXIA |  | 193 | 0.23 | 0.90 | 0.702 | 0.801 | 1.000 | 2401 | tags=18%, list=15%, signal=21% |
| 26 | HALLMARK\_UV\_RESPONSE\_UP |  | 152 | 0.21 | 0.88 | 0.624 | 0.790 | 1.000 | 3006 | tags=18%, list=19%, signal=23% |
| 27 | HALLMARK\_REACTIVE\_OXYGEN\_SPECIES\_PATHWAY |  | 49 | 0.21 | 0.88 | 0.501 | 0.772 | 1.000 | 2343 | tags=12%, list=15%, signal=14% |
| 28 | HALLMARK\_FATTY\_ACID\_METABOLISM |  | 149 | 0.21 | 0.87 | 0.726 | 0.768 | 1.000 | 1842 | tags=11%, list=12%, signal=13% |
| 29 | HALLMARK\_G2M\_CHECKPOINT |  | 196 | 0.23 | 0.82 | 0.584 | 0.824 | 1.000 | 5992 | tags=46%, list=38%, signal=74% |
| 30 | HALLMARK\_MITOTIC\_SPINDLE |  | 199 | 0.22 | 0.79 | 0.712 | 0.876 | 1.000 | 5109 | tags=32%, list=33%, signal=46% |
| 31 | HALLMARK\_ADIPOGENESIS |  | 200 | 0.16 | 0.68 | 0.712 | 0.967 | 1.000 | 2283 | tags=12%, list=15%, signal=14% |
| 32 | HALLMARK\_CHOLESTEROL\_HOMEOSTASIS |  | 71 | 0.23 | 0.68 | 0.618 | 0.940 | 1.000 | 1821 | tags=14%, list=12%, signal=16% |
| 33 | HALLMARK\_E2F\_TARGETS |  | 200 | 0.13 | 0.48 | 0.775 | 0.986 | 1.000 | 5698 | tags=36%, list=36%, signal=55% |
Table: Gene sets enriched in phenotype **CTLR\_NI (3 samples) **[plain text format]****
