# Supplementary material for: In vivo CRISPR screens reveal Serpinb9 and Adam2 as regulators of immune therapy response in lung cancer
Source: Nat Commun. 2023 May 31;14:3150. doi: 10.1038/s41467-023-38841-7 (PMC10232477; doi:10.1038/s41467-023-38841-7)
Supplement: Supplementary file 13 — Source Data [file 41467_2023_38841_MOESM13_ESM.zip › Figure 3A,B.zip!/Lungs_sgRNA analysis_KRAS /FluteRRA_screen1lung.pdf]

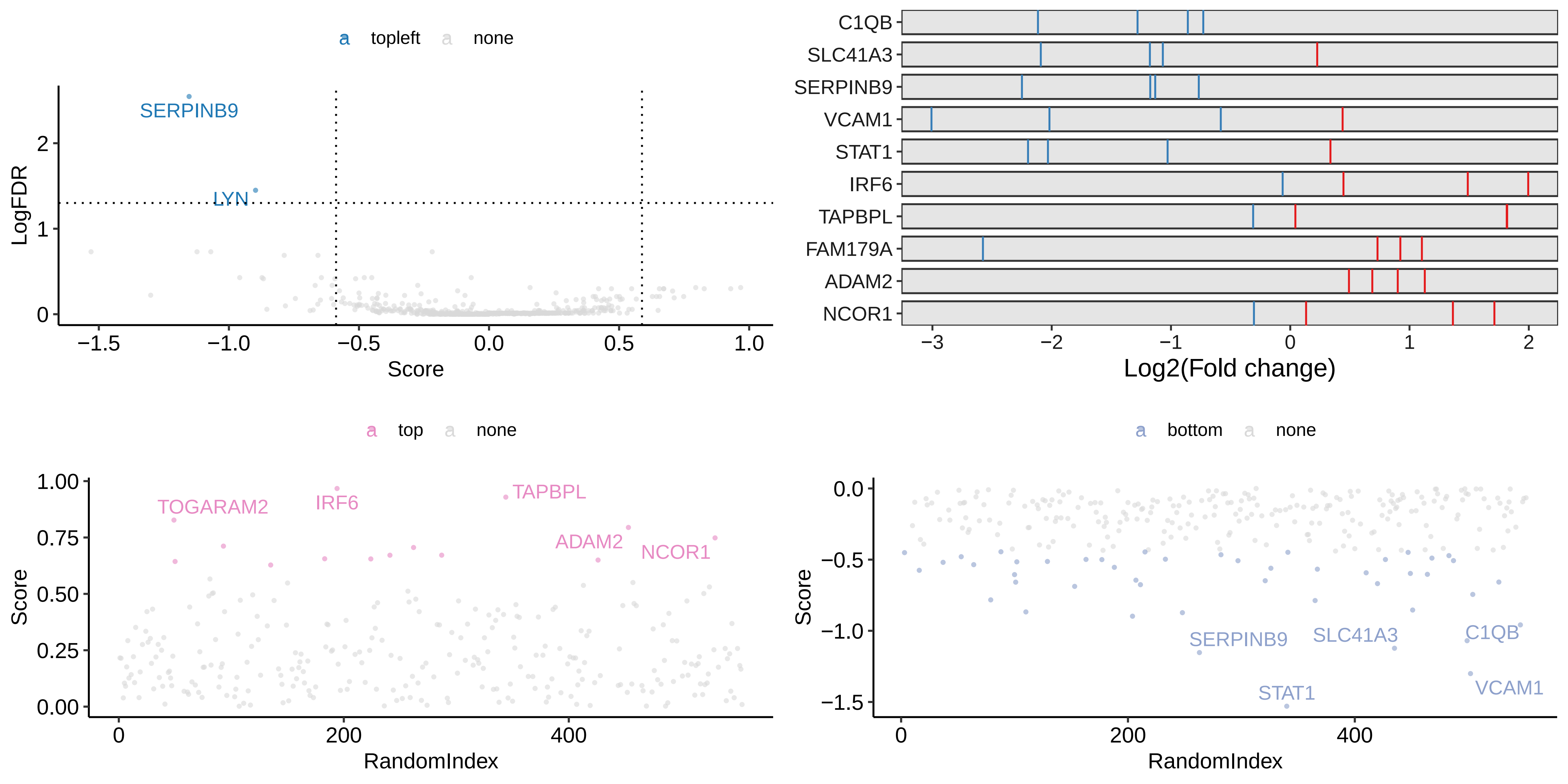

a topleft a none
SERPINB9
LYN
LogFDR
Score
−1.5 −1.0 −0.5 0.0 0.5 1.0
0 1 2
C1QB
SLC41A3
SERPINB9
VCAM1
STAT1
IRF6
TAPBPL
FAM179A
ADAM2
NCOR1
−3 −2 −1 0 1 2
Log2(Fold change)
a top a none
TOGARAM2
IRF6
TAPBPL
ADAM2
NCOR1
Score
0.00 0.25 0.50 0.75 1.00
RandomIndex
0 200 400
a bottom a none
SERPINB9
SLC41A3
C1QB
STAT1
VCAM1
Score
−1.5 −1.0 −0.5 0.0
RandomIndex
0 200 400

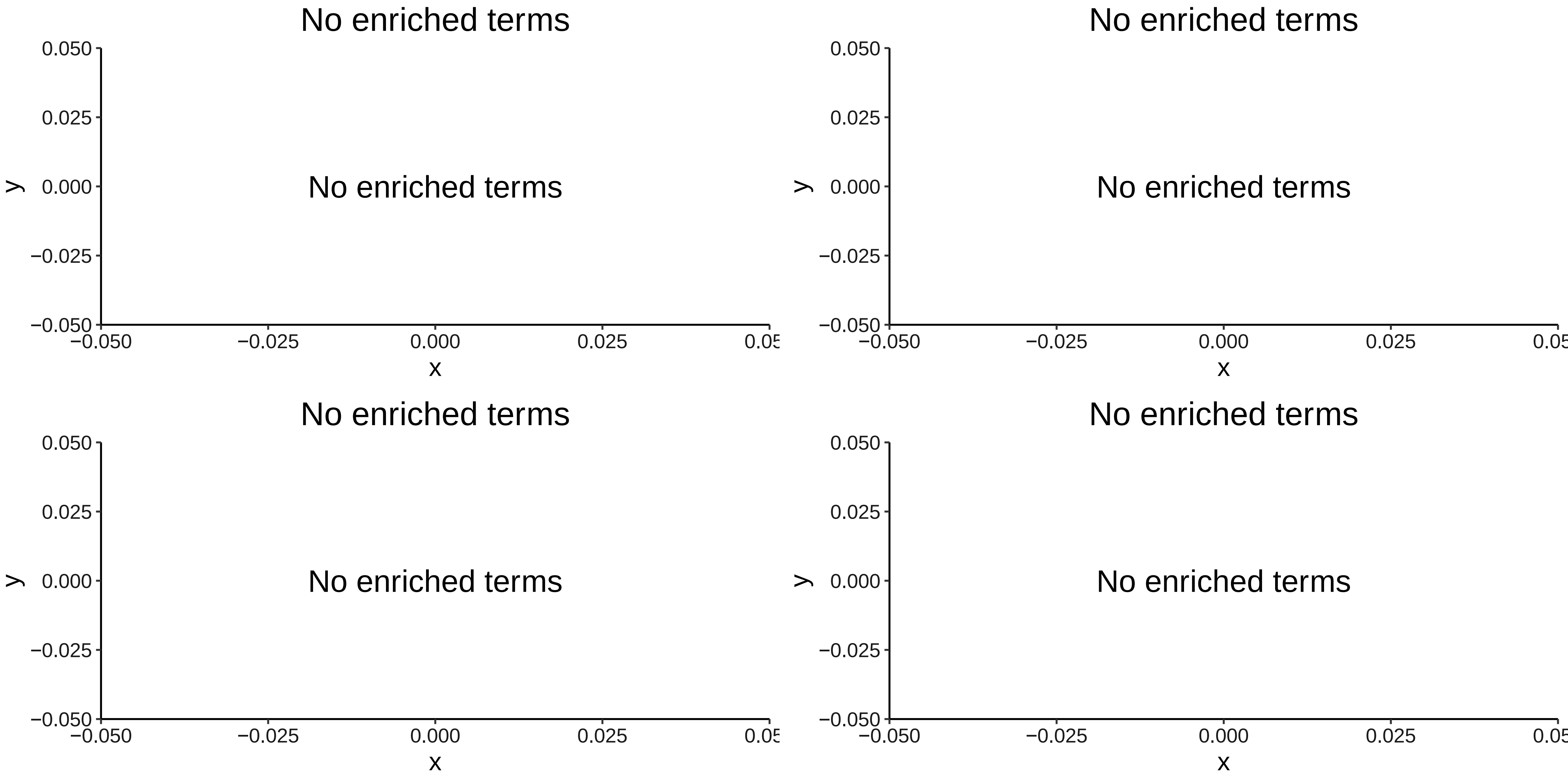
No enriched terms
0.050
0.025
0.000
−0.025
−0.050
y
No enriched terms
−0.050
−0.025
0.000
0.025
x
No enriched terms
0.050
0.025
0.000
−0.025
−0.050
y
No enriched terms
−0.050
−0.025
0.000
0.025
x
No enriched terms
0.050
0.025
0.000
−0.025
−0.050
y
No enriched terms
−0.050
−0.025
0.000
0.025
x
No enriched terms
0.050
0.025
0.000
−0.025
−0.050
y
No enriched terms
−0.050
−0.025
0.000
0.025
x

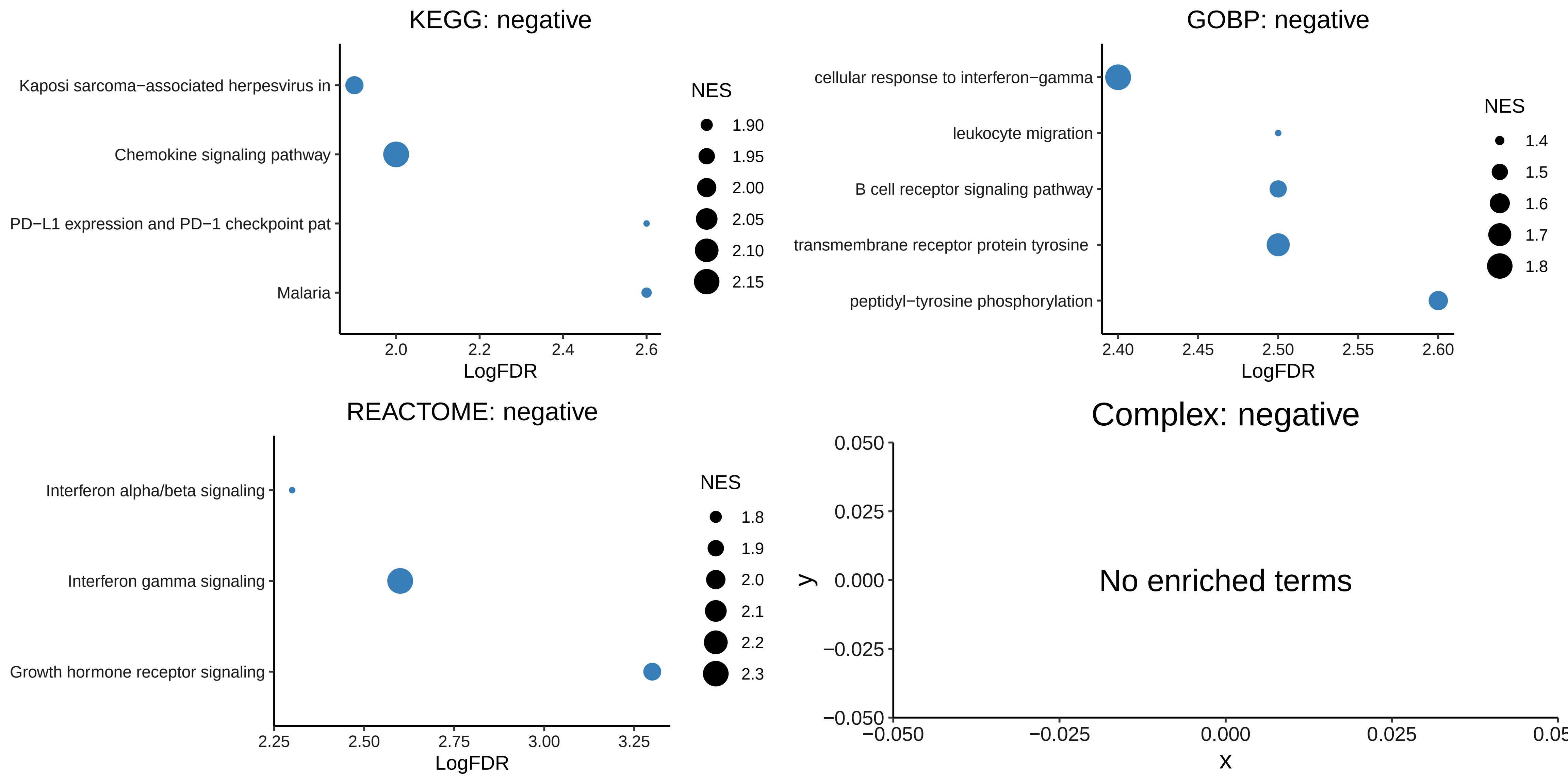

KEGG: negative
Kaposi sarcoma−associated herpesvirus in
Chemokine signaling pathway
PD−L1 expression and PD−1 checkpoint pat
Malaria
2.0
2.2
2.4
2.6
LogFDR
NES
1.90
1.95
2.00
2.05
2.10
2.15
GOBP: negative
cellular response to interferon−gamma
leukocyte migration
B cell receptor signaling pathway
transmembrane receptor protein tyrosine
peptidyl−tyrosine phosphorylation
2.40
2.45
2.50
2.55
2.60
LogFDR
NES
1.4
1.5
1.6
1.7
1.8
REACTOME: negative
Interferon alpha/beta signaling
Interferon gamma signaling
Growth hormone receptor signaling
2.25
2.50
2.75
3.00
3.25
LogFDR
NES
1.8
1.9
2.0
2.1
2.2
2.3
Complex: negative
No enriched terms
y
0.050
0.025
0.000
−0.025
−0.050
−0.050
−0.025
0.000
0.025
x

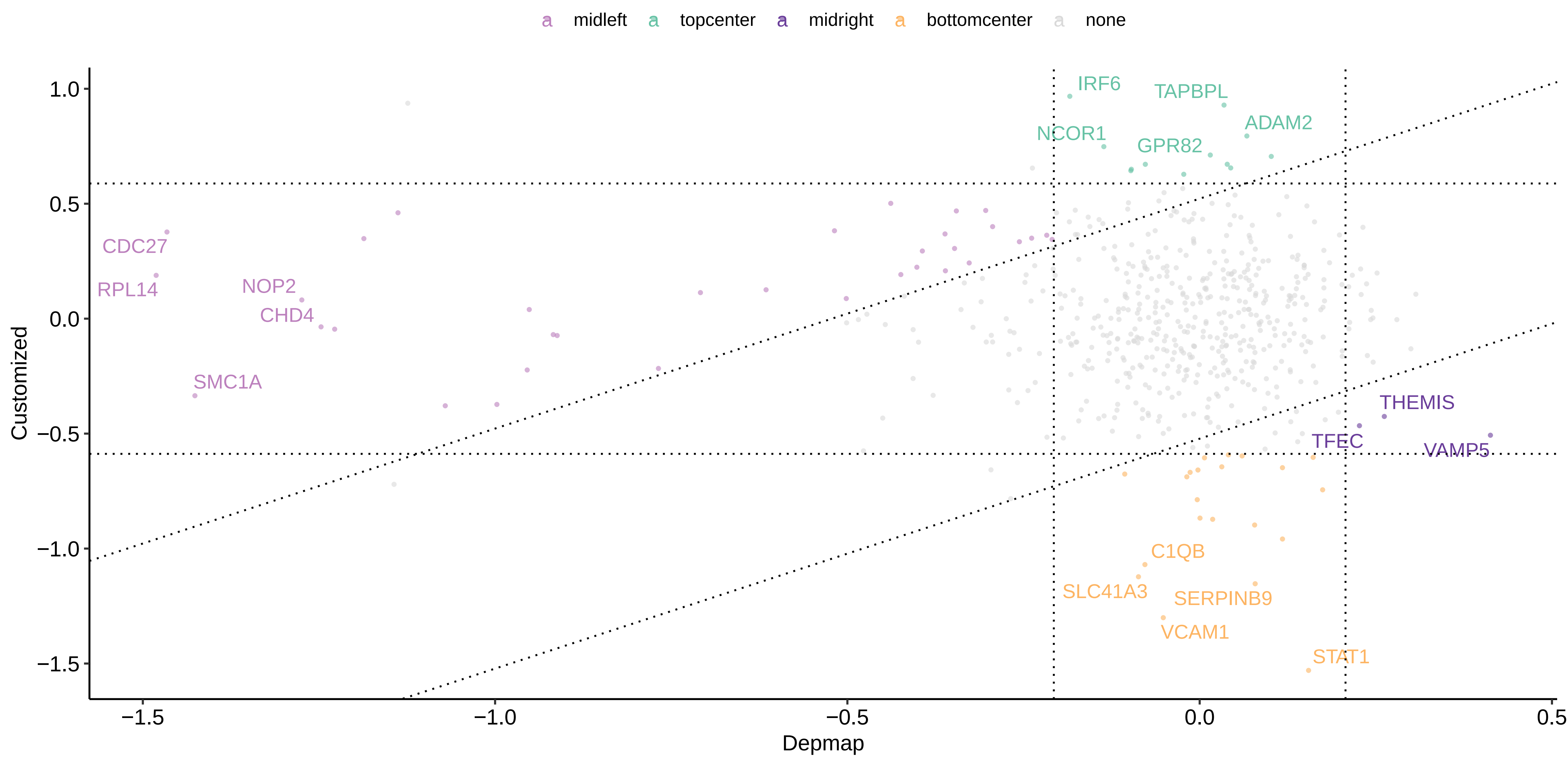

midleft
topcenter
midright
bottomcenter
none
1.0
0.5
0.0
−0.5
−1.0
−1.5
Customized
−1.5
−1.0
−0.5
0.0
0.5
Depmap
IRF6
TAPBPL
ADAM2
NCOR1
GPR82
CDC27
RPL14
NOP2
CHD4
SMC1A
THEMIS
TFEC
VAMP5
C1QB
SLC41A3
SERPINB9
VCAM1
STAT1

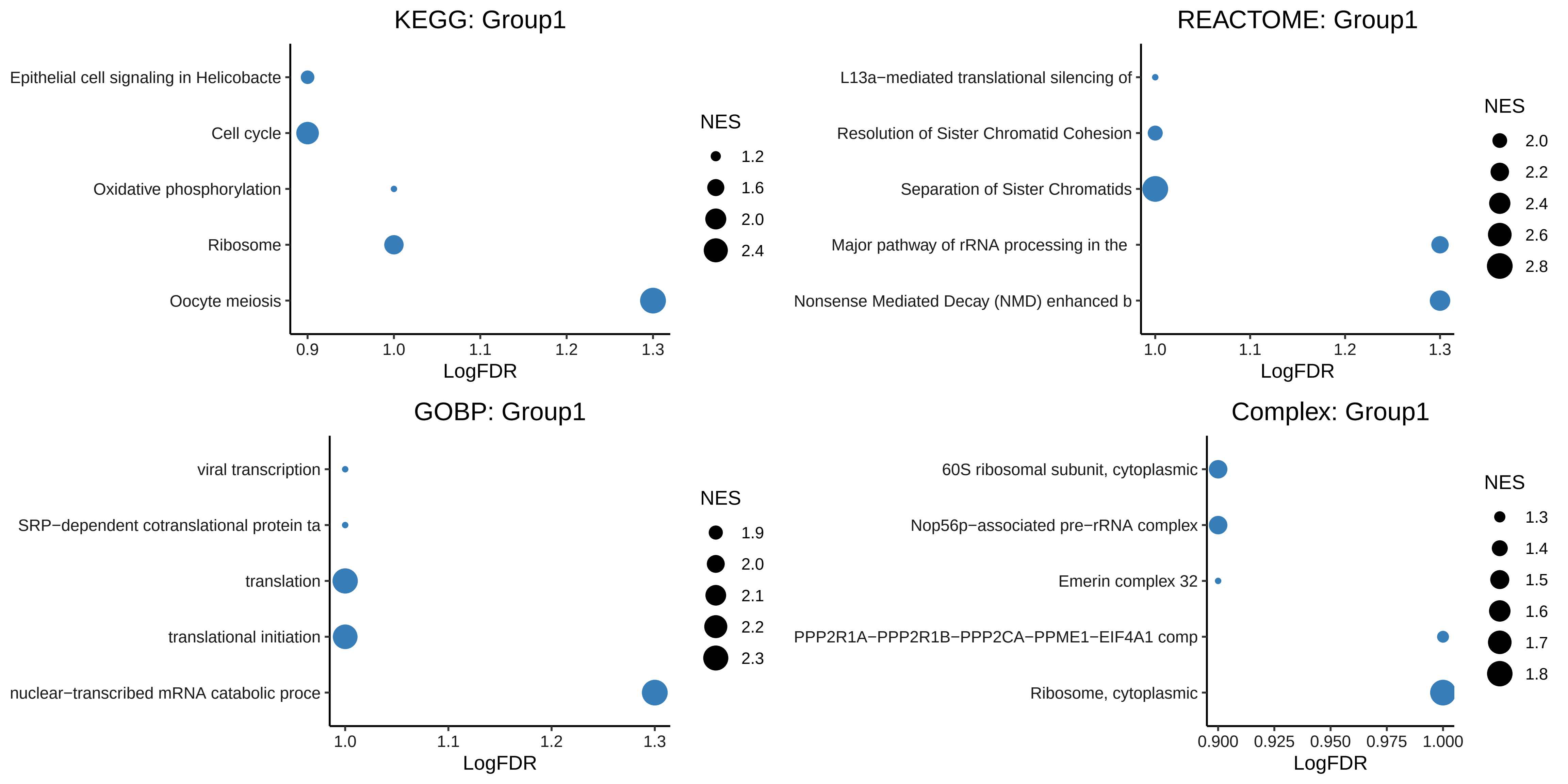
KEGG: Group1
Epithelial cell signaling in Helicobacte
Cell cycle
Oxidative phosphorylation
Ribosome
Oocyte meiosis
0.9
1.0
1.1
1.2
1.3
LogFDR
NES
1.2
1.6
2.0
2.4
REACTOME: Group1
L13a−mediated translational silencing of
Resolution of Sister Chromatid Cohesion
Separation of Sister Chromatids
Major pathway of rRNA processing in the
Nonsense Mediated Decay (NMD) enhanced b
1.0
1.1
1.2
1.3
LogFDR
NES
2.0
2.2
2.4
2.6
2.8
GOBP: Group1
viral transcription
SRP−dependent cotranslational protein ta
translation
translational initiation
nuclear−transcribed mRNA catabolic proce
1.0
1.1
1.2
1.3
LogFDR
NES
1.9
2.0
2.1
2.2
2.3
Complex: Group1
60S ribosomal subunit, cytoplasmic
Nop56p−associated pre−rRNA complex
Emerin complex 32
PPP2R1A−PPP2R1B−PPP2CA−PPME1−EIF4A1 comp
Ribosome, cytoplasmic
0.900
0.925
0.950
0.975
1.000
LogFDR
NES
1.3
1.4
1.5
1.6
1.7
1.8

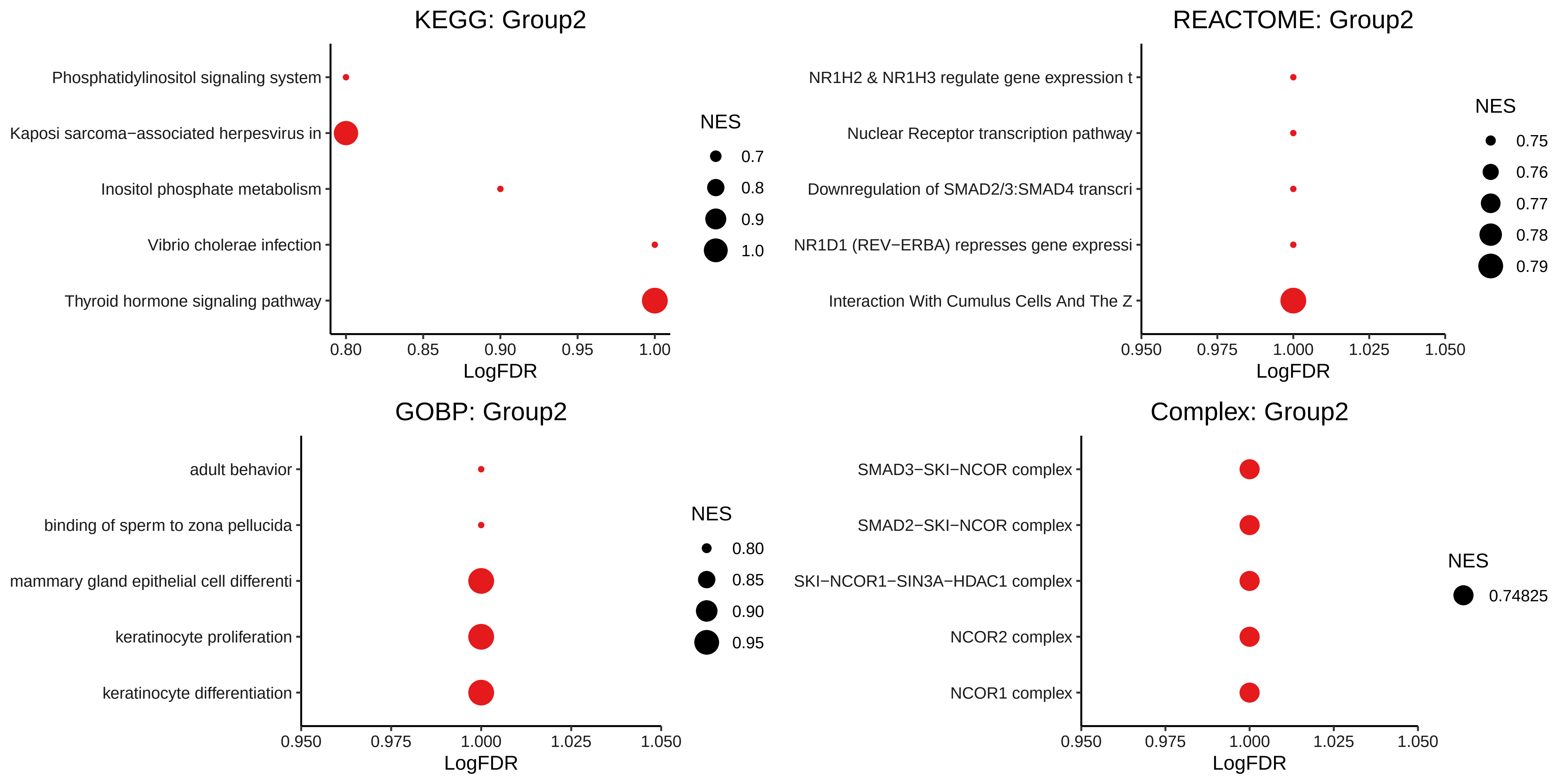

KEGG: Group2
Phosphatidylinositol signaling system
Kaposi sarcoma−associated herpesvirus in
Inositol phosphate metabolism
Vibrio cholerae infection
Thyroid hormone signaling pathway
0.80
0.85
0.90
0.95
1.00
LogFDR
NES
0.7
0.8
0.9
1.0
REACTOME: Group2
NR1H2 & NR1H3 regulate gene expression t
Nuclear Receptor transcription pathway
Downregulation of SMAD2/3:SMAD4 transcri
NR1D1 (REV−ERBA) represses gene expressi
Interaction With Cumulus Cells And The Z
0.950
0.975
1.000
1.025
1.050
LogFDR
NES
0.75
0.76
0.77
0.78
0.79
GOBP: Group2
adult behavior
binding of sperm to zona pellucida
mammary gland epithelial cell differenti
keratinocyte proliferation
keratinocyte differentiation
0.950
0.975
1.000
1.025
1.050
LogFDR
NES
0.80
0.85
0.90
0.95
Complex: Group2
SMAD3−SKI−NCOR complex
SMAD2−SKI−NCOR complex
SKI−NCOR1−SIN3A−HDAC1 complex
NCOR2 complex
NCOR1 complex
0.950
0.975
1.000
1.025
1.050
LogFDR
NES
0.74825

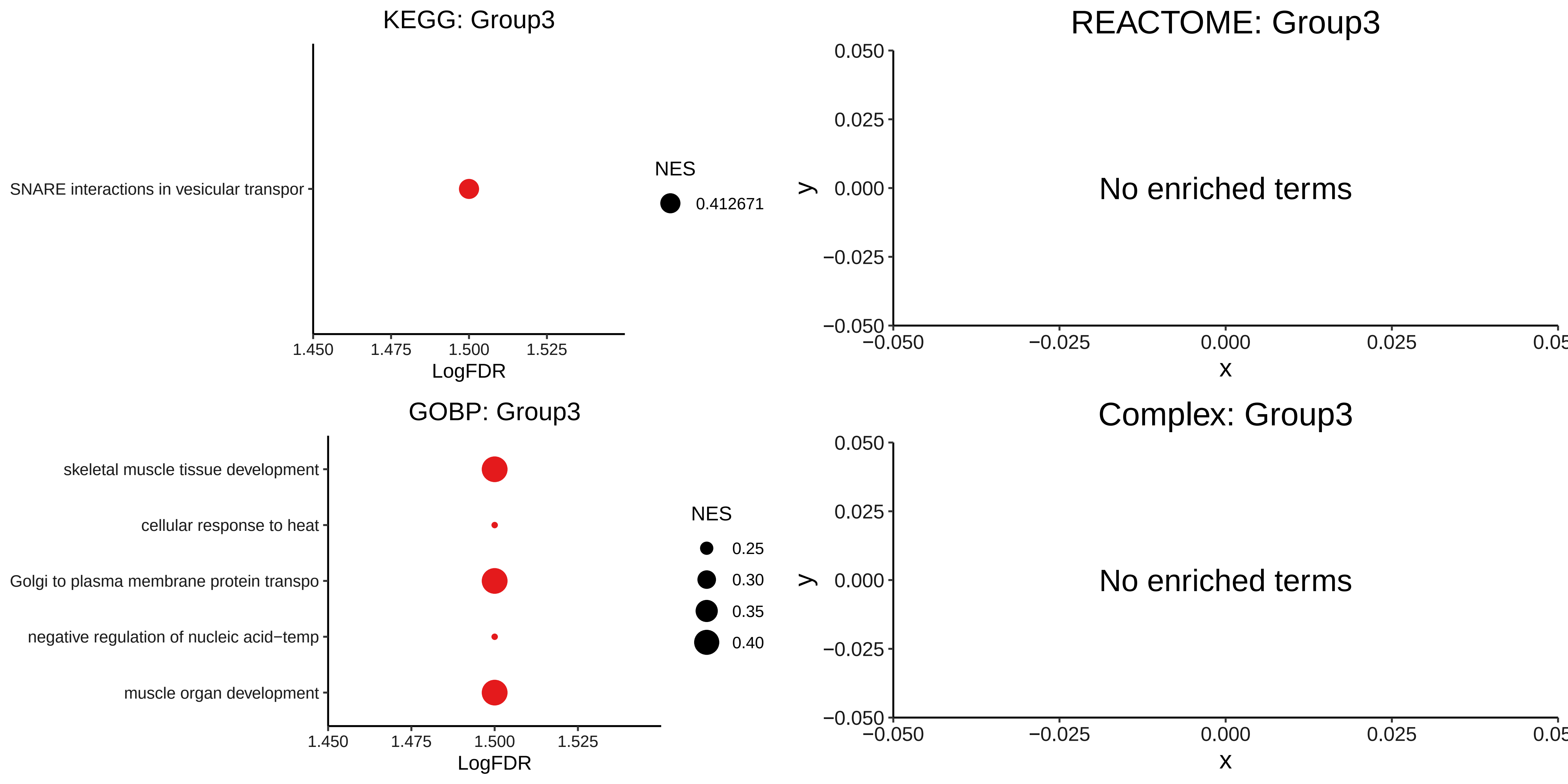
KEGG: Group3
SNARE interactions in vesicular transpor
1.450
1.475
1.500
1.525
LogFDR
NES
0.412671
REACTOME: Group3
0.050
0.025
0.000
−0.025
−0.050
y
No enriched terms
−0.050
−0.025
0.000
0.025
x
GOBP: Group3
skeletal muscle tissue development
cellular response to heat
Golgi to plasma membrane protein transpo
negative regulation of nucleic acid−temp
muscle organ development
1.450
1.475
1.500
1.525
LogFDR
NES
0.25
0.30
0.35
0.40
Complex: Group3
0.050
0.025
0.000
−0.025
−0.050
y
No enriched terms
−0.050
−0.025
0.000
0.025
x

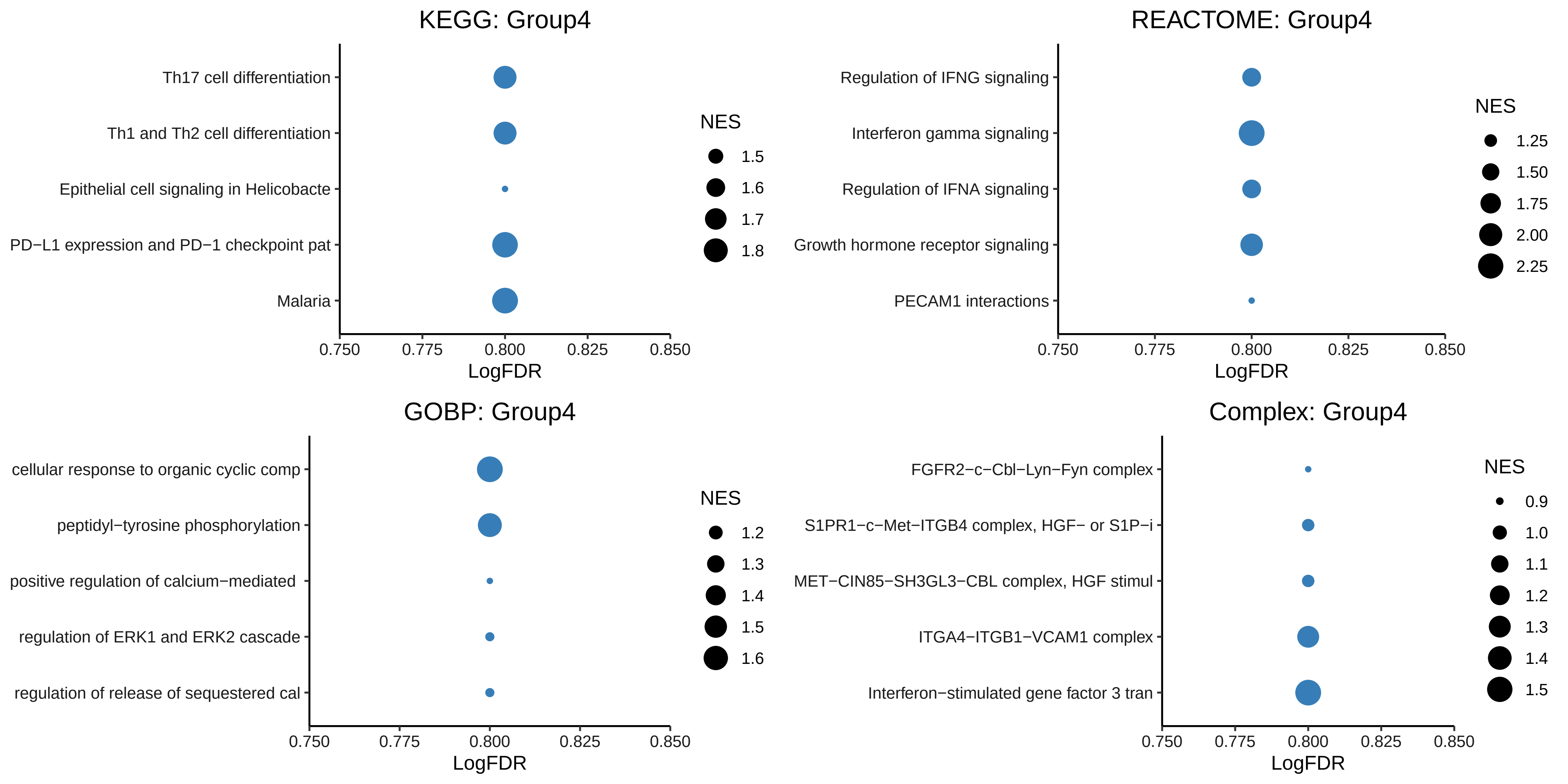

KEGG: Group4
Th17 cell differentiation
Th1 and Th2 cell differentiation
Epithelial cell signaling in Helicobacte
PD−L1 expression and PD−1 checkpoint pat
Malaria
0.750
0.775
0.800
0.825
0.850
LogFDR
NES
1.5
1.6
1.7
1.8
REACTOME: Group4
Regulation of IFNG signaling
Interferon gamma signaling
Regulation of IFNA signaling
Growth hormone receptor signaling
PECAM1 interactions
LogFDR
NES
1.25
1.50
1.75
2.00
2.25
GOBP: Group4
cellular response to organic cyclic comp
peptidyl−tyrosine phosphorylation
positive regulation of calcium−mediated
regulation of ERK1 and ERK2 cascade
regulation of release of sequestered cal
LogFDR
NES
1.2
1.3
1.4
1.5
1.6
Complex: Group4
FGFR2−c−Cbl−Lyn−Fyn complex
S1PR1−c−Met−ITGB4 complex, HGF− or S1P−i
MET−CIN85−SH3GL3−CBL complex, HGF stimul
ITGA4−ITGB1−VCAM1 complex
Interferon−stimulated gene factor 3 tran
LogFDR
NES
0.9
1.0
1.1
1.2
1.3
1.4
1.5

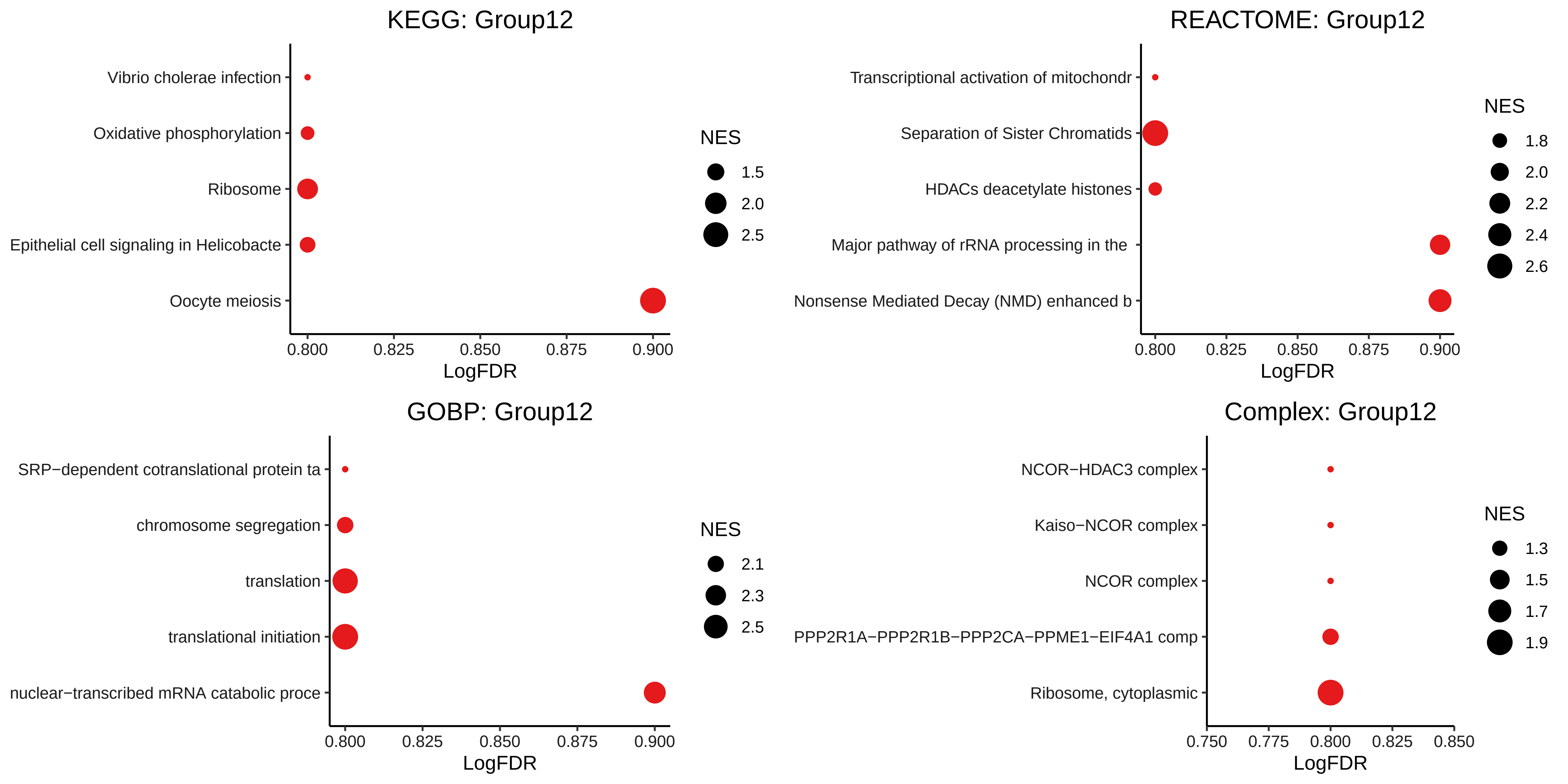
KEGG: Group12
Vibrio cholerae infection
Oxidative phosphorylation
Ribosome
Epithelial cell signaling in Helicobacte
Oocyte meiosis
0.800
0.825
0.850
0.875
0.900
LogFDR
NES
1.5
2.0
2.5
REACTOME: Group12
Transcriptional activation of mitochondr
Separation of Sister Chromatids
HDACs deacetylate histones
Major pathway of rRNA processing in the
Nonsense Mediated Decay (NMD) enhanced b
0.800
0.825
0.850
0.875
0.900
LogFDR
NES
1.8
2.0
2.2
2.4
2.6
GOBP: Group12
SRP−dependent cotranslational protein ta
chromosome segregation
translation
translational initiation
nuclear−transcribed mRNA catabolic proce
0.800
0.825
0.850
0.875
0.900
LogFDR
NES
2.1
2.3
2.5
Complex: Group12
NCOR−HDAC3 complex
Kaiso−NCOR complex
NCOR complex
PPP2R1A−PPP2R1B−PPP2CA−PPME1−EIF4A1 comp
Ribosome, cytoplasmic
0.750
0.775
0.800
0.825
0.850
LogFDR
NES
1.3
1.5
1.7
1.9

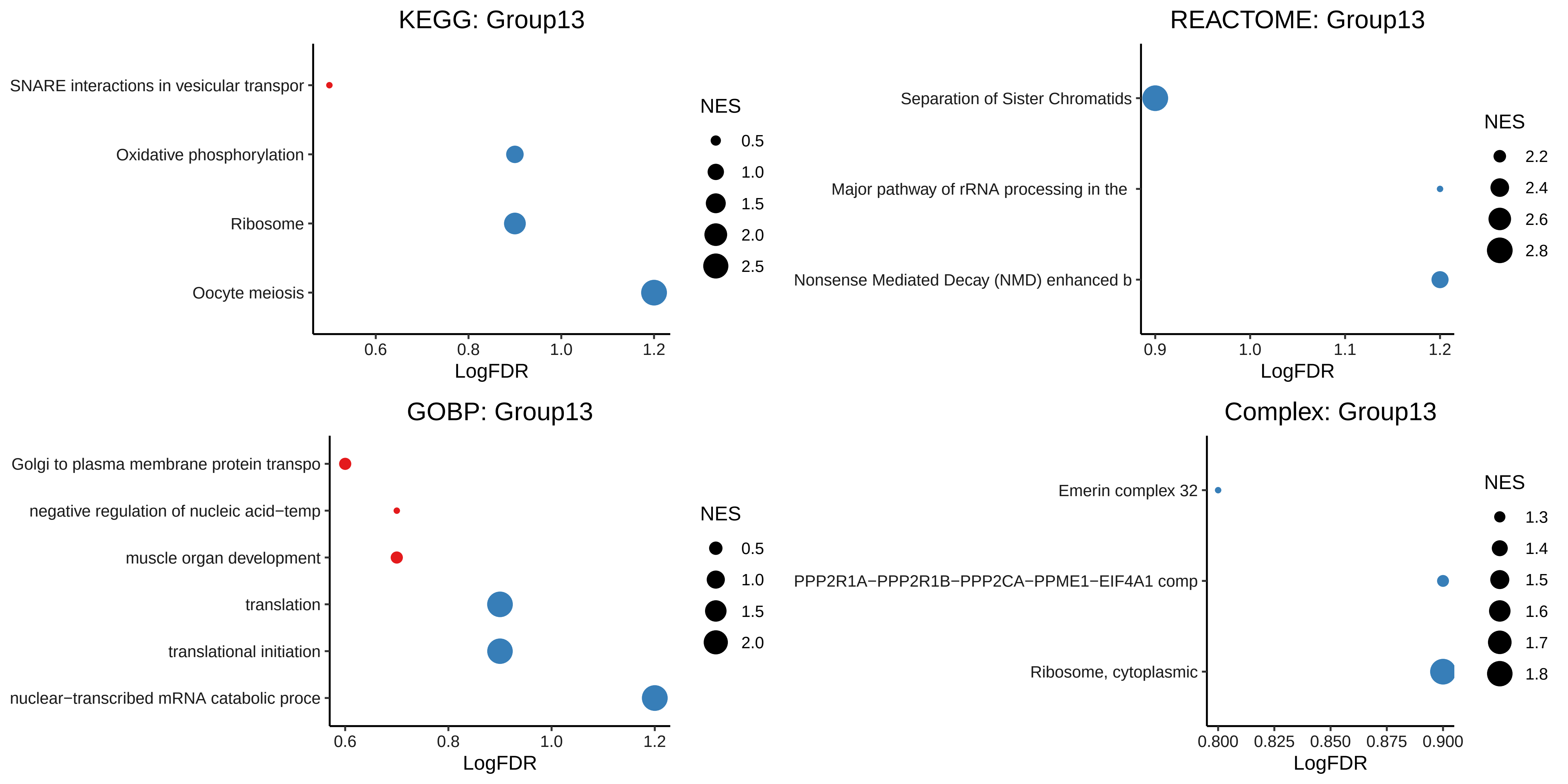
KEGG: Group13
SNARE interactions in vesicular transpor
Oxidative phosphorylation
Ribosome
Oocyte meiosis
0.6
0.8
1.0
1.2
LogFDR
NES
0.5
1.0
1.5
2.0
2.5
REACTOME: Group13
Separation of Sister Chromatids
Major pathway of rRNA processing in the
Nonsense Mediated Decay (NMD) enhanced b
0.9
1.0
1.1
1.2
LogFDR
NES
2.2
2.4
2.6
2.8
GOBP: Group13
Golgi to plasma membrane protein transpo
negative regulation of nucleic acid−temp
muscle organ development
translation
translational initiation
nuclear−transcribed mRNA catabolic proce
0.6
0.8
1.0
1.2
LogFDR
NES
0.5
1.0
1.5
2.0
Complex: Group13
Emerin complex 32
PPP2R1A−PPP2R1B−PPP2CA−PPME1−EIF4A1 comp
Ribosome, cytoplasmic
0.800
0.825
0.850
0.875
0.900
LogFDR
NES
1.3
1.4
1.5
1.6
1.7
1.8

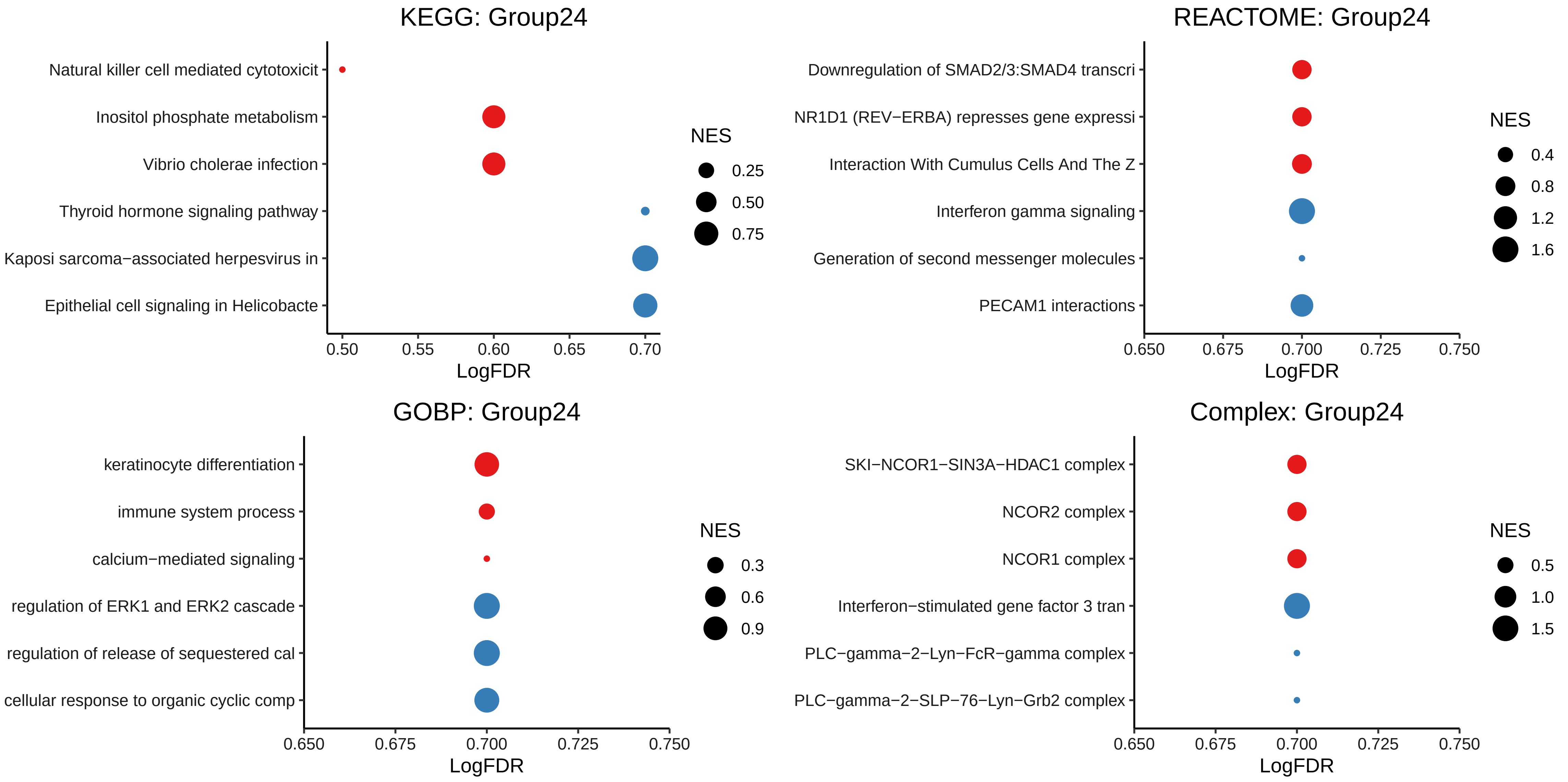

KEGG: Group24
Natural killer cell mediated cytotoxicit
Inositol phosphate metabolism
Vibrio cholerae infection
Thyroid hormone signaling pathway
Kaposi sarcoma−associated herpesvirus in
Epithelial cell signaling in Helicobacte
0.50
0.55
0.60
0.65
0.70
LogFDR
NES
0.25
0.50
0.75
REACTOME: Group24
Downregulation of SMAD2/3:SMAD4 transcri
NR1D1 (REV−ERBA) represses gene expressi
Interaction With Cumulus Cells And The Z
Interferon gamma signaling
Generation of second messenger molecules
PECAM1 interactions
0.650
0.675
0.700
0.725
0.750
LogFDR
NES
0.4
0.8
1.2
1.6
GOBP: Group24
keratinocyte differentiation
immune system process
calcium−mediated signaling
regulation of ERK1 and ERK2 cascade
regulation of release of sequestered cal
cellular response to organic cyclic comp
0.650
0.675
0.700
0.725
0.750
LogFDR
NES
0.3
0.6
0.9
Complex: Group24
SKI−NCOR1−SIN3A−HDAC1 complex
NCOR2 complex
NCOR1 complex
Interferon−stimulated gene factor 3 tran
PLC−gamma−2−Lyn−FcR−gamma complex
PLC−gamma−2−SLP−76−Lyn−Grb2 complex
0.650
0.675
0.700
0.725
0.750
LogFDR
NES
0.5
1.0
1.5

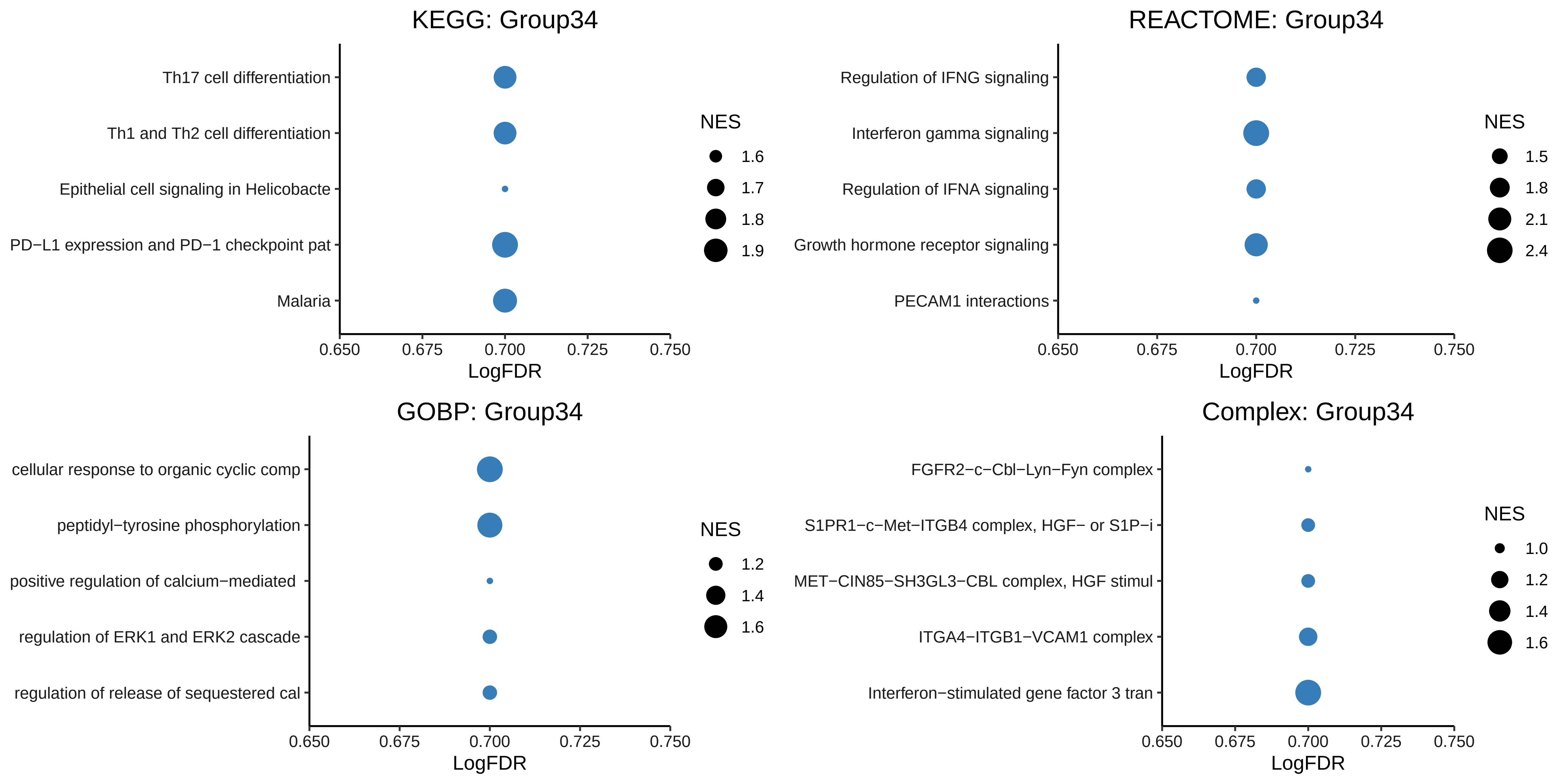

KEGG: Group34
Th17 cell differentiation
Th1 and Th2 cell differentiation
Epithelial cell signaling in Helicobacte
PD−L1 expression and PD−1 checkpoint pat
Malaria
0.650
0.675
0.700
0.725
0.750
LogFDR
NES
1.6
1.7
1.8
1.9
REACTOME: Group34
Regulation of IFNG signaling
Interferon gamma signaling
Regulation of IFNA signaling
Growth hormone receptor signaling
PECAM1 interactions
0.650
0.675
0.700
0.725
0.750
LogFDR
NES
1.5
1.8
2.1
2.4
GOBP: Group34
cellular response to organic cyclic comp
peptidyl−tyrosine phosphorylation
positive regulation of calcium−mediated
regulation of ERK1 and ERK2 cascade
regulation of release of sequestered cal
0.650
0.675
0.700
0.725
0.750
LogFDR
NES
1.2
1.4
1.6
Complex: Group34
FGFR2−c−Cbl−Lyn−Fyn complex
S1PR1−c−Met−ITGB4 complex, HGF− or S1P−i
MET−CIN85−SH3GL3−CBL complex, HGF stimul
ITGA4−ITGB1−VCAM1 complex
Interferon−stimulated gene factor 3 tran
0.650
0.675
0.700
0.725
0.750
LogFDR
NES
1.0
1.2
1.4
1.6
