# Supplementary material for: In vivo CRISPR screens reveal Serpinb9 and Adam2 as regulators of immune therapy response in lung cancer
Source: Nat Commun. 2023 May 31;14:3150. doi: 10.1038/s41467-023-38841-7 (PMC10232477; doi:10.1038/s41467-023-38841-7)
Supplement: Supplementary file 13 — Source Data [file 41467_2023_38841_MOESM13_ESM.zip › Figure 3A,B.zip!/Lungs_sgRNA analysis_KRAS /Lung-up.pdf]

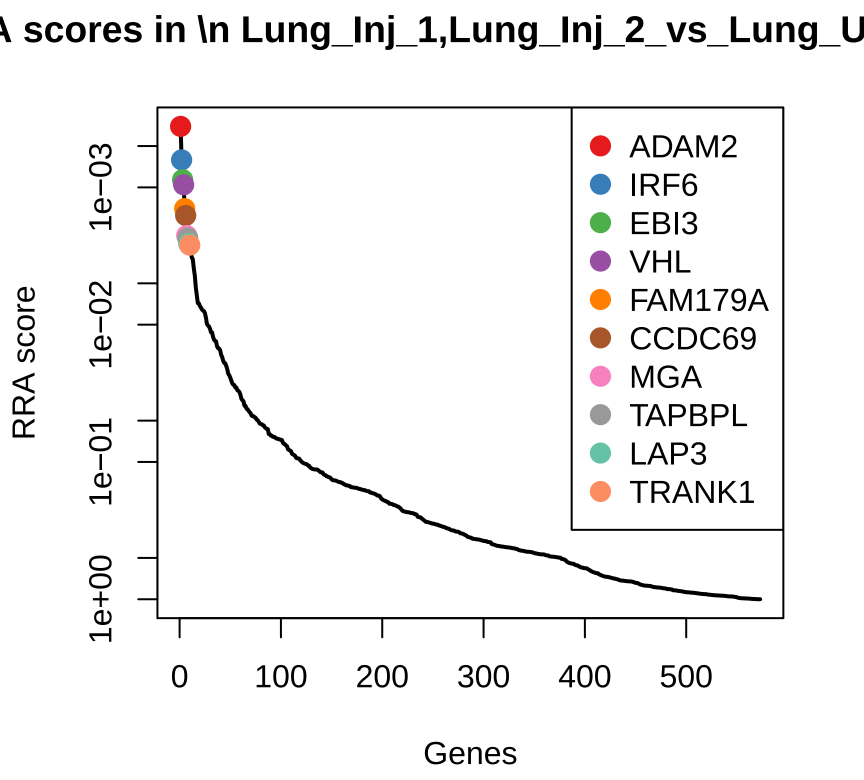

A scores in \n Lung_Inj_1,Lung_Inj_2_vs_Lung_U
RRA score
1e+00
1e−01
1e−02
1e−03
Genes
0
100
200
300
400
500
ADAM2
IRF6
EBI3
VHL
FAM179A
CCDC69
MGA
TAPBPL
LAP3
TRANK1

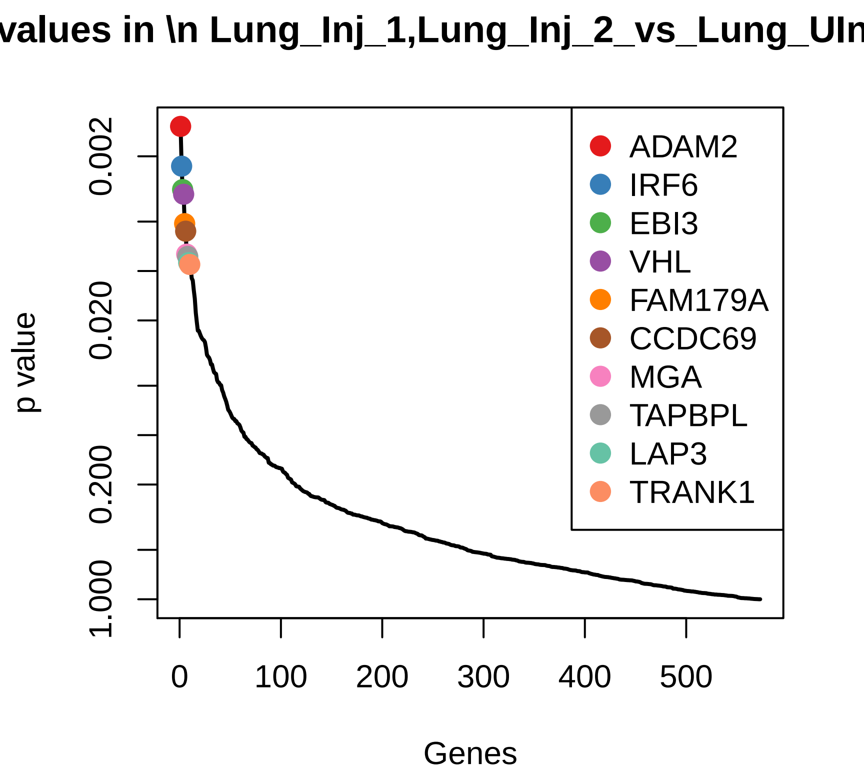

values in \n Lung_Inj_1,Lung_Inj_2_vs_Lung_UIn
p value
0.002
0.020
0.200
1.000
0
100
200
300
400
500
Genes
ADAM2
IRF6
EBI3
VHL
FAM179A
CCDC69
MGA
TAPBPL
LAP3
TRANK1

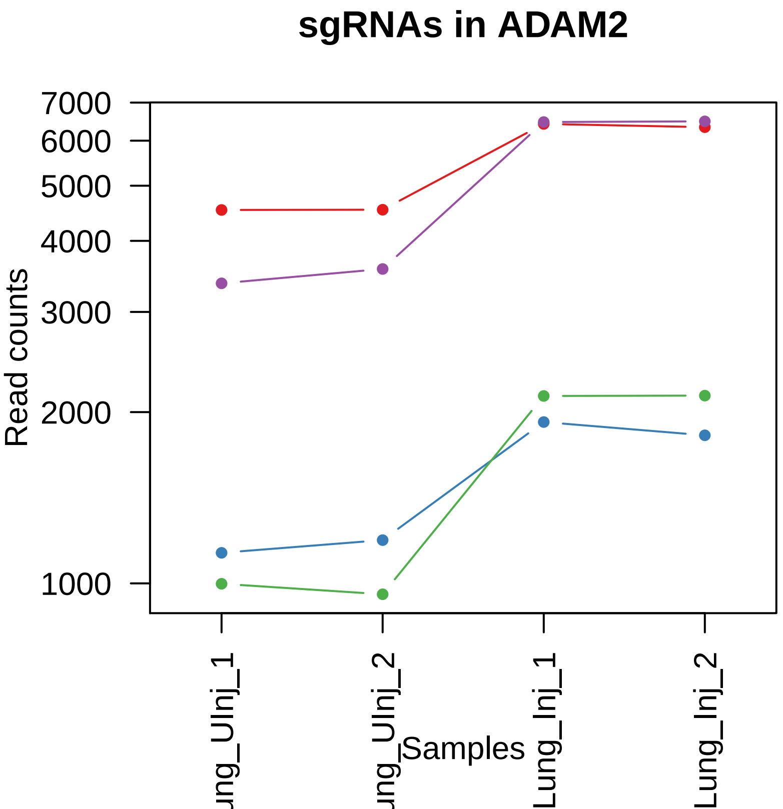

sgRNAs in ADAM2
Read counts
7000
6000
5000
4000
3000
2000
1000
ung_UInj_1
ung_UInj_2
Lung_Inj_1
Lung_Inj_2
Samples

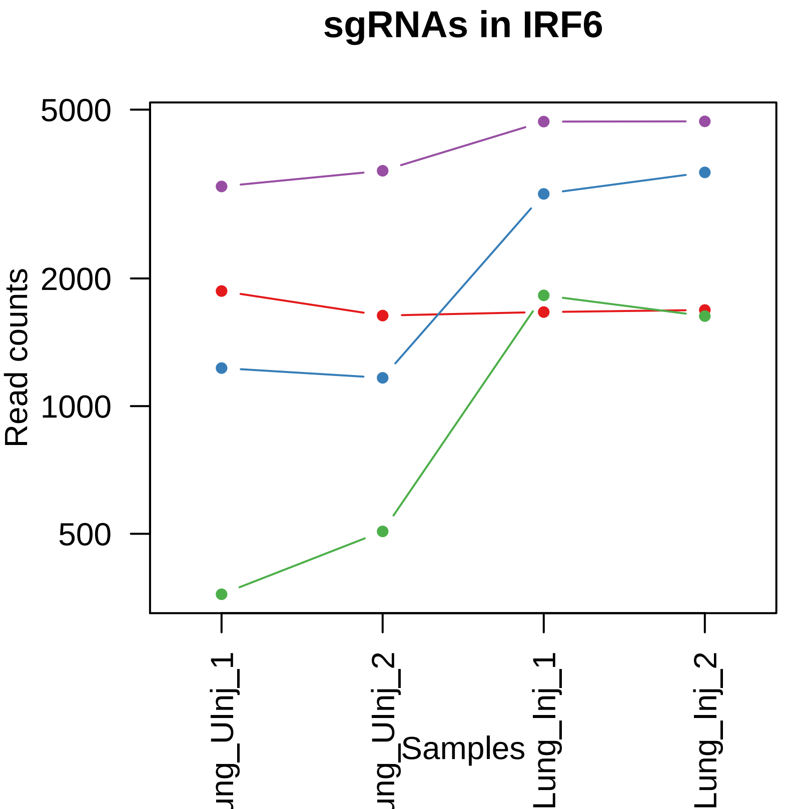
sgRNAs in IRF6
Read counts
5000
2000
1000
500
ung_UInj_1
ung_UInj_2
Lung_Inj_1
Lung_Inj_2
Samples

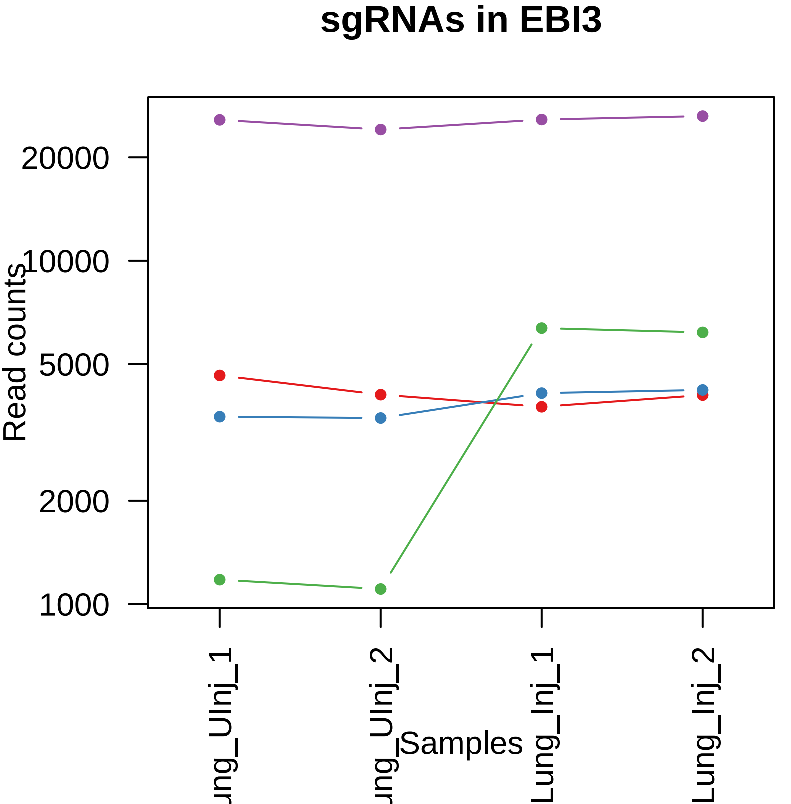
sgRNAs in EBI3
Read counts
20000
10000
5000
2000
1000
Samples

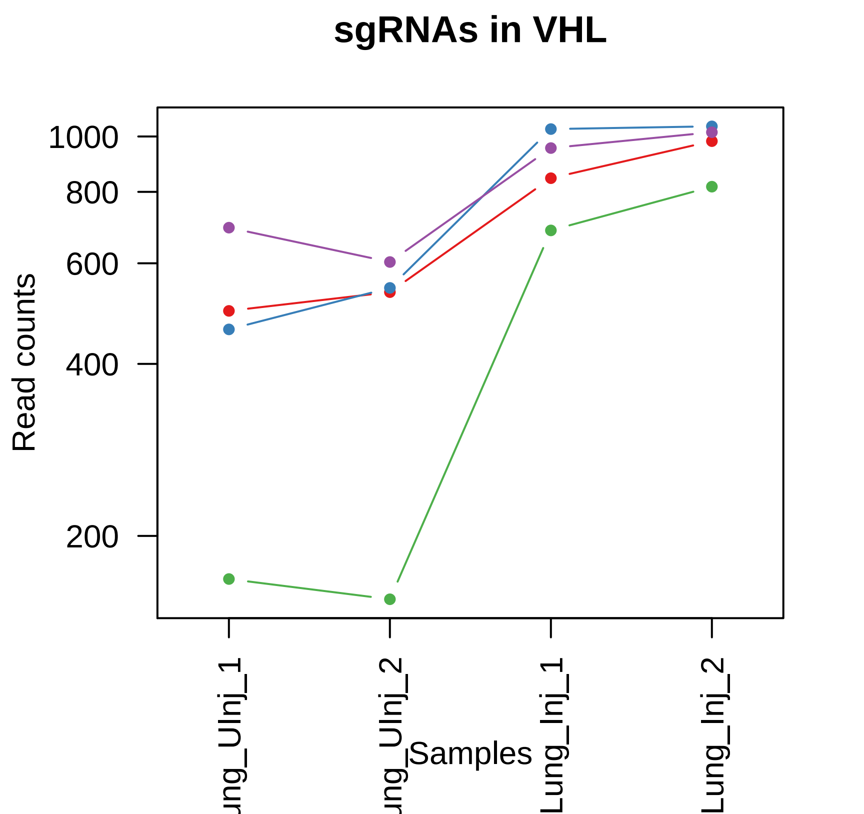

sgRNAs in VHL
Read counts
1000
800
600
400
200
Samples
Lung_Inj_1
Lung_Inj_2

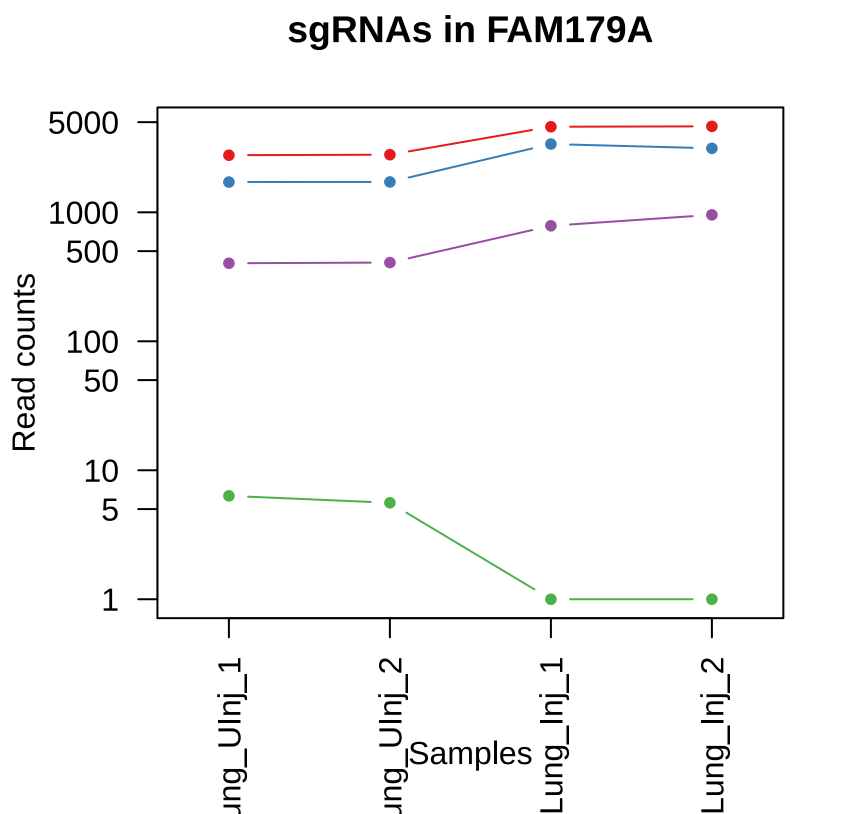

sgRNAs in FAM179A
Read counts
5000
1000
500
100
50
10
5
1
Samples
Lung_Inj_1
Lung_Inj_2

sgRNAs in CCDC69

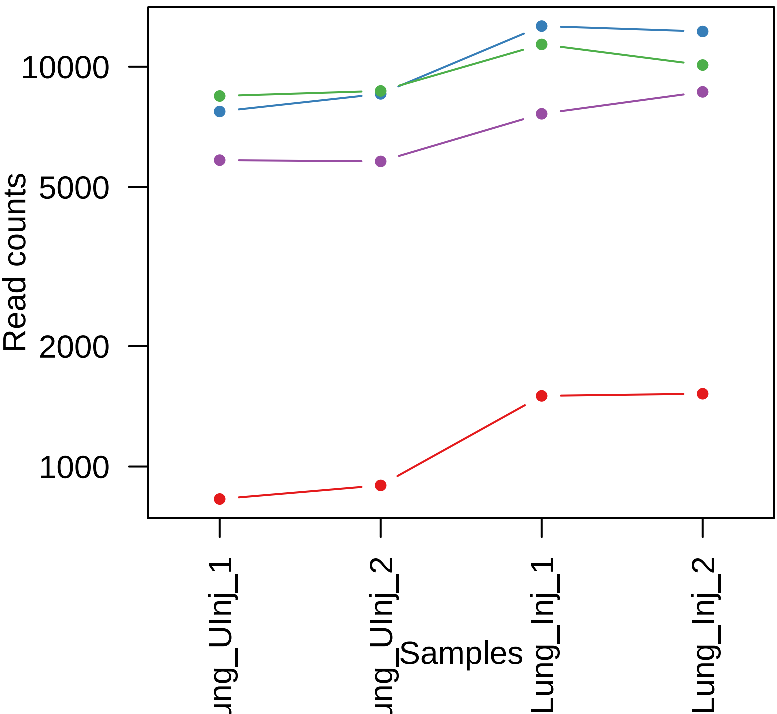

sgRNAs in MGA

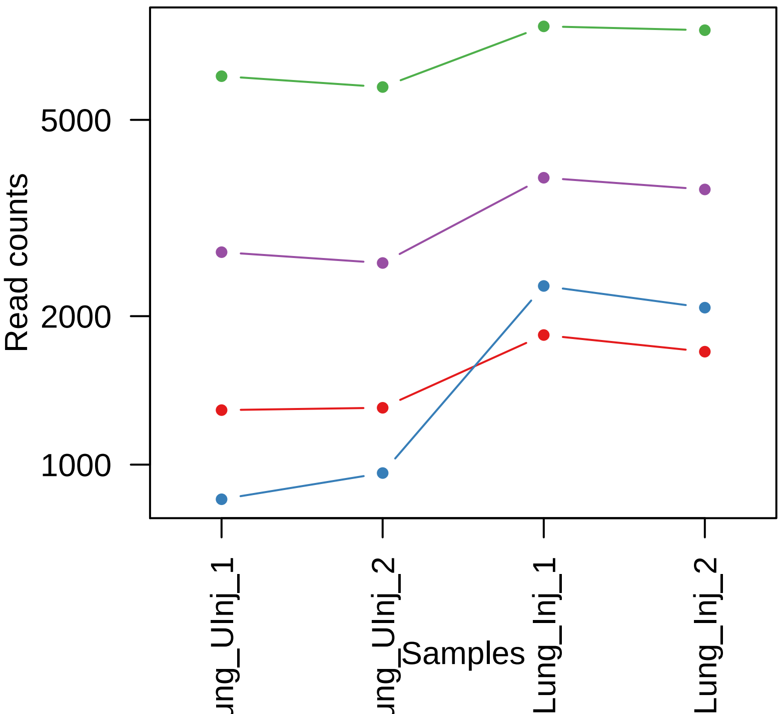

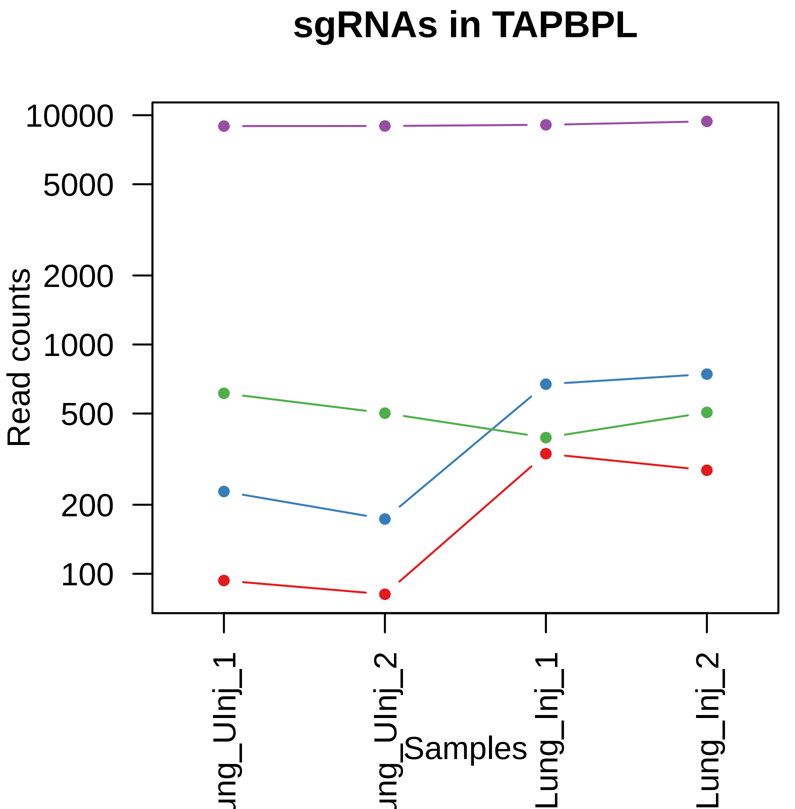
sgRNAs in TAPBPL
Read counts
10000
5000
2000
1000
500
200
100
Samples
ung_UInj_1
ung_UInj_2
Lung_Inj_1
Lung_Inj_2

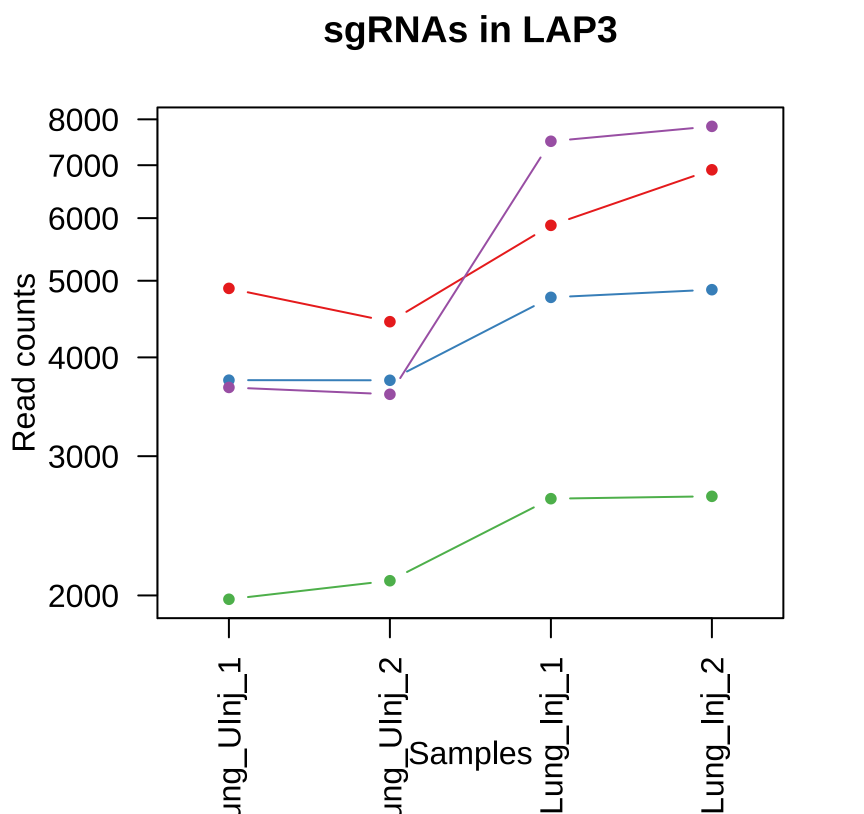

sgRNAs in LAP3
Read counts
8000
7000
6000
5000
4000
3000
2000
Samples
ung_UInj_1
ung_UInj_2
Lung_Inj_1
Lung_Inj_2

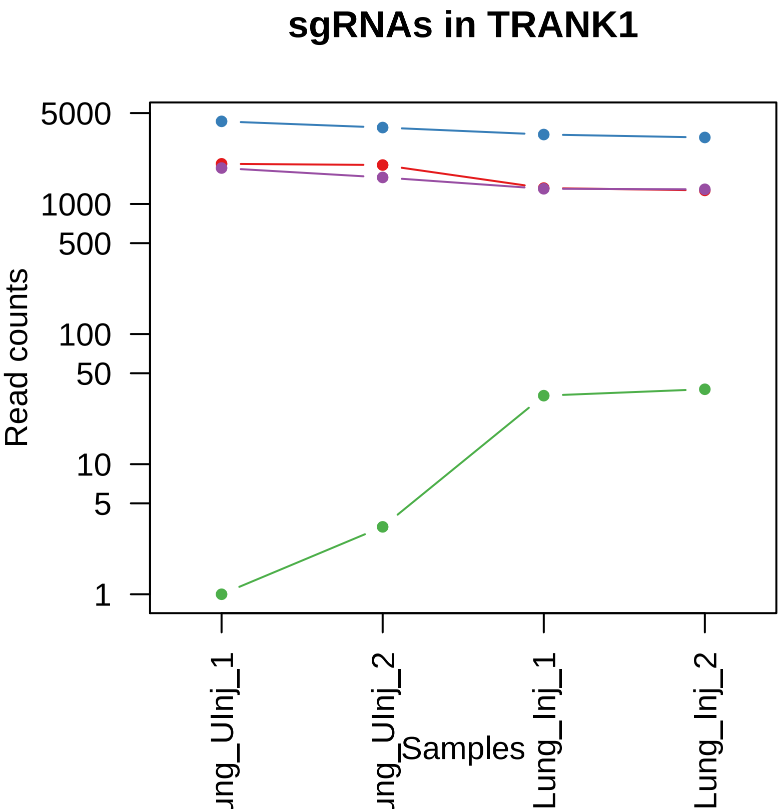
sgRNAs in TRANK1
Read counts
5000
1000
500
100
50
10
5
1
Samples
Lung_Inj_1
Lung_Inj_2
