# Supplementary material for: In vivo CRISPR screens reveal Serpinb9 and Adam2 as regulators of immune therapy response in lung cancer
Source: Nat Commun. 2023 May 31;14:3150. doi: 10.1038/s41467-023-38841-7 (PMC10232477; doi:10.1038/s41467-023-38841-7)
Supplement: Supplementary file 13 — Source Data [file 41467_2023_38841_MOESM13_ESM.zip › Figure 1G.zip!/Braf_Lung screen/sample1.pdf]

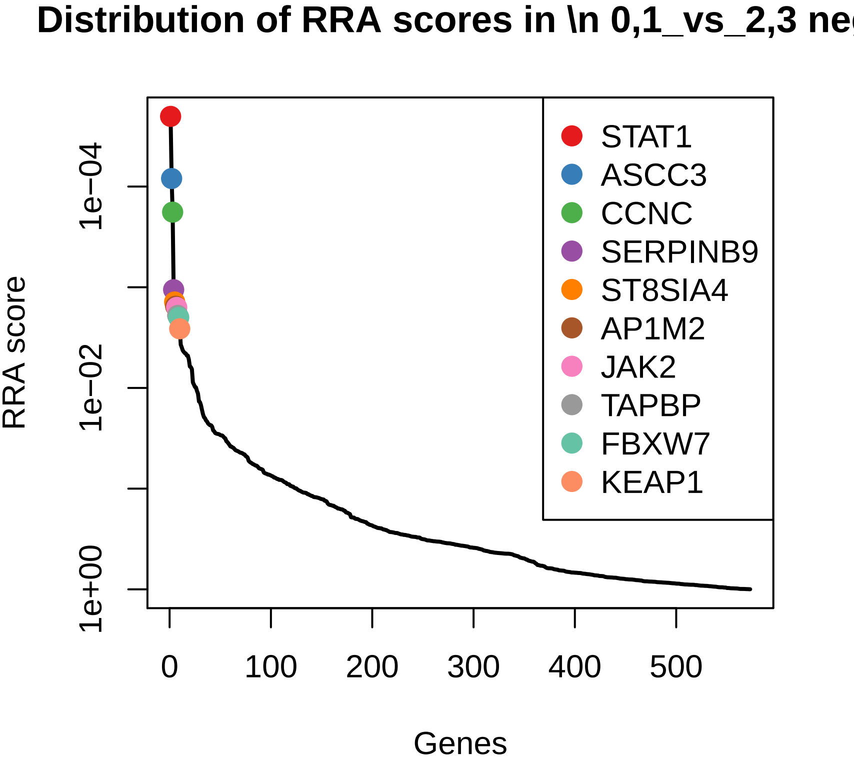

Distribution of RRA scores in \n 0,1_vs_2,3 neg
RRA score
1e−04
1e−02
1e+00
Genes
0
100
200
300
400
500
STAT1
ASCC3
CCNC
SERPINB9
ST8SIA4
AP1M2
JAK2
TAPBP
FBXW7
KEAP1

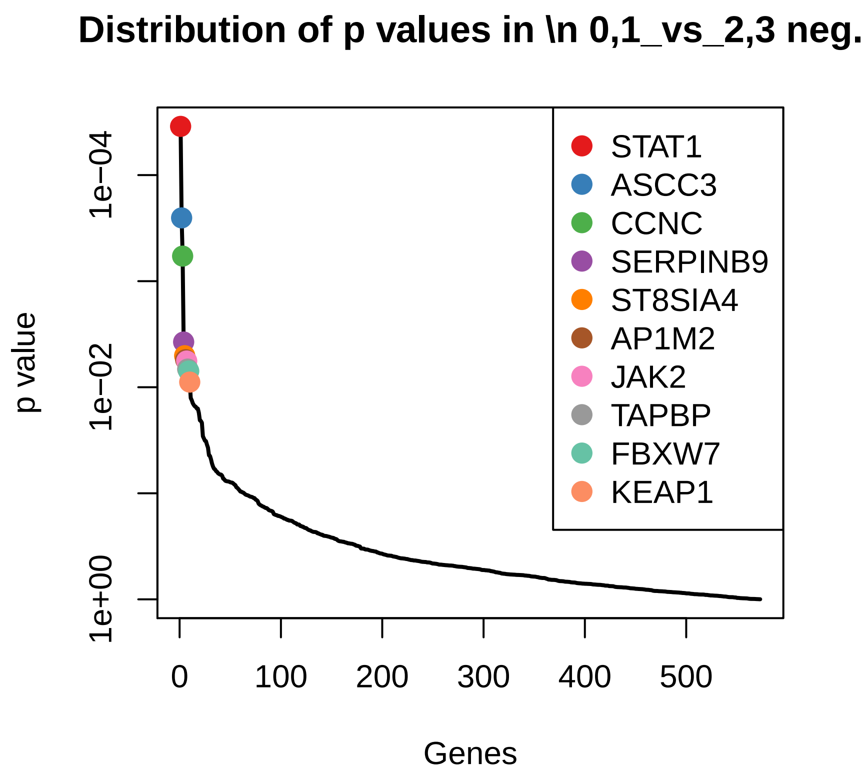

Distribution of p values in \n 0,1_vs_2,3 neg.
p value
1e−04
1e−02
1e+00
0
100
200
300
400
500
Genes
STAT1
ASCC3
CCNC
SERPINB9
ST8SIA4
AP1M2
JAK2
TAPBP
FBXW7
KEAP1

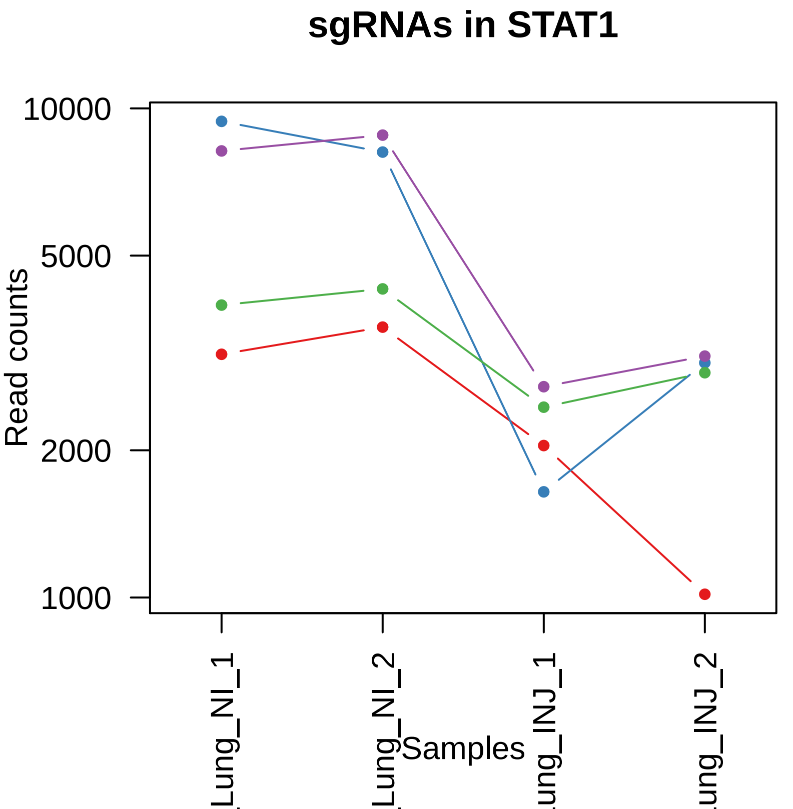
sgRNAs in STAT1
Read counts
10000
5000
2000
1000
Lung_NI_1
Lung_NI_2
Lung_INJ_1
Lung_INJ_2
Samples

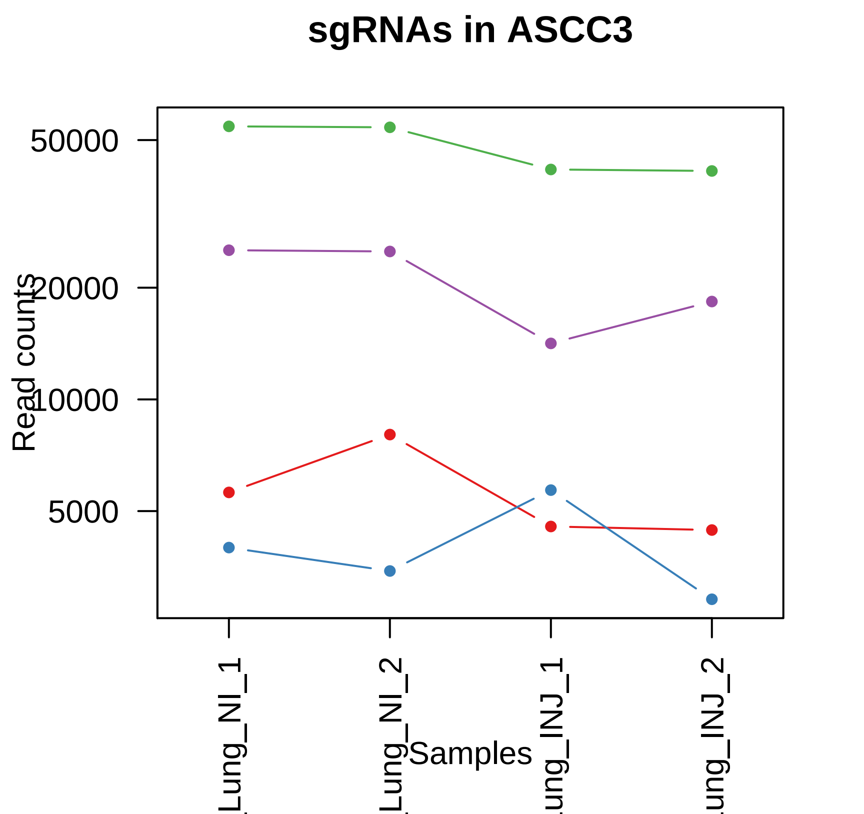
sgRNAs in ASCC3
Read counts
50000
20000
10000
5000
Samples
Lung_NI_1
Lung_NI_2

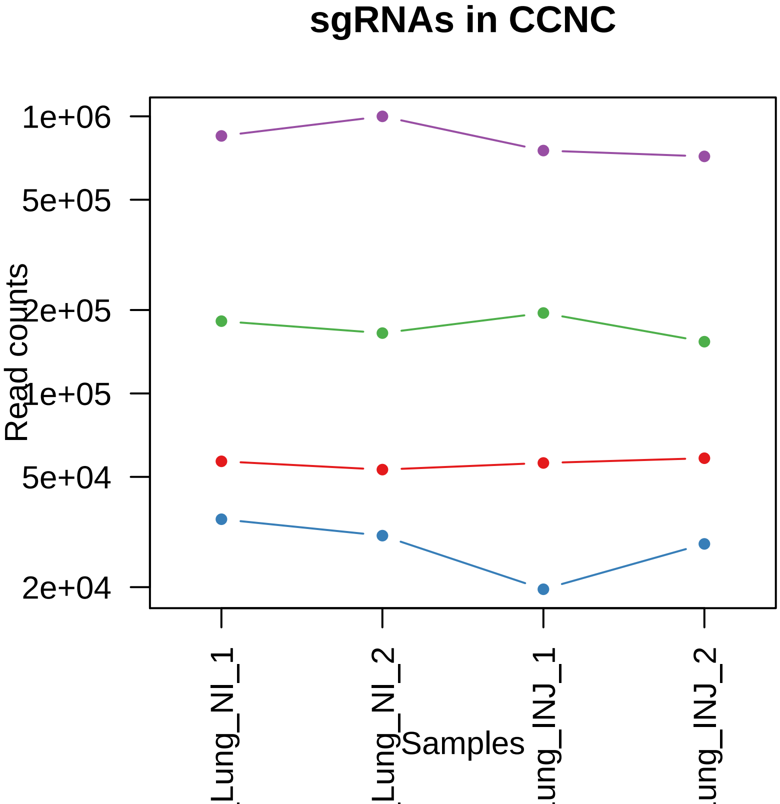

sgRNAs in CCNC
Read counts
1e+06
5e+05
2e+05
1e+05
5e+04
2e+04
Samples
Lung_NI_1
Lung_NI_2
Lung_INJ_1
Lung_INJ_2

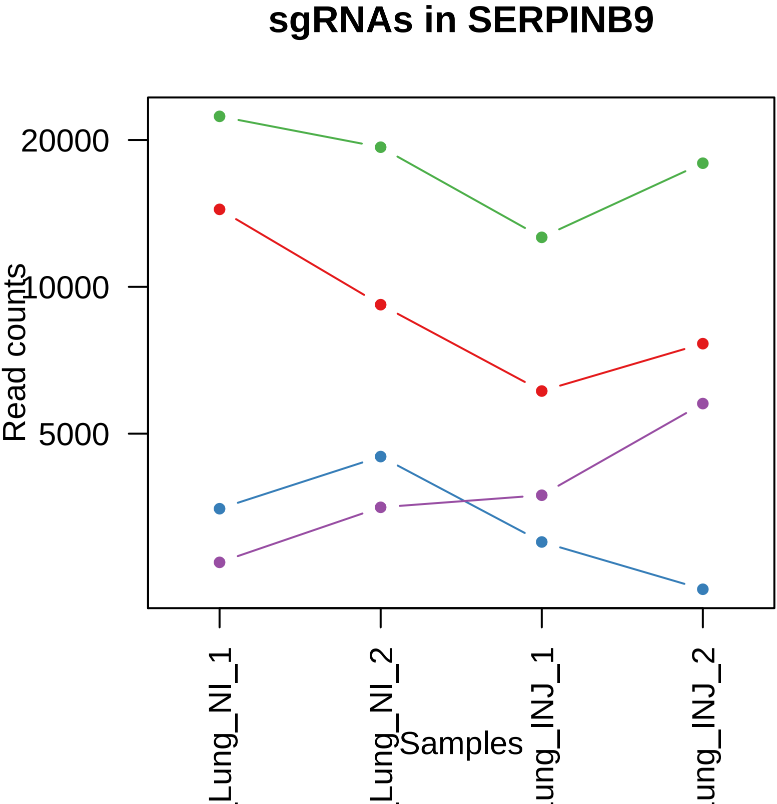

sgRNAs in SERPINB9
20000
10000
5000
Read counts
Lung_NI_1
Lung_NI_2
Samples

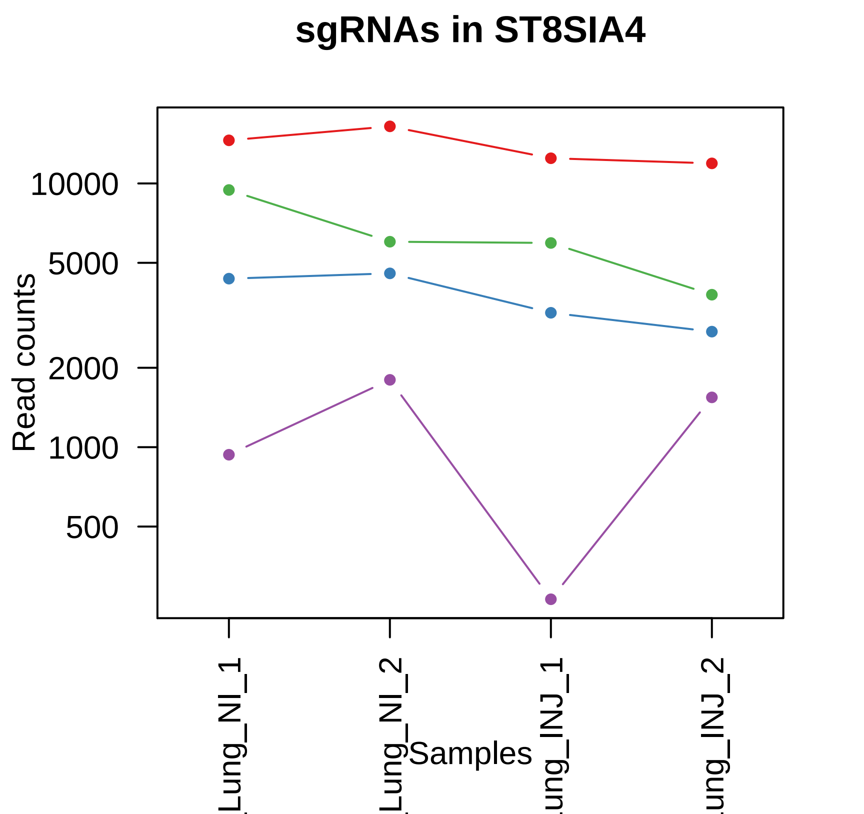

sgRNAs in ST8SIA4
Read counts
10000
5000
2000
1000
500
Samples
Lung_NI_1
Lung_NI_2
Lung_INJ_1
Lung_INJ_2

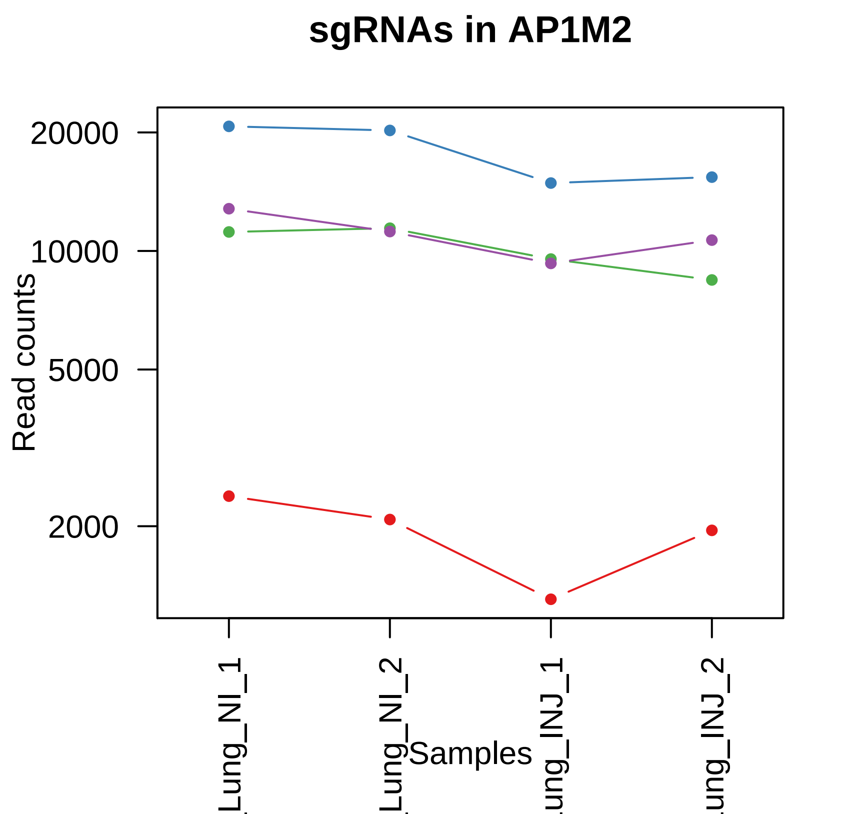

sgRNAs in AP1M2
Read counts
20000
10000
5000
2000
Lung_NI_1
Lung_NI_2
Lung_INJ_1
Lung_INJ_2
Samples

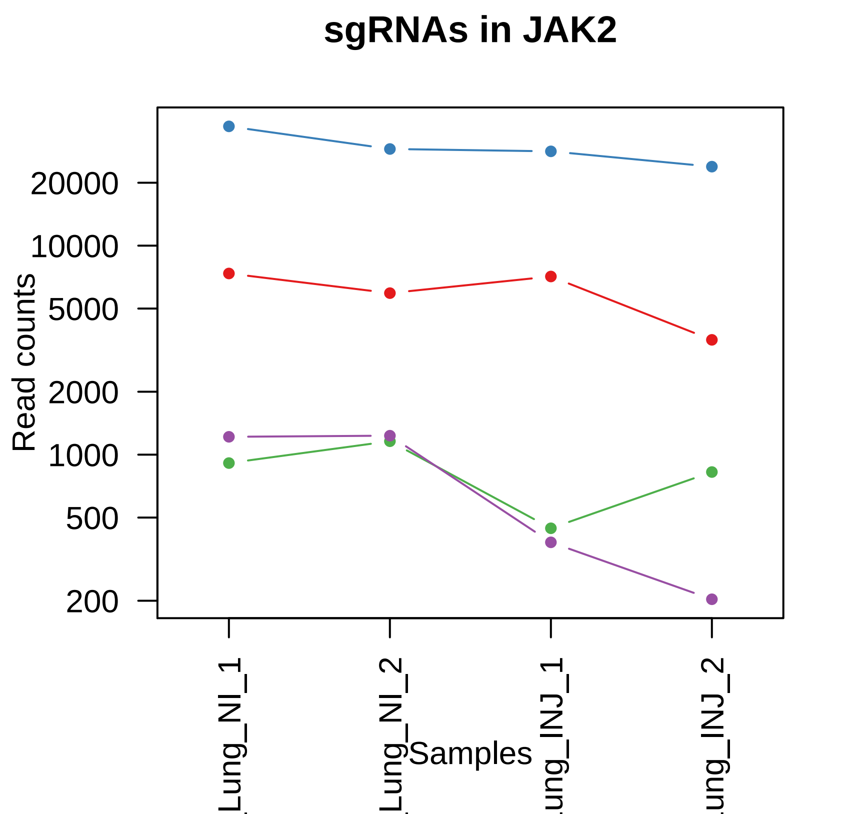

sgRNAs in JAK2
Read counts
20000
10000
5000
2000
1000
500
200
Lung_NI_1
Lung_NI_2
Lung_INJ_1
Lung_INJ_2
Samples

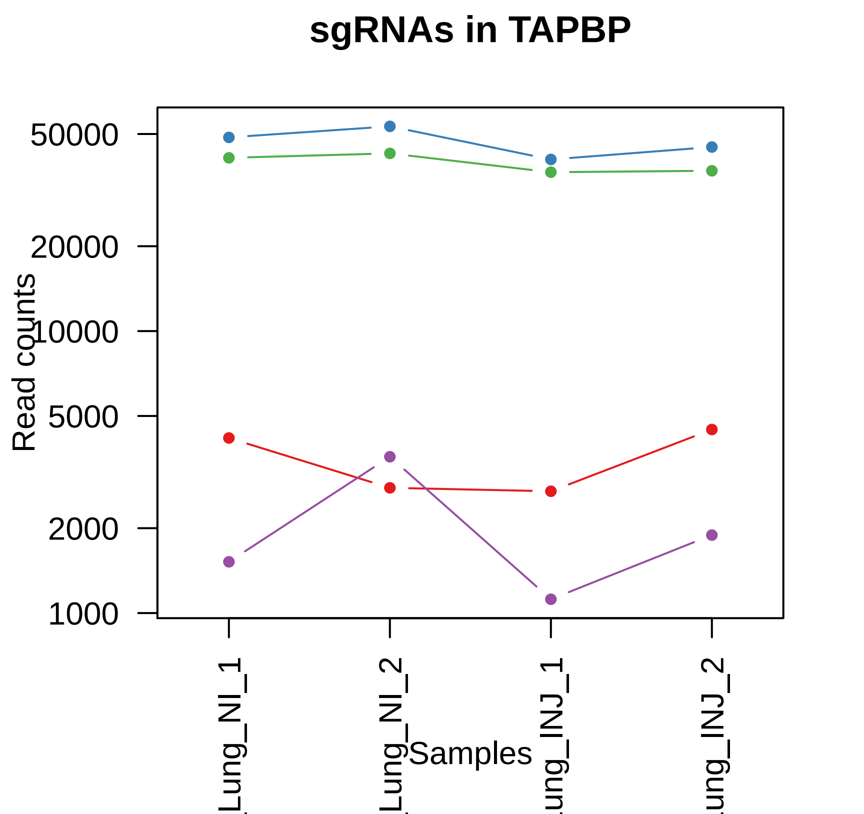

sgRNAs in TAPBP
Read counts
50000
20000
10000
5000
2000
1000
Samples
Lung_NI_1
Lung_NI_2
ung_INJ_1
ung_INJ_2

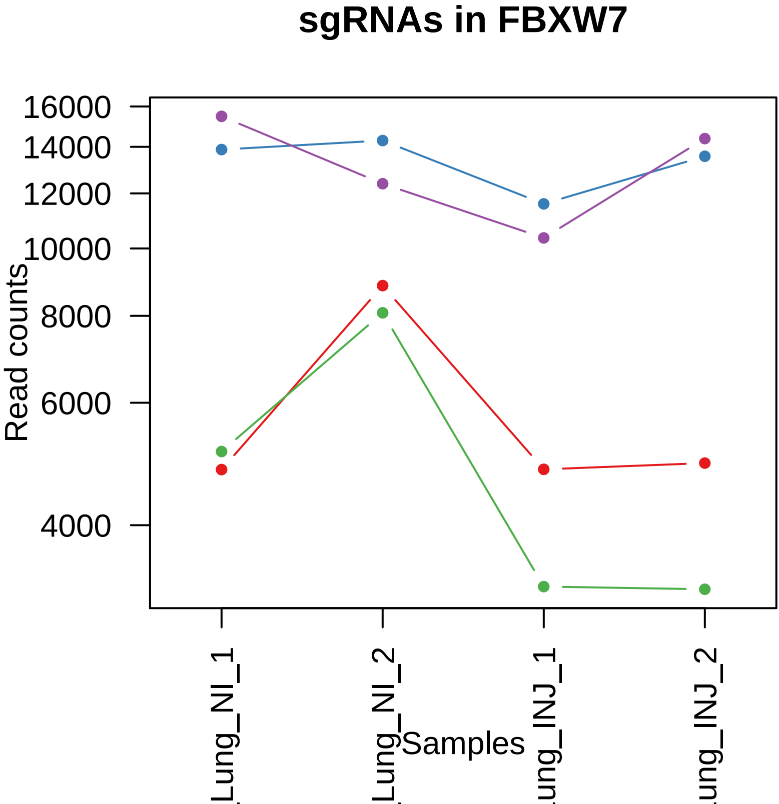

sgRNAs in FBXW7
16000
14000
12000
10000
8000
6000
4000
Read counts
Lung_NI_1
Lung_NI_2
Samples

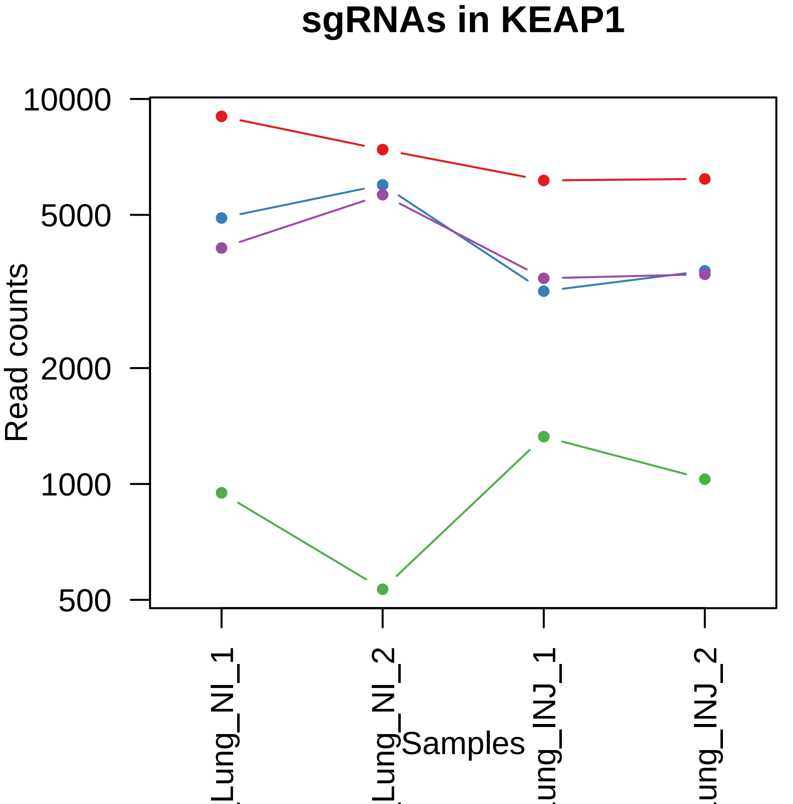

sgRNAs in KEAP1
10000
5000
2000
1000
500
Read counts
Lung_NI_1
Lung_NI_2
Samples

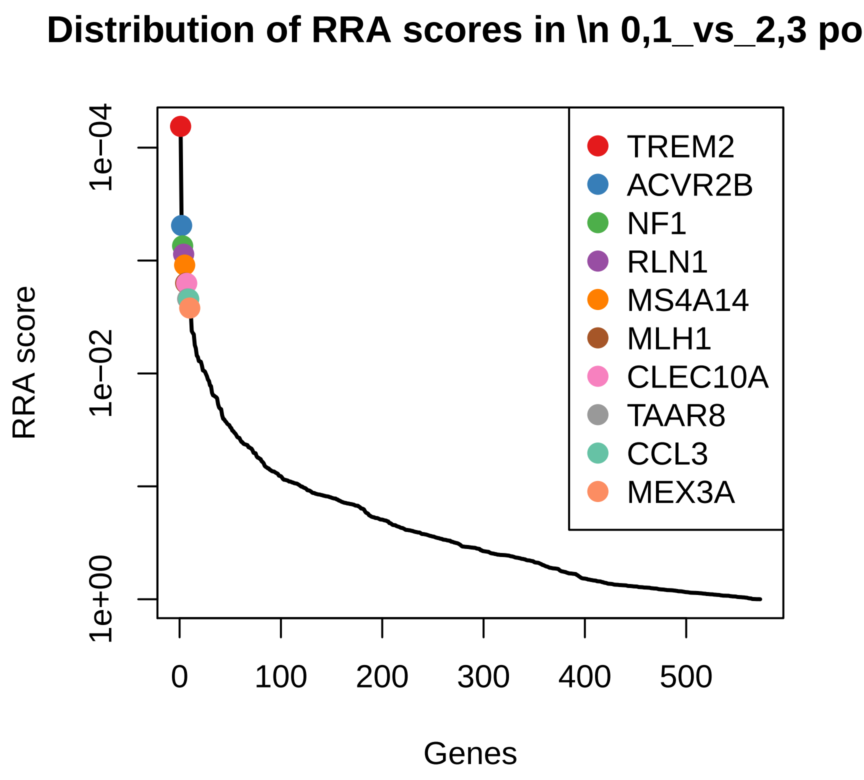

Distribution of RRA scores in \n 0,1_vs_2,3 po
RRA score
1e−04
1e−02
1e+00
Genes
0
100
200
300
400
500
TREM2
ACVR2B
NF1
RLN1
MS4A14
MLH1
CLEC10A
TAAR8
CCL3
MEX3A

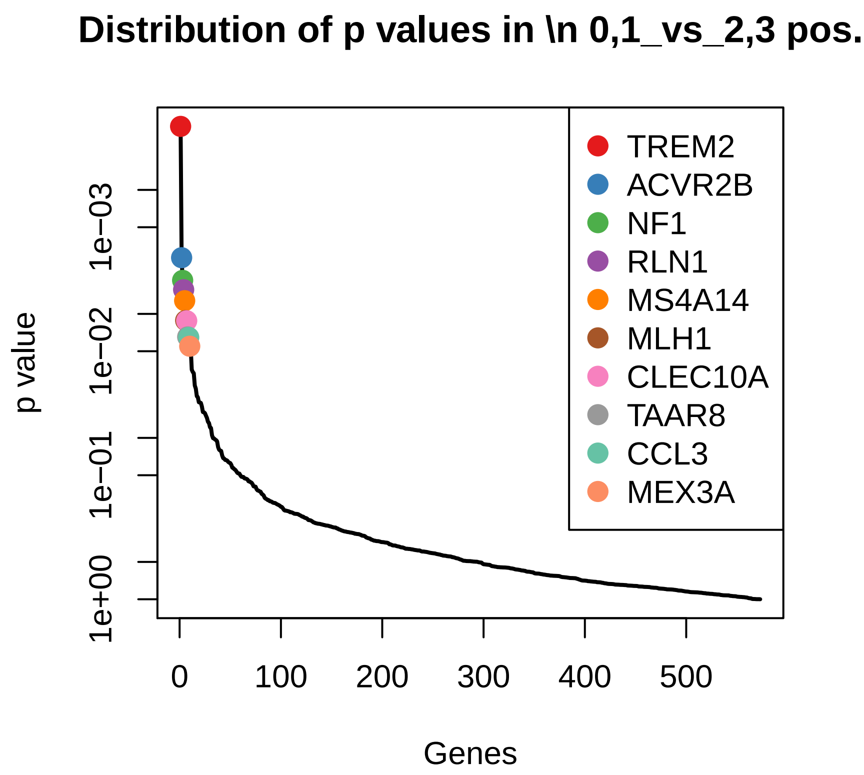

Distribution of p values in \n 0,1_vs_2,3 pos.
p value
1e+00
1e−01
1e−02
1e−03
Genes
0
100
200
300
400
500
TREM2
ACVR2B
NF1
RLN1
MS4A14
MLH1
CLEC10A
TAAR8
CCL3
MEX3A

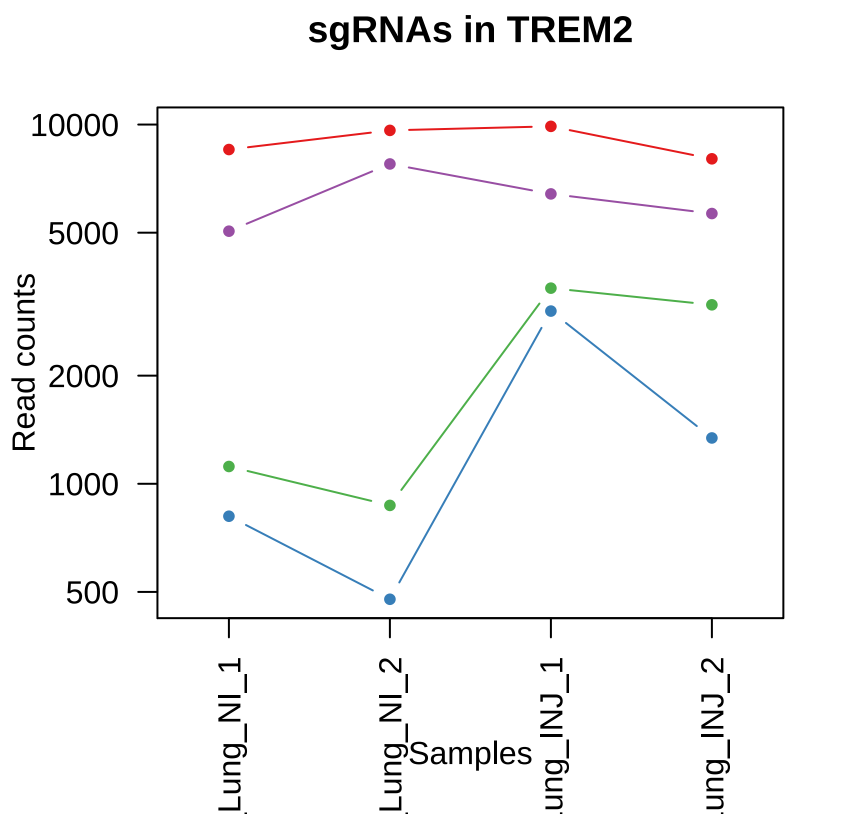

sgRNAs in TREM2
Read counts
10000
5000
2000
1000
500
Samples
Lung_NI_1
Lung_NI_2

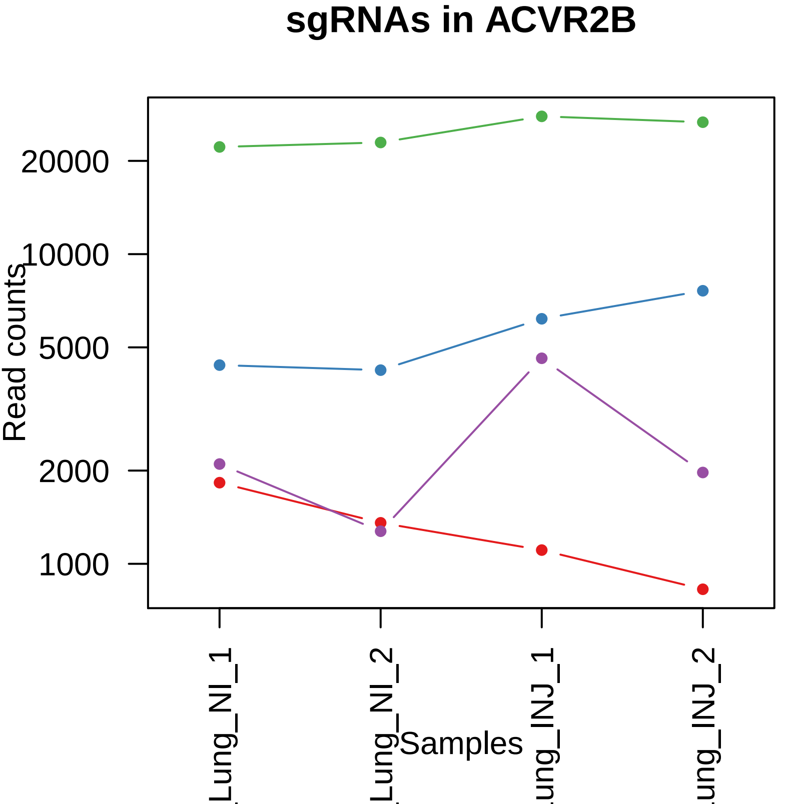

sgRNAs in ACVR2B
Read counts
20000
10000
5000
2000
1000
Samples
Lung_NI_1
Lung_NI_2

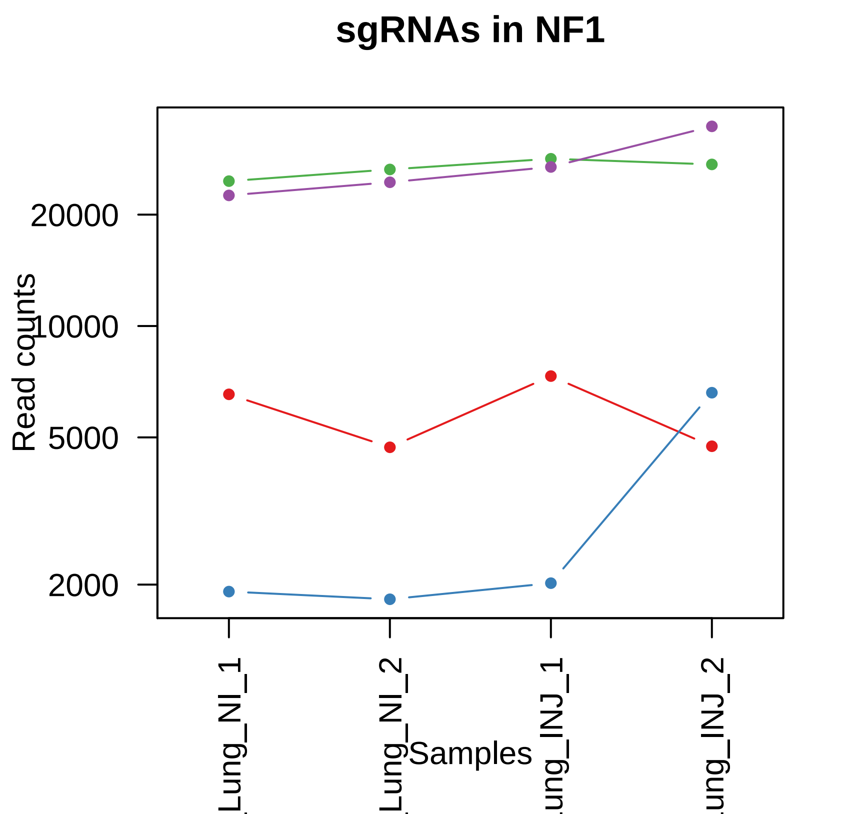
sgRNAs in NF1
Read counts
20000
10000
5000
2000
Lung_NI_1
Lung_NI_2
Lung_INJ_1
Lung_INJ_2
Samples

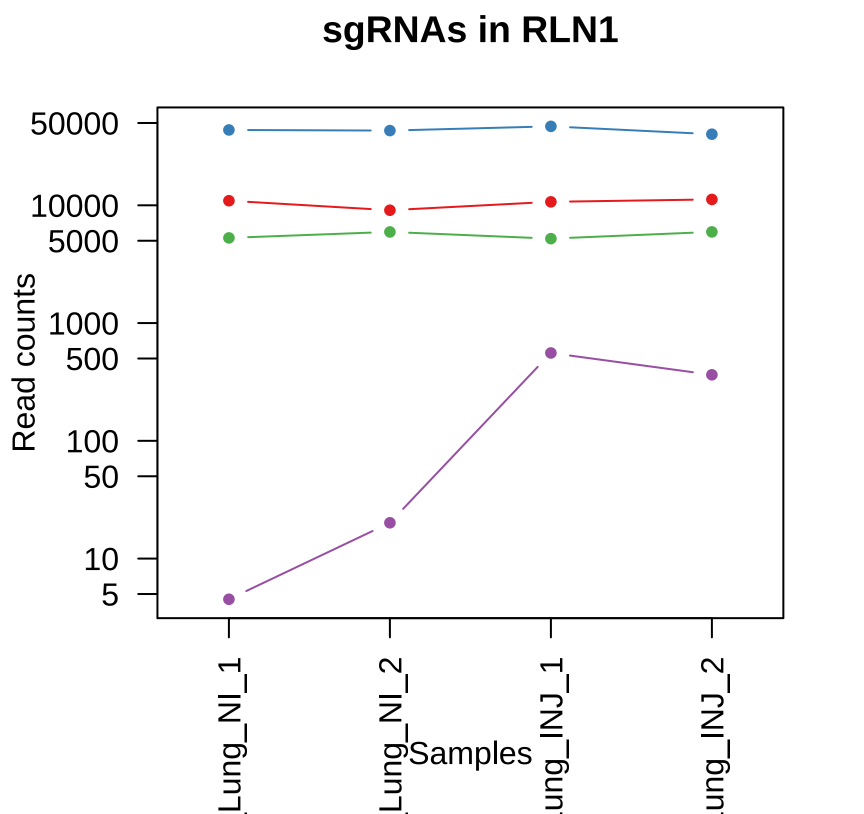

sgRNAs in RLN1
Read counts
50000
10000
5000
1000
500
100
50
10
5
Samples
Lung_NI_1
Lung_NI_2
ung_INJ_1
ung_INJ_2

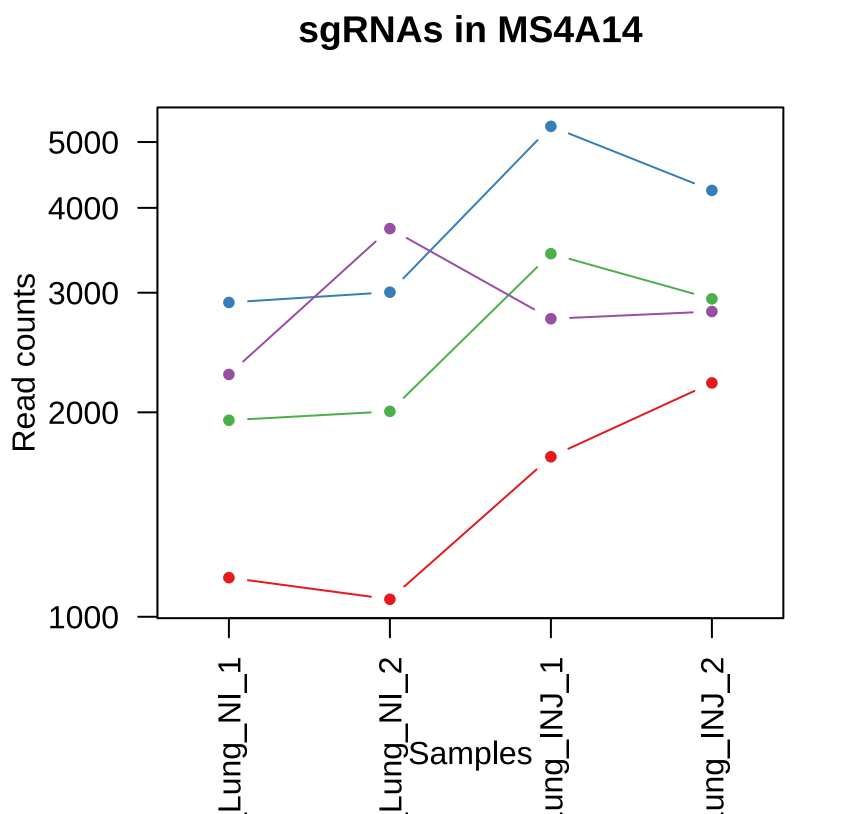

sgRNAs in MS4A14
Read counts
5000
4000
3000
2000
1000
Lung_NI_1
Lung_NI_2
Lung_INJ_1
Lung_INJ_2
Samples

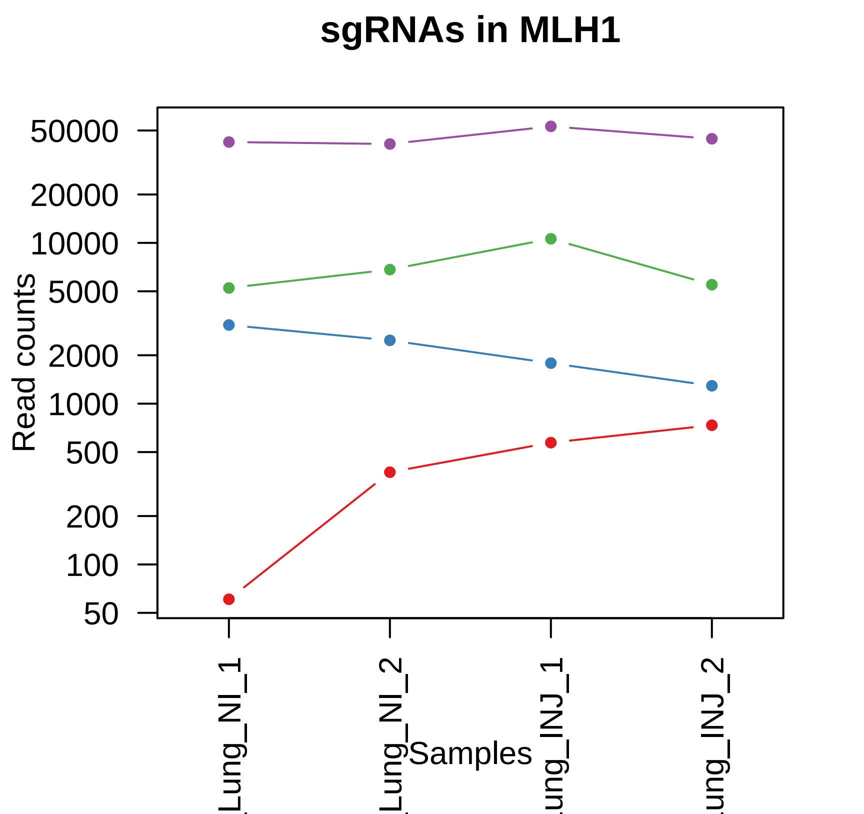

sgRNAs in MLH1
Read counts
50000
20000
10000
5000
2000
1000
500
200
100
50
Samples

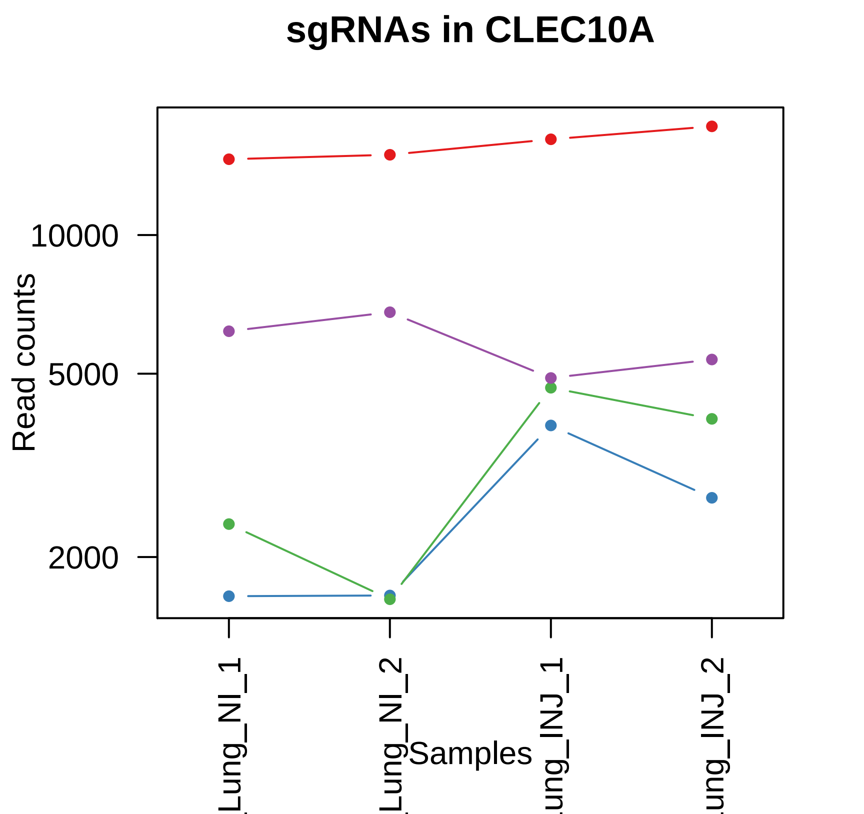

sgRNAs in CLEC10A
Read counts
10000
5000
2000
Samples
Lung_NI_1
Lung_NI_2
Lung_INJ_1
Lung_INJ_2

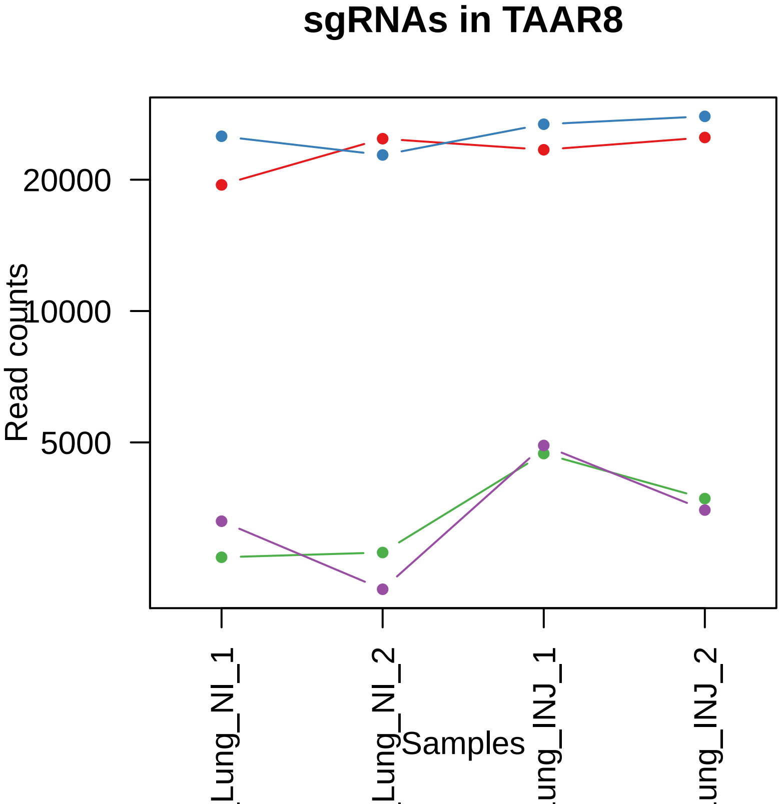
sgRNAs in TAAR8
Read counts
20000
10000
5000
Samples
Lung_NI_1
Lung_NI_2
Lung_INJ_1
Lung_INJ_2

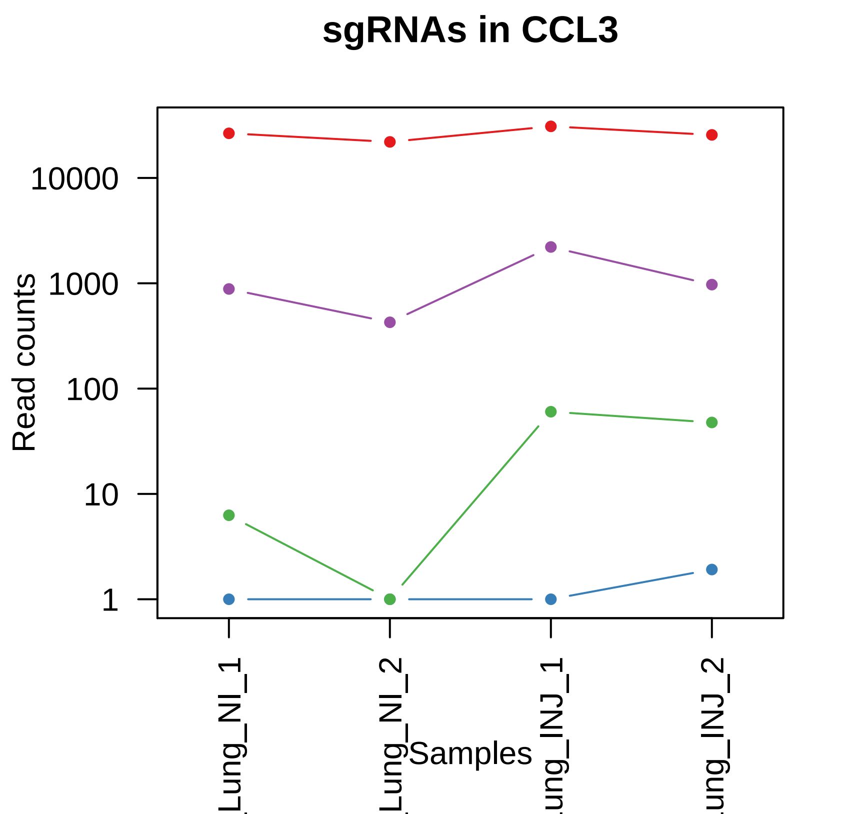

sgRNAs in CCL3
Read counts
10000
1000
100
10
1
Samples
Lung_NI_1
Lung_NI_2

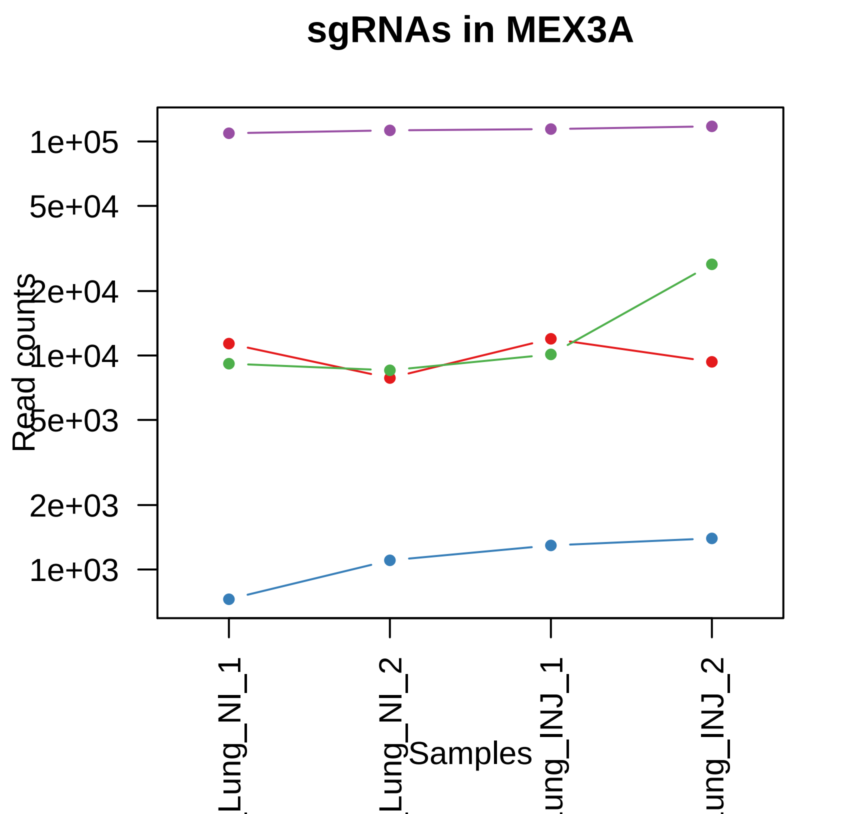
sgRNAs in MEX3A
Read counts
1e+05
5e+04
2e+04
1e+04
5e+03
2e+03
1e+03
Lung_NI_1
Lung_NI_2
Lung_INJ_1
Lung_INJ_2
Samples
