# Supplementary material for: In vivo CRISPR screens reveal Serpinb9 and Adam2 as regulators of immune therapy response in lung cancer
Source: Nat Commun. 2023 May 31;14:3150. doi: 10.1038/s41467-023-38841-7 (PMC10232477; doi:10.1038/s41467-023-38841-7)
Supplement: Supplementary file 13 — Source Data [file 41467_2023_38841_MOESM13_ESM.zip › Figure 1G.zip!/Braf_Lung screen/FluteRRA_BRAF.pdf]

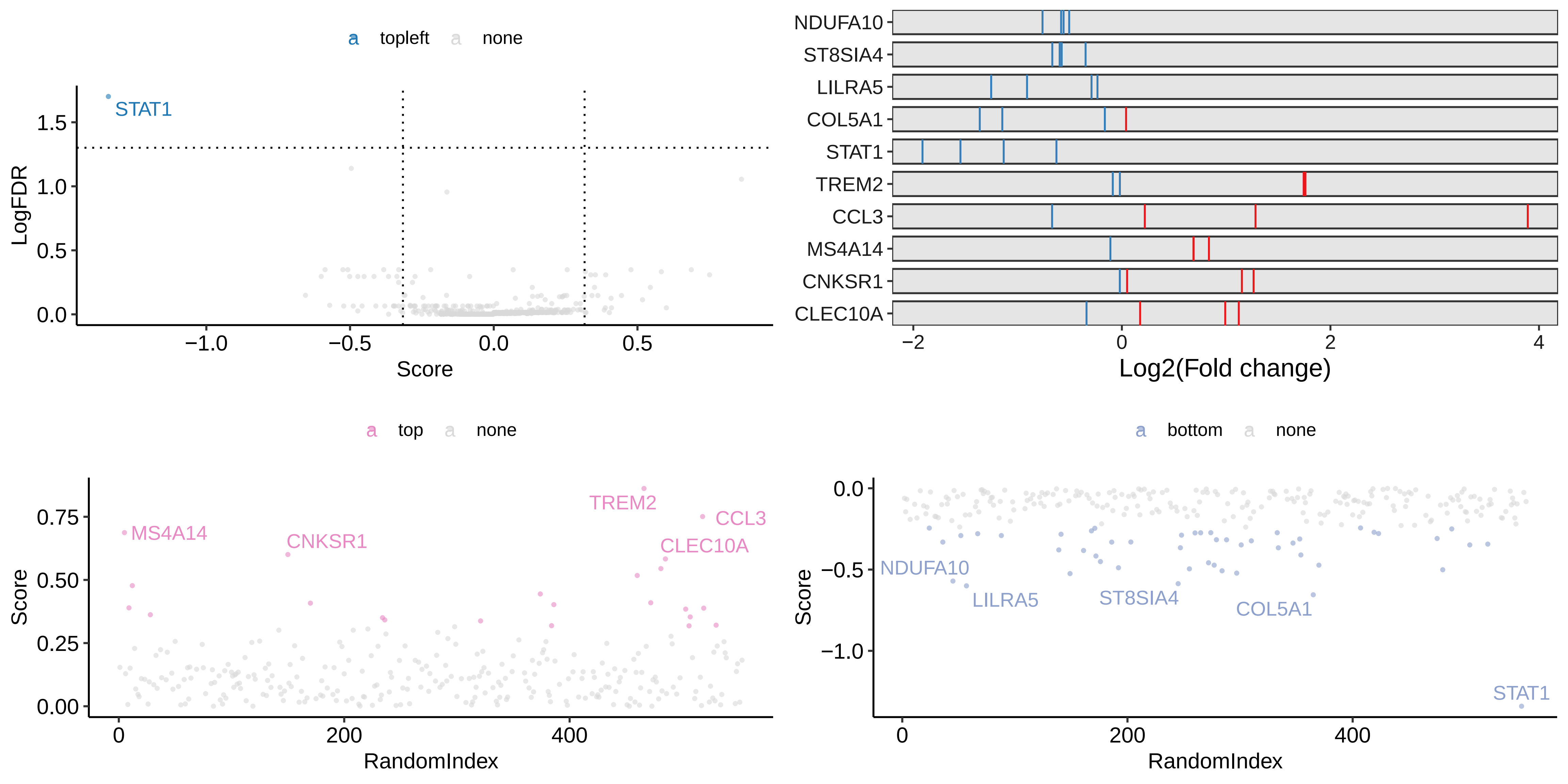

a topleft a none
STAT1
LogFDR
Score
NDUFA10
ST8SIA4
LILRA5
COL5A1
STAT1
TREM2
CCL3
MS4A14
CNKSR1
CLEC10A
Log2(Fold change)
a top a none
MS4A14
CNKSR1
TREM2
CCL3
CLEC10A
Score
RandomIndex
a bottom a none
NDUFA10
LILRA5
ST8SIA4
COL5A1
STAT1
Score
RandomIndex

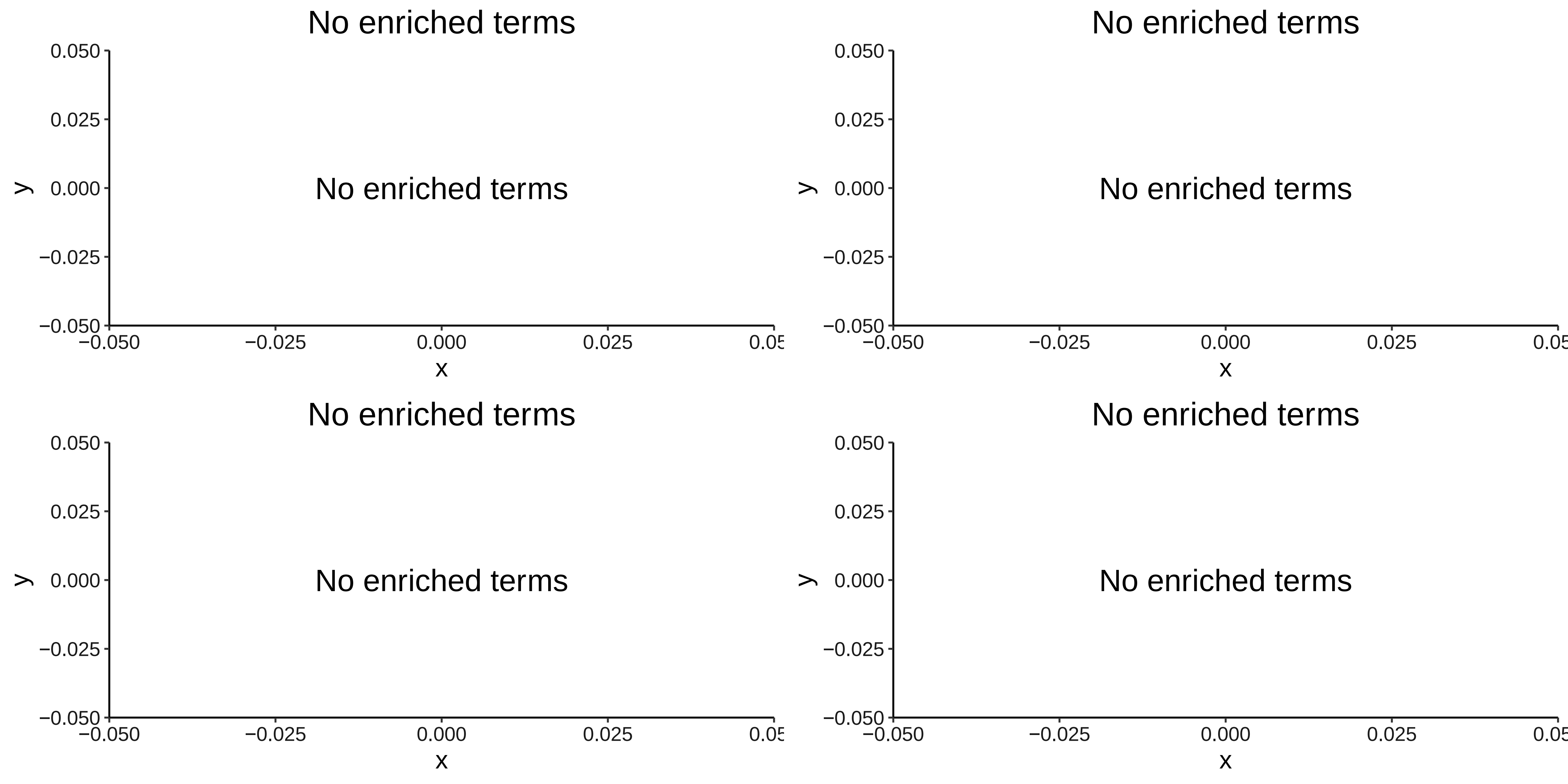
No enriched terms
No enriched terms
y
x
0.050
0.025
0.000
−0.025
−0.050
No enriched terms
No enriched terms
y
x
No enriched terms
No enriched terms
y
x
No enriched terms
No enriched terms
y
x

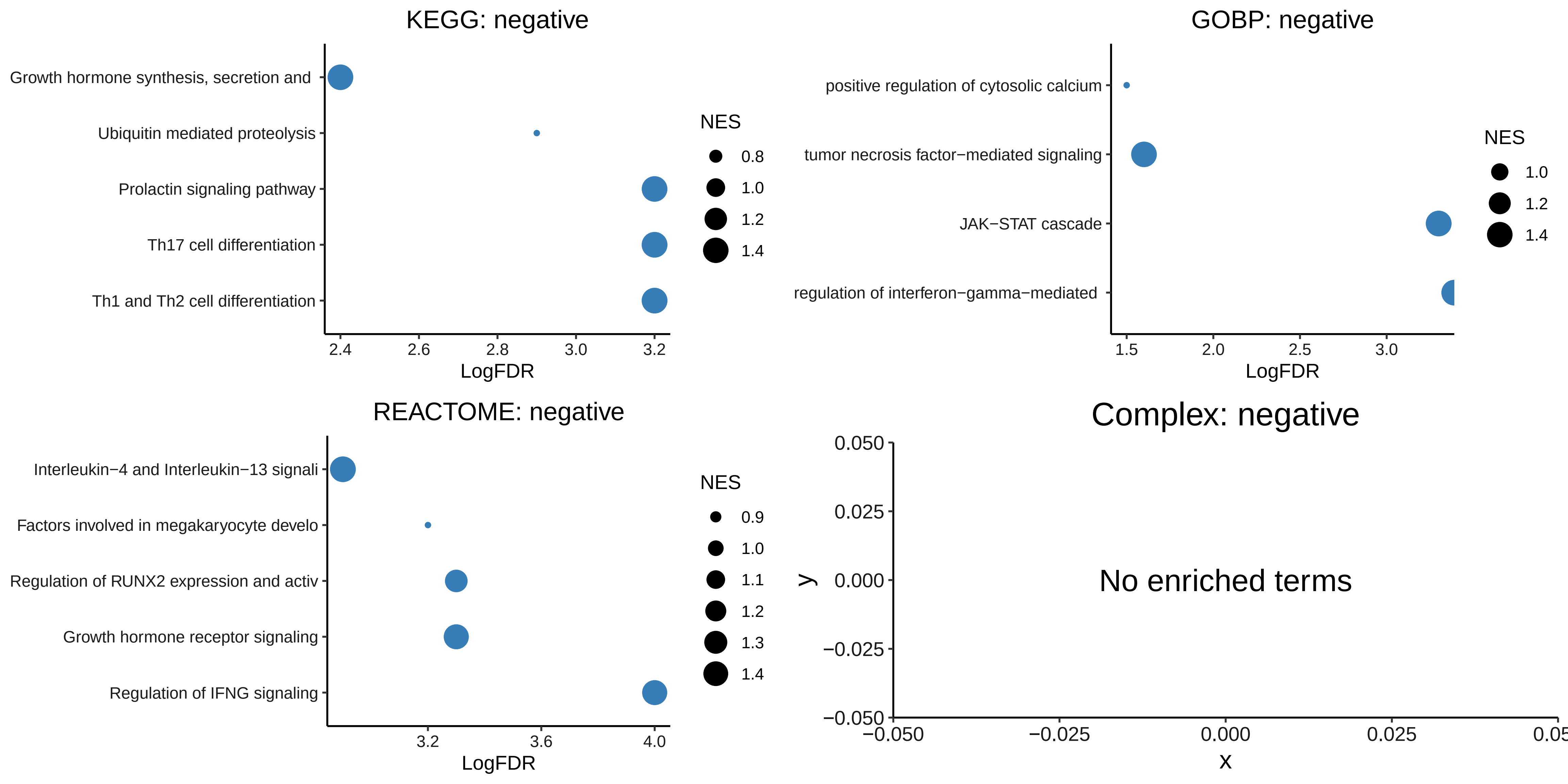
KEGG: negative
Growth hormone synthesis, secretion and
Ubiquitin mediated proteolysis
Prolactin signaling pathway
Th17 cell differentiation
Th1 and Th2 cell differentiation
2.4
2.6
2.8
3.0
3.2
LogFDR
NES
0.8
1.0
1.2
1.4
GOBP: negative
positive regulation of cytosolic calcium
tumor necrosis factor−mediated signaling
JAK−STAT cascade
regulation of interferon−gamma−mediated
1.5
2.0
2.5
3.0
LogFDR
NES
1.0
1.2
1.4
REACTOME: negative
Interleukin−4 and Interleukin−13 signali
Factors involved in megakaryocyte develo
Regulation of RUNX2 expression and activ
Growth hormone receptor signaling
Regulation of IFNG signaling
3.2
3.6
4.0
LogFDR
NES
0.9
1.0
1.1
1.2
1.3
1.4
Complex: negative
0.050
0.025
0.000
−0.025
−0.050
y
No enriched terms
−0.050
−0.025
0.000
0.025
x

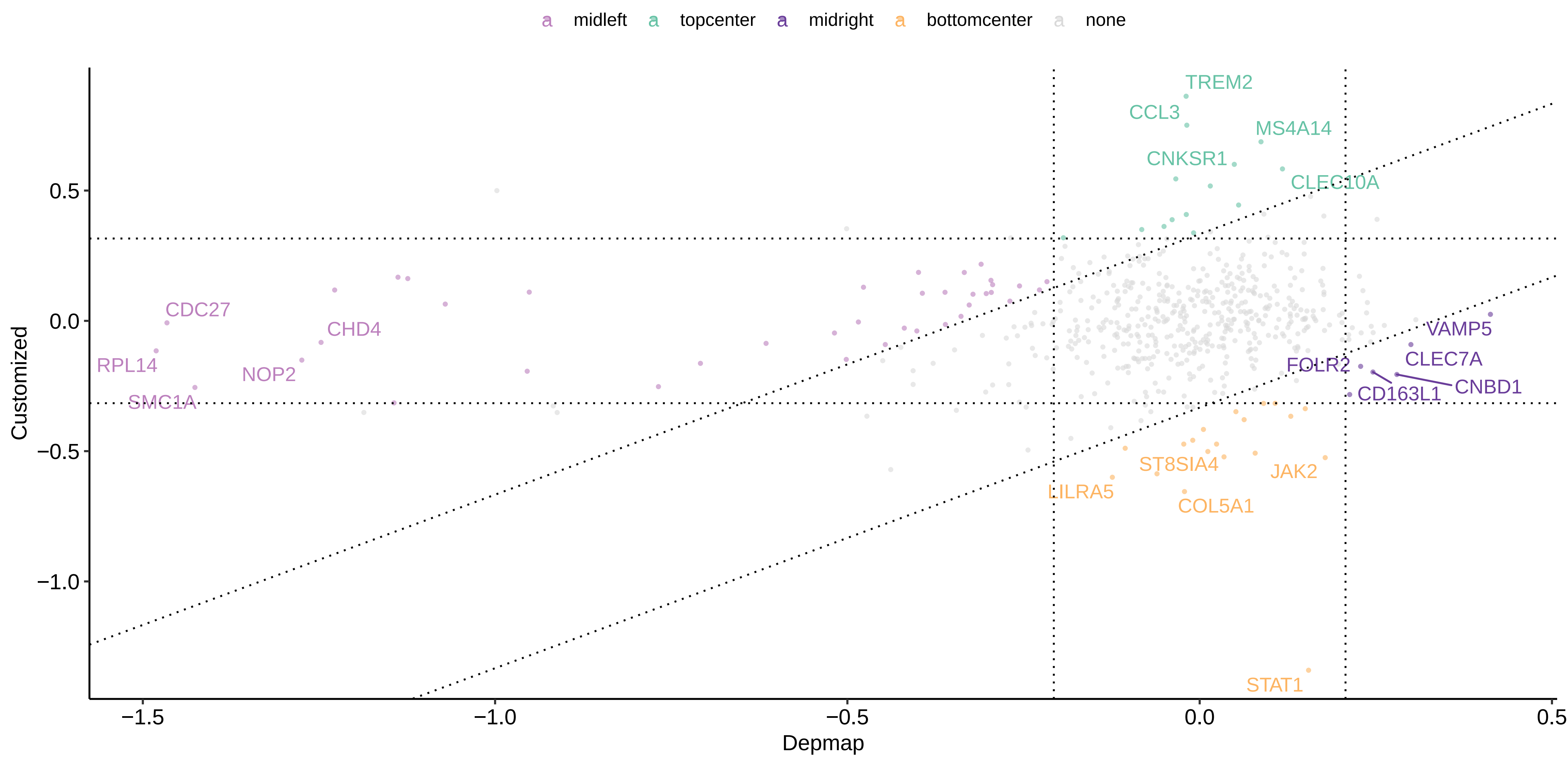

a midleft
a topcenter
a midright
a bottomcenter
a none
TREM2
CCL3
MS4A14
CNKSR1
CLEC10A
CDC27
CHD4
RPL14
NOP2
SMC1A
VAMP5
CLEC7A
FOLR2
CNBD1
CD163L1
ST8SIA4
JAK2
LILRA5
COL5A1
STAT1
0.5
0.0
−0.5
−1.0
−1.5
0.5
Customized
Depmap

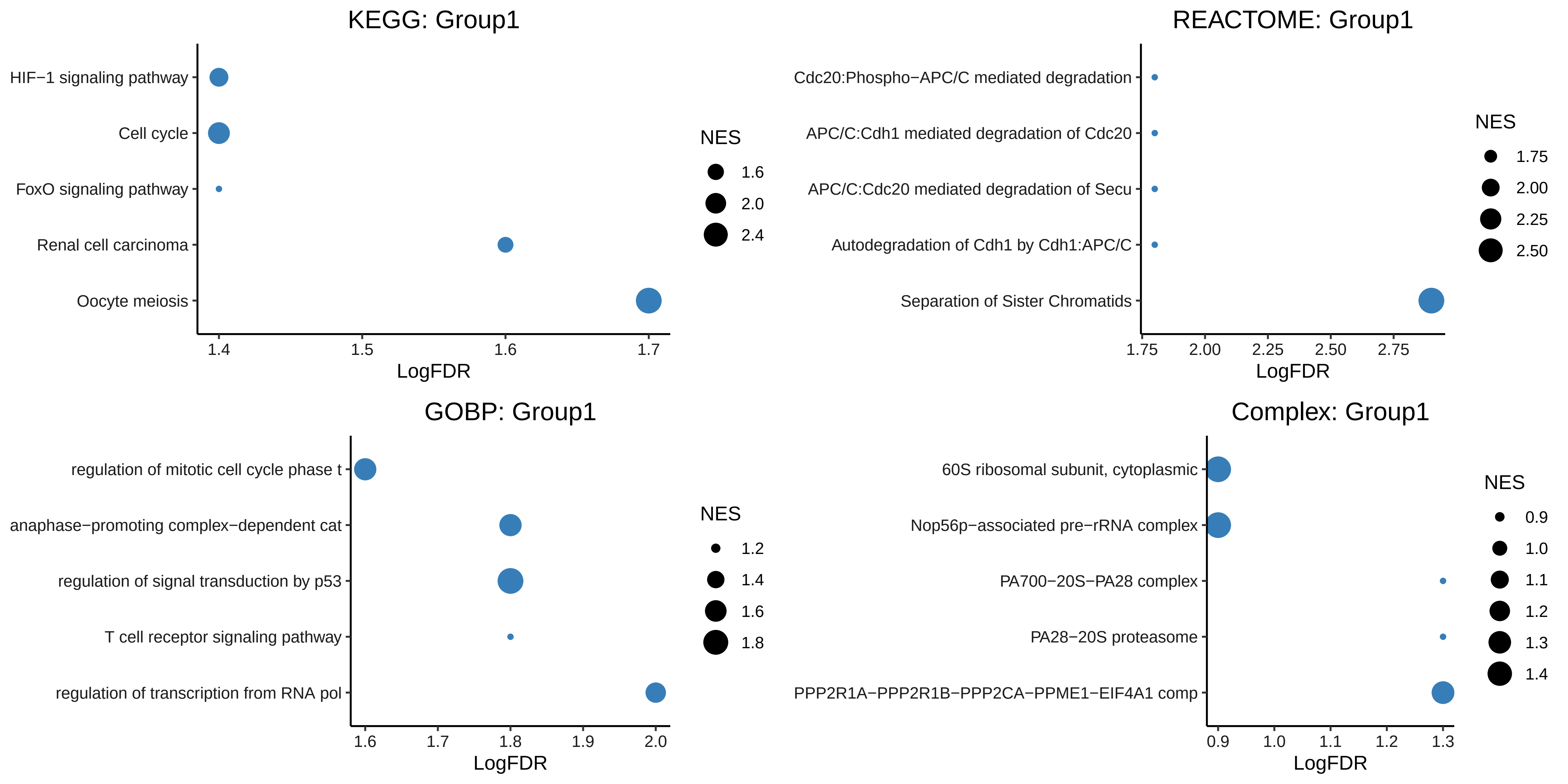
KEGG: Group1
HIF−1 signaling pathway
Cell cycle
FoxO signaling pathway
Renal cell carcinoma
Oocyte meiosis
1.4
1.5
1.6
1.7
LogFDR
NES
1.6
2.0
2.4
REACTOME: Group1
Cdc20:Phospho−APC/C mediated degradation
APC/C:Cdh1 mediated degradation of Cdc20
APC/C:Cdc20 mediated degradation of Secu
Autodegradation of Cdh1 by Cdh1:APC/C
Separation of Sister Chromatids
1.75
2.00
2.25
2.50
2.75
LogFDR
NES
1.75
2.00
2.25
2.50
GOBP: Group1
regulation of mitotic cell cycle phase t
anaphase−promoting complex−dependent cat
regulation of signal transduction by p53
T cell receptor signaling pathway
regulation of transcription from RNA pol
1.6
1.7
1.8
1.9
2.0
LogFDR
NES
1.2
1.4
1.6
1.8
Complex: Group1
60S ribosomal subunit, cytoplasmic
Nop56p−associated pre−rRNA complex
PA700−20S−PA28 complex
PA28−20S proteasome
PPP2R1A−PPP2R1B−PPP2CA−PPME1−EIF4A1 comp
0.9
1.0
1.1
1.2
1.3
LogFDR
NES
0.9
1.0
1.1
1.2
1.3
1.4

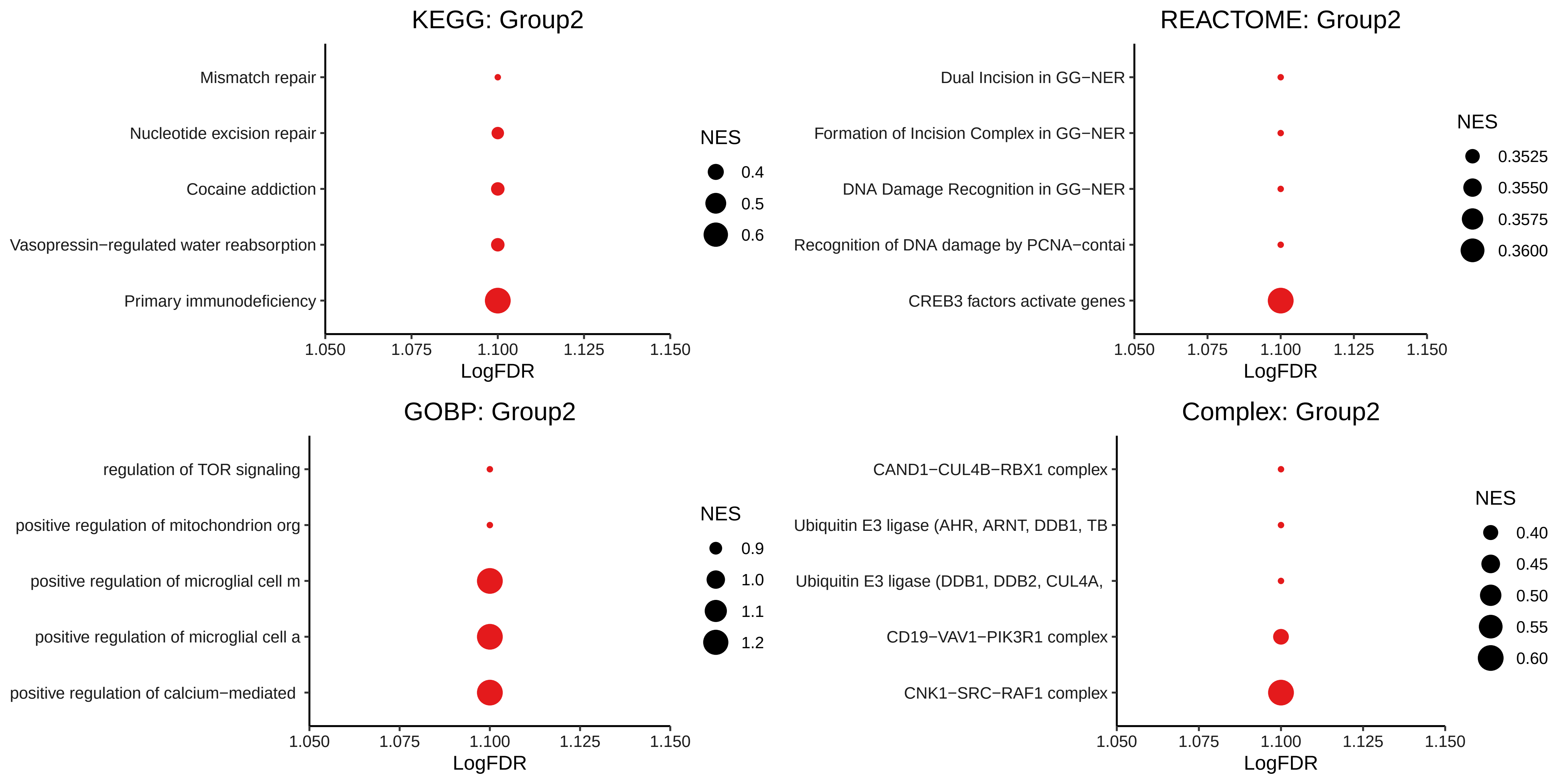

KEGG: Group2
Mismatch repair
Nucleotide excision repair
Cocaine addiction
Vasopressin−regulated water reabsorption
Primary immunodeficiency
1.050
1.075
1.100
1.125
1.150
LogFDR
NES
0.4
0.5
0.6
REACTOME: Group2
Dual Incision in GG−NER
Formation of Incision Complex in GG−NER
DNA Damage Recognition in GG−NER
Recognition of DNA damage by PCNA−contai
CREB3 factors activate genes
1.050
1.075
1.100
1.125
1.150
LogFDR
NES
0.3525
0.3550
0.3575
0.3600
GOBP: Group2
regulation of TOR signaling
positive regulation of mitochondrion org
positive regulation of microglial cell m
positive regulation of microglial cell a
positive regulation of calcium−mediated
1.050
1.075
1.100
1.125
1.150
LogFDR
NES
0.9
1.0
1.1
1.2
Complex: Group2
CAND1−CUL4B−RBX1 complex
Ubiquitin E3 ligase (AHR, ARNT, DDB1, TB
Ubiquitin E3 ligase (DDB1, DDB2, CUL4A,
CD19−VAV1−PIK3R1 complex
CNK1−SRC−RAF1 complex
1.050
1.075
1.100
1.125
1.150
LogFDR
NES
0.40
0.45
0.50
0.55
0.60

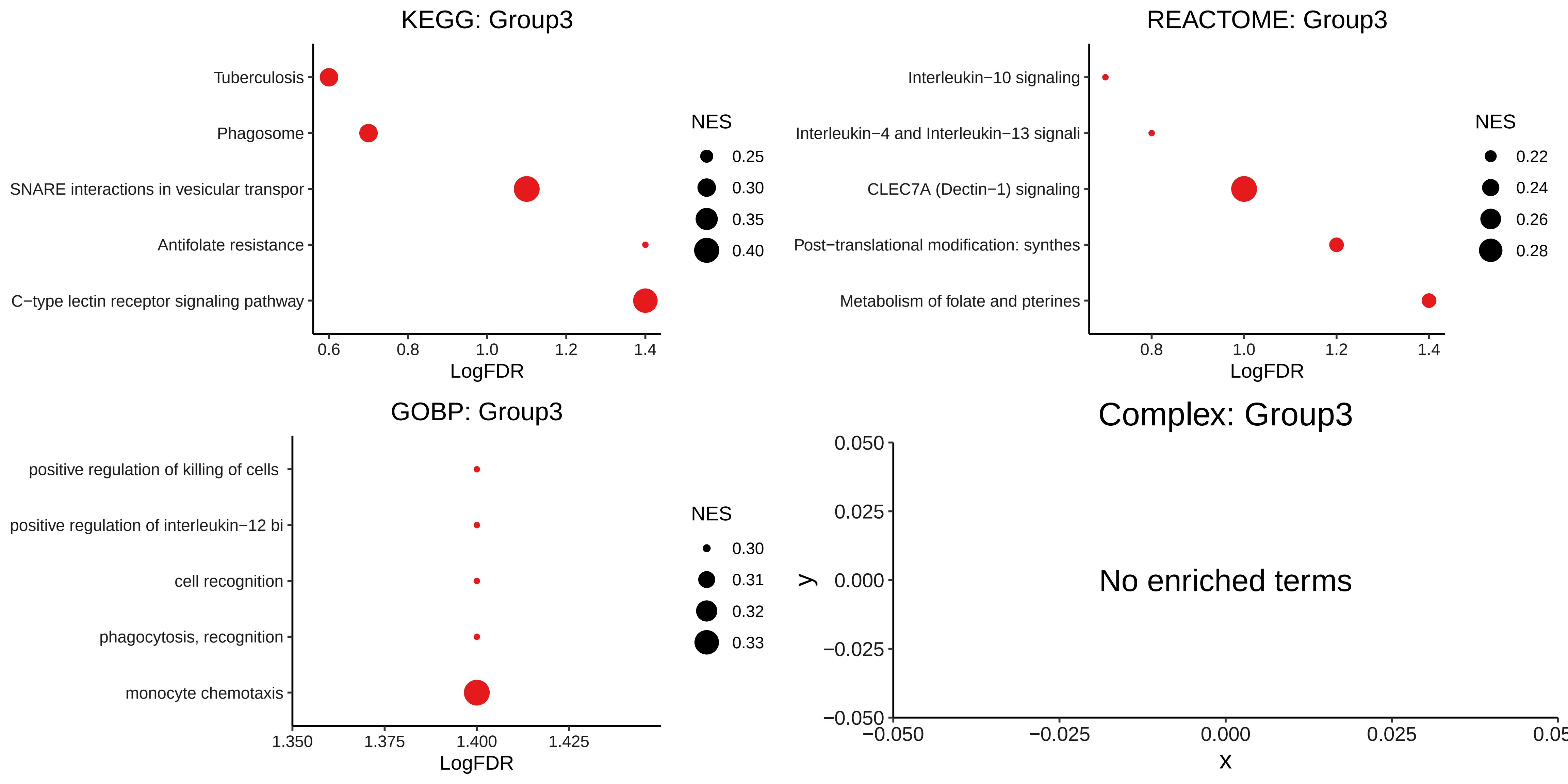
KEGG: Group3
Tuberculosis
Phagosome
SNARE interactions in vesicular transpor
Antifolate resistance
C−type lectin receptor signaling pathway
0.6
0.8
1.0
1.2
1.4
LogFDR
NES
0.25
0.30
0.35
0.40
REACTOME: Group3
Interleukin−10 signaling
Interleukin−4 and Interleukin−13 signali
CLEC7A (Dectin−1) signaling
Post−translational modification: synthes
Metabolism of folate and pterines
0.8
1.0
1.2
1.4
LogFDR
NES
0.22
0.24
0.26
0.28
GOBP: Group3
positive regulation of killing of cells
positive regulation of interleukin−12 bi
cell recognition
phagocytosis, recognition
monocyte chemotaxis
1.350
1.375
1.400
1.425
LogFDR
NES
0.30
0.31
0.32
0.33
Complex: Group3
0.050
0.025
0.000
−0.025
−0.050
y
No enriched terms
−0.050
−0.025
0.000
0.025
x

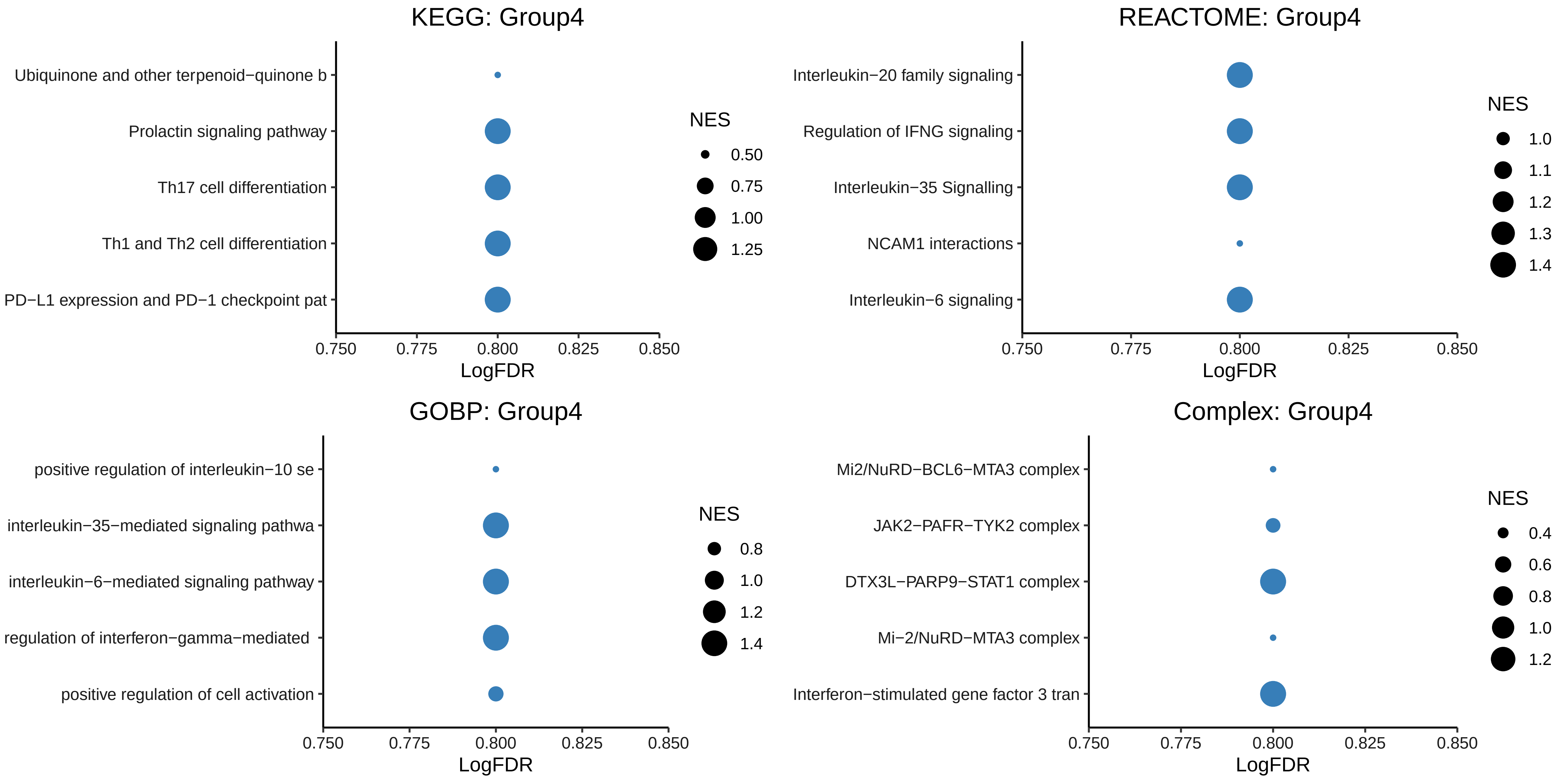
KEGG: Group4
Ubiquinone and other terpenoid−quinone b
Prolactin signaling pathway
Th17 cell differentiation
Th1 and Th2 cell differentiation
PD−L1 expression and PD−1 checkpoint pat
0.750
0.775
0.800
0.825
0.850
LogFDR
NES
0.50
0.75
1.00
1.25
REACTOME: Group4
Interleukin−20 family signaling
Regulation of IFNG signaling
Interleukin−35 Signalling
NCAM1 interactions
Interleukin−6 signaling
0.750
0.775
0.800
0.825
0.850
LogFDR
NES
1.0
1.1
1.2
1.3
1.4
GOBP: Group4
positive regulation of interleukin−10 se
interleukin−35−mediated signaling pathwa
interleukin−6−mediated signaling pathway
regulation of interferon−gamma−mediated
positive regulation of cell activation
0.750
0.775
0.800
0.825
0.850
LogFDR
NES
0.8
1.0
1.2
1.4
Complex: Group4
Mi2/NuRD−BCL6−MTA3 complex
JAK2−PAFR−TYK2 complex
DTX3L−PARP9−STAT1 complex
Mi−2/NuRD−MTA3 complex
Interferon−stimulated gene factor 3 tran
0.750
0.775
0.800
0.825
0.850
LogFDR
NES
0.4
0.6
0.8
1.0
1.2

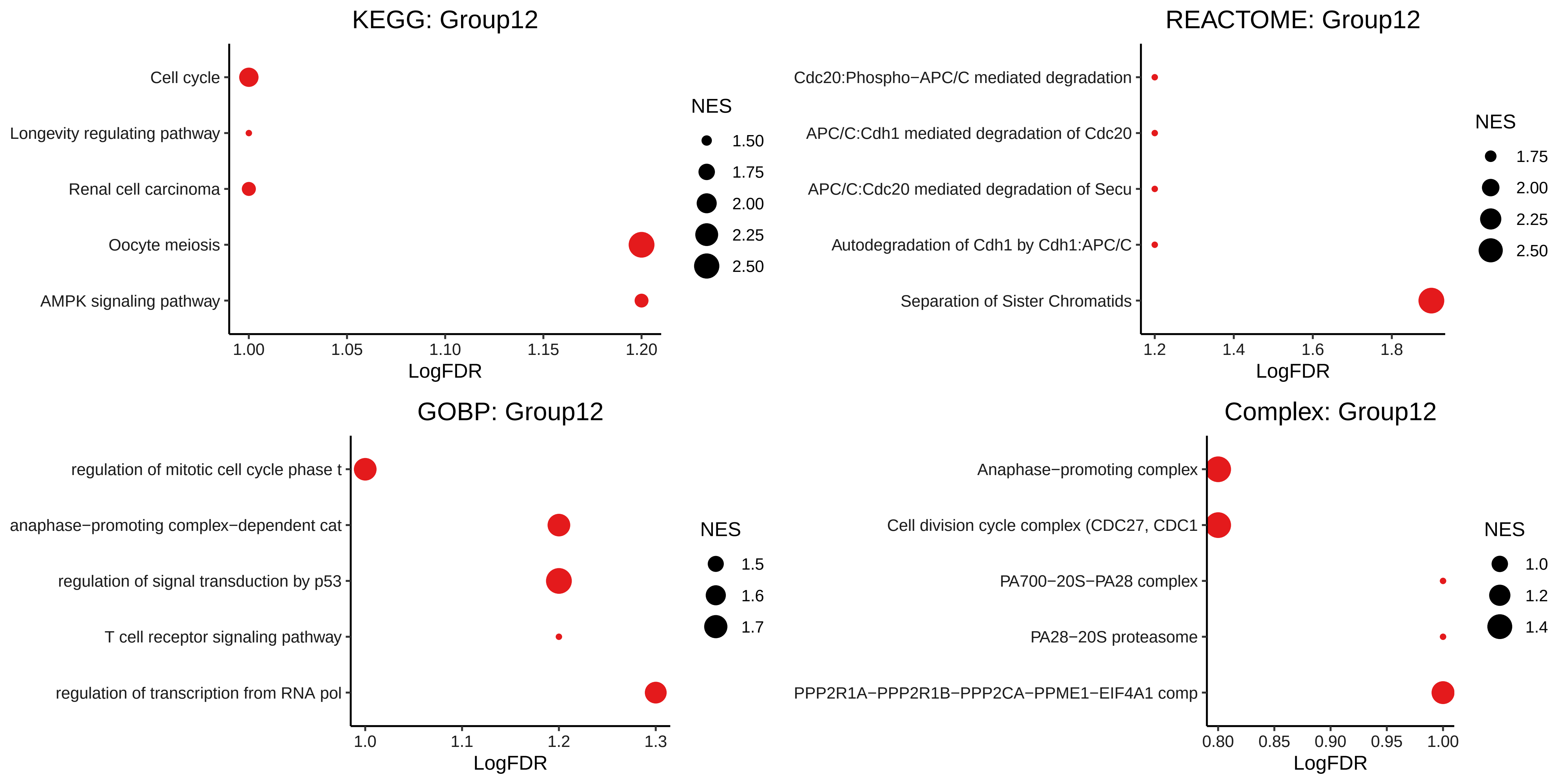
KEGG: Group12
Cell cycle
Longevity regulating pathway
Renal cell carcinoma
Oocyte meiosis
AMPK signaling pathway
1.00
1.05
1.10
1.15
1.20
LogFDR
NES
1.50
1.75
2.00
2.25
2.50
REACTOME: Group12
Cdc20:Phospho−APC/C mediated degradation
APC/C:Cdh1 mediated degradation of Cdc20
APC/C:Cdc20 mediated degradation of Secu
Autodegradation of Cdh1 by Cdh1:APC/C
Separation of Sister Chromatids
1.2
1.4
1.6
1.8
LogFDR
NES
1.75
2.00
2.25
2.50
GOBP: Group12
regulation of mitotic cell cycle phase t
anaphase−promoting complex−dependent cat
regulation of signal transduction by p53
T cell receptor signaling pathway
regulation of transcription from RNA pol
1.0
1.1
1.2
1.3
LogFDR
NES
1.5
1.6
1.7
Complex: Group12
Anaphase−promoting complex
Cell division cycle complex (CDC27, CDC1
PA700−20S−PA28 complex
PA28−20S proteasome
PPP2R1A−PPP2R1B−PPP2CA−PPME1−EIF4A1 comp
0.80
0.85
0.90
0.95
1.00
LogFDR
NES
1.0
1.2
1.4

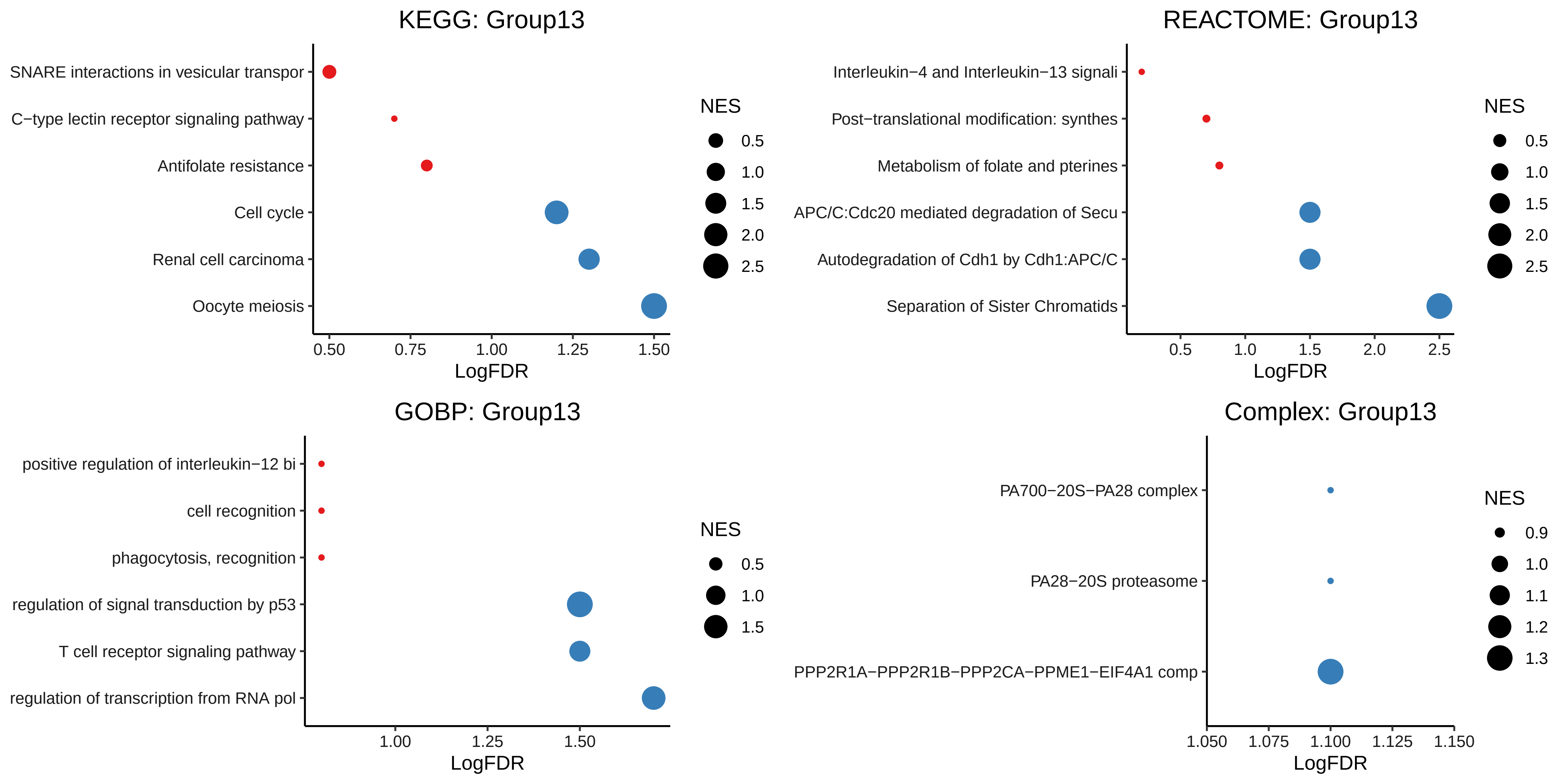
KEGG: Group13
SNARE interactions in vesicular transpor
C−type lectin receptor signaling pathway
Antifolate resistance
Cell cycle
Renal cell carcinoma
Oocyte meiosis
0.50
0.75
1.00
1.25
1.50
LogFDR
NES
0.5
1.0
1.5
2.0
2.5
REACTOME: Group13
Interleukin−4 and Interleukin−13 signali
Post−translational modification: synthes
Metabolism of folate and pterines
APC/C:Cdc20 mediated degradation of Secu
Autodegradation of Cdh1 by Cdh1:APC/C
Separation of Sister Chromatids
0.5
1.0
1.5
2.0
2.5
LogFDR
NES
0.5
1.0
1.5
2.0
2.5
GOBP: Group13
positive regulation of interleukin−12 bi
cell recognition
phagocytosis, recognition
regulation of signal transduction by p53
T cell receptor signaling pathway
regulation of transcription from RNA pol
1.00
1.25
1.50
LogFDR
NES
0.5
1.0
1.5
Complex: Group13
PA700−20S−PA28 complex
PA28−20S proteasome
PPP2R1A−PPP2R1B−PPP2CA−PPME1−EIF4A1 comp
1.050
1.075
1.100
1.125
1.150
LogFDR
NES
0.9
1.0
1.1
1.2
1.3

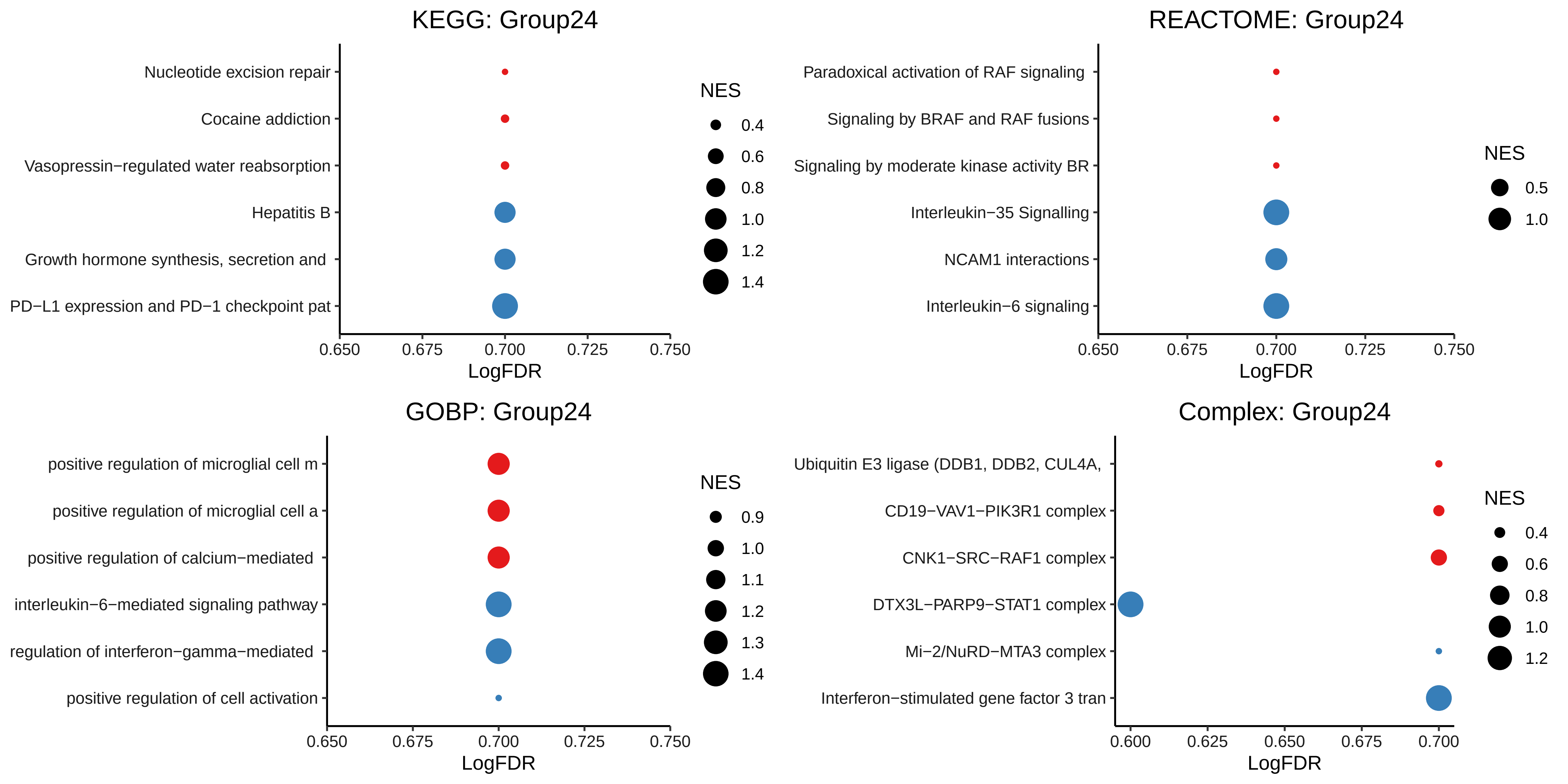

KEGG: Group24
Nucleotide excision repair
Cocaine addiction
Vasopressin−regulated water reabsorption
Hepatitis B
Growth hormone synthesis, secretion and
PD−L1 expression and PD−1 checkpoint pat
0.650
0.675
0.700
0.725
0.750
LogFDR
NES
0.4
0.6
0.8
1.0
1.2
1.4
REACTOME: Group24
Paradoxical activation of RAF signaling
Signaling by BRAF and RAF fusions
Signaling by moderate kinase activity BR
Interleukin−35 Signalling
NCAM1 interactions
Interleukin−6 signaling
0.650
0.675
0.700
0.725
0.750
LogFDR
NES
0.5
1.0
GOBP: Group24
positive regulation of microglial cell m
positive regulation of microglial cell a
positive regulation of calcium−mediated
interleukin−6−mediated signaling pathway
regulation of interferon−gamma−mediated
positive regulation of cell activation
0.650
0.675
0.700
0.725
0.750
LogFDR
NES
0.9
1.0
1.1
1.2
1.3
1.4
Complex: Group24
Ubiquitin E3 ligase (DDB1, DDB2, CUL4A,
CD19−VAV1−PIK3R1 complex
CNK1−SRC−RAF1 complex
DTX3L−PARP9−STAT1 complex
Mi−2/NuRD−MTA3 complex
Interferon−stimulated gene factor 3 tran
0.600
0.625
0.650
0.675
0.700
LogFDR
NES
0.4
0.6
0.8
1.0
1.2

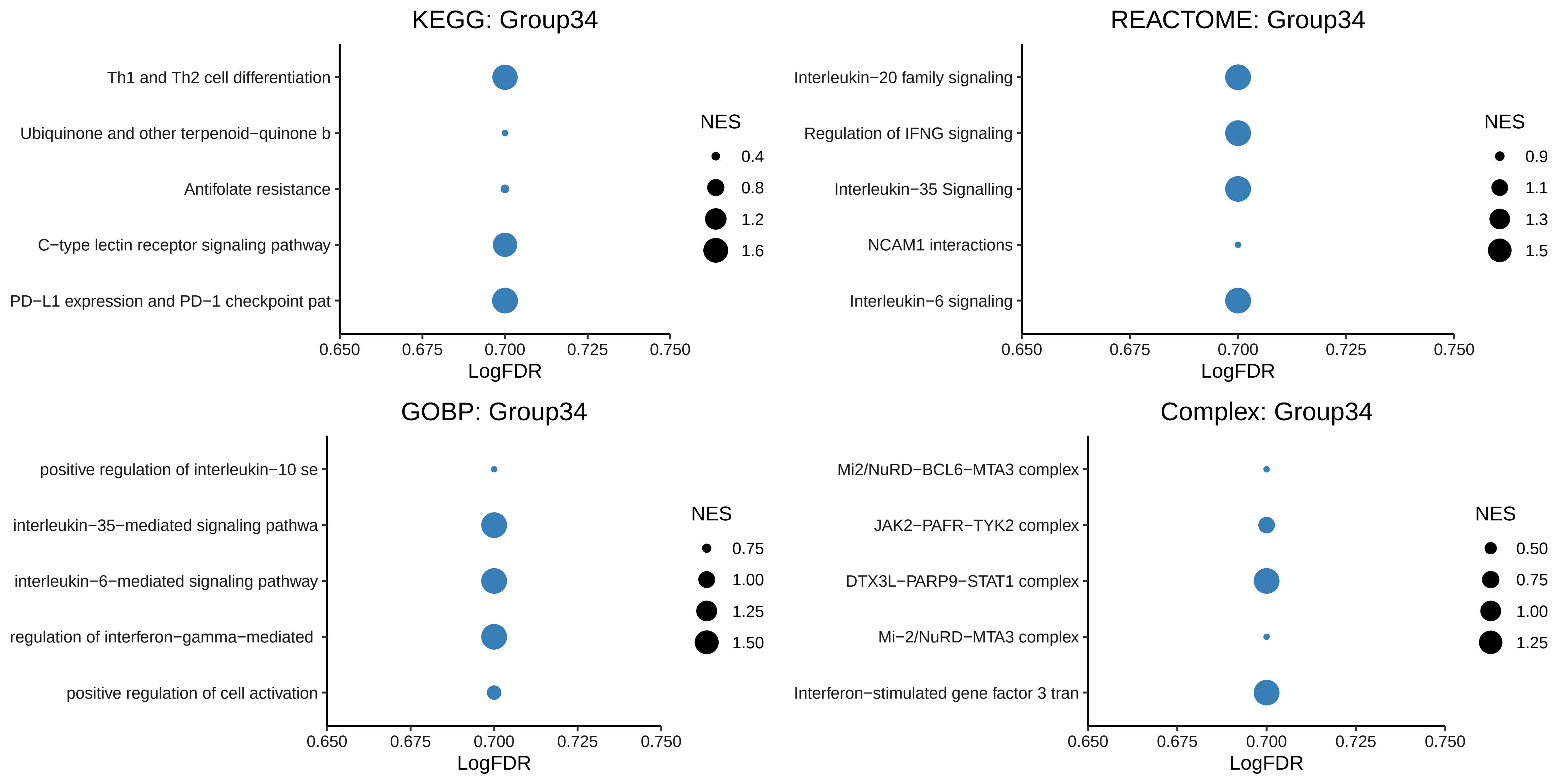
KEGG: Group34
Th1 and Th2 cell differentiation
Ubiquinone and other terpenoid−quinone b
Antifolate resistance
C−type lectin receptor signaling pathway
PD−L1 expression and PD−1 checkpoint pat
0.650
0.675
0.700
0.725
0.750
LogFDR
NES
0.4
0.8
1.2
1.6
REACTOME: Group34
Interleukin−20 family signaling
Regulation of IFNG signaling
Interleukin−35 Signalling
NCAM1 interactions
Interleukin−6 signaling
0.650
0.675
0.700
0.725
0.750
LogFDR
NES
0.9
1.1
1.3
1.5
GOBP: Group34
positive regulation of interleukin−10 se
interleukin−35−mediated signaling pathwa
interleukin−6−mediated signaling pathway
regulation of interferon−gamma−mediated
positive regulation of cell activation
0.650
0.675
0.700
0.725
0.750
LogFDR
NES
0.75
1.00
1.25
1.50
Complex: Group34
Mi2/NuRD−BCL6−MTA3 complex
JAK2−PAFR−TYK2 complex
DTX3L−PARP9−STAT1 complex
Mi−2/NuRD−MTA3 complex
Interferon−stimulated gene factor 3 tran
0.650
0.675
0.700
0.725
0.750
LogFDR
NES
0.50
0.75
1.00
1.25
