# Supplementary material for: In vivo CRISPR screens reveal Serpinb9 and Adam2 as regulators of immune therapy response in lung cancer
Source: Nat Commun. 2023 May 31;14:3150. doi: 10.1038/s41467-023-38841-7 (PMC10232477; doi:10.1038/s41467-023-38841-7)
Supplement: Supplementary file 13 — Source Data [file 41467_2023_38841_MOESM13_ESM.zip › Figure 1F.zip!/Lungs_sgRNA analysis_KRAS/Lung_down.pdf]

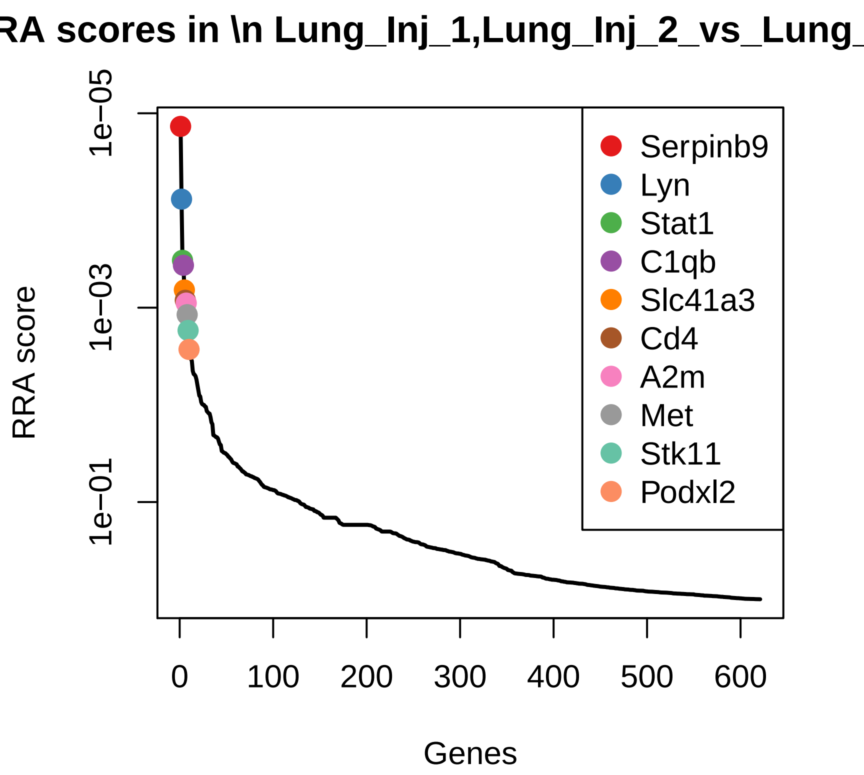
RRA scores in \n Lung_Inj_1,Lung_Inj_2_vs_Lung_
RRA score
1e−05
1e−03
1e−01
0
100
200
300
400
500
600
Genes
Serpinb9
Lyn
Stat1
C1qb
Slc41a3
Cd4
A2m
Met
Stk11
Podxl2

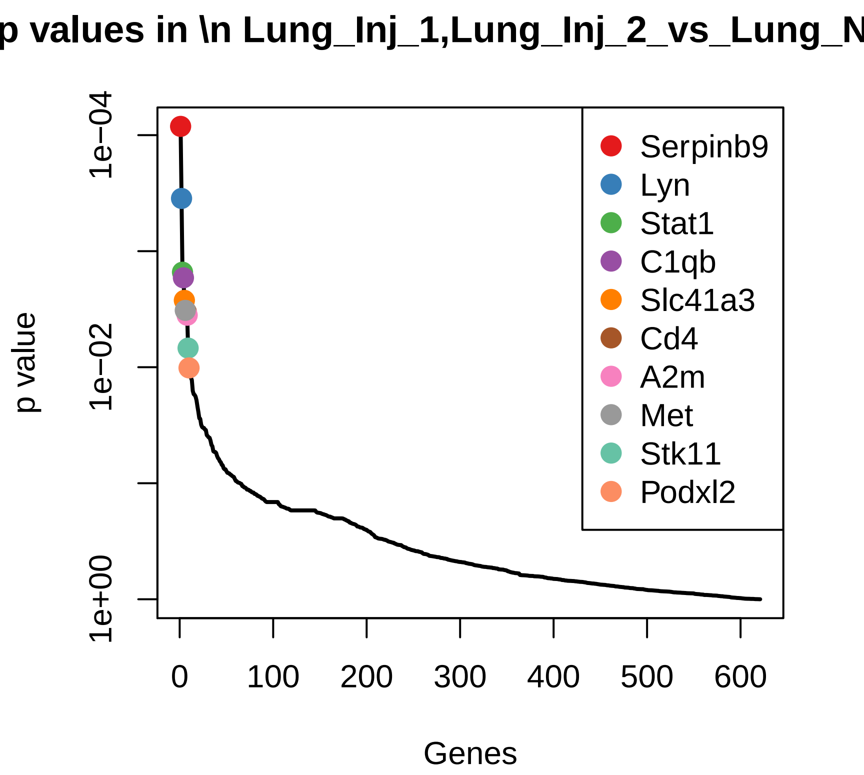

p values in \n Lung_Inj_1,Lung_Inj_2_vs_Lung_N
p value
1e−04
1e−02
1e+00
0
100
200
300
400
500
600
Genes
Serpinb9
Lyn
Stat1
C1qb
Slc41a3
Cd4
A2m
Met
Stk11
Podxl2

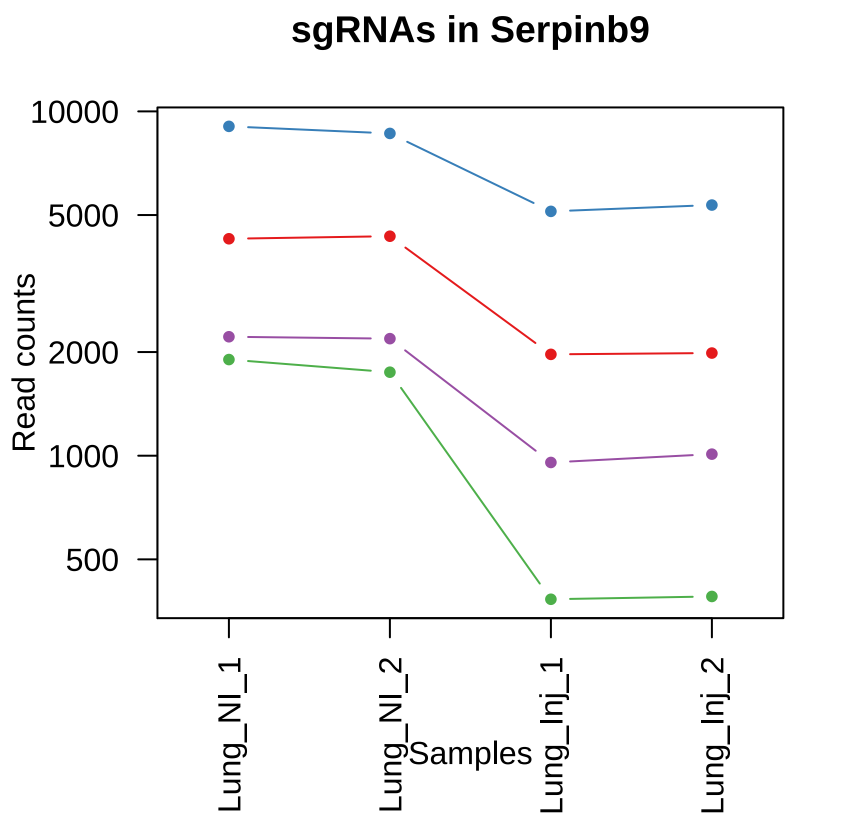

sgRNAs in Serpinb9
Read counts
10000
5000
2000
1000
500
Lung_NI_1
Lung_NI_2
Lung_Inj_1
Lung_Inj_2
Samples

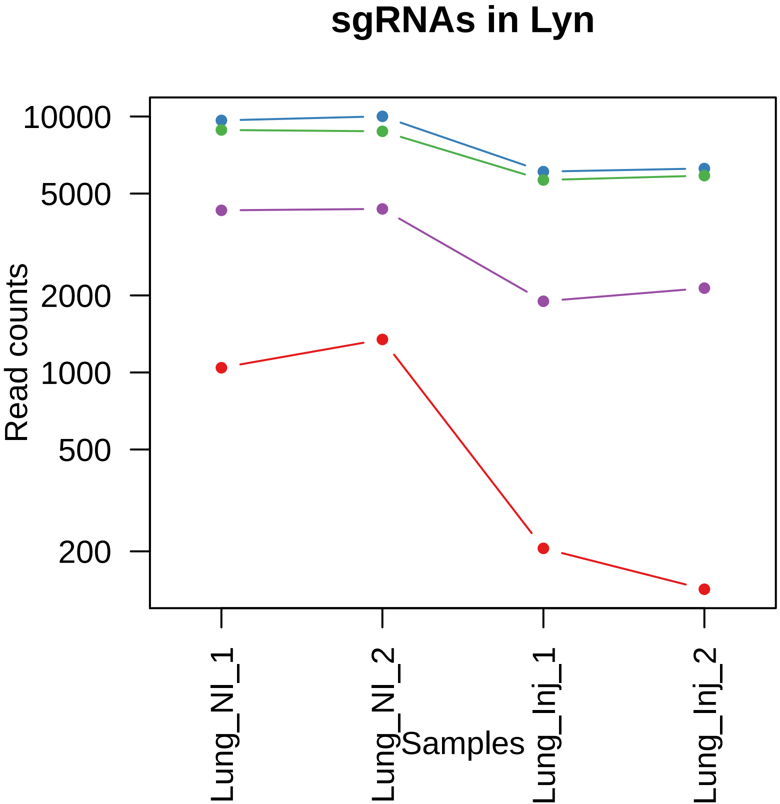

sgRNAs in Lyn
Read counts
10000
5000
2000
1000
500
200
Lung_NI_1
Lung_NI_2
Lung_Inj_1
Lung_Inj_2
Samples

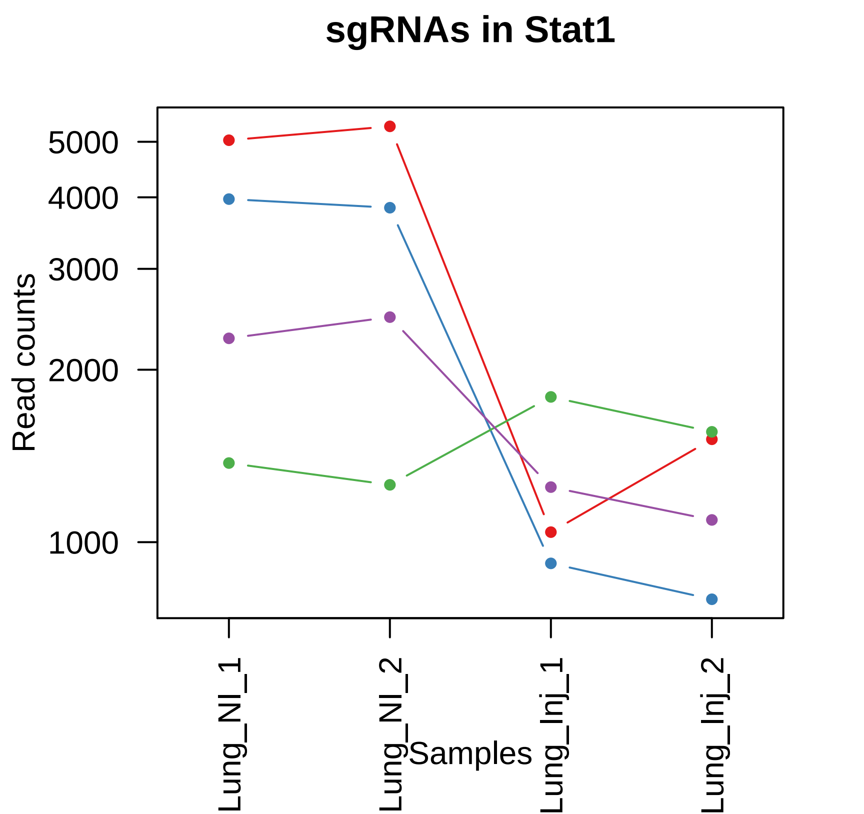

sgRNAs in Stat1
Read counts
5000
4000
3000
2000
1000
Lung_NI_1
Lung_NI_2
Lung_Inj_1
Lung_Inj_2
Samples

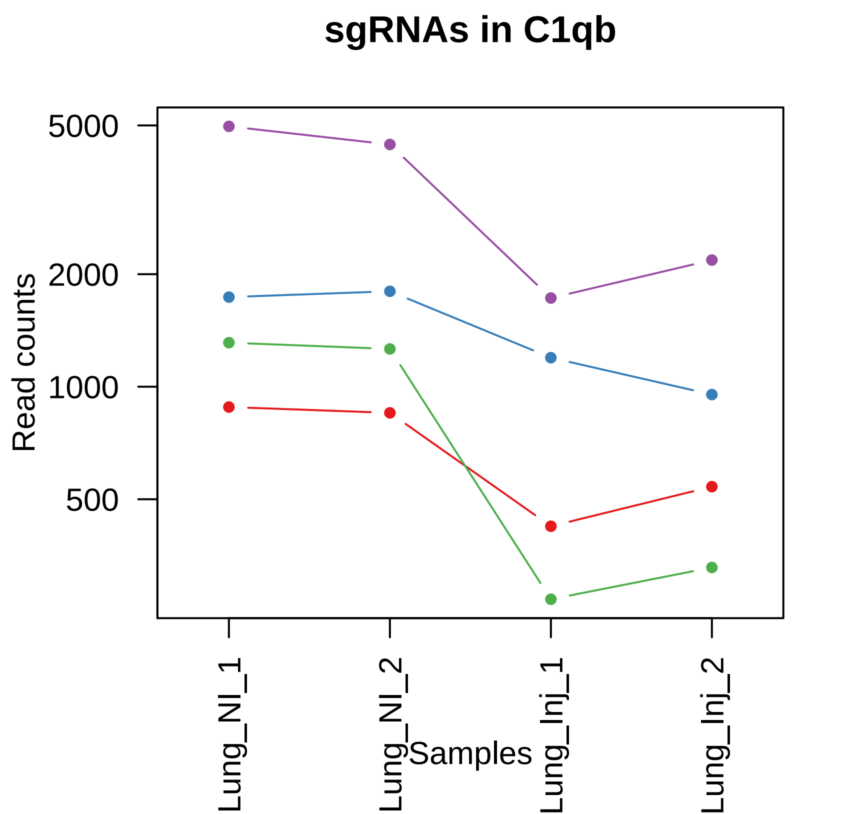
sgRNAs in C1qb
Read counts
5000
2000
1000
500
Lung_NI_1
Lung_NI_2
Lung_Inj_1
Lung_Inj_2
Samples

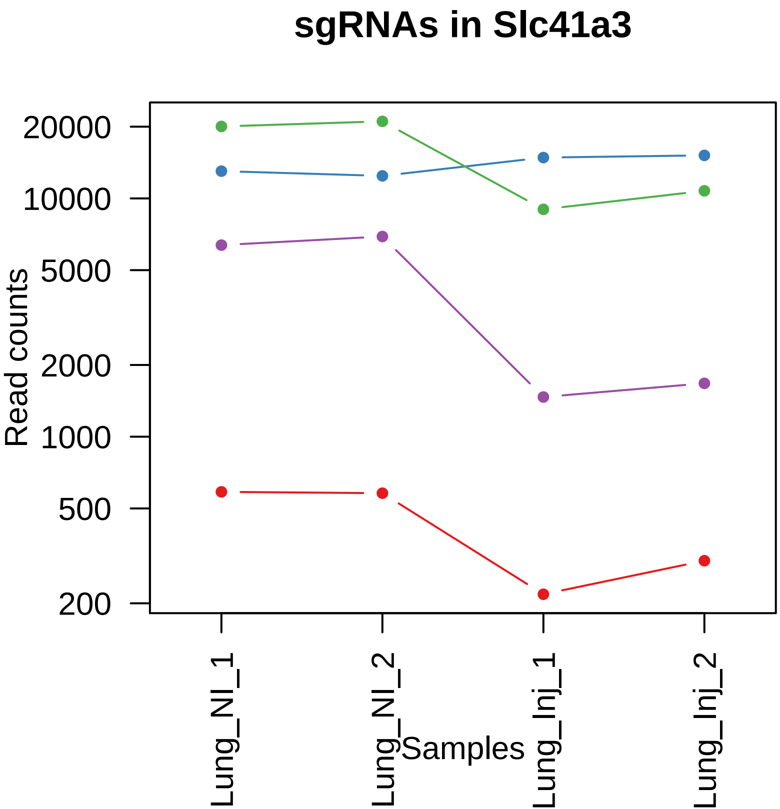

sgRNAs in Slc41a3
Read counts
20000
10000
5000
2000
1000
500
200
Lung_NI_1
Lung_NI_2
Lung_Inj_1
Lung_Inj_2
Samples

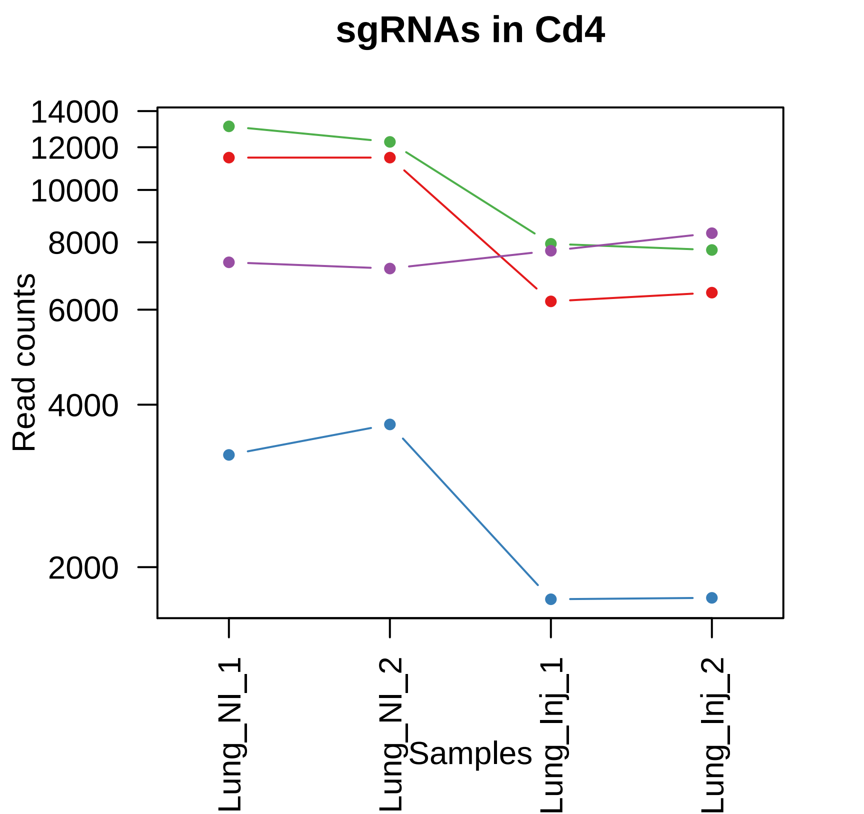

sgRNAs in Cd4
14000
12000
10000
8000
6000
4000
2000
Read counts
Lung_NI_1
Lung_NI_2
Lung_Inj_1
Lung_Inj_2
Samples

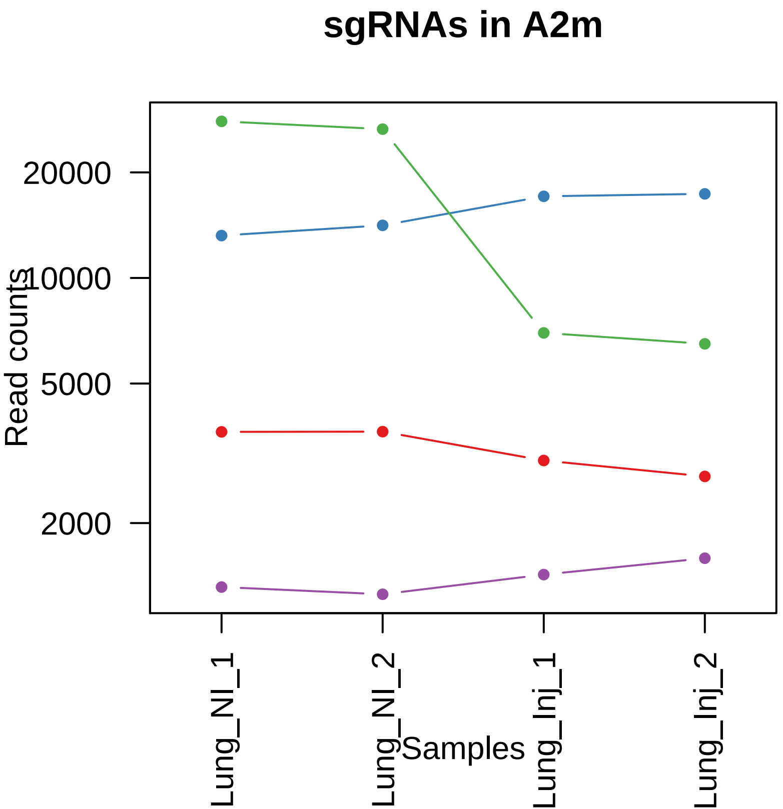

sgRNAs in A2m
Read counts
20000
10000
5000
2000
Lung_NI_1
Lung_NI_2
Lung_Inj_1
Lung_Inj_2
Samples

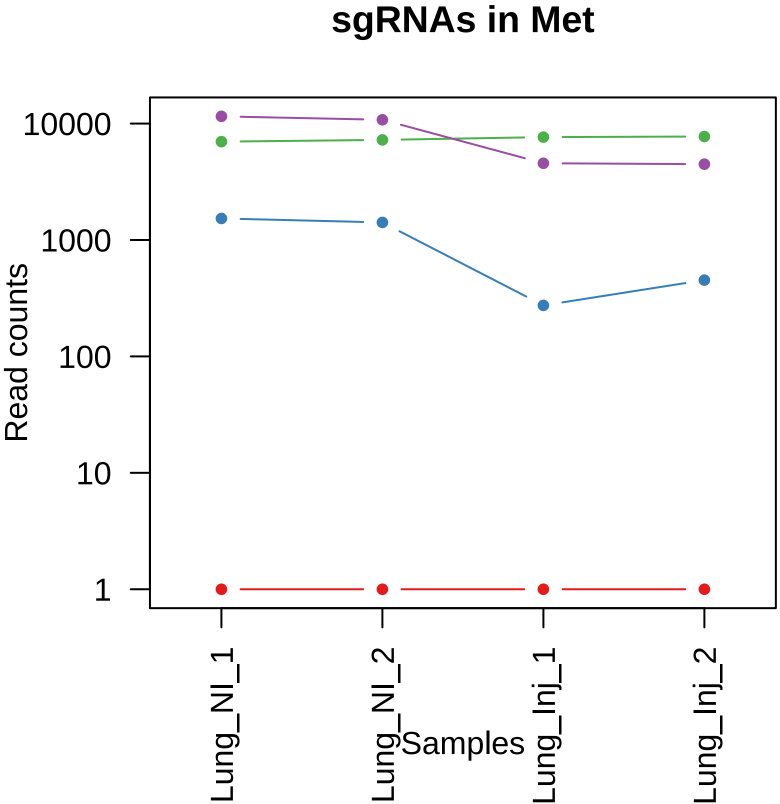

sgRNAs in Met
Read counts
10000
1000
100
10
1
Lung_NI_1
Lung_NI_2
Lung_Inj_1
Lung_Inj_2
Samples

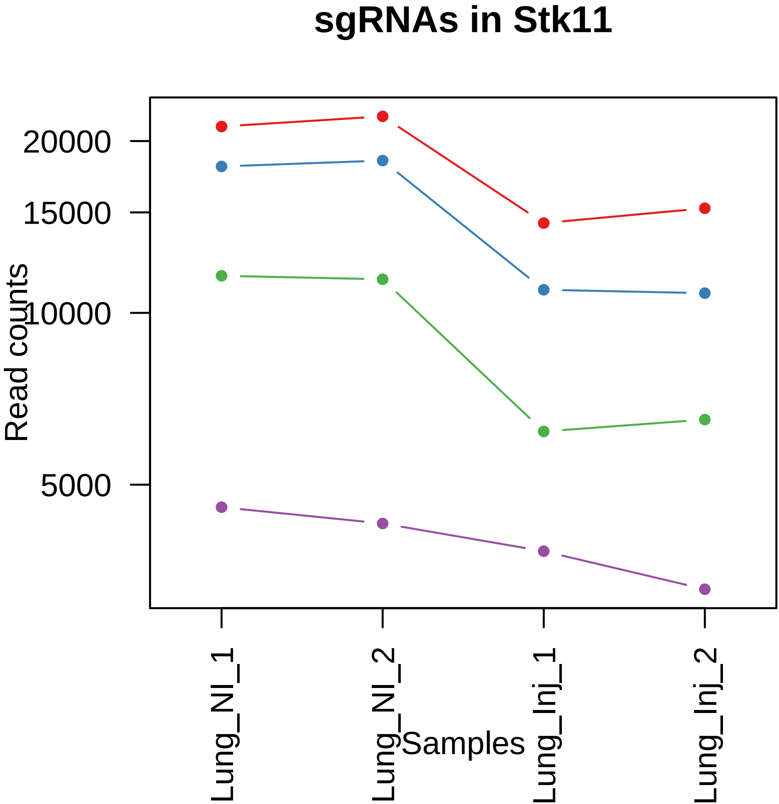
sgRNAs in Stk11
Read counts
20000
15000
10000
5000
Lung_NI_1
Lung_NI_2
Lung_Inj_1
Lung_Inj_2
Samples

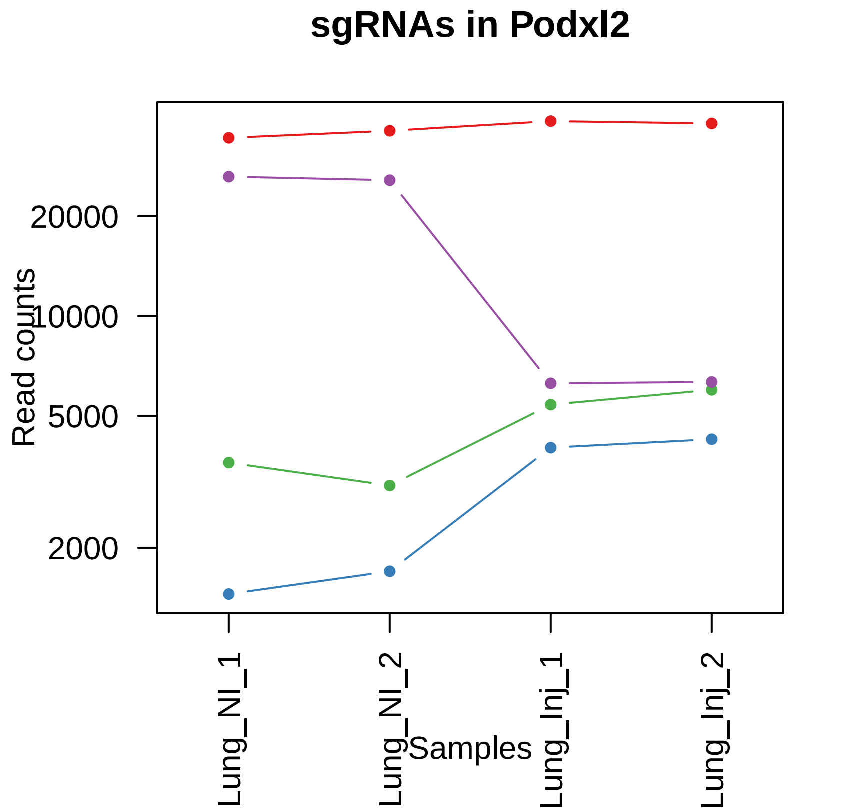
sgRNAs in Podxl2
Read counts
20000
10000
5000
2000
Lung_NI_1
Lung_NI_2
Lung_Inj_1
Lung_Inj_2
Samples
